# Recent admixture between species of the fungal pathogen *Histoplasma*

**DOI:** 10.1002/evl3.59

**Published:** 2018-06-22

**Authors:** Colin S. Maxwell, Victoria E. Sepulveda, David A. Turissini, William E. Goldman, Daniel R. Matute

**Affiliations:** ^1^ Biology Department University of North Carolina Chapel Hill North Carolina 27599; ^2^ Department of Microbiology and Immunology, School of Medicine University of North Carolina Chapel Hill North Carolina 27599

**Keywords:** Gene exchange, Hidden Markov Model (HMM), introgression, speciation

## Abstract

Hybridization between species of pathogens has the potential to speed evolution of virulence by providing the raw material for adaptation through introgression or by assembling new combinations of virulence traits. Fungal diseases are a source high morbidity, and remain difficult to treat. Yet the frequency of hybridization between fungal species has rarely been explored, and the functional role of introgressed alleles remains largely unknown. *Histoplasma mississippiense* and *H. ohiense* are sympatric throughout their range in North America and have distinct virulence strategies, making them an ideal system to examine the role introgression may play in fungal pathogens. We identified introgressed tracts in the genomes of a sample of *H. mississippiense* and *H. ohiense* isolates. We found strong evidence in each species for recent admixture, but introgressed alleles were present at low frequencies, suggesting that they were deleterious. Consistent with this, coding and regulatory sequences were strongly depleted within introgressed regions, whereas intergenic regions were enriched, indicating that functional introgressed alleles were frequently deleterious in their new genomic context. Surprisingly, we found only two isolates with substantial admixture: the *H. mississippiense* and *H. ohiense* genomic reference strains, WU24 and G217B, respectively. Our results show that recent admixture has occurred, that it is frequently deleterious and that conclusions based on studies of the *H. mississippiense* and *H. ohiense* type strains should be revisited with more representative samples from the genus.

**Impact Summary**

Fungal diseases are difficult to treat since their cell biology is more similar to humans than bacteria. This means that the potential for new fungal diseases is particularly troubling. Hybridization between two closely related species of fungi could create a new fungal disease with the virulence traits of each species, but we do not know in which species this is likely to happen. This study used a population genetic method to analyze the genomes of individuals from two species of closely related fungi in the genus *Histoplasma* that coexist in North America to look for evidence that they mated in the past. Our data show that the species hybridized recently, which means that there is the potential for a dangerous hybrid to emerge in the future.

The extent to which differentiated species exchange genes when they are in contact likely depends on a number of factors including the taxa, the geographical distribution of the species, and the divergence time between the potentially hybridizing species. However, our knowledge of this aspect of speciation genetics is generally lacking in fungi. Most—but not all—studies investigating gene flow across fungal species have done so by using low resolution markers (e.g. (Devier et al. 2017; Gladieux et al. 2017; Heil et al. 2017) reviewed in (Schardl and Craven 2003; Stukenbrock 2013, 2016)). Although these studies provided evidence consistent with introgression, they are unable to rule out alternative explanations for tracts of shared ancestry and cannot determine which alleles have crossed species boundaries. The use of population‐level sampling and whole‐genome sequences can address these limitations.

Introgression and gene exchange may be important for understanding the emergence of virulence in pathogens and closely related species. There are at least two scenarios whereby gene flow across species boundaries can influence the evolution of virulence. First, species that are disease agents can potentially exchange genes with nonpathogenic species through hybridization. Genetic variation that is introduced into a population through gene exchange could then act as the raw material for adaptation, and could even transmit virulence traits directly from one species to another (Coyne and Orr 2004; Arnold 2006). Second, if closely related species of pathogens differ in their virulence strategies, then introgression could lead to hybrids with the virulence factors of both species. Thus, quantifying the magnitude of gene exchange between closely related species is at the interface of speciation genetics and the molecular genetics of virulence, and has the potential to inform our understanding of the dynamics of the evolution of virulence. Species from the genus *Histoplasma* are an ideal system to investigate the possible role of introgression on pathogen evolution because they are closely related enough to exchange genes, diverged enough to enable introgression to be detected, and differ in their virulence strategies (Kasuga et al. 1999; Sepúlveda et al. 2014, 2017).

*Histoplasma* is a genus of dimorphic fungi. *Histoplasma* species are the causal agents of histoplasmosis, a lung disease with a high morbidity around the world (Knox 2014). Histoplasmosis is one of the most common pulmonary diseases, with hundreds of thousands of new infections occurring annually in the United States alone. Histoplasmosis is particularly common in immunosuppressed patients; up to 25% of the HIV‐positive population develops histoplasmosis, which frequently turns fatal (Knox 2014; Hage et al. 2015; Wheat et al. 2016). *Histoplasma* is an ideal model system to understand dimorphic fungal pathogens. The yeast stage is unicellular, uninucleate, and haploid, making it amenable to a broad range of molecular genetic strategies (Carr and Shearer 1998; Woods et al. 1998; Rappleye et al. 2004; Sepúlveda et al. 2014). Hypotheses generated by genomic studies can be functionally validated in *Histoplasma* using gene disruption or RNAi, and fine scale population genetics can be applied in a way that is challenging in diploid organisms. Furthermore, mice are natural hosts for *Histoplasma* and can be used for experimental infections to test virulence of genetically manipulated strains (Ajello and Runyon 1953; Williams et al. 1978; Sebghati et al. 2000). Thus, *Histoplasma* is considered one of the premier model genetic system for fungal pathogenesis.

*Histoplasma* is composed of at least four species but it is likely to harbor more (Kasuga et al. 1999, 2003; Balajee et al. 2013; Sepúlveda et al. 2017). A highly divergent African lineage seems to have diverged from all the four endemic American species over 10 mya (Sepúlveda et al. 2017). One of these species, *H. suramericanum*, is restricted to South America (Sepúlveda et al. 2017). *Histoplasma capsulatum sensu stricto* is found in Panama and the northern portion of South America. The remaining two species, *H. ohiense* and *H. mississippiense*, coexist in North America—a region of the world with numerous reported cases of histoplasmosis (Graybill 1988; Wheat 2003; Freifeld et al. 2005; Hage et al. 2015). These latter two species provide an unprecedented opportunity to assess the possibility of gene exchange between fungal pathogens because they are fully sympatric across their whole range, are the most closely related species of *Histoplasma*, and are one of the most recently diverged species pairs of pathogenic fungi. Furthermore, they strongly differ in their virulence strategies and resistance to antifungals. *Histoplasma mississippiense* is more resistant to commonly used antifungals than *H. ohiense* (Goughenour et al. 2015) and (as most *Histoplasma* species) uses α‐(1,3)‐glucan in its cell wall to avoid immune recognition by the host, making the presence of this polysaccharide essential for virulence (Marion et al. 2006; Sepúlveda et al. 2014). *Histoplasma ohiense*, on the other hand, is the only *Histoplasma* species that lacks α‐(1,3)‐glucan in its cell wall, and is the only one to use Yps3p, a homolog of the *Blastomyces dermatitidis* adhesin Bad1p, as a yeast‐specific virulence factor (Bohse and Woods 2007). Thus, gene flow is not only possible between these species, but it could have a substantial impact on the evolution of pathogenesis in these species.

Our previous work confirmed that admixture does occur between *H. ohiense* and *H. mississippiense*, but it did not identify the precise alleles that had crossed the species boundary (Sepúlveda et al. 2017). In order to elucidate the role of introgression on the evolution of pathogenesis, the identity and frequency of introgressed alleles must be established. In this report, we identify the precise alleles that have crossed the species boundary in this species pair of pathogenic fungi. We find introgression in the two directions of the cross and present both strong evidence that the admixture event that led to the formation of these advanced intercrosses is recent and that introgressed functional elements are selected against in hybrids. Surprisingly, and rather ironically, the vast majority of introgressed material is present in only one individual per species: the genomic reference strain for each species. Thus, the reference strains for these species are actually advanced intercrosses between the two species.

## Methods

### GENOMIC DATA

We obtained 11 *H. ohiense* genomes and 10 *H. mississippiense* genomes (Sepúlveda et al. 2017). These 21 genomes were sequenced in parallel with similar target average coverage. Tables 1 and 2 show the list of genomes included in this study and their accession numbers.

**Table 1:** Characteristics of the introgressed genetic material from *H. ohiense* into *H. mississippiense* described for each isolate

Individual	Number of tracts	Cumulative length (bp)	Percentage of introgressed genome	Maximum haplotype length (bp)
505	1	842	0.0022	842
CI_19	0	0	0	0
CI_22	0	0	0	0
CI_24	0	0	0	0
CI_42	1	842	0.0022	842
CI_43	0	0	0	0
CI_7	1	803	0.0021	803
DOWNS	1	803	0.0021	803
UCLA_531	0	0	0	0
WU24	151	6,325,384	16.688	187,896

Each row represents a single *H. mississippiense* isolate. Introgression is rare in most isolates with the clear exception of WU24, an outlier isolate with more than 150 putative introgressions. *N* = 10 individuals were analyzed.

**Table 2:** Characteristics of the introgressed genetic material from *H. mississippiense* into *H. ohiense* described for each isolate

Individual	Number of tracts	Cumulative length	Percentage of introgressed genome	Maximum haplotype length
1986	3	33,891	0.0894	28,087
CI_10	2	1125	0.003	582
CI_17	3	13,138	0.0347	7625
CI_18	1	543	0.0014	543
CI_30	3	3380	0.0089	1762
CI_35	1	995	0.0026	995
CI_4	2	1125	0.003	582
CI_6	1	2178	0.0057	2178
CI_9	2	1542	0.0041	870
G217B	209	2,222,320	5.863	99,973
G222B	1	2615	0.0069	2615

Introgression is rare in most isolates with the clear exception of G217B, an outlier isolate with more than 200 putative introgressions. Each row represents a single *H. ohiense* isolate. *N* = 11 individuals were analyzed.

### READ MAPPING AND VARIANT CALLING

Reads were mapped to the *Histoplasma H88* genome (Sepúlveda et al. 2017) using bwa version 0.7.12 (Li and Durbin 2009, 2010). Bam files were merged using Samtools version 0.1.19 (Li et al. 2009). Indels were identified and reads were locally remapped in the merged bam files using the GATK version 3.2‐2 RealignerTargetCreator and IndelRealigner functions (McKenna et al. 2010; DePristo et al. 2011). We used GATK UnifiedGenotyper to call SNPs (parameter het = 0.01). We filtered the vcf file with the following paramters: QD = 2.0, FS_filter = 60.0, MQ_filter = 30.0, MQ_Rank_Sum_filter = –12.5, and Read_Pos_Rank_Sum_filter = –8.0. Finally, we used two filters based on coverage. We excluded sites covered by fewer than five reads, and sites with a coverage greater than the 99^th^ quantile of the genomic coverage distribution.

### INT‐HMM

**Selecting markers for the hidden Markov model**: Previous work based on the ABBA‐BABA D statistic suggested that introgression has occurred between *H. ohiens*e and *H. mississippiense* (Sepúlveda et al. 2017). However, although the ABBA‐BABA D statistic can evaluate whether introgression has occurred, it does not determine specific regions of the genome that have crossed the species boundary. To address this question, we used a hidden Markov model (HMM) originally designed to detect introgression in diploids (i.e., Int‐HMM; (Turissini and Matute 2017)) to identify introgressed regions in individuals from these two *Histoplasma* species. We adapted this model to detect introgression in haploids by assuming no heterozygous states. The HMM identifies introgressions between a pair of diverged population or species: a donor, and a recipient (i.e., that admixed individual) by inferring the ancestry of every SNP in the genome. Then it identifies a consecutive group of SNPs from the donor in the recipient background. Donor markers had to satisfy two criteria. First, they had to be monomorphic in the donor species. Second, they had to have an allele frequency difference between the two species greater of at least 30%. We also required that every individual in the donor species and at least one individual in the recipient species had a called genotype.

**Transition Probabilities**: Int‐HMM is a tool developed for the detection of introgression in diploids. We modified the model to detect introgression in haploids like *Histoplasma*. The likelihood of the HMM in the tool to move between hidden states is controlled by the transition probabilities between sites. The starting probabilities and separate transition between states depends on the physical distance between markers and are calculated for each marker in the genome. We assumed that the per‐site starting probabilities follow a Poisson distribution with a constant equal to the per site recombination rate, c, times the distance between the two adjacent sites. We used c = 10^−9^. The base transition probabilities for nonerror (*a*) were determined by:

$$\;a_{i}=\;c\left(x_{i}-x_{i-1}\right)e^{-c\left(x_{i}-x_{i-1}\right)}$$

as in (Turissini and Matute 2017). Since Int‐HMM was initially formulated for diploids, we modified the transition probabilities of the model to account for two possible states, donor (d) and recipient (r). The transition probability matrix represents the probability of transferring from the state denoted by the row to that of the column:

**Table:** 

		State (*i*)	
		d (Donor)	r (Recipient)
State (i‐1)	d (Donor)	$\left[\begin{array}{cc} 1-a_{i} & a_{i}\\ a_{i} & 1-a_{i} \end{array}\right]$	
	r (Recipient)		

Note that this transition probability matrix is similar to the one originally formulated for Int‐HMM (Turissini and Matute 2017) but does not include heterozygous states.

**Emission Probabilities**: The modified version of Int‐HMM only used biallelic sites as stated immediately above. All emission probabilities were calculated as specified originally in Int‐HMM.

### IDENTIFYING INTROGRESSION TRACTS

**Figure 1:**
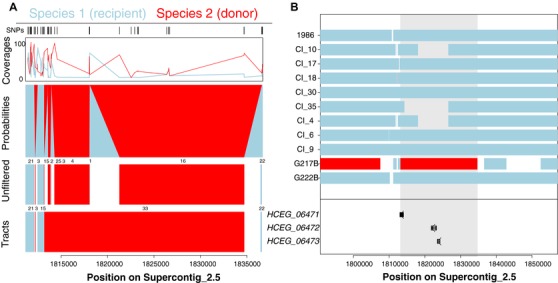
Int‐HMM uses a Hidden Markov model to detect introgressed haplotypes. (A) The algorithm uses ancestry information and coverage on each site to infer tracts of introgression. “Probabilities” are the output probabilities returned by Int‐HMM for the two states at each site (light blue: recipient, red: donor). Int‐HMM has two more possible states, donor error state, and recipient error state, which we did not observed (See also Files S1 and S2). “Unfiltered” represents the raw tracks obtained by grouping contiguous blocks of SNPs with the same most probable state. White space in this panel indicates no information for ancestry. “Tracts” are the inferred tracts after applying the filters listed in “Identifying introgression tracts” (i.e., connecting small haplotypes). (B) An example of an introgression. The inferred introgressed haplotype from H. mississippiense into H. ohiense contains three genes (HCEG_06471, HCEG_06472, and HCEG_06473) present only in the isolate G217B (*H. ohiense*).

Int‐HMM determined the most probable genotype for each marker in each individual. We defined tracts as contiguous markers with the same genotype, and applied filters to the raw results to smooth haplotypes and infer the minimal number of introgressed segments. We defined introgressed SNPs as those with an emission probability for the introgression state (*d*) (i.e., from the donor) higher than 50%. In cases where we identified a region of *d* with at least 10 introgressed SNP markers flanked on one side by a small tract (under 10 SNPs) from the recipient that in turn was flanked by a single larger tract that was completely *d*, the two introgressed regions were merged and consolidated into a single tract. We applied this filter four times, to allow identification of larger introgressed tracts that were broken up by small sections of the recipient species. These broken regions might be caused by gene conversion, double recombination events, or sequencing error (Fig. 1, Panel A shows an example).

### APPROXIMATE AGE OF THE ADMIXTURE EVENT

We used the average length of the introgression tracts to perform a first‐order estimate of the number of generations of recombination that occurred between hybridization and the present. We use the relationship between ancestry tract length and time since a single admixture event (Gravel 2012; Jin et al. 2014; Schumer et al. 2016) to solve for the number of generations of recombination given the average ancestry tract length of donor and the recombination rate:

T admix =1/LM∗pB

where T_admix_ is the number of generations since admixture, L_M_ is the average tract length in Morgans of the minor parent and p_B_ is the proportion of the genome derived from the major parent (probability of recombining). *Histoplasma* does reproduce sexually—although the frequency of sexual reproduction is unknown (Kwon‐Chung 1972, 1973, 1975; Kwon‐Chung and Weeks 1974). The rate of recombination has also not been measured in *Histoplasma*, so we used the rate of recombination measured in tetrad analyses in two other ascomycetes: *Schizosaccharomyces pombe* and *Saccharomyces cerevisiae*. In both species, the average recombination rate is 0.4 cM/kb (Rattray and Symington 1993; Turney et al. 2004). Other calculations suggest similar rates (in terms of the order of magnitude) for other ascomycete filamentous fungi (mean recombination rate in *Zymoseptoria* and *Neurospora* is both on the order of 0.1 cM/kb; Croll et al. 2015, Gladieux et al. 2015). It is worth noting that there is extensive variation in recombination rates in fungi. The minimum and maximum value known for the group (0.877 cM/kb and 0.008 cM/kb; Stapley et al. 2017) differ by three orders of magnitude. For example, *Filobasidiella neoformans*—Basidiomycetes—, and *Phycomyces blakesleeanus*—Zygomycetes— have mean recombination rates that are an order of magnitude lower than those observed in ascomycetes (Hsueh et al. 2006; Chaudhary et al. 2013). Due to this variation across Fungi, all calculations regarding the timing of introgression should be consider qualitative and not a point estimate.

Given the lack of overlap between introgressions in the two reciprocal directions of introgression, we treated each direction as an independent event. Since only one isolate per species shows evidence of introgressions (see Results), we did not use the mean haplotype length per species but rather used mean haplotype length for the two admixed individuals. Thus, our estimates are for the time to most recent admixture.

This estimate's accuracy relies on three assumptions that could be violated in our data: (*i*) that selection has not been acting on introgressed regions (i.e., they are neutral), (*ii*) that there is no bias in the detection of introgressions, and (*iii*) that there was only a single admixture event. The first assumption is almost certainly violated in this case, given the evidence for selection against introgressed regions (see below).

Given that the vast majority of introgression we detected was confined to a single individual in each species, we took advantage of the information provided by the percentage of genome introgressed in the admixed lines to calculate what generation of admixture they were if they were the result of a single hybridization and then repeated backcrossing. The amount of residual admixture after a single hybridization event and repeated backcrossing follows:

$$\mathrm{Residual}\;\mathrm{admixture}\;=\;{\left(\frac{1}{2}\right)}^{t}$$

where *t* is the number of generations of backcrossing.

Both these approaches should be viewed as a first approximation and be treated more as a qualitative contextualization of our data than as a precise date.

### ENRICHMENT BY SEQUENCE TYPE

If introgression was generally deleterious, it is reasonable to assume that selection would operate most efficiently against regions encoding functional elements (e.g. coding sequences, promoters, etc.). To test if a particular type of sequence was more or less prone to appear in introgressed regions, we partitioned the genome by sequence type into one of the following seven categories using *H. capsulatum var. duboisii* H88 genome annotations: CDS (coding sequence), 5’ ‐UTR, 3’ ‐UTR, intron, 2kb upstream inter (intergenic sequence 2kb upstream of a gene), 10kb inter (intergenic sequence within 10kb of a gene excluding 2kb upstream of a gene), and intergenic (intergenic sequence more than 10kb from a gene). Using *H. capsulatum var. duboisii* H88 annotations meant that errors in annotation would likely be present in both *H. ohiens*e or *H. mississippiense*, thus removing bias. Introgressions present in more than one individual but with different endpoints among isolates were broken into blocks, and these blocks were treated separately in the permutation test.

We calculated a summary statistic for each of the seven categories using the following definitions: “Introgressed percentage” is the percentage of introgressions overlapping a given sequence type that occurred in any of the four possible introgression directions (two different species pairs and two reciprocal directions), “Genomic percentage” is the percentage of the genome represented by a given sequence type, and “Enrichment” is the ratio between the percentage of introgressions of a given sequence type and the percentage of the genome encompassed by the same sequence type. To assess the significance of the “Enrichment” ratio, we calculated the likelihood that a randomly generated set of haplotypes, with the same size distribution of the introgressed haplotypes, showed the observed “Enrichment” value. We resampled the genome 10,000 times to create a distribution of resampled enrichments for each of the six sequence types. If introgressions are more likely than expected by random chance to occur within a certain type of sequence, then that sequence type should show an “Enrichment” value larger than 1. If introgressions are less likely than expected by random chance to occur within a type of sequence, then that sequence type will have an “Enrichment” value lower than 1. *P*‐values were calculated by permutations on the randomly generated distribution (i.e., 10,000 resamplings).

## Results

Previous analyses of genetic variation across populations using PCA and *Treemix* suggested that the vast majority of the genetic variation in *Histoplasma* is portioned across species, but also found evidence of shared genetic ancestry that was likely caused by introgression (Sepúlveda et al. 2017). We used a probabilistic framework to detect introgressions at the individual level using genome wide data for 11 *H. ohiense* and 10 *H. mississippiense individuals*. An example of this approach is shown in Figure 1. Our goal was to detect the precise genomic location of introgressions and their allele frequency in the two species.

### INTROGRESSED TRACTS ARE SEGREGATING AT LOW FREQUENCY IN EACH SPECIES

We inferred the average amount of introgressed material in each of the two reciprocal directions. As predicted by genome‐wide analyses (Sepulveda et al. 2017), we find evidence for introgressions that all occur at low frequency (see below). All the introgressions are shown in Files S1 and S2. Only one isolate from each species has a significant amount of introgression, and the amount of introgression in these isolates differs between species. The *H. mississippiense* isolate WU24—an isolate from Missouri, USA— carries 16.7% of genetic material from *H. ohiense* (Table 1). The *H. ohiense* isolate G217B carries 5.7% of genetic material from *H. mississippiense* (Table 2). All other isolates carry less than 0.1% of introgression in their genome, generally less than five tracts per isolate that usually differed between isolates. These results indicate that introgressions are segregating at low frequency in these species.

### LARGE INTROGRESSED TRACTS INDICATE RECENT ADMIXTURE

**Figure 2:**
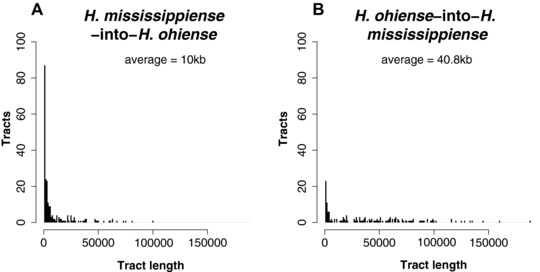
Distributions of the size of introgressed haplotypes in two species of *Histoplasma* identified using Int‐HMM. The Int‐HMM algorithm was run on each individual separately and the distribution of haplotype sizes was computed for each of the two directions of introgression. (A) Size distribution of introgressed haplotypes from *H. mississippiense* into *H. ohiense*. (B) Size distribution of introgressed haplotypes from *H. ohiense* into *H. mississippiense*. In the two reciprocal directions, there are large introgressions (over 50 kb) but the majority of the introgressions are small.

**Figure 3:**
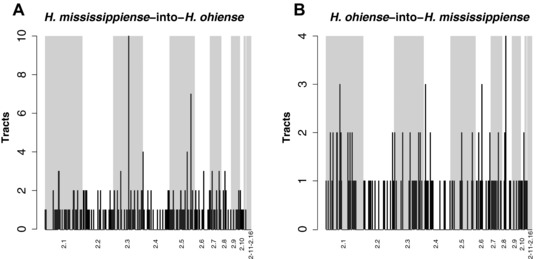
Genomic distributions of introgression tracts in two species of *Histoplasma*. We plotted the number of introgressions on each supercontig by counting the number of introgressed tracts in 500 kb windows. Each supercontig is represented by either a gray or white rectangle with the exception of supercontigs 2.11–2.16 that are small (collectively represent 1.4% of the genome) and were pooled together. Each panel shows a direction of introgression. Note the *y*‐axis differ. (A) Genome wide frequency of introgressions from *H. mississippiense* into *H. ohiense*. (B) Genome wide frequency of introgressions from *H. ohiense* into *H. mississippiense*.

We characterized the haplotype size frequency distribution and the location of introgressions between *H. ohiense* and *H. mississippiense*. We found that the majority of introgressed haplotypes are small, but that there is a long tail of larger introgressions in both directions (Fig. 2). The average size of a *H. mississippiense*‐to‐*H. ohiense* introgression was 10.0 kb (Fig. 2A). In the reciprocal direction, *H. ohiense*–to‐*H. mississippiense*, the average size of an introgression was 40.8 kb (Fig. 2B). This difference in haplotype size suggests that the admixture events that led to shared ancestry might have occurred at different times, or that the tolerance to introgressions differs in the genomes of *H. mississippiense* and *H. ohiense* (see below). We found that all the major supercontigs have introgressions (supercontigs 1–10) (Fig. 3), which is seemingly different from a previous report (Sepúlveda et al. 2017) in which the phylogenetic signal of shared ancestry was found exclusively in the smaller supercontigs (e.g., 11–14). However, these results are not inconsistent. Rather, they stem from the manner in which the D statistic infers the existence of introgression. The smaller contigs have a greater proportion of introgression—perhaps by chance, and therefore the phylogenetic signature of introgression is strongest. Thus, Int‐HMM can better detect introgression since it does not require a preponderance of introgressed regions in any contig.

Some of the introgressions were large haplotypes. In the single isolate of *H. mississippiense* with substantial admixture, WU24, we found eight haplotypes larger than 50 kb (File S1), and in the admixed isolate of *H. ohiense*, G217B, we found two (File S2). If we assume a similar recombination rate to that measured in other ascomycetous fungi (*Saccharomyces cerevisiae* and *Schizosaccharomyces pombe*, both with an average recombination rate of 0.4 cM/kb), each of these haplotypes represents ∼12 cM. We calculated an approximate time for the admixture event using the mean haplotype size of the introgressions found in each direction. We restricted these analyses to the two admixed individuals from the two species. We assumed that the rate of recombination in *Histoplasma* was similar to known recombination rates in other ascomycetous fungi: ∼0.4 cM/kb (Rattray and Symington 1993; Turney et al. 2004). These calculations suggest that introgression from *H. ohiense* into *H. mississippiense* occurred 1.64 ± 1.34 generations ago and introgression from *H. mississippiense* into *H. ohiense* occurred between 4.1 ± 2.2 generations ago. These age estimates are not statistically different from each other (Two‐Sample Fisher‐Pitman Permutation Test, *Z* = 3.811, *P* = 0.14). The percentage of introgressed genome indicates that WU24 and G217B are likely to be the result of four and six generations of backcrossing, respectively. Since the landscape of recombination remains unknown in *Histoplasma*, and there is substantial variation in recombination rates across species (Stapley et al. 2017), within species (Smukowski and Noor 2011, Ritz et al. 2017), and within genomes (e.g., Brion et al. 2017), these calculations should be considered a qualitative estimate of the recency of gene flow, and not a precise estimate of the time of admixture. Regardless of the precise age of admixture, the large size of the haplotypes indicates that hybridization occurred recently and rules out the possibility of retention of ancestral polymorphism (i.e., segregating variation that predates speciation).

### FUNCTIONAL GENOMIC ELEMENTS STRONGLY DEPLETED IN INTROGRESSED REGIONS IN BOTH SPECIES

We assessed whether any type of sequence was more likely to be found in introgressed regions than others. We divided the genome into seven categories: (1) 10 kb intergenic (intergenic sequence within 10 kb of a gene excluding 2 kb upstream of a gene), (2) 2 kb upstream intergenic (intergenic sequence 2 kb upstream of a gene), (3) 3’‐untranslated region (UTR), (4) 5’‐UTR, (5) introns, (6) coding sequences (CDS), and (7) intergenic (regions over 10 kb from a gene). (This analysis does not incorporate transposable elements or repetitive regions.) Functional elements are likely contained in the first six categories, whereas intergenic regions are less likely to be functional. For simplicity, we will refer to the first six categories as “functional elements,” although we stress that this is only a claim about the probability that an element is functional. In both reciprocal directions, we find that only intergenic sequences are overrepresented (∼3.5 and fourfold enrichment; Table 3). In contrast, all other regions, which harbor functional elements (CDS, 10 kb intergenic, 2 kb upstream intergenic, 3’‐UTR, 5’‐UTR, introns) are significantly underrepresented in introgressed regions (e.g., 20‐ and 40‐fold depletion of CDSs; Table 3). Note that the significant enrichments and depletions remain significant even using a Bonferroni correction for multiple testing. Taken together, these results strongly suggest negative selection against functional elements once introgressed.

**Table 3:** Introgressions are more likely to occur in noncoding regions of the genome

Direction	Sequence type	Length (kb)	Introgressed percentage	Genomic percentage	Enrichment	*P*‐value
***H. ohiense*‐into‐*H. mississippiense***	10kb inter	269.2	6.4	15.0	0.42	0.52
***H. ohiense*‐into‐*H. mississippiense***	2kb upstream inter	89.0	2.1	19.0	0.11	<0.0001
***H. ohiense*‐into‐*H. mississippiense***	3’ ‐UTR	1.4	0.0	3.0	0.01	<0.0001
***H. ohiense*‐into‐*H. mississippiense***	5’ ‐UTR	0.7	0.0	1.1	0.01	<0.0001
***H. ohiense*‐into‐*H. mississippiense***	CDS	26.1	0.6	33.2	0.02	<0.0001
***H. ohiense*‐into‐*H. mississippiense***	**intergenic**	**3832.5**	**90.8**	**21.1**	**4.31**	**<0.0001**
***H. ohiense*‐into‐*H. mississippiense***	intron	3.5	0.1	7.2	0.01	<0.0001
***H. mississippiense* ‐into‐*H. ohiense***	10kb inter	232.7	16.4	15.0	1.09	0.45
***H. mississippiense* ‐into‐*H. ohiense***	2kb upstream inter	112.0	7.9	19.0	0.42	0.24
***H. mississippiense* ‐into‐*H. ohiense***	3’ ‐UTR	1.0	0.1	3.0	0.02	<0.0001
***H. mississippiense* ‐into‐*H. ohiense***	5’ ‐UTR	0.3	0.0	1.1	0.02	<0.0001
***H. mississippiense* ‐into‐*H. ohiense***	CDS	25.6	1.8	33.2	0.05	<0.0001
***H. mississippiense* ‐into‐*H. ohiense***	**intergenic**	**1040.6**	**73.4**	**21.1**	**3.49**	**<0.0001**
***H. mississippiense* ‐into‐*H. ohiense***	intron	4.9	0.3	7.2	0.05	<0.0001

We studied what functional categories were more likely to be found in introgressions. We portioned the *Histoplasma* genome by sequence type in one of seven categories: 10 kb inter (intergenic sequence within 10 kb of a gene), 2 kb upstream inter (intergenic sequence 2 kb upstream of a gene), 3’‐UTR, 5’‐UTR, CDS (coding sequence), intergenic (intergenic sequence more than 10 kb from a gene), and intron. “Length (kb)” is the cumulative amount of introgressed DNA in each of the seven functional categories. “Introgressed percentage” is defined as the percentage of introgressions that contain each sequence type, “Genomic percentage” is the percentage of the genome in each of the seven categories, and “Enrichment” is defined as the ratio of introgressed percentage to genomic percentage. *P*‐values were calculated with permutation tests (see Methods for a full description). Overrepresented categories are bolded.

## Discussion

In this study, we investigate the timing of introgression and the identity of introgressed alleles in two species of the pathogenic fungus *Histoplasma*: *H. ohiense* and *H. mississippiense*. These are the only known species of *Histoplasma* to extensively share a geographic range and to co‐occur in the same locations. Previous studies have suggested that *Histoplasma* species could exchange genetic material, and that the most likely explanation for shared ancestry between *Histoplasma* species is introgression and not incomplete lineage sorting (Sepúlveda et al. 2017). Our study extends these results by examining the precise location of introgressions and their characteristics. We confirm that *H. ohiense* and *H. mississippiense* have exchanged genes, but that introgression is likely to be generally deleterious. Surprisingly, the vast majority of introgressed material is found only in the genomic reference strains for each species. Our results establish the frequency and fitness consequences of introgression in *H. ohiense* and *H. mississippiense* and suggest that caution is warranted in extending the phenotypes ascribed to the genomic reference strains of these species to the species more broadly.

We identified specific introgression tracts across species to examine general patterns of gene exchange. The majority of introgressions in either species are both large and at low frequency in our sample. Based on the size of the introgressions and the percentage of introgression in the genomes, we conclude that at least one admixture event between the *Histoplasma* species very likely occurred just a few sexual generations ago. Yet these introgressions are at low frequency, with the majority of them present in only a single individual. This suggests that many introgressions are deleterious or lethal in their new genomic context. Consistent with this, functional elements (CDSs, promoter regions, etc.) are underrepresented in the introgressed segments of the genomes of each species. Instead, most of the genetic material that has crossed species boundaries is located in intergenic regions. These regions are less likely to be functional and thus less likely to interact negatively with other elements in their new genomic context. A parsimonious explanation of these results is recent admixture followed by the rapid purging of functional introgressed alleles in each species.

In contrast to similar explorations of the introgression landscape in *Coccidioides* (Neafsey et al. 2010) and *Cryptococcus* (Desjardins et al. 2017), where introgression is found in small haplotypes, we found large shared haplotypes between *H. mississippiense* and *H. ohiense*. This size distribution seems to be more akin to the patterns found across species of *Neurospora* (Corcoran et al. 2016) and *Magnaporthe* (Gladieux et al. 2018), where large introgressions also have been observed. Defining the introgression landscape in fungi requires additional full genome sequences and clearly we cannot draw conclusions from such a small number of cases. A larger number of systematic studies of fungal introgression will reveal whether there are generalities (i.e., types of genes, or functional categories, more likely to cross species boundaries) or particularities that differentiate fungi from other taxa.

Perhaps the most important implication of our study is the urgent need to rethink the current isolates used as representatives in *Histoplasma*. The representative isolate for *H. mississippiense* in recent years has been WU24, while the representative isolate for *H. ohiense* is G217B. Both are the reference genome for their respective species. These isolates were used as the gold standards for the genus *Histoplasma* even before a taxonomical rearrangement of the genus was proposed. Yet both of these isolates carry large introgressions. WU24, the representative of *H. mississippiense*, carries as much genetic material (16.7%) from *H. ohiense* as an individual after two rounds of back‐crossing would. G217B, the representative of *H. ohiense* carries as much genetic material (5.7%) from *H. mississippiense* as a sixth‐generation backcross. Furthermore, these two isolates are the only isolates that carry substantial introgressions within those species as introgressions in other isolates cover less than 0.1% of their genomes. These results are important because hybrid individuals (even when they are advanced intercrosses) show aberrant or transgressive traits. The possibility of transgressive segregation of traits is intriguing given the well‐established divergent virulence strategies between these isolates. Our results indicate the need to verify the genetic ancestry of isolates before they become entrenched as the representative of a species.

## Conclusions

*Histoplasma* is a diverse genus that harbors species that differ in their virulence strategies. Even though their taxonomic status was elevated recently (Sepúlveda et al. 2017), the species of the genus have long been recognized to differ in key components of their pathogenicity, virulence, and drug resistance. In spite of stark differences between them, individuals from different species of the genus are able to interbreed and have done so recently. However, most alleles of functional elements in the genome have been expunged in the hybrids, strongly suggesting that they have been purged by selection. Similar patterns have been observed in sex chromosomes of organisms with heterogametic sexes where introgression is less frequent than in autosomes (Carneiro et al. 2010, 2014; Phifer‐Rixey et al. 2014; Maroja et al. 2015; Muirhead and Presgraves 2015; Turissini and Matute 2017) and in humans and Neanderthals where an underrepresentation of introgressed alleles involved in spermatogenesis has been interpreted as suggestive evidence of hybrid sterility (Sankararaman et al. 2016). An alternative explanation involves differences in mutational load in the two hybridizing lineages (Harris and Nielsen 2013; Juric et al. 2016; Steinrücken et al. 2018). A full assessment of the demographic history of the two species, and the identification of potential defects in hybrid progeny will be necessary before establishing which of these two options is more likely.

Our results have implications not only for fungi but pathogens in general. In cases where hybrid breakdown is not too severe, admixture is considered a possible source of introduced variation. This idea has been proposed as a possible source of concern in pathogens that might develop new mechanisms of pathogenesis or acquire drug resistance by the introduction of alleles from a different species (Stukenbrock 2013, 2016). The introgressed regions in hybrid *Histoplasma* are enriched for those alleles that would seem least likely to affect virulence. However, gene exchange does occur, and our results do not rule out alleles that do affect virulence. Furthermore, each of these isolates is in fact virulent, indicating that hybrids can at least retain virulence traits. Therefore, monitoring gene exchange in pathogens is an imperative need to avoid the emergence of new health threats.

Associate Editor: L. Bromham

## Supporting information


**FILE S1**. Inferred introgressions from *H. ohiense* into *H. mississippiense* inferred using Int‐HMM. Blue markers represent the *H. mississippiense* background; red blocks are introgressions from *H. ohiense*. Each of the 152 pages of the file shows an introgression with its relevant information (position, size, number of SNPS used to infer it —max_introgress_snps—). ‘*i* ind’ refers to the number of individuals (*i*) that show any evidence for shared ancestry in the shown genomic window. Sections of the genome with no evidence for introgression are not shown. The only isolate that shows evidence for extensive introgression is *H. mississippiense* WU24.

**FILE S2**. Inferred introgressions from *H. mississippiense* into *H. ohiense* inferred using Int‐HMM. Blue markers represent the *H. ohiense* background; red blocks are introgressions from *H. mississippiense*. Each of the 214 pages of the file shows an introgression with its relevant information (position, size, number of SNPS used to infer it —max_introgress_snps—). ‘*i* ind’ refers to the number of individuals (*i*) that show any evidence for shared ancestry in the shown genomic window. Sections of the genome with no evidence for introgression are not shown. The only isolate that shows evidence for extensive introgression is *H. ohiense* G217B.

## References

[evl359-bib-0001] Ajello, L. , and L. C. Runyon . 1953 Infection of mice with single spores of *Histoplasma capsulatum* . J. Bacteriol. 66:34–40. 13069463 10.1128/jb.66.1.34-40.1953 PMC357088

[evl359-bib-0002] Arnold, M. L. 2006 Evolution through genetic exchange. Oxford Univ. Press, Oxford, U.K.

[evl359-bib-0003] Balajee, S. A. , S. F. Hurst , L. S. Chang , M. Miles , E. Beeler , C. Hale , et al. 2013 Multilocus sequence typing of *Histoplasma capsulatum* in formalin‐fixed paraffin‐embedded tissues from cats living in non‐endemic regions reveals a new phylogenetic clade. Med. Mycol. 51:345–351. 23072593 10.3109/13693786.2012.733430

[evl359-bib-0004] Bohse, M. L. , and J. P. Woods . 2007 RNA interference‐mediated silencing of the YPS3 gene of *Histoplasma capsulatum* reveals virulence defects. Infect. Immun. 75:2811–2817. 17403872 10.1128/IAI.00304-07 PMC1932869

[evl359-bib-0005] Brion, C. , S. Legrand , J. Peter , C. Caradec , D. Pflieger , J. Hou , et al. 2017 Variation of the meiotic recombination landscape and properties over a broad evolutionary distance in yeasts. PLOS Genet. 13:e1006917. 28763437 10.1371/journal.pgen.1006917 PMC5554000

[evl359-bib-0006] Carneiro, M. , F. W. Albert , S. Afonso , R. J. Pereira , H. Burbano , R. Campos , et al. 2014 The Genomic architecture of population divergence between subspecies of the European rabbit. PLoS Genet. 10:e1003519. 25166595 10.1371/journal.pgen.1003519 PMC4148185

[evl359-bib-0007] Carneiro, M. , J. A. Blanco Aguiar , R. Villafuerte , N. Ferrand , and M. W. Nachman . 2010 Speciation in the European rabbit (*Oryctolagus cuniculus*): islands of differentiation on the x chromosome and autosomes. Evolution 64:3443–3460. 20666840 10.1111/j.1558-5646.2010.01092.x PMC3058625

[evl359-bib-0008] Carr, J. , and G. Shearer . 1998 Genome size, complexity, and ploidy of the pathogenic fungus *Histoplasma capsulatum* . J. Bacteriol. 180:6697–6703. 9852017 10.1128/jb.180.24.6697-6703.1998 PMC107776

[evl359-bib-0009] Chaudhary, S. , S. Polaino , V. P. Shakya , and A. Idnurm . 2013 A new genetic linkage map of the zygomycete fungus *Phycomyces blakesleeanus* . PLOS One 8:e58931. 23516579 10.1371/journal.pone.0058931 PMC3597544

[evl359-bib-0010] Corcoran, P. , J. L. Anderson , D. J. Jacobson , Y. Sun , P. Ni , M. Lascoux , and H. Johannesson . 2016 Introgression maintains the genetic integrity of the mating‐type determining chromosome of the fungus *Neurospora tetrasperma* . Genome Res. 26:486‐98. 26893460 10.1101/gr.197244.115 PMC4817772

[evl359-bib-0011] Coyne, J. A. , and H. A. Orr . 2004 Speciation. Sinauer Associates. Sunderland, MA, USA.

[evl359-bib-0012] Croll D. , M. H. Lendenmann , E. Stewart , and B. A. McDonald . 2015 The impact of recombination hotspots on genome evolution of a fungal plant pathogen. Genetics 201:1213–1228. 26392286 10.1534/genetics.115.180968 PMC4649646

[evl359-bib-0013] DePristo, M. A. , E. Banks , R. Poplin , K. V Garimella , J. R. Maguire , C. Hartl , et al. 2011 A framework for variation discovery and genotyping using next‐generation DNA sequencing data. Nat. Genet. 43:491–498. 21478889 10.1038/ng.806 PMC3083463

[evl359-bib-0014] Desjardins, C. A. , C. Giamberardino , S. M. Sykes , C. H. Yu , J. L. Tenor , Y. Chen , et al. 2017 Population genomics and the evolution of virulence in the fungal pathogen *Cryptococcus neoformans* . Genome Res. 27:1207–1219. 28611159 10.1101/gr.218727.116 PMC5495072

[evl359-bib-0015] Devier, B. , G. Aguileta , M. E. Hood , and T. Giraud . 2017 Using phylogenies of pheromone receptor genes in the *Microbotryum violaceum* species complex to investigate possible speciation by hybridization. Mycologia 102:689–696. 10.3852/09-192 20524600

[evl359-bib-0016] Freifeld, A. G. , P. C. Iwen , B. L. Lesiak , R. K. Gilroy , R. B. Stevens , and A. C. Kalil . 2005 Histoplasmosis in solid organ transplant recipients at a large Midwestern university transplant center. Transpl. Infect. Dis. 7:109–115. 16390398 10.1111/j.1467-8365.2005.00105.x

[evl359-bib-0017] Gladieux, P. , B. A. Wilson , F. Perraudeau , L.A. Montoya , D. Kowbel , C. Hann‐Soden , et al. 2015 Genomic sequencing reveals historical, demographic and selective factors associated with the diversification of the fire‐associated fungus *Neurospora discreta* . Mol. Ecol. 4:5657–5675. 10.1111/mec.13417 26453896

[evl359-bib-0018] Gladieux P , B. Condon , S. Ravel , D. Soanes , J. L. Maciel , A. Nhani , et al. 2018 Gene flow between divergent cereal‐and grass‐specific lineages of the rice blast fungus *Magnaporthe oryzae* . mBio. 9:e01219‐17. 29487238 10.1128/mBio.01219-17 PMC5829825

[evl359-bib-0019] Goughenour, K. D. , J.‐M. Balada‐Llasat , and C. A. Rappleye . 2015 Quantitative microplate‐based growth assay for determination of antifungal susceptibility of *Histoplasma capsulatum* yeasts. J. Clin. Microbiol. 53:3286–3295. 26246483 10.1128/JCM.00795-15 PMC4572531

[evl359-bib-0020] Gravel, S. 2012 Population genetics models of local ancestry. Genetics 191:607–619. 22491189 10.1534/genetics.112.139808 PMC3374321

[evl359-bib-0021] Graybill, J. R. 1988 Histoplasmosis and AIDS. J. Infect. Dis. 158:623–626. 3045214 10.1093/infdis/158.3.623

[evl359-bib-0022] Hage, C. A. , M. M. Azar , N. Bahr , J. Loyd , and L. J. Wheat . 2015 Histoplasmosis: up‐to‐date evidence‐based approach to diagnosis and management. Semin. Respir. Crit. Care Med. 36:729–745. 26398539 10.1055/s-0035-1562899

[evl359-bib-0023] Harris, K. , and R. Nielsen . 2013 Inferring demographic history from a spectrum of shared haplotype lengths. PLoS Genet. 9:e1003521. 23754952 10.1371/journal.pgen.1003521 PMC3675002

[evl359-bib-0024] Heil, C. S. , J. N. Burton , I. Liachko , A. Friedrich , N. A. Hanson , C. L. Morris , et al. 2017 Identification of a novel interspecific hybrid yeast from a metagenomic spontaneously inoculated beer sample using Hi‐C. Yeast 35:71–84. 28892574 10.1002/yea.3280 PMC5771821

[evl359-bib-0025] Hsueh, Y. P. , A. Idnurm , and J. Heitman . 2006 Recombination hotspots flank the *Cryptococcus* mating‐type locus: implications for the evolution of a fungal sex chromosome. PLOS Genet. 3:e184. 10.1371/journal.pgen.0020184 PMC1630710 17083277

[evl359-bib-0026] Jin, W. , R. Li , Y. Zhou , and S. Xu . 2014 Distribution of ancestral chromosomal segments in admixed genomes and its implications for inferring population history and admixture mapping. Eur. J. Hum. Genet. 22:930–937. 24253859 10.1038/ejhg.2013.265 PMC4060116

[evl359-bib-0027] Juric, I. , S. Aeschbacher , and G. Coop . 2016 The strength of selection against Neanderthal introgression. PLoS Genet. 12:e1006340. 27824859 10.1371/journal.pgen.1006340 PMC5100956

[evl359-bib-0028] Kasuga, T. , J. W. Taylor , and T. J. White . 1999 Phylogenetic relationships of varieties and geographical groups of the human pathogenic fungus *Histoplasma capsulatum* Darling. J. Clin. Microbiol. 37:653–663. 9986828 10.1128/jcm.37.3.653-663.1999 PMC84508

[evl359-bib-0029] Kasuga, T. , T. J. White , G. Koenig , J. Mcewen , A. Restrepo , E. Castañeda , et al. 2003 Phylogeography of the fungal pathogen *Histoplasma capsulatum. Mol* . Ecol. 12:3383–3401. 10.1046/j.1365-294x.2003.01995.x 14629354

[evl359-bib-0030] Knox, K. S. 2014 Perspective on coccidioidomycosis and histoplasmosis. Am. J. Respir. Crit. Care Med. 189:752–753. 24628318 10.1164/rccm.201311-2024LE

[evl359-bib-0031] Kwon‐Chung, K. J. 1972 *Emmonsiella capsulata*: perfect state of *Histoplasma capsulatum* . Science 177:368–369. 5035491 10.1126/science.177.4046.368

[evl359-bib-0032] Kwon‐Chung, K. J. 1975 Perfect state (*Emmonsiella capsulata*) of the fungus causing large‐form African histoplasmosis. Mycologia 67:980‐990. 1196334

[evl359-bib-0033] Kwon‐Chung, K. J. 1973 Studies on *Emmonsiella capsulata* I. Heterothallism and development of the ascocarp. Mycologia 65:109–121. 4686214

[evl359-bib-0034] Kwon‐Chung, K. J. , and R. J. Weeks . 1974 Studies on *Emmonsiella capsulata* (*Histoplasma capsulatum*): II. Distribution of the two mating types in 13 endemic states of the United States. Am. J. Epidemiol. 99:44–49. 4811175 10.1093/oxfordjournals.aje.a121583

[evl359-bib-0035] Li, H. , and R. Durbin . 2009 Fast and accurate short read alignment with Burrows/Wheeler transform. Bioinformatics 25:1754–1760. 19451168 10.1093/bioinformatics/btp324 PMC2705234

[evl359-bib-0036] Li, H. , B. Handsaker , A. Wysoker , T. Fennell , G. Marth , G. Abecasis and R. Durbin . 2009 The sequence alignment/map format and SAMtools. Bioinformatics 25:2078–2079. 19505943 10.1093/bioinformatics/btp352 PMC2723002

[evl359-bib-0037] Marion, C. L. , C. A. Rappleye , J. T. Engle , and W. E. Goldman . 2006 An α‐(1,4)‐amylase is essential for α‐(1,3)‐glucan production and virulence in *Histoplasma capsulatum* . Mol. Microbiol. 62:970–983. 17038119 10.1111/j.1365-2958.2006.05436.x

[evl359-bib-0038] Maroja, L. S. , E. L. Larson , S. M. Bogdanowicz , and R. G. Harrison . 2015 Genes with restricted introgression in a field cricket (*Gryllus firmus/Gryllus pennsylvanicus*) hybrid zone are concentrated on the *X* chromosome and a single autosome. G3 5:2219–27. 26311650 10.1534/g3.115.021246 PMC4632042

[evl359-bib-0039] McKenna, A. , M. Hanna , E. Banks , A. Sivachenko , K. Cibulskis , A. Kernytsky , et al. 2010 The Genome Analysis Toolkit: a MapReduce framework for analyzing next‐generation DNA sequencing data. Genome Res. 20:1297–1303. 20644199 10.1101/gr.107524.110 PMC2928508

[evl359-bib-0040] Muirhead, C. A. , and D. C. Presgraves . 2015 Hybrid incompatibilities, local adaptation, and the genomic distribution of natural introgression between species. Am. Nat. 187:249–261. 26807751 10.1086/684583

[evl359-bib-0041] Neafsey, D. E. , B. M. Barker , T. J. Sharpton , J. E. Stajich , D. J. Park , E. Whiston , et al. 2010 Population genomic sequencing of *Coccidioides* fungi reveals recent hybridization and transposon control. Genome Res. 20:938–946. 20516208 10.1101/gr.103911.109 PMC2892095

[evl359-bib-0042] Phifer‐Rixey, M. , M. Bomhoff , and M. W. Nachman . 2014 Genome‐wide patterns of differentiation among house mouse subspecies. Genetics 198:283–297. 24996909 10.1534/genetics.114.166827 PMC4174939

[evl359-bib-0043] Rappleye, C. A. , J. T. Engle , and W. E. Goldman . 2004 RNA interference in *Histoplasma capsulatum* demonstrates a role for α‐(1,3)‐glucan in virulence. Mol. Microbiol. 53:153–165. 15225311 10.1111/j.1365-2958.2004.04131.x

[evl359-bib-0044] Rattray, A. J. , and L. S. Symington . 1993 Stimulation of meiotic recombination in yeast by an ARS element. Genetics 134:175–188. 8514126 10.1093/genetics/134.1.175 PMC1205420

[evl359-bib-0045] Ritz, K. R. , M.A. Noor , and N. D. Singh . 2017 Variation in recombination rate: adaptive or not? Trends Genet. 33:364–374. 28359582 10.1016/j.tig.2017.03.003

[evl359-bib-0046] Sankararaman, S. , S. Mallick , N. Patterson , and D. Reich . 2016 The combined landscape of Denisovan and Neanderthal ancestry in present‐day humans. Curr. Biol. 26:1241–1247. 27032491 10.1016/j.cub.2016.03.037 PMC4864120

[evl359-bib-0047] Schardl, C. L. , and K. D. Craven . 2003 Interspecific hybridization in plant‐associated fungi and oomycetes: a review. Mol. Ecol. 12:2861–2873. 14629368 10.1046/j.1365-294x.2003.01965.x

[evl359-bib-0048] Schumer, M. , R. Cui , D. L. Powell , G. G. Rosenthal , and P. Andolfatto . 2016 Ancient hybridization and genomic stabilization in a swordtail fish. Mol. Ecol. 25:2661–2679. 26937625 10.1111/mec.13602

[evl359-bib-0049] Sebghati, T. S. , J. T. Engle , and W. E. Goldman . 2000 Intracellular parasitism by *Histoplasma capsulatum*: fungal virulence and calcium dependence. Science 290:1368–1372. 11082066 10.1126/science.290.5495.1368

[evl359-bib-0050] Sepúlveda, V. E. , R. Márquez , D. A. Turissini , W. E. Goldman , and D. R. Matute . 2017 Genome sequences reveal cryptic speciation in the human pathogen *Histoplasma capsulatum* . mBio 8:e01339–17. 29208741 10.1128/mBio.01339-17 PMC5717386

[evl359-bib-0051] Sepúlveda, V. E. , C. L. Williams , and W. E. Goldman . 2014 Comparison of phylogenetically distinct Histoplasma strains reveals evolutionarily divergent virulence strategies. mBio 5:e01376–14. 24987093 10.1128/mBio.01376-14 PMC4161242

[evl359-bib-0052] Smukowski, C.S. , and M. A. Noor . 2011 Recombination rate variation in closely related species. Heredity 107:496–508. 21673743 10.1038/hdy.2011.44 PMC3242630

[evl359-bib-0053] Stapley, J. , P. G. Feulner , S. E. Johnston , A. W. Santure , and C. M. Smadja . 2017 Variation in recombination frequency and distribution across eukaryotes: patterns and processes. Phil. Trans. R Soc. B. 372:20160455. 29109219 10.1098/rstb.2016.0455 PMC5698618

[evl359-bib-0054] Steinrücken, M. , J. P. Spence , J. A. Kamm , E. Wieczorek , and Y. S. Song . 2018 Model‐based detection and analysis of introgressed Neanderthal ancestry in modern humans. Mol. Ecol. 10.1111/mec.14565. PMC6165692 29603507

[evl359-bib-0055] Stukenbrock, E. H. 2013 Evolution, selection and isolation: a genomic view of speciation in fungal plant pathogens. New Phytol. 199:895–907. 23782262 10.1111/nph.12374

[evl359-bib-0056] Stukenbrock, E. H. 2016 The role of hybridization in the evolution and emergence of new fungal plant pathogens. Phytopathology 106:104–112. 26824768 10.1094/PHYTO-08-15-0184-RVW

[evl359-bib-0057] Turissini, D. A. , and D. R. Matute . 2017 Fine scale mapping of genomic introgressions within the *Drosophila yakuba* clade. PLoS Genet. 13:e1006971. 28873409 10.1371/journal.pgen.1006971 PMC5600410

[evl359-bib-0058] Turney, D. , T. de Los Santos , and N. M. Hollingsworth . 2004 Does chromosome size affect map distance and genetic interference in budding yeast? Genetics 168:2421–2424. 15611199 10.1534/genetics.104.033555 PMC1448730

[evl359-bib-0059] Wheat, L. J. 2003 Current diagnosis of histoplasmosis. Trends Microbiol. 11:488–494. 14557032 10.1016/j.tim.2003.08.007

[evl359-bib-0060] Wheat, L. J. , M. M. Azar , N. C. Bahr , A. Spec , R. F. Relich , and C. Hage . 2016 Histoplasmosis. Infect. Dis. Clin. North Am. 30:207–227. 26897068 10.1016/j.idc.2015.10.009

[evl359-bib-0061] Williams, D. M. , J. R. Graybill , and D. J. Drutz . 1978 *Histoplasma capsulatum* infection in nude mice. Infect. Immun. 21:973–977. 309438 10.1128/iai.21.3.973-977.1978 PMC422092

[evl359-bib-0062] Woods, J. P. , E. L. Heinecke , and W. E. Goldman . 1998 Electrotransformation and expression of bacterial genes encoding hygromycin phosphotransferase and beta‐galactosidase in the pathogenic fungus *Histoplasma capsulatum* . Infect. Immun. 66:1697–1707. 9529100 10.1128/iai.66.4.1697-1707.1998 PMC108107

