# Supplementary material for: Recent admixture between species of the fungal pathogen Histoplasma
Source: Evol Lett. 2018 Jun 22;2(3):210–20. doi: 10.1002/evl3.59 (PMC6121842; doi:10.1002/evl3.59)
Supplement: Supplementary file 2 — FILE S2. Inferred introgressions from H. mississippiense into H. ohiense inferred using Int‐HMM. Blue markers represent the H. ohiense background; red blocks are introgressions from H. mississippiense. Each of the 214 pages of the file shows an introgression with its relevant information (position, size, number of SNPS used to infer it —max_introgress_snps—). ‘i ind’ refers to the number of individuals (i) that show any evidence for shared ancestry in the shown genomic window. Sections of the genome with no evidence for introgression are not shown. The only isolate that shows evidence for extensive introgression is H. ohiense G217B. [file EVL3-2-210-s002.pdf]

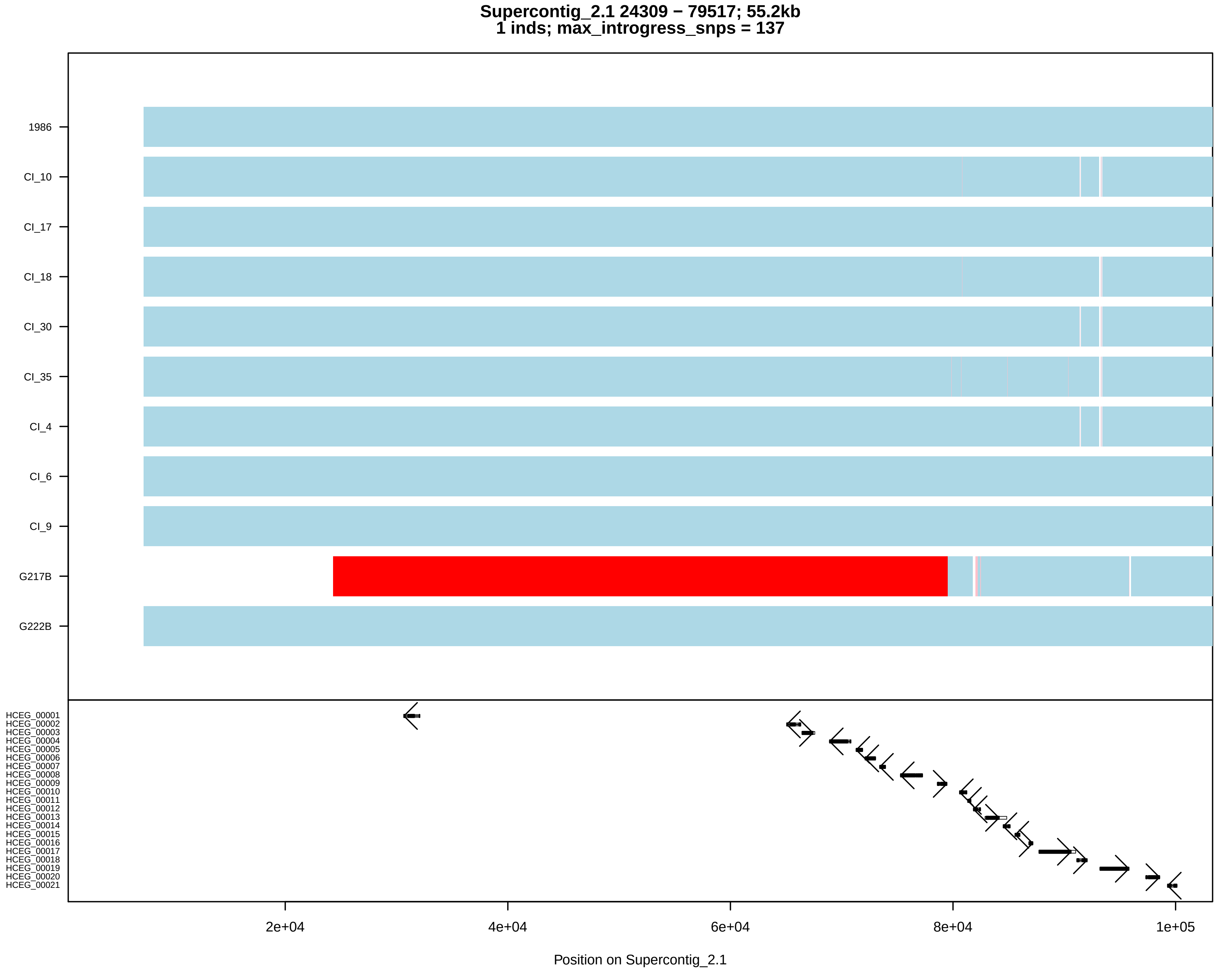
Supercontig_2.1 24309 − 79517; 55.2kb
1 inds; max_introgress_snps = 137
1986
CI_10
CI_17
CI_18
CI_30
CI_35
CI_4
CI_6
CI_9
G217B
G222B
HCEG_00001
HCEG_00002
HCEG_00003
HCEG_00004
HCEG_00005
HCEG_00006
HCEG_00007
HCEG_00008
HCEG_00009
HCEG_00010
HCEG_00011
HCEG_00012
HCEG_00013
HCEG_00014
HCEG_00015
HCEG_00016
HCEG_00017
HCEG_00018
HCEG_00019
HCEG_00020
HCEG_00021
2e+04
4e+04
6e+04
8e+04
1e+05
Position on Supercontig_2.1

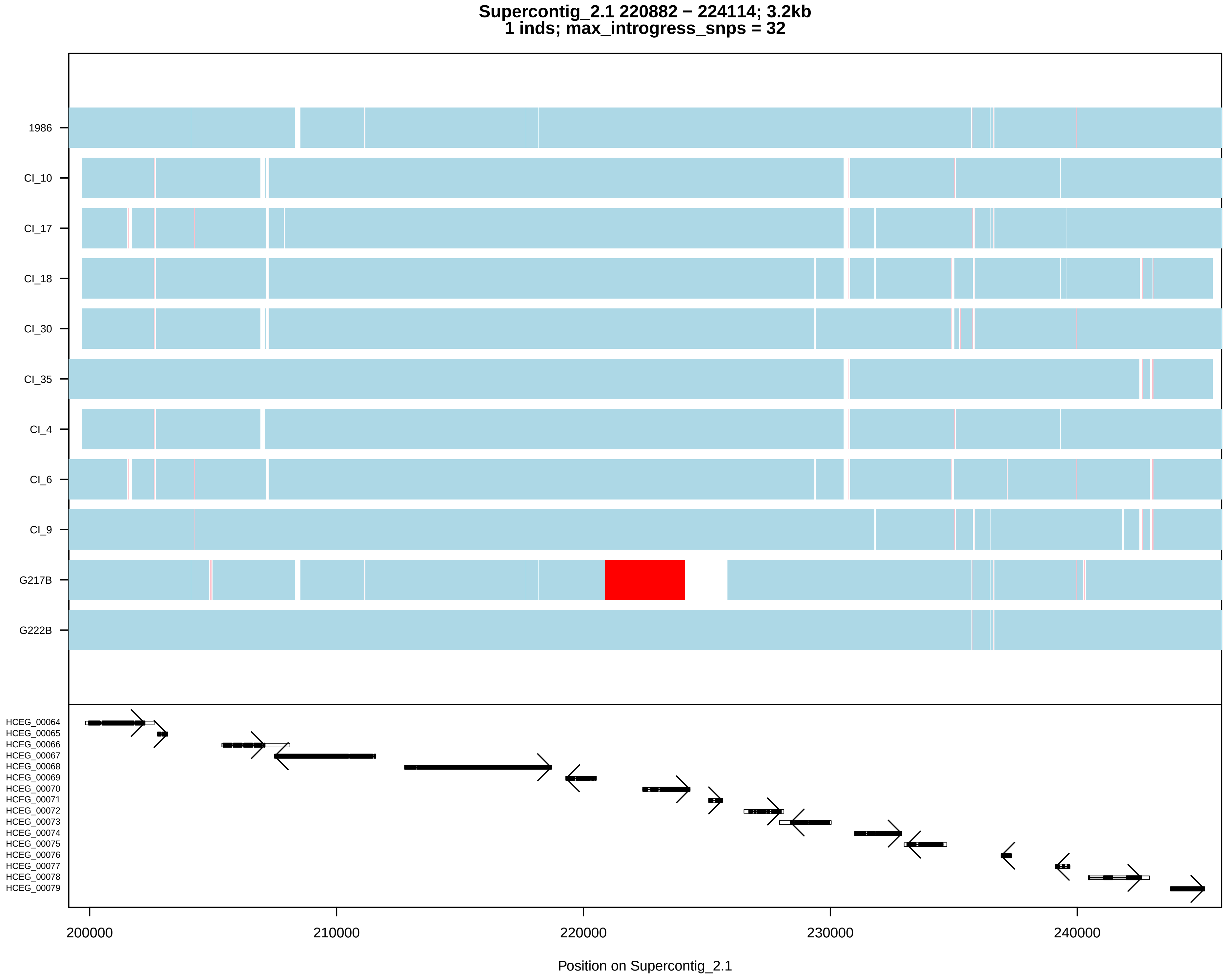
Supercontig_2.1 220882 − 224114; 3.2kb
1 inds; max_introgress_snps = 32
1986
CI_10
CI_17
CI_18
CI_30
CI_35
CI_4
CI_6
CI_9
G217B
G222B
HCEG_00064
HCEG_00065
HCEG_00066
HCEG_00067
HCEG_00068
HCEG_00069
HCEG_00070
HCEG_00071
HCEG_00072
HCEG_00073
HCEG_00074
HCEG_00075
HCEG_00076
HCEG_00077
HCEG_00078
HCEG_00079
200000
210000
220000
230000
240000
Position on Supercontig_2.1

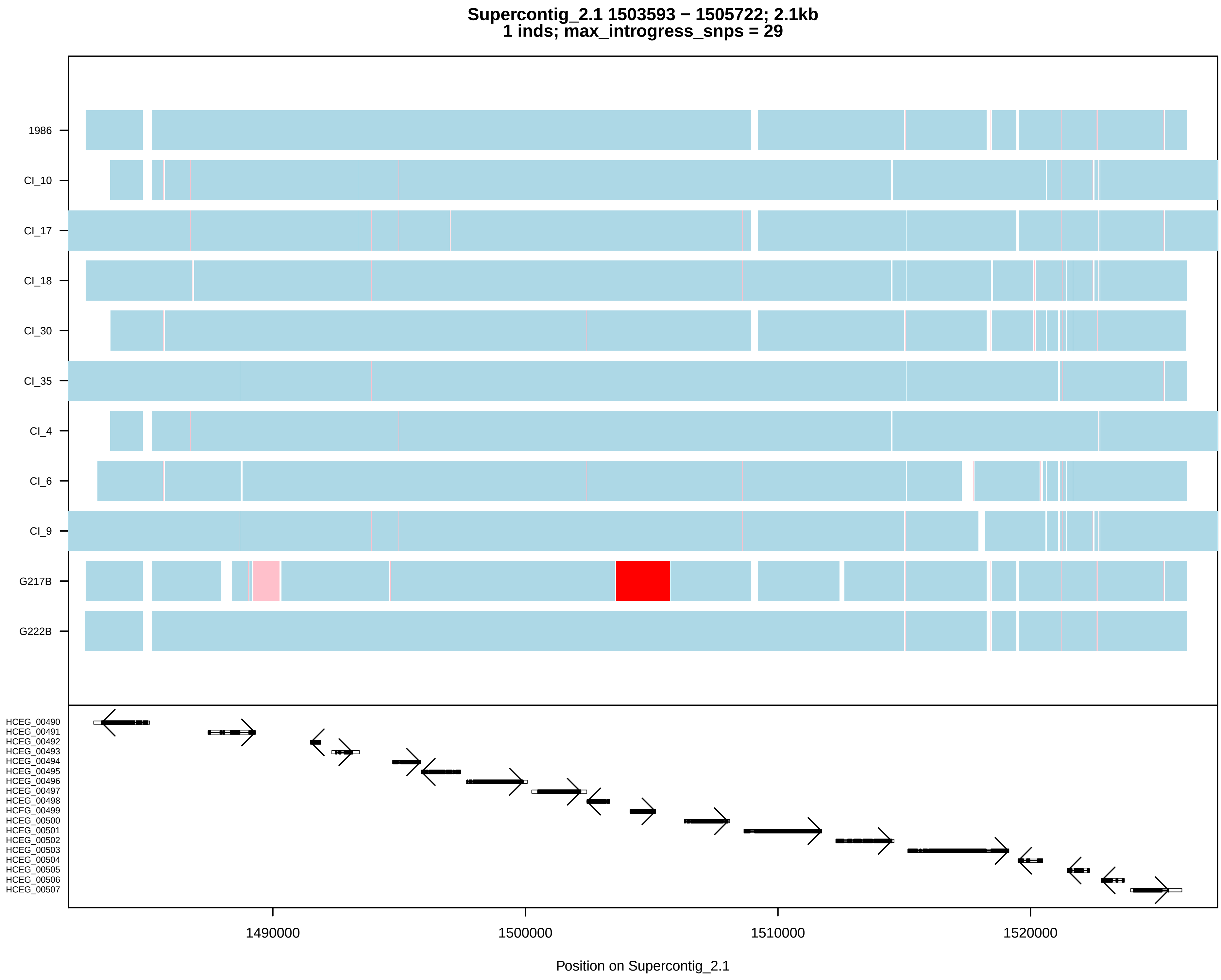
Supercontig_2.1 1503593 − 1505722; 2.1kb
1 inds; max_introgress_snps = 29
1986
CI_10
CI_17
CI_18
CI_30
CI_35
CI_4
CI_6
CI_9
G217B
G222B
HCEG_00490
HCEG_00491
HCEG_00492
HCEG_00493
HCEG_00494
HCEG_00495
HCEG_00496
HCEG_00497
HCEG_00498
HCEG_00499
HCEG_00500
HCEG_00501
HCEG_00502
HCEG_00503
HCEG_00504
HCEG_00505
HCEG_00506
HCEG_00507
1490000
1500000
1510000
1520000
Position on Supercontig_2.1

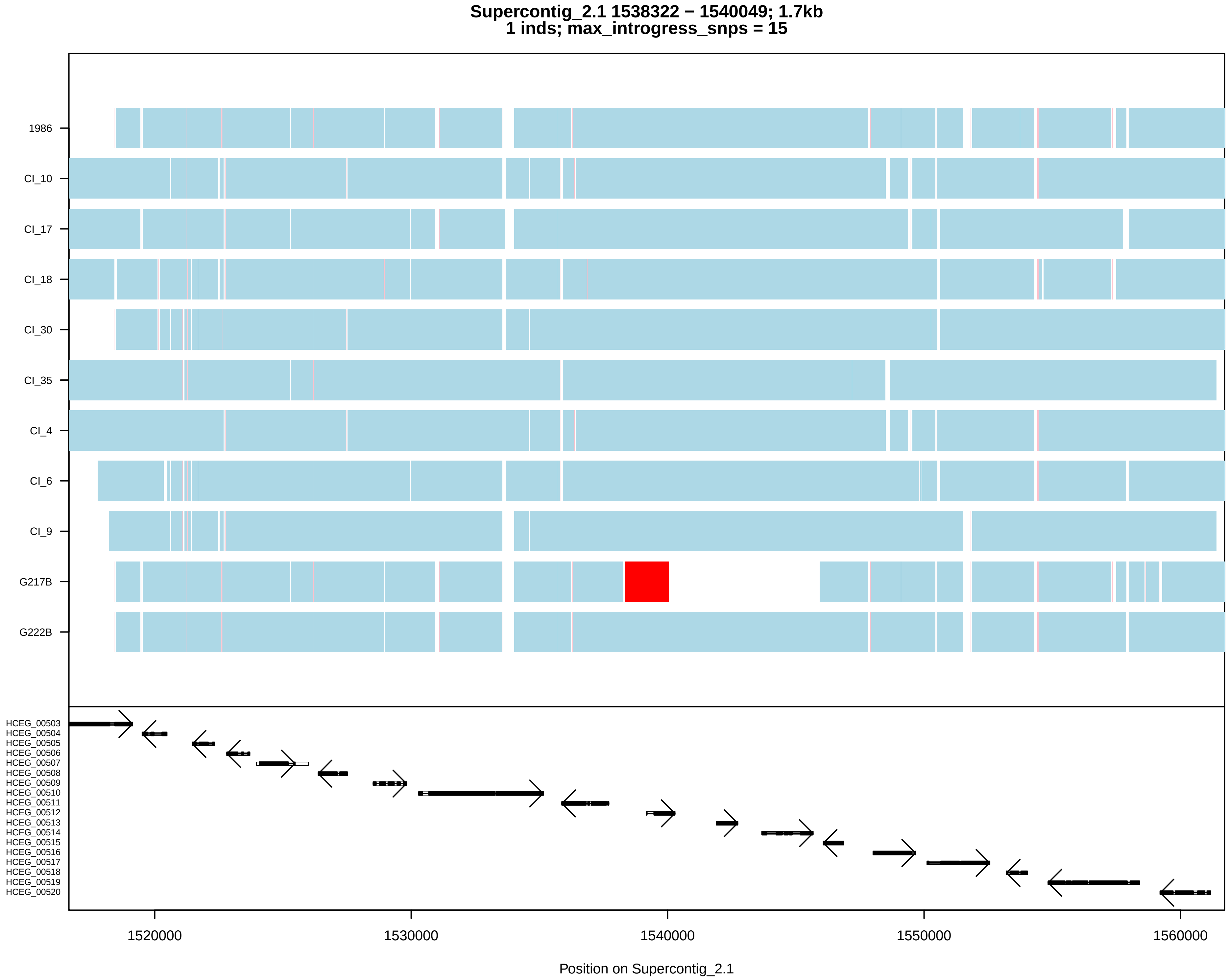
Supercontig_2.1 1538322 − 1540049; 1.7kb
1 inds; max_introgress_snps = 15
1986
CI_10
CI_17
CI_18
CI_30
CI_35
CI_4
CI_6
CI_9
G217B
G222B
HCEG_00503
HCEG_00504
HCEG_00505
HCEG_00506
HCEG_00507
HCEG_00508
HCEG_00509
HCEG_00510
HCEG_00511
HCEG_00512
HCEG_00513
HCEG_00514
HCEG_00515
HCEG_00516
HCEG_00517
HCEG_00518
HCEG_00519
HCEG_00520
1520000
1530000
1540000
1550000
1560000
Position on Supercontig_2.1

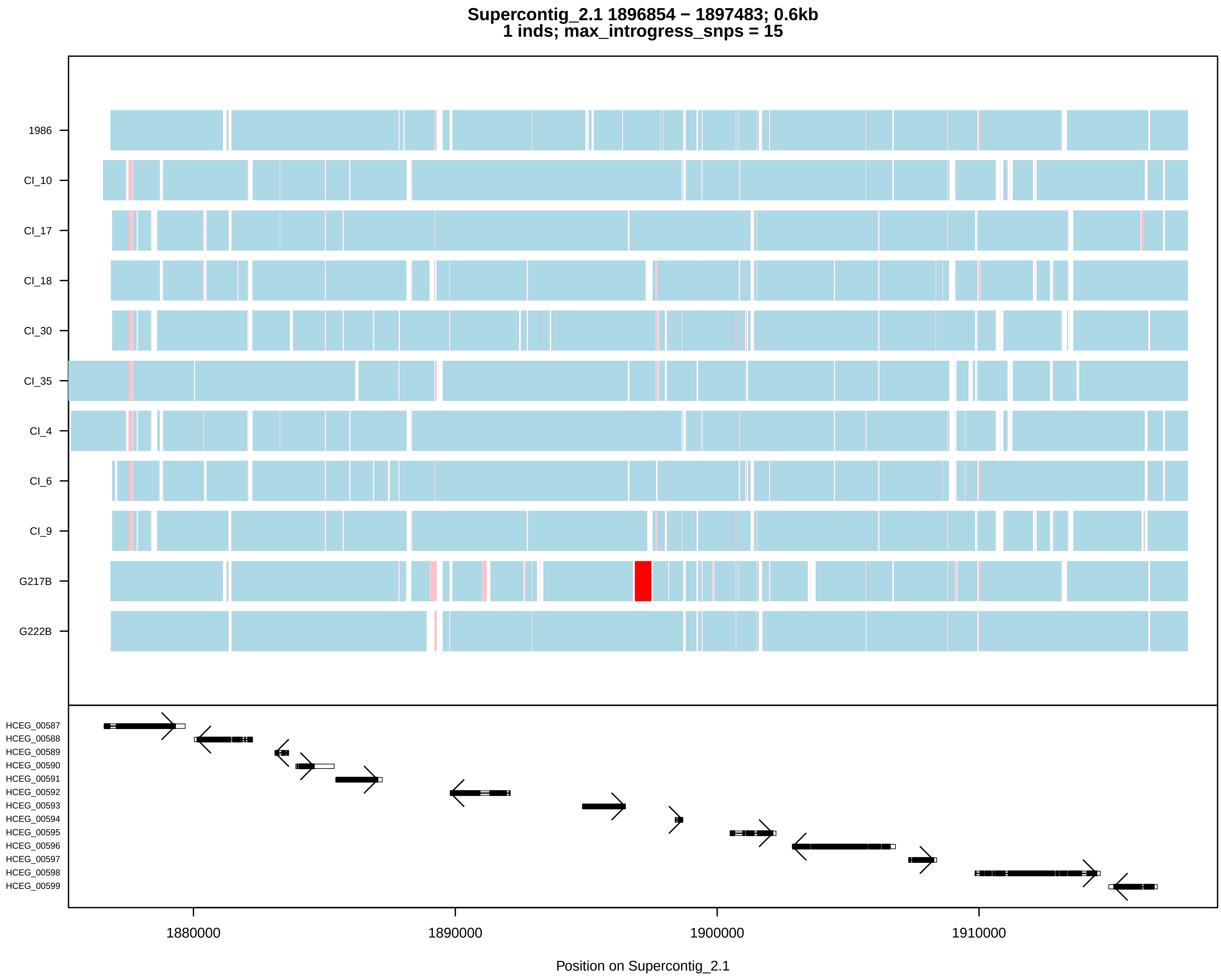

Supercontig_2.1 1896854 − 1897483; 0.6kb
1 inds; max_introgress_snps = 15
1986
CI_10
CI_17
CI_18
CI_30
CI_35
CI_4
CI_6
CI_9
G217B
G222B
HCEG_00587
HCEG_00588
HCEG_00589
HCEG_00590
HCEG_00591
HCEG_00592
HCEG_00593
HCEG_00594
HCEG_00595
HCEG_00596
HCEG_00597
HCEG_00598
HCEG_00599
1880000
1890000
1900000
1910000
Position on Supercontig_2.1

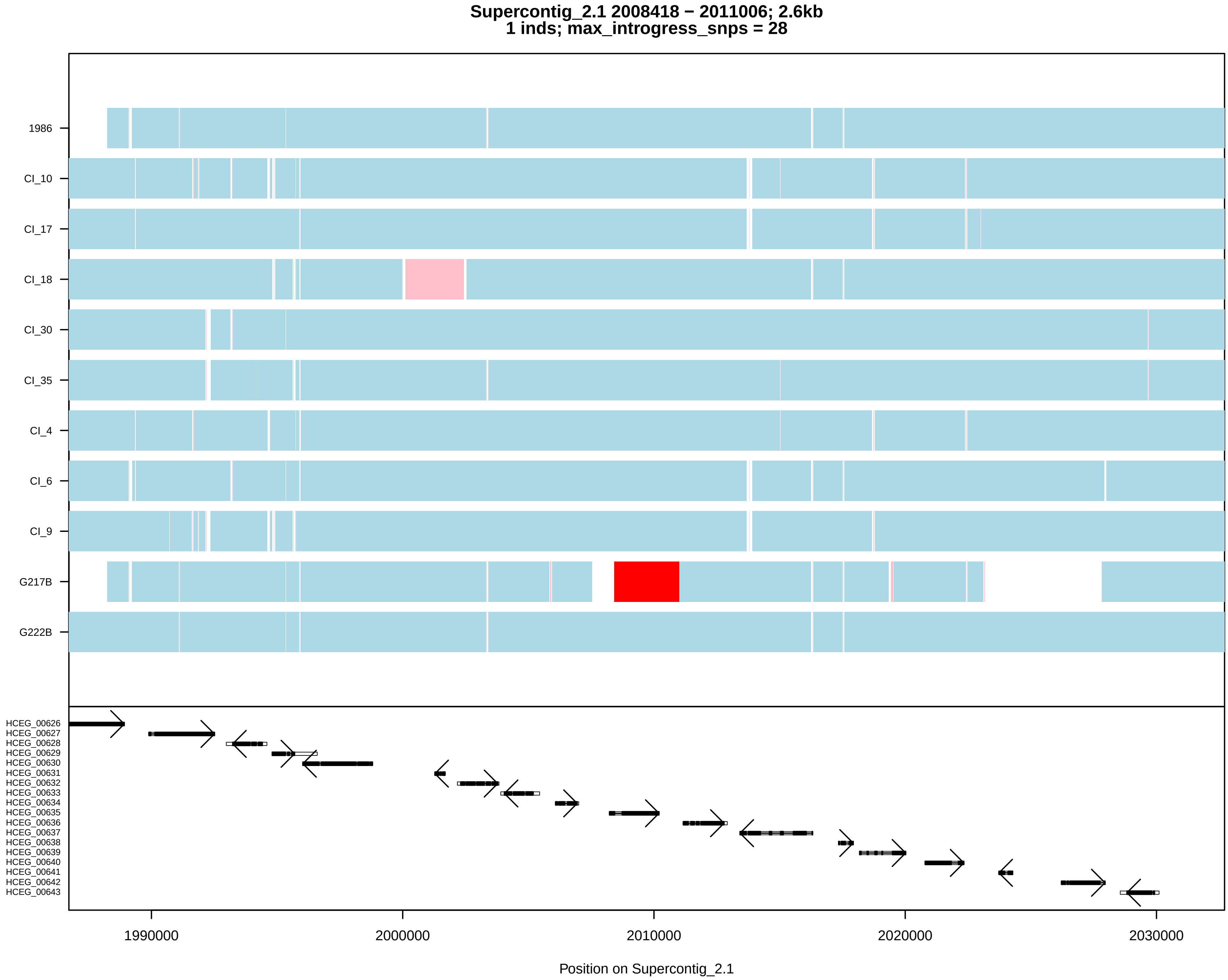
Supercontig_2.1 2008418 − 2011006; 2.6kb
1 inds; max_introgress_snps = 28
1986
CI_10
CI_17
CI_18
CI_30
CI_35
CI_4
CI_6
CI_9
G217B
G222B
HCEG_00626
HCEG_00627
HCEG_00628
HCEG_00629
HCEG_00630
HCEG_00631
HCEG_00632
HCEG_00633
HCEG_00634
HCEG_00635
HCEG_00636
HCEG_00637
HCEG_00638
HCEG_00639
HCEG_00640
HCEG_00641
HCEG_00642
HCEG_00643
1990000
2000000
2010000
2020000
2030000
Position on Supercontig_2.1

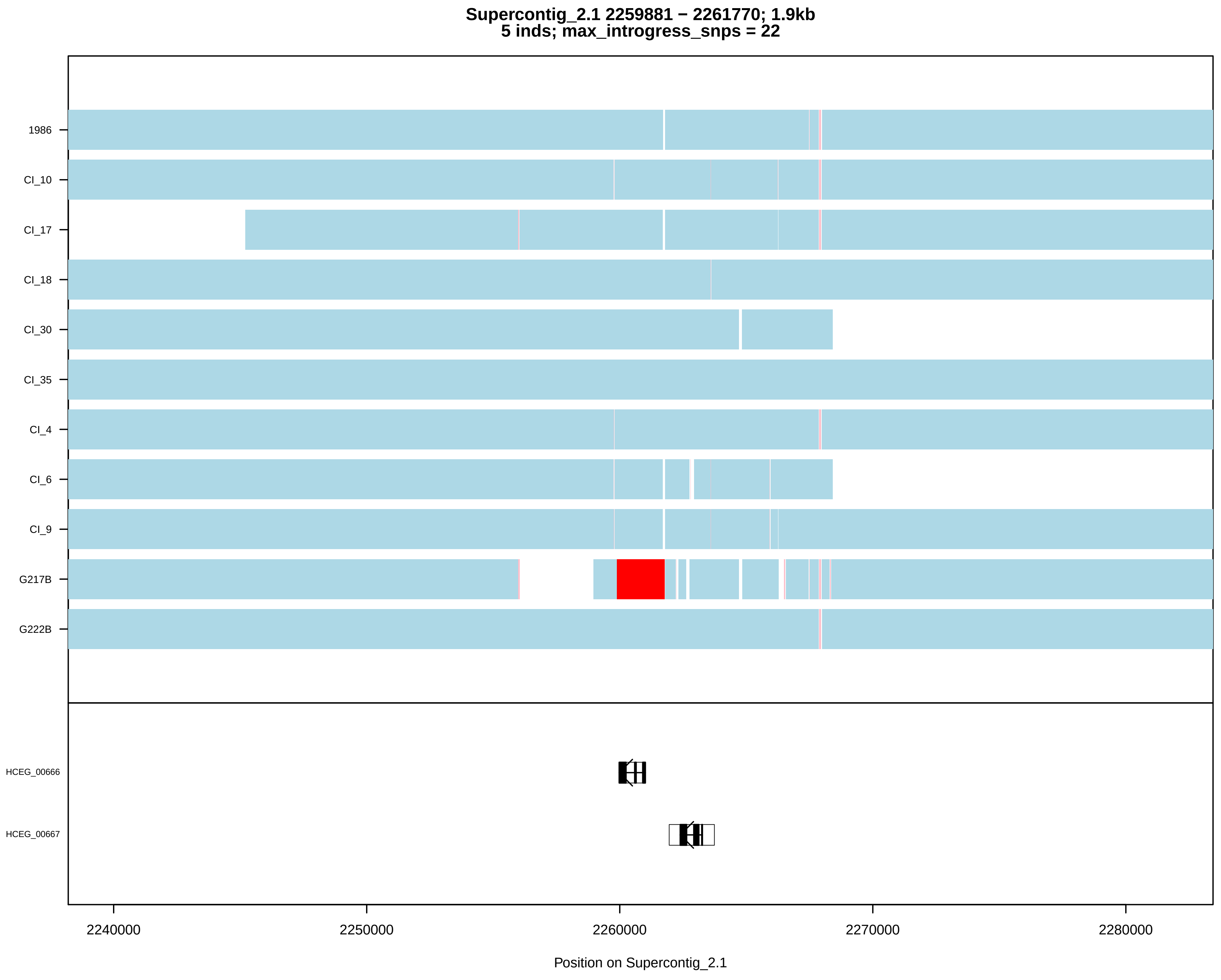

Supercontig_2.1 2259881 − 2261770; 1.9kb
5 inds; max_introgress_snps = 22
1986
CI_10
CI_17
CI_18
CI_30
CI_35
CI_4
CI_6
CI_9
G217B
G222B
HCEG_00666
HCEG_00667
2240000
2250000
2260000
2270000
2280000
Position on Supercontig_2.1

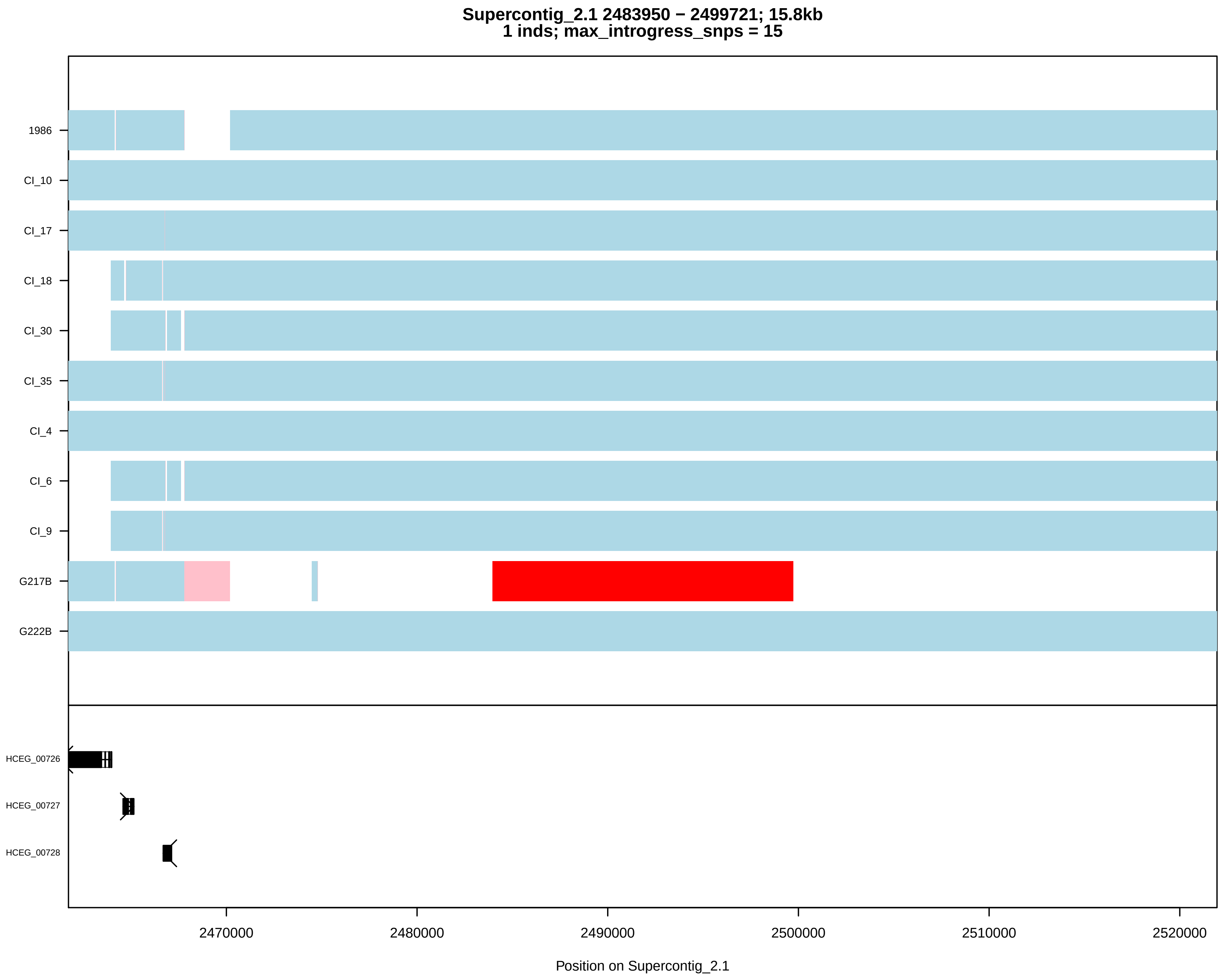
Supercontig_2.1 2483950 − 2499721; 15.8kb
1 inds; max_introgress_snps = 15
1986
CI_10
CI_17
CI_18
CI_30
CI_35
CI_4
CI_6
CI_9
G217B
G222B
HCEG_00726
HCEG_00727
HCEG_00728
2470000
2480000
2490000
2500000
2510000
2520000
Position on Supercontig_2.1

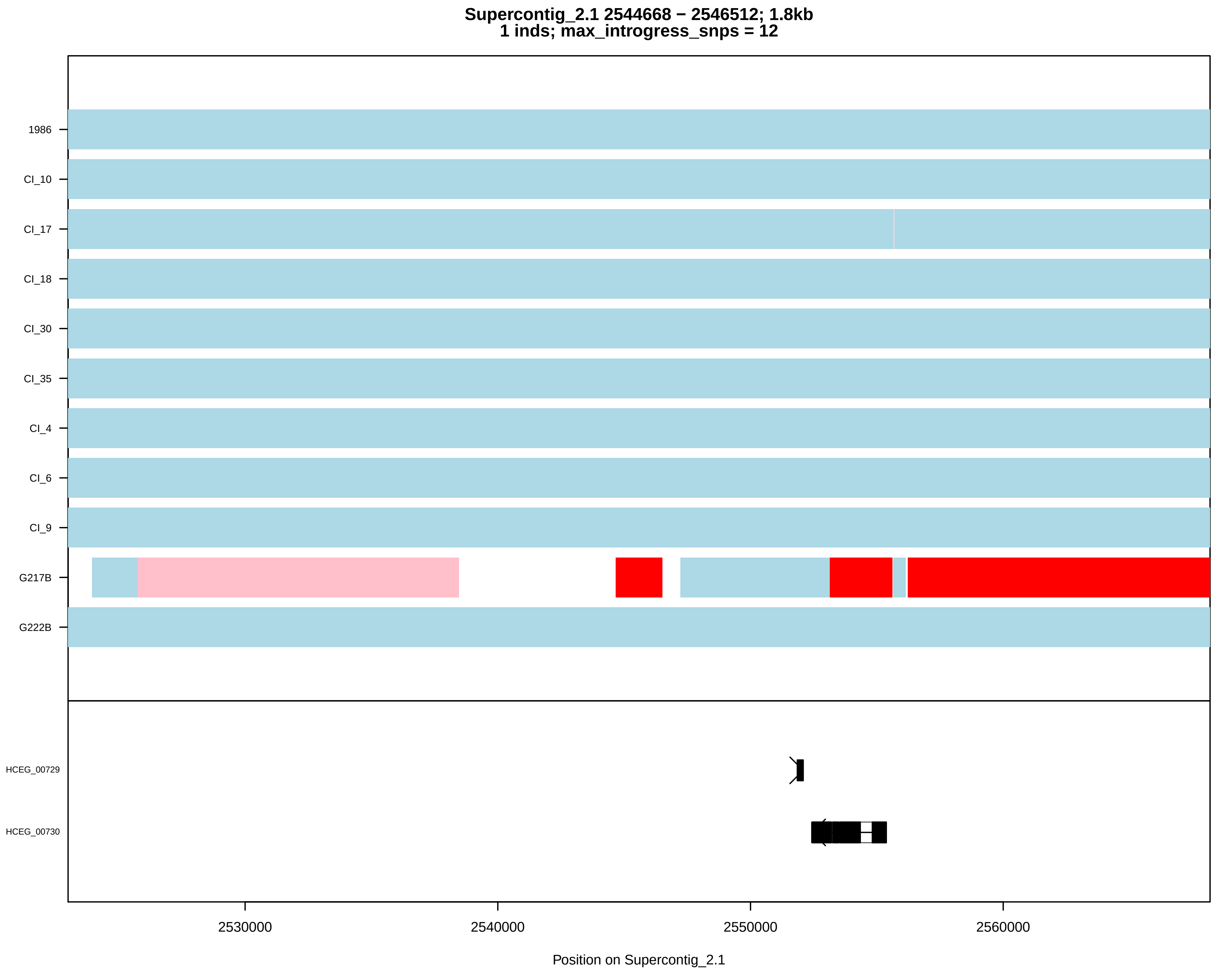
Supercontig_2.1 2544668 − 2546512; 1.8kb
1 inds; max_introgress_snps = 12
1986
CI_10
CI_17
CI_18
CI_30
CI_35
CI_4
CI_6
CI_9
G217B
G222B
HCEG_00729
HCEG_00730
2530000
2540000
2550000
2560000
Position on Supercontig_2.1

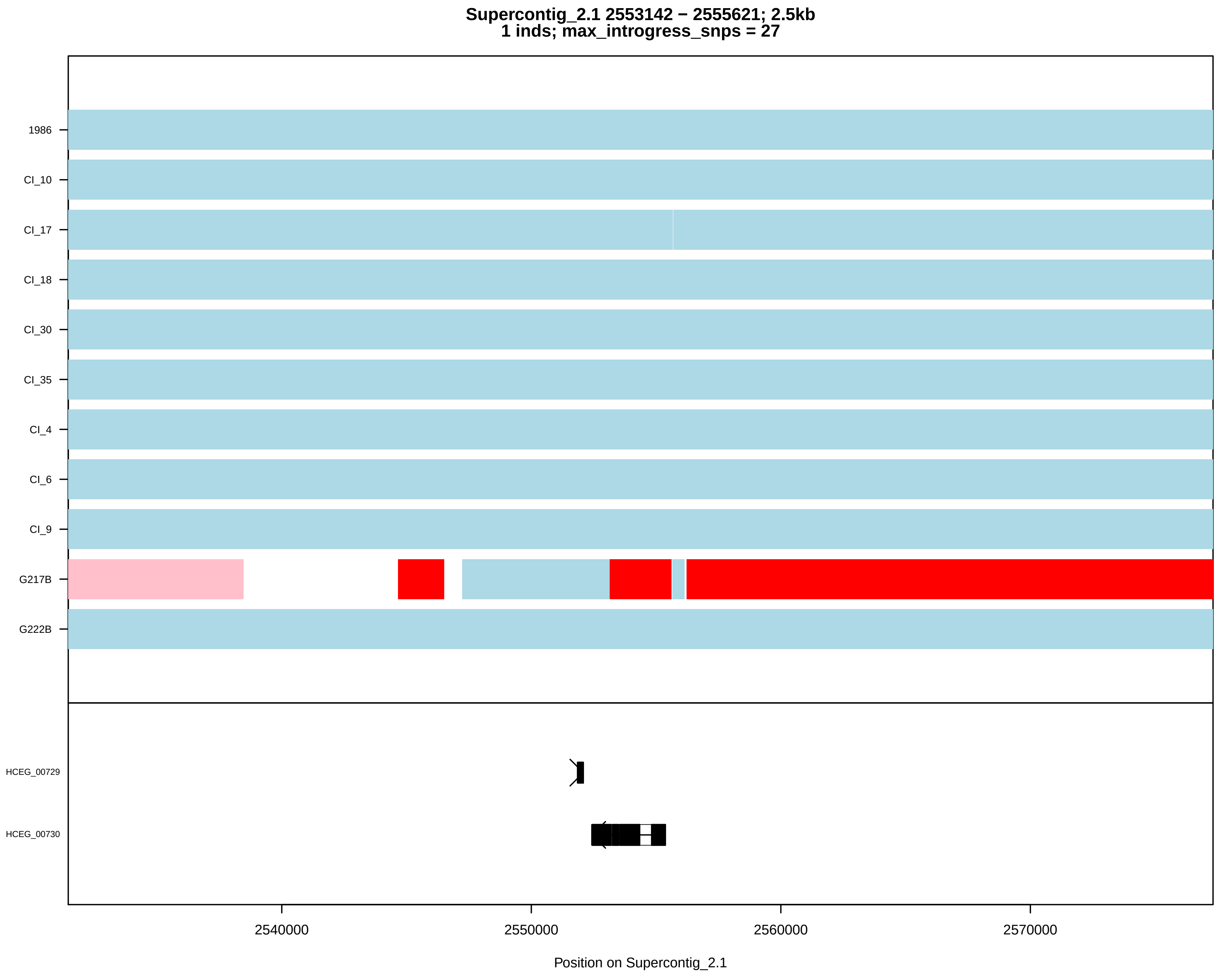
Supercontig_2.1 2553142 − 2555621; 2.5kb
1 inds; max_introgress_snps = 27
1986
CI_10
CI_17
CI_18
CI_30
CI_35
CI_4
CI_6
CI_9
G217B
G222B
HCEG_00729
HCEG_00730
2540000
2550000
2560000
2570000
Position on Supercontig_2.1

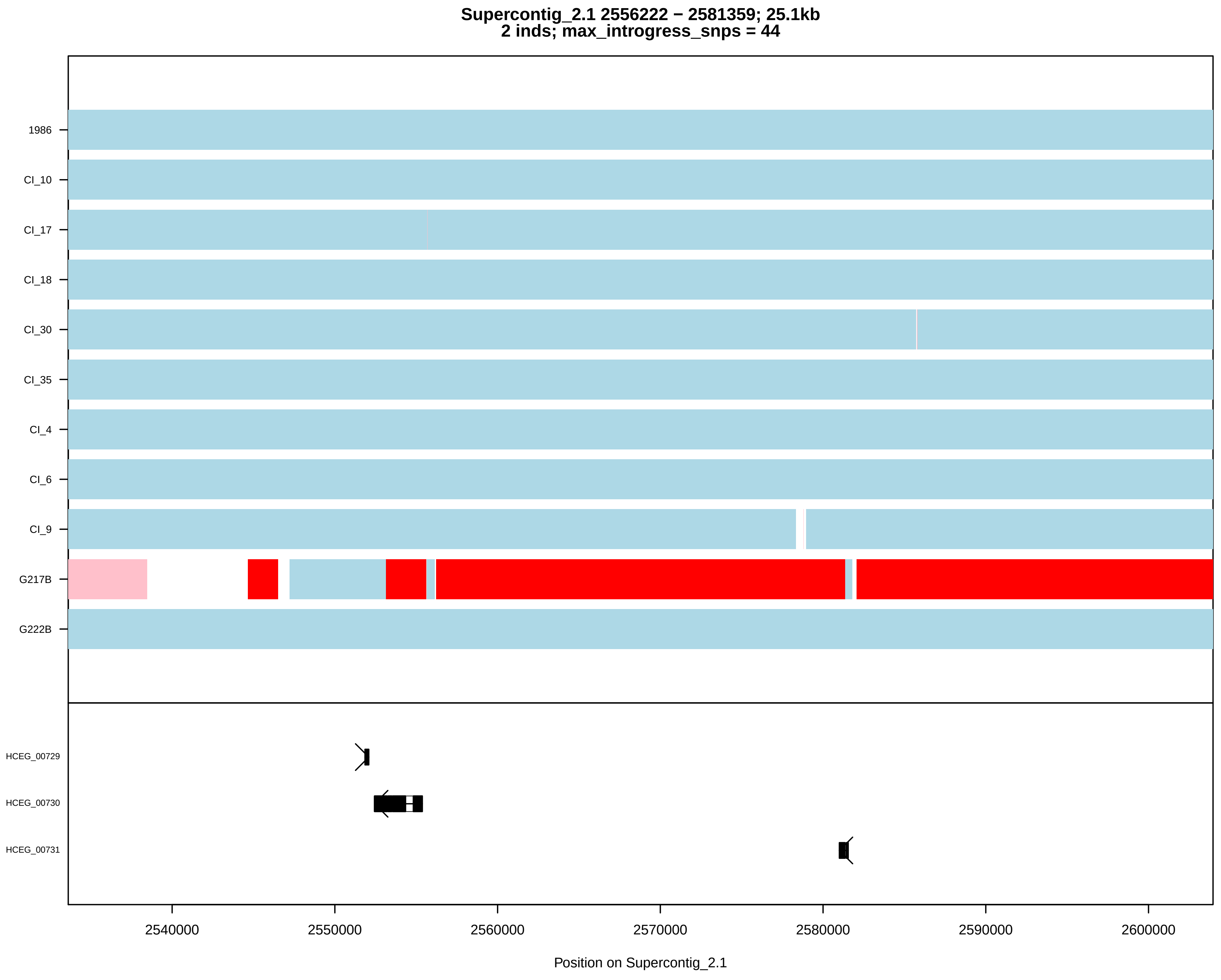

Supercontig_2.1 2556222 − 2581359; 25.1kb
2 inds; max_introgress_snps = 44
1986
CI_10
CI_17
CI_18
CI_30
CI_35
CI_4
CI_6
CI_9
G217B
G222B
HCEG_00729
HCEG_00730
HCEG_00731
2540000
2550000
2560000
2570000
2580000
2590000
2600000
Position on Supercontig_2.1

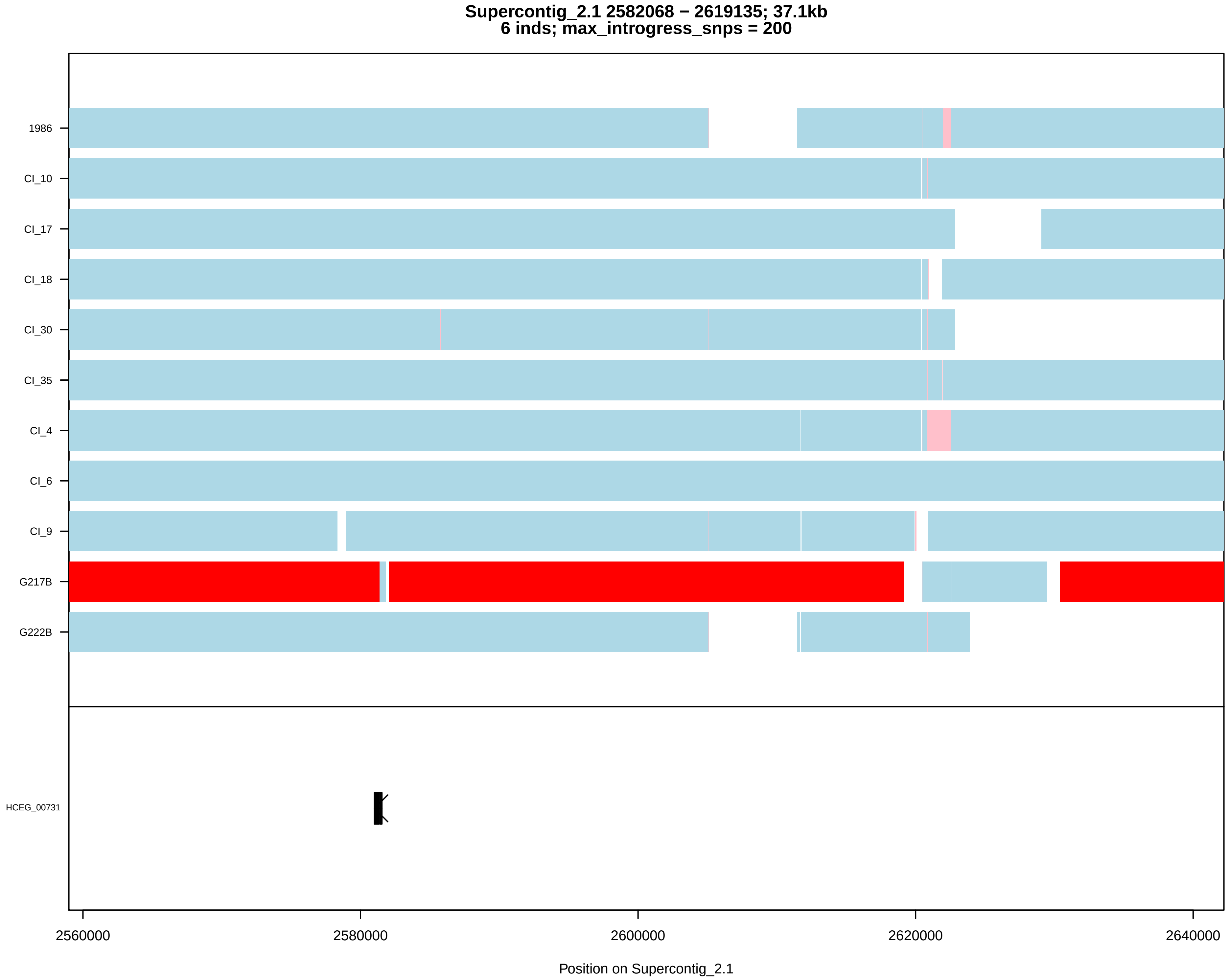

Supercontig_2.1 2582068 − 2619135; 37.1kb
6 inds; max_introgress_snps = 200
1986
CI_10
CI_17
CI_18
CI_30
CI_35
CI_4
CI_6
CI_9
G217B
G222B
HCEG_00731
2560000
2580000
2600000
2620000
2640000
Position on Supercontig_2.1

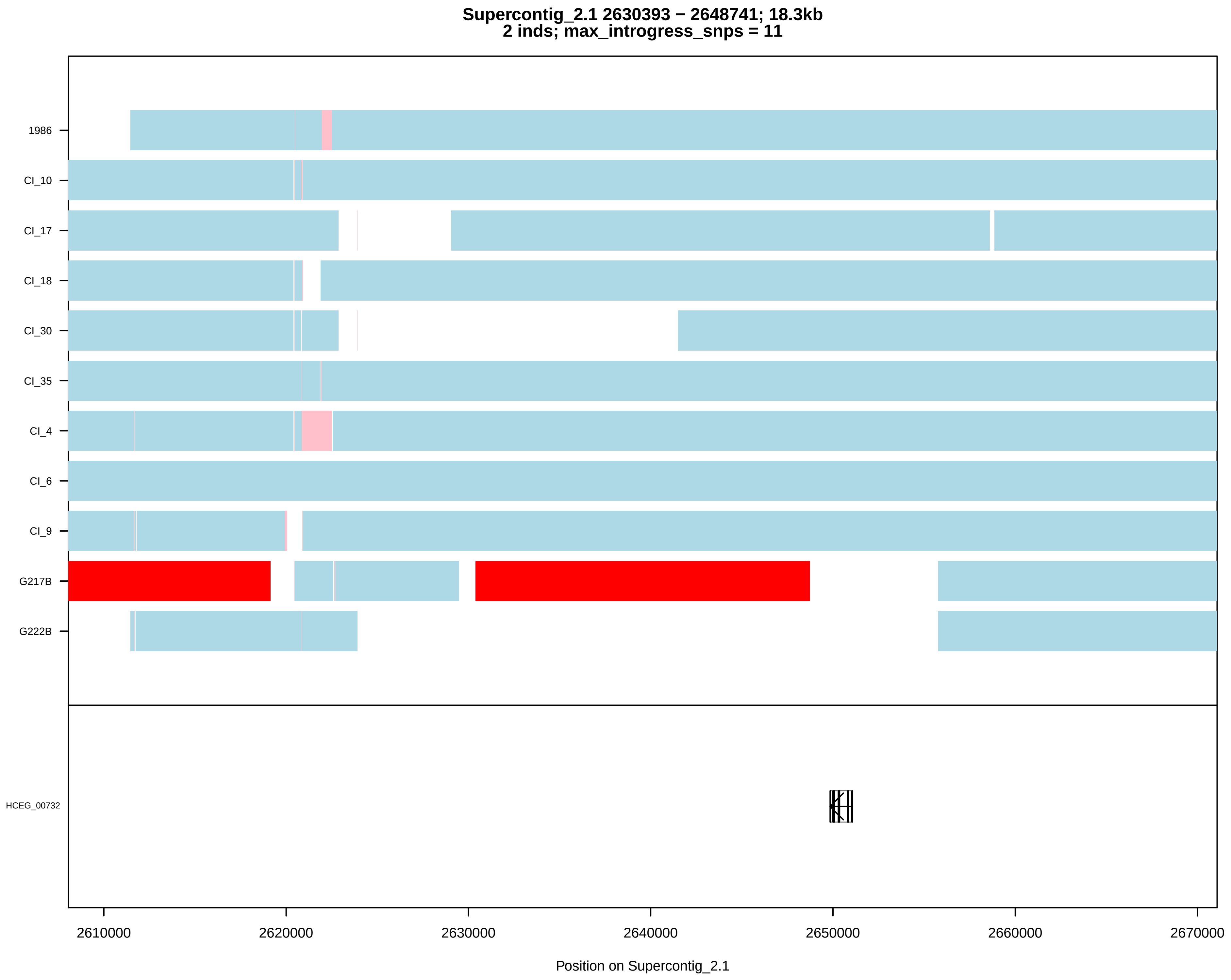
Supercontig_2.1 2630393 − 2648741; 18.3kb
2 inds; max_introgress_snps = 11
1986
CI_10
CI_17
CI_18
CI_30
CI_35
CI_4
CI_6
CI_9
G217B
G222B
HCEG_00732
2610000
2620000
2630000
2640000
2650000
2660000
2670000
Position on Supercontig_2.1

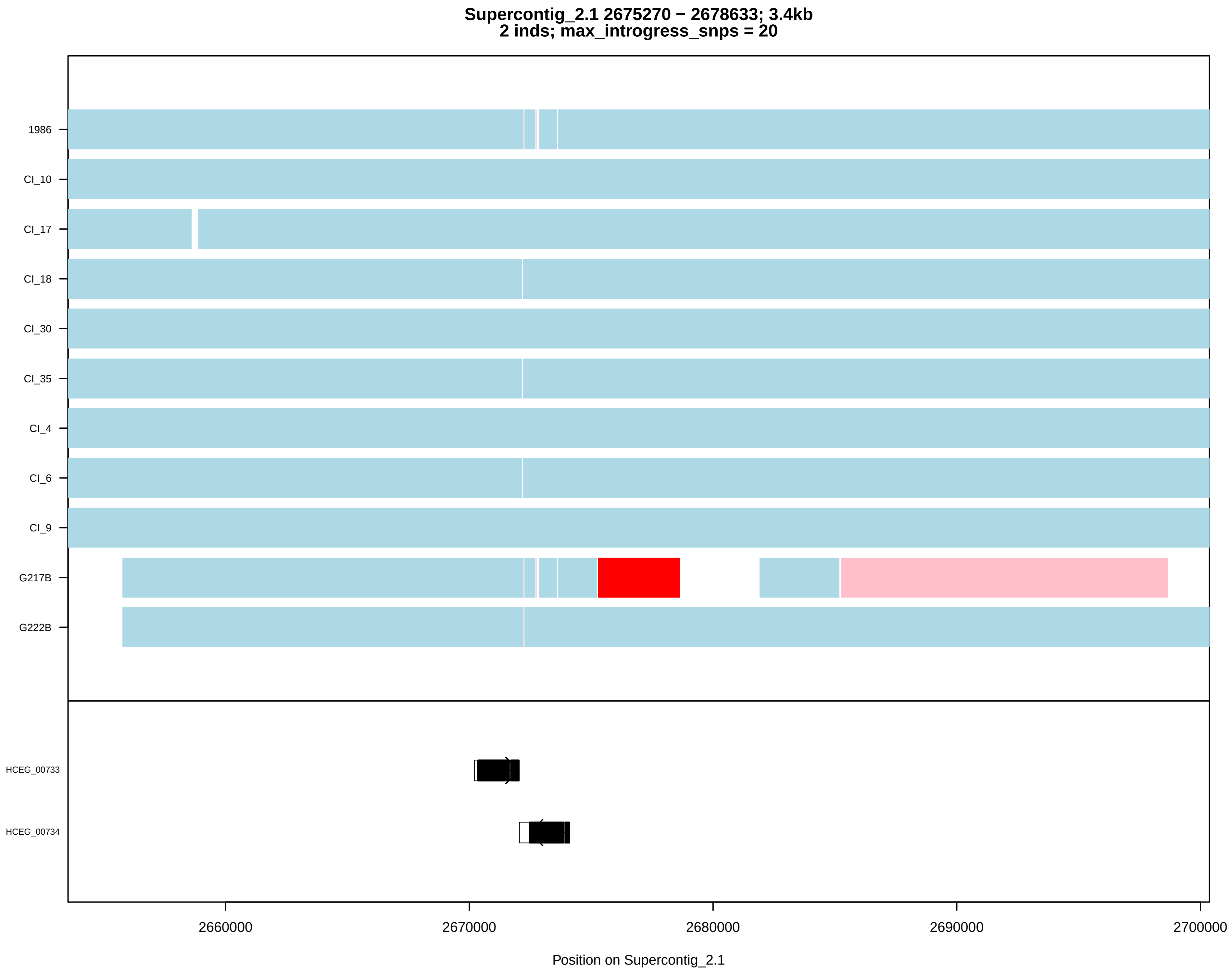
Supercontig_2.1 2675270 − 2678633; 3.4kb
2 inds; max_introgress_snps = 20
1986
CI_10
CI_17
CI_18
CI_30
CI_35
CI_4
CI_6
CI_9
G217B
G222B
HCEG_00733
HCEG_00734
2660000
2670000
2680000
2690000
2700000
Position on Supercontig_2.1

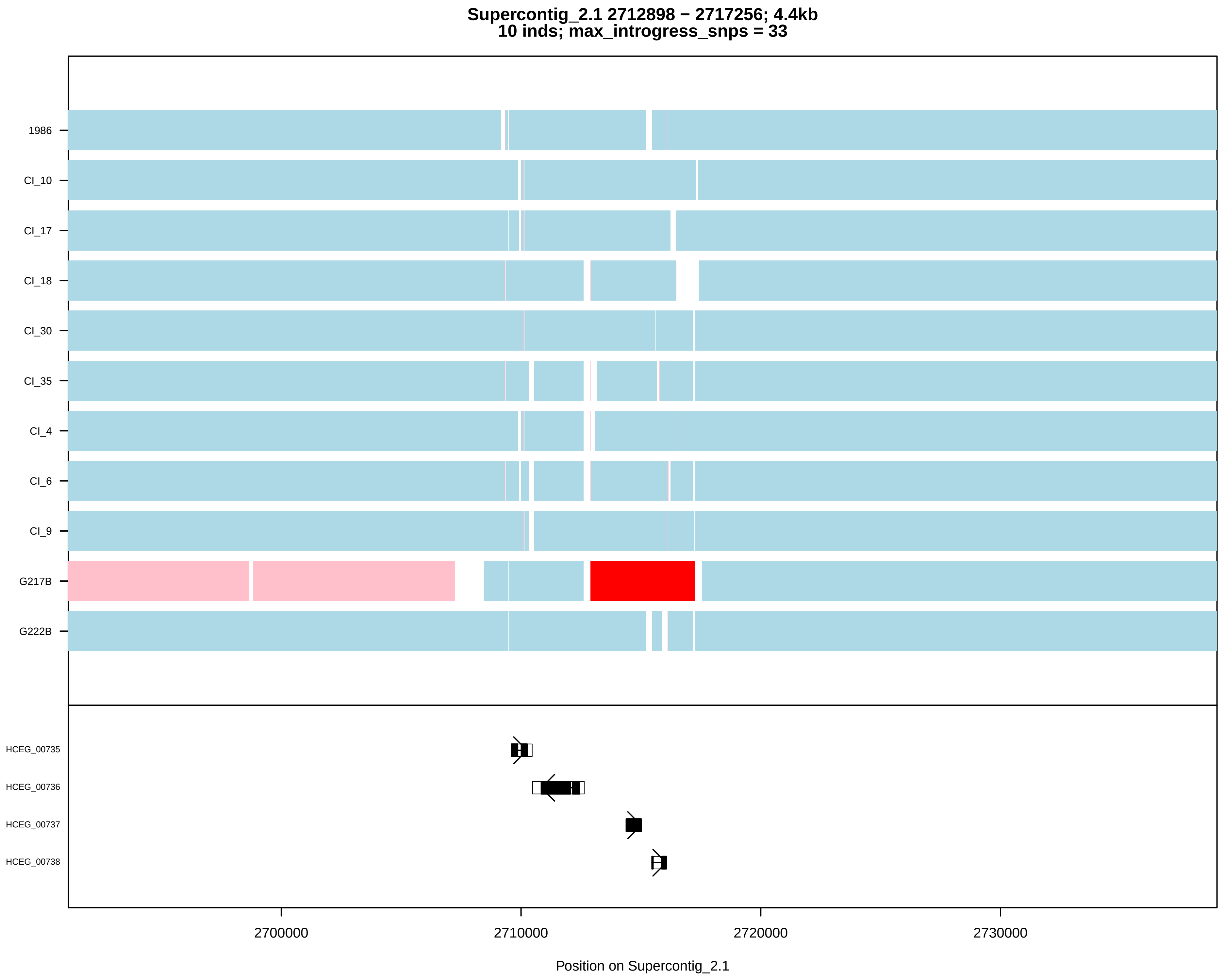
Supercontig_2.1 2712898 − 2717256; 4.4kb
10 inds; max_introgress_snps = 33
1986
CI_10
CI_17
CI_18
CI_30
CI_35
CI_4
CI_6
CI_9
G217B
G222B
HCEG_00735
HCEG_00736
HCEG_00737
HCEG_00738
2700000
2710000
2720000
2730000
Position on Supercontig_2.1

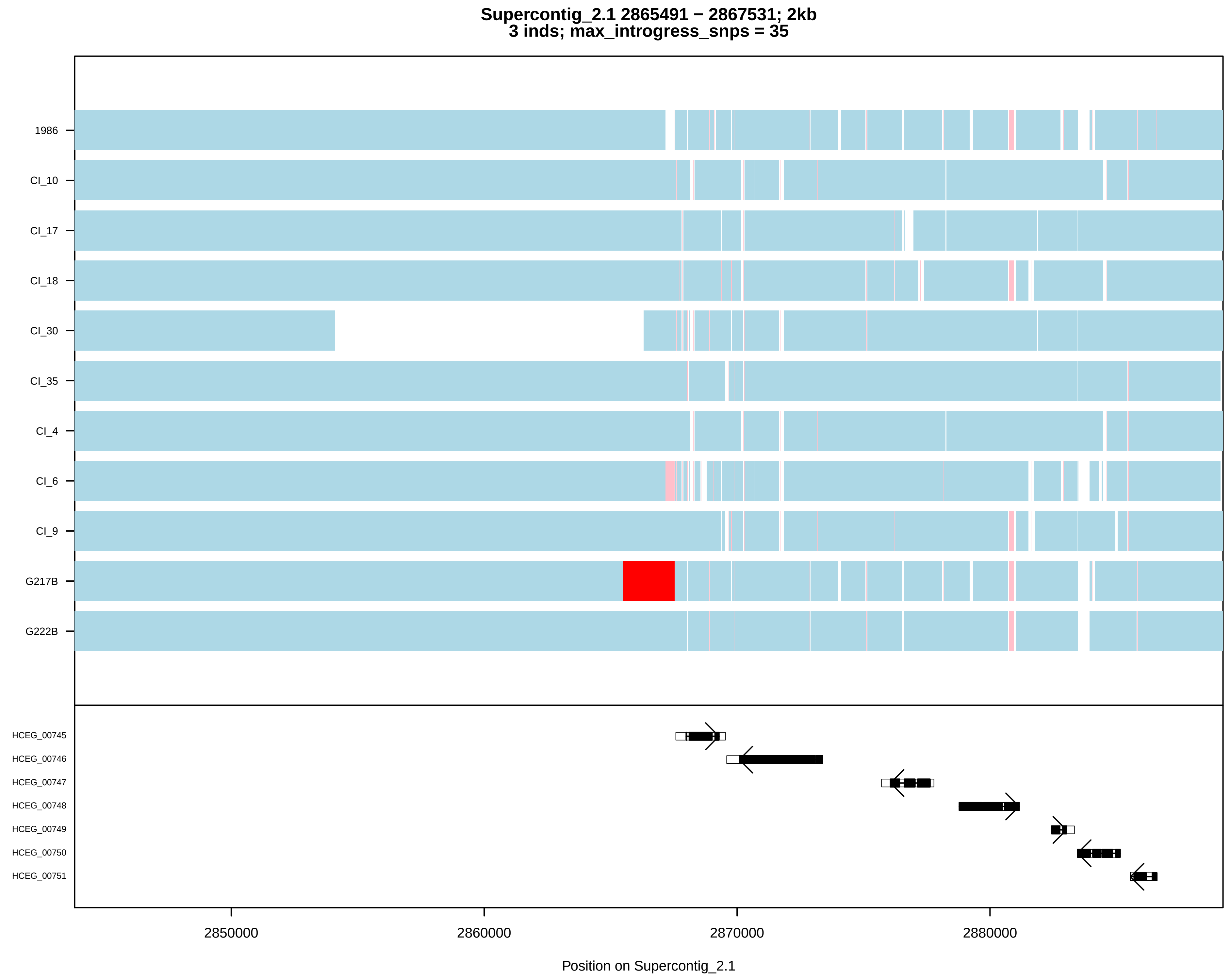

Supercontig_2.1 2865491 − 2867531; 2kb
3 inds; max_introgress_snps = 35
1986
CI_10
CI_17
CI_18
CI_30
CI_35
CI_4
CI_6
CI_9
G217B
G222B
HCEG_00745
HCEG_00746
HCEG_00747
HCEG_00748
HCEG_00749
HCEG_00750
HCEG_00751
2850000
2860000
2870000
2880000
Position on Supercontig_2.1

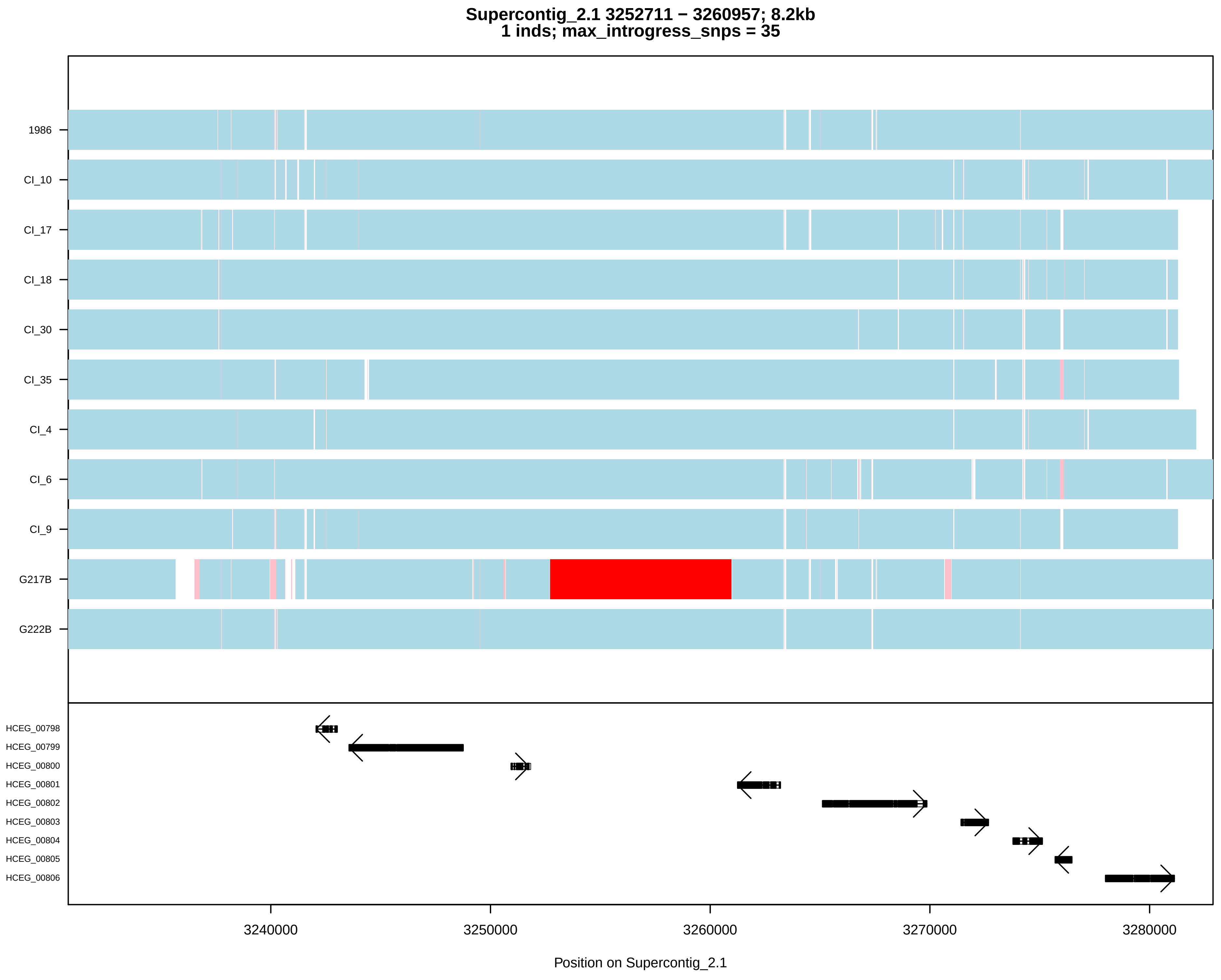

Supercontig_2.1 3252711 − 3260957; 8.2kb
1 inds; max_introgress_snps = 35
1986
CI_10
CI_17
CI_18
CI_30
CI_35
CI_4
CI_6
CI_9
G217B
G222B
HCEG_00798
HCEG_00799
HCEG_00800
HCEG_00801
HCEG_00802
HCEG_00803
HCEG_00804
HCEG_00805
HCEG_00806
3240000
3250000
3260000
3270000
3280000
Position on Supercontig_2.1

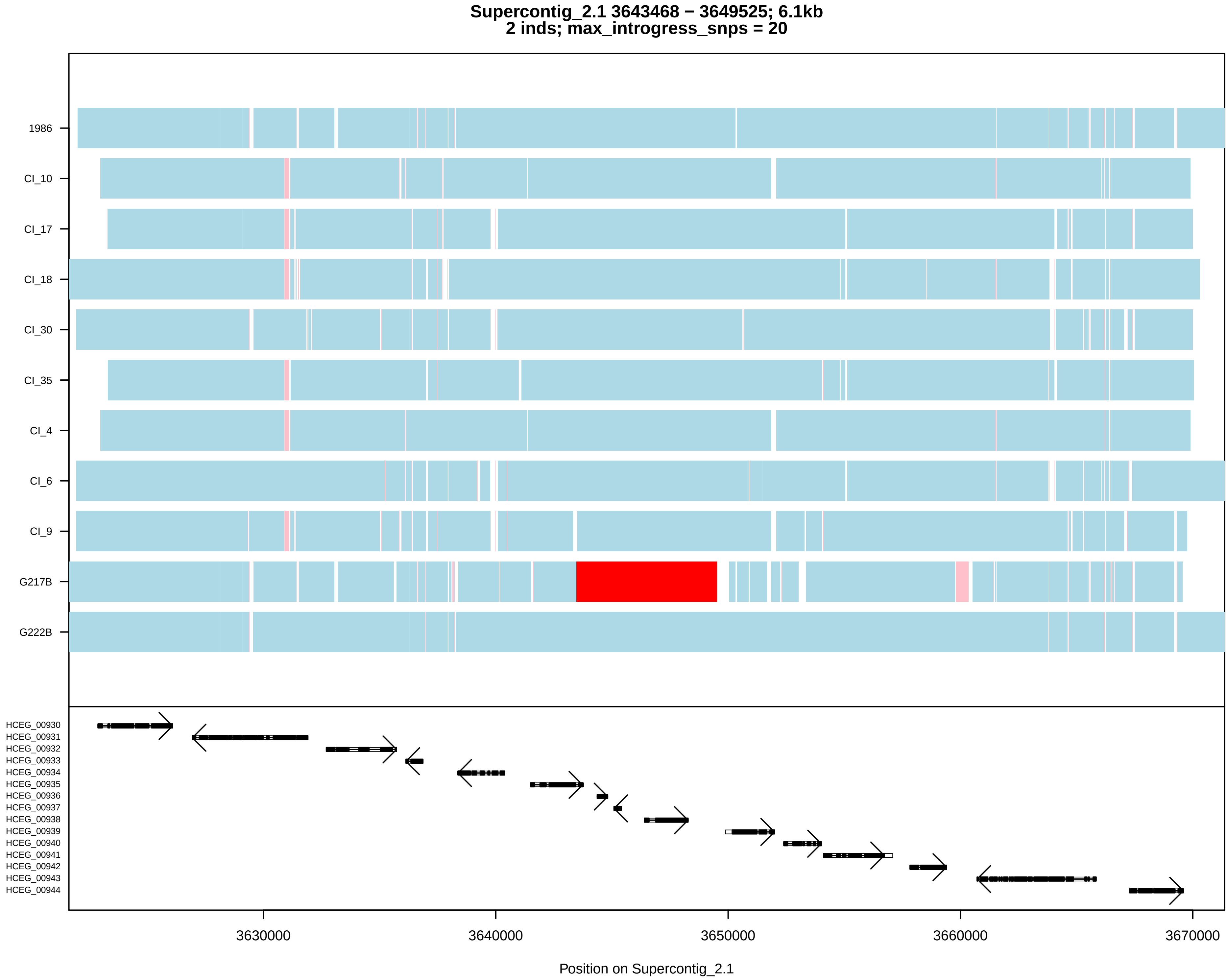

Supercontig_2.1 3643468 − 3649525; 6.1kb
2 inds; max_introgress_snps = 20
1986
CI_10
CI_17
CI_18
CI_30
CI_35
CI_4
CI_6
CI_9
G217B
G222B
HCEG_00930
HCEG_00931
HCEG_00932
HCEG_00933
HCEG_00934
HCEG_00935
HCEG_00936
HCEG_00937
HCEG_00938
HCEG_00939
HCEG_00940
HCEG_00941
HCEG_00942
HCEG_00943
HCEG_00944
3630000
3640000
3650000
3660000
3670000
Position on Supercontig_2.1

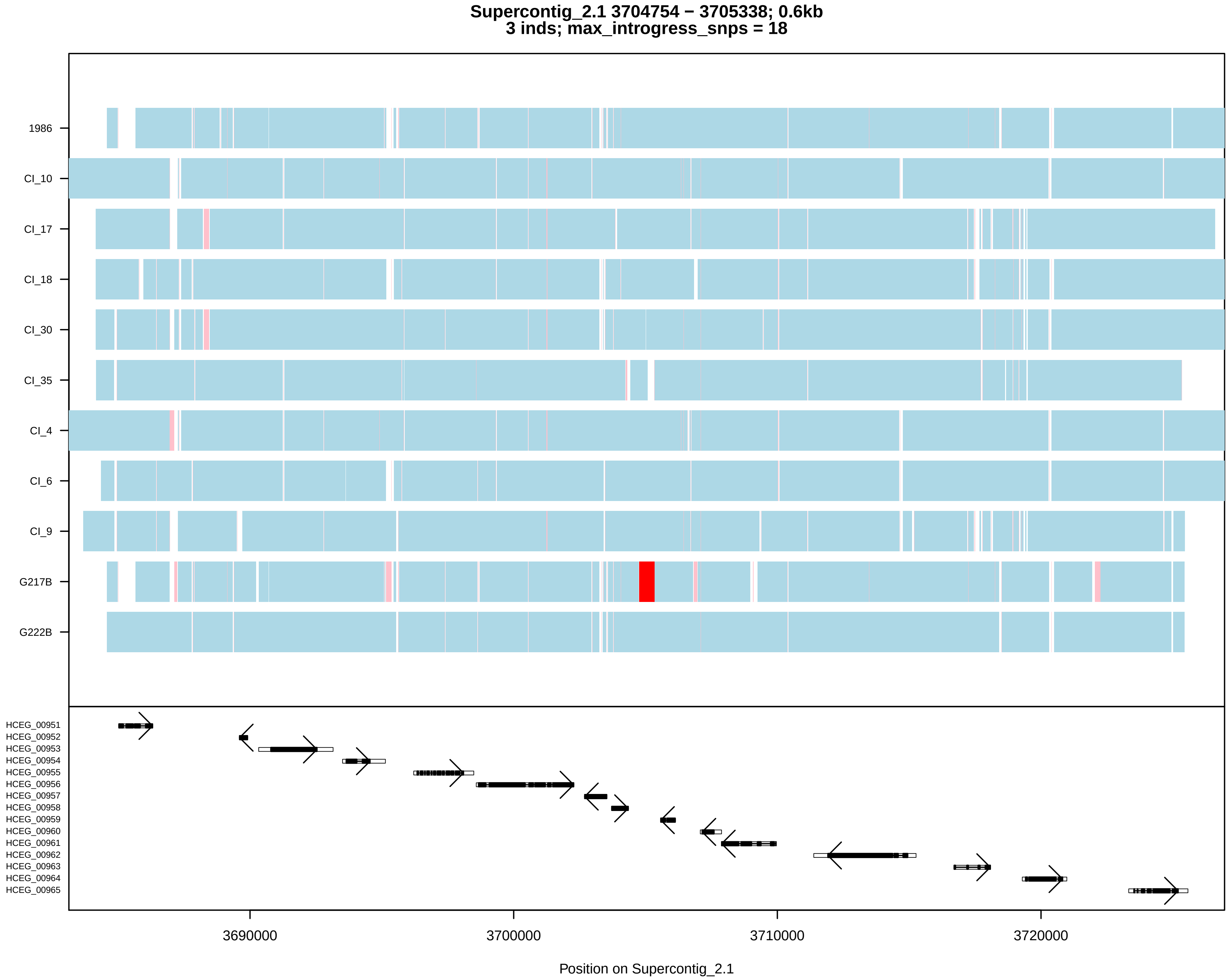

Supercontig_2.1 3704754 − 3705338; 0.6kb
3 inds; max_introgress_snps = 18
1986
CI_10
CI_17
CI_18
CI_30
CI_35
CI_4
CI_6
CI_9
G217B
G222B
HCEG_00951
HCEG_00952
HCEG_00953
HCEG_00954
HCEG_00955
HCEG_00956
HCEG_00957
HCEG_00958
HCEG_00959
HCEG_00960
HCEG_00961
HCEG_00962
HCEG_00963
HCEG_00964
HCEG_00965
3690000
3700000
3710000
3720000
Position on Supercontig_2.1

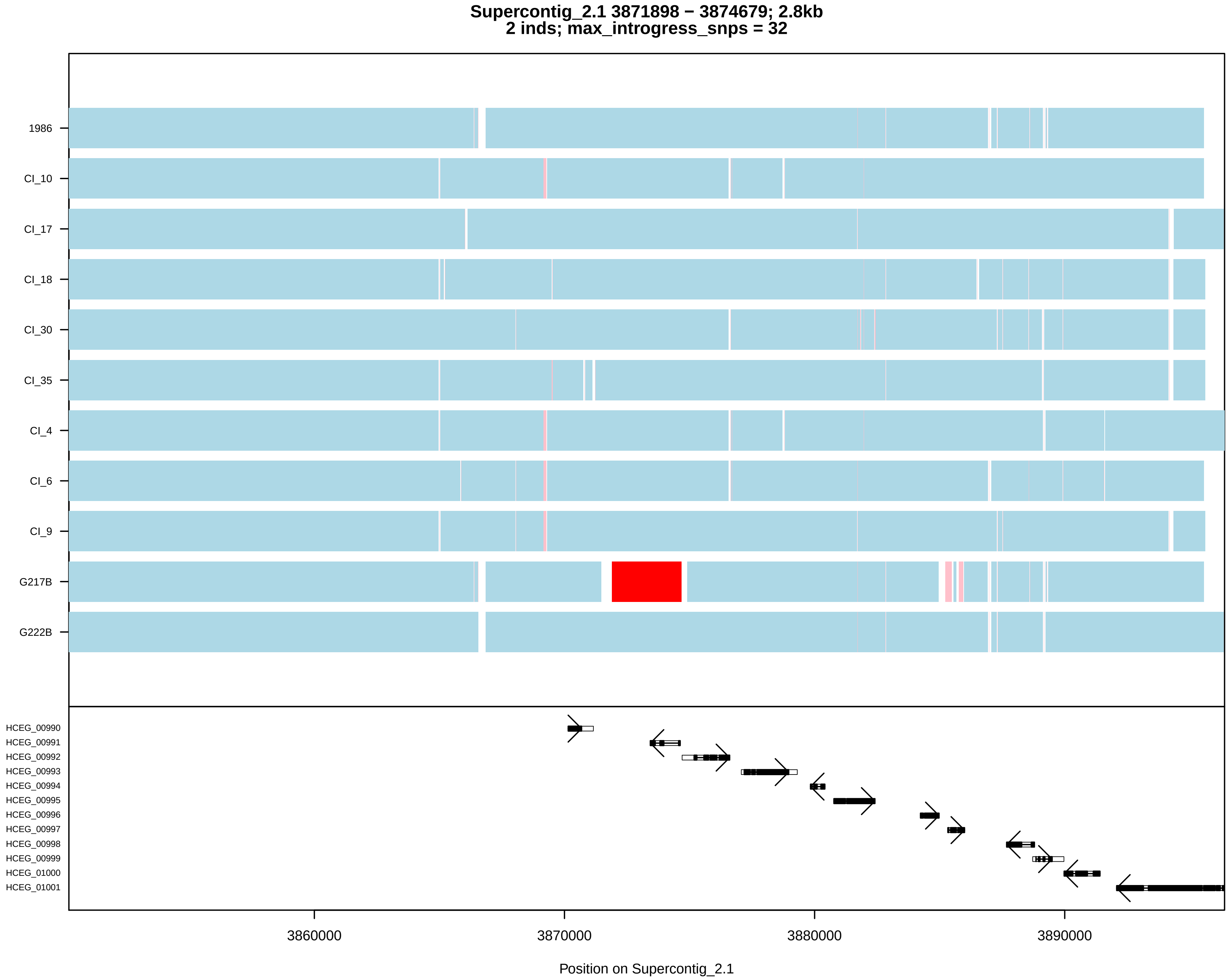

Supercontig_2.1 3871898 − 3874679; 2.8kb
2 inds; max_introgress_snps = 32
1986
CI_10
CI_17
CI_18
CI_30
CI_35
CI_4
CI_6
CI_9
G217B
G222B
HCEG_00990
HCEG_00991
HCEG_00992
HCEG_00993
HCEG_00994
HCEG_00995
HCEG_00996
HCEG_00997
HCEG_00998
HCEG_00999
HCEG_01000
HCEG_01001
3860000
3870000
3880000
3890000
Position on Supercontig_2.1

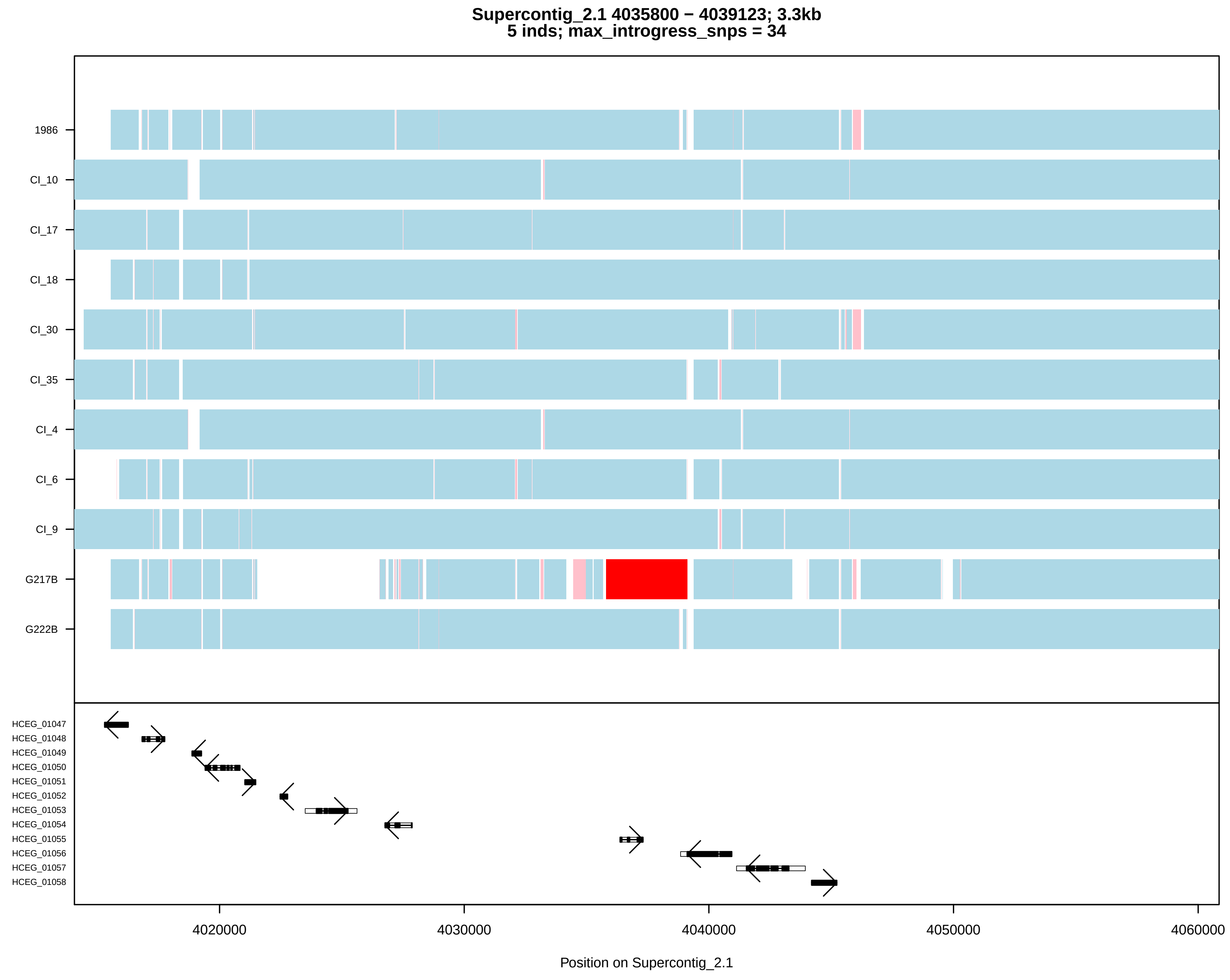
Supercontig_2.1 4035800 − 4039123; 3.3kb
5 inds; max_introgress_snps = 34
1986
CI_10
CI_17
CI_18
CI_30
CI_35
CI_4
CI_6
CI_9
G217B
G222B
HCEG_01047
HCEG_01048
HCEG_01049
HCEG_01050
HCEG_01051
HCEG_01052
HCEG_01053
HCEG_01054
HCEG_01055
HCEG_01056
HCEG_01057
HCEG_01058
4020000
4030000
4040000
4050000
4060000
Position on Supercontig_2.1

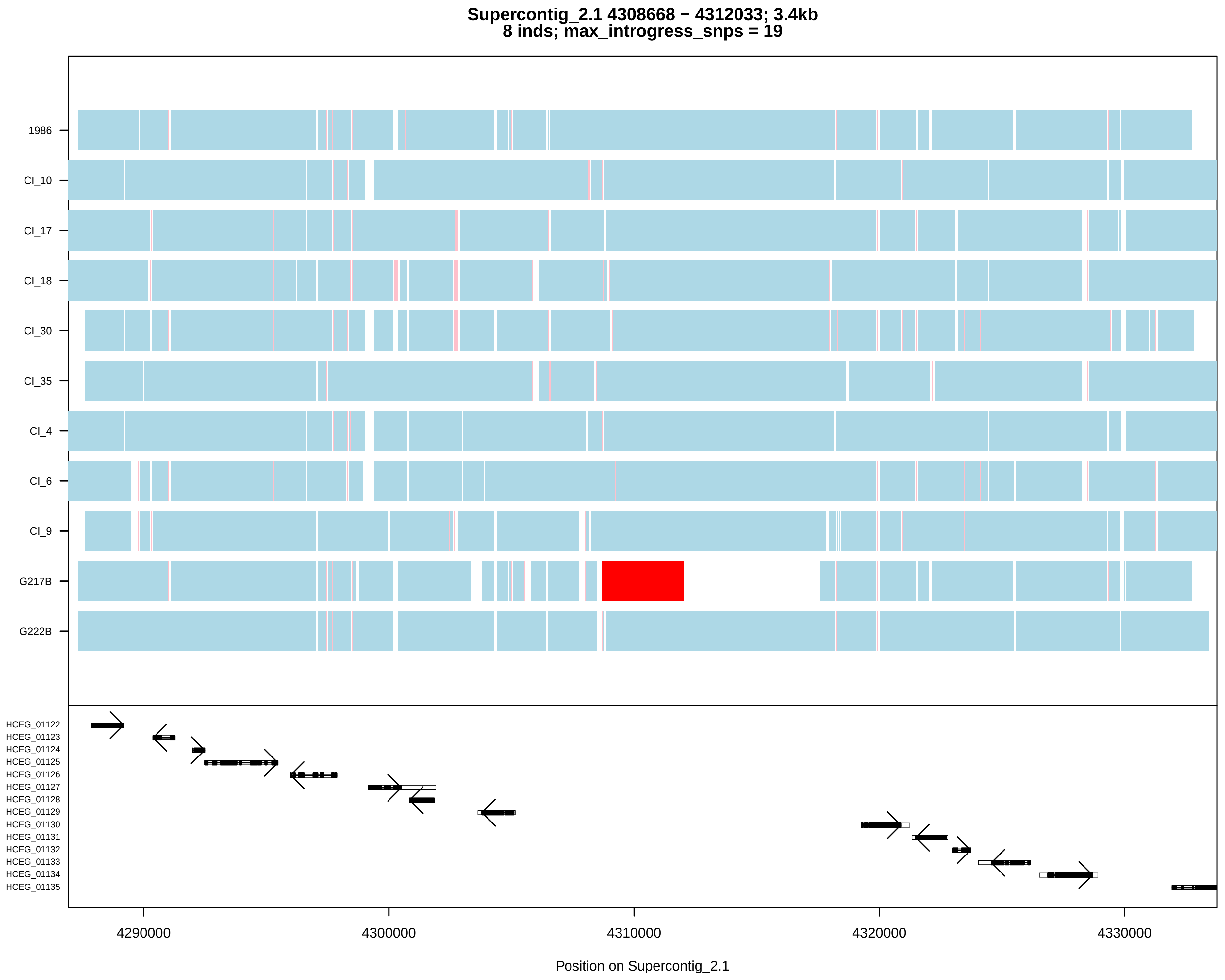
Supercontig_2.1 4308668 − 4312033; 3.4kb
8 inds; max_introgress_snps = 19
1986
CI_10
CI_17
CI_18
CI_30
CI_35
CI_4
CI_6
CI_9
G217B
G222B
HCEG_01122
HCEG_01123
HCEG_01124
HCEG_01125
HCEG_01126
HCEG_01127
HCEG_01128
HCEG_01129
HCEG_01130
HCEG_01131
HCEG_01132
HCEG_01133
HCEG_01134
HCEG_01135
4290000
4300000
4310000
4320000
4330000
Position on Supercontig_2.1

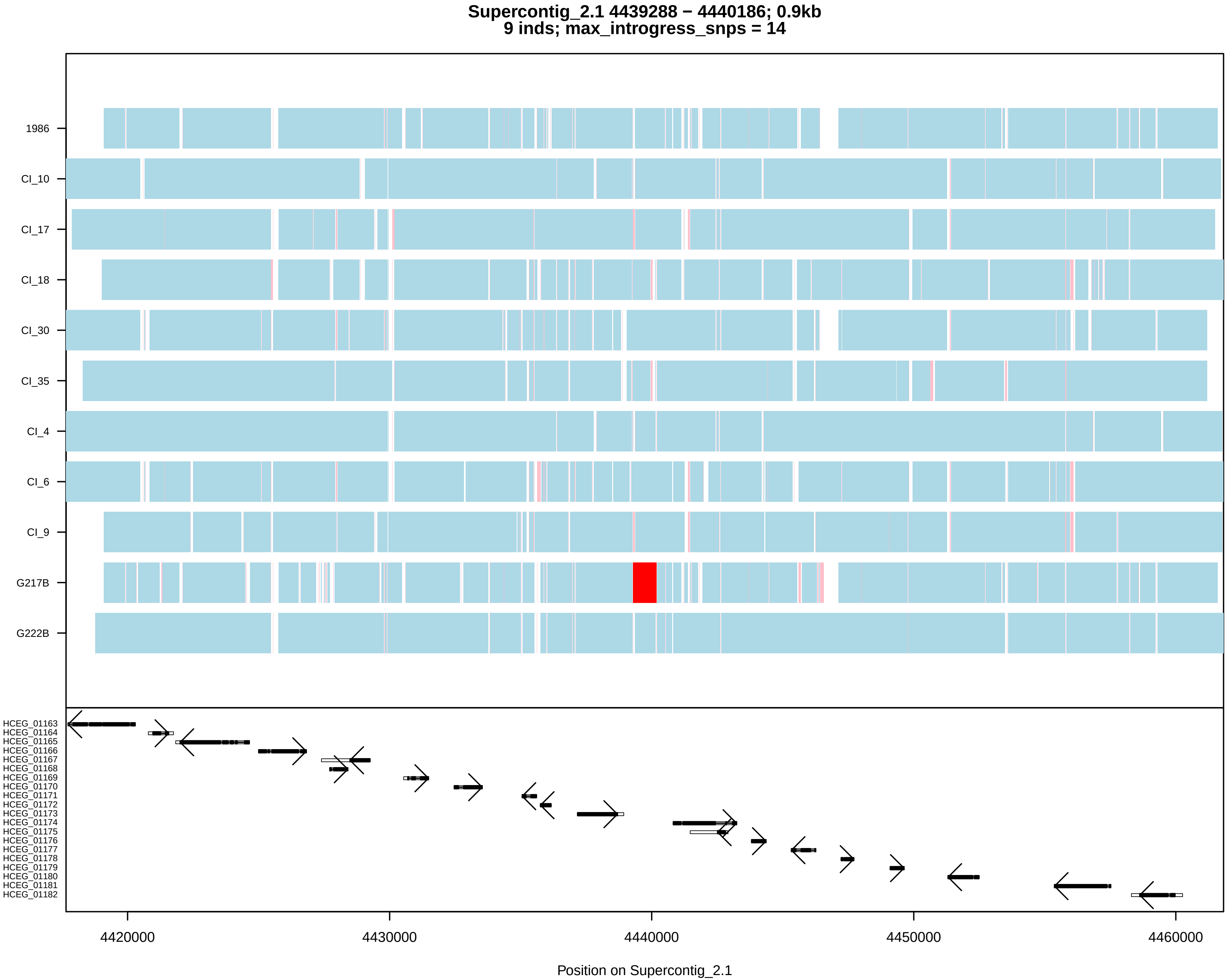
Supercontig_2.1 4439288 − 4440186; 0.9kb
9 inds; max_introgress_snps = 14
1986
CI_10
CI_17
CI_18
CI_30
CI_35
CI_4
CI_6
CI_9
G217B
G222B
HCEG_01163
HCEG_01164
HCEG_01165
HCEG_01166
HCEG_01167
HCEG_01168
HCEG_01169
HCEG_01170
HCEG_01171
HCEG_01172
HCEG_01173
HCEG_01174
HCEG_01175
HCEG_01176
HCEG_01177
HCEG_01178
HCEG_01179
HCEG_01180
HCEG_01181
HCEG_01182
4420000
4430000
4440000
4450000
4460000
Position on Supercontig_2.1

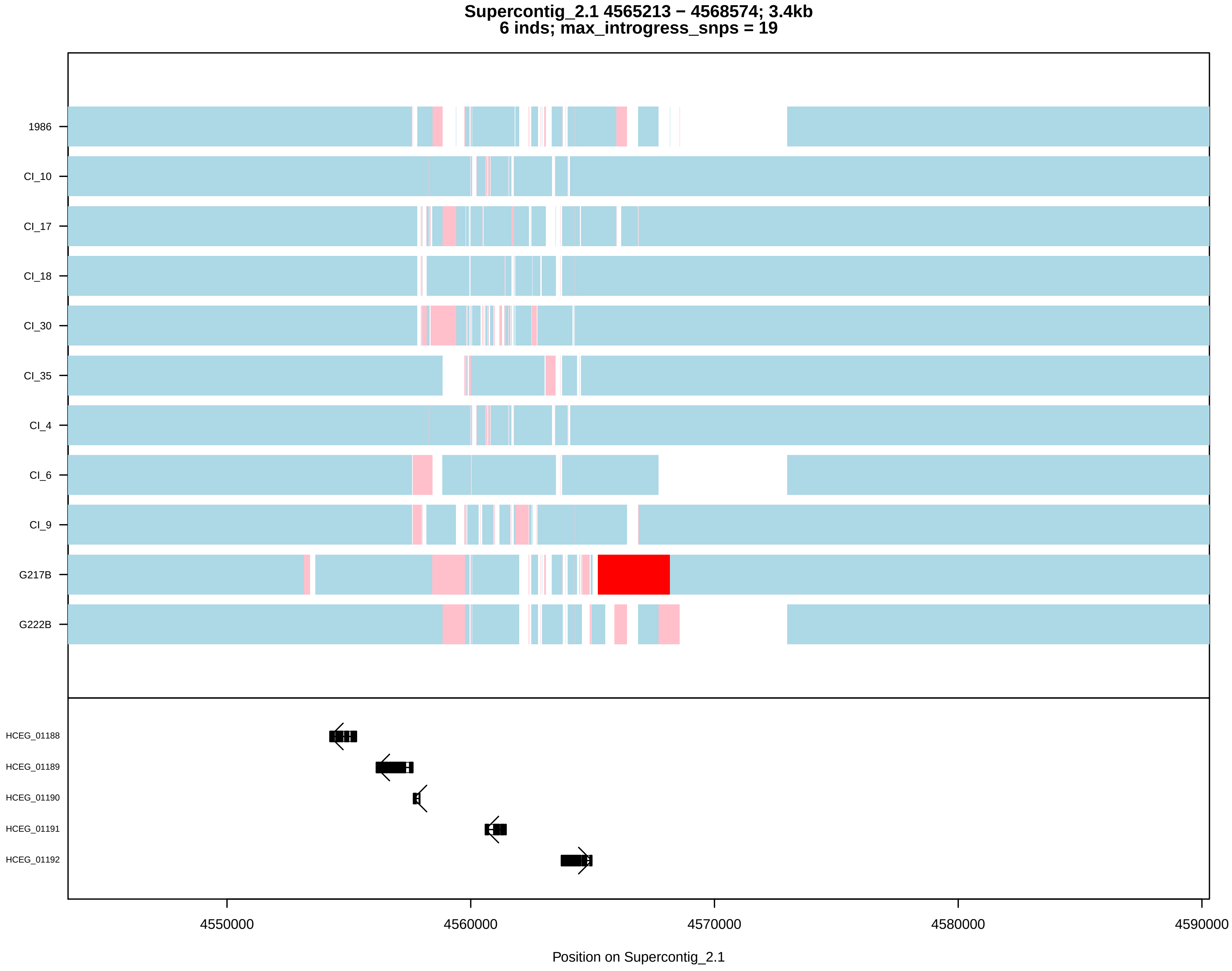

Supercontig_2.1 4565213 − 4568574; 3.4kb
6 inds; max_introgress_snps = 19
1986
CI_10
CI_17
CI_18
CI_30
CI_35
CI_4
CI_6
CI_9
G217B
G222B
HCEG_01188
HCEG_01189
HCEG_01190
HCEG_01191
HCEG_01192
4550000
4560000
4570000
4580000
4590000
Position on Supercontig_2.1

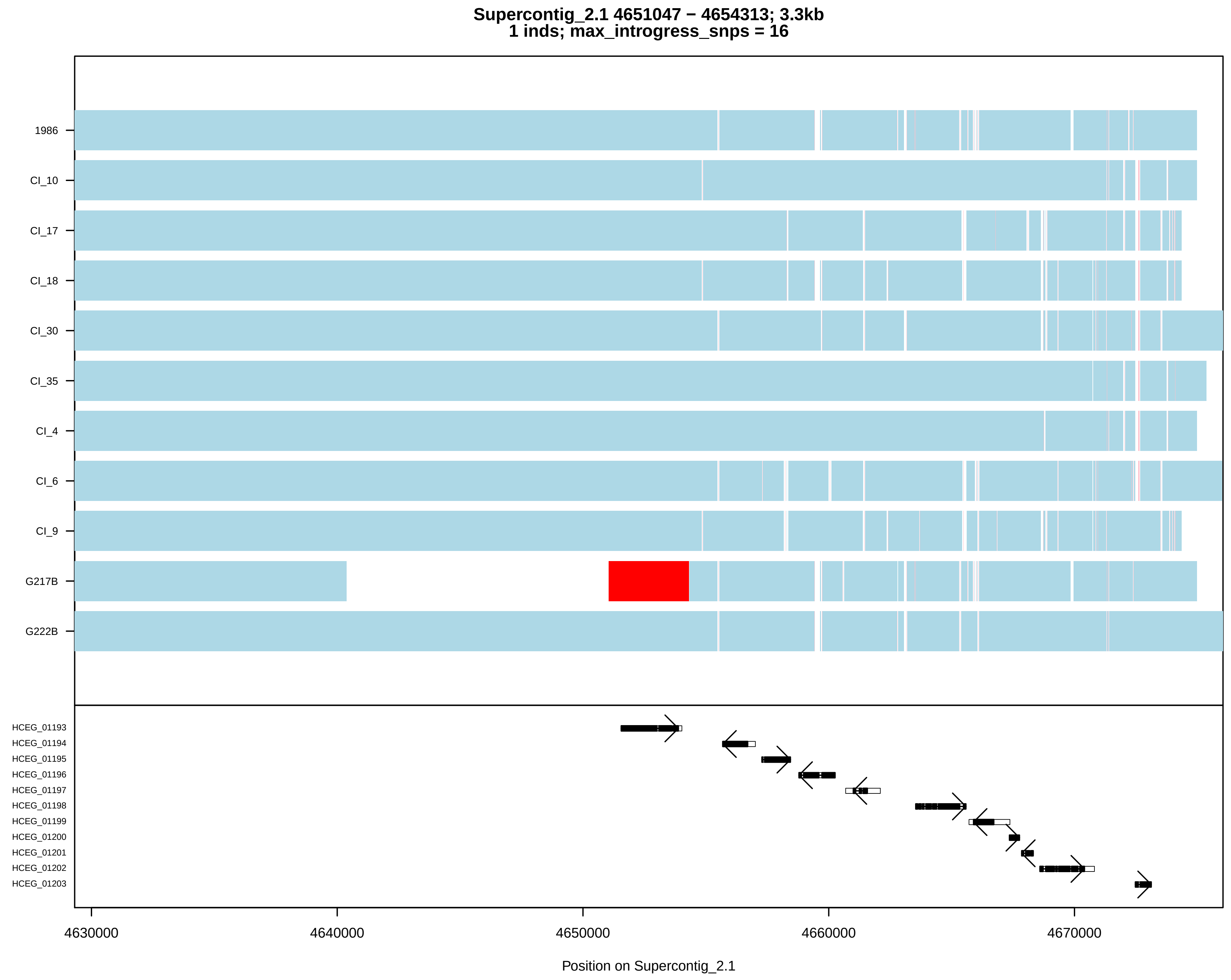

Supercontig_2.1 4651047 − 4654313; 3.3kb
1 inds; max_introgress_snps = 16
1986
CI_10
CI_17
CI_18
CI_30
CI_35
CI_4
CI_6
CI_9
G217B
G222B
HCEG_01193
HCEG_01194
HCEG_01195
HCEG_01196
HCEG_01197
HCEG_01198
HCEG_01199
HCEG_01200
HCEG_01201
HCEG_01202
HCEG_01203
4630000
4640000
4650000
4660000
4670000
Position on Supercontig_2.1

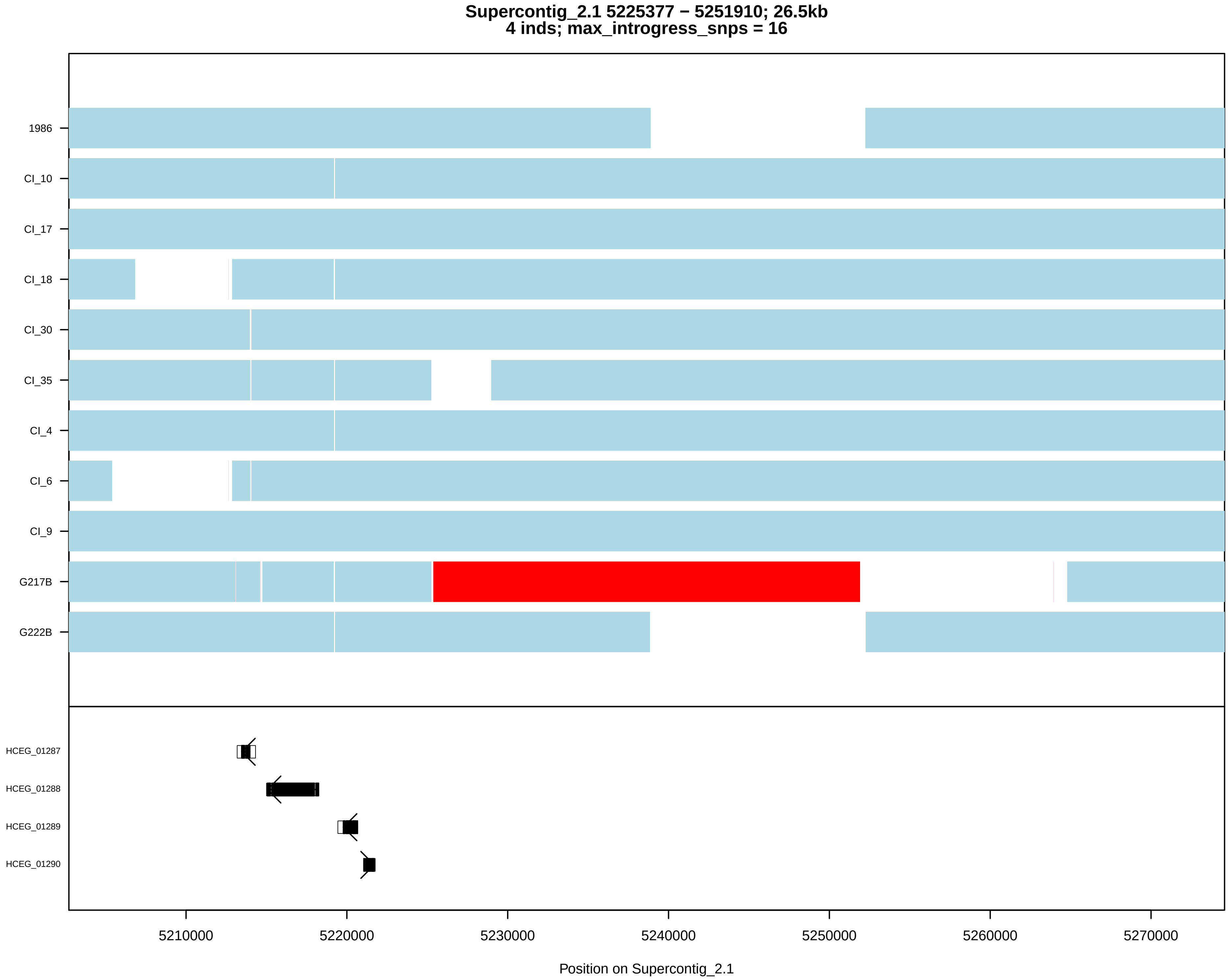

Supercontig_2.1 5225377 − 5251910; 26.5kb
4 inds; max_introgress_snps = 16
1986
CI_10
CI_17
CI_18
CI_30
CI_35
CI_4
CI_6
CI_9
G217B
G222B
HCEG_01287
HCEG_01288
HCEG_01289
HCEG_01290
5210000
5220000
5230000
5240000
5250000
5260000
5270000
Position on Supercontig_2.1

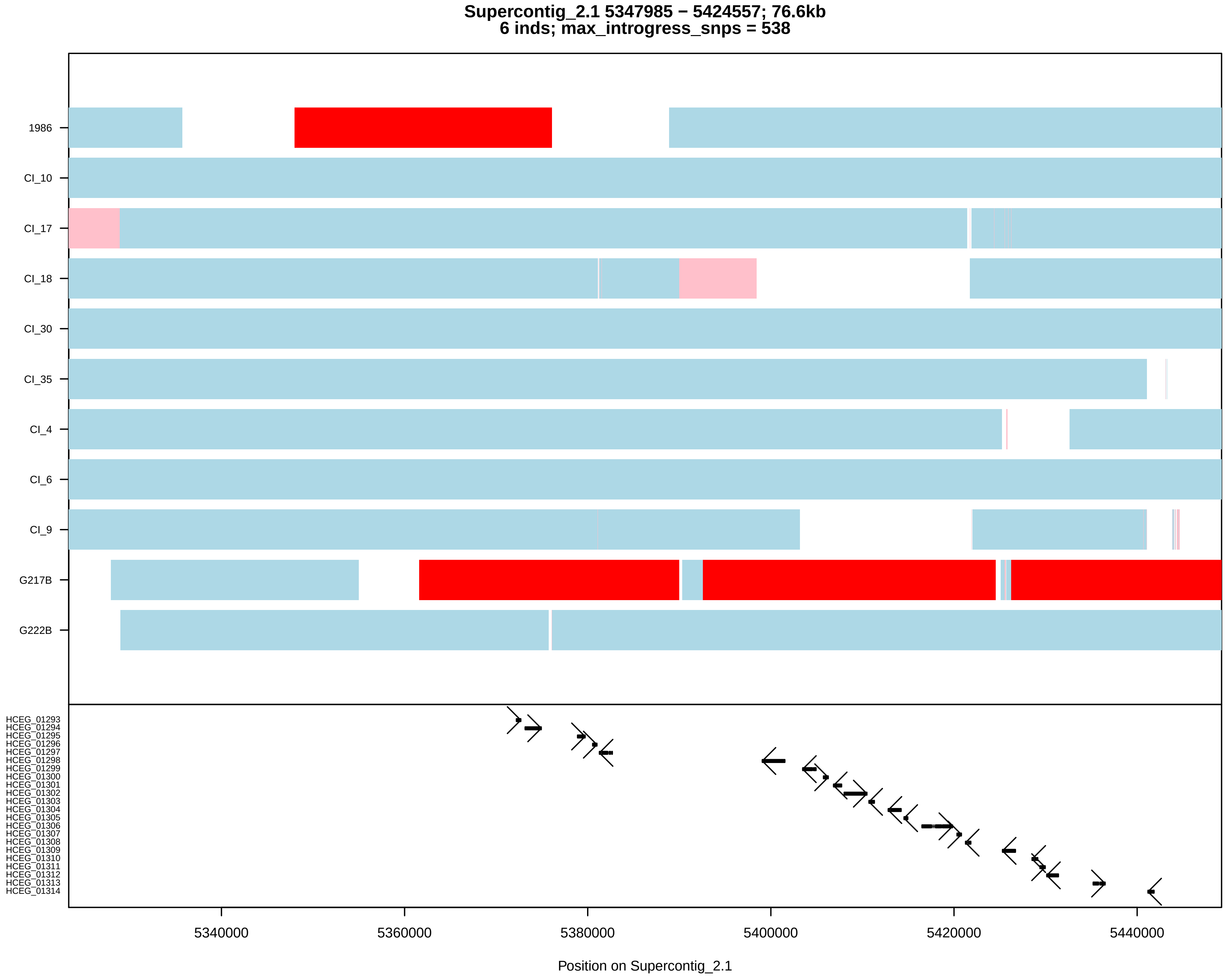

Supercontig_2.1 5347985 − 5424557; 76.6kb
6 inds; max_introgress_snps = 538
1986
CI_10
CI_17
CI_18
CI_30
CI_35
CI_4
CI_6
CI_9
G217B
G222B
HCEG_01293
HCEG_01294
HCEG_01295
HCEG_01296
HCEG_01297
HCEG_01298
HCEG_01299
HCEG_01300
HCEG_01301
HCEG_01302
HCEG_01303
HCEG_01304
HCEG_01305
HCEG_01306
HCEG_01307
HCEG_01308
HCEG_01309
HCEG_01310
HCEG_01311
HCEG_01312
HCEG_01313
HCEG_01314
5340000
5360000
5380000
5400000
5420000
5440000
Position on Supercontig_2.1

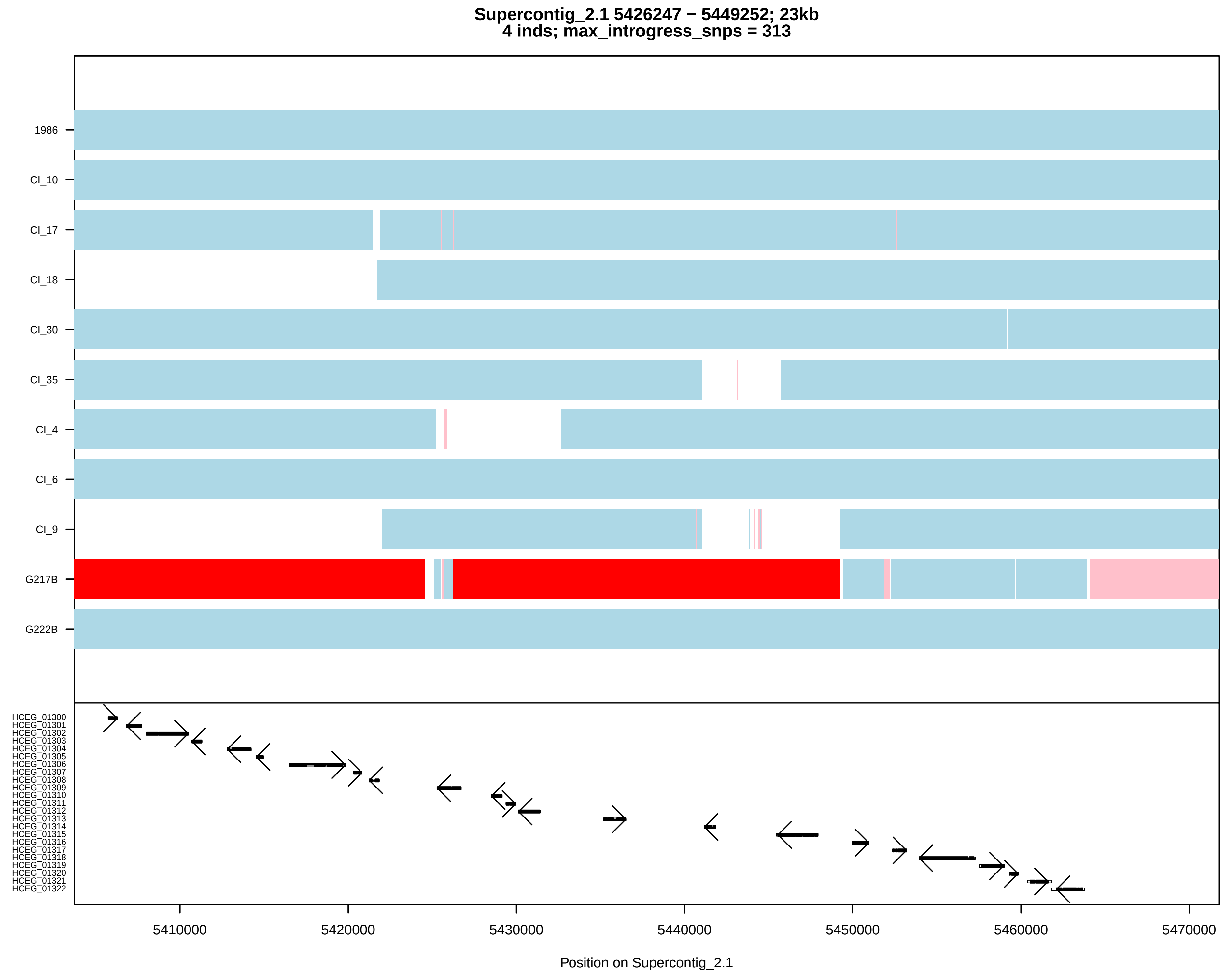

Supercontig_2.1 5426247 − 5449252; 23kb
4 inds; max_introgress_snps = 313
1986
CI_10
CI_17
CI_18
CI_30
CI_35
CI_4
CI_6
CI_9
G217B
G222B
HCEG_01300
HCEG_01301
HCEG_01302
HCEG_01303
HCEG_01304
HCEG_01305
HCEG_01306
HCEG_01307
HCEG_01308
HCEG_01309
HCEG_01310
HCEG_01311
HCEG_01312
HCEG_01313
HCEG_01314
HCEG_01315
HCEG_01316
HCEG_01317
HCEG_01318
HCEG_01319
HCEG_01320
HCEG_01321
HCEG_01322
5410000
5420000
5430000
5440000
5450000
5460000
5470000
Position on Supercontig_2.1

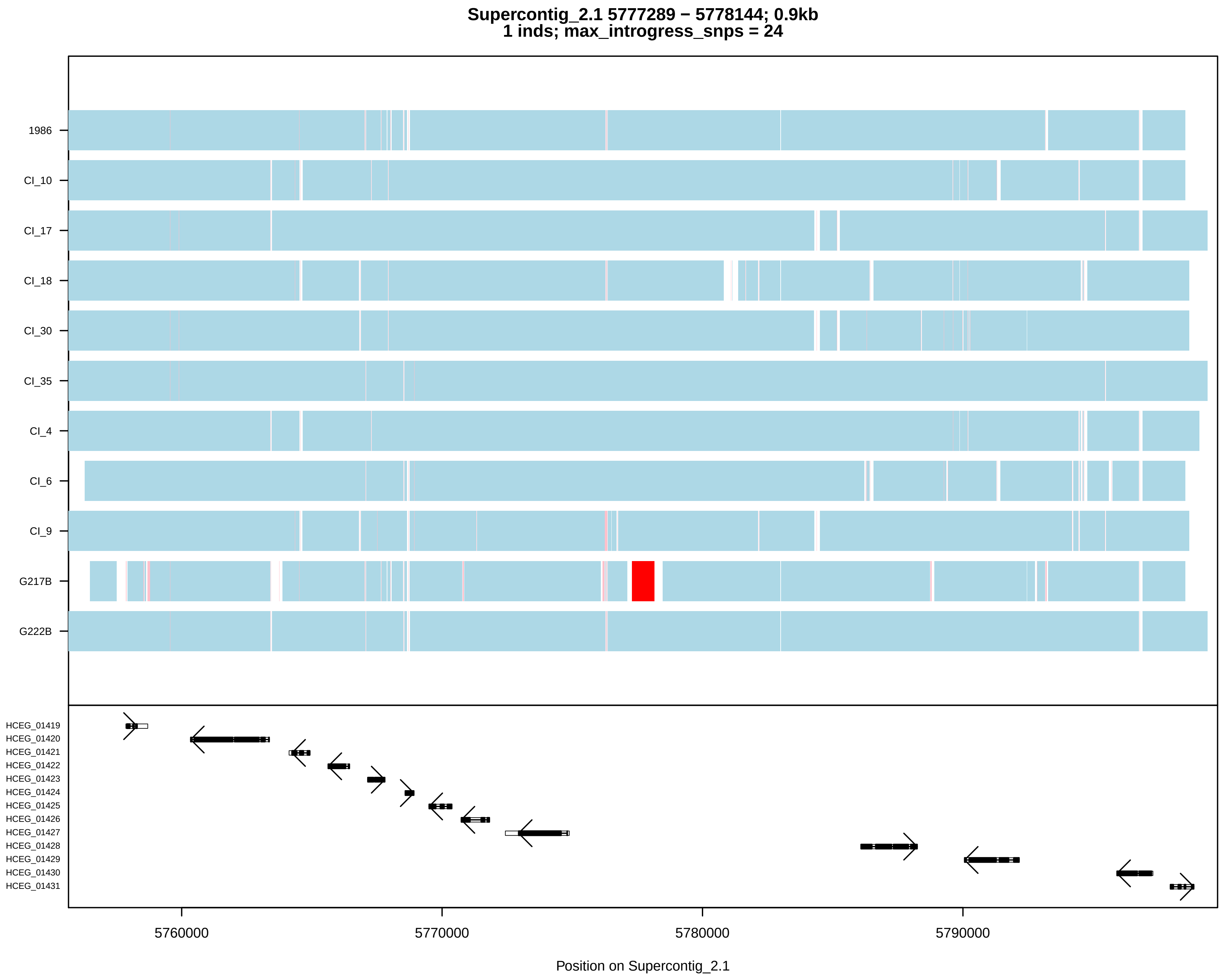
Supercontig_2.1 5777289 − 5778144; 0.9kb
1 inds; max_introgress_snps = 24
1986
CI_10
CI_17
CI_18
CI_30
CI_35
CI_4
CI_6
CI_9
G217B
G222B
HCEG_01419
HCEG_01420
HCEG_01421
HCEG_01422
HCEG_01423
HCEG_01424
HCEG_01425
HCEG_01426
HCEG_01427
HCEG_01428
HCEG_01429
HCEG_01430
HCEG_01431
5760000
5770000
5780000
5790000
Position on Supercontig_2.1

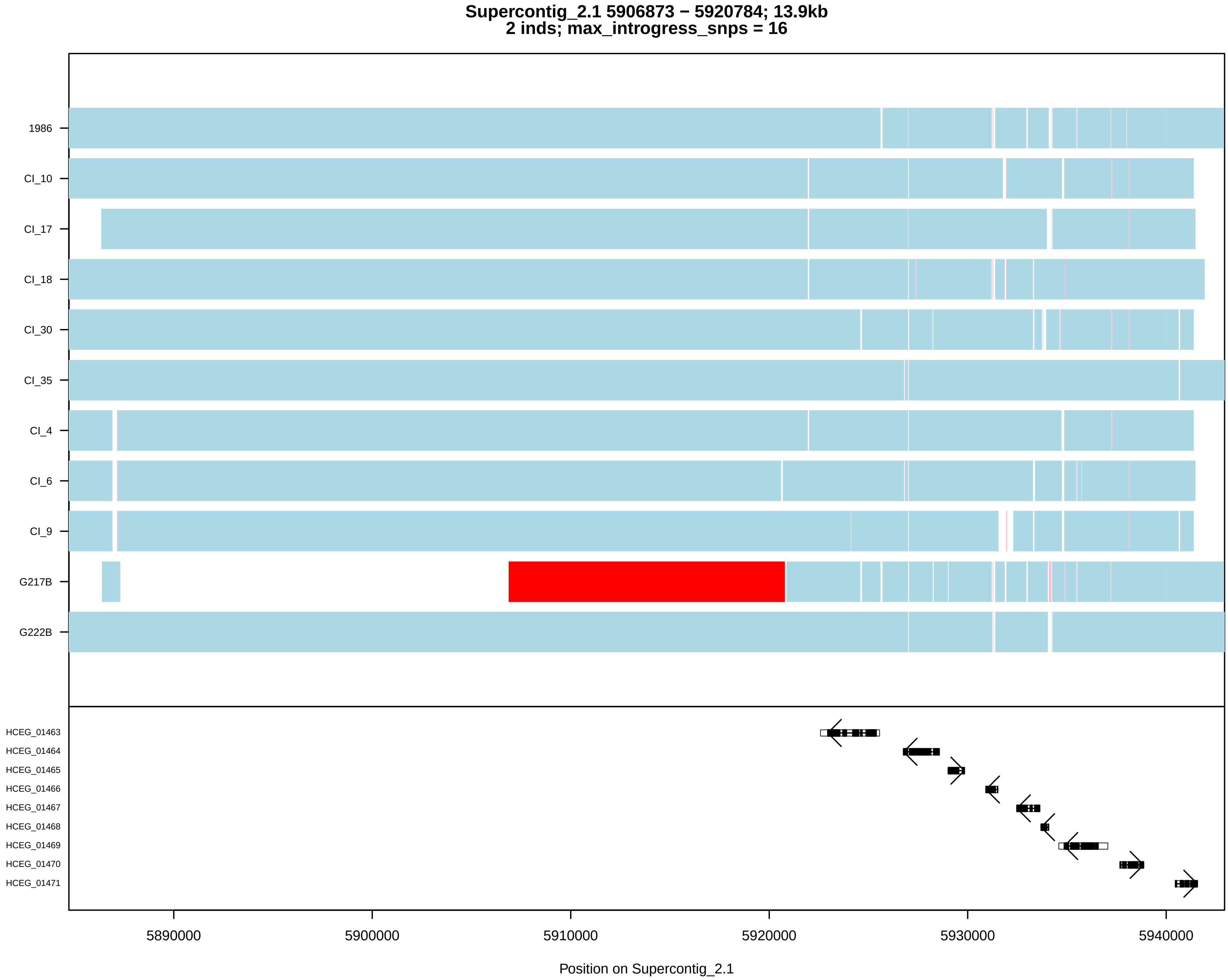
Supercontig_2.1 5906873 − 5920784; 13.9kb
2 inds; max_introgress_snps = 16
1986
CI_10
CI_17
CI_18
CI_30
CI_35
CI_4
CI_6
CI_9
G217B
G222B
HCEG_01463
HCEG_01464
HCEG_01465
HCEG_01466
HCEG_01467
HCEG_01468
HCEG_01469
HCEG_01470
HCEG_01471
5890000
5900000
5910000
5920000
5930000
5940000
Position on Supercontig_2.1

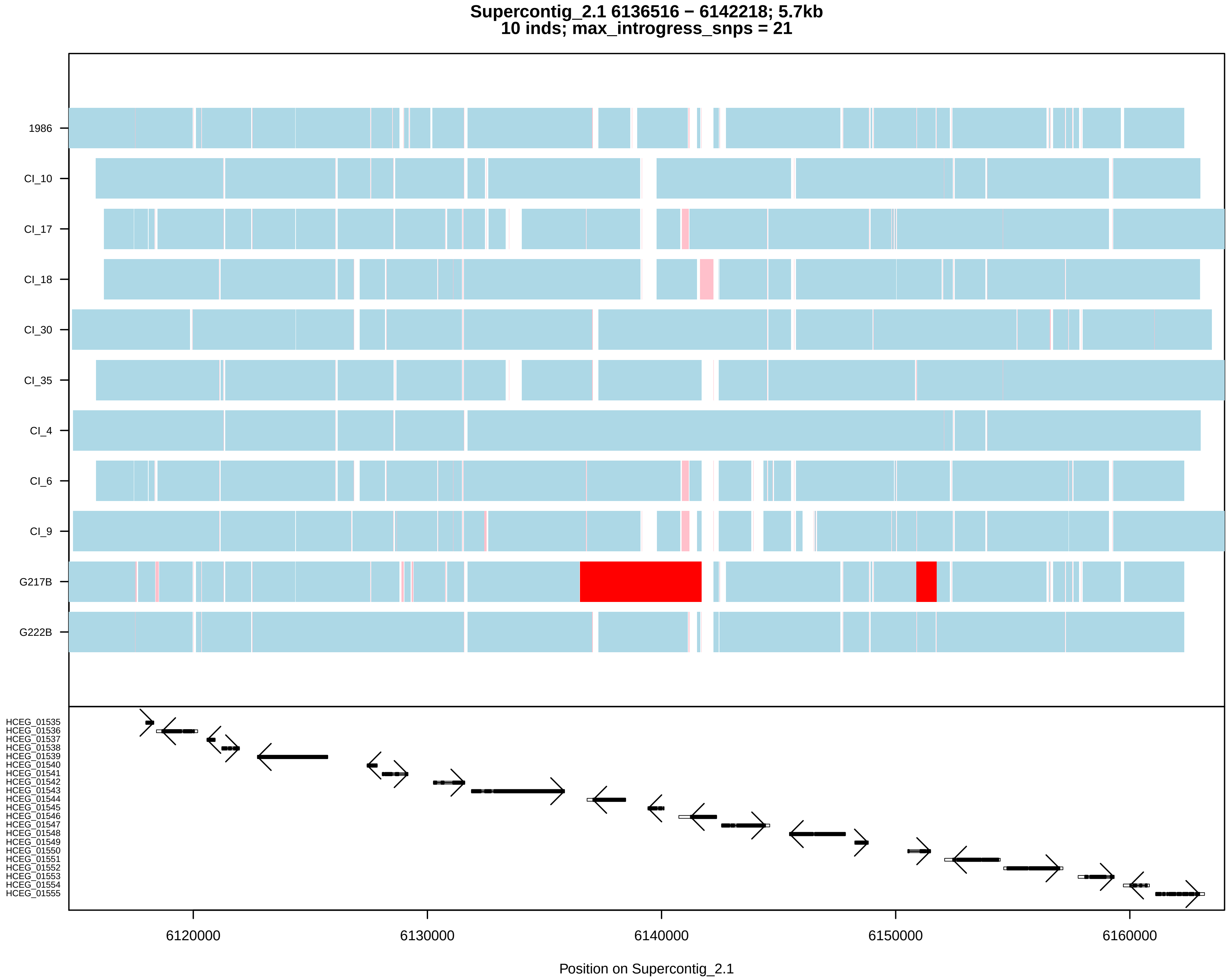
Supercontig_2.1 6136516 − 6142218; 5.7kb
10 inds; max_introgress_snps = 21
1986
CI_10
CI_17
CI_18
CI_30
CI_35
CI_4
CI_6
CI_9
G217B
G222B
HCEG_01535
HCEG_01536
HCEG_01537
HCEG_01538
HCEG_01539
HCEG_01540
HCEG_01541
HCEG_01542
HCEG_01543
HCEG_01544
HCEG_01545
HCEG_01546
HCEG_01547
HCEG_01548
HCEG_01549
HCEG_01550
HCEG_01551
HCEG_01552
HCEG_01553
HCEG_01554
HCEG_01555
6120000
6130000
6140000
6150000
6160000
Position on Supercontig_2.1

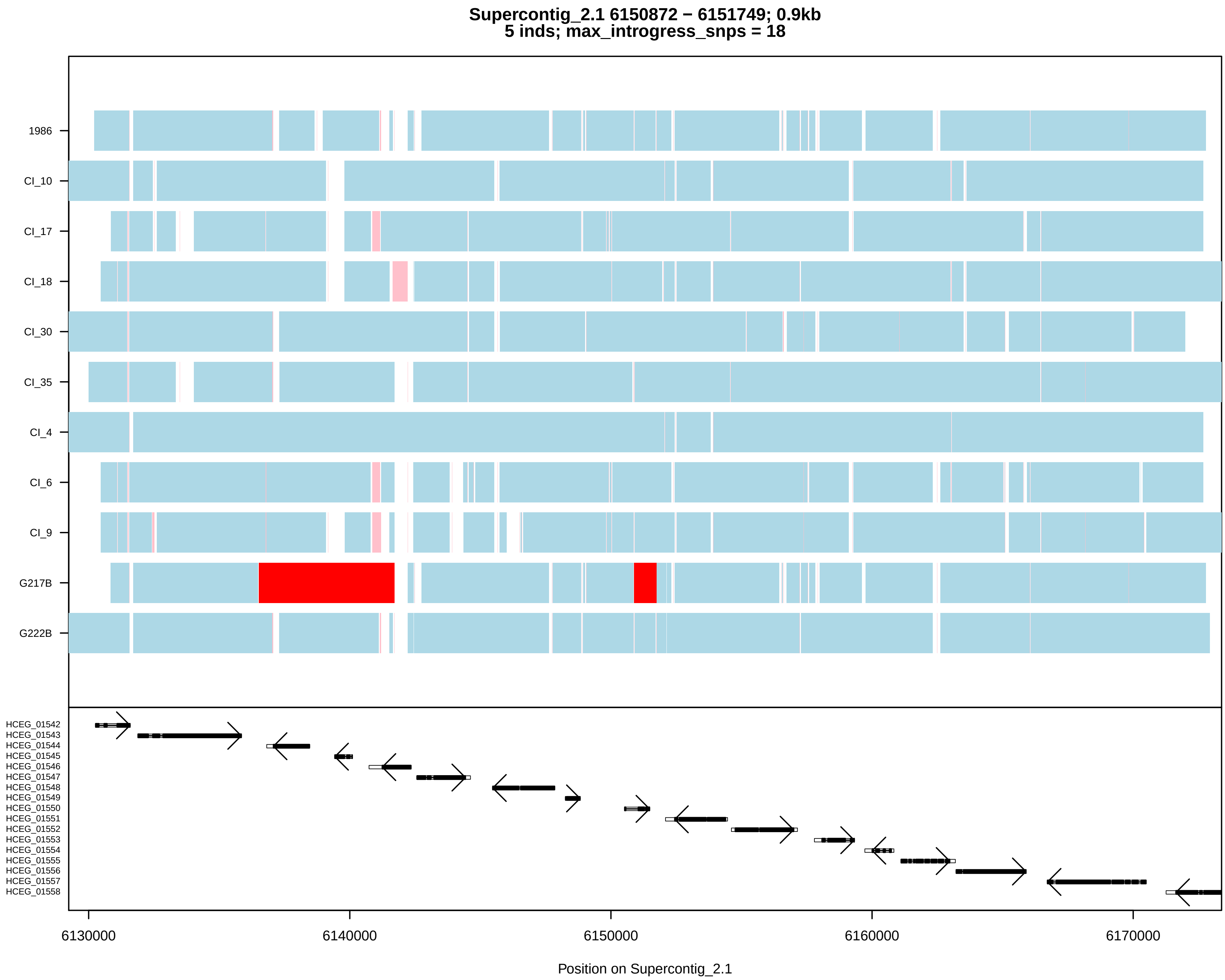
Supercontig_2.1 6150872 − 6151749; 0.9kb
5 inds; max_introgress_snps = 18
1986
CI_10
CI_17
CI_18
CI_30
CI_35
CI_4
CI_6
CI_9
G217B
G222B
HCEG_01542
HCEG_01543
HCEG_01544
HCEG_01545
HCEG_01546
HCEG_01547
HCEG_01548
HCEG_01549
HCEG_01550
HCEG_01551
HCEG_01552
HCEG_01553
HCEG_01554
HCEG_01555
HCEG_01556
HCEG_01557
HCEG_01558
6130000
6140000
6150000
6160000
6170000
Position on Supercontig_2.1

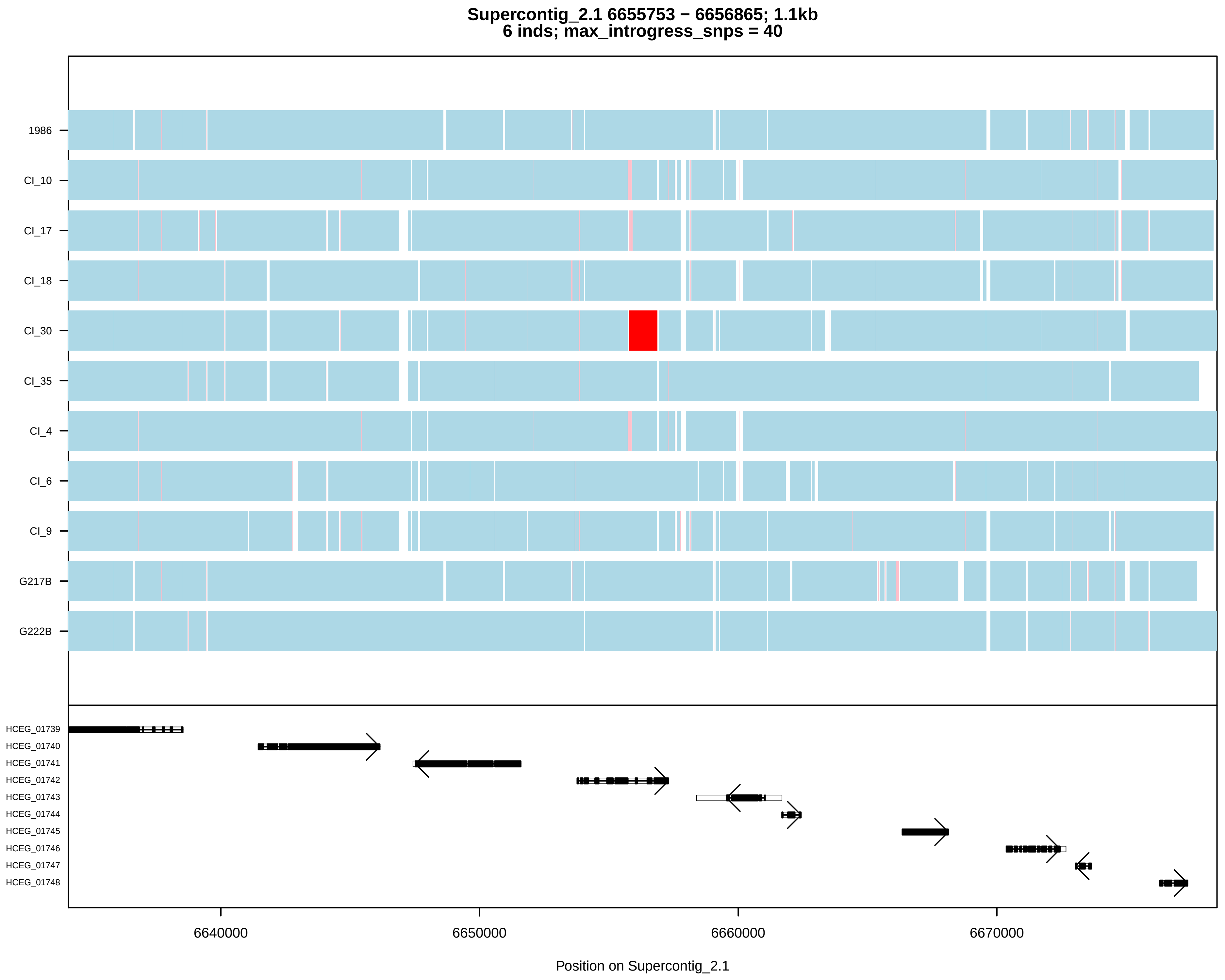
Supercontig_2.1 6655753 − 6656865; 1.1kb
6 inds; max_introgress_snps = 40
1986
CI_10
CI_17
CI_18
CI_30
CI_35
CI_4
CI_6
CI_9
G217B
G222B
HCEG_01739
HCEG_01740
HCEG_01741
HCEG_01742
HCEG_01743
HCEG_01744
HCEG_01745
HCEG_01746
HCEG_01747
HCEG_01748
6640000
6650000
6660000
6670000
Position on Supercontig_2.1

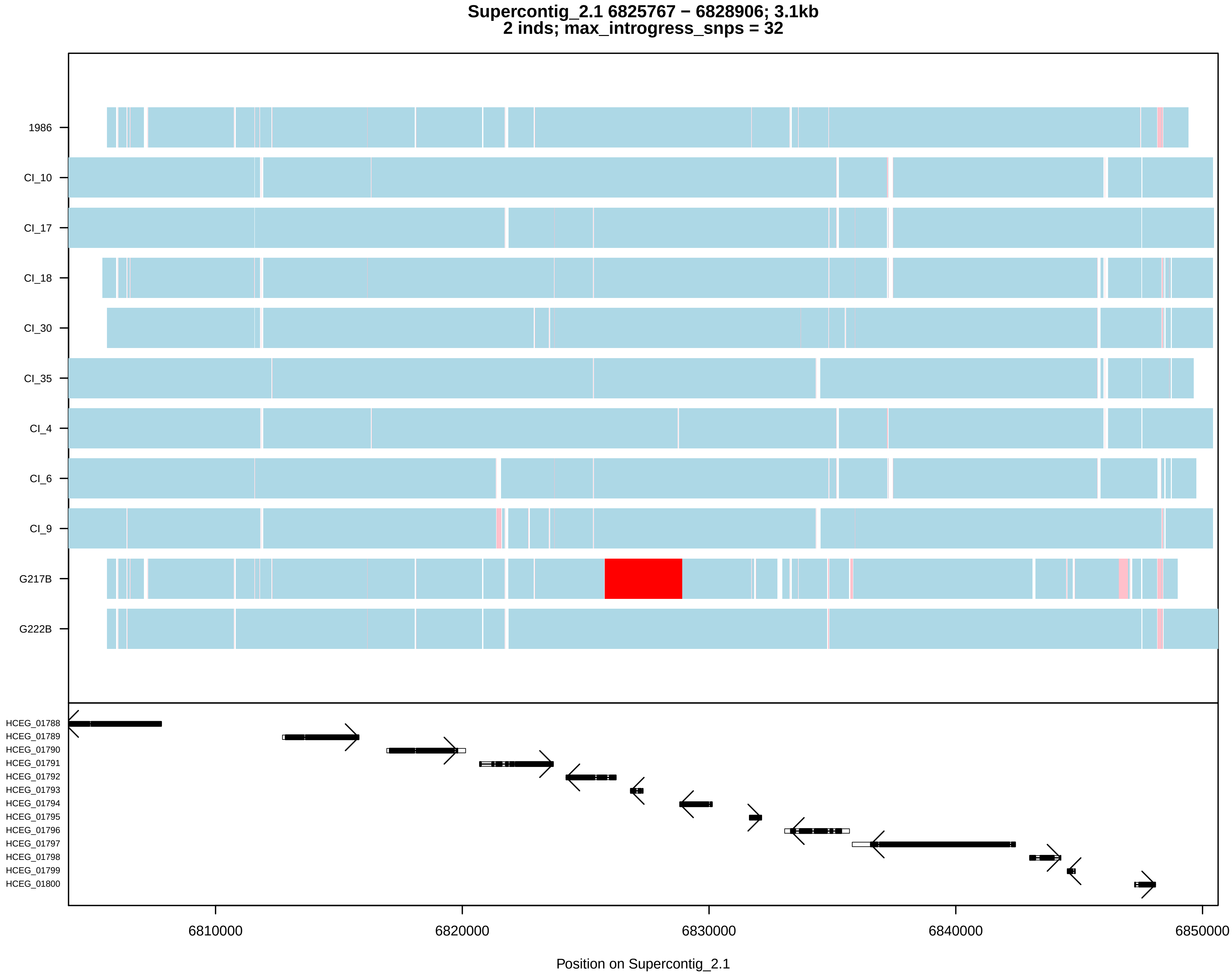

Supercontig_2.1 6825767 − 6828906; 3.1kb
2 inds; max_introgress_snps = 32
1986
CI_10
CI_17
CI_18
CI_30
CI_35
CI_4
CI_6
CI_9
G217B
G222B
HCEG_01788
HCEG_01789
HCEG_01790
HCEG_01791
HCEG_01792
HCEG_01793
HCEG_01794
HCEG_01795
HCEG_01796
HCEG_01797
HCEG_01798
HCEG_01799
HCEG_01800
6810000
6820000
6830000
6840000
6850000
Position on Supercontig_2.1

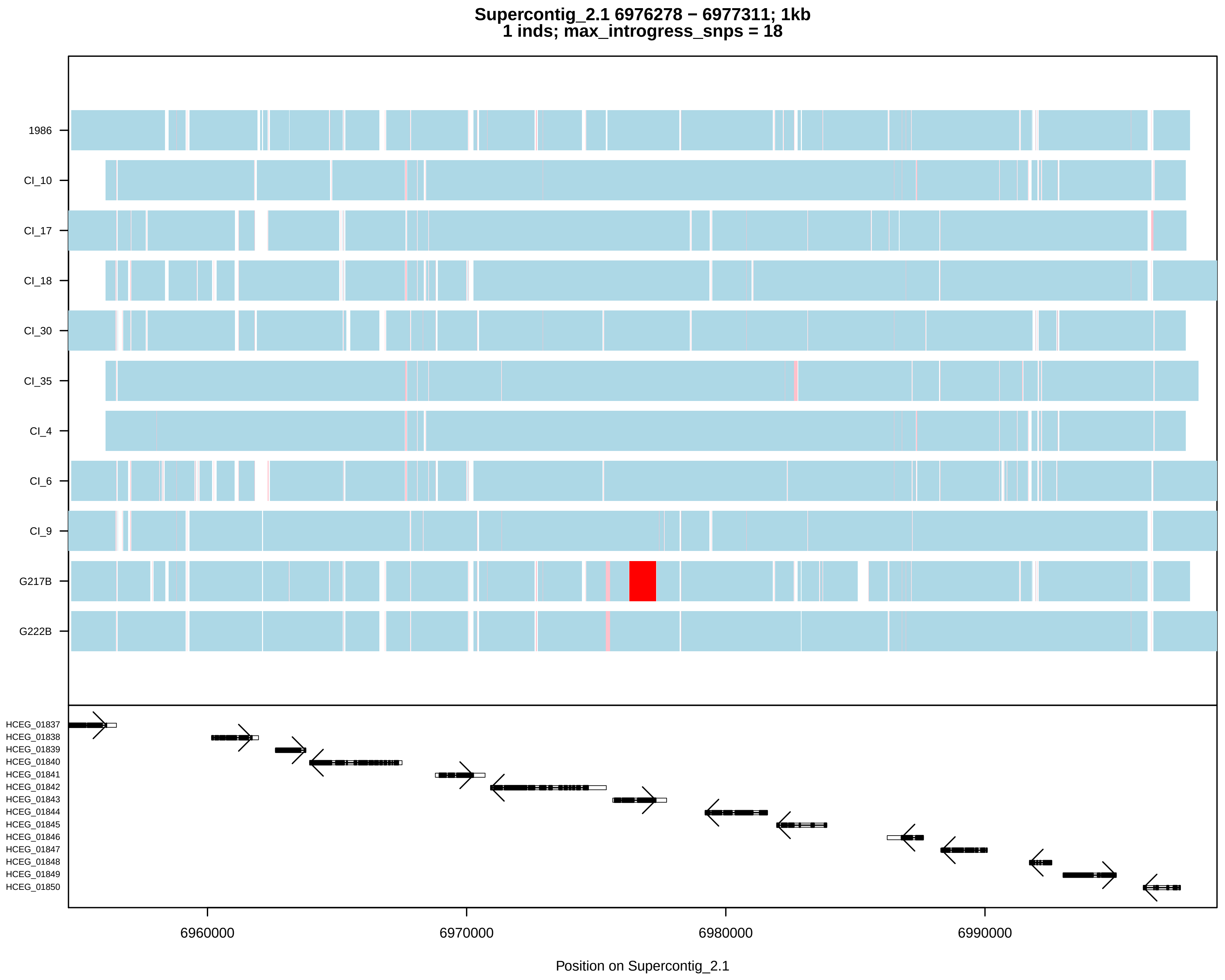
Supercontig_2.1 6976278 − 6977311; 1kb
1 inds; max_introgress_snps = 18
1986
CI_10
CI_17
CI_18
CI_30
CI_35
CI_4
CI_6
CI_9
G217B
G222B
HCEG_01837
HCEG_01838
HCEG_01839
HCEG_01840
HCEG_01841
HCEG_01842
HCEG_01843
HCEG_01844
HCEG_01845
HCEG_01846
HCEG_01847
HCEG_01848
HCEG_01849
HCEG_01850
6960000
6970000
6980000
6990000
Position on Supercontig_2.1

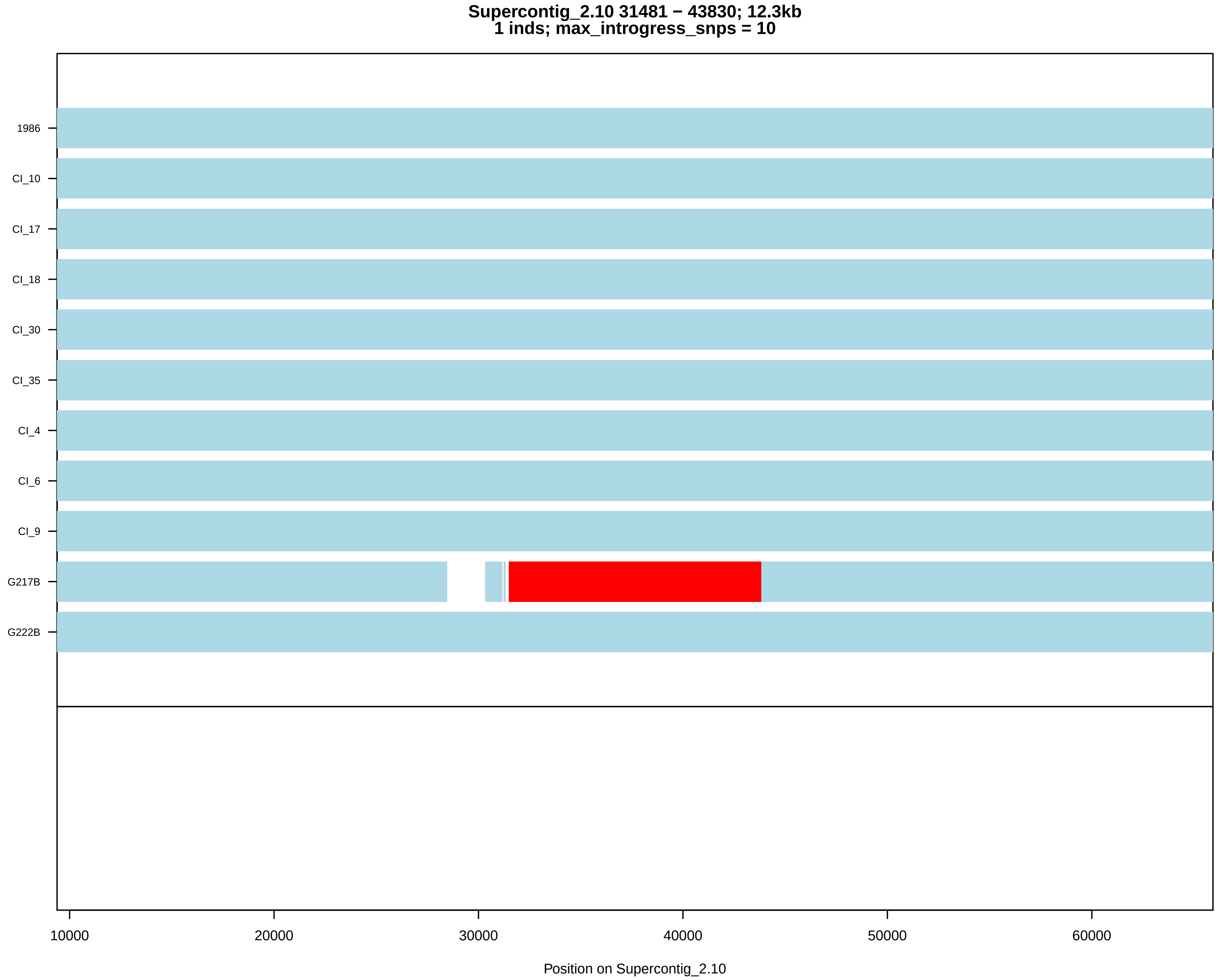
Supercontig_2.10 31481 − 43830; 12.3kb
1 inds; max_introgress_snps = 10
1986
CI_10
CI_17
CI_18
CI_30
CI_35
CI_4
CI_6
CI_9
G217B
G222B
10000
20000
30000
40000
50000
60000
Position on Supercontig_2.10

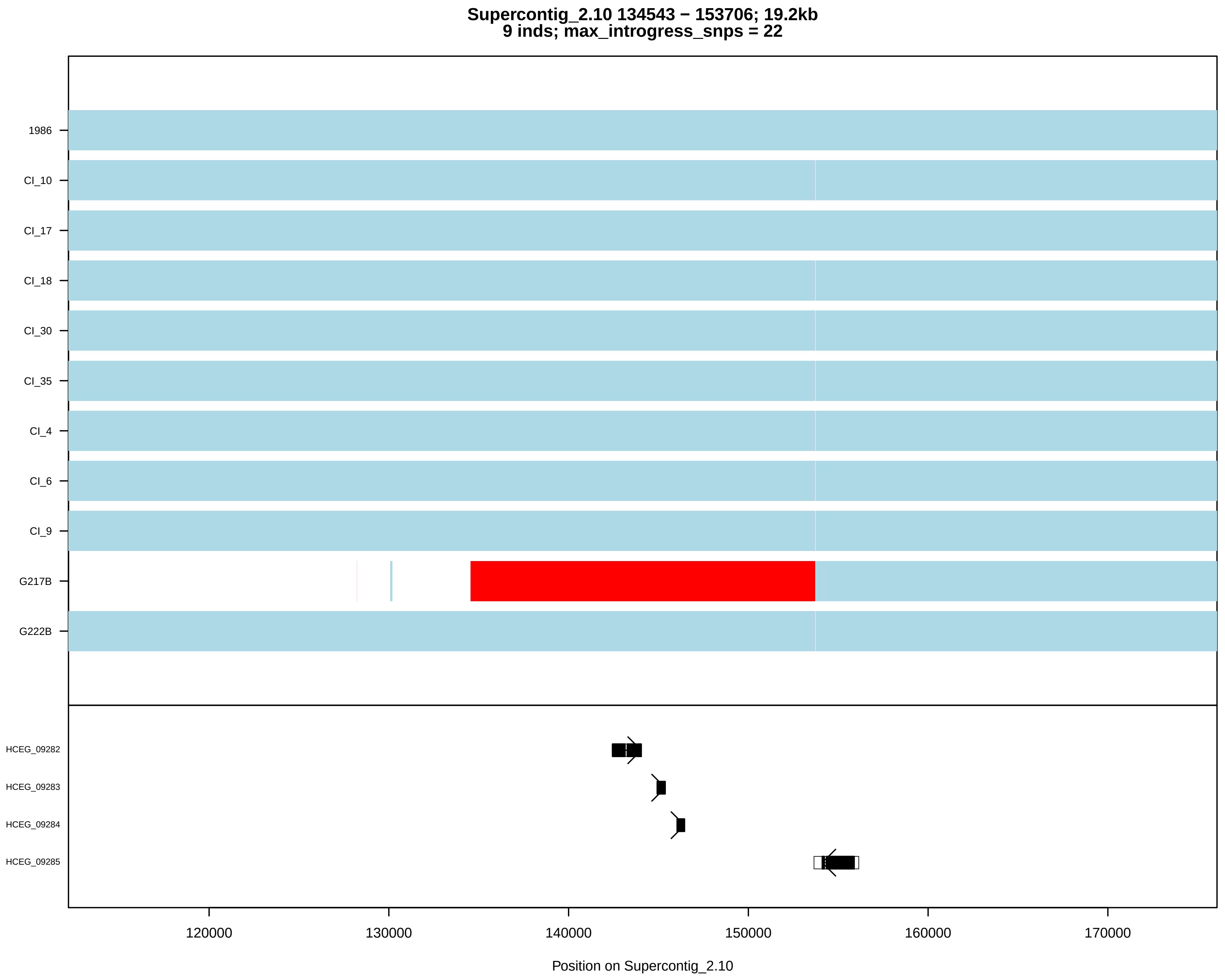
Supercontig_2.10 134543 − 153706; 19.2kb
9 inds; max_introgress_snps = 22
1986
CI_10
CI_17
CI_18
CI_30
CI_35
CI_4
CI_6
CI_9
G217B
G222B
HCEG_09282
HCEG_09283
HCEG_09284
HCEG_09285
120000
130000
140000
150000
160000
170000
Position on Supercontig_2.10

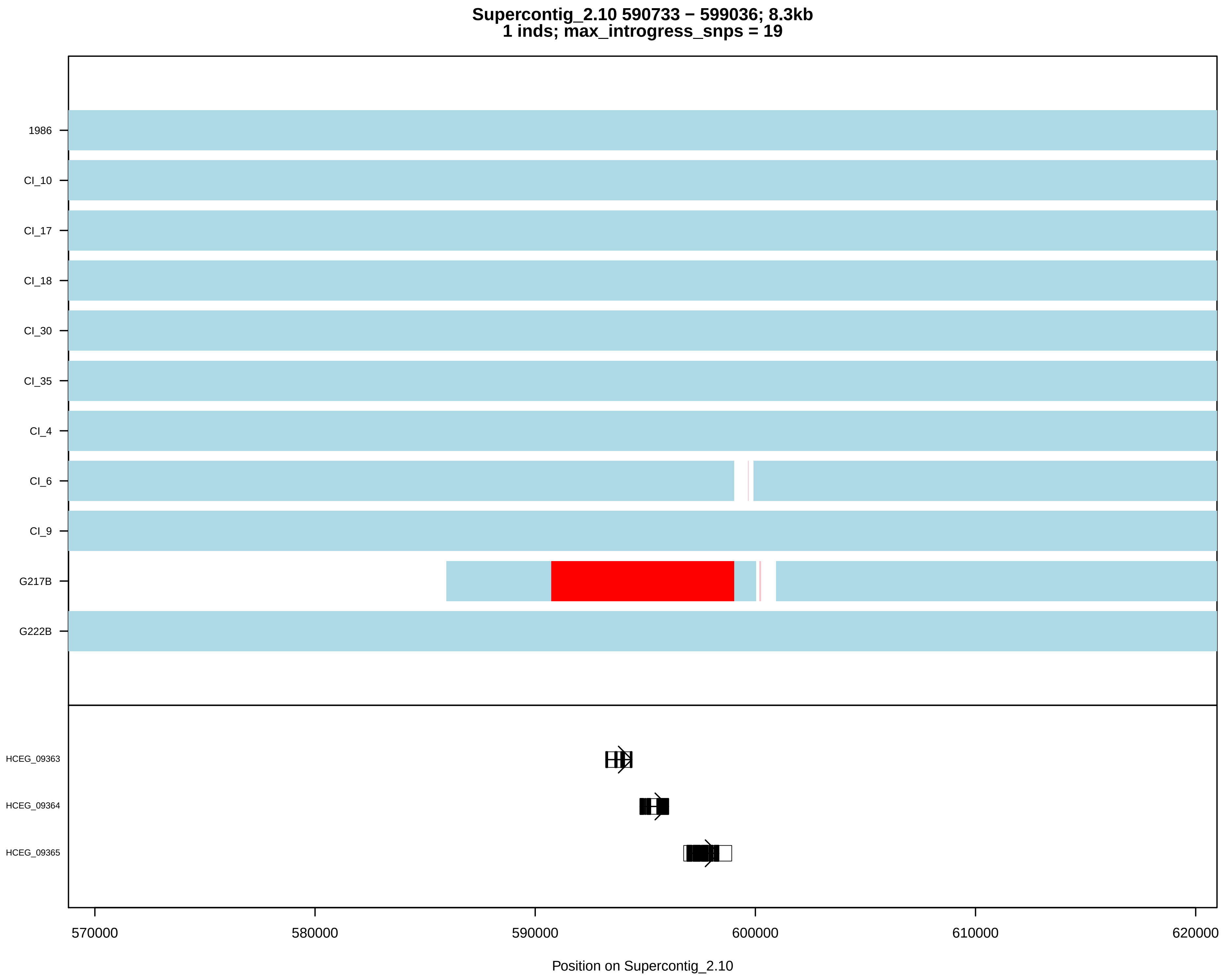
Supercontig_2.10 590733 − 599036; 8.3kb
1 inds; max_introgress_snps = 19
1986
CI_10
CI_17
CI_18
CI_30
CI_35
CI_4
CI_6
CI_9
G217B
G222B
HCEG_09363
HCEG_09364
HCEG_09365
570000
580000
590000
600000
610000
620000
Position on Supercontig_2.10

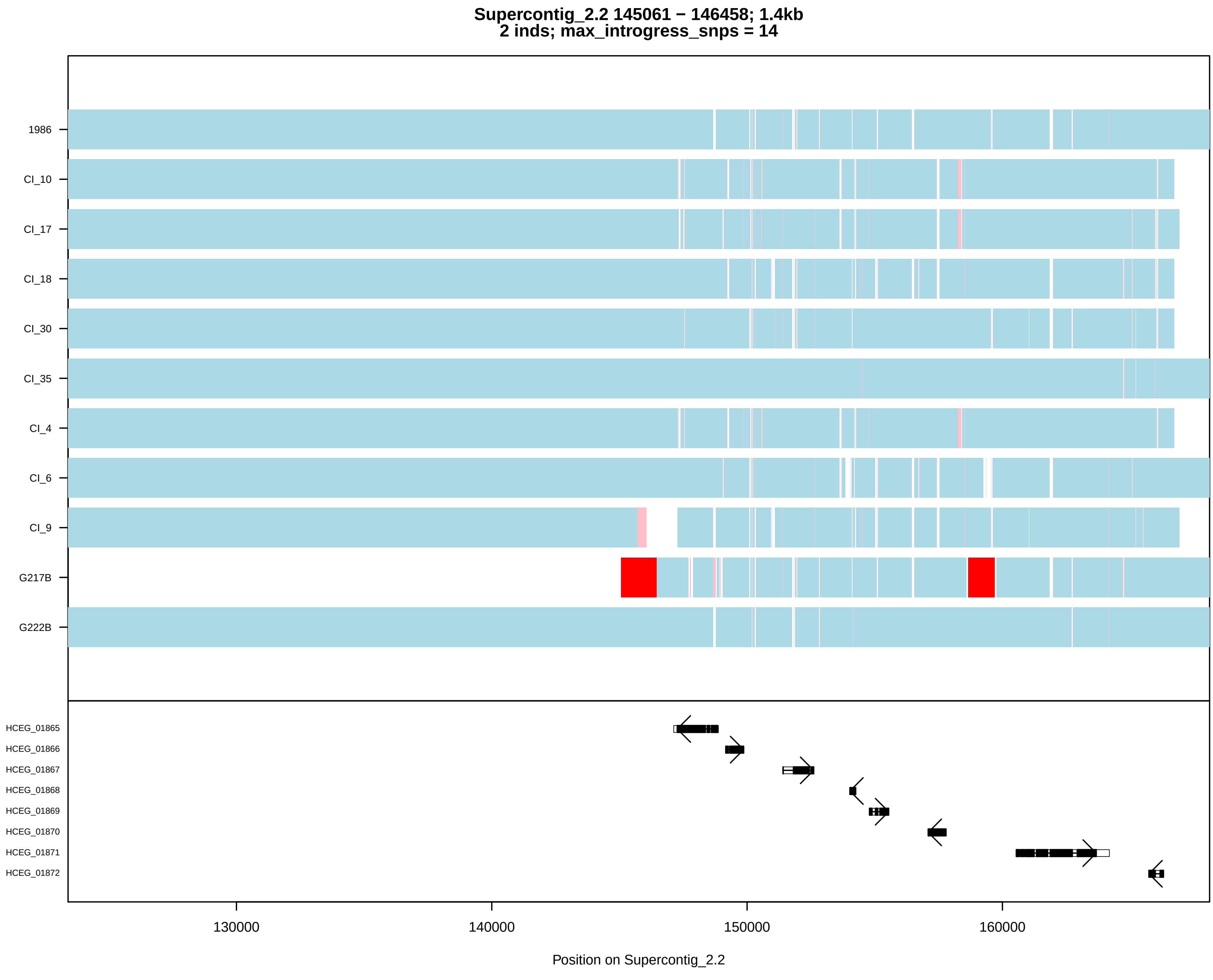
Supercontig_2.2 145061 − 146458; 1.4kb
2 inds; max_introgress_snps = 14
1986
CI_10
CI_17
CI_18
CI_30
CI_35
CI_4
CI_6
CI_9
G217B
G222B
HCEG_01865
HCEG_01866
HCEG_01867
HCEG_01868
HCEG_01869
HCEG_01870
HCEG_01871
HCEG_01872
130000
140000
150000
160000
Position on Supercontig_2.2

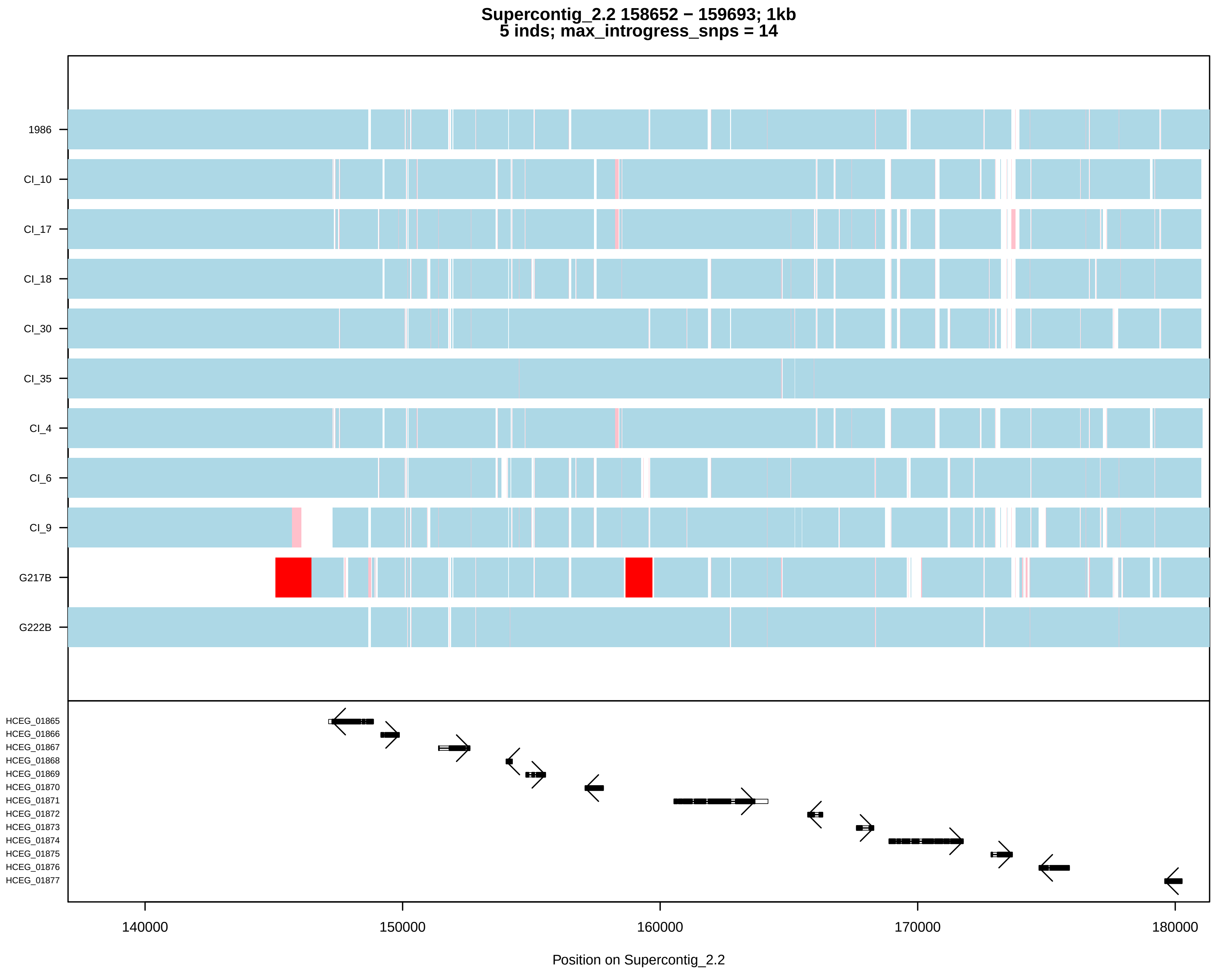

Supercontig_2.2 158652 − 159693; 1kb
5 inds; max_introgress_snps = 14
1986
CI_10
CI_17
CI_18
CI_30
CI_35
CI_4
CI_6
CI_9
G217B
G222B
HCEG_01865
HCEG_01866
HCEG_01867
HCEG_01868
HCEG_01869
HCEG_01870
HCEG_01871
HCEG_01872
HCEG_01873
HCEG_01874
HCEG_01875
HCEG_01876
HCEG_01877
140000
150000
160000
170000
180000
Position on Supercontig_2.2

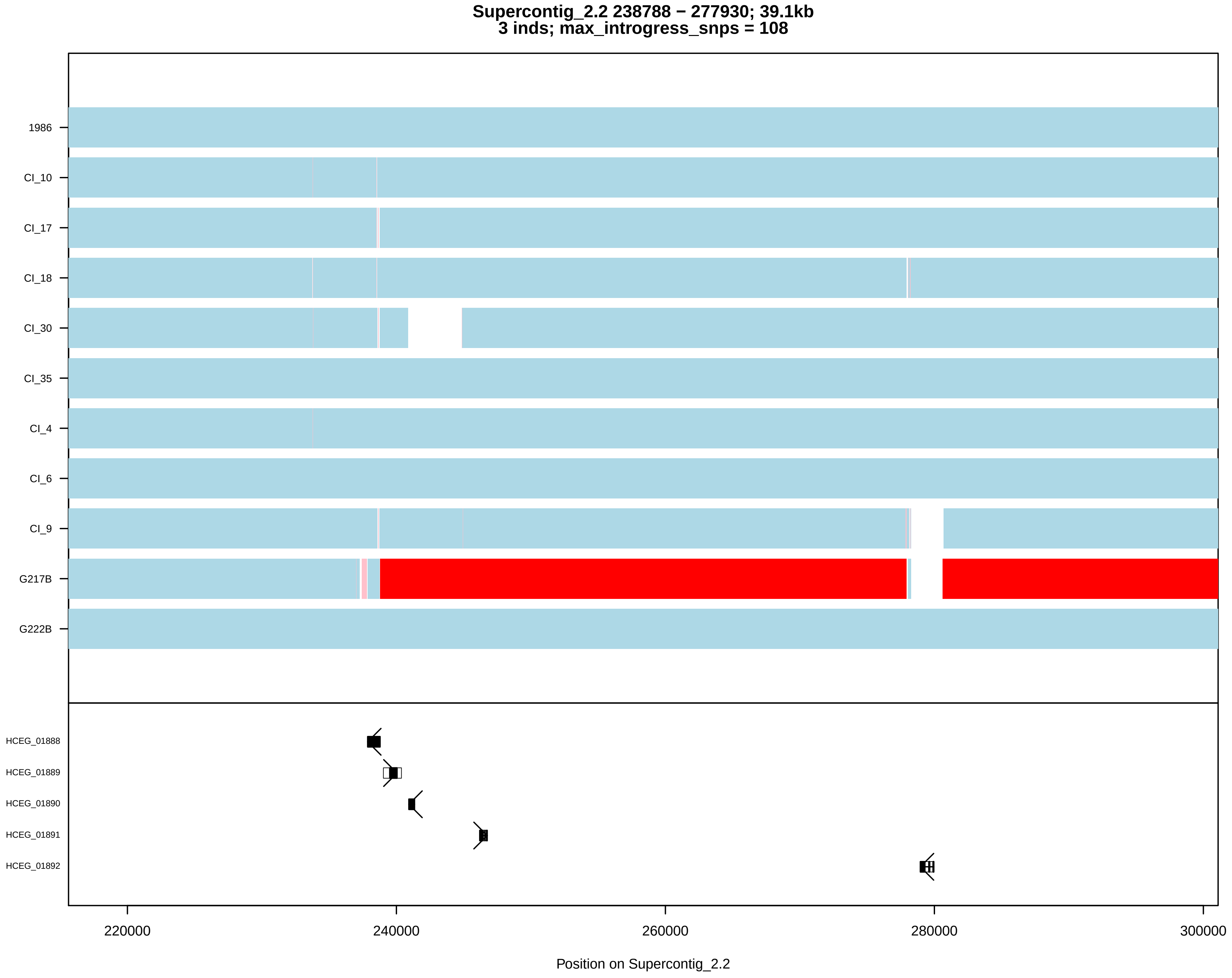

Supercontig_2.2 238788 − 277930; 39.1kb
3 inds; max_introgress_snps = 108
1986
CI_10
CI_17
CI_18
CI_30
CI_35
CI_4
CI_6
CI_9
G217B
G222B
HCEG_01888
HCEG_01889
HCEG_01890
HCEG_01891
HCEG_01892
220000
240000
260000
280000
300000
Position on Supercontig_2.2

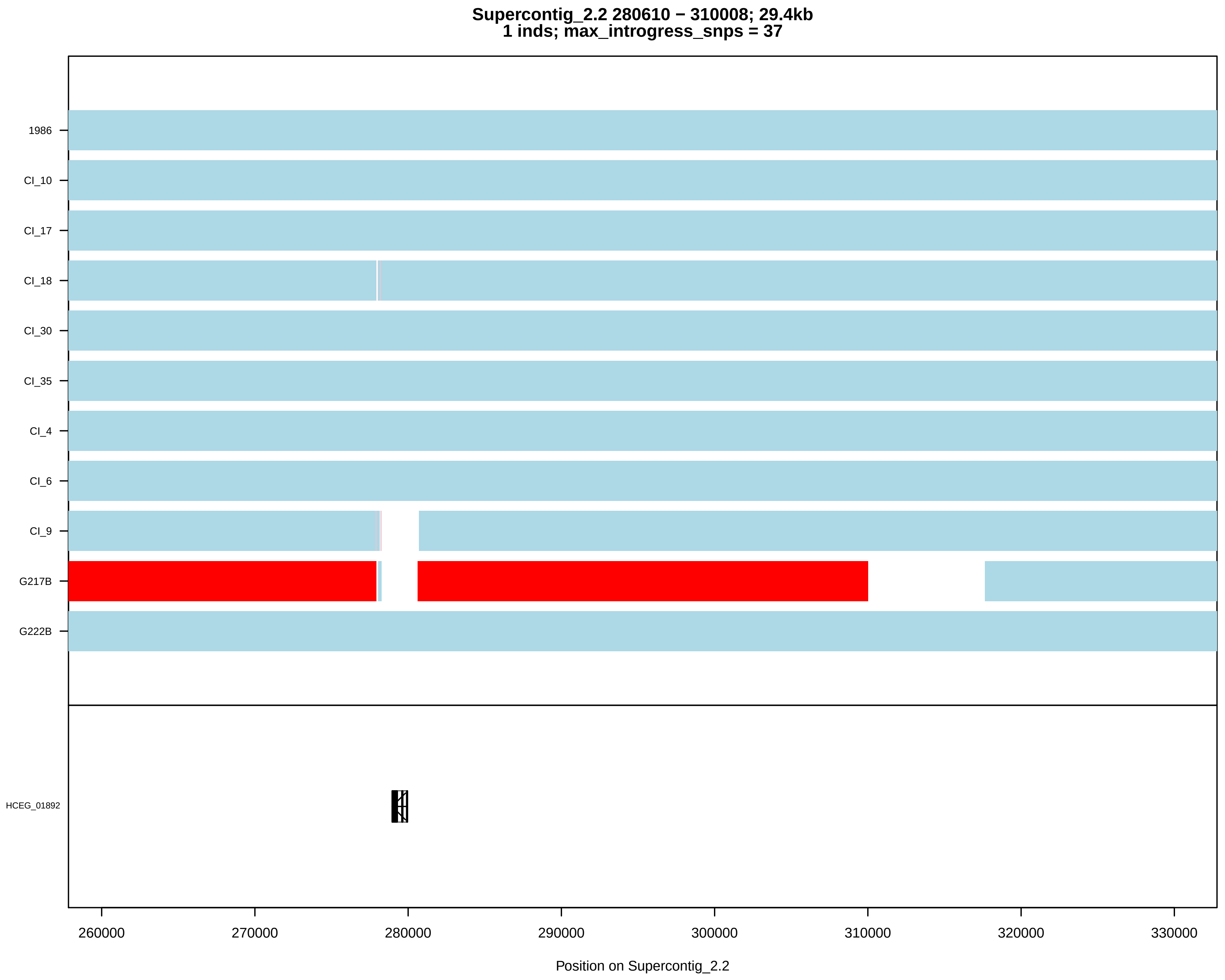

Supercontig_2.2 280610 − 310008; 29.4kb
1 inds; max_introgress_snps = 37
1986
CI_10
CI_17
CI_18
CI_30
CI_35
CI_4
CI_6
CI_9
G217B
G222B
HCEG_01892
260000
270000
280000
290000
300000
310000
320000
330000
Position on Supercontig_2.2

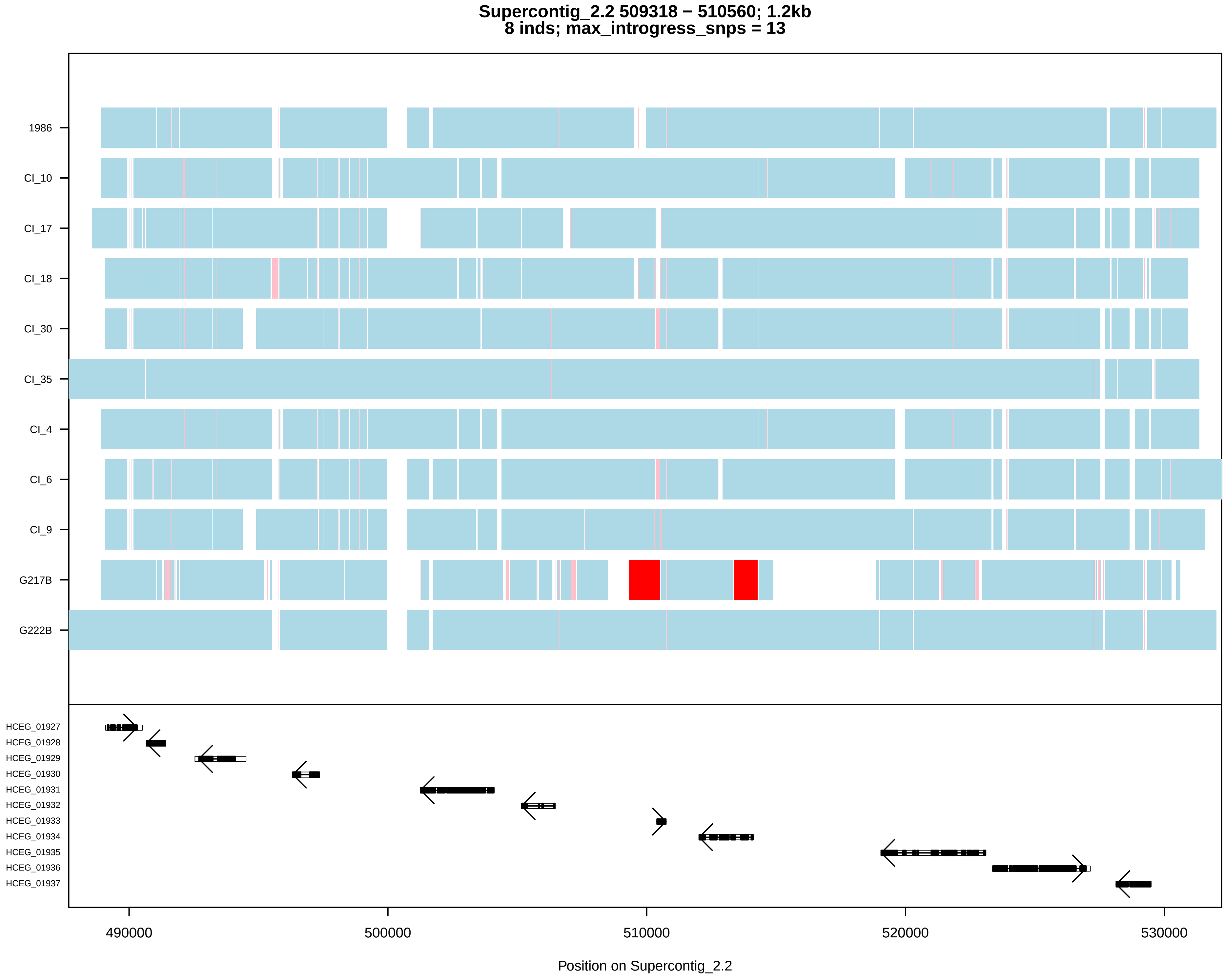

Supercontig_2.2 509318 − 510560; 1.2kb
8 inds; max_introgress_snps = 13
1986
CI_10
CI_17
CI_18
CI_30
CI_35
CI_4
CI_6
CI_9
G217B
G222B
HCEG_01927
HCEG_01928
HCEG_01929
HCEG_01930
HCEG_01931
HCEG_01932
HCEG_01933
HCEG_01934
HCEG_01935
HCEG_01936
HCEG_01937
490000
500000
510000
520000
530000
Position on Supercontig_2.2

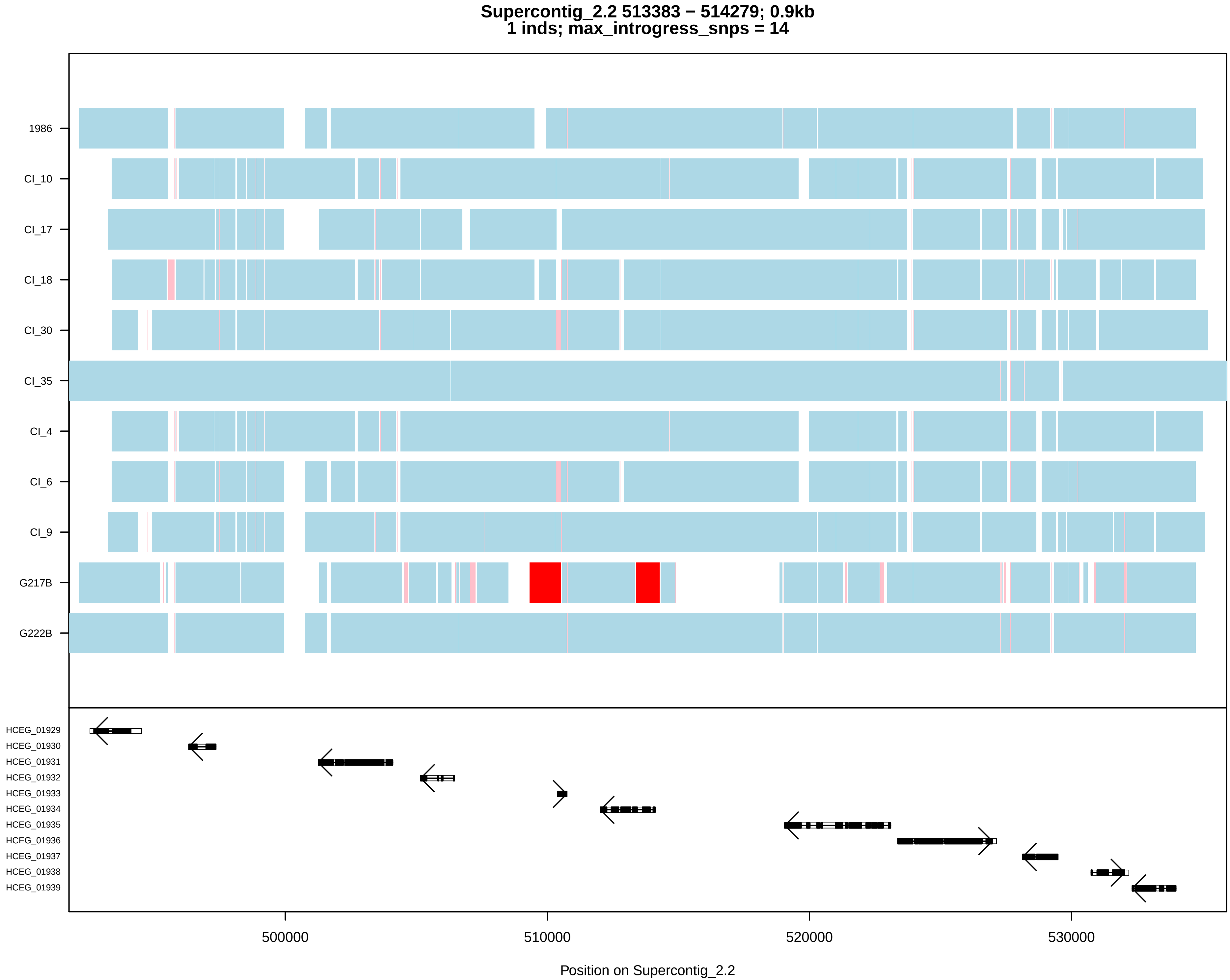

Supercontig_2.2 513383 − 514279; 0.9kb
1 inds; max_introgress_snps = 14
1986
CI_10
CI_17
CI_18
CI_30
CI_35
CI_4
CI_6
CI_9
G217B
G222B
HCEG_01929
HCEG_01930
HCEG_01931
HCEG_01932
HCEG_01933
HCEG_01934
HCEG_01935
HCEG_01936
HCEG_01937
HCEG_01938
HCEG_01939
500000
510000
520000
530000
Position on Supercontig_2.2

Supercontig_2.2 743913 − 745262; 1.4kb
1 inds; max_introgress_snps = 50

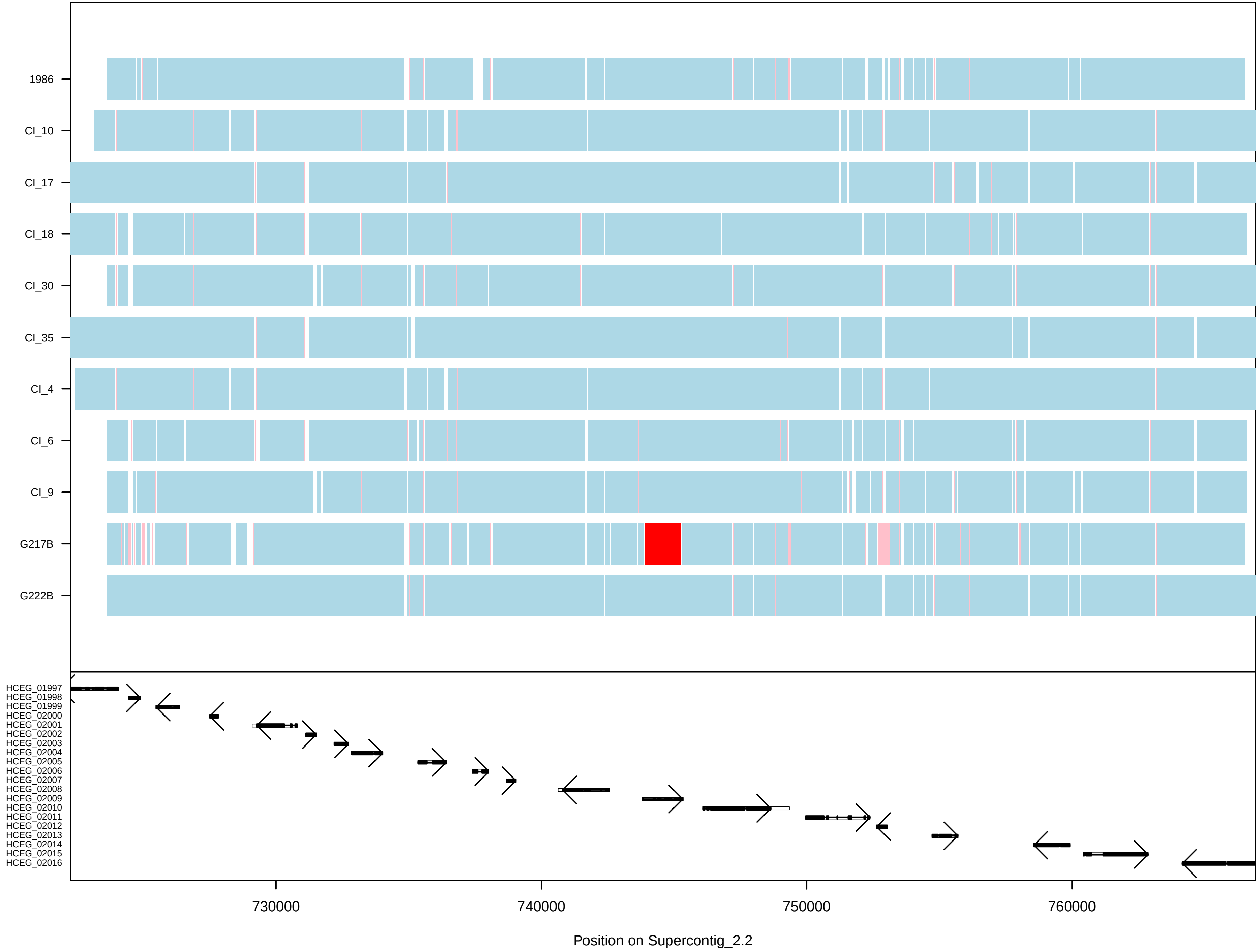

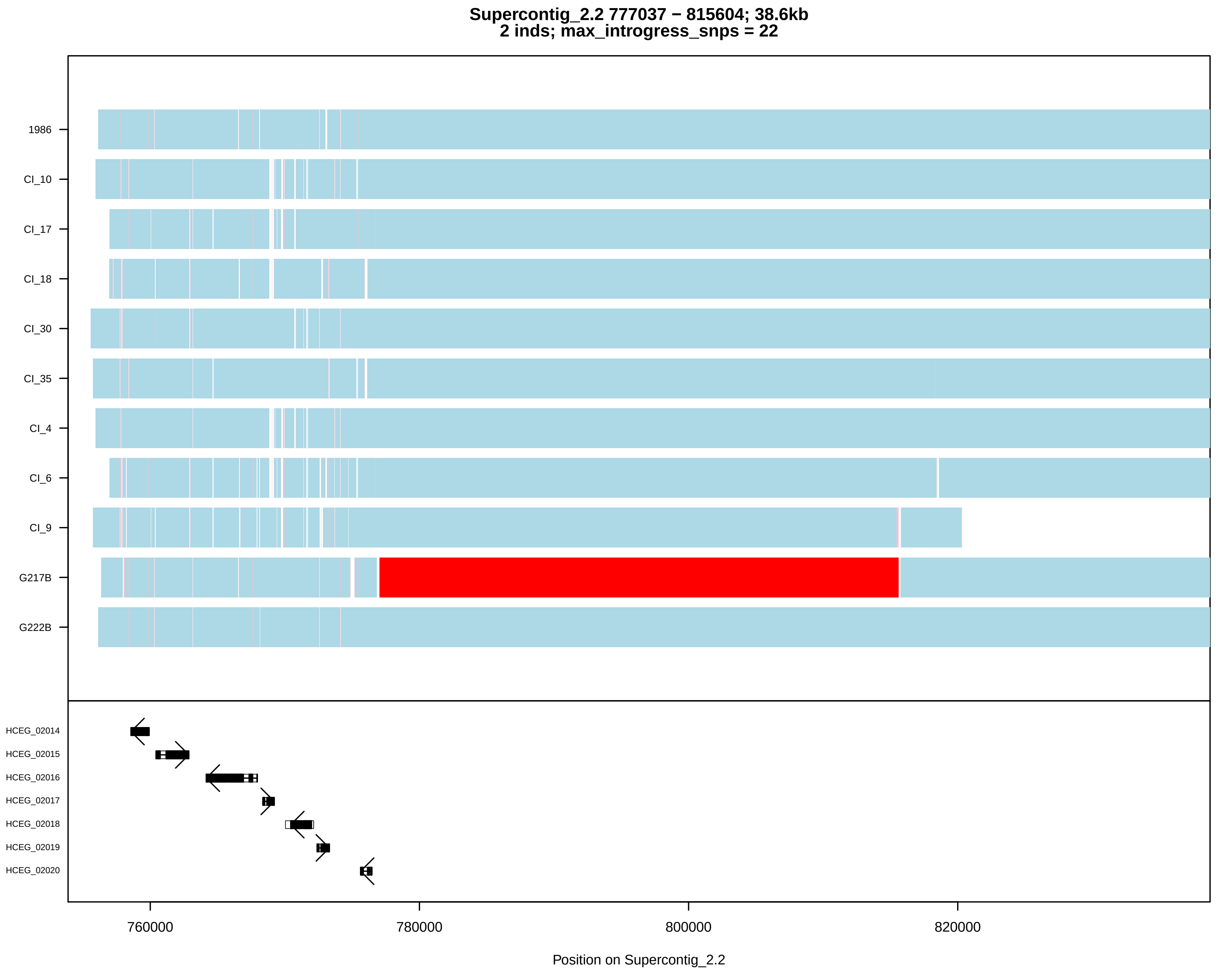
Supercontig_2.2 777037 − 815604; 38.6kb
2 inds; max_introgress_snps = 22
1986
CI_10
CI_17
CI_18
CI_30
CI_35
CI_4
CI_6
CI_9
G217B
G222B
HCEG_02014
HCEG_02015
HCEG_02016
HCEG_02017
HCEG_02018
HCEG_02019
HCEG_02020
760000
780000
800000
820000
Position on Supercontig_2.2

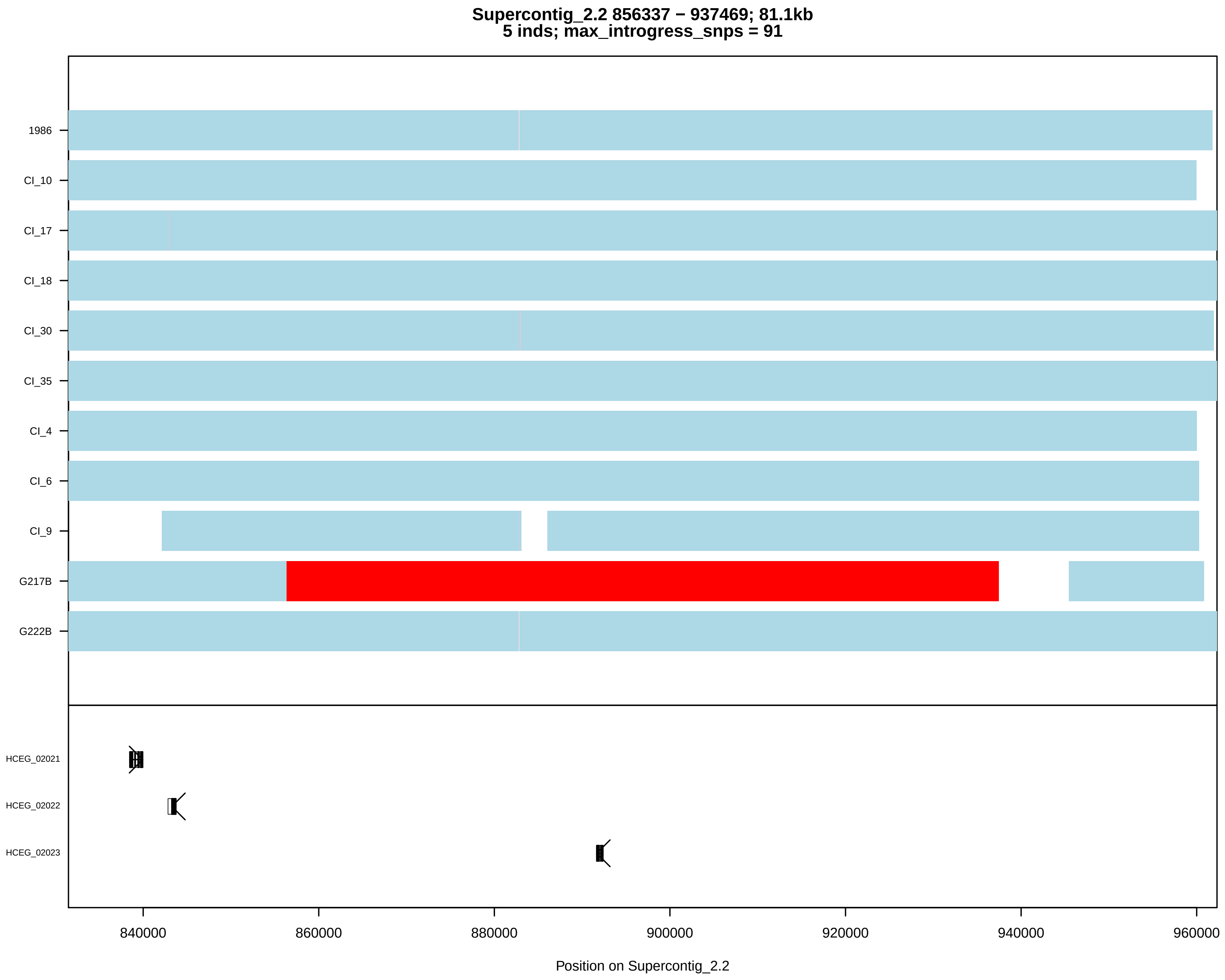

Supercontig_2.2 856337 − 937469; 81.1kb
5 inds; max_introgress_snps = 91
1986
CI_10
CI_17
CI_18
CI_30
CI_35
CI_4
CI_6
CI_9
G217B
G222B
HCEG_02021
HCEG_02022
HCEG_02023
840000
860000
880000
900000
920000
940000
960000
Position on Supercontig_2.2

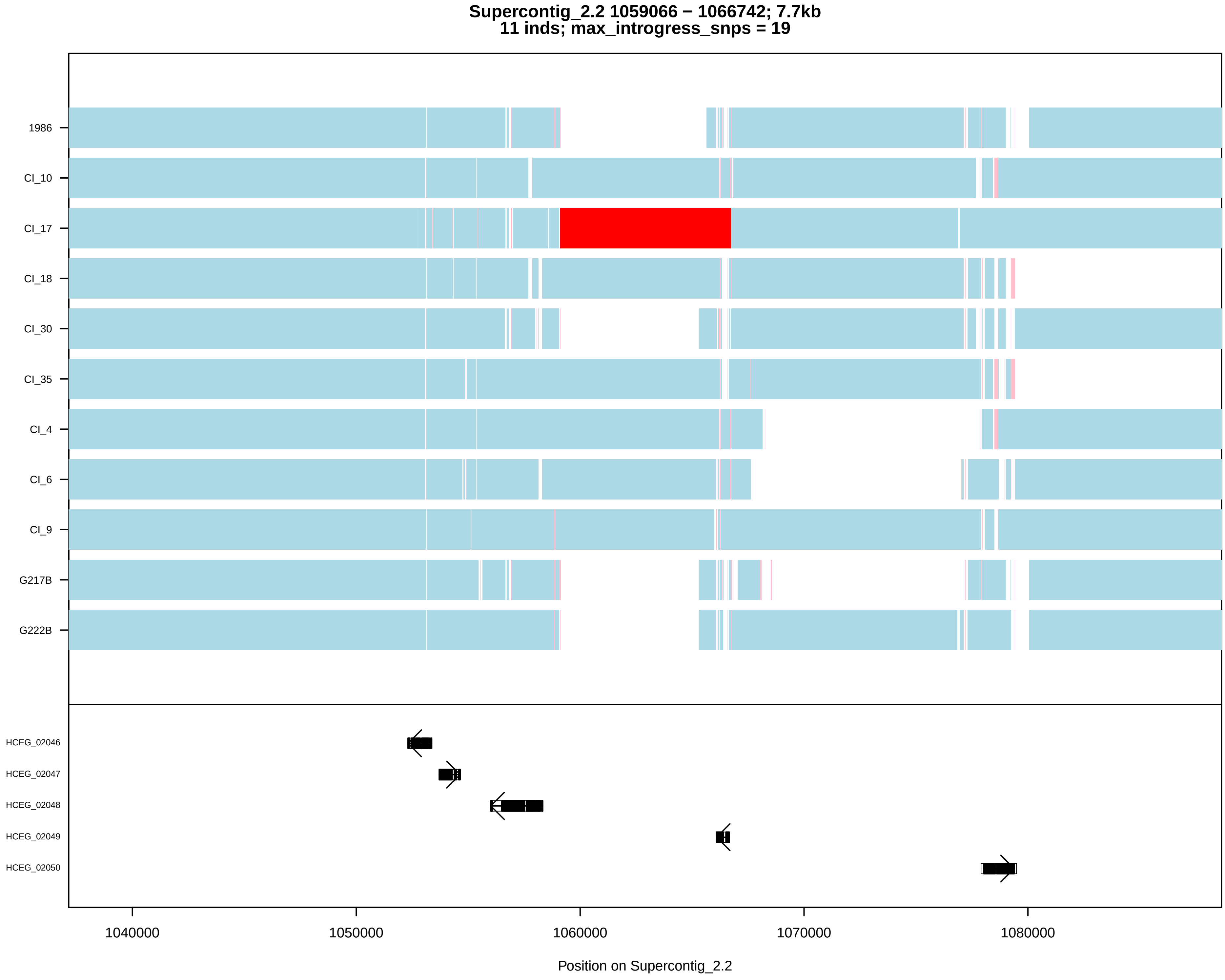

Supercontig_2.2 1059066 − 1066742; 7.7kb
11 inds; max_introgress_snps = 19
1986
CI_10
CI_17
CI_18
CI_30
CI_35
CI_4
CI_6
CI_9
G217B
G222B
HCEG_02046
HCEG_02047
HCEG_02048
HCEG_02049
HCEG_02050
1040000
1050000
1060000
1070000
1080000
Position on Supercontig_2.2

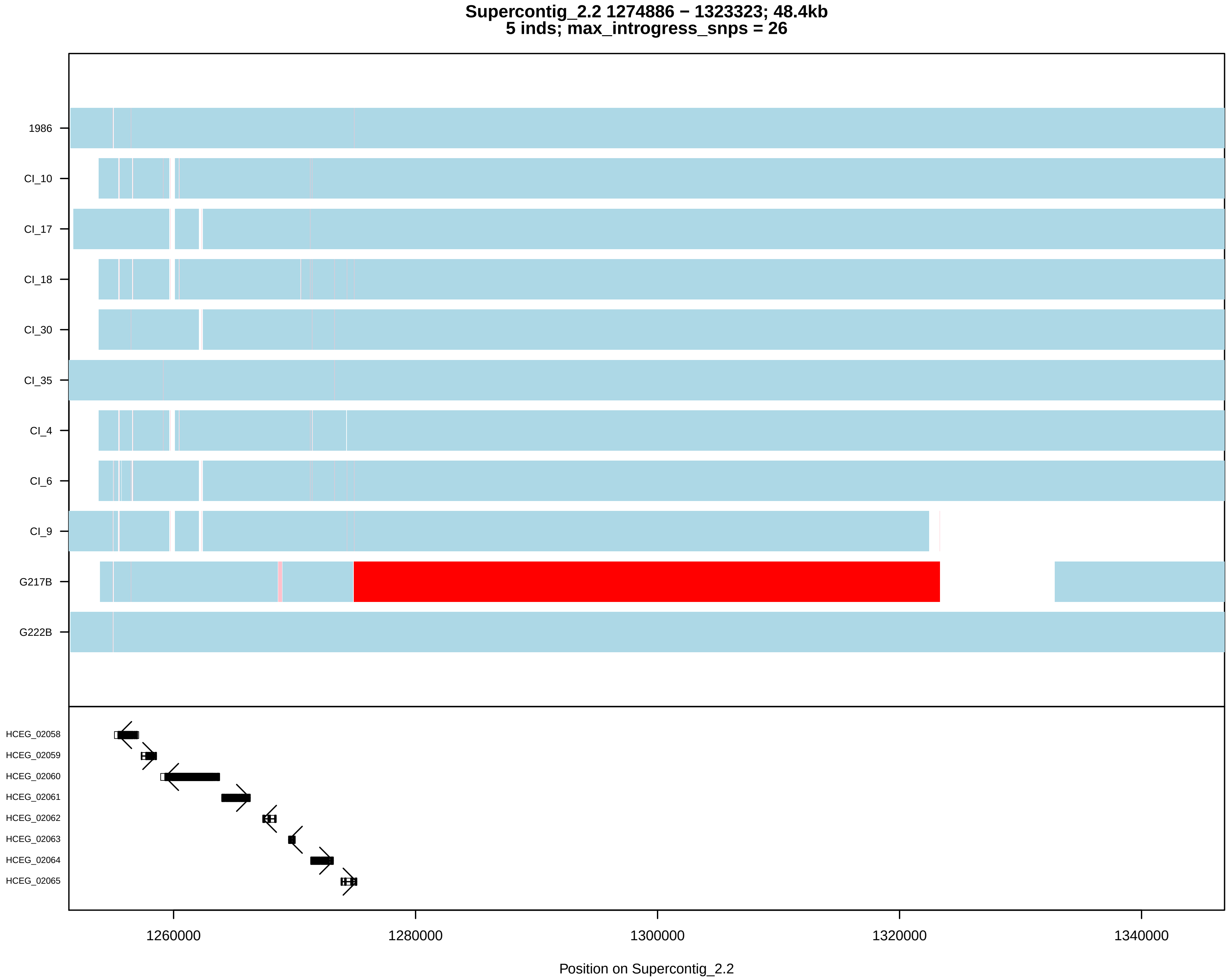
Supercontig_2.2 1274886 − 1323323; 48.4kb
5 inds; max_introgress_snps = 26
1986
CI_10
CI_17
CI_18
CI_30
CI_35
CI_4
CI_6
CI_9
G217B
G222B
HCEG_02058
HCEG_02059
HCEG_02060
HCEG_02061
HCEG_02062
HCEG_02063
HCEG_02064
HCEG_02065
1260000
1280000
1300000
1320000
1340000
Position on Supercontig_2.2

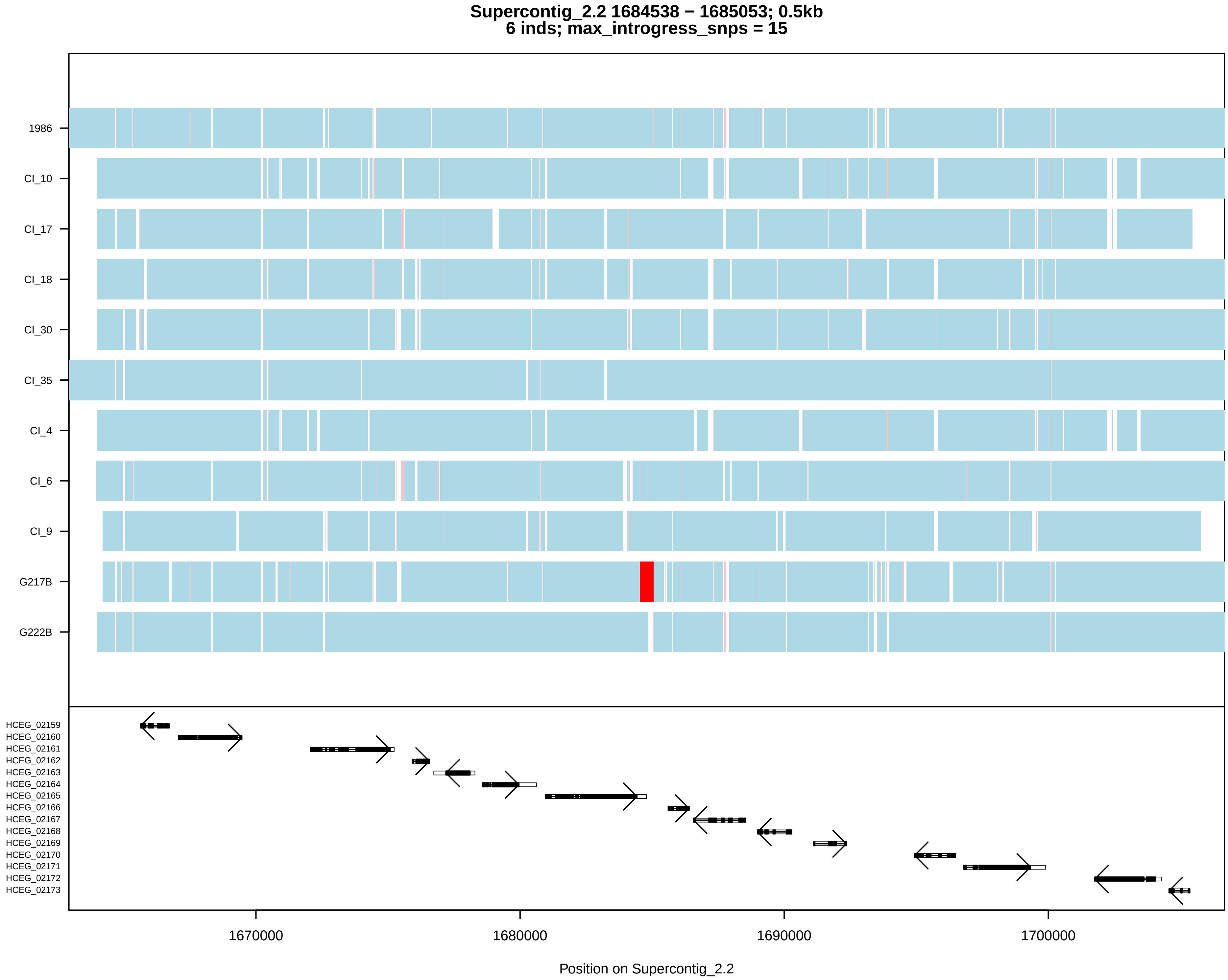
Supercontig_2.2 1684538 − 1685053; 0.5kb
6 inds; max_introgress_snps = 15
1986
CI_10
CI_17
CI_18
CI_30
CI_35
CI_4
CI_6
CI_9
G217B
G222B
HCEG_02159
HCEG_02160
HCEG_02161
HCEG_02162
HCEG_02163
HCEG_02164
HCEG_02165
HCEG_02166
HCEG_02167
HCEG_02168
HCEG_02169
HCEG_02170
HCEG_02171
HCEG_02172
HCEG_02173
1670000
1680000
1690000
1700000
Position on Supercontig_2.2

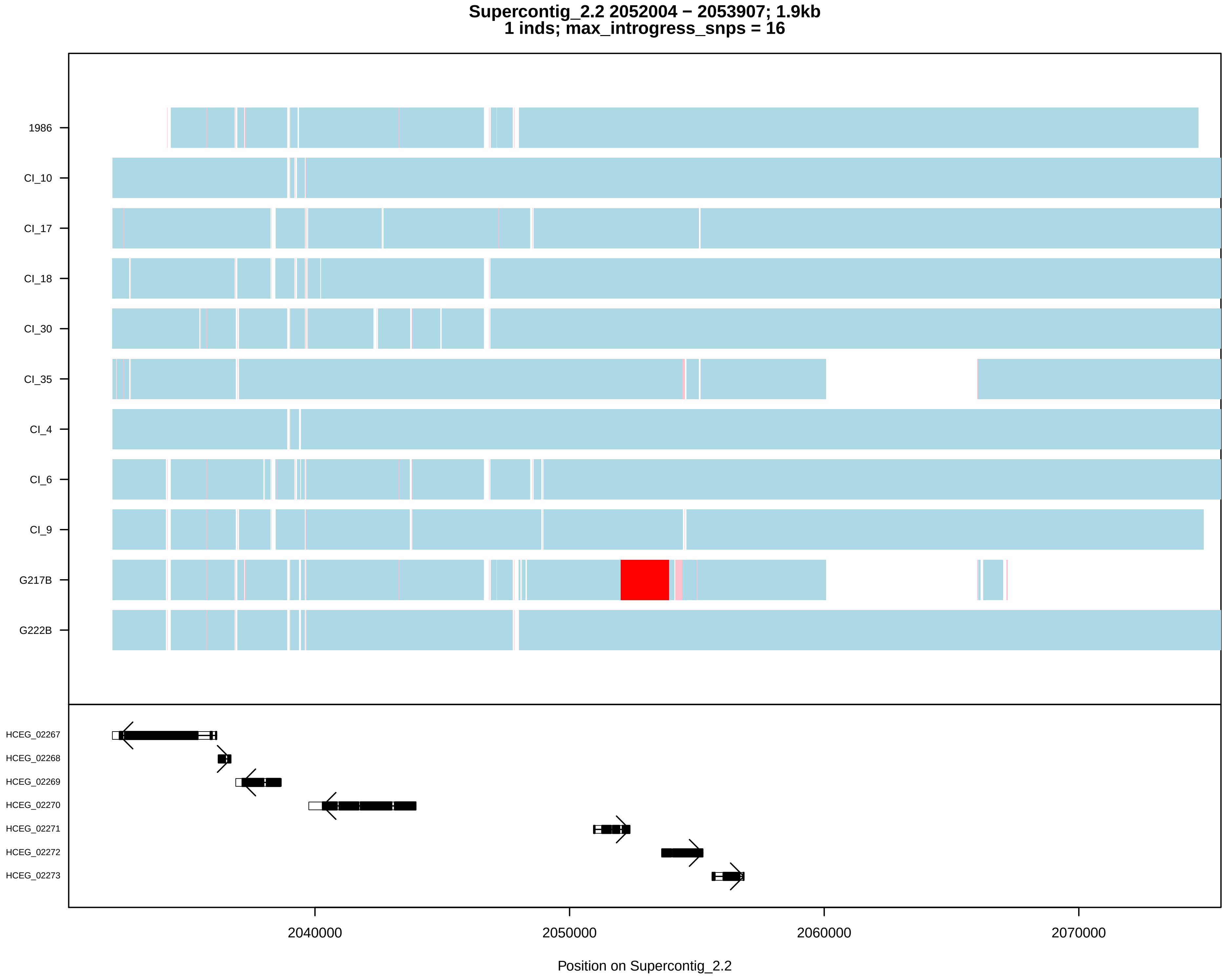

Supercontig_2.2 2052004 − 2053907; 1.9kb
1 inds; max_introgress_snps = 16
1986
CI_10
CI_17
CI_18
CI_30
CI_35
CI_4
CI_6
CI_9
G217B
G222B
HCEG_02267
HCEG_02268
HCEG_02269
HCEG_02270
HCEG_02271
HCEG_02272
HCEG_02273
2040000
2050000
2060000
2070000
Position on Supercontig_2.2

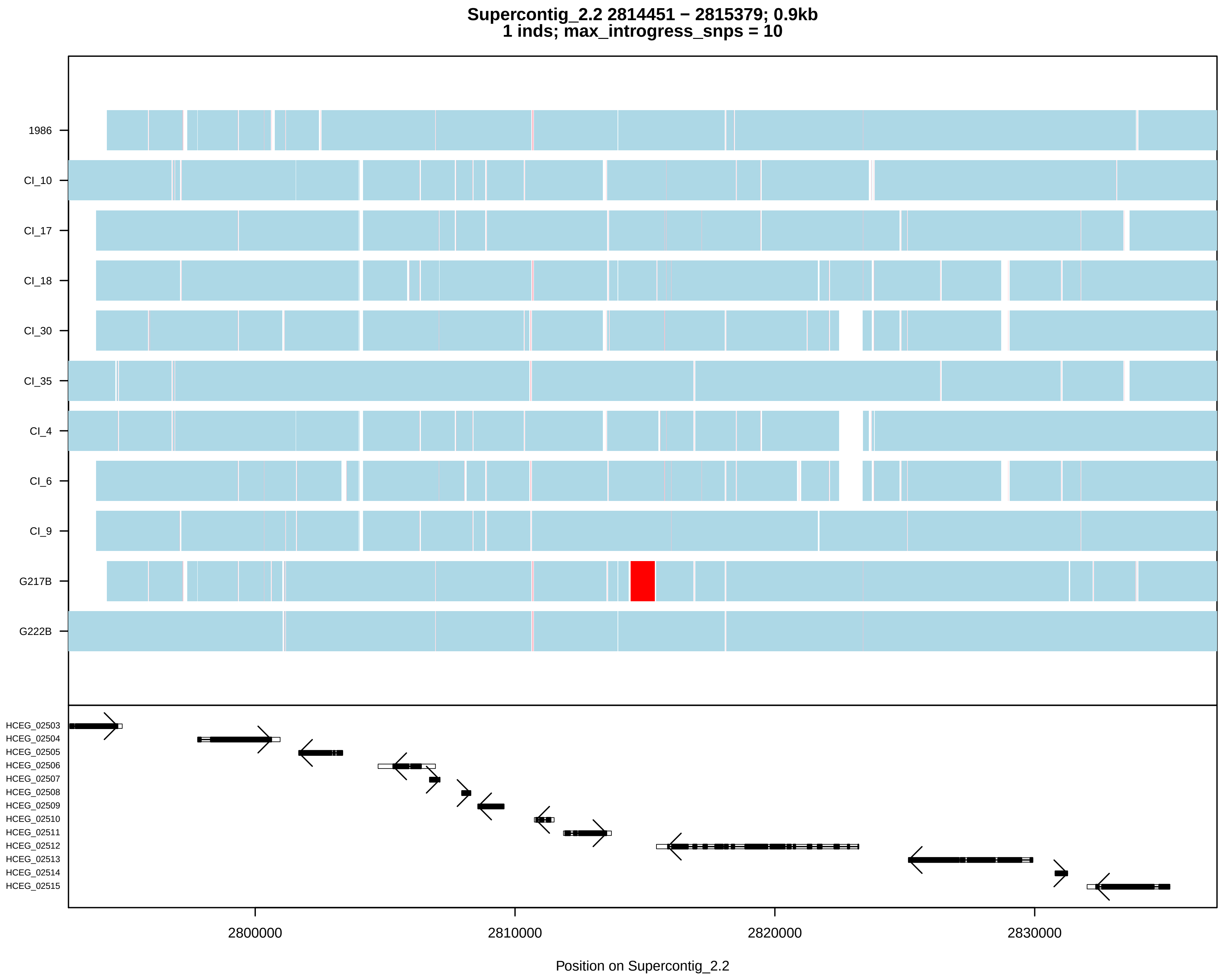

Supercontig_2.2 2814451 − 2815379; 0.9kb
1 inds; max_introgress_snps = 10
1986
CI_10
CI_17
CI_18
CI_30
CI_35
CI_4
CI_6
CI_9
G217B
G222B
HCEG_02503
HCEG_02504
HCEG_02505
HCEG_02506
HCEG_02507
HCEG_02508
HCEG_02509
HCEG_02510
HCEG_02511
HCEG_02512
HCEG_02513
HCEG_02514
HCEG_02515
2800000
2810000
2820000
2830000
Position on Supercontig_2.2

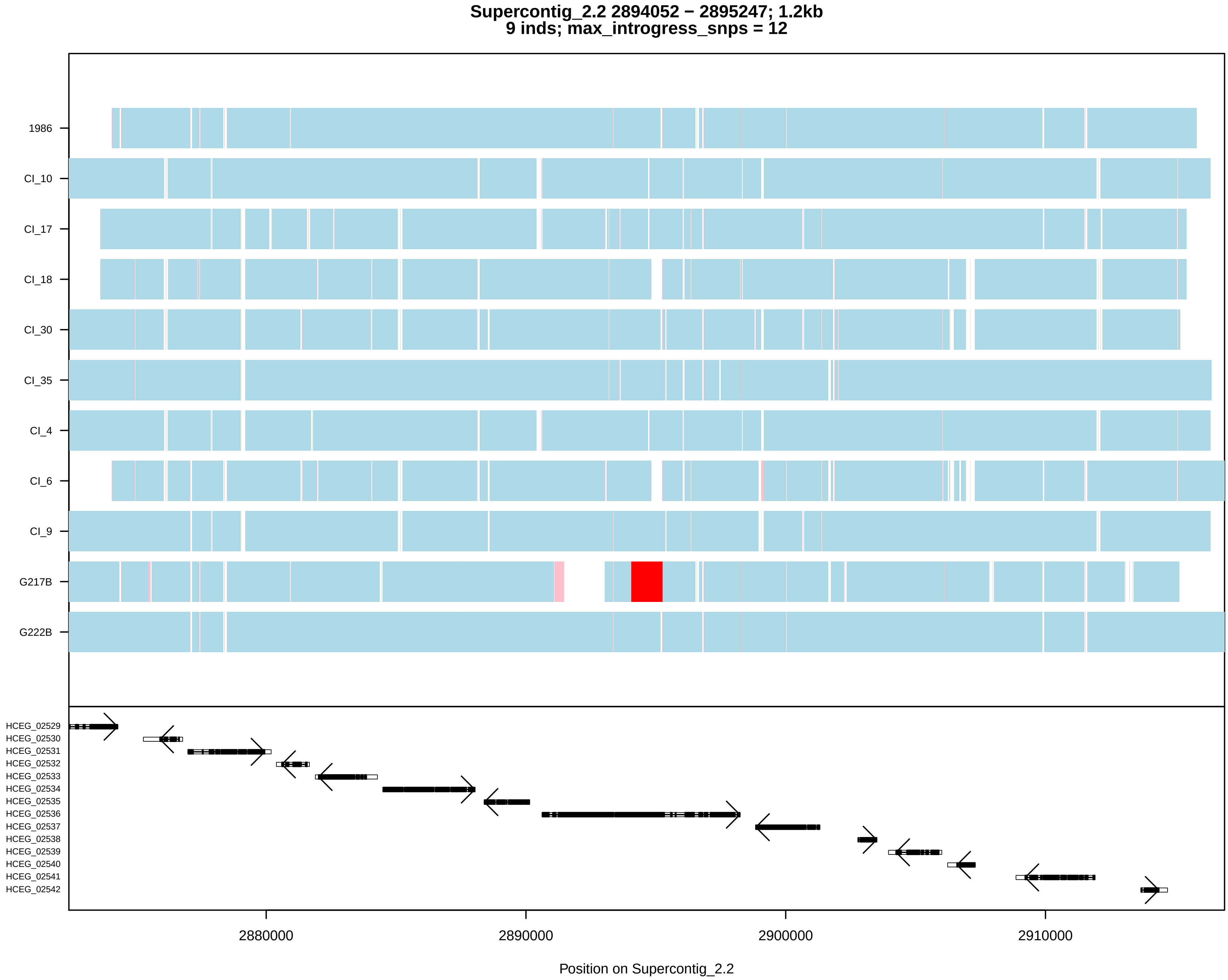

Supercontig_2.2 2894052 − 2895247; 1.2kb
9 inds; max_introgress_snps = 12
1986
CI_10
CI_17
CI_18
CI_30
CI_35
CI_4
CI_6
CI_9
G217B
G222B
HCEG_02529
HCEG_02530
HCEG_02531
HCEG_02532
HCEG_02533
HCEG_02534
HCEG_02535
HCEG_02536
HCEG_02537
HCEG_02538
HCEG_02539
HCEG_02540
HCEG_02541
HCEG_02542
2880000
2890000
2900000
2910000
Position on Supercontig_2.2

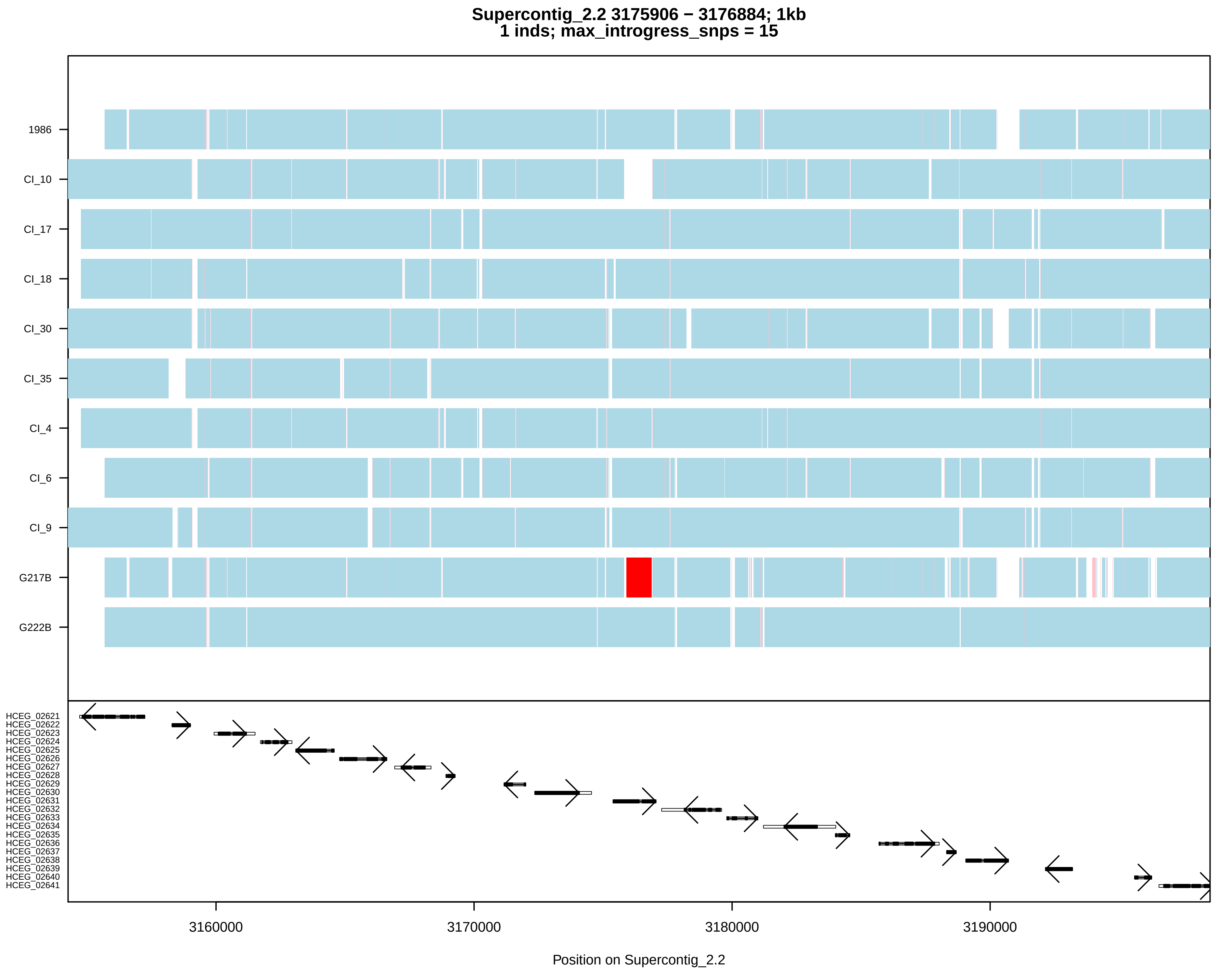

Supercontig_2.2 3175906 − 3176884; 1kb
1 inds; max_introgress_snps = 15
1986
CI_10
CI_17
CI_18
CI_30
CI_35
CI_4
CI_6
CI_9
G217B
G222B
HCEG_02621
HCEG_02622
HCEG_02623
HCEG_02624
HCEG_02625
HCEG_02626
HCEG_02627
HCEG_02628
HCEG_02629
HCEG_02630
HCEG_02631
HCEG_02632
HCEG_02633
HCEG_02634
HCEG_02635
HCEG_02636
HCEG_02637
HCEG_02638
HCEG_02639
HCEG_02640
HCEG_02641
3160000
3170000
3180000
3190000
Position on Supercontig_2.2

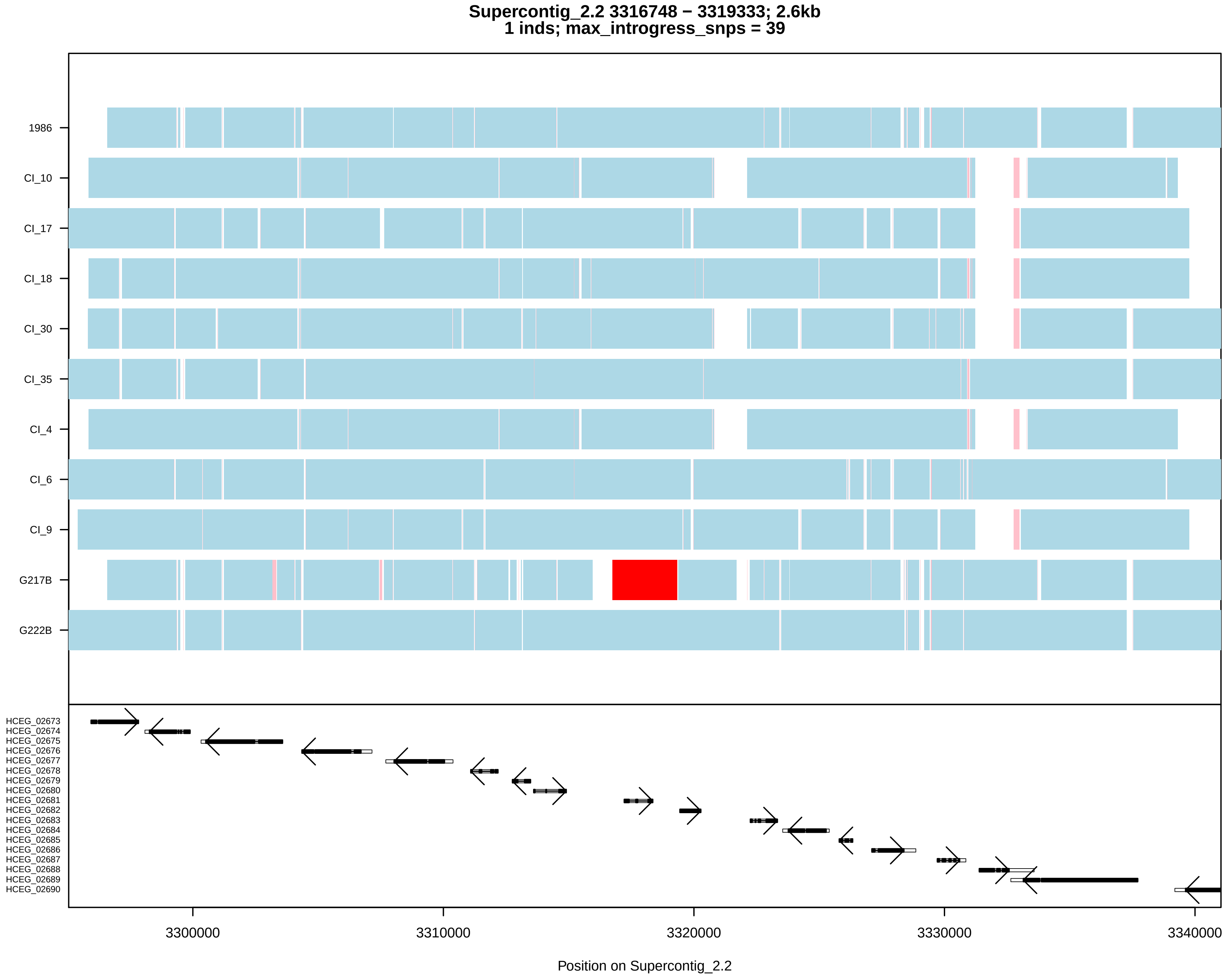

Supercontig_2.2 3316748 − 3319333; 2.6kb
1 inds; max_introgress_snps = 39
1986
CI_10
CI_17
CI_18
CI_30
CI_35
CI_4
CI_6
CI_9
G217B
G222B
HCEG_02673
HCEG_02674
HCEG_02675
HCEG_02676
HCEG_02677
HCEG_02678
HCEG_02679
HCEG_02680
HCEG_02681
HCEG_02682
HCEG_02683
HCEG_02684
HCEG_02685
HCEG_02686
HCEG_02687
HCEG_02688
HCEG_02689
HCEG_02690
3300000
3310000
3320000
3330000
3340000
Position on Supercontig_2.2

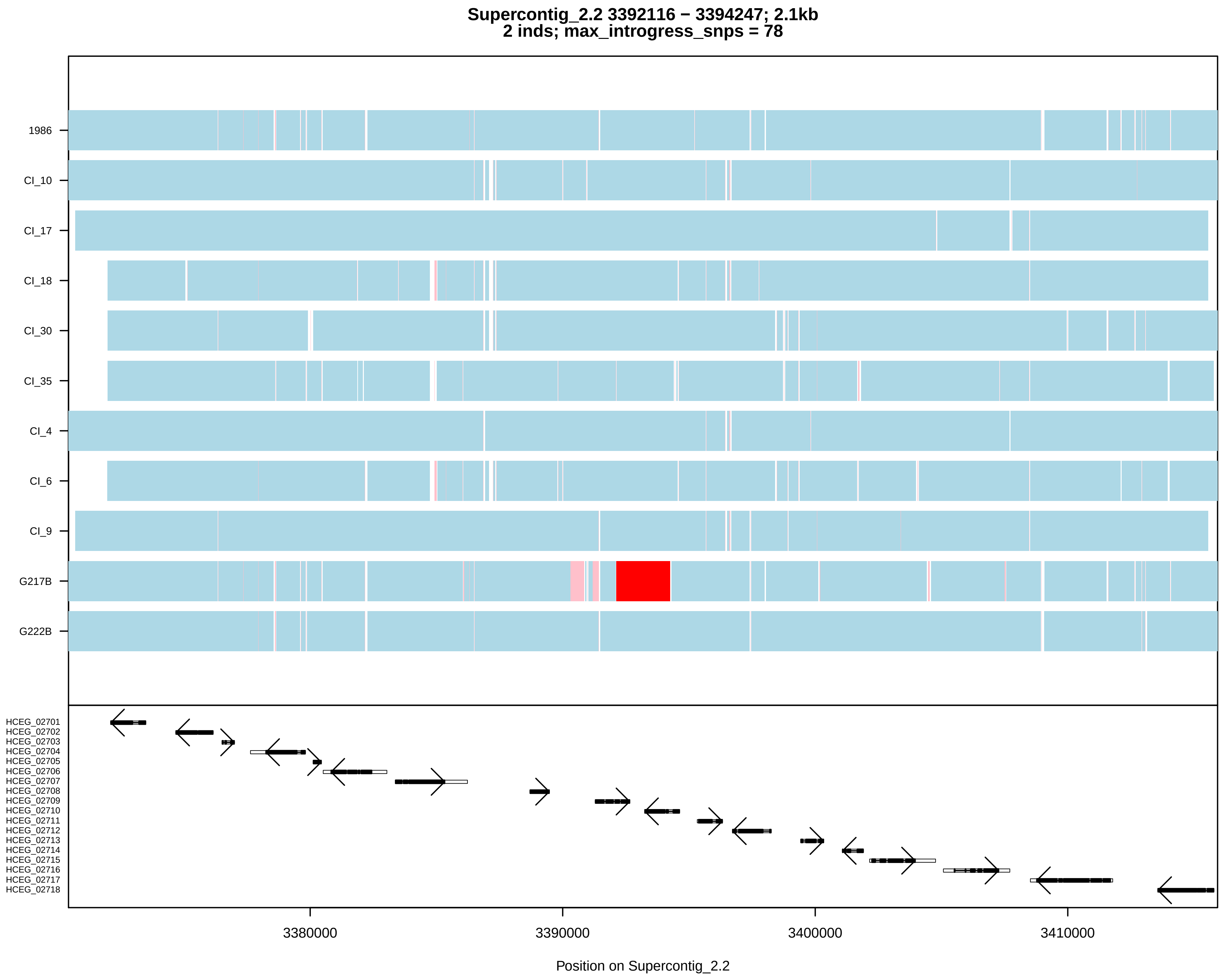

Supercontig_2.2 3392116 − 3394247; 2.1kb
2 inds; max_introgress_snps = 78
1986
CI_10
CI_17
CI_18
CI_30
CI_35
CI_4
CI_6
CI_9
G217B
G222B
HCEG_02701
HCEG_02702
HCEG_02703
HCEG_02704
HCEG_02705
HCEG_02706
HCEG_02707
HCEG_02708
HCEG_02709
HCEG_02710
HCEG_02711
HCEG_02712
HCEG_02713
HCEG_02714
HCEG_02715
HCEG_02716
HCEG_02717
HCEG_02718
3380000
3390000
3400000
3410000
Position on Supercontig_2.2

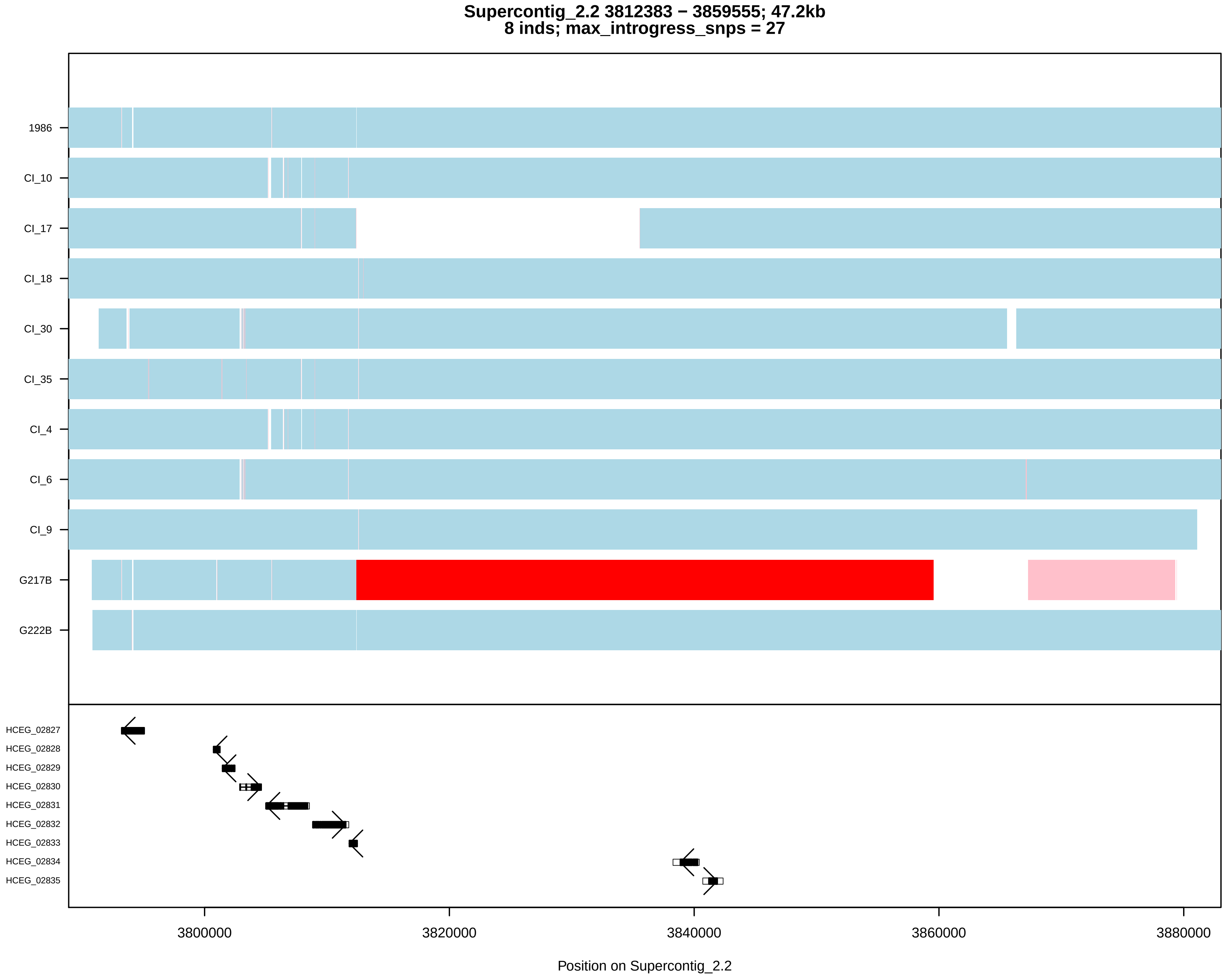

Supercontig_2.2 3812383 − 3859555; 47.2kb
8 inds; max_introgress_snps = 27
1986
CI_10
CI_17
CI_18
CI_30
CI_35
CI_4
CI_6
CI_9
G217B
G222B
HCEG_02827
HCEG_02828
HCEG_02829
HCEG_02830
HCEG_02831
HCEG_02832
HCEG_02833
HCEG_02834
HCEG_02835
3800000
3820000
3840000
3860000
3880000
Position on Supercontig_2.2

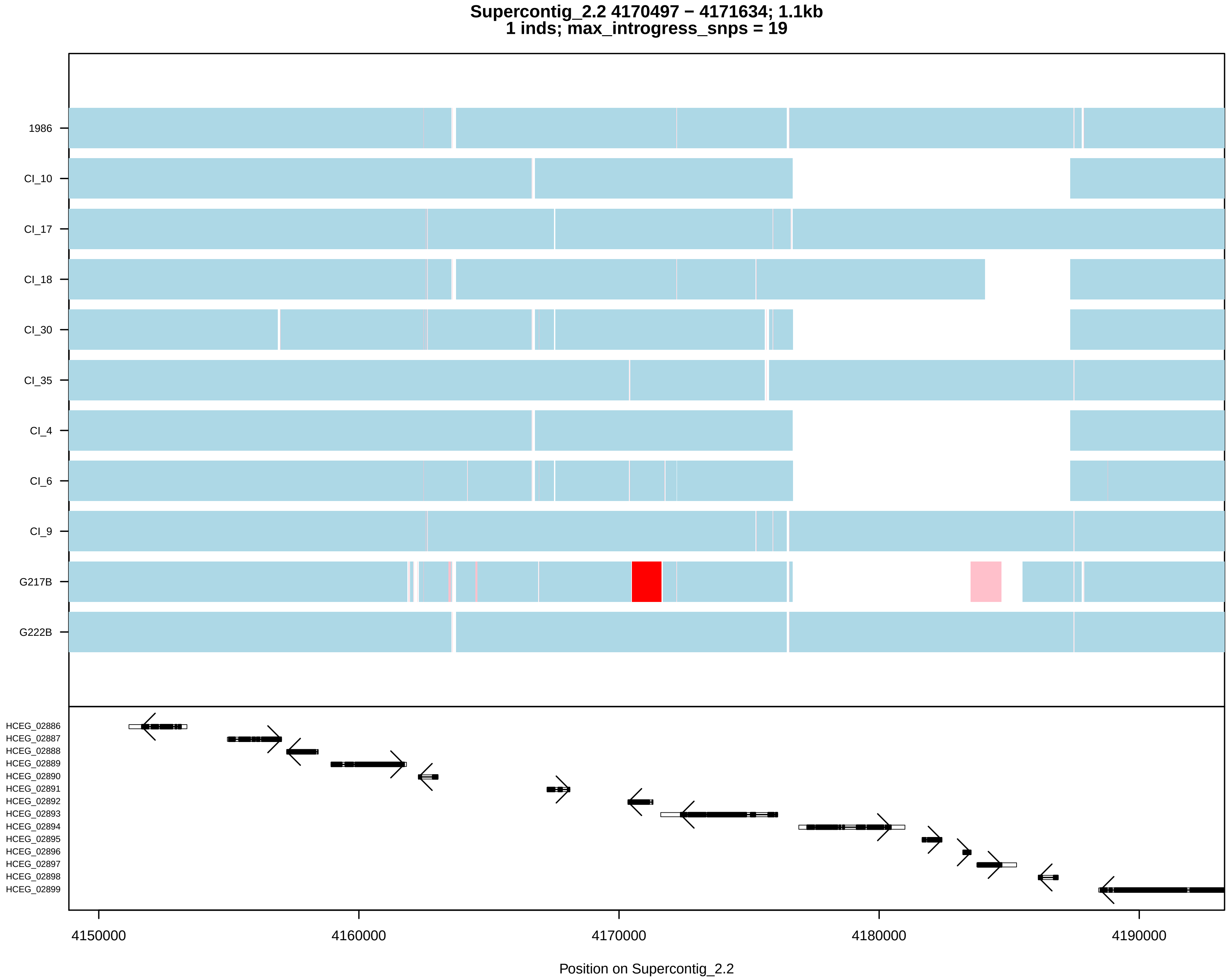
Supercontig_2.2 4170497 − 4171634; 1.1kb
1 inds; max_introgress_snps = 19
1986
CI_10
CI_17
CI_18
CI_30
CI_35
CI_4
CI_6
CI_9
G217B
G222B
HCEG_02886
HCEG_02887
HCEG_02888
HCEG_02889
HCEG_02890
HCEG_02891
HCEG_02892
HCEG_02893
HCEG_02894
HCEG_02895
HCEG_02896
HCEG_02897
HCEG_02898
HCEG_02899
4150000
4160000
4170000
4180000
4190000
Position on Supercontig_2.2

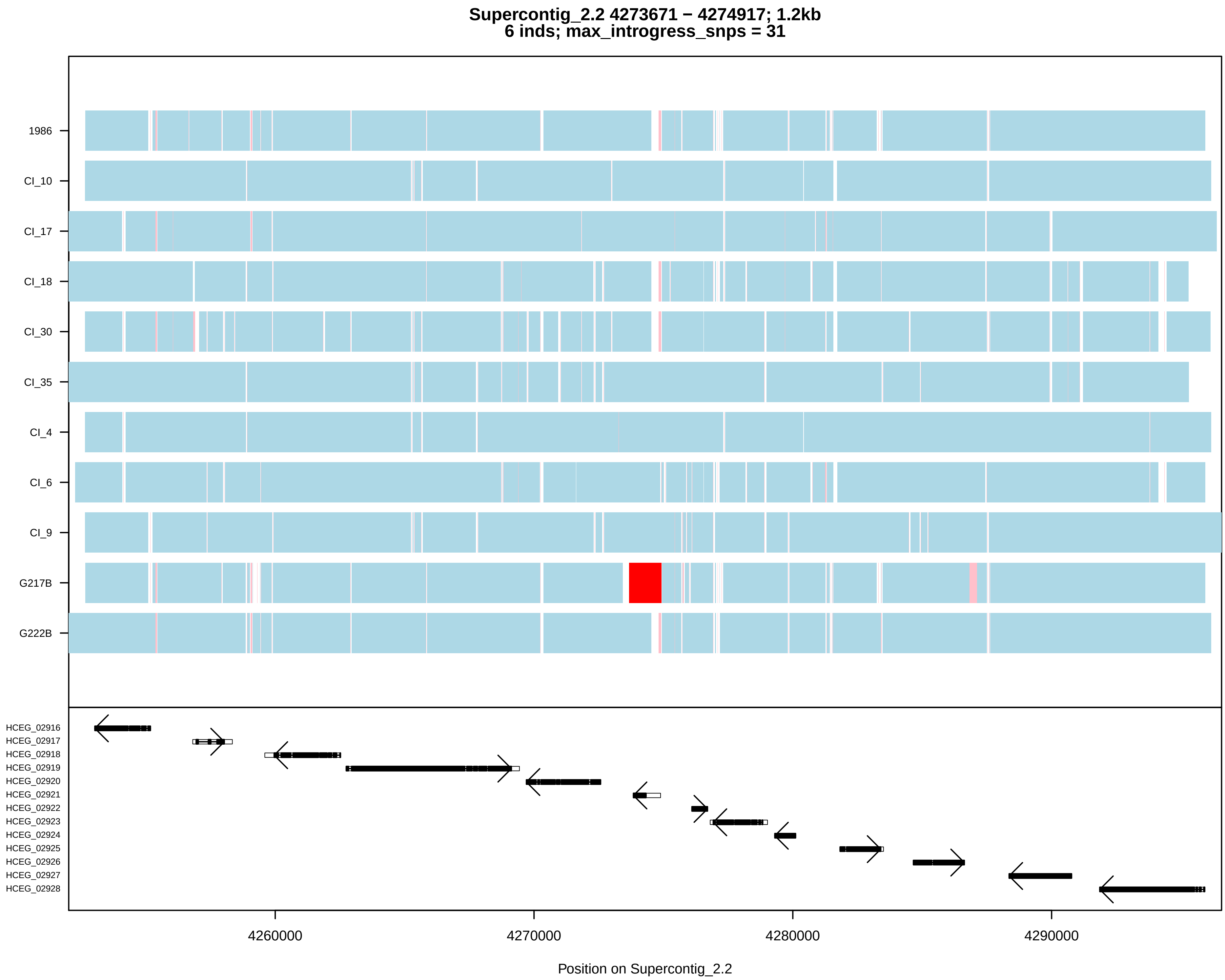
Supercontig_2.2 4273671 − 4274917; 1.2kb
6 inds; max_introgress_snps = 31
1986
CI_10
CI_17
CI_18
CI_30
CI_35
CI_4
CI_6
CI_9
G217B
G222B
HCEG_02916
HCEG_02917
HCEG_02918
HCEG_02919
HCEG_02920
HCEG_02921
HCEG_02922
HCEG_02923
HCEG_02924
HCEG_02925
HCEG_02926
HCEG_02927
HCEG_02928
4260000
4270000
4280000
4290000
Position on Supercontig_2.2

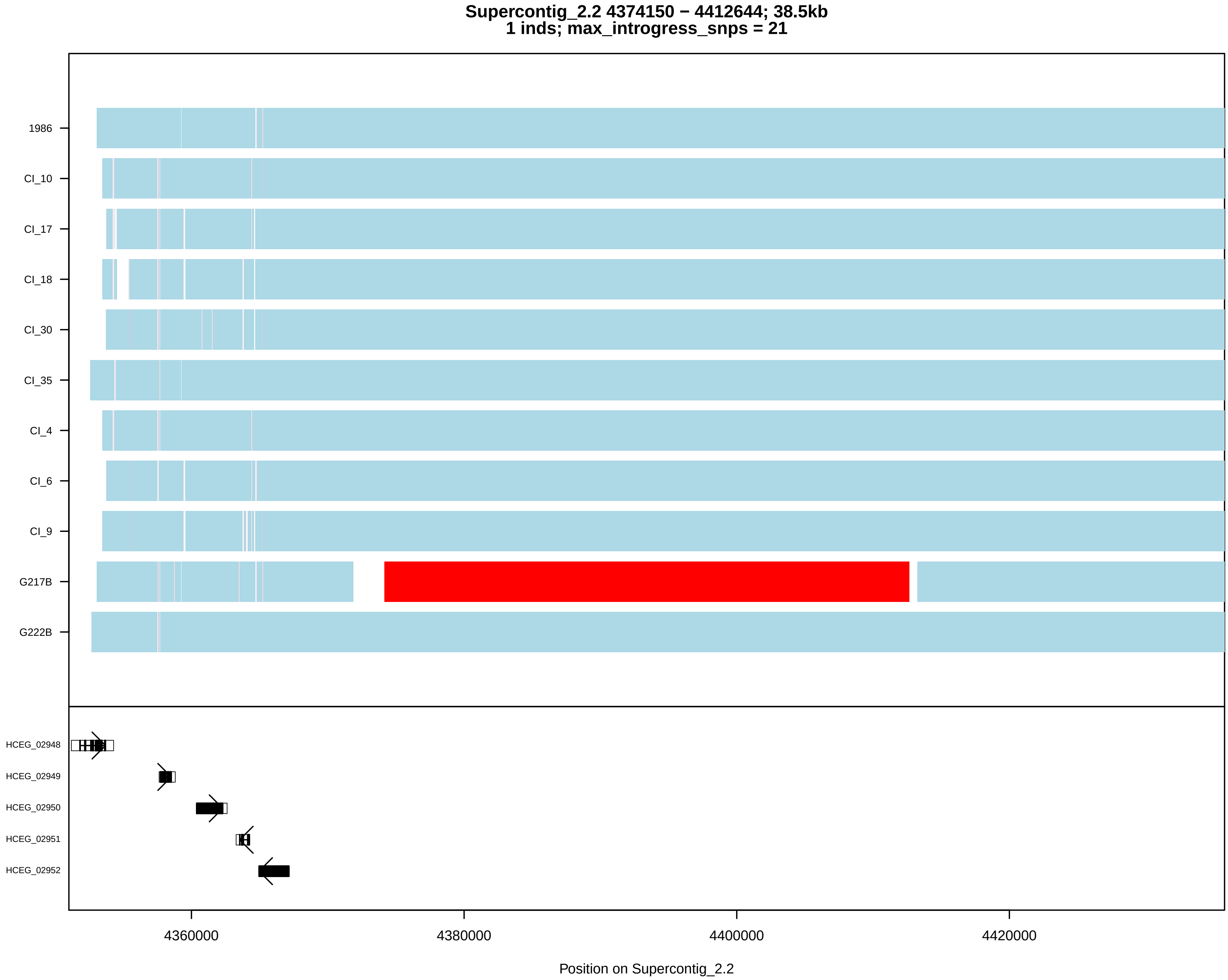
Supercontig_2.2 4374150 − 4412644; 38.5kb
1 inds; max_introgress_snps = 21
1986
CI_10
CI_17
CI_18
CI_30
CI_35
CI_4
CI_6
CI_9
G217B
G222B
HCEG_02948
HCEG_02949
HCEG_02950
HCEG_02951
HCEG_02952
4360000
4380000
4400000
4420000
Position on Supercontig_2.2

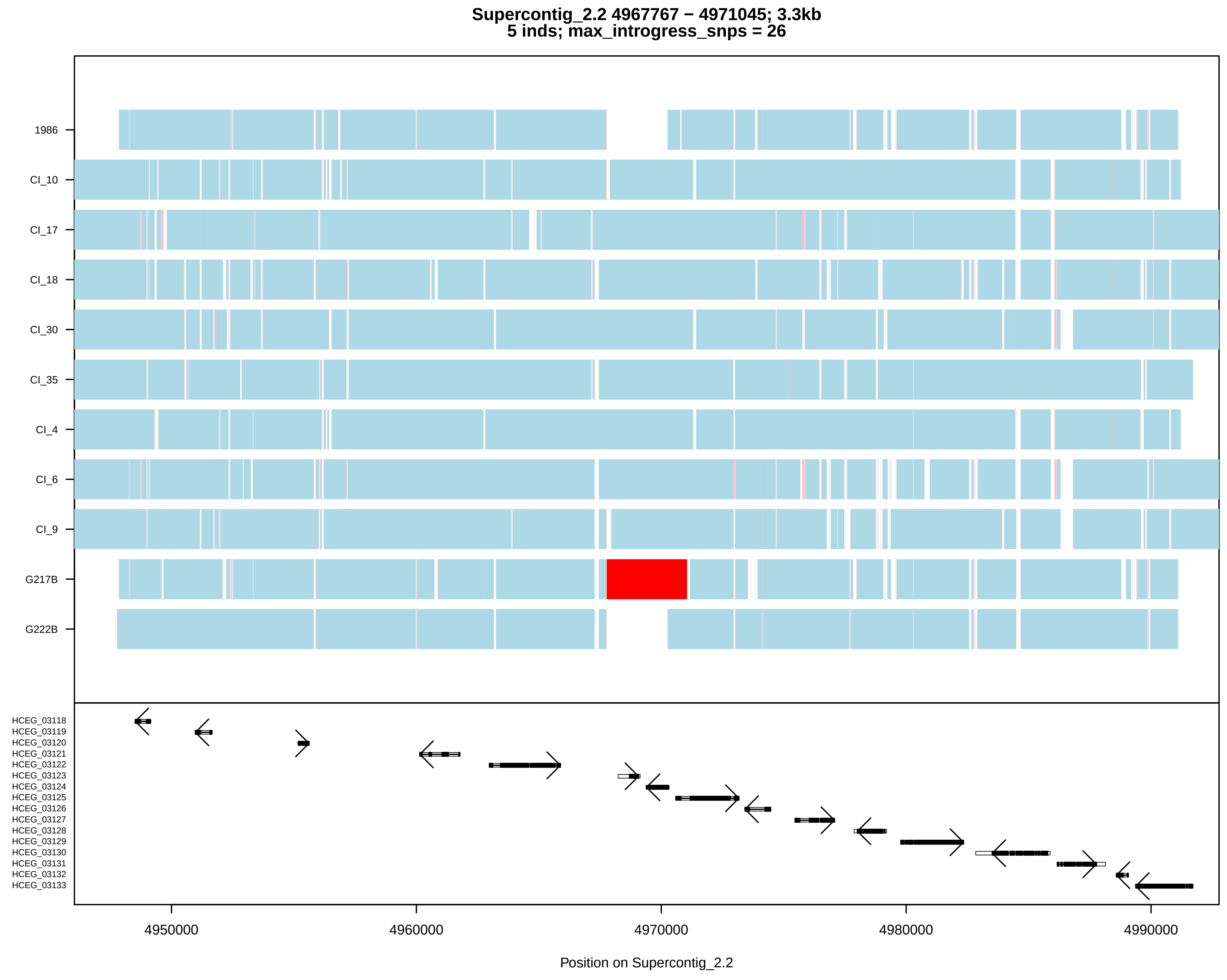
Supercontig_2.2 4967767 − 4971045; 3.3kb
5 inds; max_introgress_snps = 26
1986
CI_10
CI_17
CI_18
CI_30
CI_35
CI_4
CI_6
CI_9
G217B
G222B
HCEG_03118
HCEG_03119
HCEG_03120
HCEG_03121
HCEG_03122
HCEG_03123
HCEG_03124
HCEG_03125
HCEG_03126
HCEG_03127
HCEG_03128
HCEG_03129
HCEG_03130
HCEG_03131
HCEG_03132
HCEG_03133
4950000
4960000
4970000
4980000
4990000
Position on Supercontig_2.2

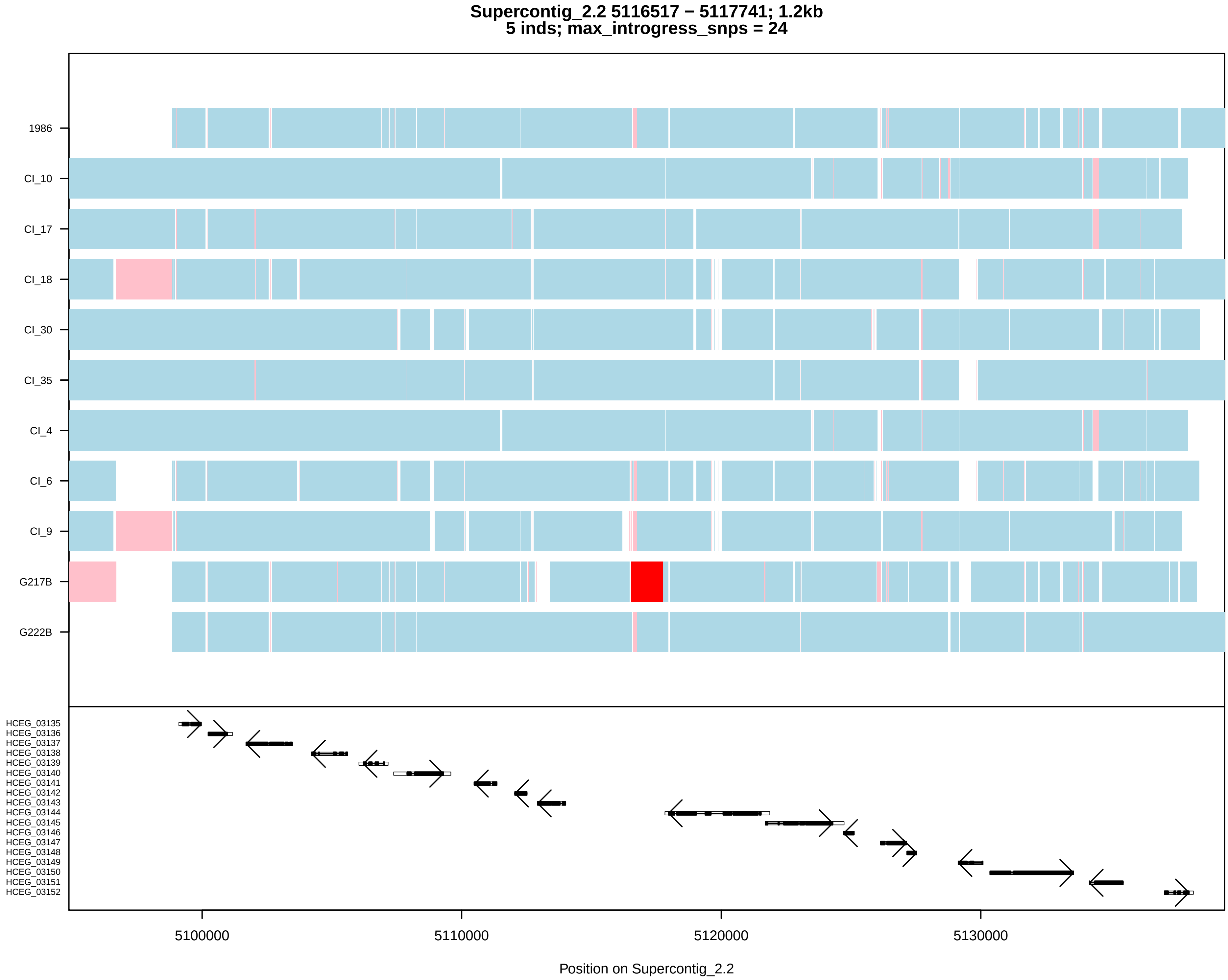
Supercontig_2.2 5116517 − 5117741; 1.2kb
5 inds; max_introgress_snps = 24
1986
CI_10
CI_17
CI_18
CI_30
CI_35
CI_4
CI_6
CI_9
G217B
G222B
HCEG_03135
HCEG_03136
HCEG_03137
HCEG_03138
HCEG_03139
HCEG_03140
HCEG_03141
HCEG_03142
HCEG_03143
HCEG_03144
HCEG_03145
HCEG_03146
HCEG_03147
HCEG_03148
HCEG_03149
HCEG_03150
HCEG_03151
HCEG_03152
5100000
5110000
5120000
5130000
Position on Supercontig_2.2

Supercontig_2.2 5169429 − 5170399; 1kb
1 inds; max_introgress_snps = 16

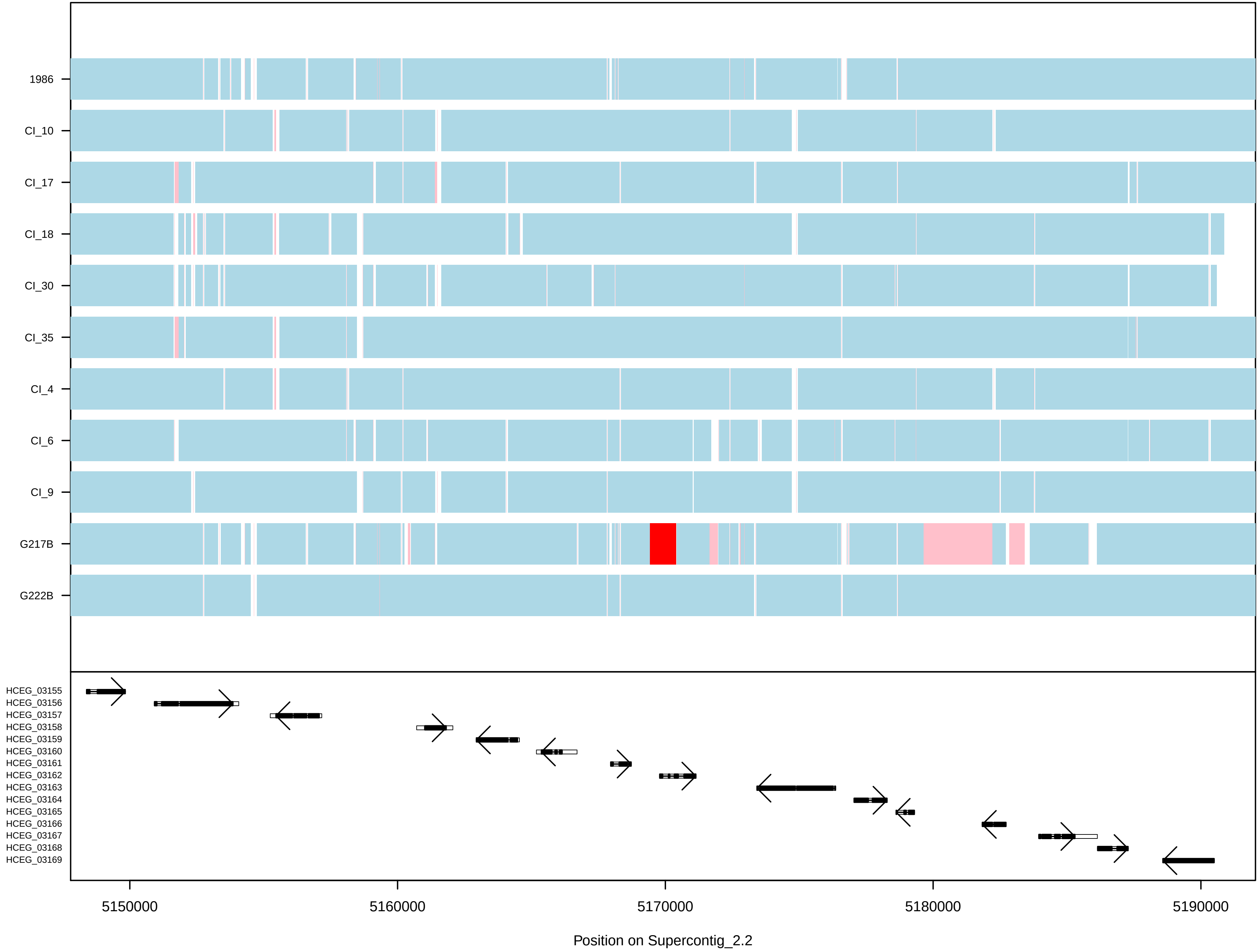

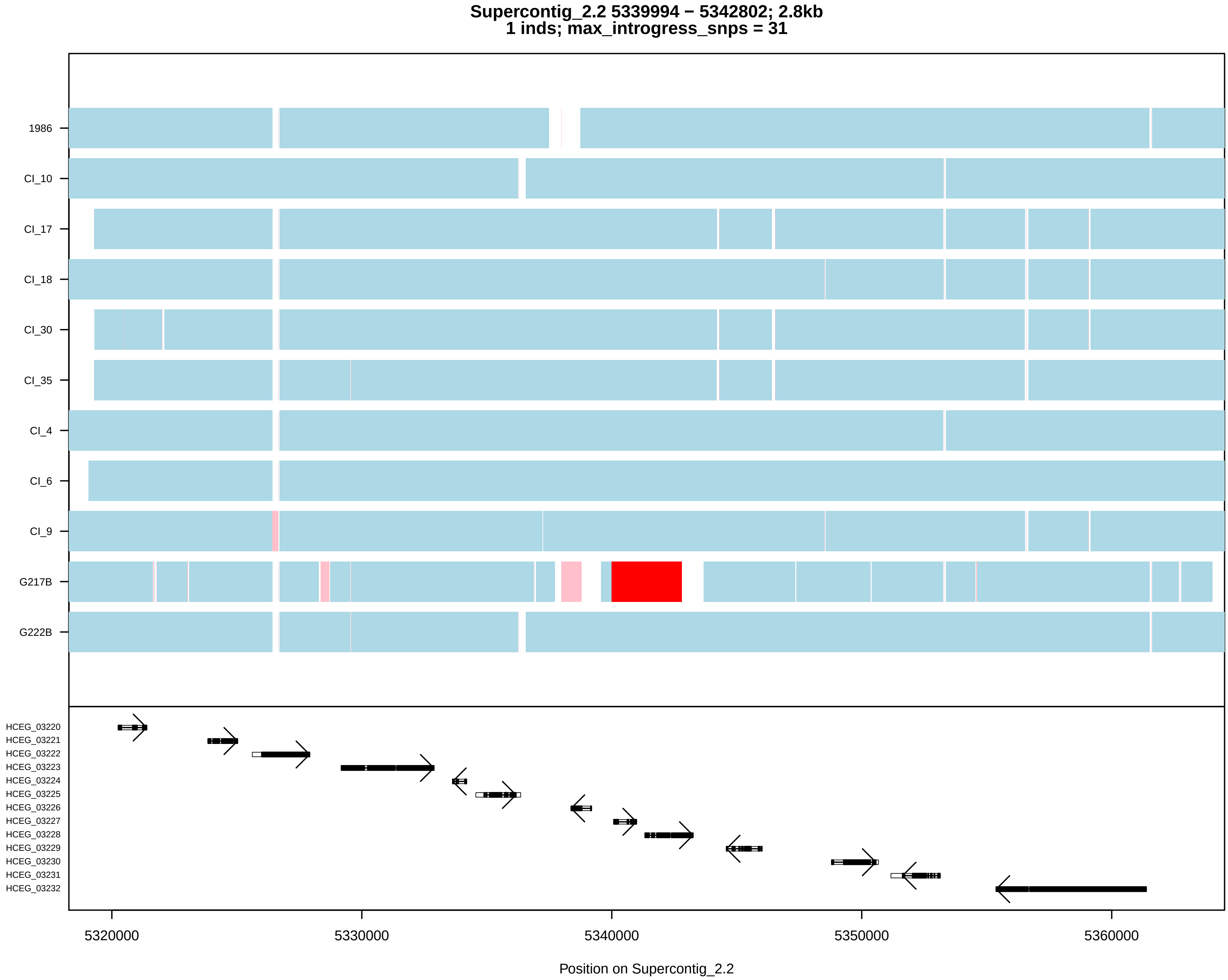
Supercontig_2.2 5339994 − 5342802; 2.8kb
1 inds; max_introgress_snps = 31
1986
CI_10
CI_17
CI_18
CI_30
CI_35
CI_4
CI_6
CI_9
G217B
G222B
HCEG_03220
HCEG_03221
HCEG_03222
HCEG_03223
HCEG_03224
HCEG_03225
HCEG_03226
HCEG_03227
HCEG_03228
HCEG_03229
HCEG_03230
HCEG_03231
HCEG_03232
5320000
5330000
5340000
5350000
5360000
Position on Supercontig_2.2

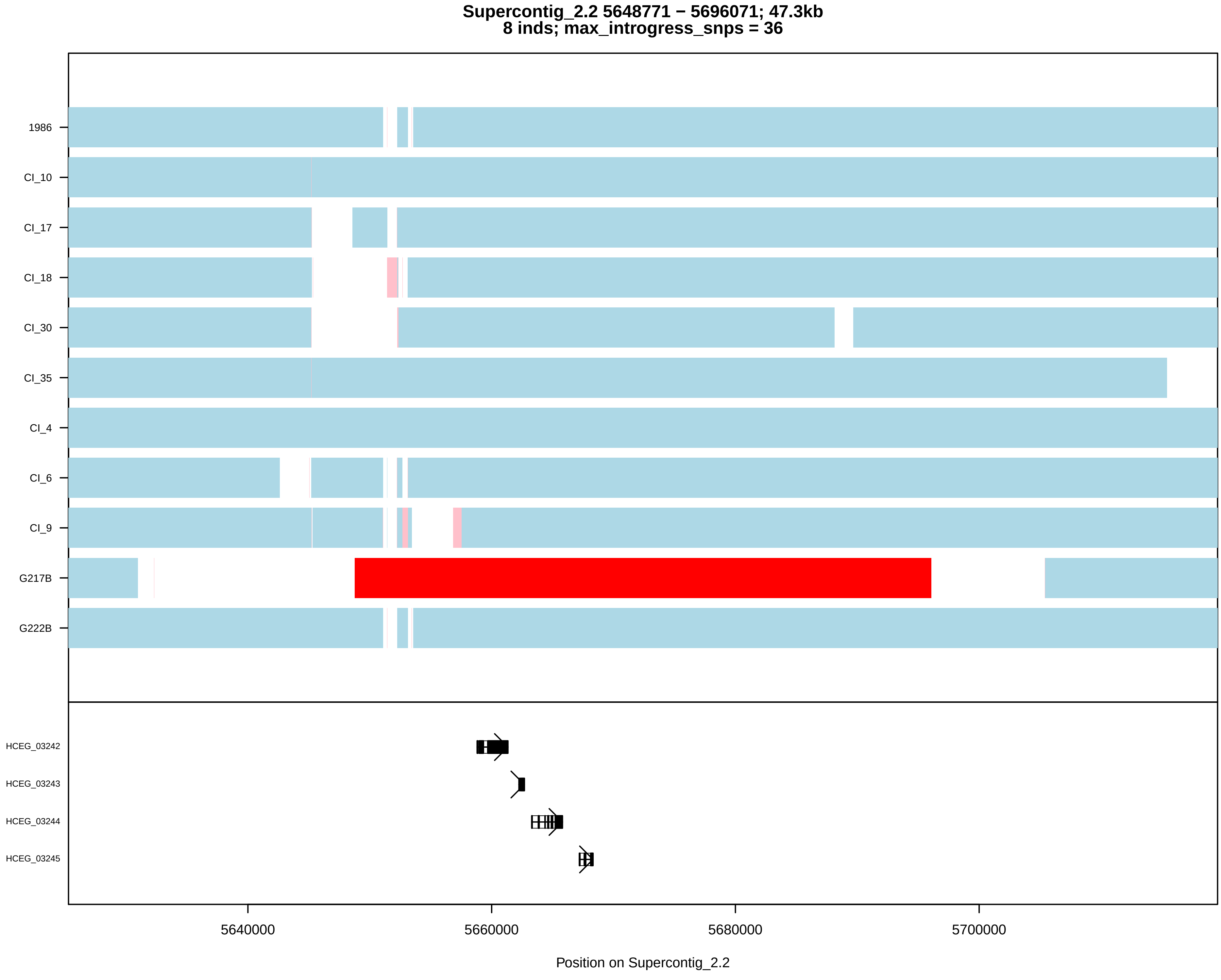
Supercontig_2.2 5648771 − 5696071; 47.3kb
8 inds; max_introgress_snps = 36
1986
CI_10
CI_17
CI_18
CI_30
CI_35
CI_4
CI_6
CI_9
G217B
G222B
HCEG_03242
HCEG_03243
HCEG_03244
HCEG_03245
5640000
5660000
5680000
5700000
Position on Supercontig_2.2

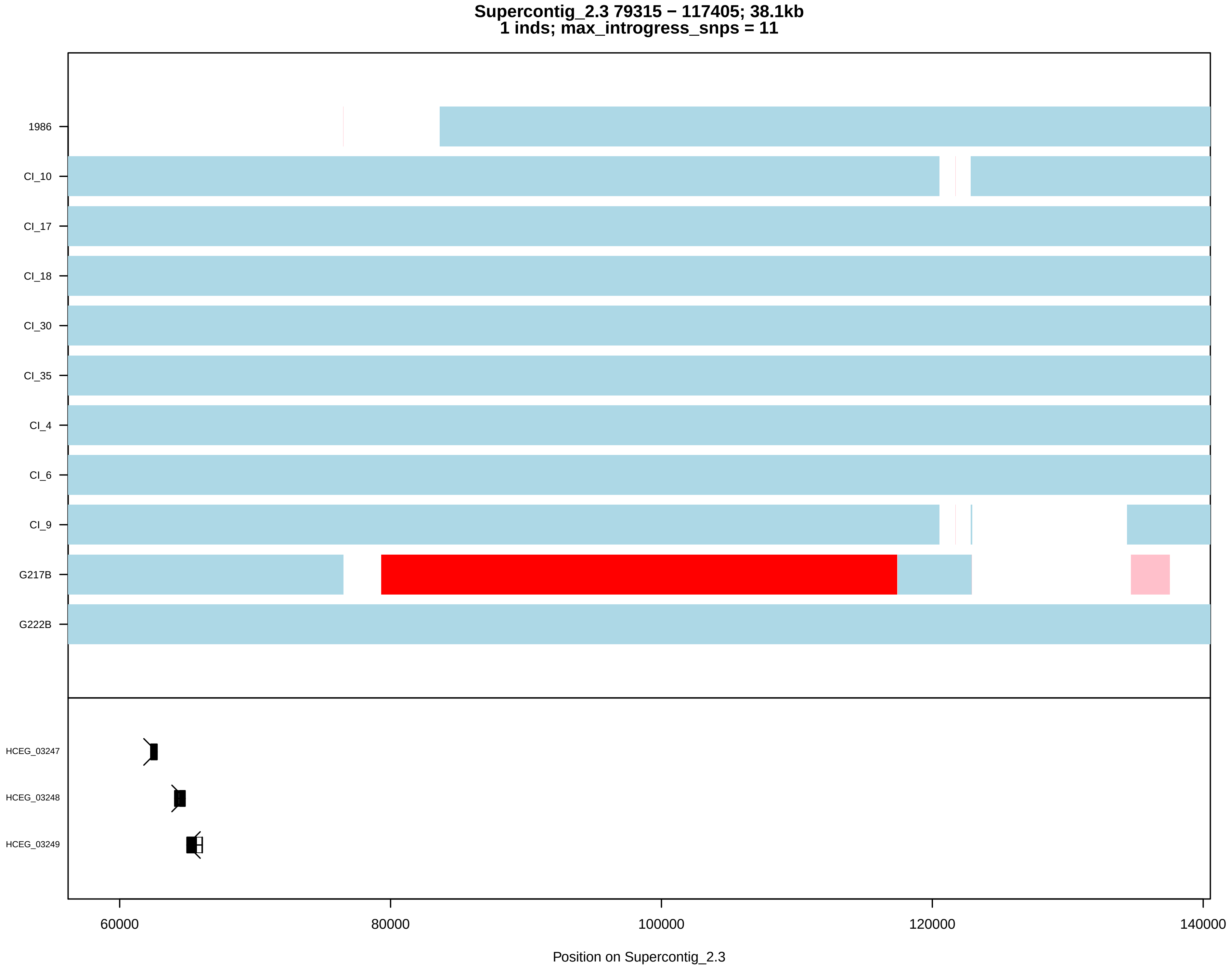
Supercontig_2.3 79315 − 117405; 38.1kb
1 inds; max_introgress_snps = 11
1986
CI_10
CI_17
CI_18
CI_30
CI_35
CI_4
CI_6
CI_9
G217B
G222B
HCEG_03247
HCEG_03248
HCEG_03249
60000
80000
100000
120000
140000
Position on Supercontig_2.3

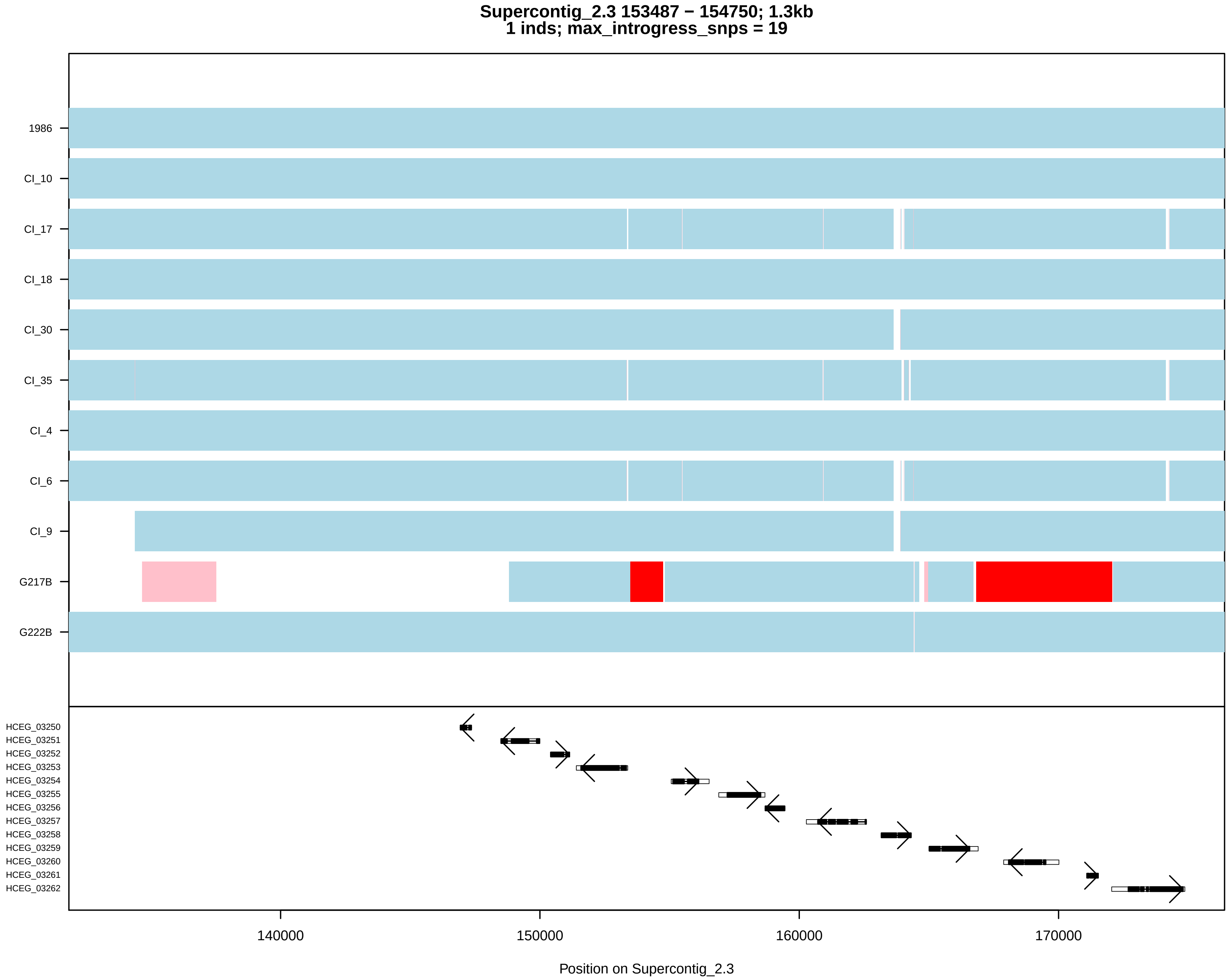
Supercontig_2.3 153487 − 154750; 1.3kb
1 inds; max_introgress_snps = 19
1986
CI_10
CI_17
CI_18
CI_30
CI_35
CI_4
CI_6
CI_9
G217B
G222B
HCEG_03250
HCEG_03251
HCEG_03252
HCEG_03253
HCEG_03254
HCEG_03255
HCEG_03256
HCEG_03257
HCEG_03258
HCEG_03259
HCEG_03260
HCEG_03261
HCEG_03262
140000
150000
160000
170000
Position on Supercontig_2.3

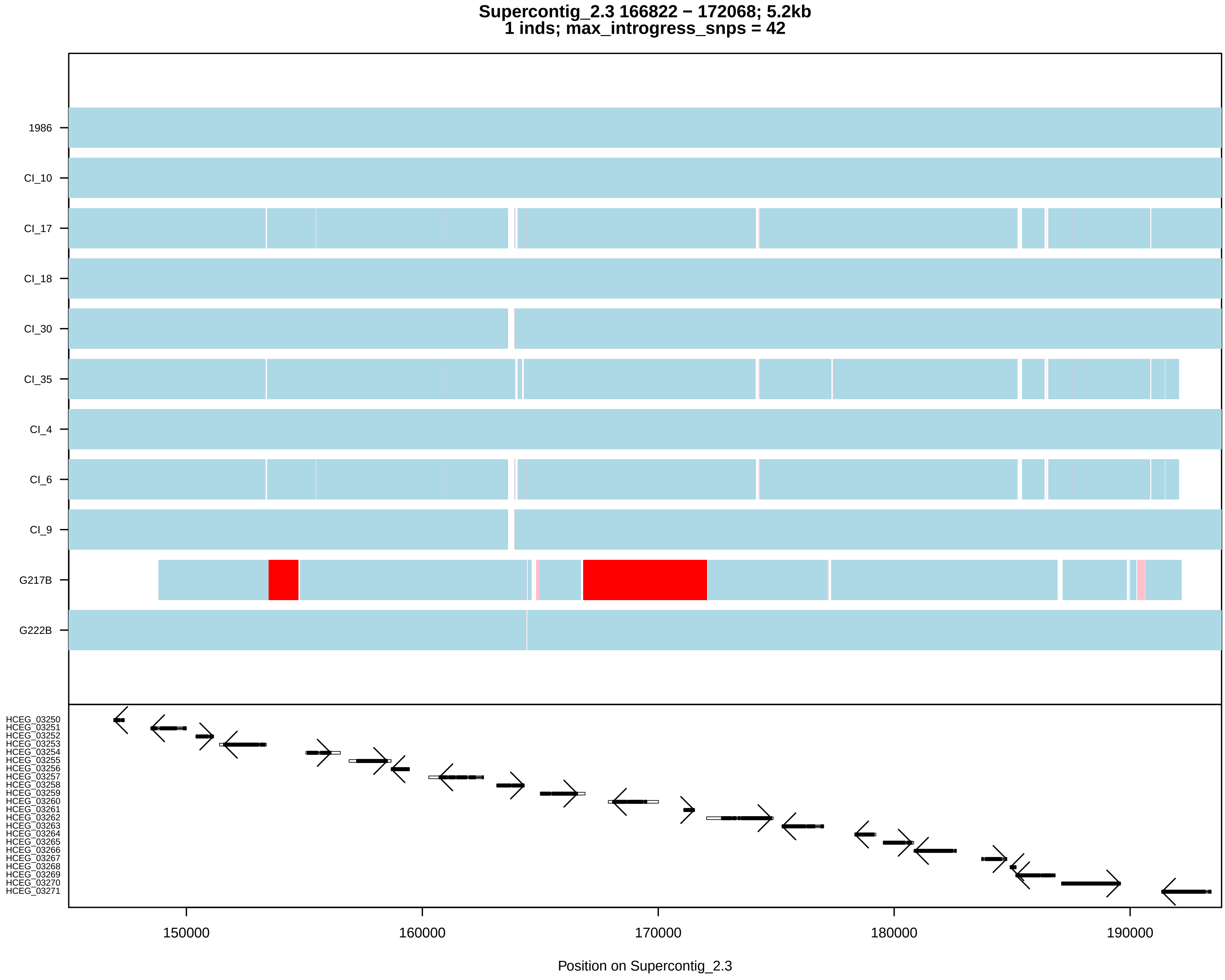
Supercontig_2.3 166822 − 172068; 5.2kb
1 inds; max_introgress_snps = 42
1986
CI_10
CI_17
CI_18
CI_30
CI_35
CI_4
CI_6
CI_9
G217B
G222B
HCEG_03250
HCEG_03251
HCEG_03252
HCEG_03253
HCEG_03254
HCEG_03255
HCEG_03256
HCEG_03257
HCEG_03258
HCEG_03259
HCEG_03260
HCEG_03261
HCEG_03262
HCEG_03263
HCEG_03264
HCEG_03265
HCEG_03266
HCEG_03267
HCEG_03268
HCEG_03269
HCEG_03270
HCEG_03271
150000
160000
170000
180000
190000
Position on Supercontig_2.3

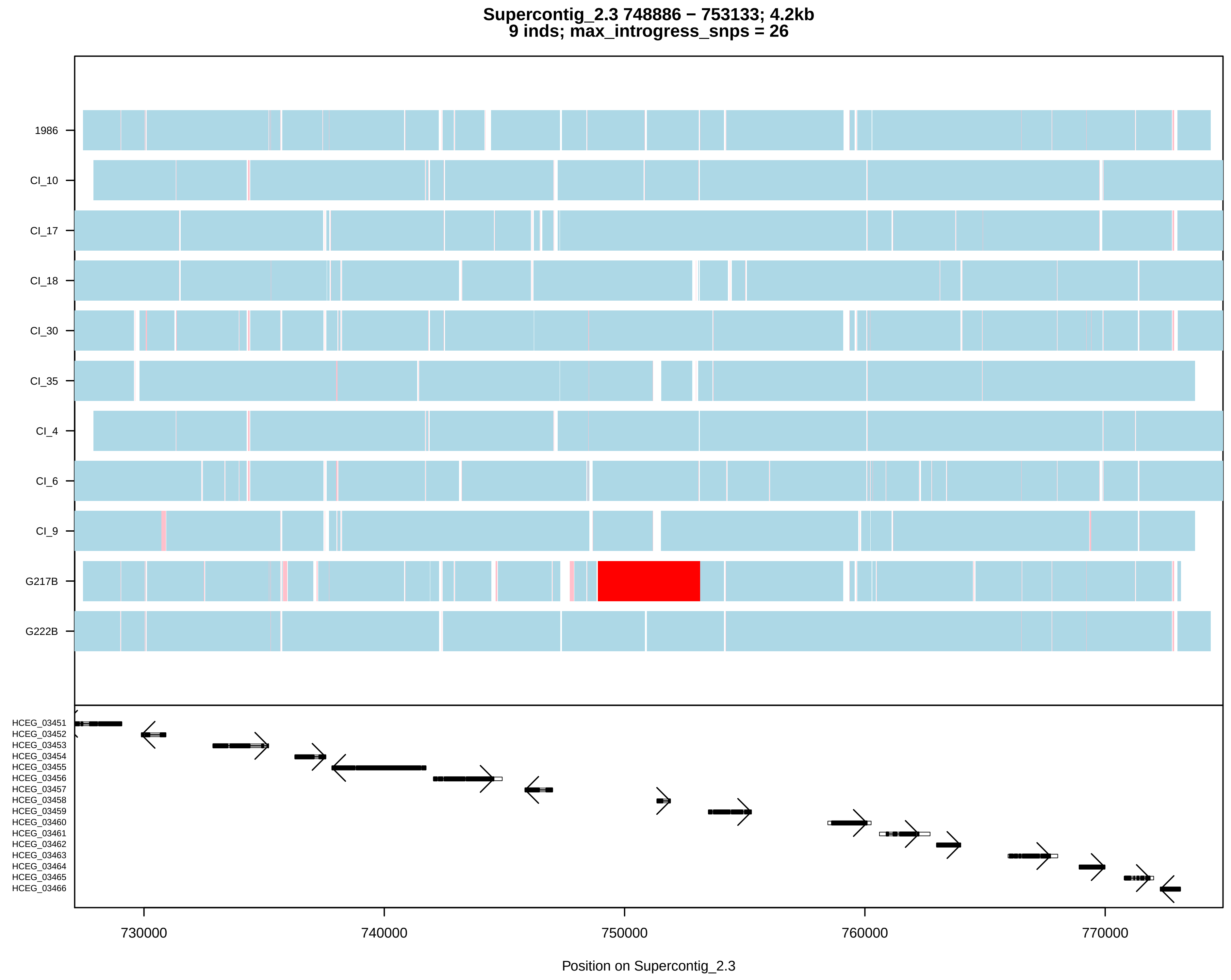
Supercontig_2.3 748886 − 753133; 4.2kb
9 inds; max_introgress_snps = 26
1986
CI_10
CI_17
CI_18
CI_30
CI_35
CI_4
CI_6
CI_9
G217B
G222B
HCEG_03451
HCEG_03452
HCEG_03453
HCEG_03454
HCEG_03455
HCEG_03456
HCEG_03457
HCEG_03458
HCEG_03459
HCEG_03460
HCEG_03461
HCEG_03462
HCEG_03463
HCEG_03464
HCEG_03465
HCEG_03466
730000
740000
750000
760000
770000
Position on Supercontig_2.3

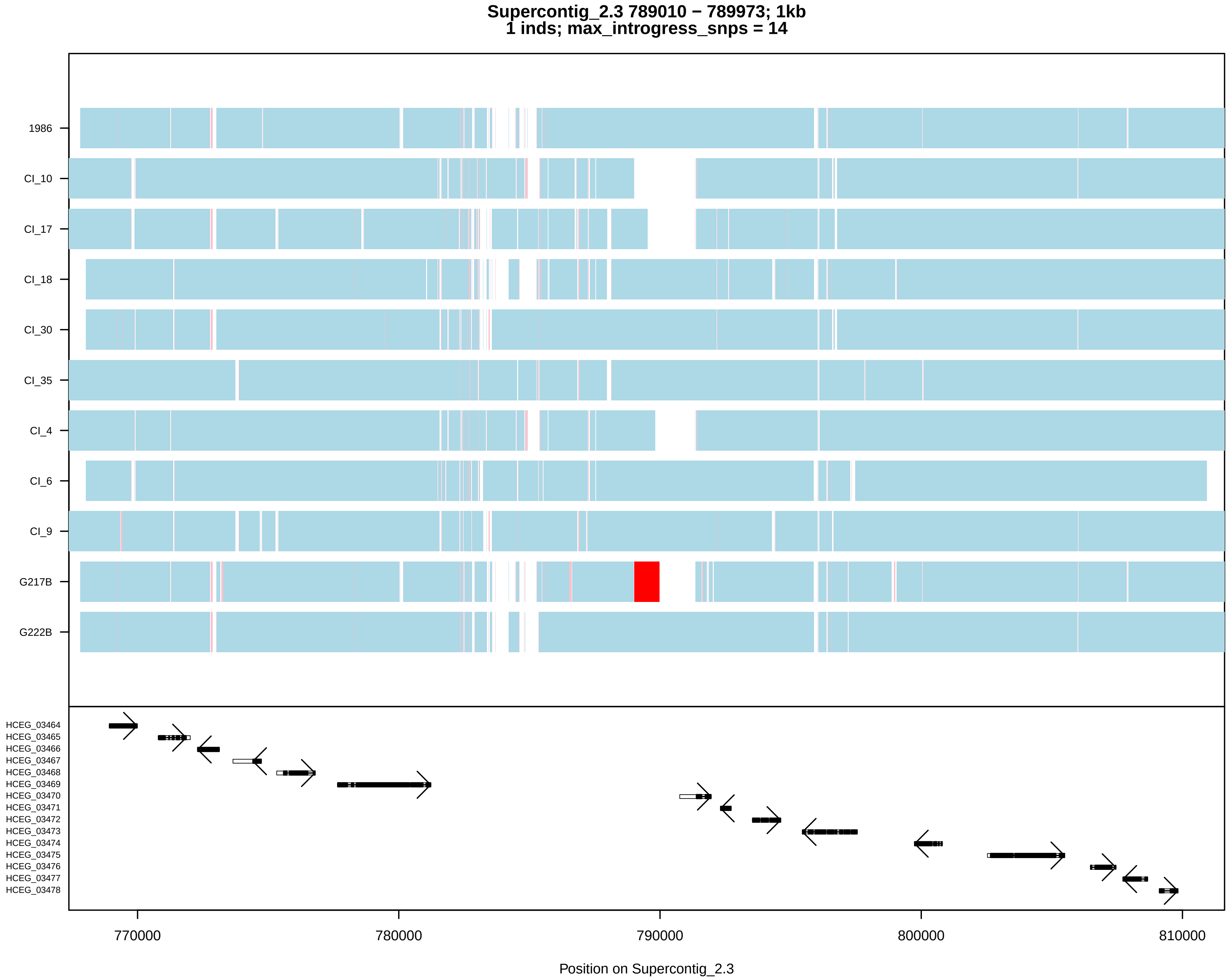

Supercontig_2.3 789010 − 789973; 1kb
1 inds; max_introgress_snps = 14
1986
CI_10
CI_17
CI_18
CI_30
CI_35
CI_4
CI_6
CI_9
G217B
G222B
HCEG_03464
HCEG_03465
HCEG_03466
HCEG_03467
HCEG_03468
HCEG_03469
HCEG_03470
HCEG_03471
HCEG_03472
HCEG_03473
HCEG_03474
HCEG_03475
HCEG_03476
HCEG_03477
HCEG_03478
770000
780000
790000
800000
810000
Position on Supercontig_2.3

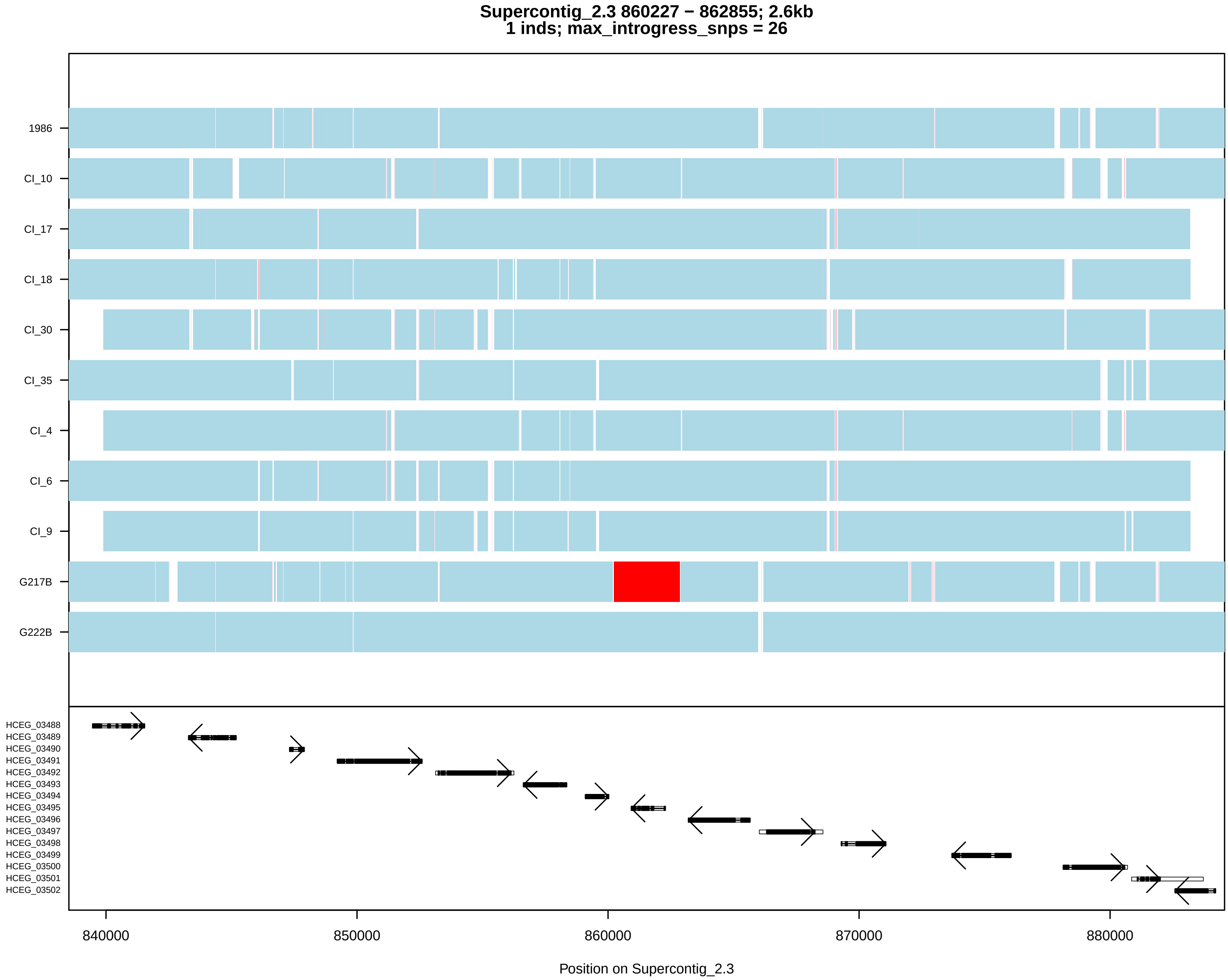
Supercontig_2.3 860227 − 862855; 2.6kb
1 inds; max_introgress_snps = 26
1986
CI_10
CI_17
CI_18
CI_30
CI_35
CI_4
CI_6
CI_9
G217B
G222B
HCEG_03488
HCEG_03489
HCEG_03490
HCEG_03491
HCEG_03492
HCEG_03493
HCEG_03494
HCEG_03495
HCEG_03496
HCEG_03497
HCEG_03498
HCEG_03499
HCEG_03500
HCEG_03501
HCEG_03502
840000
850000
860000
870000
880000
Position on Supercontig_2.3

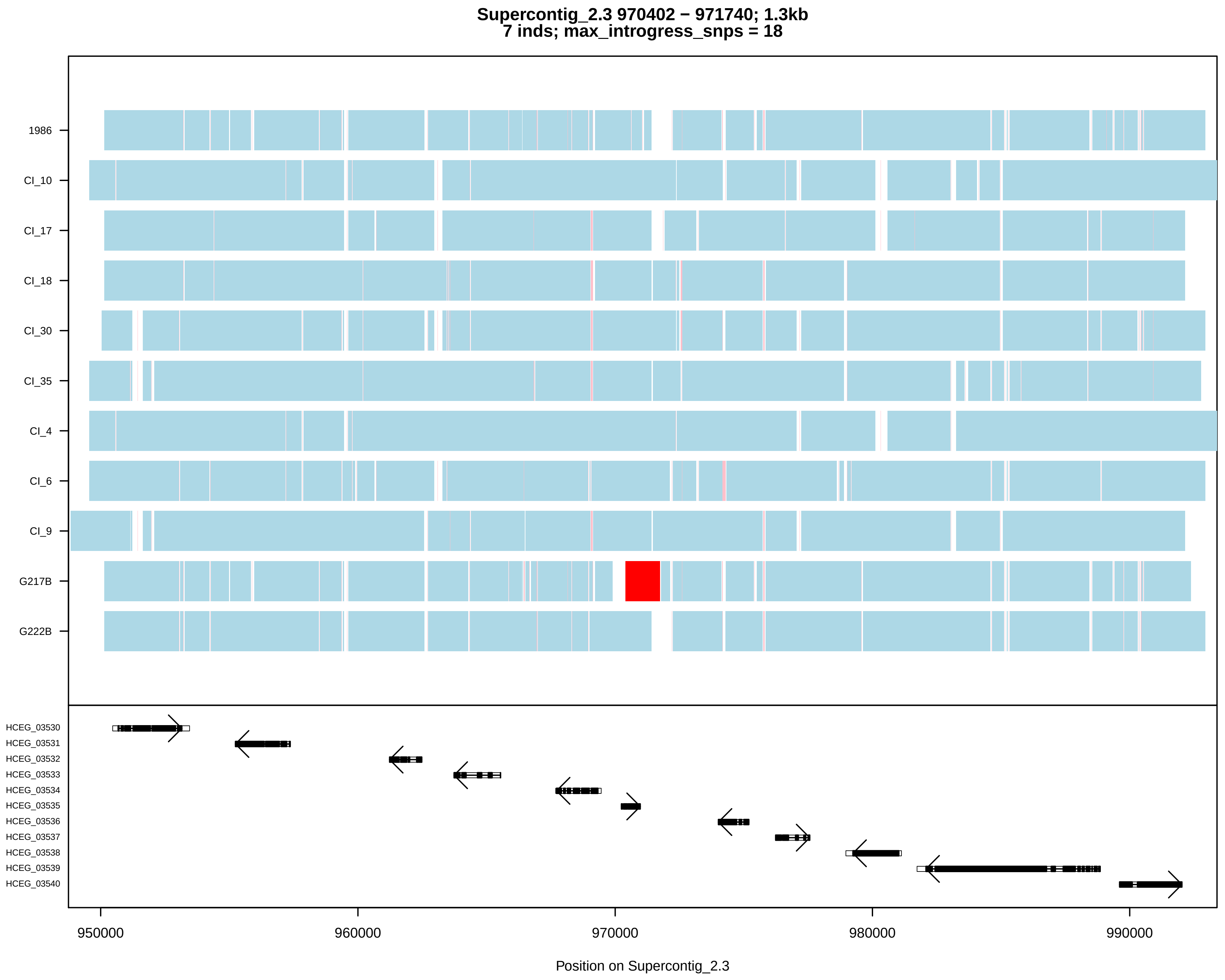
Supercontig_2.3 970402 − 971740; 1.3kb
7 inds; max_introgress_snps = 18
1986
CI_10
CI_17
CI_18
CI_30
CI_35
CI_4
CI_6
CI_9
G217B
G222B
HCEG_03530
HCEG_03531
HCEG_03532
HCEG_03533
HCEG_03534
HCEG_03535
HCEG_03536
HCEG_03537
HCEG_03538
HCEG_03539
HCEG_03540
950000
960000
970000
980000
990000
Position on Supercontig_2.3

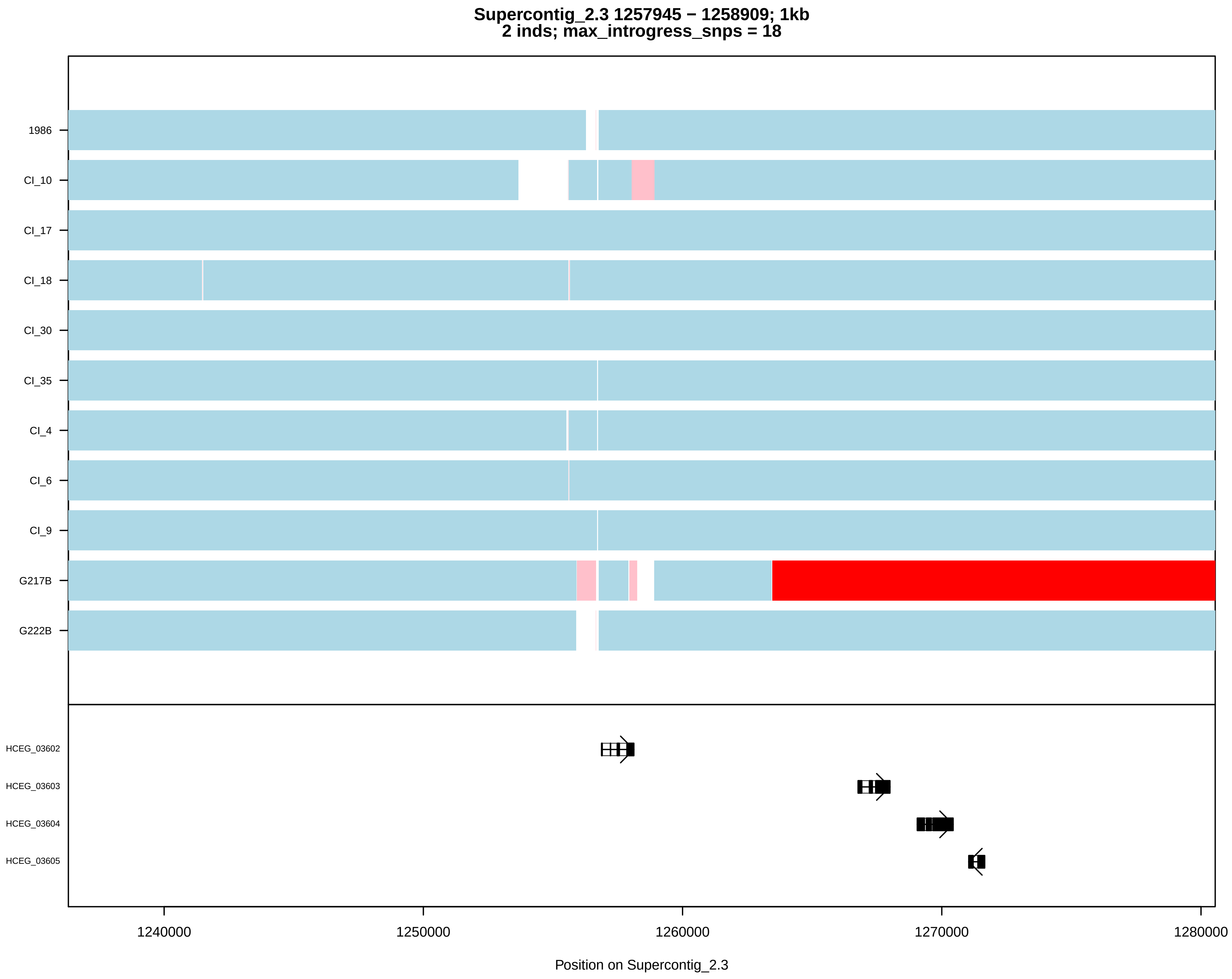

Supercontig_2.3 1257945 − 1258909; 1kb
2 inds; max_introgress_snps = 18
1986
CI_10
CI_17
CI_18
CI_30
CI_35
CI_4
CI_6
CI_9
G217B
G222B
HCEG_03602
HCEG_03603
HCEG_03604
HCEG_03605
1240000
1250000
1260000
1270000
1280000
Position on Supercontig_2.3

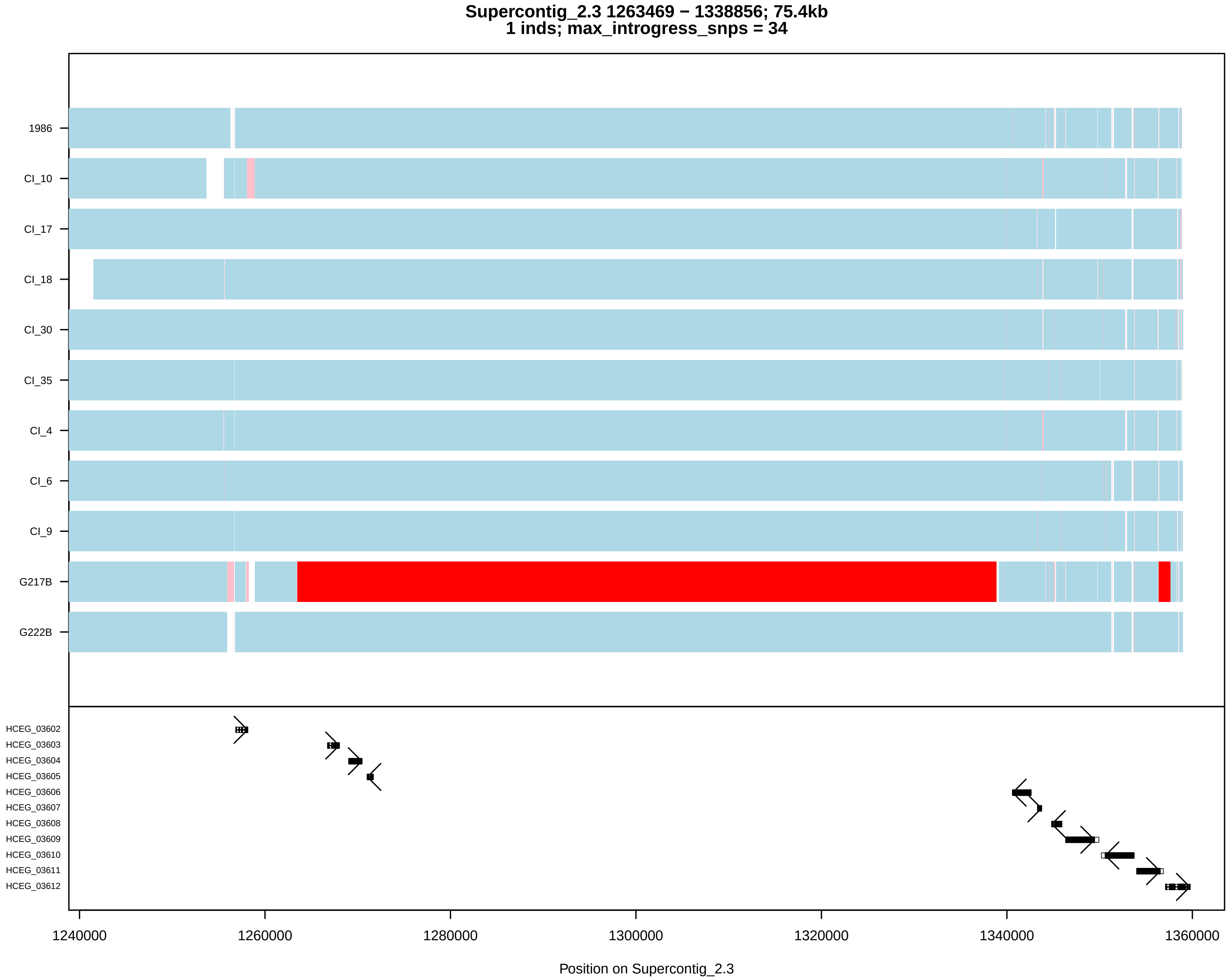
Supercontig_2.3 1263469 − 1338856; 75.4kb
1 inds; max_introgress_snps = 34
1986
CI_10
CI_17
CI_18
CI_30
CI_35
CI_4
CI_6
CI_9
G217B
G222B
HCEG_03602
HCEG_03603
HCEG_03604
HCEG_03605
HCEG_03606
HCEG_03607
HCEG_03608
HCEG_03609
HCEG_03610
HCEG_03611
HCEG_03612
1240000
1260000
1280000
1300000
1320000
1340000
1360000
Position on Supercontig_2.3

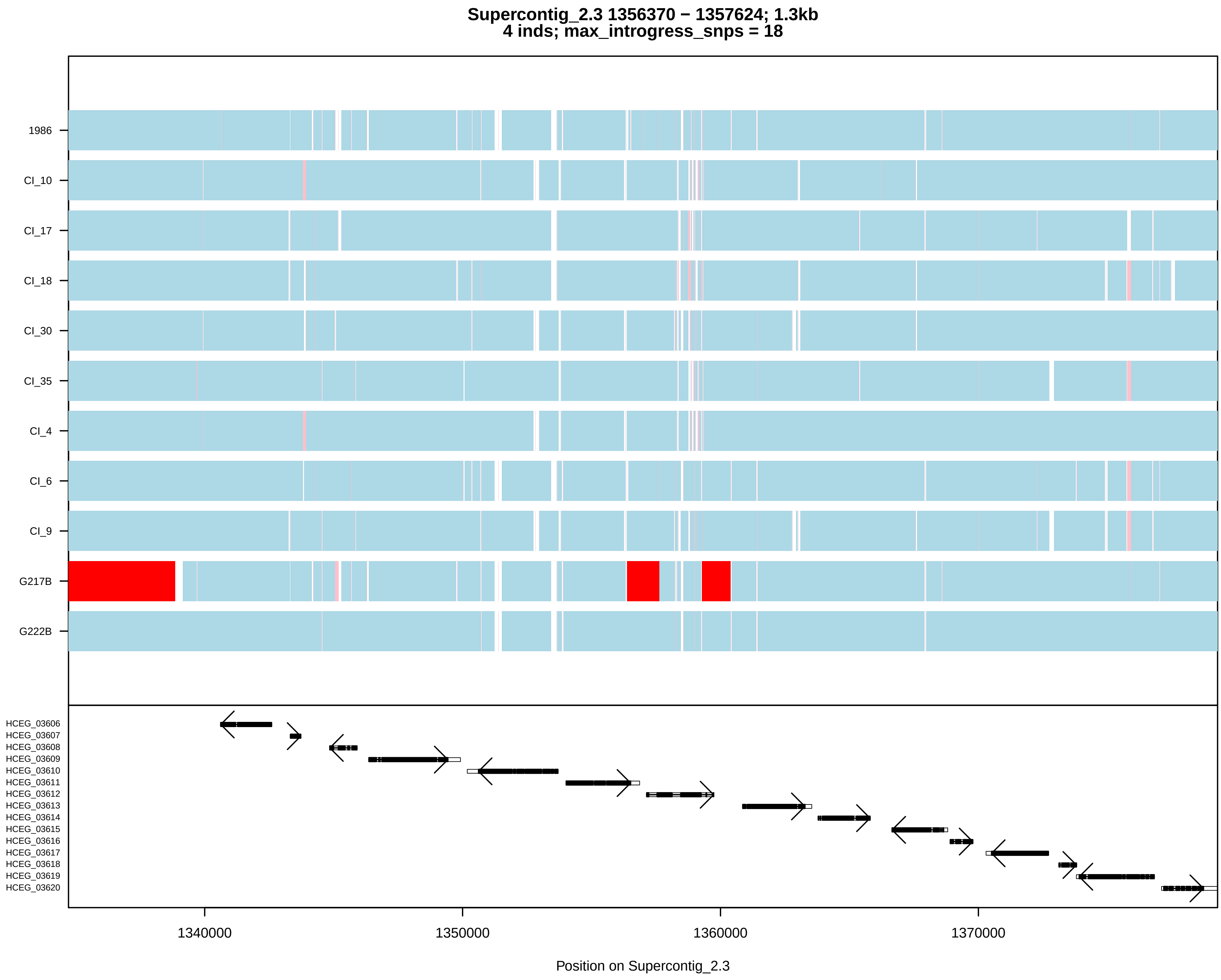
Supercontig_2.3 1356370 − 1357624; 1.3kb
4 inds; max_introgress_snps = 18
1986
CI_10
CI_17
CI_18
CI_30
CI_35
CI_4
CI_6
CI_9
G217B
G222B
HCEG_03606
HCEG_03607
HCEG_03608
HCEG_03609
HCEG_03610
HCEG_03611
HCEG_03612
HCEG_03613
HCEG_03614
HCEG_03615
HCEG_03616
HCEG_03617
HCEG_03618
HCEG_03619
HCEG_03620
1340000
1350000
1360000
1370000
Position on Supercontig_2.3

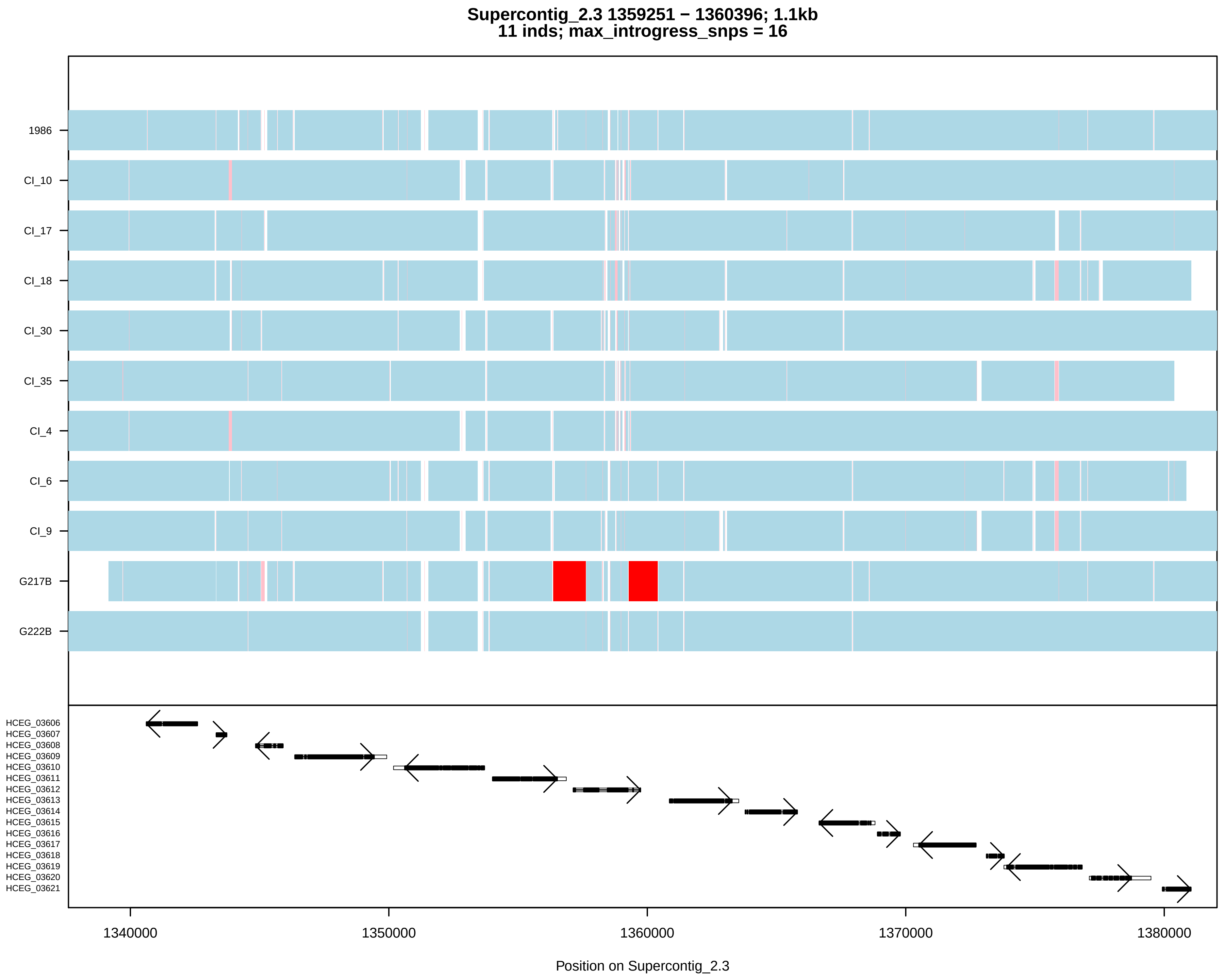

Supercontig_2.3 1359251 − 1360396; 1.1kb
11 inds; max_introgress_snps = 16
1986
CI_10
CI_17
CI_18
CI_30
CI_35
CI_4
CI_6
CI_9
G217B
G222B
HCEG_03606
HCEG_03607
HCEG_03608
HCEG_03609
HCEG_03610
HCEG_03611
HCEG_03612
HCEG_03613
HCEG_03614
HCEG_03615
HCEG_03616
HCEG_03617
HCEG_03618
HCEG_03619
HCEG_03620
HCEG_03621
1340000
1350000
1360000
1370000
1380000
Position on Supercontig_2.3

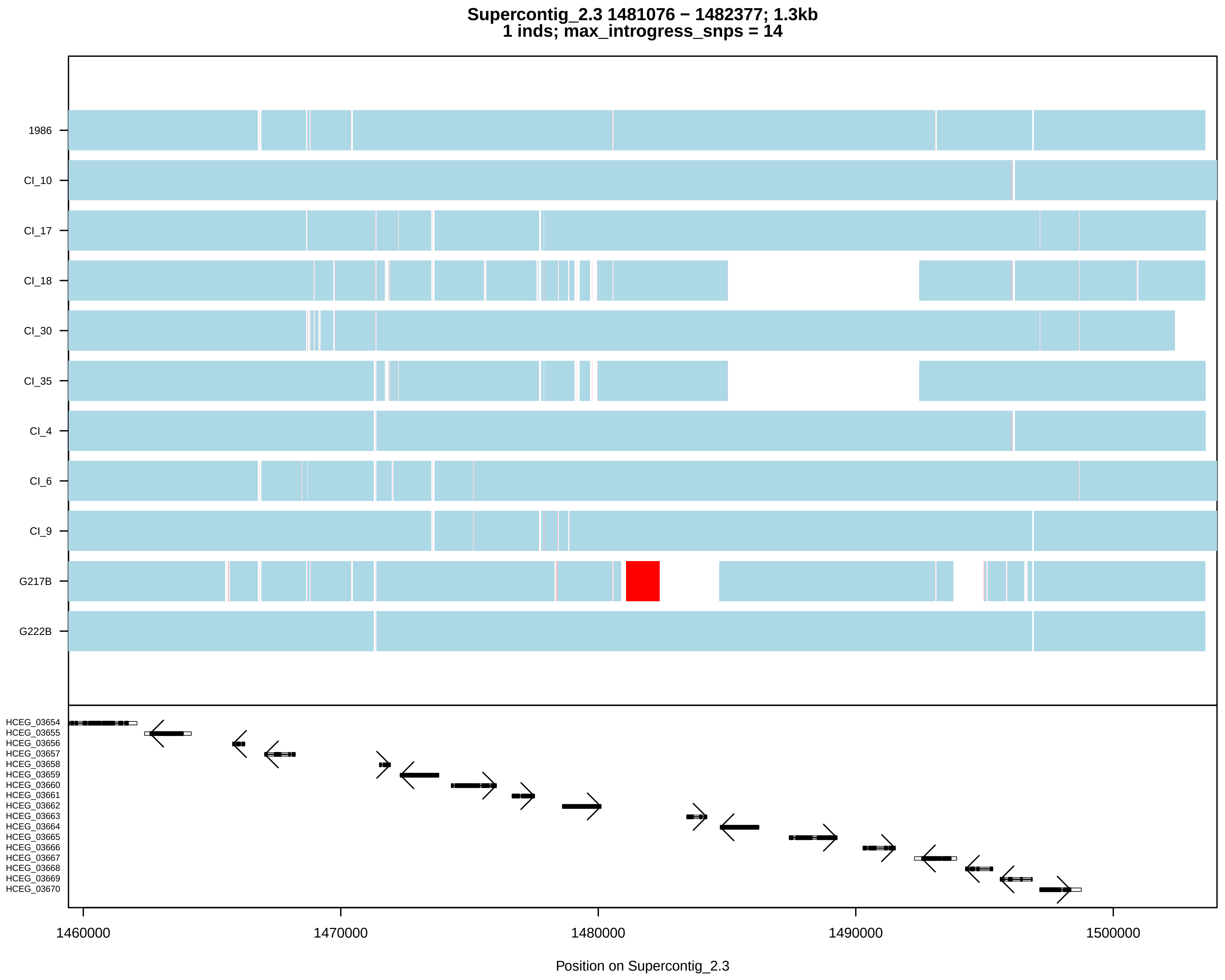

Supercontig_2.3 1481076 − 1482377; 1.3kb
1 inds; max_introgress_snps = 14
1986
CI_10
CI_17
CI_18
CI_30
CI_35
CI_4
CI_6
CI_9
G217B
G222B
HCEG_03654
HCEG_03655
HCEG_03656
HCEG_03657
HCEG_03658
HCEG_03659
HCEG_03660
HCEG_03661
HCEG_03662
HCEG_03663
HCEG_03664
HCEG_03665
HCEG_03666
HCEG_03667
HCEG_03668
HCEG_03669
HCEG_03670
1460000
1470000
1480000
1490000
1500000
Position on Supercontig_2.3

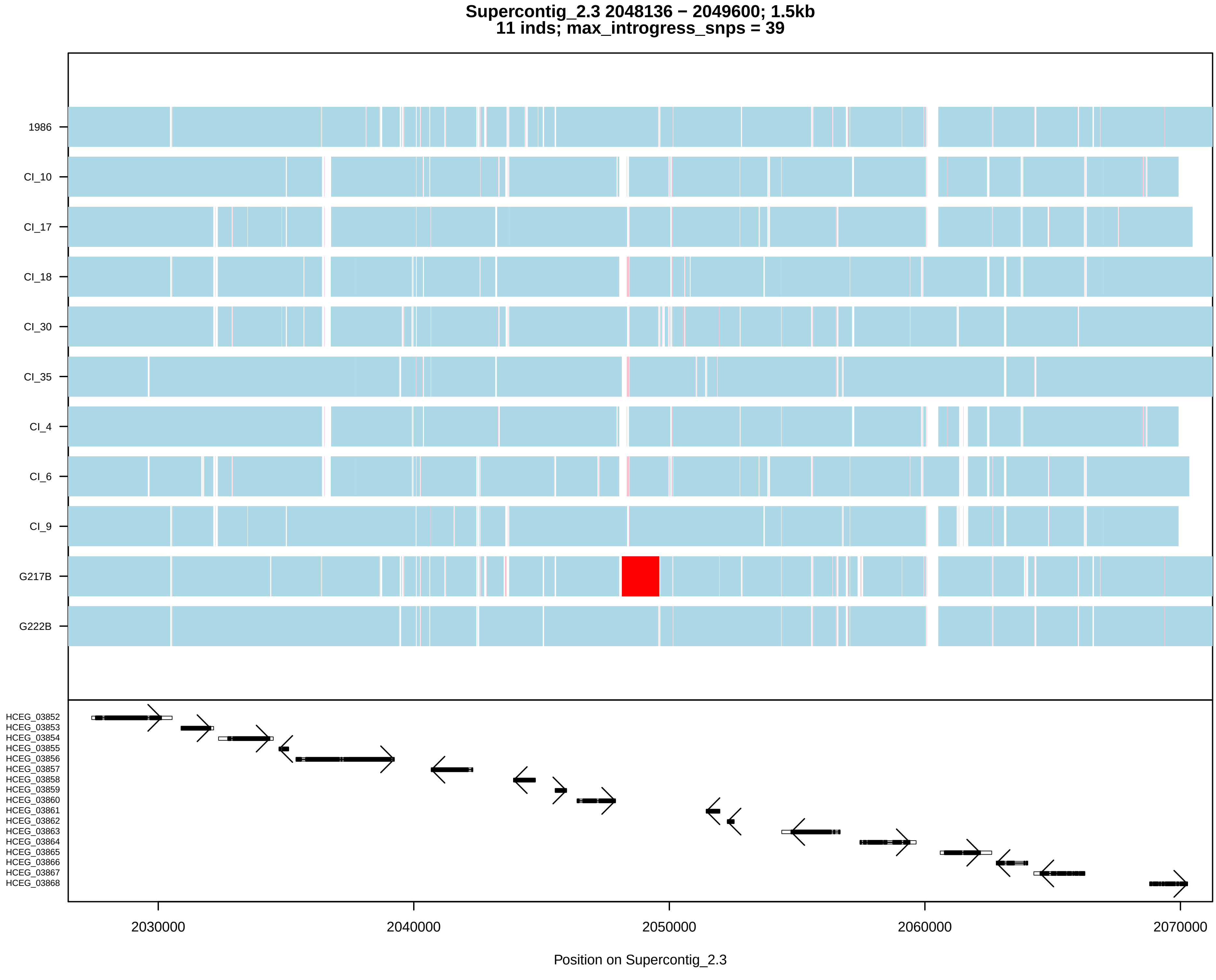

Supercontig_2.3 2048136 − 2049600; 1.5kb
11 inds; max_introgress_snps = 39
1986
CI_10
CI_17
CI_18
CI_30
CI_35
CI_4
CI_6
CI_9
G217B
G222B
HCEG_03852
HCEG_03853
HCEG_03854
HCEG_03855
HCEG_03856
HCEG_03857
HCEG_03858
HCEG_03859
HCEG_03860
HCEG_03861
HCEG_03862
HCEG_03863
HCEG_03864
HCEG_03865
HCEG_03866
HCEG_03867
HCEG_03868
2030000
2040000
2050000
2060000
2070000
Position on Supercontig_2.3

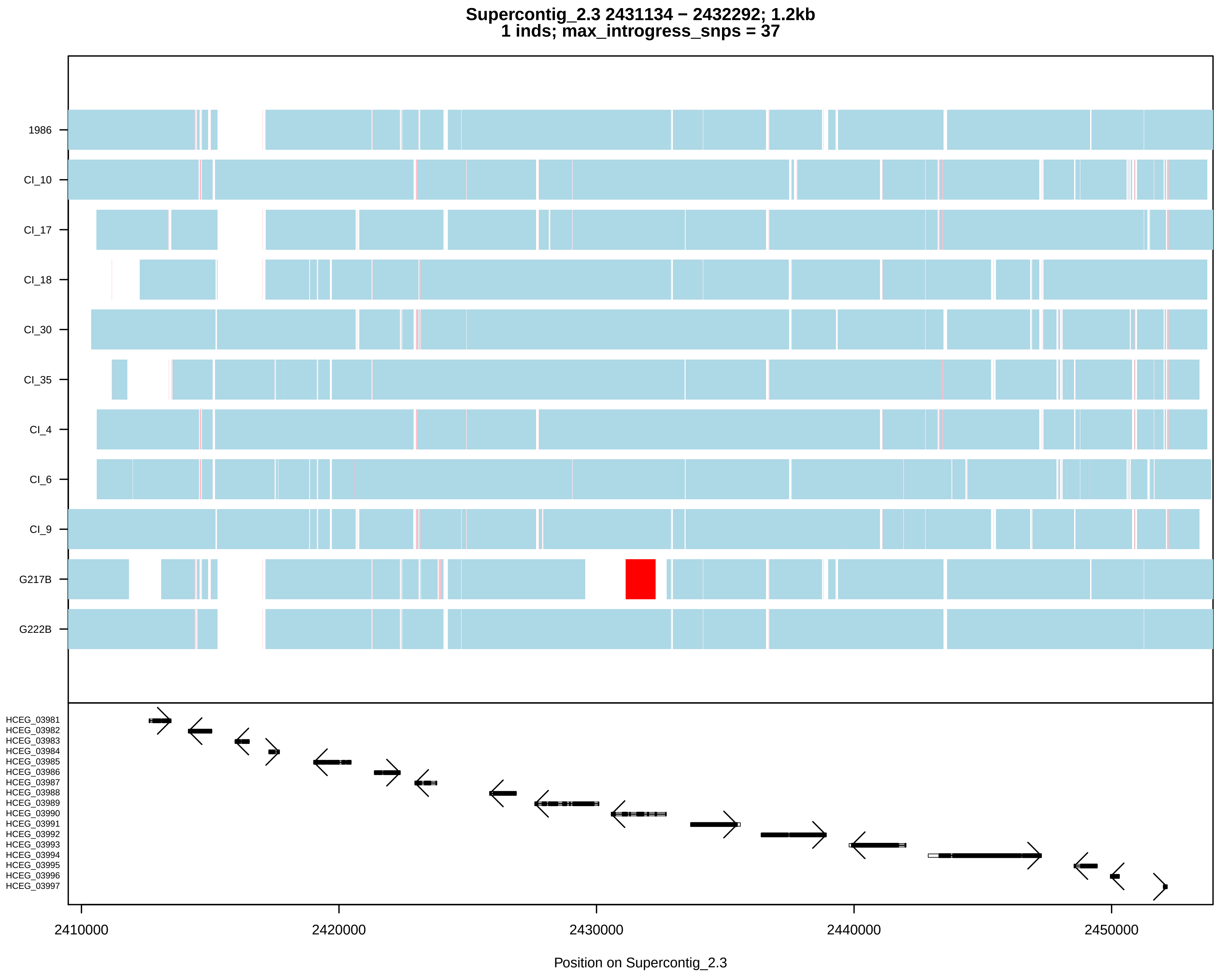
Supercontig_2.3 2431134 − 2432292; 1.2kb
1 inds; max_introgress_snps = 37
1986
CI_10
CI_17
CI_18
CI_30
CI_35
CI_4
CI_6
CI_9
G217B
G222B
HCEG_03981
HCEG_03982
HCEG_03983
HCEG_03984
HCEG_03985
HCEG_03986
HCEG_03987
HCEG_03988
HCEG_03989
HCEG_03990
HCEG_03991
HCEG_03992
HCEG_03993
HCEG_03994
HCEG_03995
HCEG_03996
HCEG_03997
2410000
2420000
2430000
2440000
2450000
Position on Supercontig_2.3

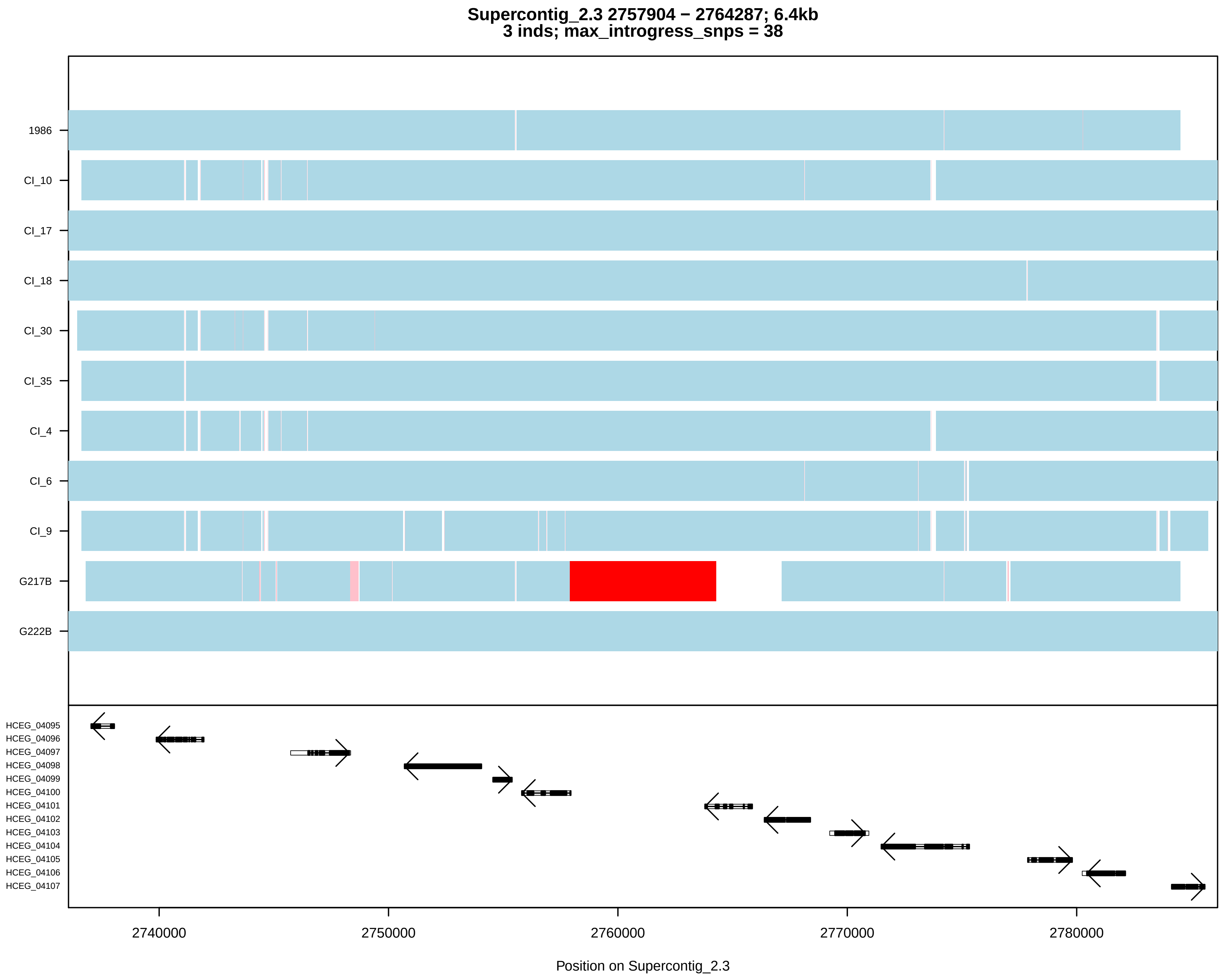

Supercontig_2.3 2757904 − 2764287; 6.4kb
3 inds; max_introgress_snps = 38
1986
CI_10
CI_17
CI_18
CI_30
CI_35
CI_4
CI_6
CI_9
G217B
G222B
HCEG_04095
HCEG_04096
HCEG_04097
HCEG_04098
HCEG_04099
HCEG_04100
HCEG_04101
HCEG_04102
HCEG_04103
HCEG_04104
HCEG_04105
HCEG_04106
HCEG_04107
2740000
2750000
2760000
2770000
2780000
Position on Supercontig_2.3

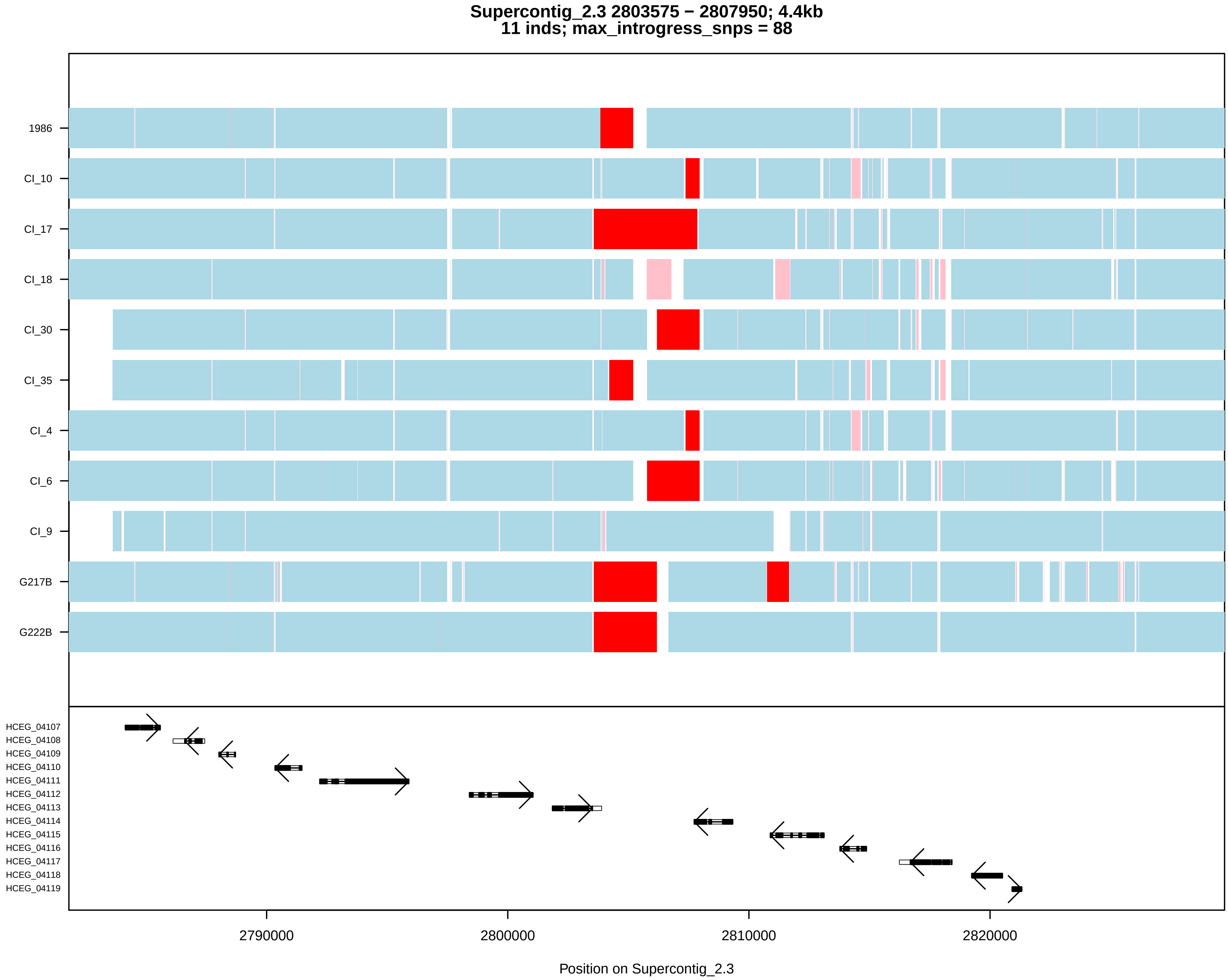
Supercontig_2.3 2803575 − 2807950; 4.4kb
11 inds; max_introgress_snps = 88
1986
CI_10
CI_17
CI_18
CI_30
CI_35
CI_4
CI_6
CI_9
G217B
G222B
HCEG_04107
HCEG_04108
HCEG_04109
HCEG_04110
HCEG_04111
HCEG_04112
HCEG_04113
HCEG_04114
HCEG_04115
HCEG_04116
HCEG_04117
HCEG_04118
HCEG_04119
2790000
2800000
2810000
2820000
Position on Supercontig_2.3

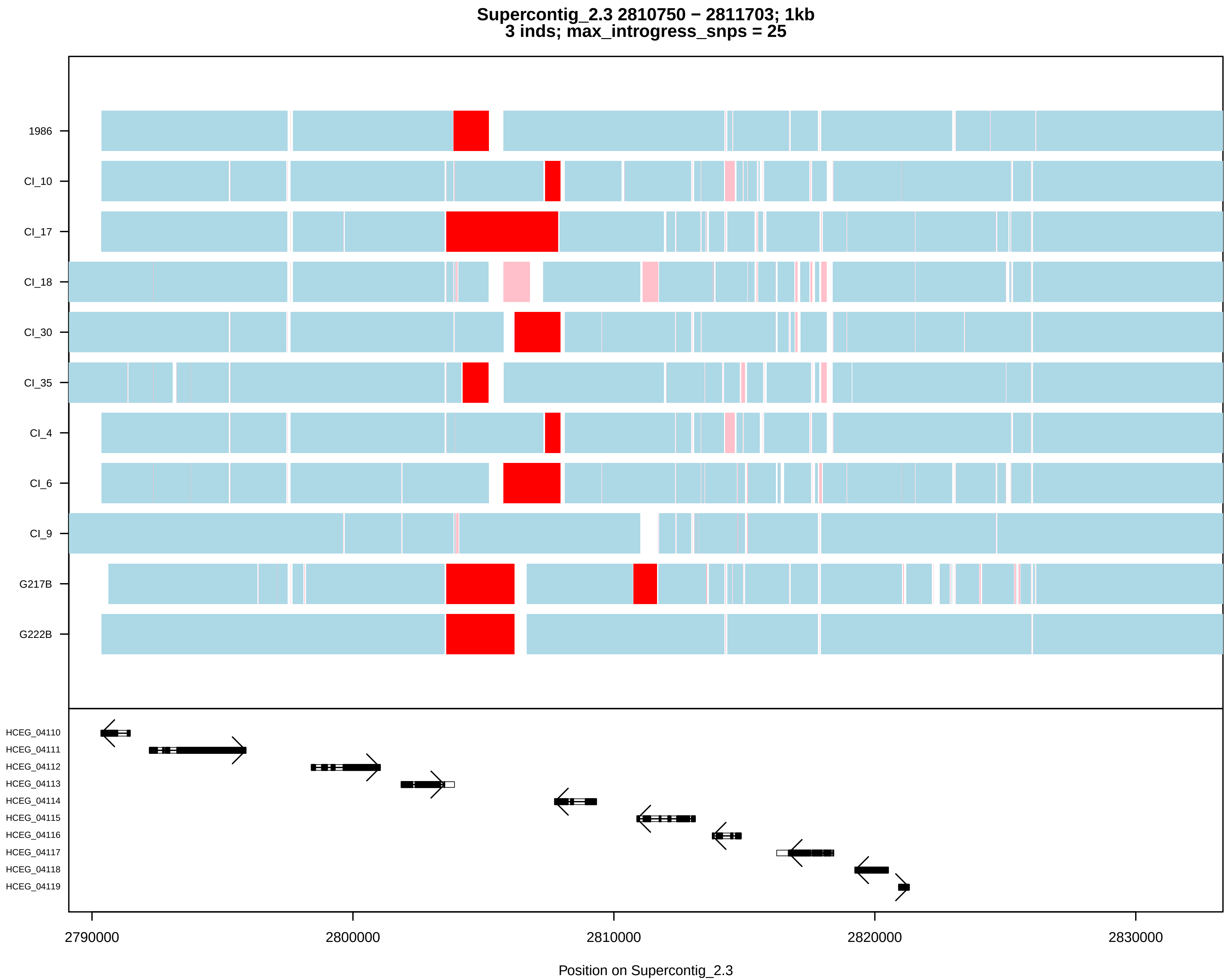
Supercontig_2.3 2810750 − 2811703; 1kb
3 inds; max_introgress_snps = 25
1986
CI_10
CI_17
CI_18
CI_30
CI_35
CI_4
CI_6
CI_9
G217B
G222B
HCEG_04110
HCEG_04111
HCEG_04112
HCEG_04113
HCEG_04114
HCEG_04115
HCEG_04116
HCEG_04117
HCEG_04118
HCEG_04119
2790000
2800000
2810000
2820000
2830000
Position on Supercontig_2.3

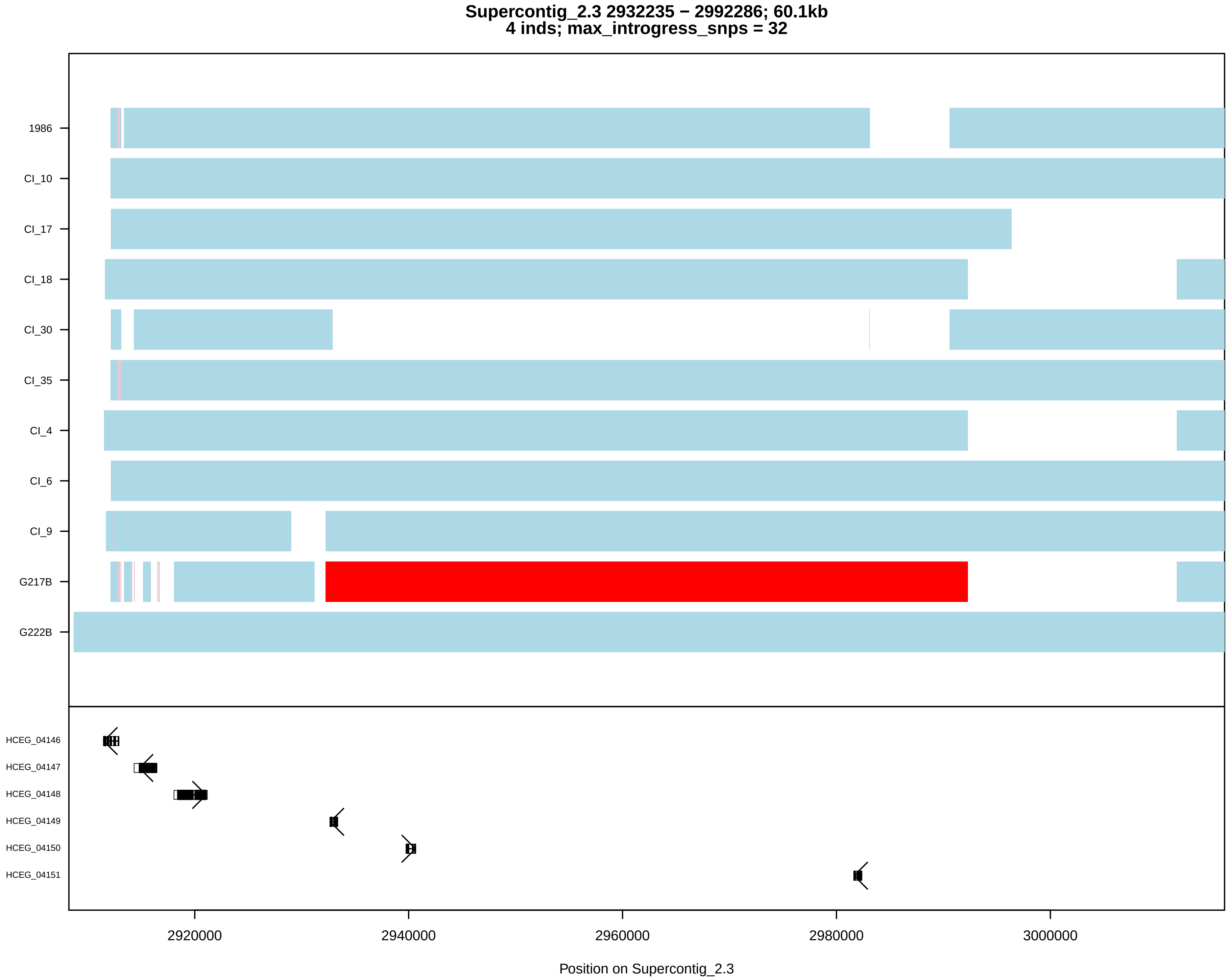

Supercontig_2.3 2932235 − 2992286; 60.1kb
4 inds; max_introgress_snps = 32
1986
CI_10
CI_17
CI_18
CI_30
CI_35
CI_4
CI_6
CI_9
G217B
G222B
HCEG_04146
HCEG_04147
HCEG_04148
HCEG_04149
HCEG_04150
HCEG_04151
2920000
2940000
2960000
2980000
3000000
Position on Supercontig_2.3

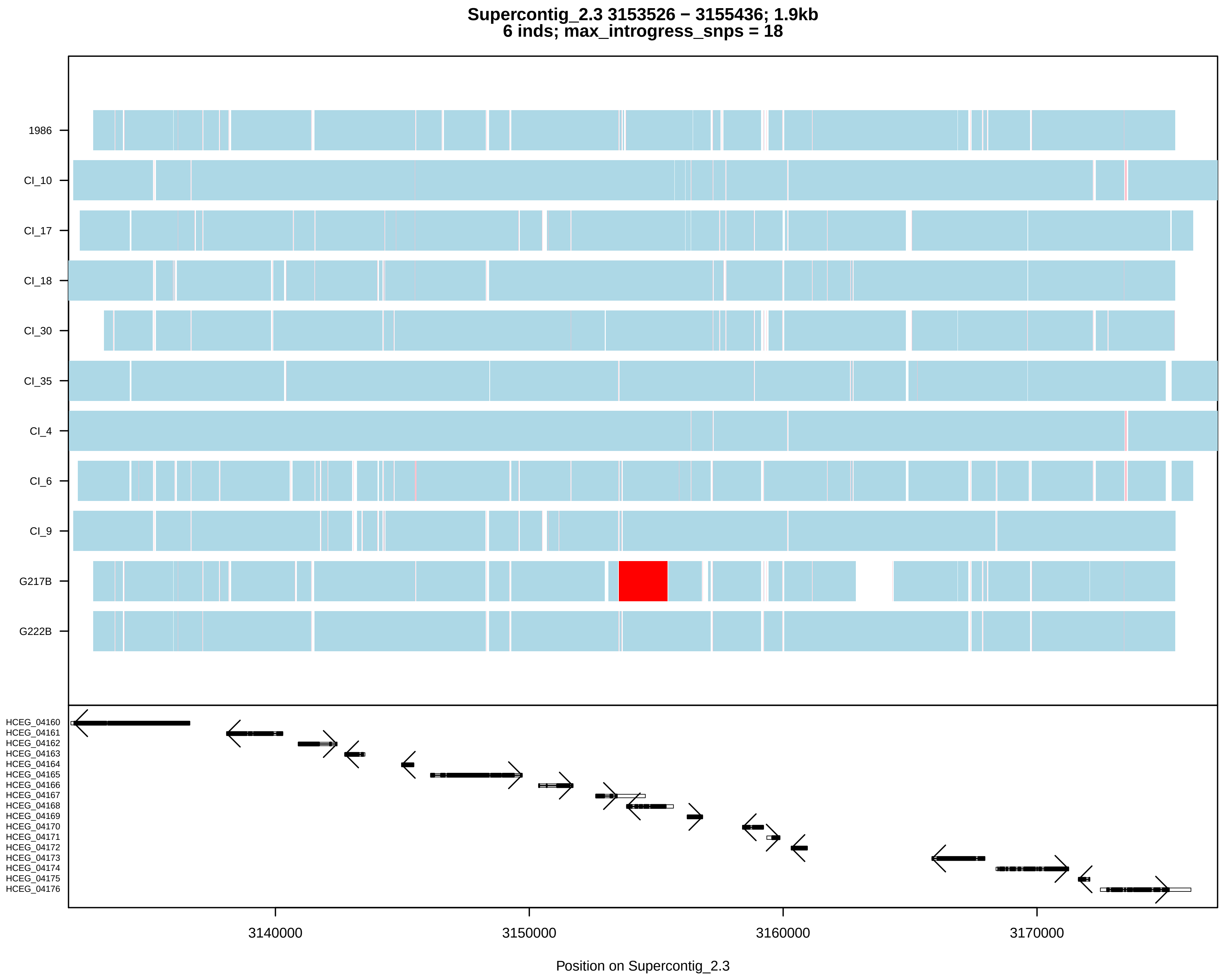
Supercontig_2.3 3153526 − 3155436; 1.9kb
6 inds; max_introgress_snps = 18
1986
CI_10
CI_17
CI_18
CI_30
CI_35
CI_4
CI_6
CI_9
G217B
G222B
HCEG_04160
HCEG_04161
HCEG_04162
HCEG_04163
HCEG_04164
HCEG_04165
HCEG_04166
HCEG_04167
HCEG_04168
HCEG_04169
HCEG_04170
HCEG_04171
HCEG_04172
HCEG_04173
HCEG_04174
HCEG_04175
HCEG_04176
3140000
3150000
3160000
3170000
Position on Supercontig_2.3

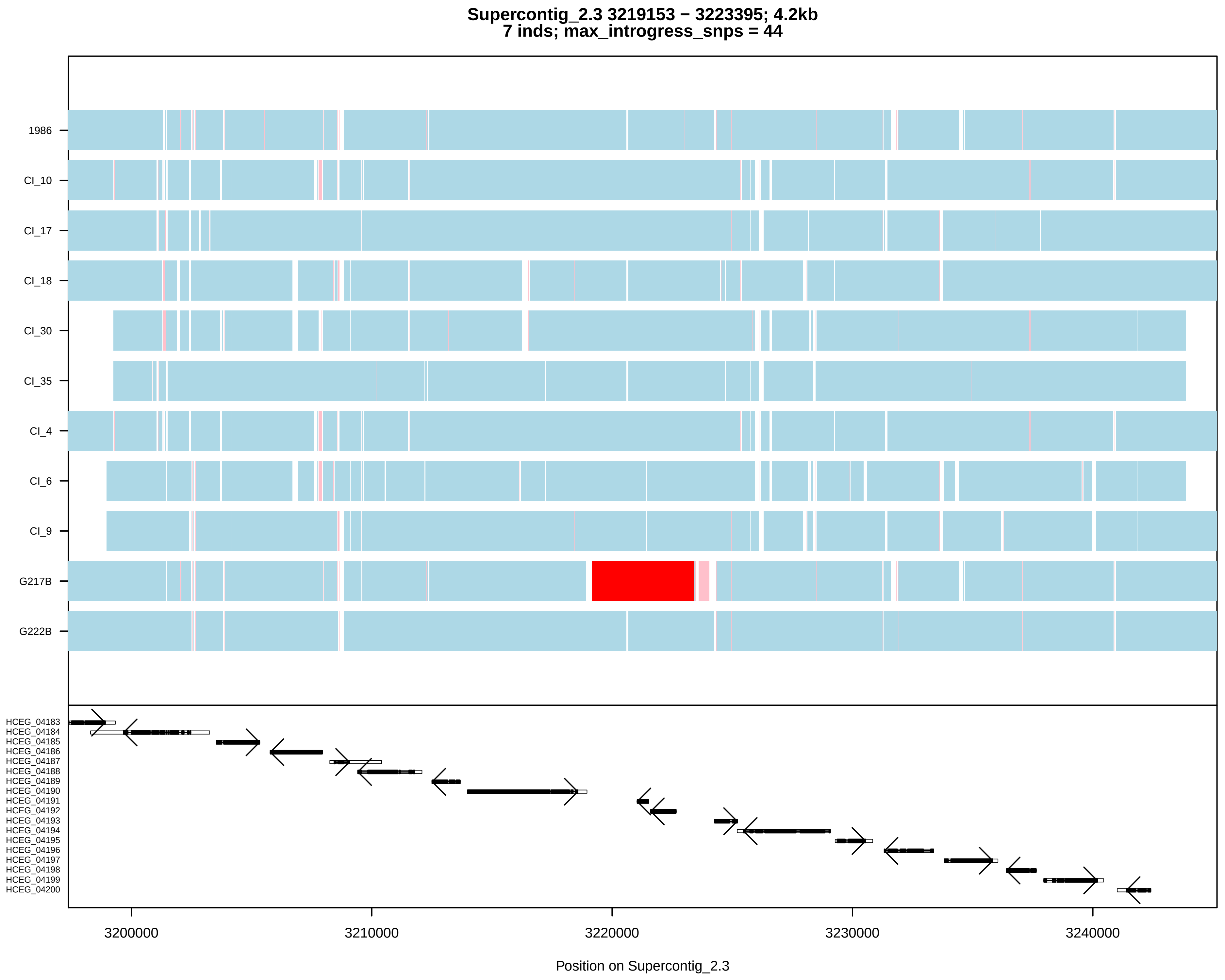
Supercontig_2.3 3219153 − 3223395; 4.2kb
7 inds; max_introgress_snps = 44
1986
CI_10
CI_17
CI_18
CI_30
CI_35
CI_4
CI_6
CI_9
G217B
G222B
HCEG_04183
HCEG_04184
HCEG_04185
HCEG_04186
HCEG_04187
HCEG_04188
HCEG_04189
HCEG_04190
HCEG_04191
HCEG_04192
HCEG_04193
HCEG_04194
HCEG_04195
HCEG_04196
HCEG_04197
HCEG_04198
HCEG_04199
HCEG_04200
3200000
3210000
3220000
3230000
3240000
Position on Supercontig_2.3

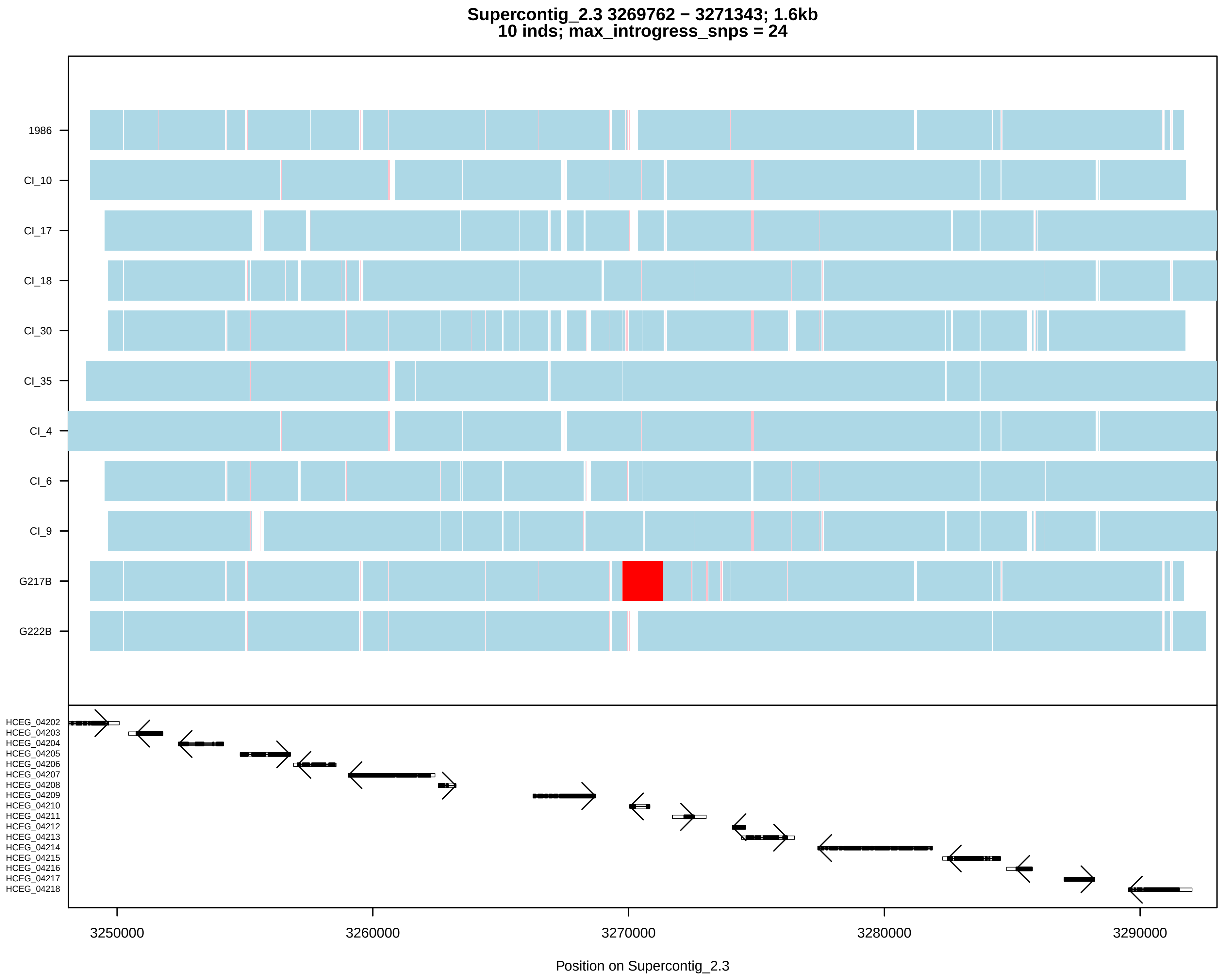

Supercontig_2.3 3269762 − 3271343; 1.6kb
10 inds; max_introgress_snps = 24
1986
CI_10
CI_17
CI_18
CI_30
CI_35
CI_4
CI_6
CI_9
G217B
G222B
HCEG_04202
HCEG_04203
HCEG_04204
HCEG_04205
HCEG_04206
HCEG_04207
HCEG_04208
HCEG_04209
HCEG_04210
HCEG_04211
HCEG_04212
HCEG_04213
HCEG_04214
HCEG_04215
HCEG_04216
HCEG_04217
HCEG_04218
3250000
3260000
3270000
3280000
3290000
Position on Supercontig_2.3

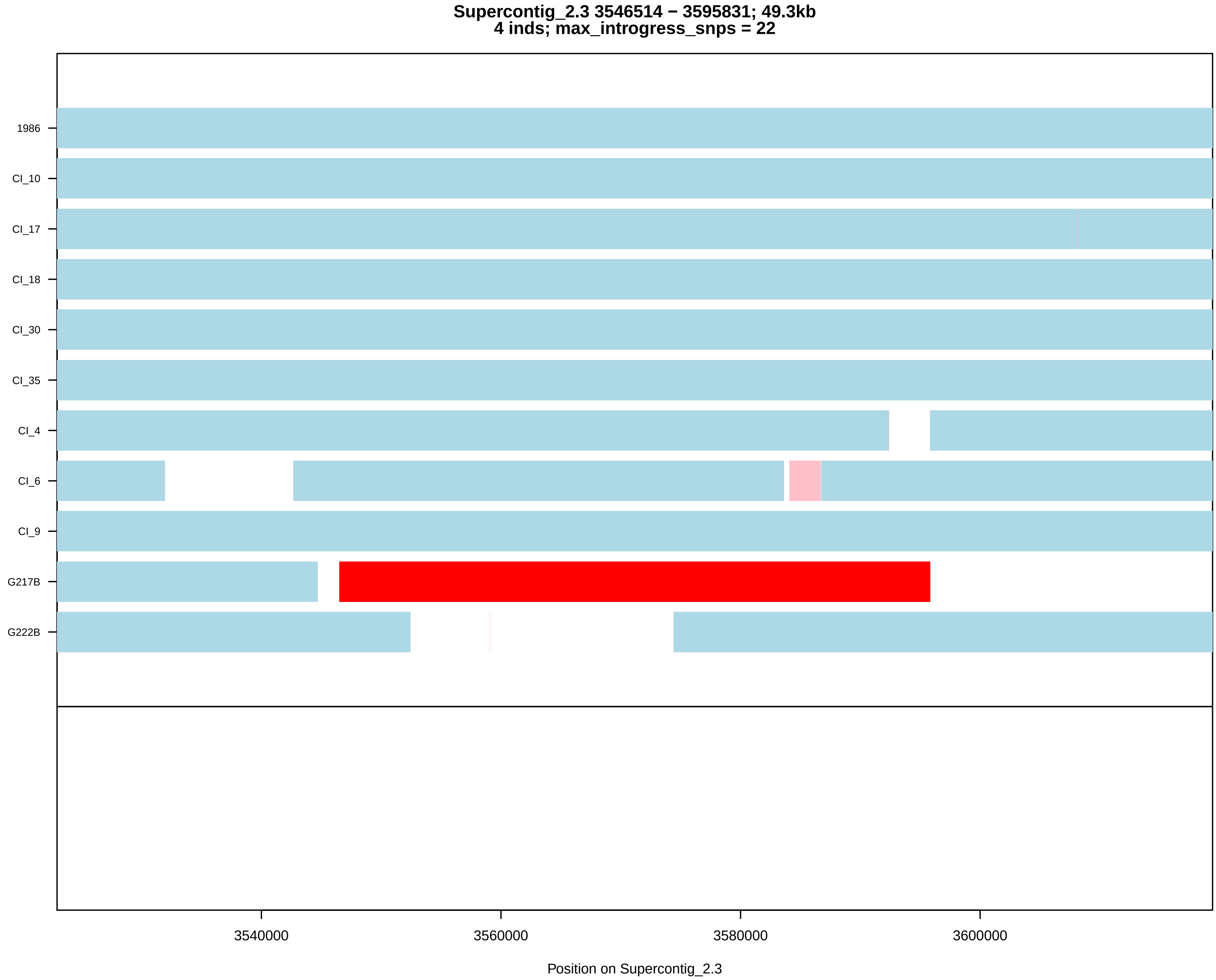

Supercontig_2.3 3546514 − 3595831; 49.3kb
4 inds; max_introgress_snps = 22
1986
CI_10
CI_17
CI_18
CI_30
CI_35
CI_4
CI_6
CI_9
G217B
G222B
3540000
3560000
3580000
3600000
Position on Supercontig_2.3

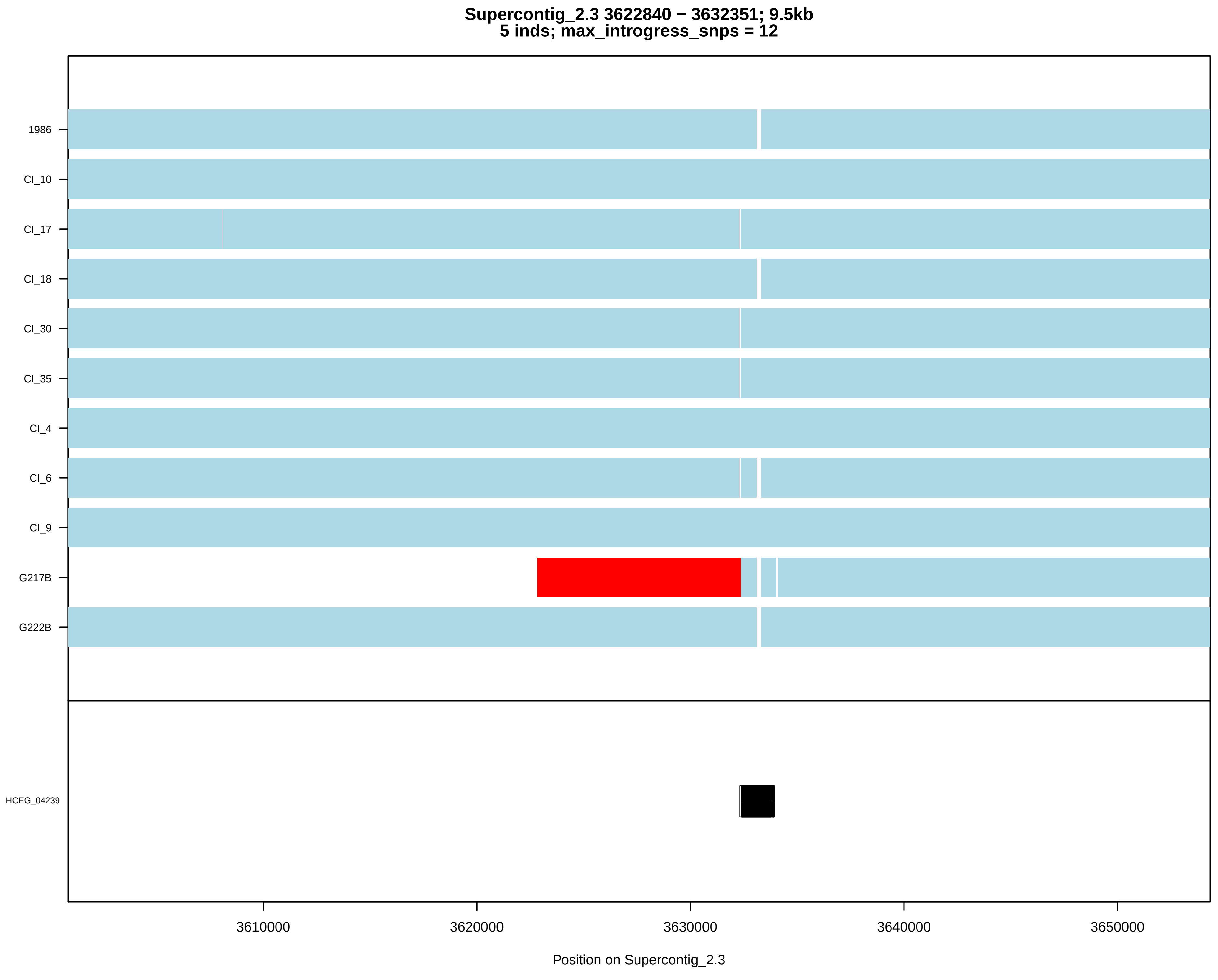
Supercontig_2.3 3622840 − 3632351; 9.5kb
5 inds; max_introgress_snps = 12
1986
CI_10
CI_17
CI_18
CI_30
CI_35
CI_4
CI_6
CI_9
G217B
G222B
HCEG_04239
3610000
3620000
3630000
3640000
3650000
Position on Supercontig_2.3

Supercontig_2.3 3756271 − 3771802; 15.5kb
9 inds; max_introgress_snps = 44

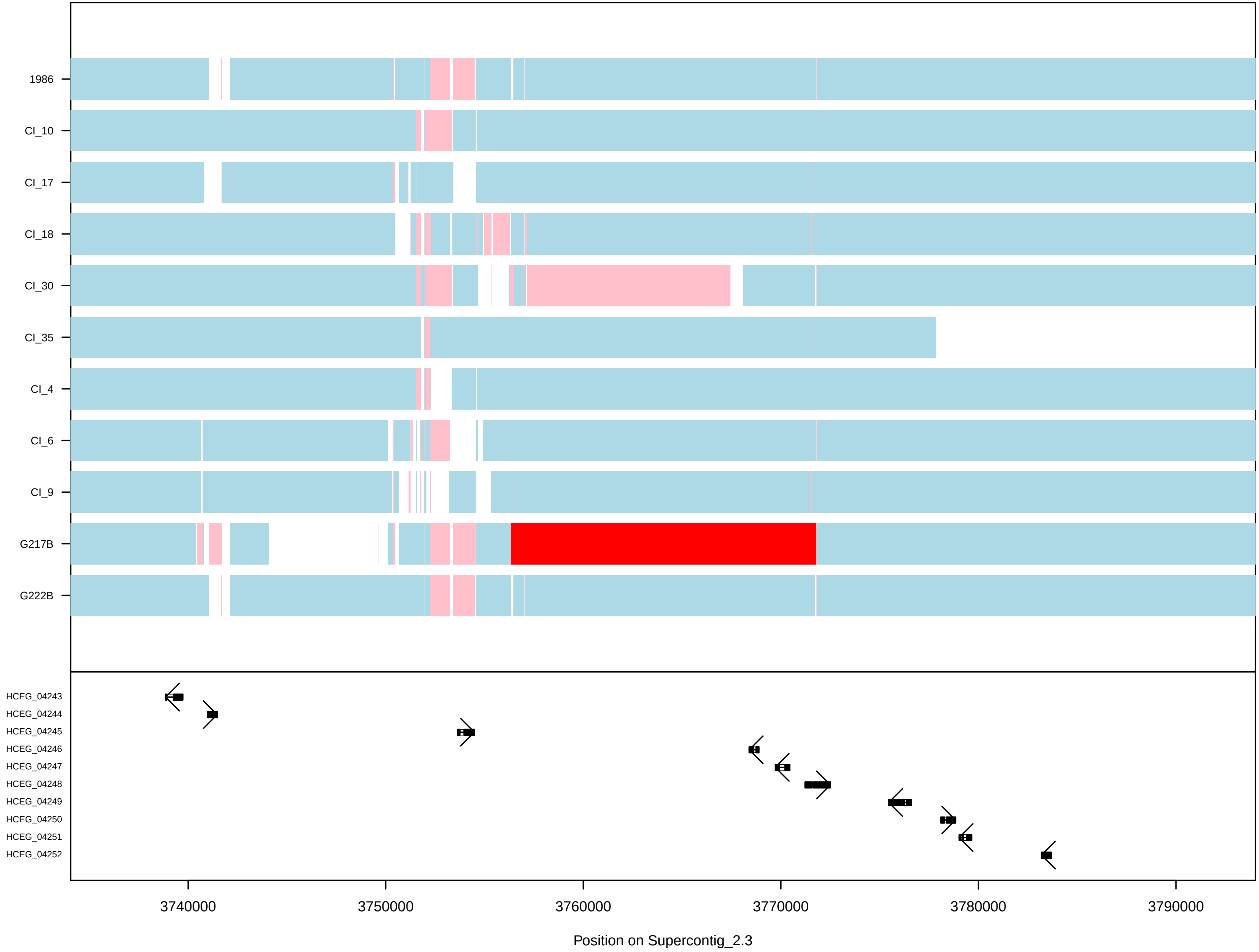

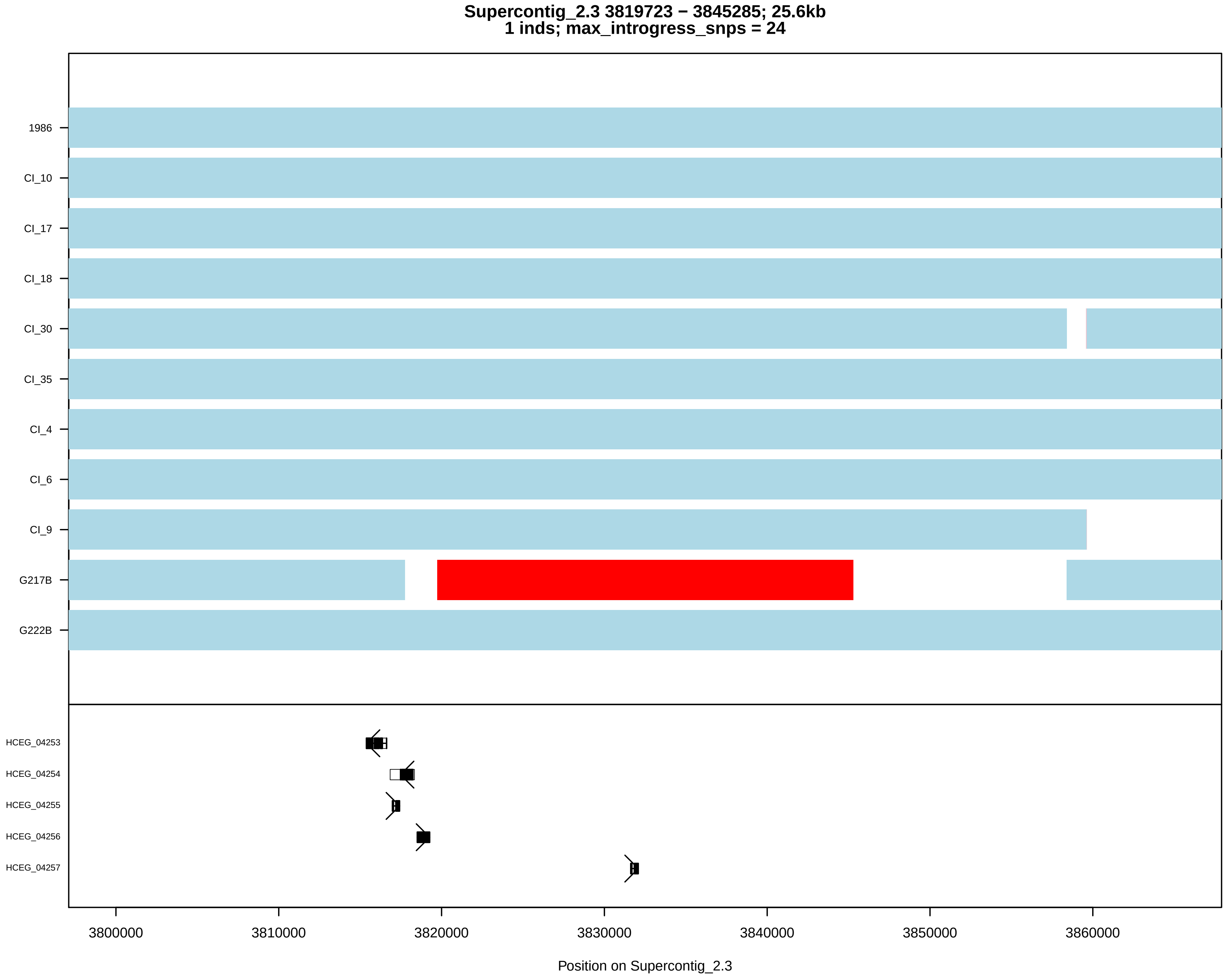
Supercontig_2.3 3819723 − 3845285; 25.6kb
1 inds; max_introgress_snps = 24
1986
CI_10
CI_17
CI_18
CI_30
CI_35
CI_4
CI_6
CI_9
G217B
G222B
HCEG_04253
HCEG_04254
HCEG_04255
HCEG_04256
HCEG_04257
3800000
3810000
3820000
3830000
3840000
3850000
3860000
Position on Supercontig_2.3

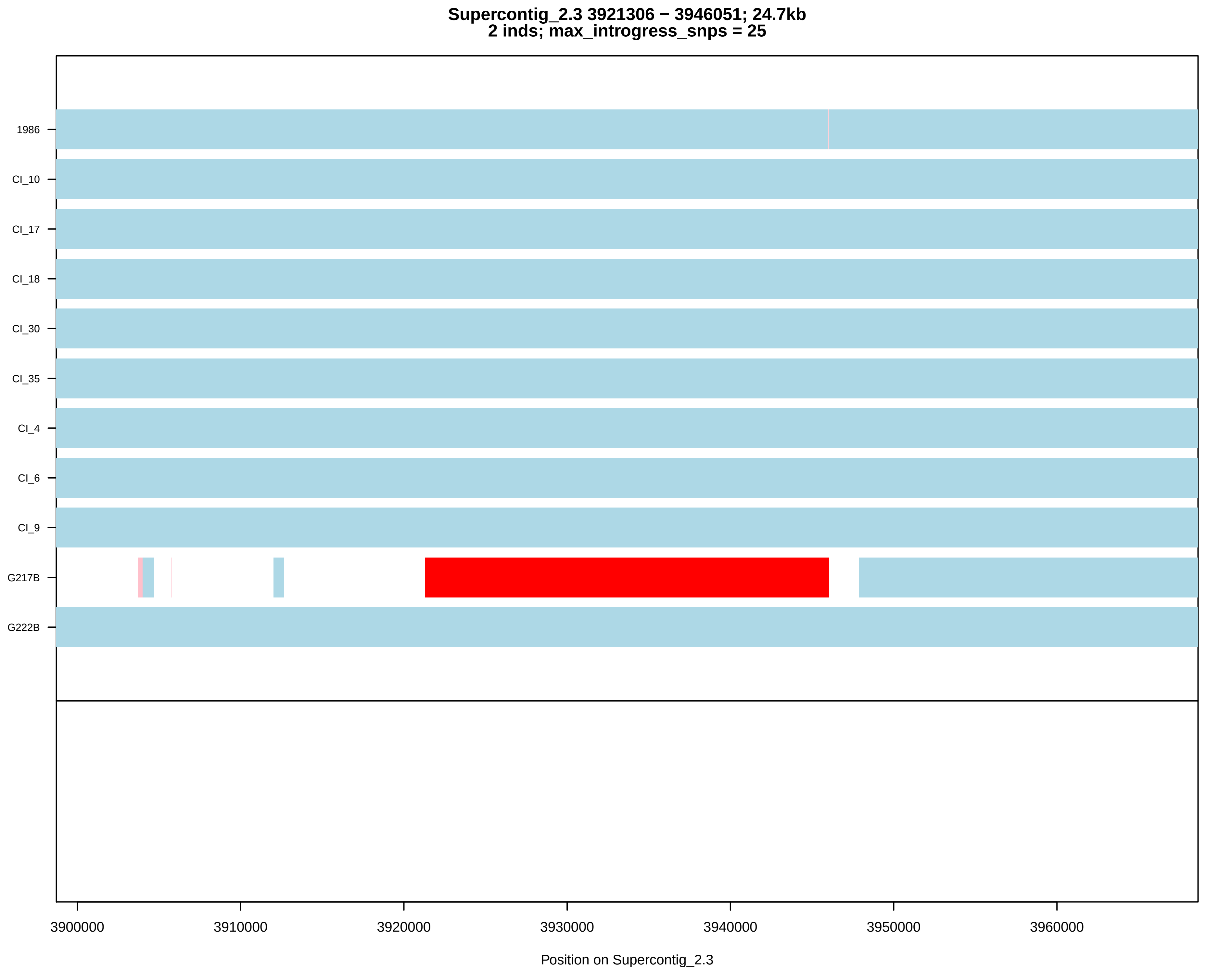
Supercontig_2.3 3921306 − 3946051; 24.7kb
2 inds; max_introgress_snps = 25
1986
CI_10
CI_17
CI_18
CI_30
CI_35
CI_4
CI_6
CI_9
G217B
G222B
3900000
3910000
3920000
3930000
3940000
3950000
3960000
Position on Supercontig_2.3

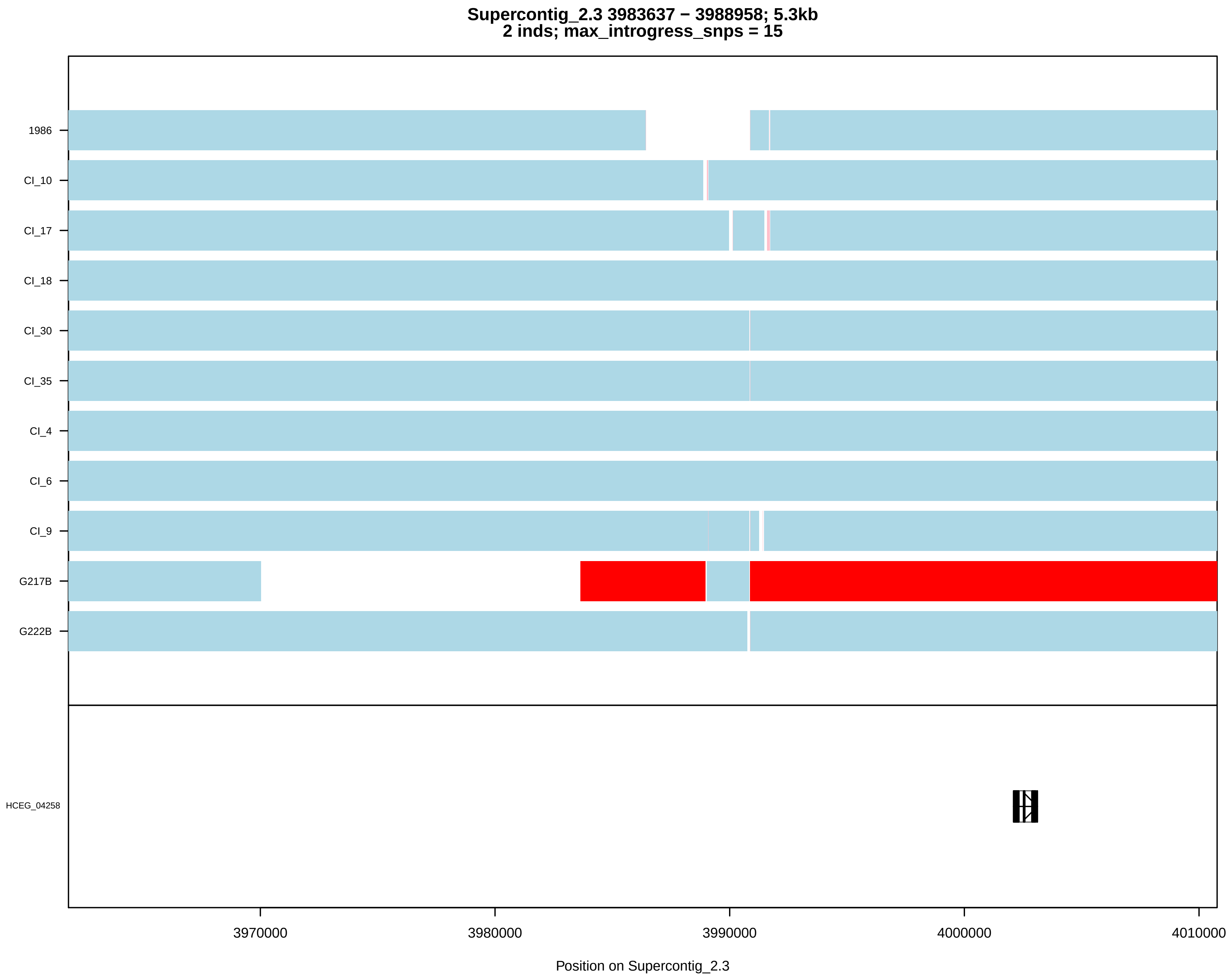

Supercontig_2.3 3983637 − 3988958; 5.3kb
2 inds; max_introgress_snps = 15
1986
CI_10
CI_17
CI_18
CI_30
CI_35
CI_4
CI_6
CI_9
G217B
G222B
HCEG_04258
3970000
3980000
3990000
4000000
4010000
Position on Supercontig_2.3

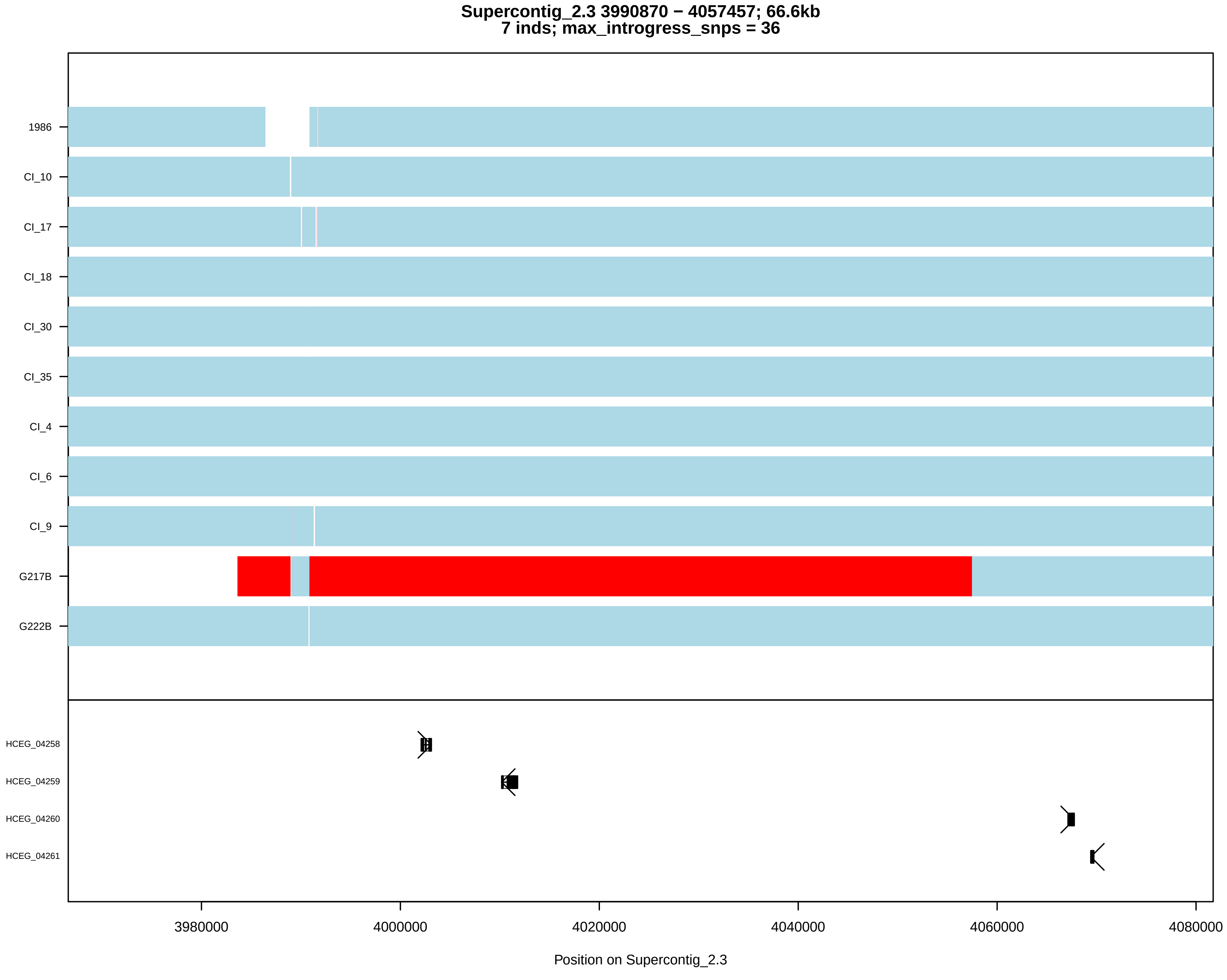
Supercontig_2.3 3990870 − 4057457; 66.6kb
7 inds; max_introgress_snps = 36
1986
CI_10
CI_17
CI_18
CI_30
CI_35
CI_4
CI_6
CI_9
G217B
G222B
HCEG_04258
HCEG_04259
HCEG_04260
HCEG_04261
3980000
4000000
4020000
4040000
4060000
4080000
Position on Supercontig_2.3

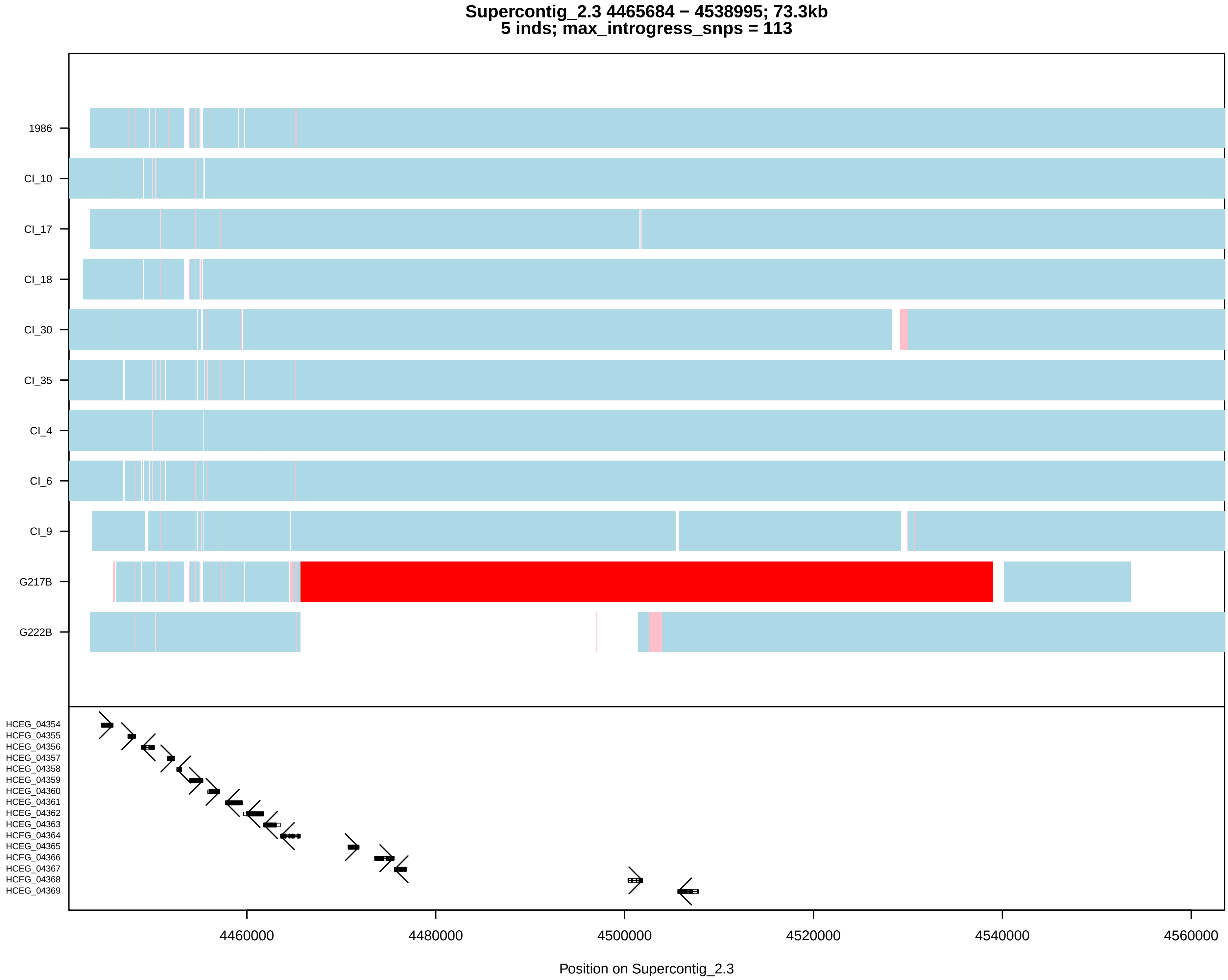
Supercontig_2.3 4465684 − 4538995; 73.3kb
5 inds; max_introgress_snps = 113
1986
CI_10
CI_17
CI_18
CI_30
CI_35
CI_4
CI_6
CI_9
G217B
G222B
HCEG_04354
HCEG_04355
HCEG_04356
HCEG_04357
HCEG_04358
HCEG_04359
HCEG_04360
HCEG_04361
HCEG_04362
HCEG_04363
HCEG_04364
HCEG_04365
HCEG_04366
HCEG_04367
HCEG_04368
HCEG_04369
4460000
4480000
4500000
4520000
4540000
4560000
Position on Supercontig_2.3

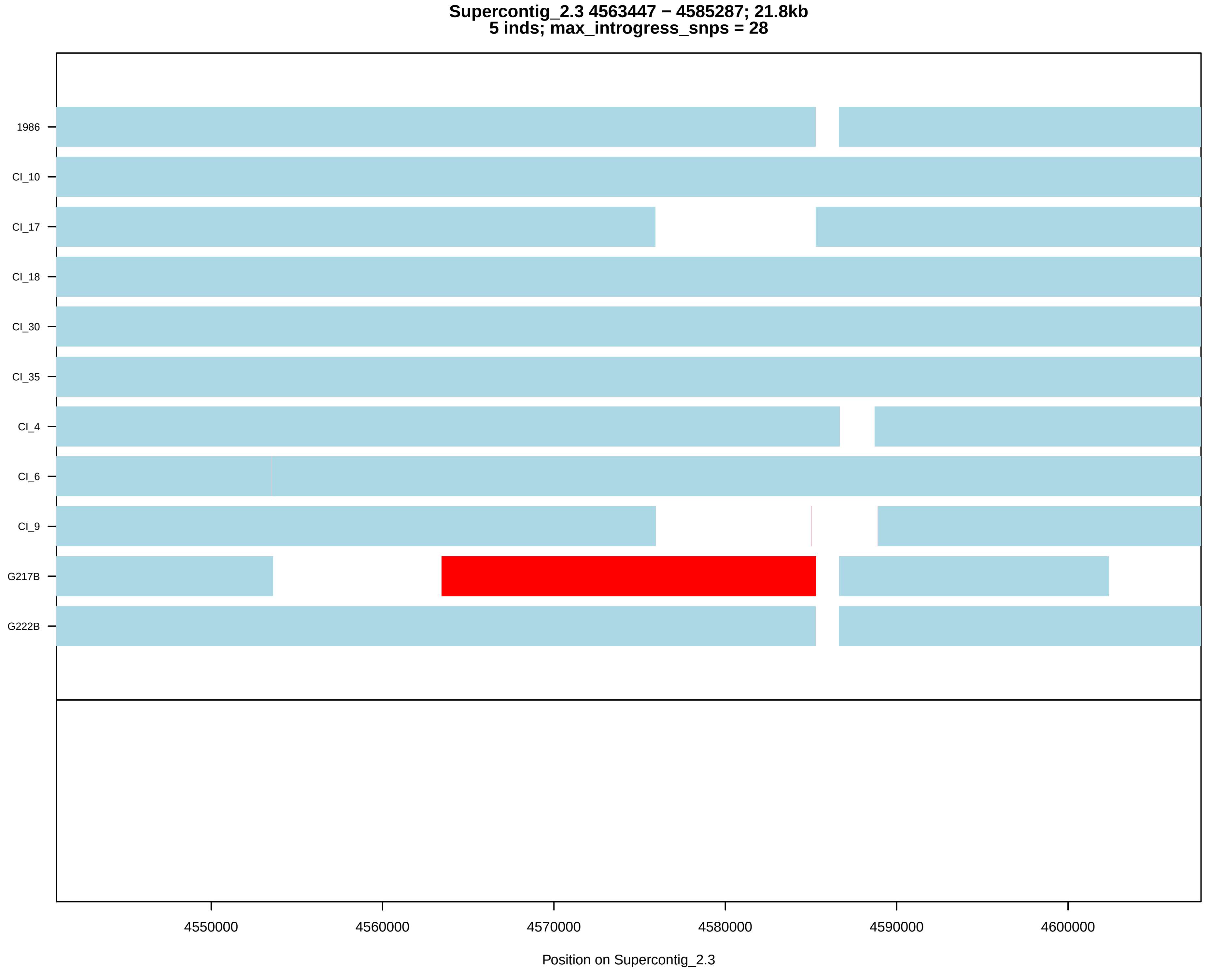

Supercontig_2.3 4563447 − 4585287; 21.8kb
5 inds; max_introgress_snps = 28
1986
CI_10
CI_17
CI_18
CI_30
CI_35
CI_4
CI_6
CI_9
G217B
G222B
4550000
4560000
4570000
4580000
4590000
4600000
Position on Supercontig_2.3

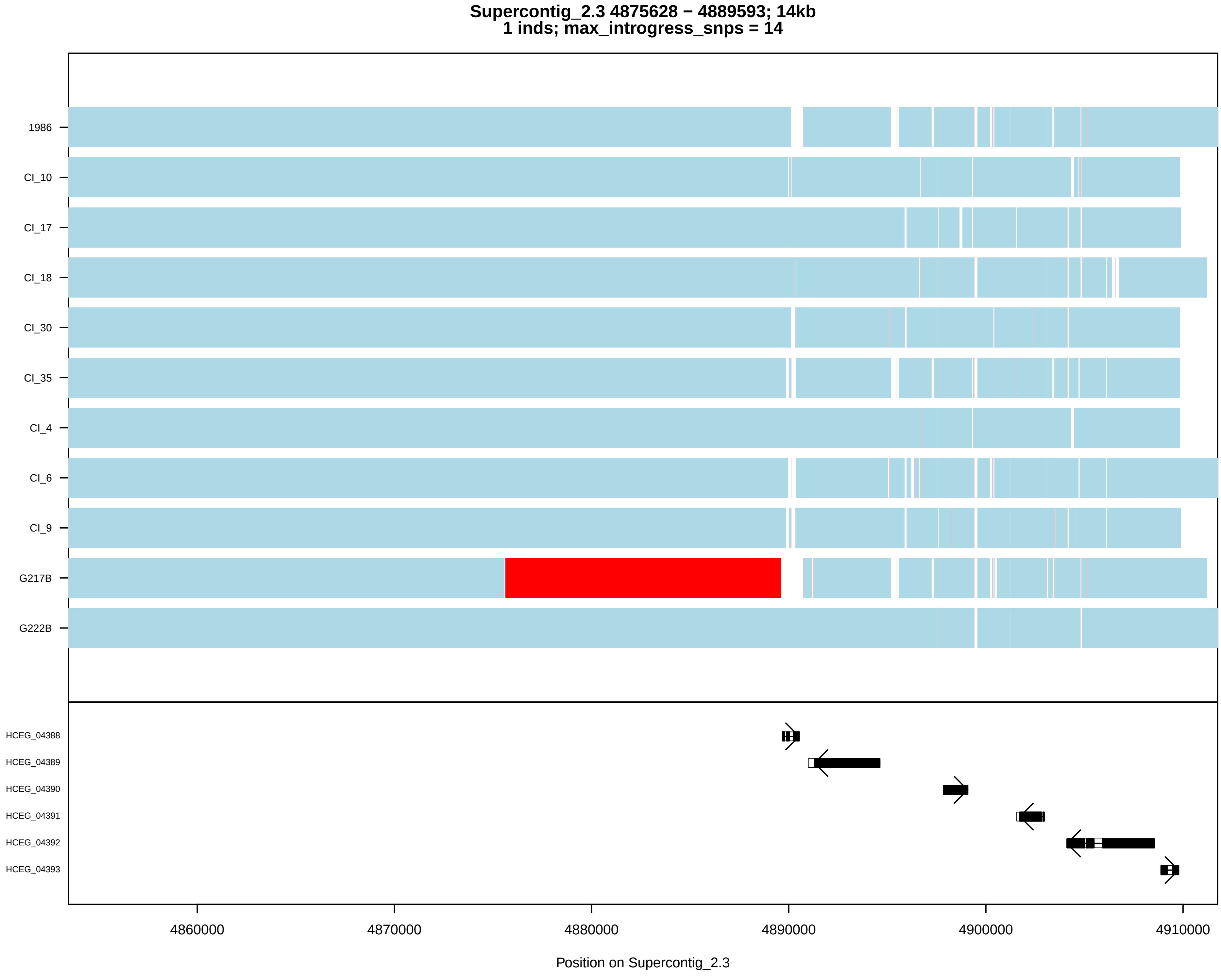

Supercontig_2.3 4875628 − 4889593; 14kb
1 inds; max_introgress_snps = 14
1986
CI_10
CI_17
CI_18
CI_30
CI_35
CI_4
CI_6
CI_9
G217B
G222B
HCEG_04388
HCEG_04389
HCEG_04390
HCEG_04391
HCEG_04392
HCEG_04393
4860000
4870000
4880000
4890000
4900000
4910000
Position on Supercontig_2.3

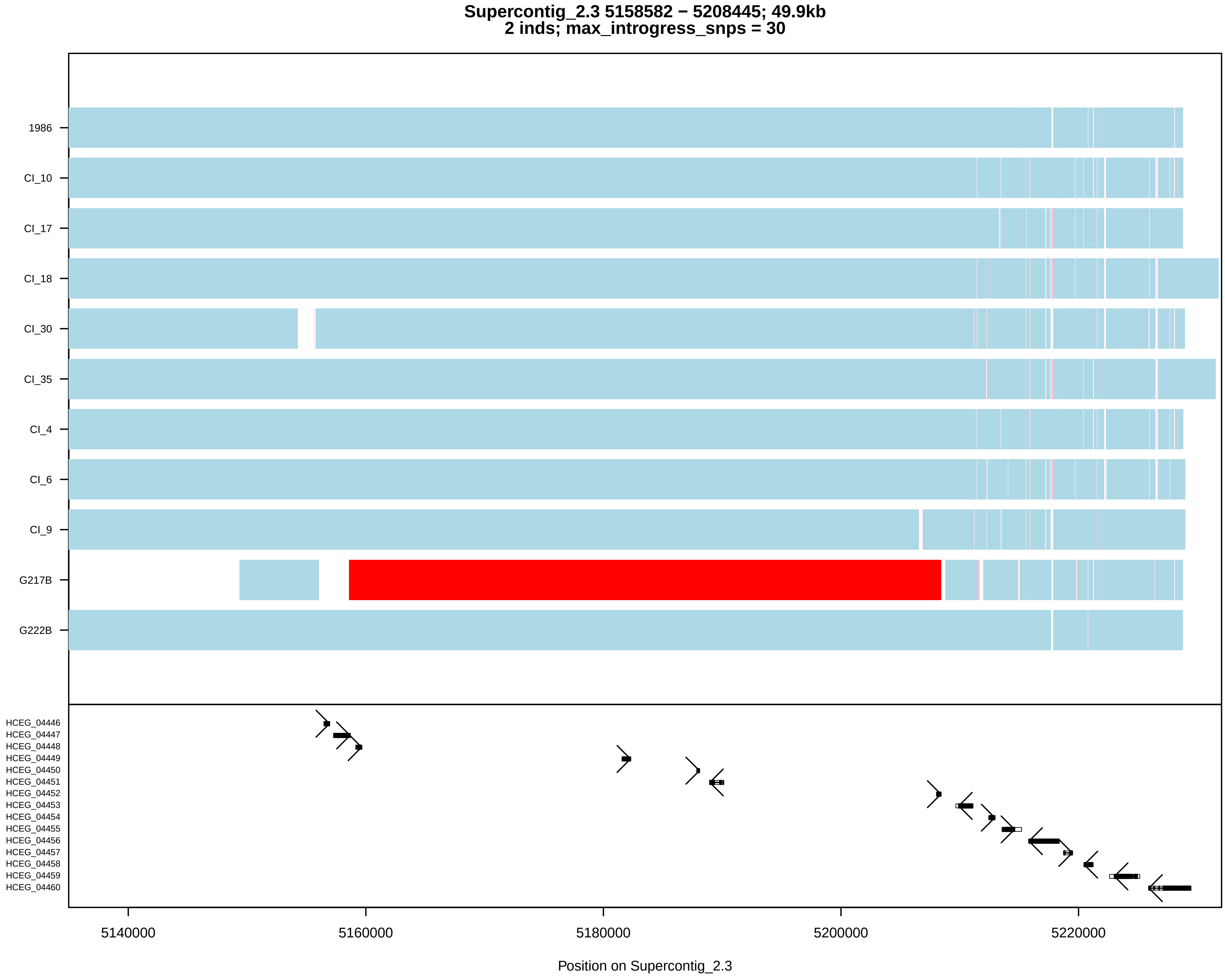

Supercontig_2.3 5158582 − 5208445; 49.9kb
2 inds; max_introgress_snps = 30
1986
CI_10
CI_17
CI_18
CI_30
CI_35
CI_4
CI_6
CI_9
G217B
G222B
HCEG_04446
HCEG_04447
HCEG_04448
HCEG_04449
HCEG_04450
HCEG_04451
HCEG_04452
HCEG_04453
HCEG_04454
HCEG_04455
HCEG_04456
HCEG_04457
HCEG_04458
HCEG_04459
HCEG_04460
5140000
5160000
5180000
5200000
5220000
Position on Supercontig_2.3

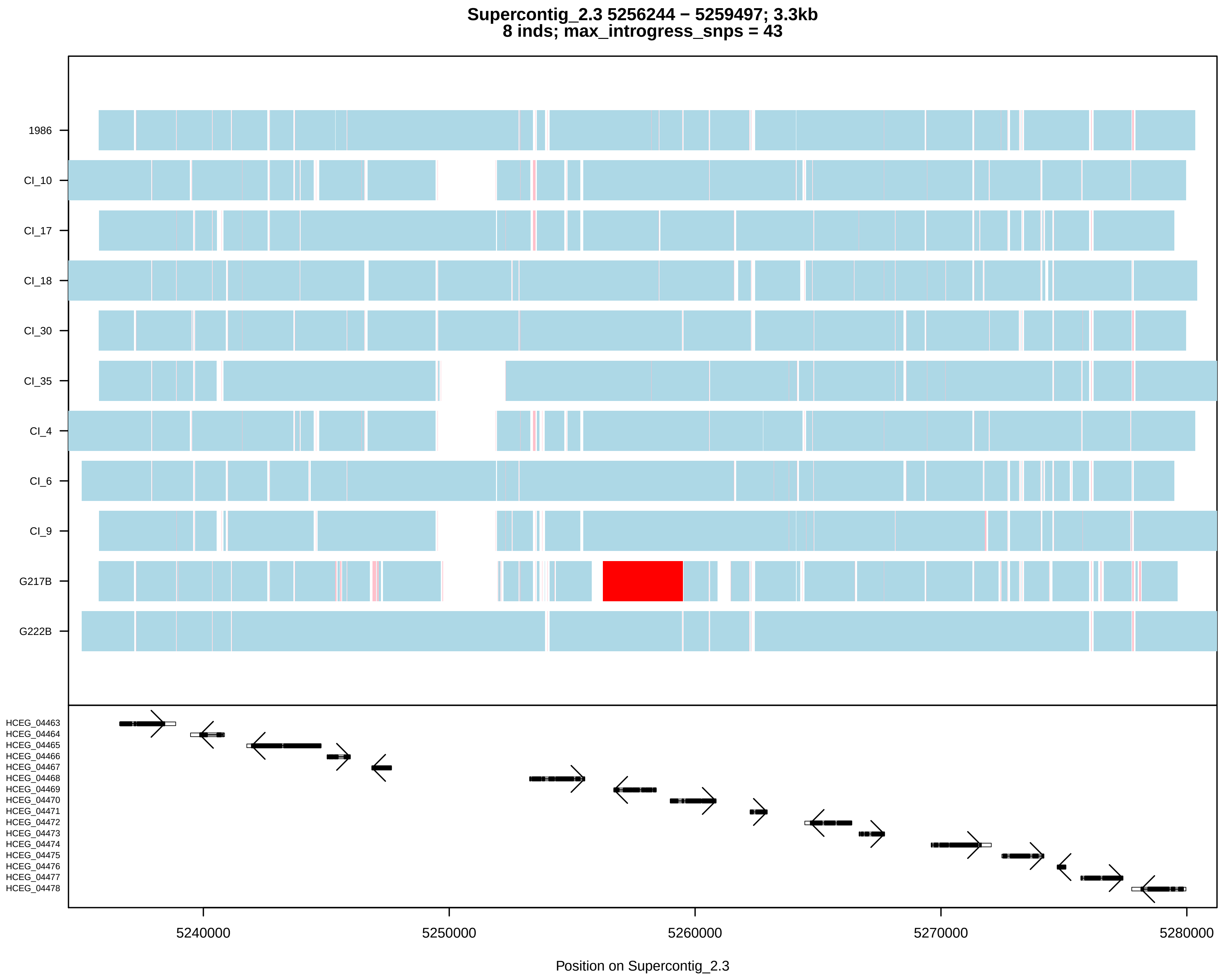
Supercontig_2.3 5256244 − 5259497; 3.3kb
8 inds; max_introgress_snps = 43
1986
CI_10
CI_17
CI_18
CI_30
CI_35
CI_4
CI_6
CI_9
G217B
G222B
HCEG_04463
HCEG_04464
HCEG_04465
HCEG_04466
HCEG_04467
HCEG_04468
HCEG_04469
HCEG_04470
HCEG_04471
HCEG_04472
HCEG_04473
HCEG_04474
HCEG_04475
HCEG_04476
HCEG_04477
HCEG_04478
5240000
5250000
5260000
5270000
5280000
Position on Supercontig_2.3

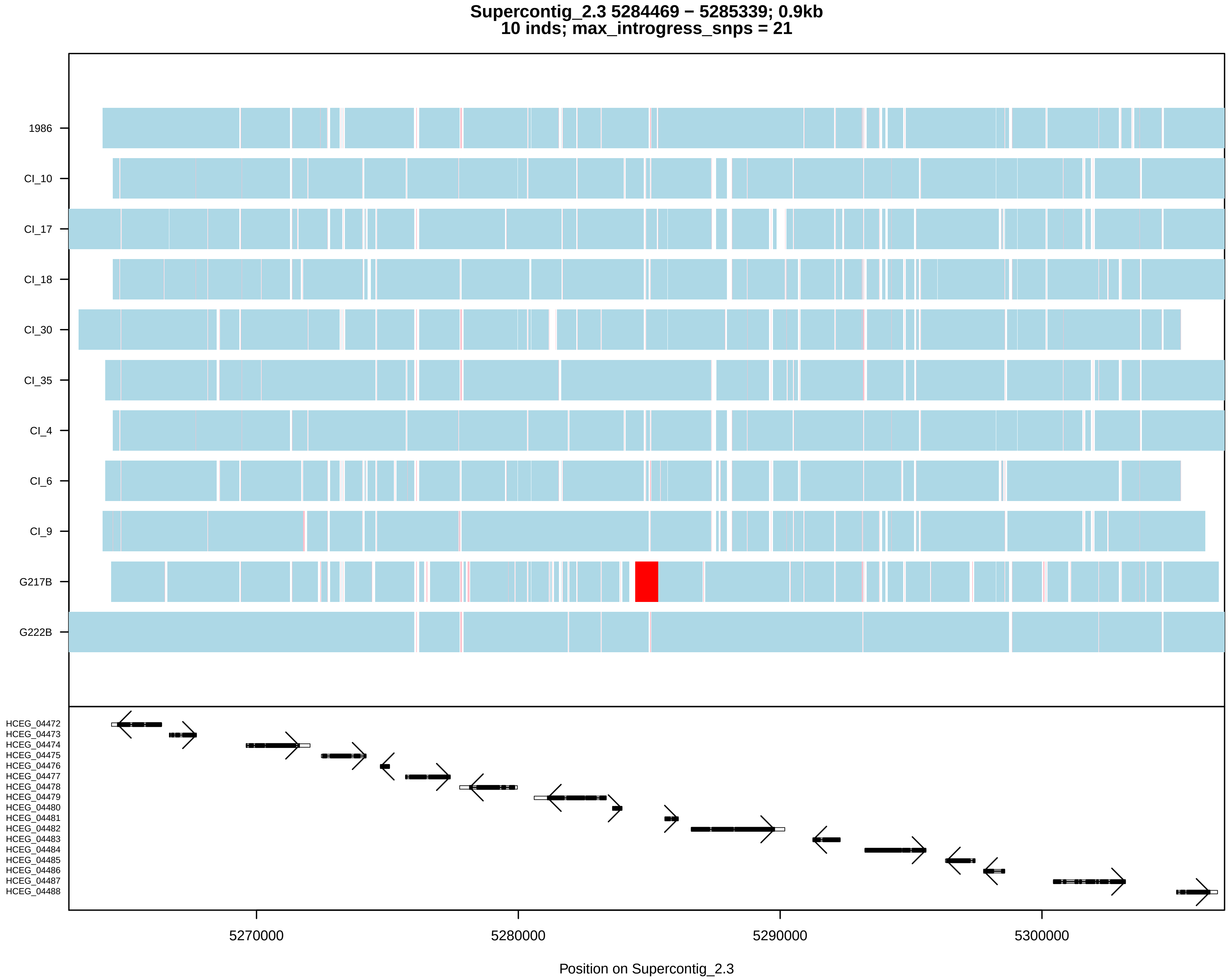
Supercontig_2.3 5284469 − 5285339; 0.9kb
10 inds; max_introgress_snps = 21
1986
CI_10
CI_17
CI_18
CI_30
CI_35
CI_4
CI_6
CI_9
G217B
G222B
HCEG_04472
HCEG_04473
HCEG_04474
HCEG_04475
HCEG_04476
HCEG_04477
HCEG_04478
HCEG_04479
HCEG_04480
HCEG_04481
HCEG_04482
HCEG_04483
HCEG_04484
HCEG_04485
HCEG_04486
HCEG_04487
HCEG_04488
5270000
5280000
5290000
5300000
Position on Supercontig_2.3

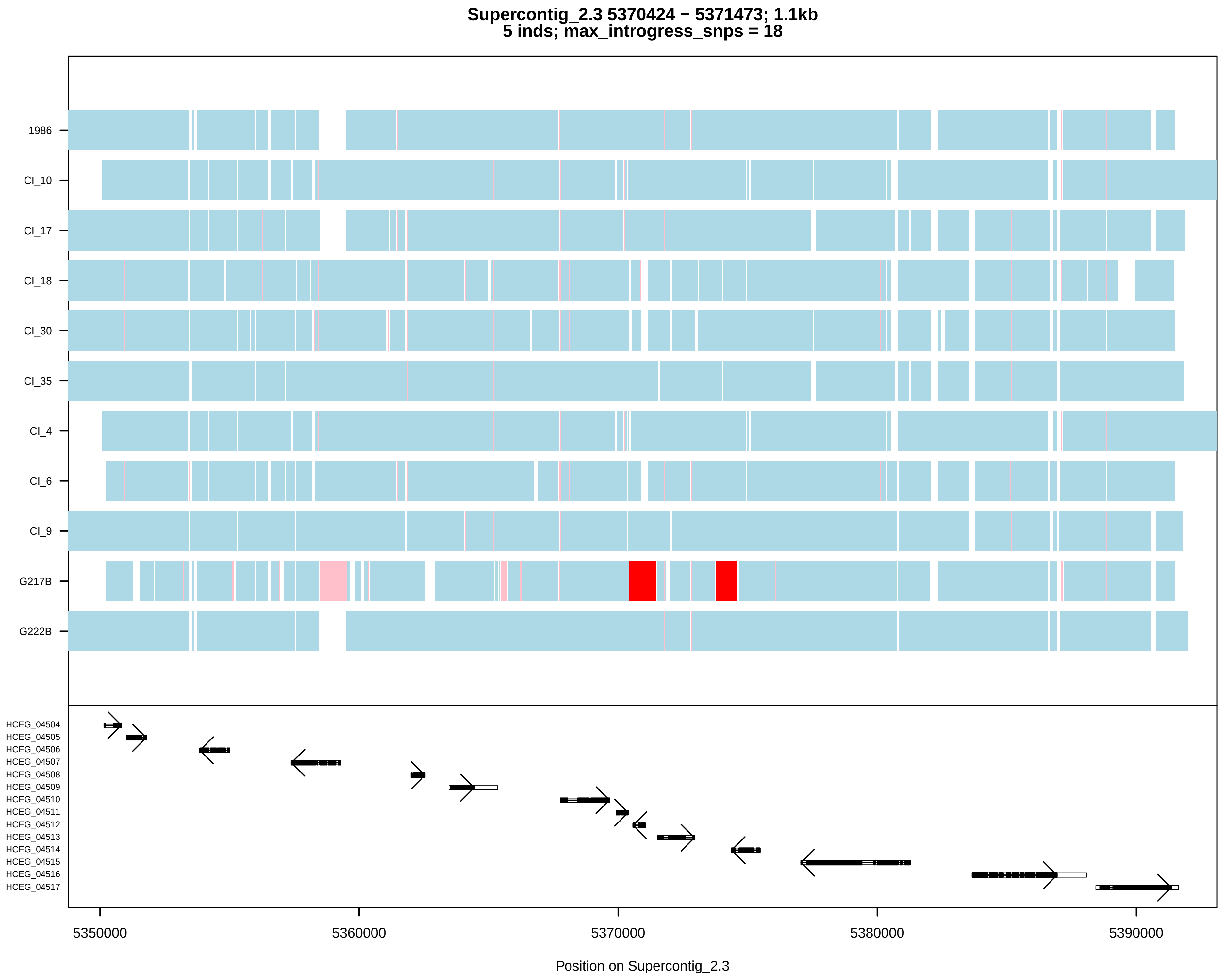
Supercontig_2.3 5370424 − 5371473; 1.1kb
5 inds; max_introgress_snps = 18
1986
CI_10
CI_17
CI_18
CI_30
CI_35
CI_4
CI_6
CI_9
G217B
G222B
HCEG_04504
HCEG_04505
HCEG_04506
HCEG_04507
HCEG_04508
HCEG_04509
HCEG_04510
HCEG_04511
HCEG_04512
HCEG_04513
HCEG_04514
HCEG_04515
HCEG_04516
HCEG_04517
5350000
5360000
5370000
5380000
5390000
Position on Supercontig_2.3

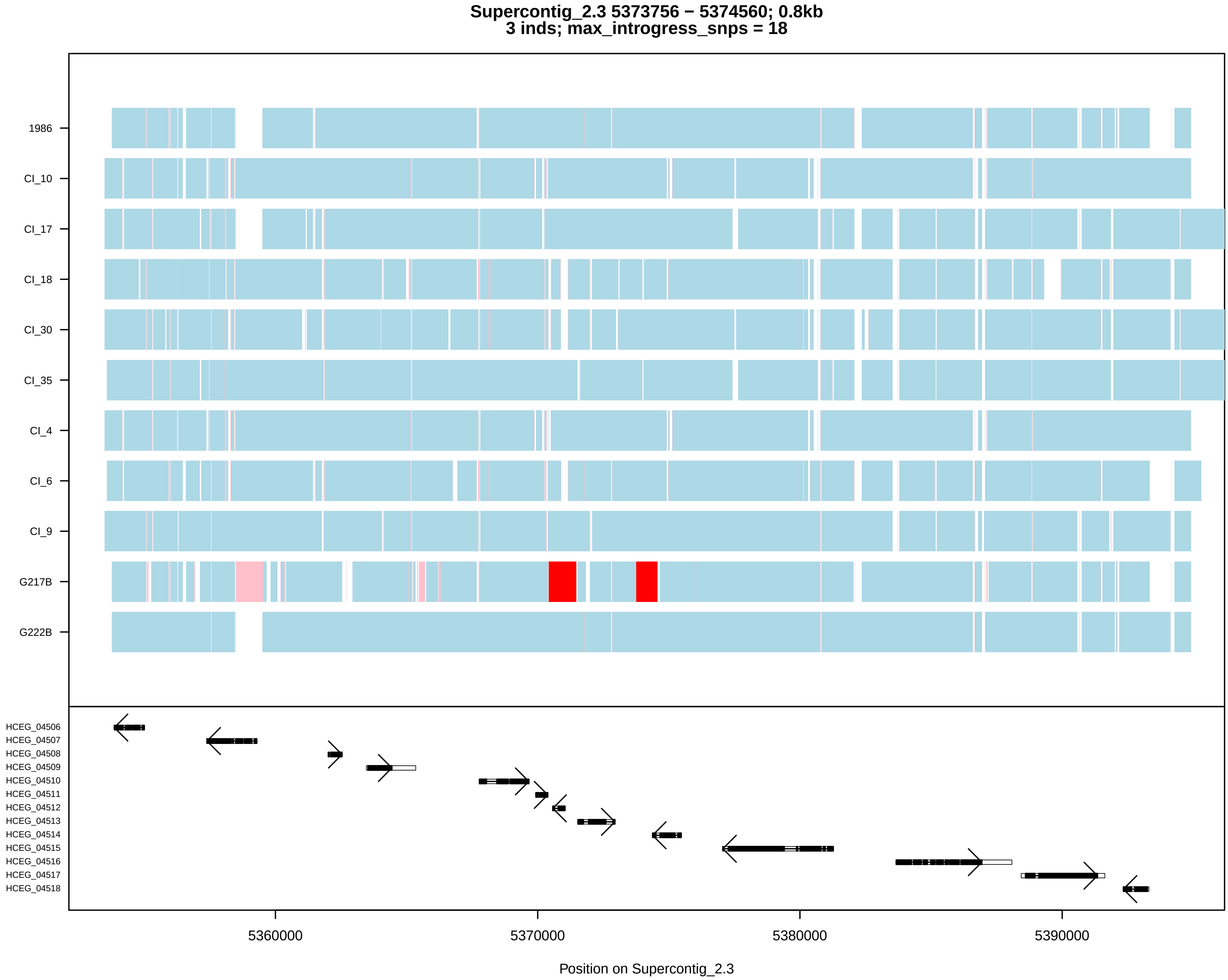
Supercontig_2.3 5373756 − 5374560; 0.8kb
3 inds; max_introgress_snps = 18
1986
CI_10
CI_17
CI_18
CI_30
CI_35
CI_4
CI_6
CI_9
G217B
G222B
HCEG_04506
HCEG_04507
HCEG_04508
HCEG_04509
HCEG_04510
HCEG_04511
HCEG_04512
HCEG_04513
HCEG_04514
HCEG_04515
HCEG_04516
HCEG_04517
HCEG_04518
5360000
5370000
5380000
5390000
Position on Supercontig_2.3

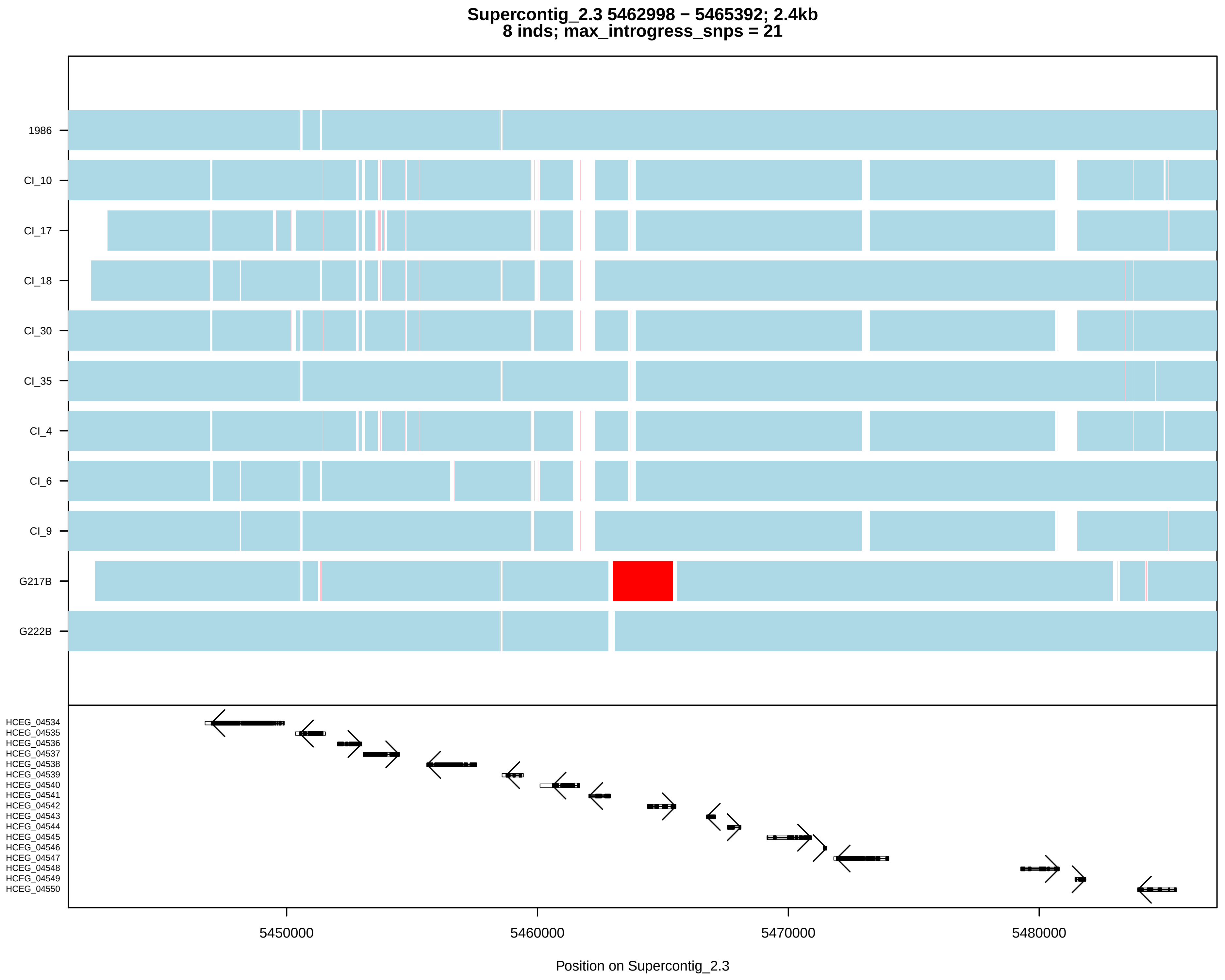

Supercontig_2.3 5462998 − 5465392; 2.4kb
8 inds; max_introgress_snps = 21
1986
CI_10
CI_17
CI_18
CI_30
CI_35
CI_4
CI_6
CI_9
G217B
G222B
HCEG_04534
HCEG_04535
HCEG_04536
HCEG_04537
HCEG_04538
HCEG_04539
HCEG_04540
HCEG_04541
HCEG_04542
HCEG_04543
HCEG_04544
HCEG_04545
HCEG_04546
HCEG_04547
HCEG_04548
HCEG_04549
HCEG_04550
5450000
5460000
5470000
5480000
Position on Supercontig_2.3

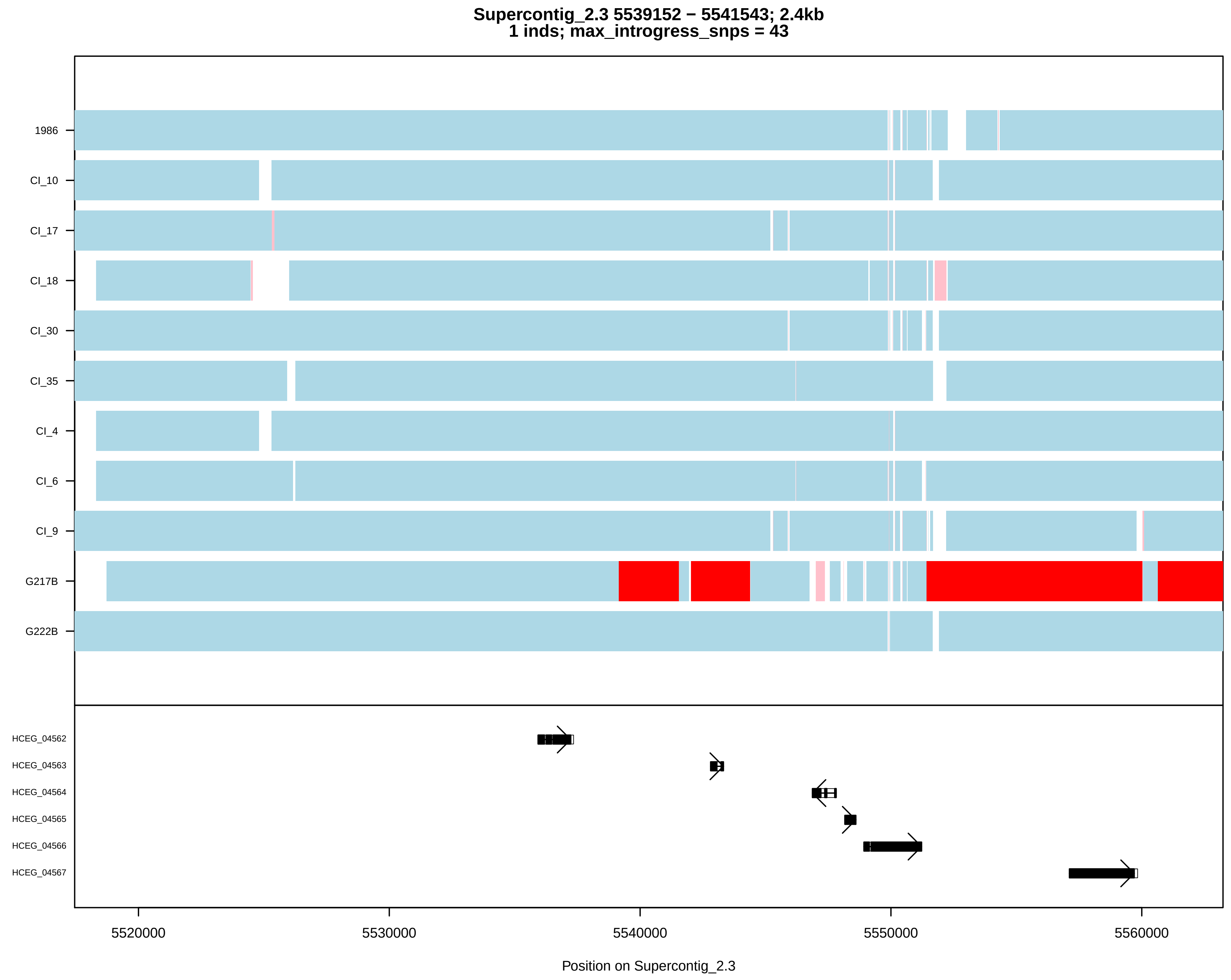

Supercontig_2.3 5539152 − 5541543; 2.4kb
1 inds; max_introgress_snps = 43
1986
CI_10
CI_17
CI_18
CI_30
CI_35
CI_4
CI_6
CI_9
G217B
G222B
HCEG_04562
HCEG_04563
HCEG_04564
HCEG_04565
HCEG_04566
HCEG_04567
5520000
5530000
5540000
5550000
5560000
Position on Supercontig_2.3

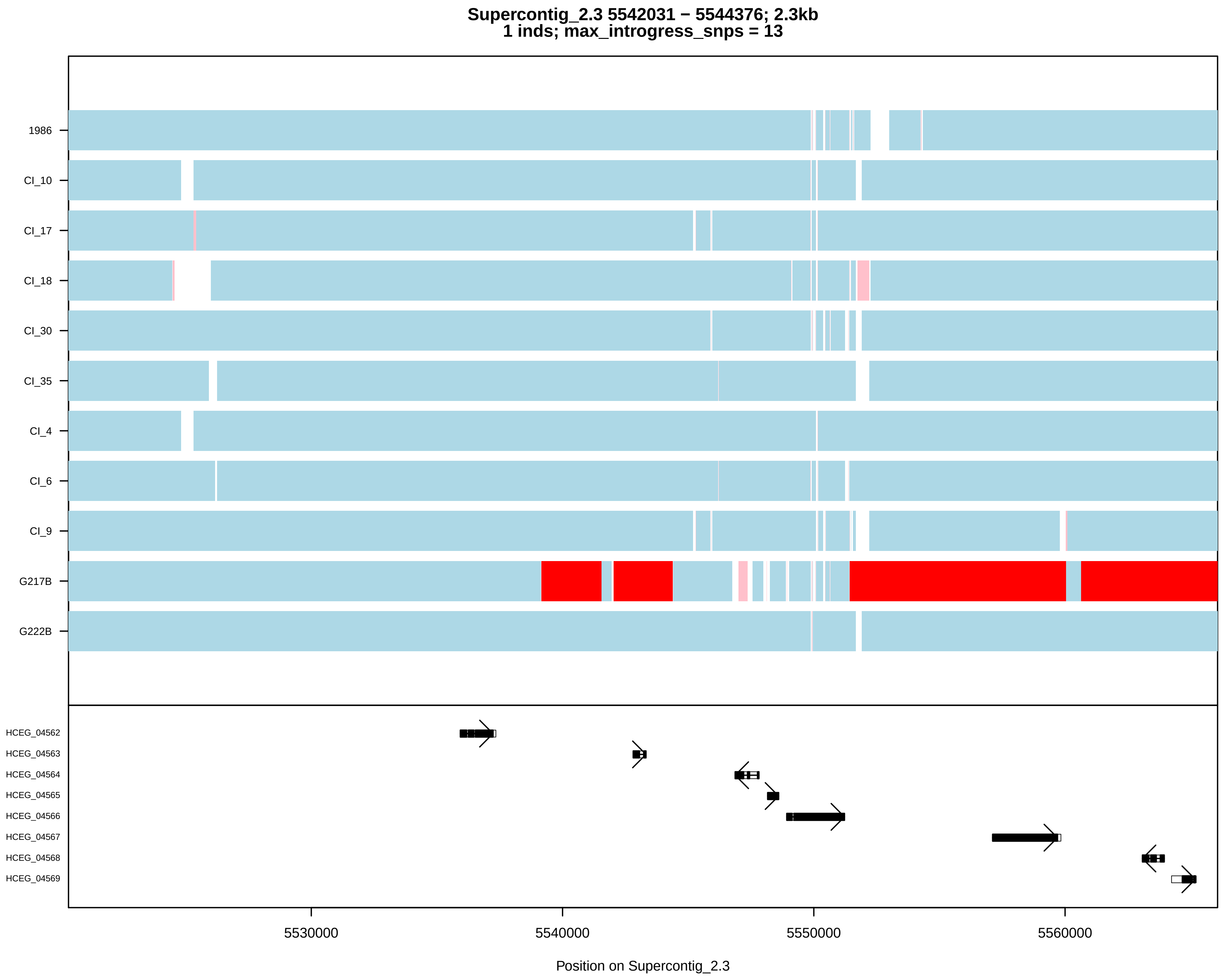
Supercontig_2.3 5542031 − 5544376; 2.3kb
1 inds; max_introgress_snps = 13
1986
CI_10
CI_17
CI_18
CI_30
CI_35
CI_4
CI_6
CI_9
G217B
G222B
HCEG_04562
HCEG_04563
HCEG_04564
HCEG_04565
HCEG_04566
HCEG_04567
HCEG_04568
HCEG_04569
5530000
5540000
5550000
5560000
Position on Supercontig_2.3

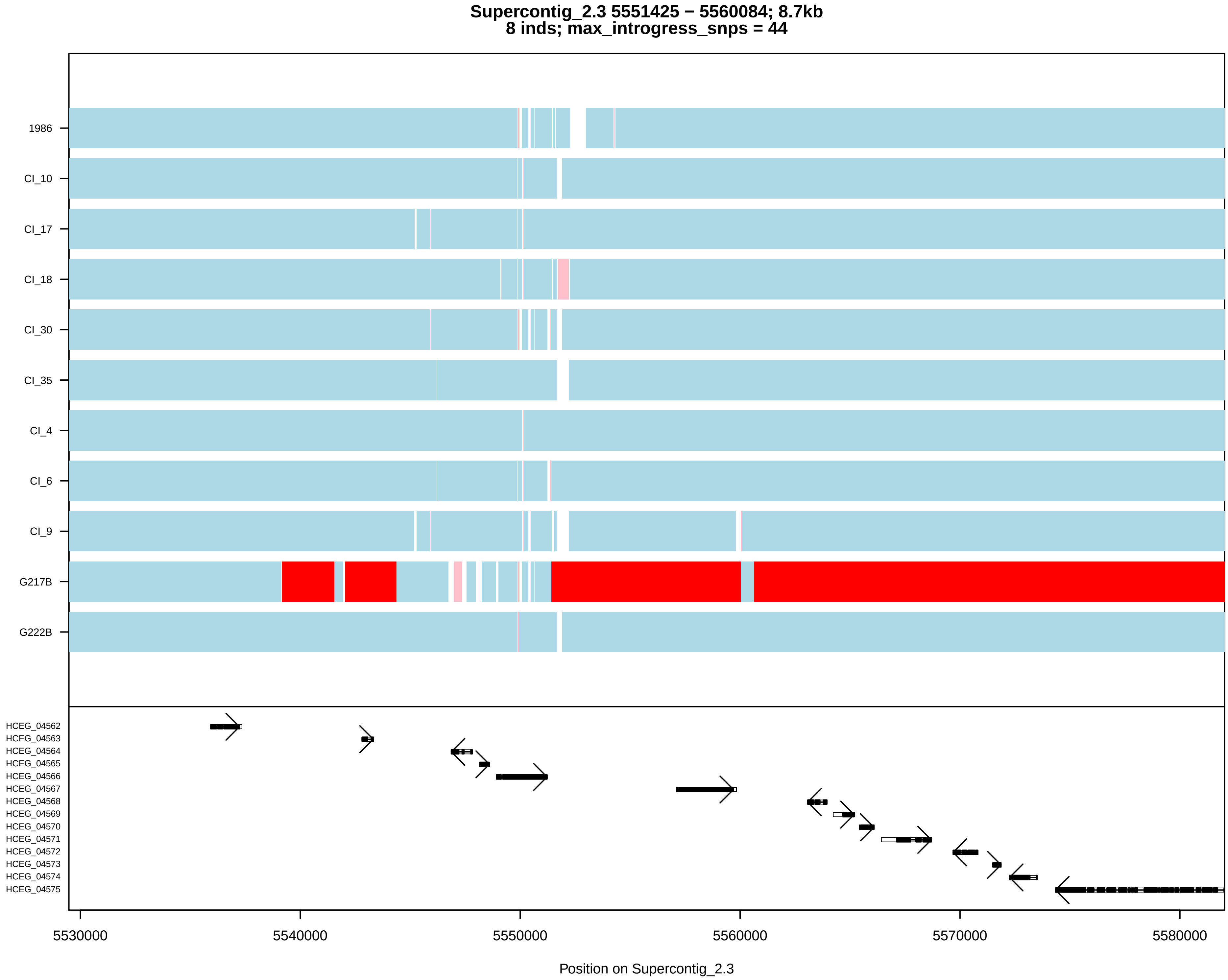

Supercontig_2.3 5551425 − 5560084; 8.7kb
8 inds; max_introgress_snps = 44
1986
CI_10
CI_17
CI_18
CI_30
CI_35
CI_4
CI_6
CI_9
G217B
G222B
HCEG_04562
HCEG_04563
HCEG_04564
HCEG_04565
HCEG_04566
HCEG_04567
HCEG_04568
HCEG_04569
HCEG_04570
HCEG_04571
HCEG_04572
HCEG_04573
HCEG_04574
HCEG_04575
5530000
5540000
5550000
5560000
5570000
5580000
Position on Supercontig_2.3

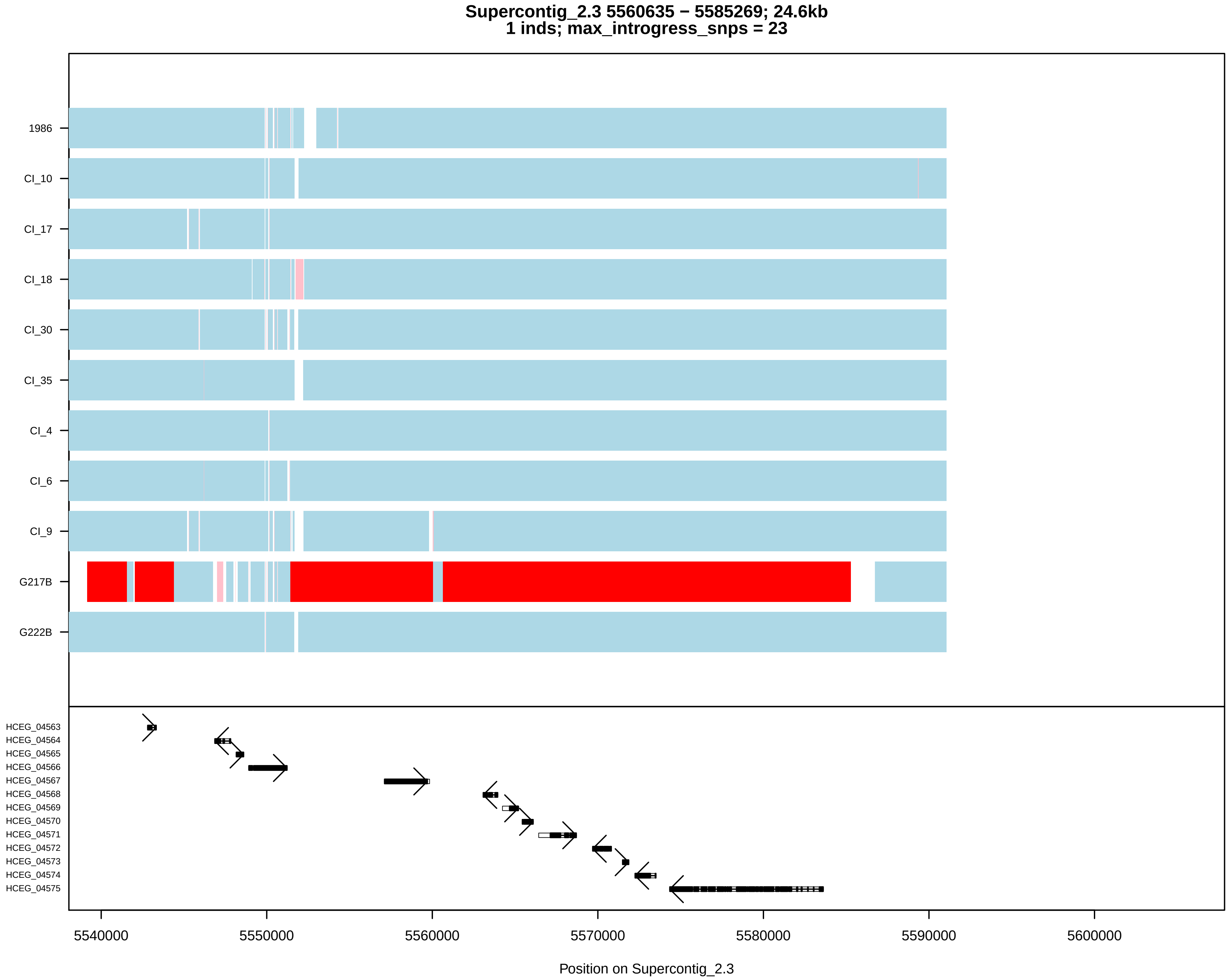

Supercontig_2.3 5560635 − 5585269; 24.6kb
1 inds; max_introgress_snps = 23
1986
CI_10
CI_17
CI_18
CI_30
CI_35
CI_4
CI_6
CI_9
G217B
G222B
HCEG_04563
HCEG_04564
HCEG_04565
HCEG_04566
HCEG_04567
HCEG_04568
HCEG_04569
HCEG_04570
HCEG_04571
HCEG_04572
HCEG_04573
HCEG_04574
HCEG_04575
5540000
5550000
5560000
5570000
5580000
5590000
5600000
Position on Supercontig_2.3

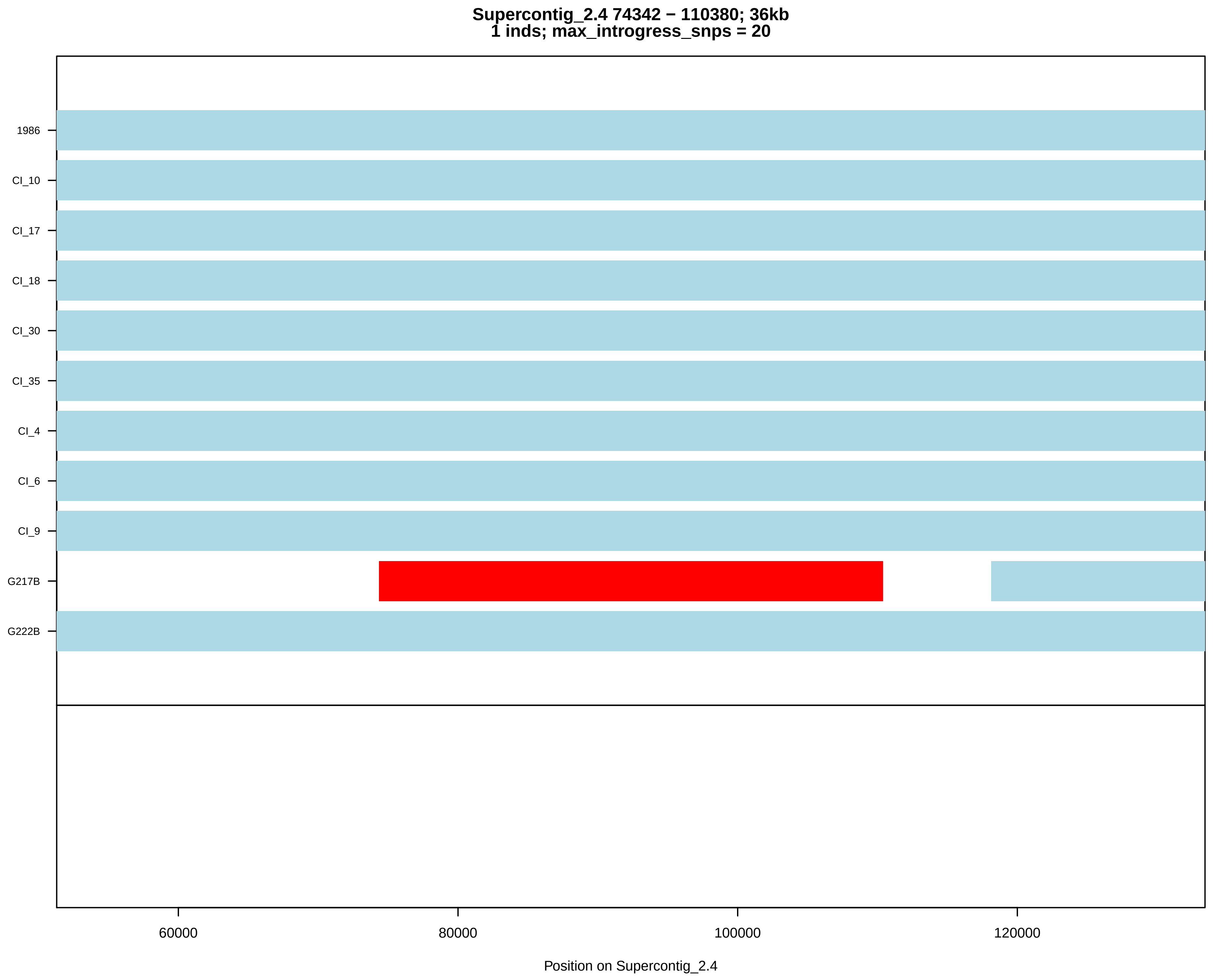

Supercontig_2.4 74342 − 110380; 36kb
1 inds; max_introgress_snps = 20
1986
CI_10
CI_17
CI_18
CI_30
CI_35
CI_4
CI_6
CI_9
G217B
G222B
60000
80000
100000
120000
Position on Supercontig_2.4

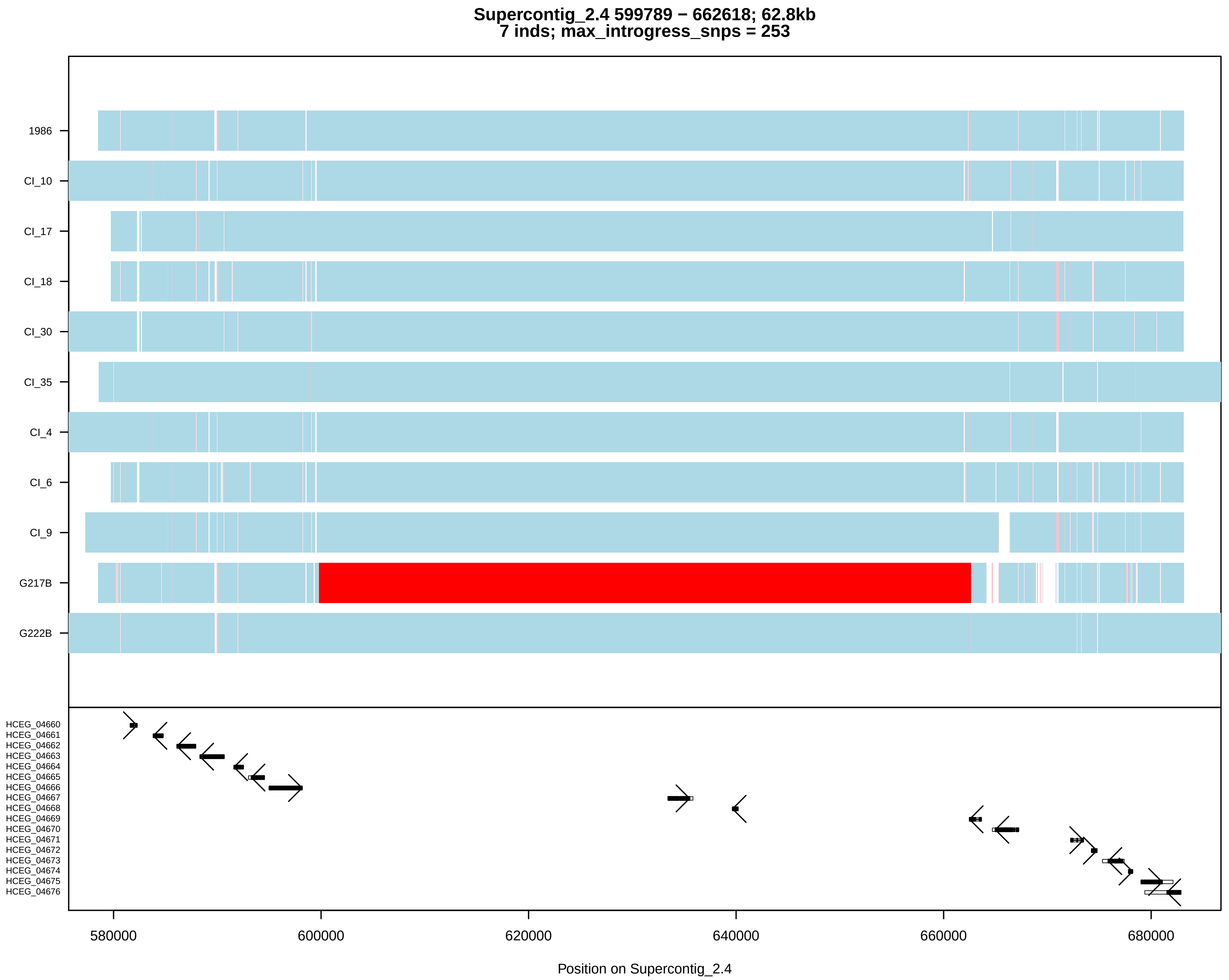
Supercontig_2.4 599789 − 662618; 62.8kb
7 inds; max_introgress_snps = 253
1986
CI_10
CI_17
CI_18
CI_30
CI_35
CI_4
CI_6
CI_9
G217B
G222B
HCEG_04660
HCEG_04661
HCEG_04662
HCEG_04663
HCEG_04664
HCEG_04665
HCEG_04666
HCEG_04667
HCEG_04668
HCEG_04669
HCEG_04670
HCEG_04671
HCEG_04672
HCEG_04673
HCEG_04674
HCEG_04675
HCEG_04676
580000
600000
620000
640000
660000
680000
Position on Supercontig_2.4

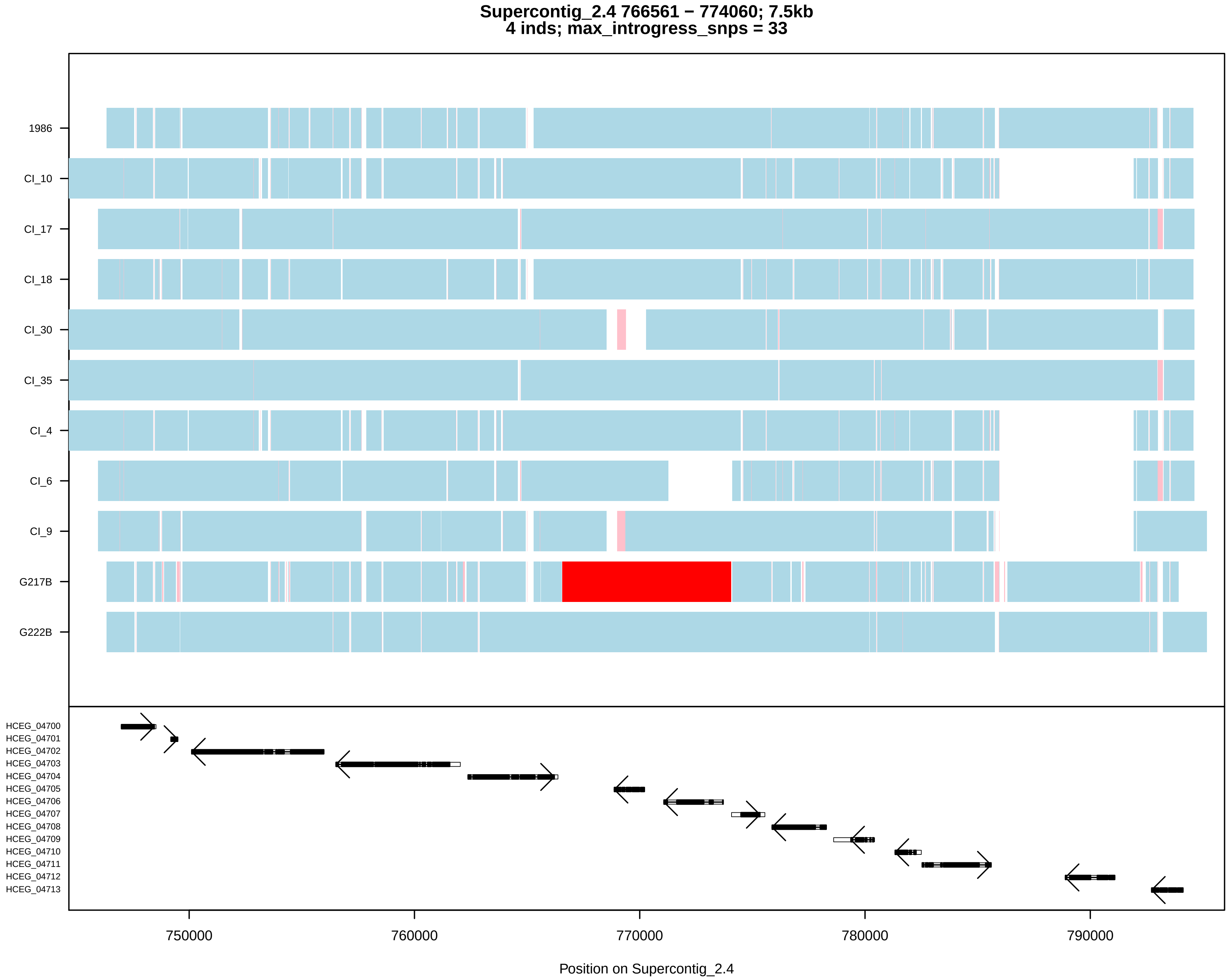

Supercontig_2.4 766561 − 774060; 7.5kb
4 inds; max_introgress_snps = 33
1986
CI_10
CI_17
CI_18
CI_30
CI_35
CI_4
CI_6
CI_9
G217B
G222B
HCEG_04700
HCEG_04701
HCEG_04702
HCEG_04703
HCEG_04704
HCEG_04705
HCEG_04706
HCEG_04707
HCEG_04708
HCEG_04709
HCEG_04710
HCEG_04711
HCEG_04712
HCEG_04713
750000
760000
770000
780000
790000
Position on Supercontig_2.4

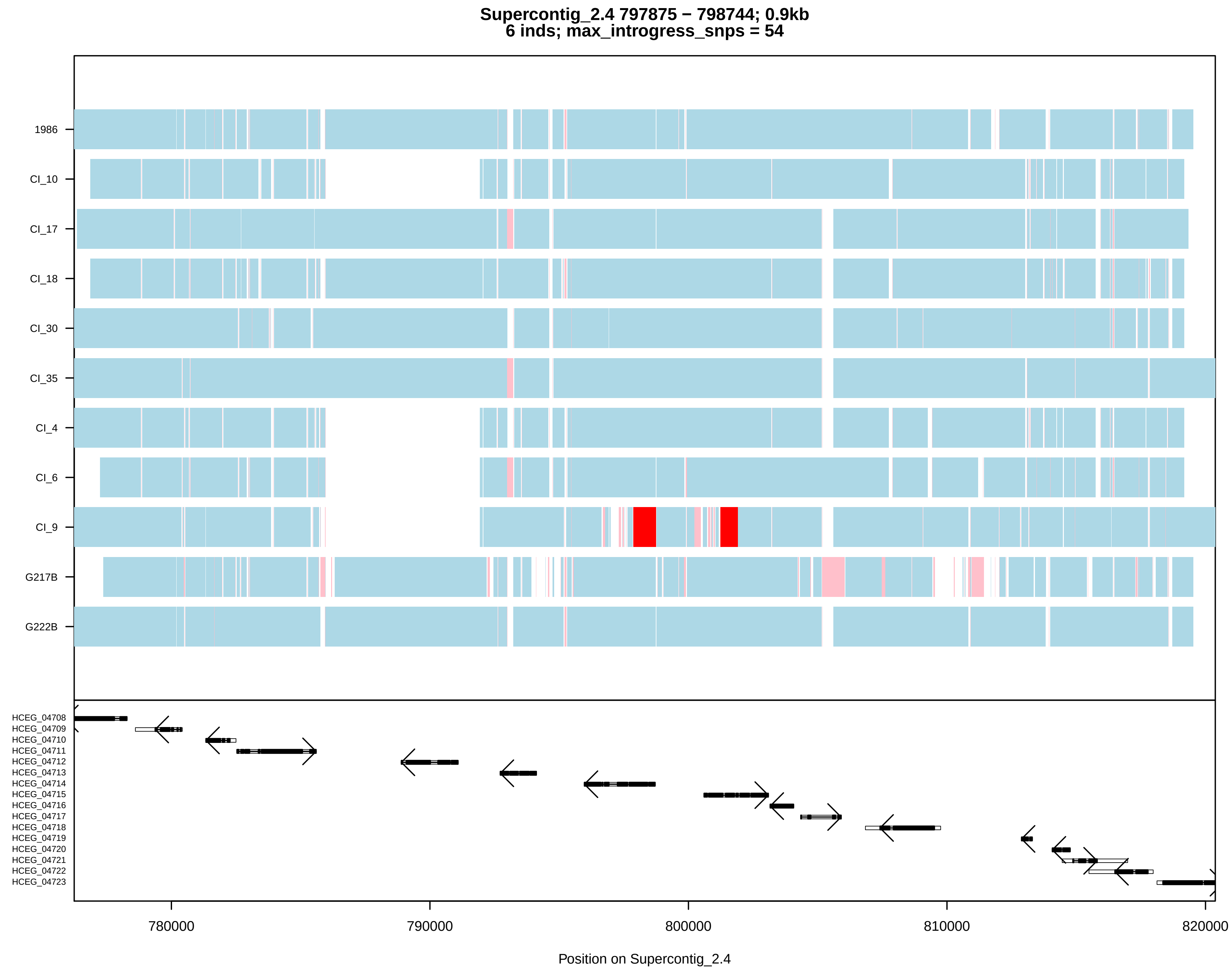

Supercontig_2.4 797875 − 798744; 0.9kb
6 inds; max_introgress_snps = 54
1986
CI_10
CI_17
CI_18
CI_30
CI_35
CI_4
CI_6
CI_9
G217B
G222B
HCEG_04708
HCEG_04709
HCEG_04710
HCEG_04711
HCEG_04712
HCEG_04713
HCEG_04714
HCEG_04715
HCEG_04716
HCEG_04717
HCEG_04718
HCEG_04719
HCEG_04720
HCEG_04721
HCEG_04722
HCEG_04723
780000
790000
800000
810000
820000
Position on Supercontig_2.4

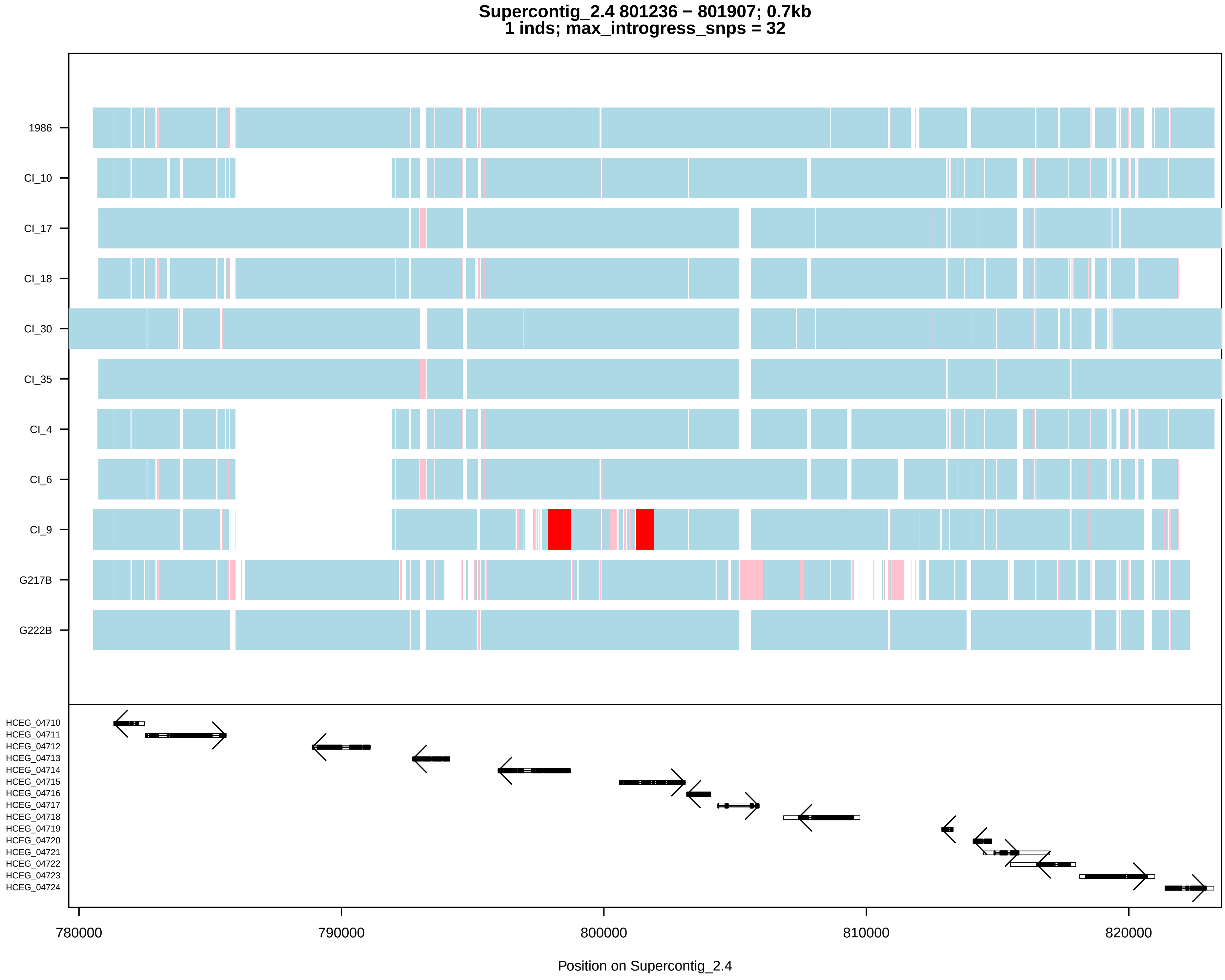

Supercontig_2.4 801236 − 801907; 0.7kb
1 inds; max_introgress_snps = 32
1986
CI_10
CI_17
CI_18
CI_30
CI_35
CI_4
CI_6
CI_9
G217B
G222B
HCEG_04710
HCEG_04711
HCEG_04712
HCEG_04713
HCEG_04714
HCEG_04715
HCEG_04716
HCEG_04717
HCEG_04718
HCEG_04719
HCEG_04720
HCEG_04721
HCEG_04722
HCEG_04723
HCEG_04724
780000
790000
800000
810000
820000
Position on Supercontig_2.4

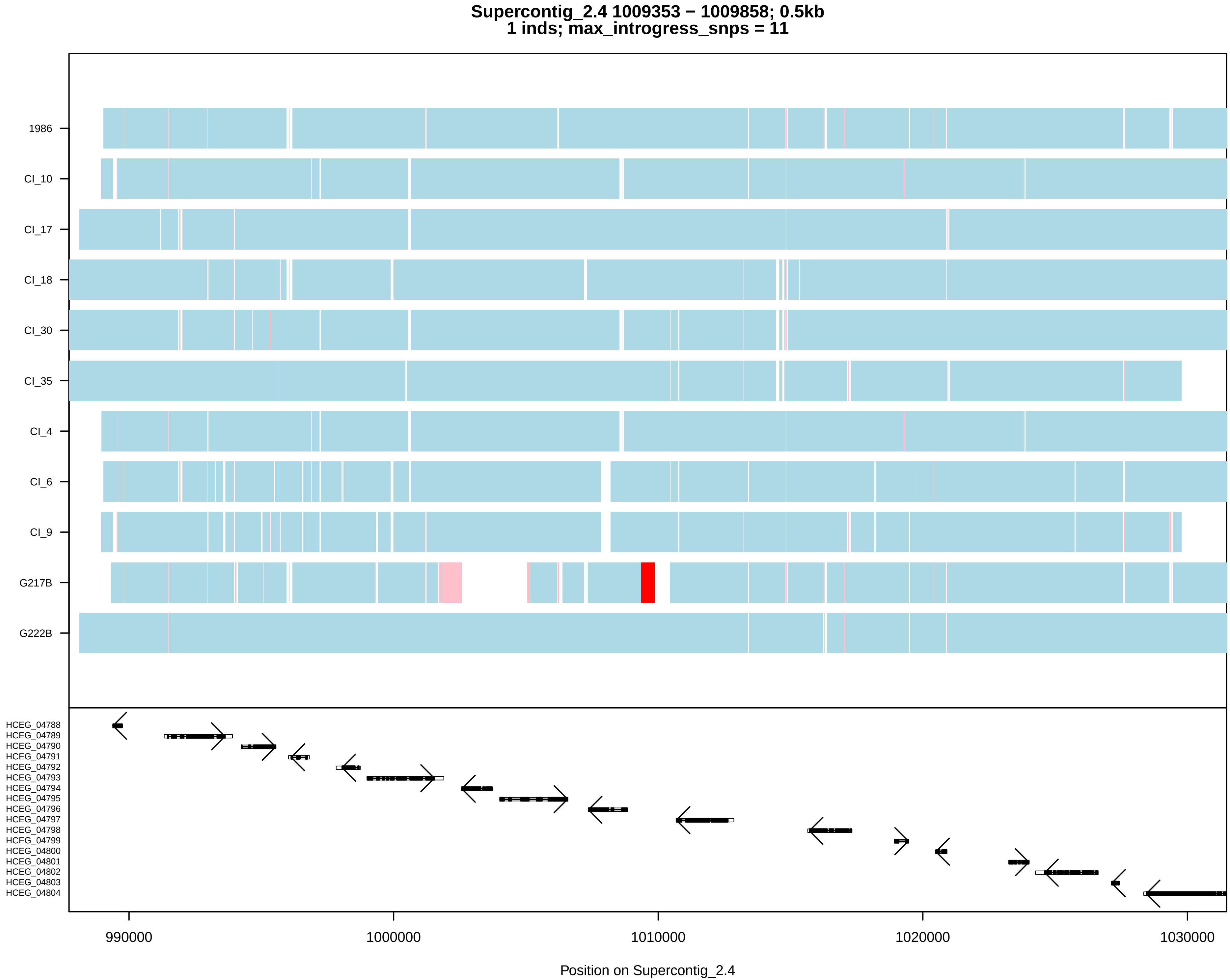

Supercontig_2.4 1009353 − 1009858; 0.5kb
1 inds; max_introgress_snps = 11
1986
CI_10
CI_17
CI_18
CI_30
CI_35
CI_4
CI_6
CI_9
G217B
G222B
HCEG_04788
HCEG_04789
HCEG_04790
HCEG_04791
HCEG_04792
HCEG_04793
HCEG_04794
HCEG_04795
HCEG_04796
HCEG_04797
HCEG_04798
HCEG_04799
HCEG_04800
HCEG_04801
HCEG_04802
HCEG_04803
HCEG_04804
990000
1000000
1010000
1020000
1030000
Position on Supercontig_2.4

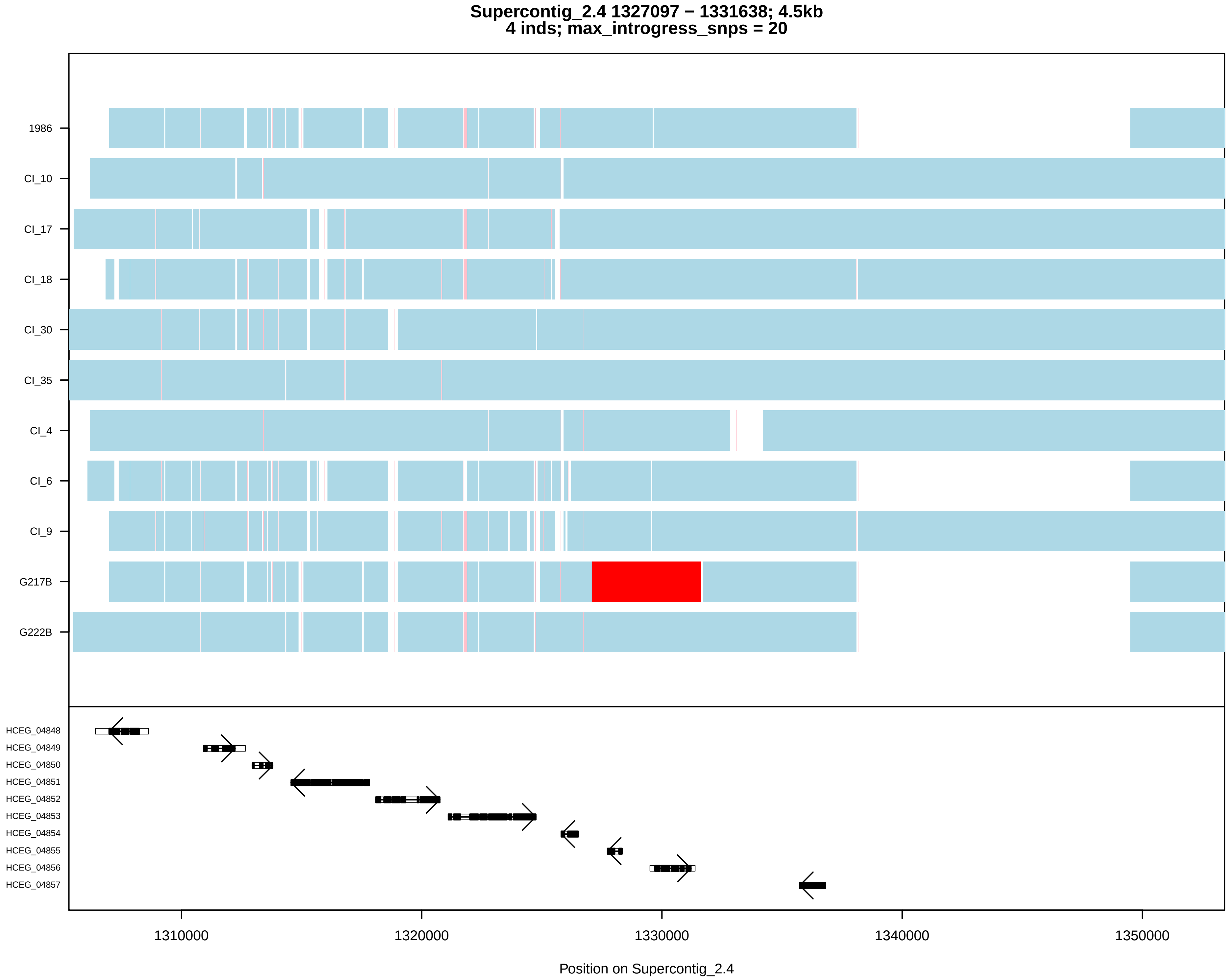

Supercontig_2.4 1327097 − 1331638; 4.5kb
4 inds; max_introgress_snps = 20
1986
CI_10
CI_17
CI_18
CI_30
CI_35
CI_4
CI_6
CI_9
G217B
G222B
HCEG_04848
HCEG_04849
HCEG_04850
HCEG_04851
HCEG_04852
HCEG_04853
HCEG_04854
HCEG_04855
HCEG_04856
HCEG_04857
1310000
1320000
1330000
1340000
1350000
Position on Supercontig_2.4

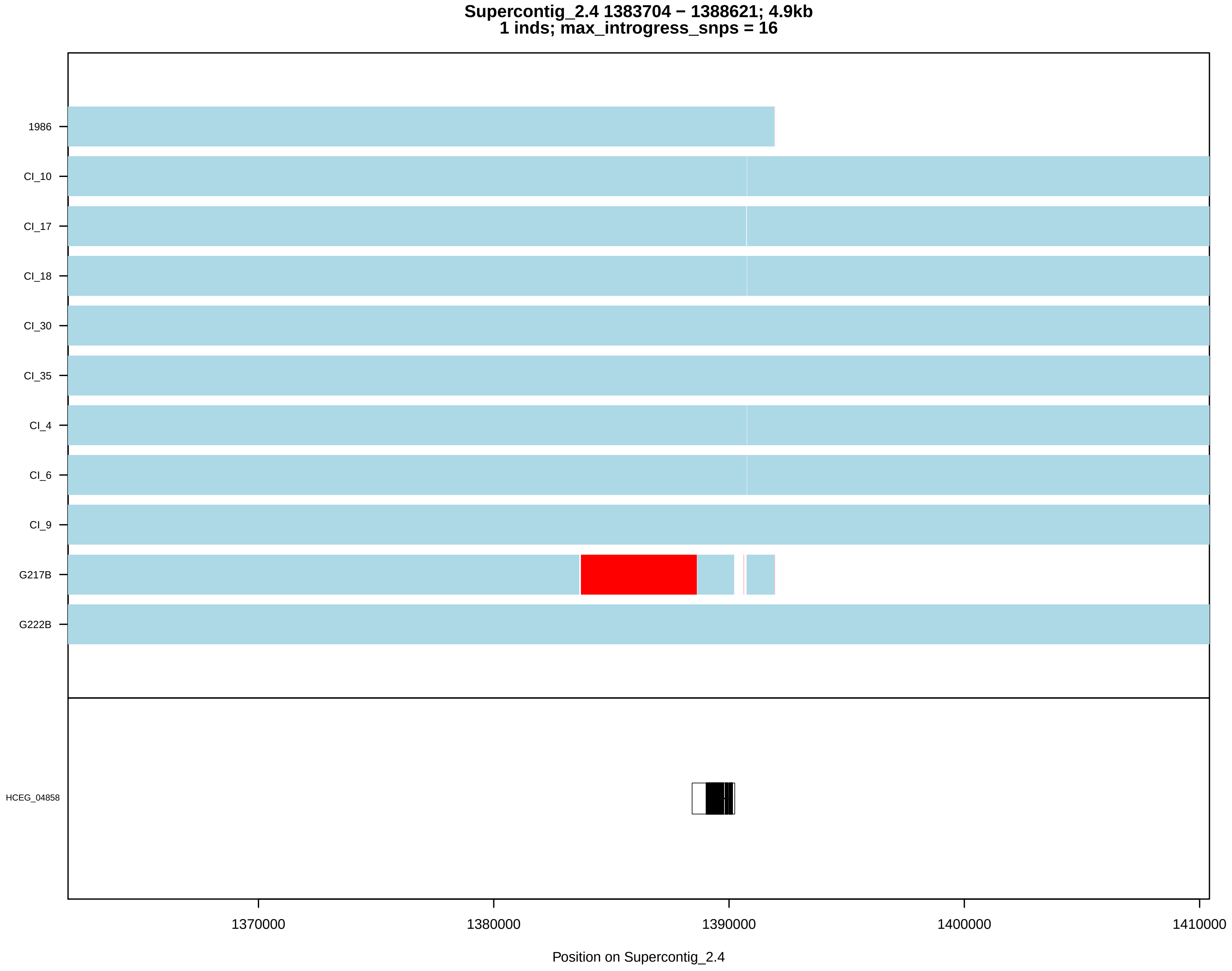
Supercontig_2.4 1383704 − 1388621; 4.9kb
1 inds; max_introgress_snps = 16
1986
CI_10
CI_17
CI_18
CI_30
CI_35
CI_4
CI_6
CI_9
G217B
G222B
HCEG_04858
1370000
1380000
1390000
1400000
1410000
Position on Supercontig_2.4

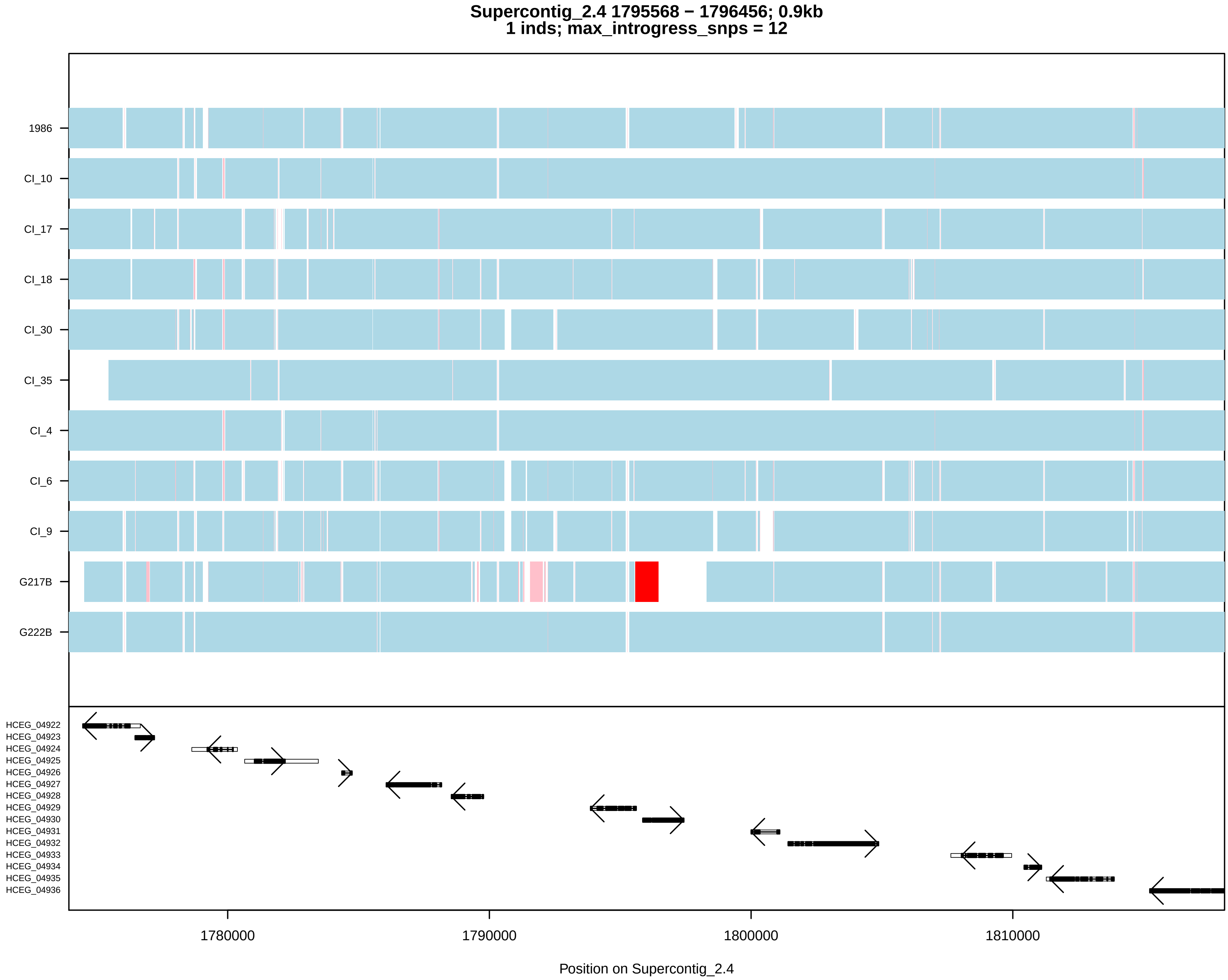

Supercontig_2.4 1795568 − 1796456; 0.9kb
1 inds; max_introgress_snps = 12
1986
CI_10
CI_17
CI_18
CI_30
CI_35
CI_4
CI_6
CI_9
G217B
G222B
HCEG_04922
HCEG_04923
HCEG_04924
HCEG_04925
HCEG_04926
HCEG_04927
HCEG_04928
HCEG_04929
HCEG_04930
HCEG_04931
HCEG_04932
HCEG_04933
HCEG_04934
HCEG_04935
HCEG_04936
1780000
1790000
1800000
1810000
Position on Supercontig_2.4

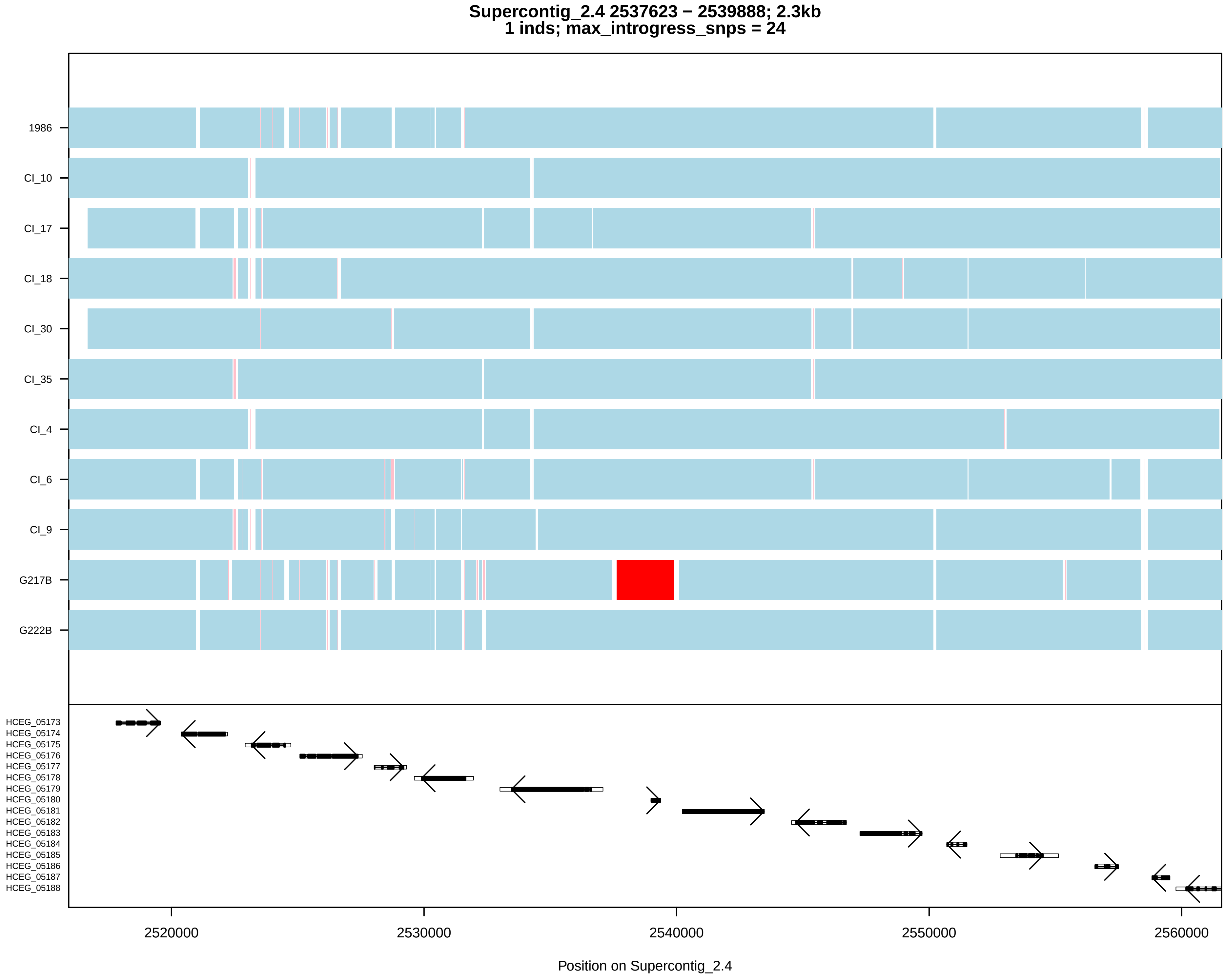

Supercontig_2.4 2537623 − 2539888; 2.3kb
1 inds; max_introgress_snps = 24
1986
CI_10
CI_17
CI_18
CI_30
CI_35
CI_4
CI_6
CI_9
G217B
G222B
HCEG_05173
HCEG_05174
HCEG_05175
HCEG_05176
HCEG_05177
HCEG_05178
HCEG_05179
HCEG_05180
HCEG_05181
HCEG_05182
HCEG_05183
HCEG_05184
HCEG_05185
HCEG_05186
HCEG_05187
HCEG_05188
2520000
2530000
2540000
2550000
2560000
Position on Supercontig_2.4

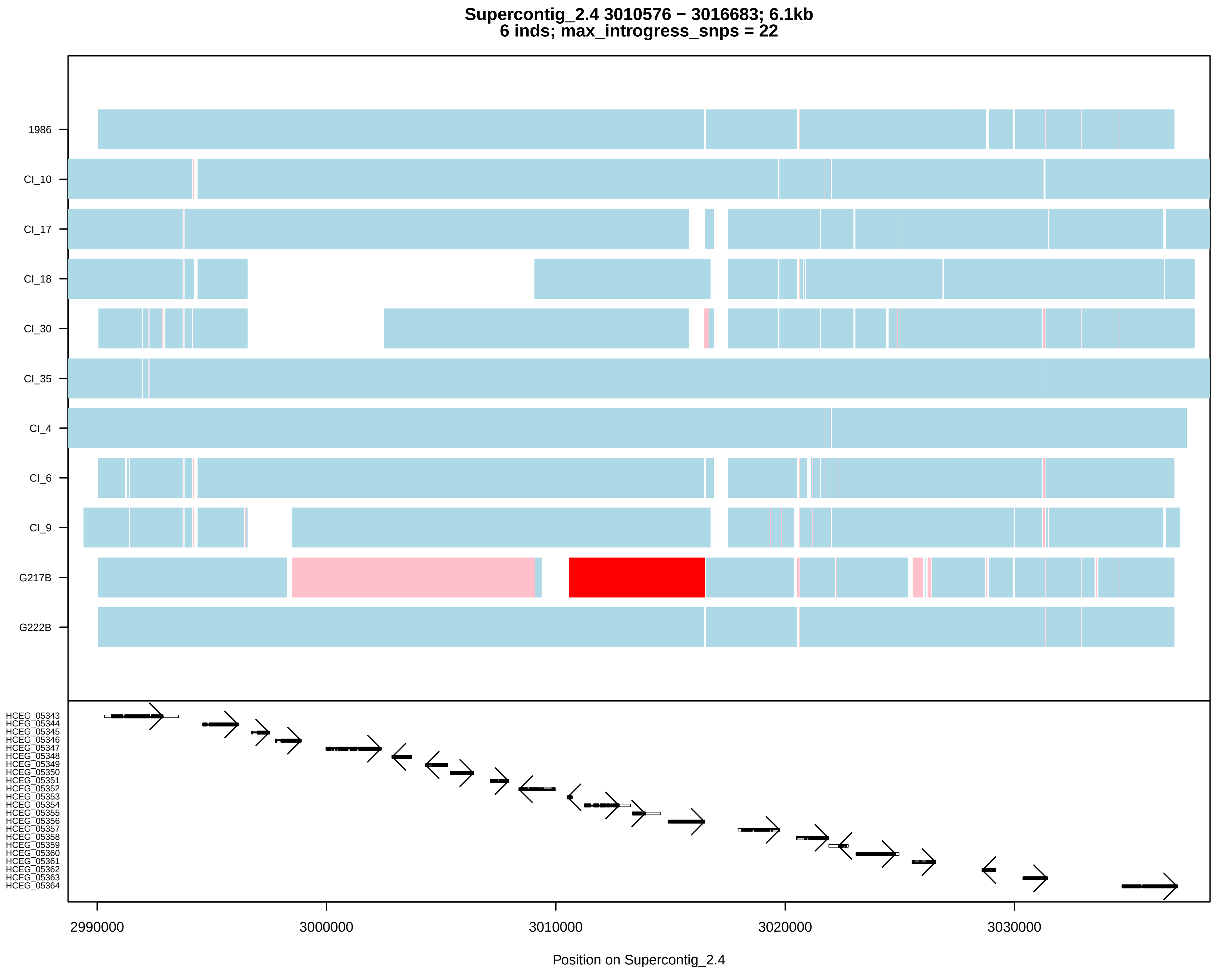
Supercontig_2.4 3010576 − 3016683; 6.1kb
6 inds; max_introgress_snps = 22
1986
CI_10
CI_17
CI_18
CI_30
CI_35
CI_4
CI_6
CI_9
G217B
G222B
HCEG_05343
HCEG_05344
HCEG_05345
HCEG_05346
HCEG_05347
HCEG_05348
HCEG_05349
HCEG_05350
HCEG_05351
HCEG_05352
HCEG_05353
HCEG_05354
HCEG_05355
HCEG_05356
HCEG_05357
HCEG_05358
HCEG_05359
HCEG_05360
HCEG_05361
HCEG_05362
HCEG_05363
HCEG_05364
2990000
3000000
3010000
3020000
3030000
Position on Supercontig_2.4

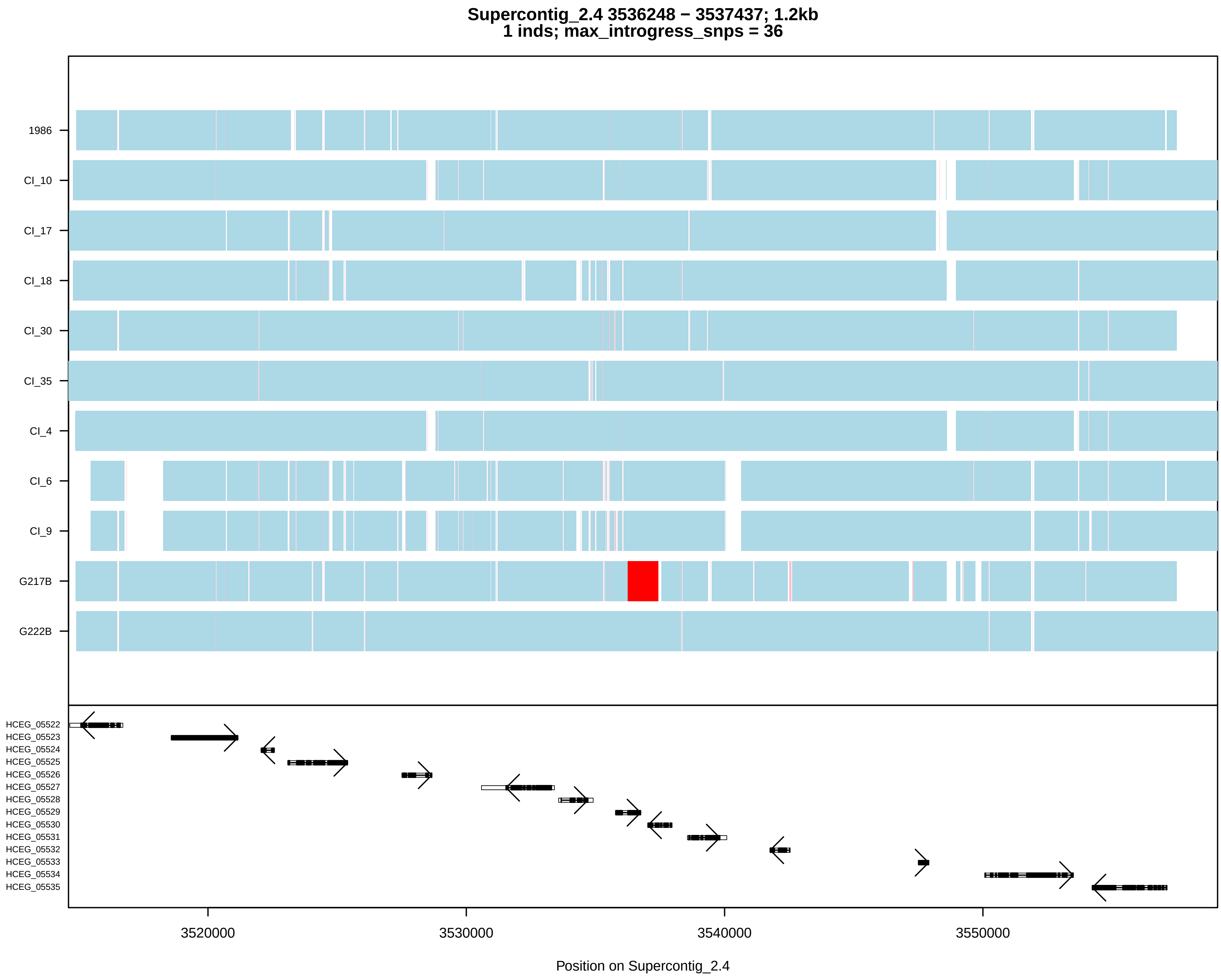
Supercontig_2.4 3536248 − 3537437; 1.2kb
1 inds; max_introgress_snps = 36
1986
CI_10
CI_17
CI_18
CI_30
CI_35
CI_4
CI_6
CI_9
G217B
G222B
HCEG_05522
HCEG_05523
HCEG_05524
HCEG_05525
HCEG_05526
HCEG_05527
HCEG_05528
HCEG_05529
HCEG_05530
HCEG_05531
HCEG_05532
HCEG_05533
HCEG_05534
HCEG_05535
3520000
3530000
3540000
3550000
Position on Supercontig_2.4

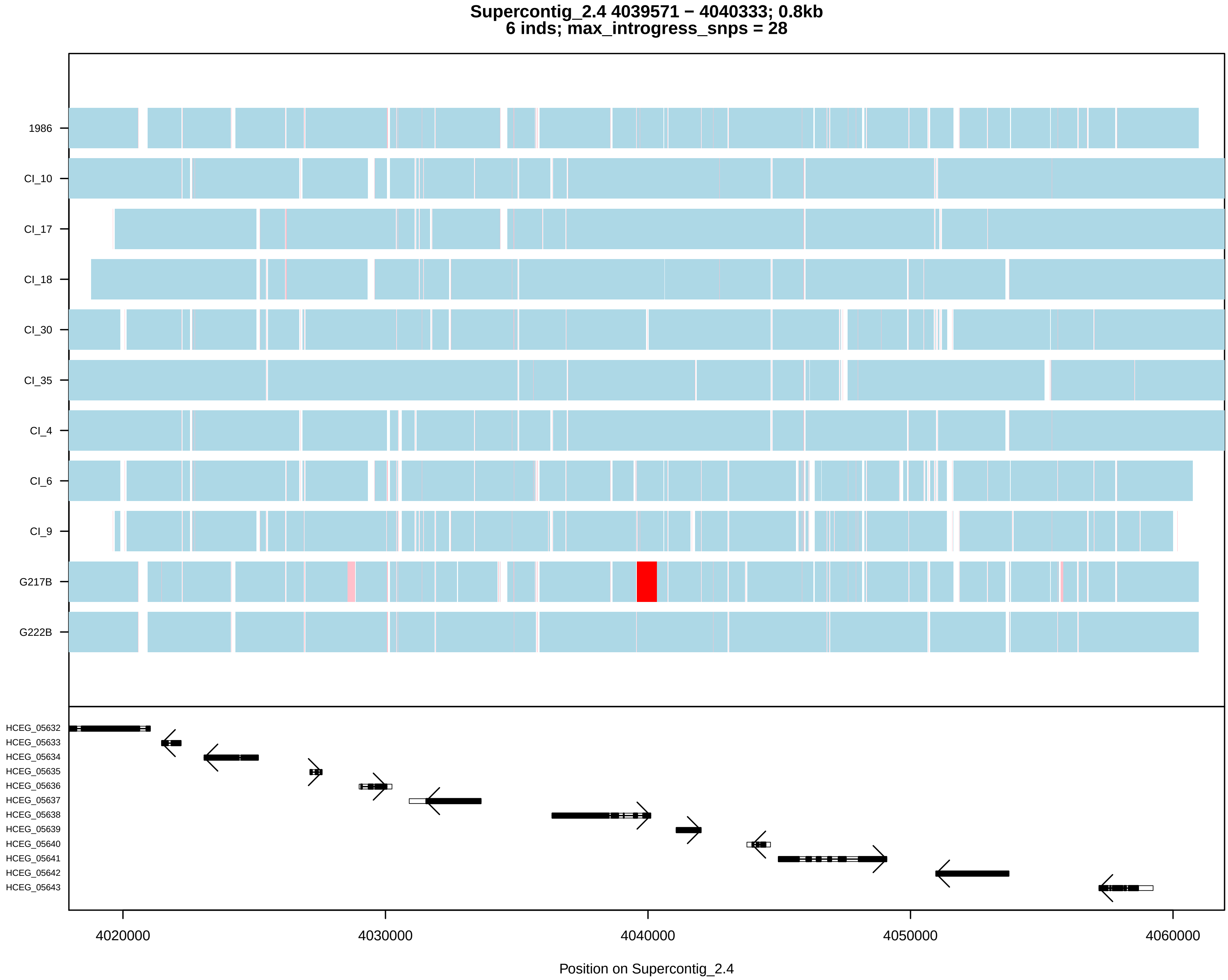
Supercontig_2.4 4039571 − 4040333; 0.8kb
6 inds; max_introgress_snps = 28
1986
CI_10
CI_17
CI_18
CI_30
CI_35
CI_4
CI_6
CI_9
G217B
G222B
HCEG_05632
HCEG_05633
HCEG_05634
HCEG_05635
HCEG_05636
HCEG_05637
HCEG_05638
HCEG_05639
HCEG_05640
HCEG_05641
HCEG_05642
HCEG_05643
4020000
4030000
4040000
4050000
4060000
Position on Supercontig_2.4

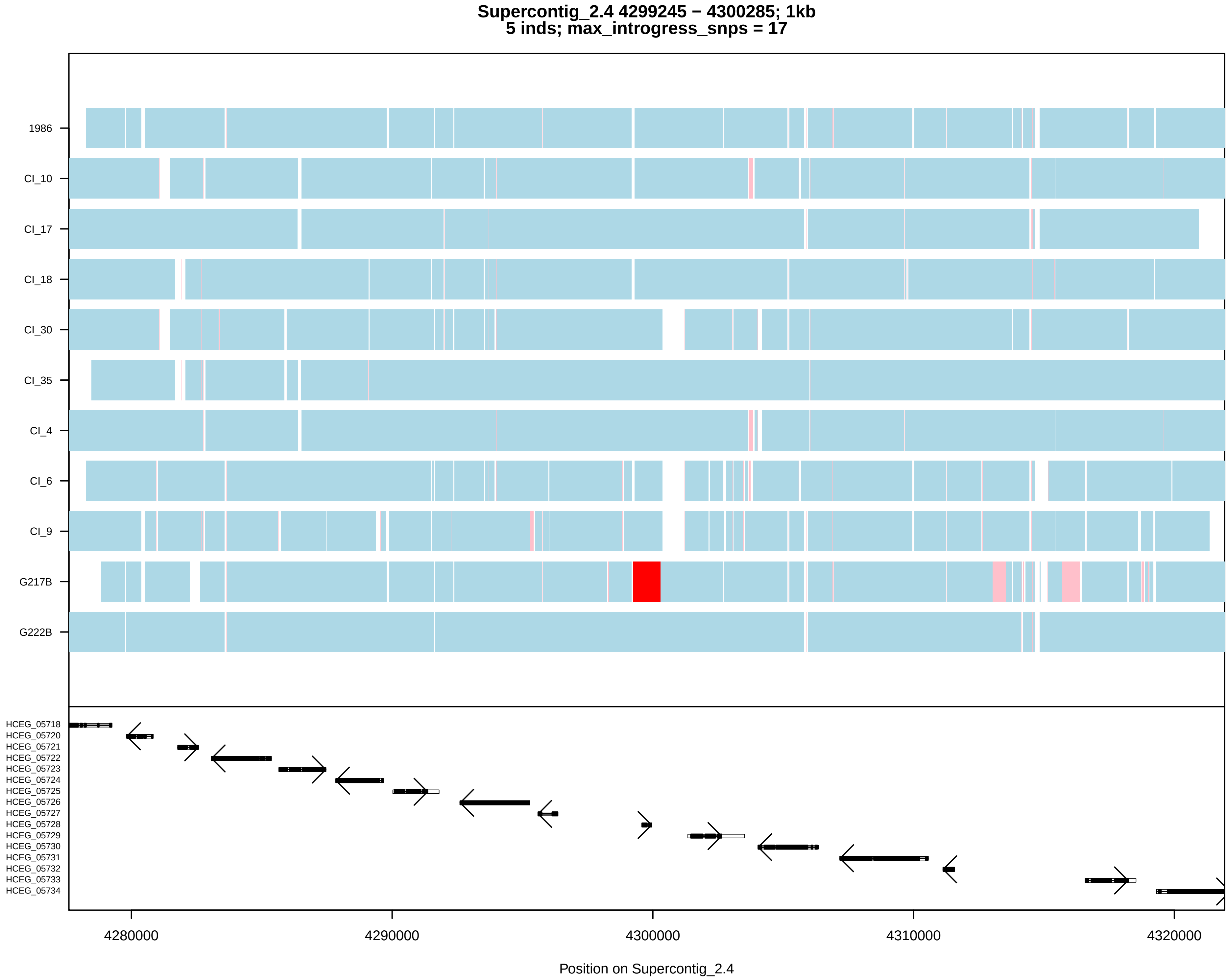

Supercontig_2.4 4299245 − 4300285; 1kb
5 inds; max_introgress_snps = 17
1986
CI_10
CI_17
CI_18
CI_30
CI_35
CI_4
CI_6
CI_9
G217B
G222B
HCEG_05718
HCEG_05720
HCEG_05721
HCEG_05722
HCEG_05723
HCEG_05724
HCEG_05725
HCEG_05726
HCEG_05727
HCEG_05728
HCEG_05729
HCEG_05730
HCEG_05731
HCEG_05732
HCEG_05733
HCEG_05734
4280000
4290000
4300000
4310000
4320000
Position on Supercontig_2.4

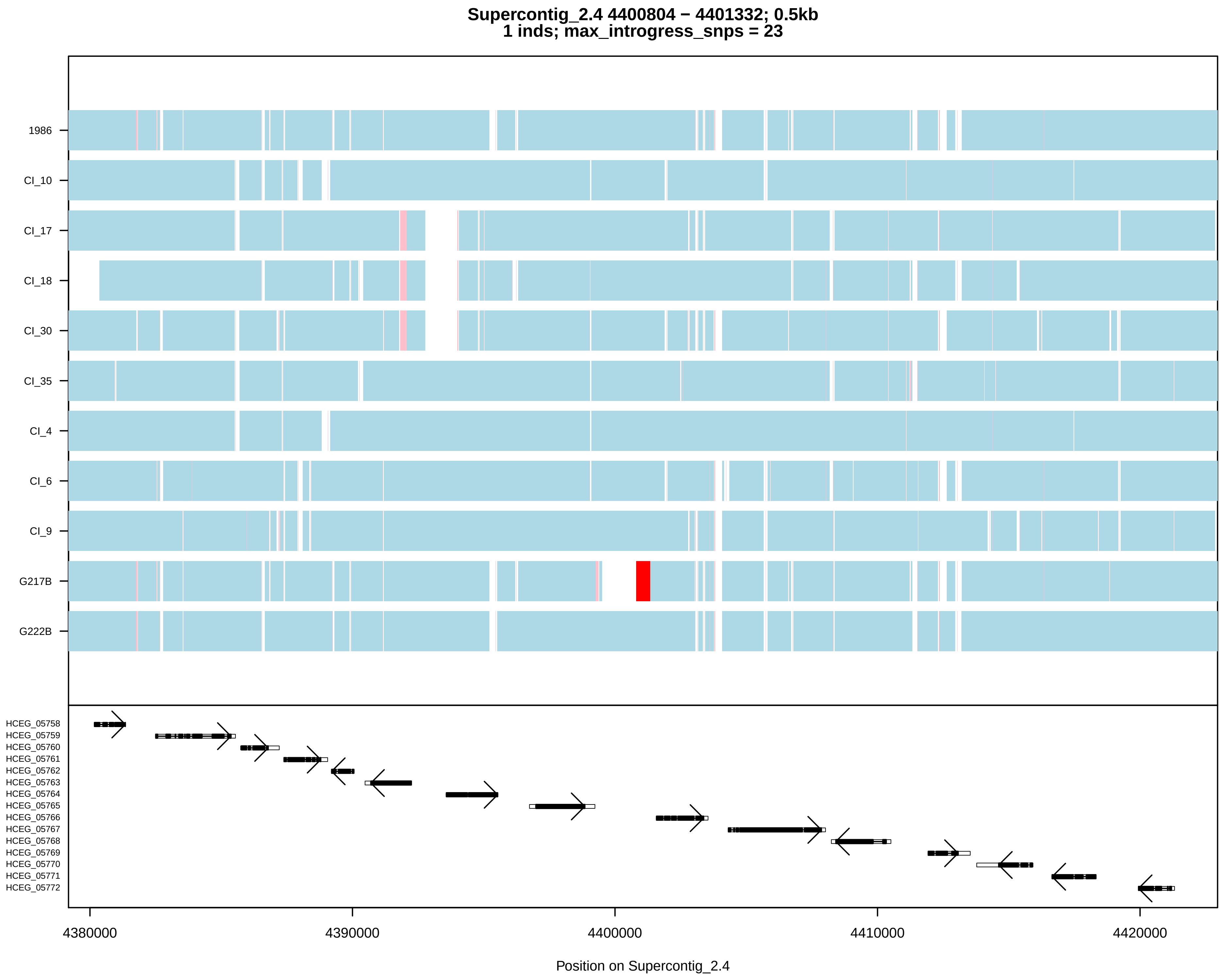

Supercontig_2.4 4400804 − 4401332; 0.5kb
1 inds; max_introgress_snps = 23
1986
CI_10
CI_17
CI_18
CI_30
CI_35
CI_4
CI_6
CI_9
G217B
G222B
HCEG_05758
HCEG_05759
HCEG_05760
HCEG_05761
HCEG_05762
HCEG_05763
HCEG_05764
HCEG_05765
HCEG_05766
HCEG_05767
HCEG_05768
HCEG_05769
HCEG_05770
HCEG_05771
HCEG_05772
4380000
4390000
4400000
4410000
4420000
Position on Supercontig_2.4

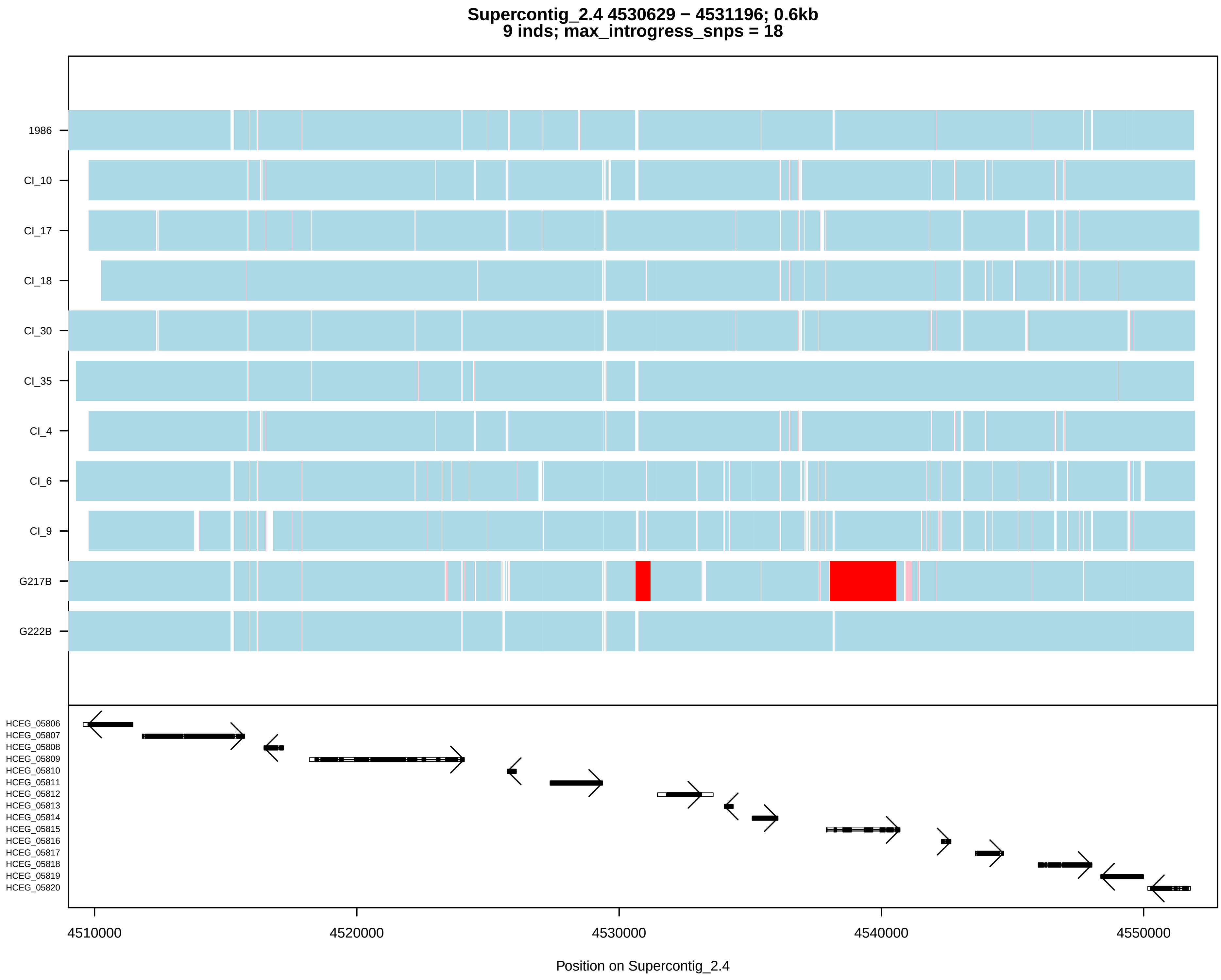

Supercontig_2.4 4530629 − 4531196; 0.6kb
9 inds; max_introgress_snps = 18
1986
CI_10
CI_17
CI_18
CI_30
CI_35
CI_4
CI_6
CI_9
G217B
G222B
HCEG_05806
HCEG_05807
HCEG_05808
HCEG_05809
HCEG_05810
HCEG_05811
HCEG_05812
HCEG_05813
HCEG_05814
HCEG_05815
HCEG_05816
HCEG_05817
HCEG_05818
HCEG_05819
HCEG_05820
4510000
4520000
4530000
4540000
4550000
Position on Supercontig_2.4

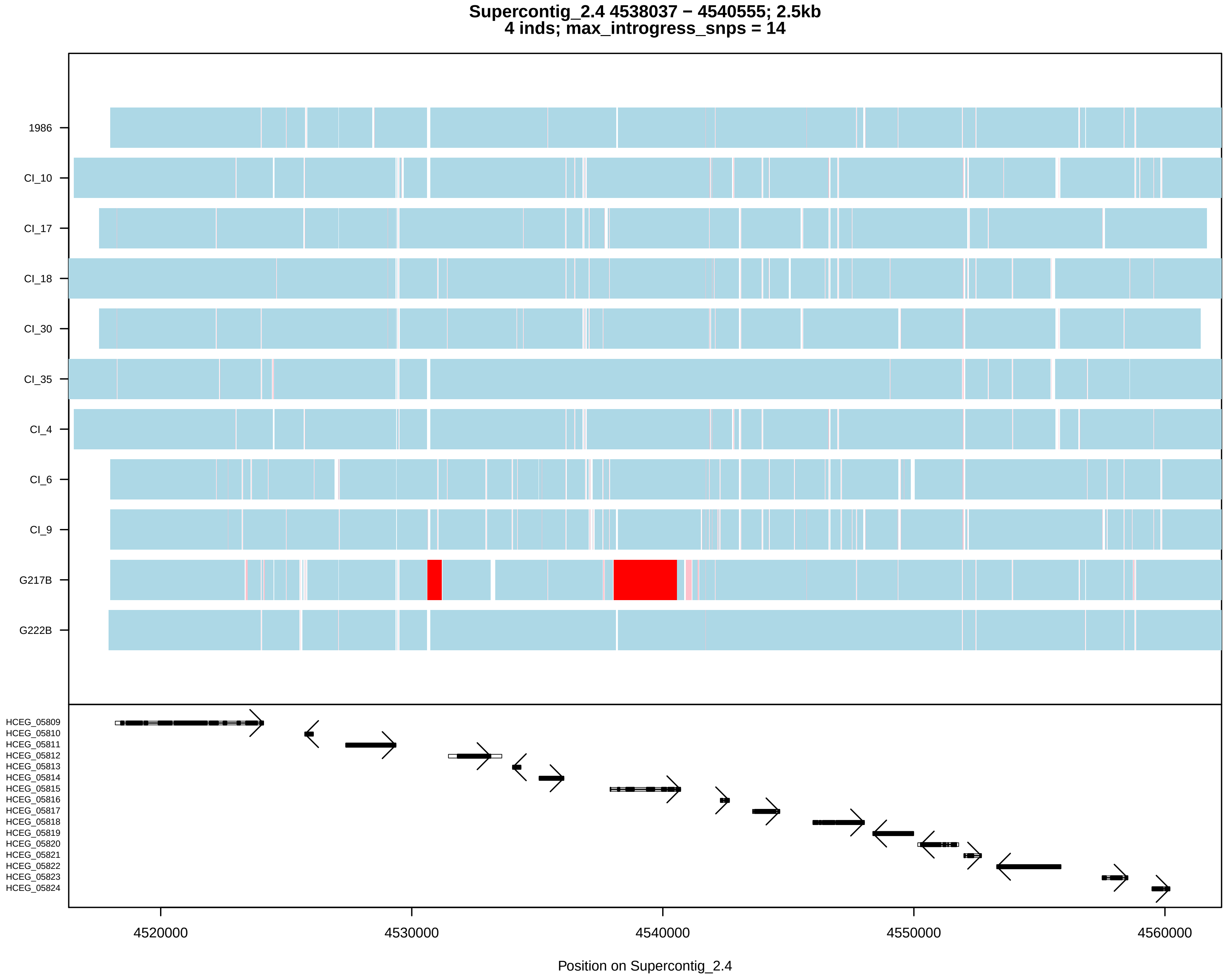
Supercontig_2.4 4538037 − 4540555; 2.5kb
4 inds; max_introgress_snps = 14
1986
CI_10
CI_17
CI_18
CI_30
CI_35
CI_4
CI_6
CI_9
G217B
G222B
HCEG_05809
HCEG_05810
HCEG_05811
HCEG_05812
HCEG_05813
HCEG_05814
HCEG_05815
HCEG_05816
HCEG_05817
HCEG_05818
HCEG_05819
HCEG_05820
HCEG_05821
HCEG_05822
HCEG_05823
HCEG_05824
4520000
4530000
4540000
4550000
4560000
Position on Supercontig_2.4

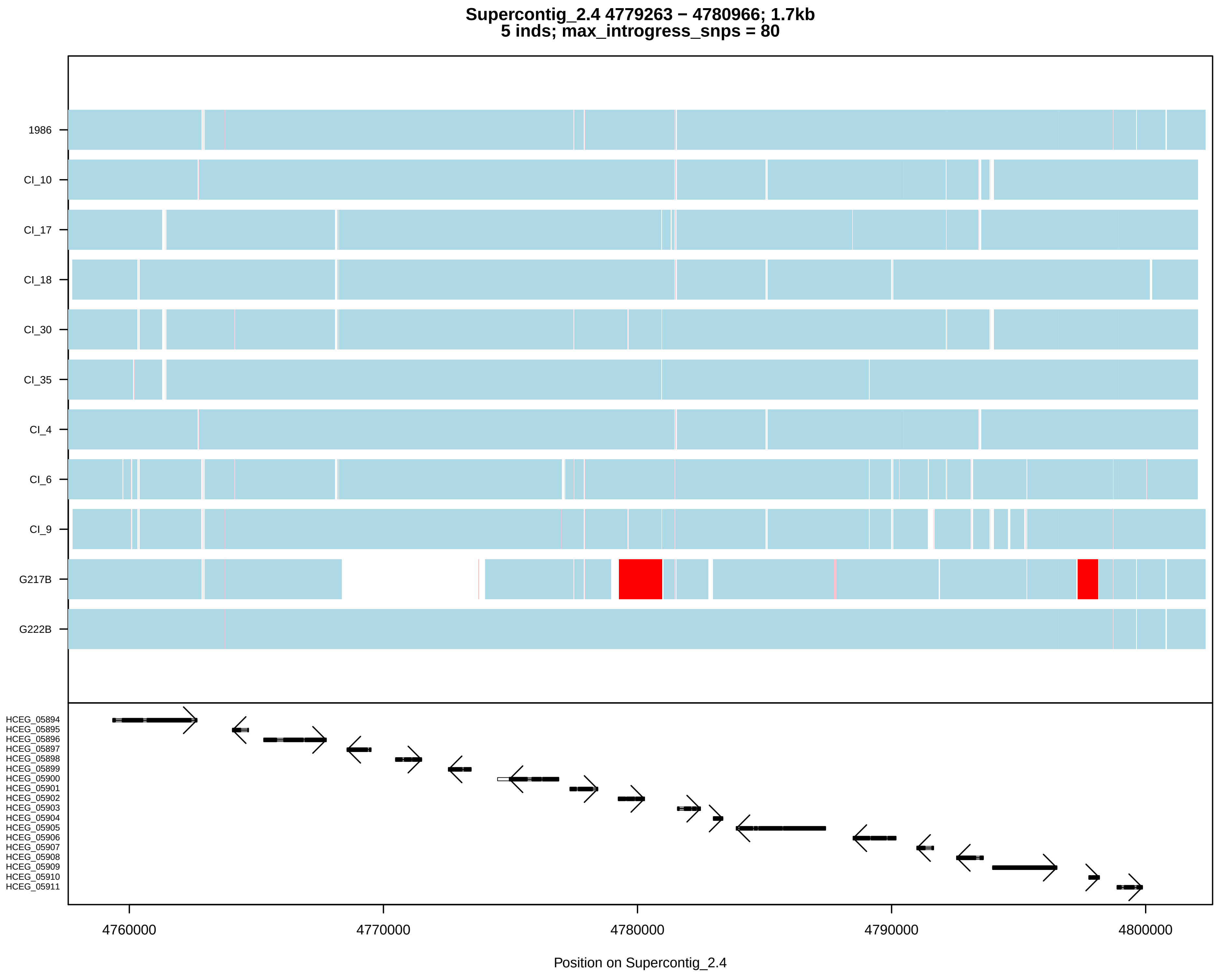
Supercontig_2.4 4779263 − 4780966; 1.7kb
5 inds; max_introgress_snps = 80
1986
CI_10
CI_17
CI_18
CI_30
CI_35
CI_4
CI_6
CI_9
G217B
G222B
HCEG_05894
HCEG_05895
HCEG_05896
HCEG_05897
HCEG_05898
HCEG_05899
HCEG_05900
HCEG_05901
HCEG_05902
HCEG_05903
HCEG_05904
HCEG_05905
HCEG_05906
HCEG_05907
HCEG_05908
HCEG_05909
HCEG_05910
HCEG_05911
4760000
4770000
4780000
4790000
4800000
Position on Supercontig_2.4

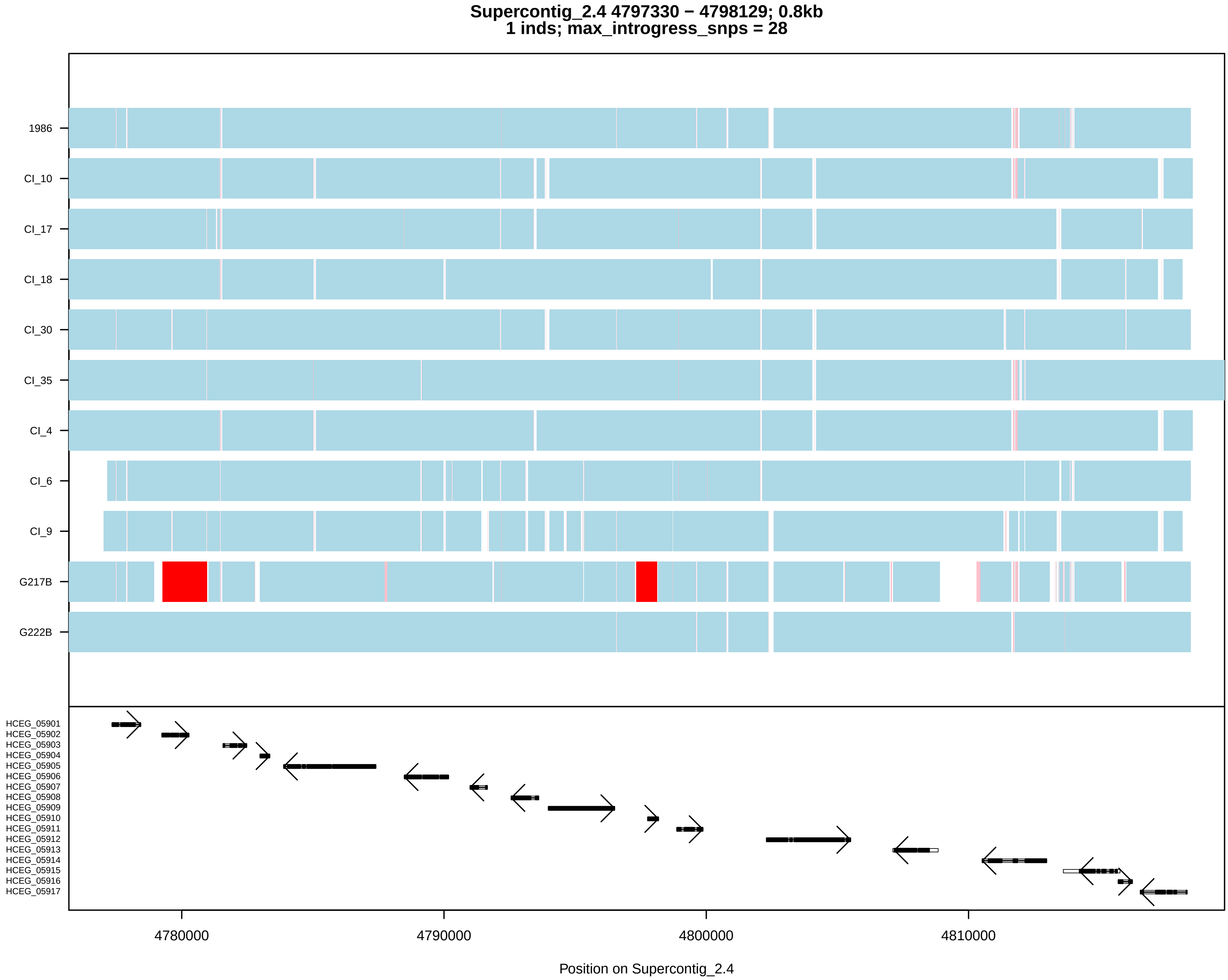

Supercontig_2.4 4797330 − 4798129; 0.8kb
1 inds; max_introgress_snps = 28
1986
CI_10
CI_17
CI_18
CI_30
CI_35
CI_4
CI_6
CI_9
G217B
G222B
HCEG_05901
HCEG_05902
HCEG_05903
HCEG_05904
HCEG_05905
HCEG_05906
HCEG_05907
HCEG_05908
HCEG_05909
HCEG_05910
HCEG_05911
HCEG_05912
HCEG_05913
HCEG_05914
HCEG_05915
HCEG_05916
HCEG_05917
4780000
4790000
4800000
4810000
Position on Supercontig_2.4

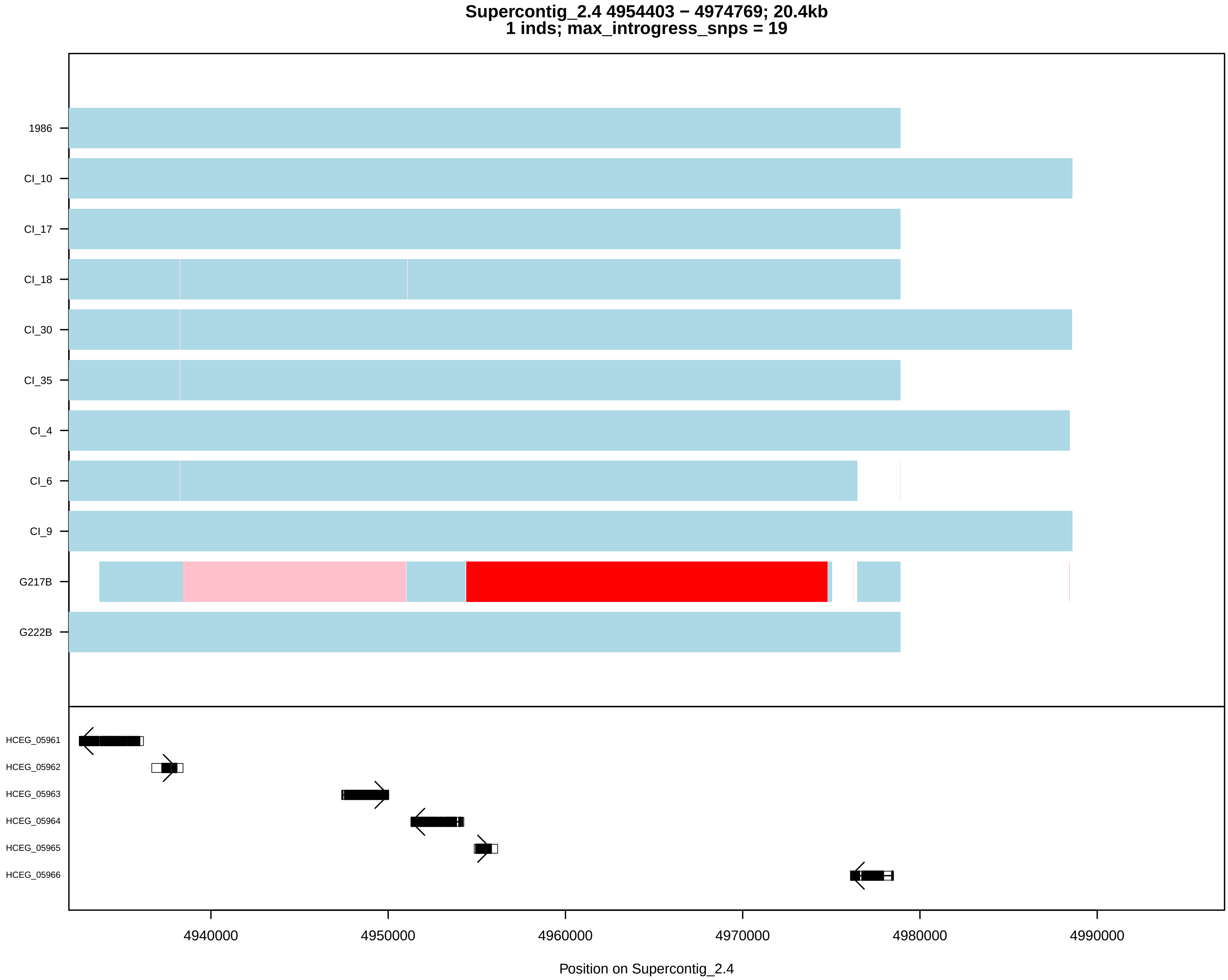

Supercontig_2.4 4954403 − 4974769; 20.4kb
1 inds; max_introgress_snps = 19
1986
CI_10
CI_17
CI_18
CI_30
CI_35
CI_4
CI_6
CI_9
G217B
G222B
HCEG_05961
HCEG_05962
HCEG_05963
HCEG_05964
HCEG_05965
HCEG_05966
4940000
4950000
4960000
4970000
4980000
4990000
Position on Supercontig_2.4

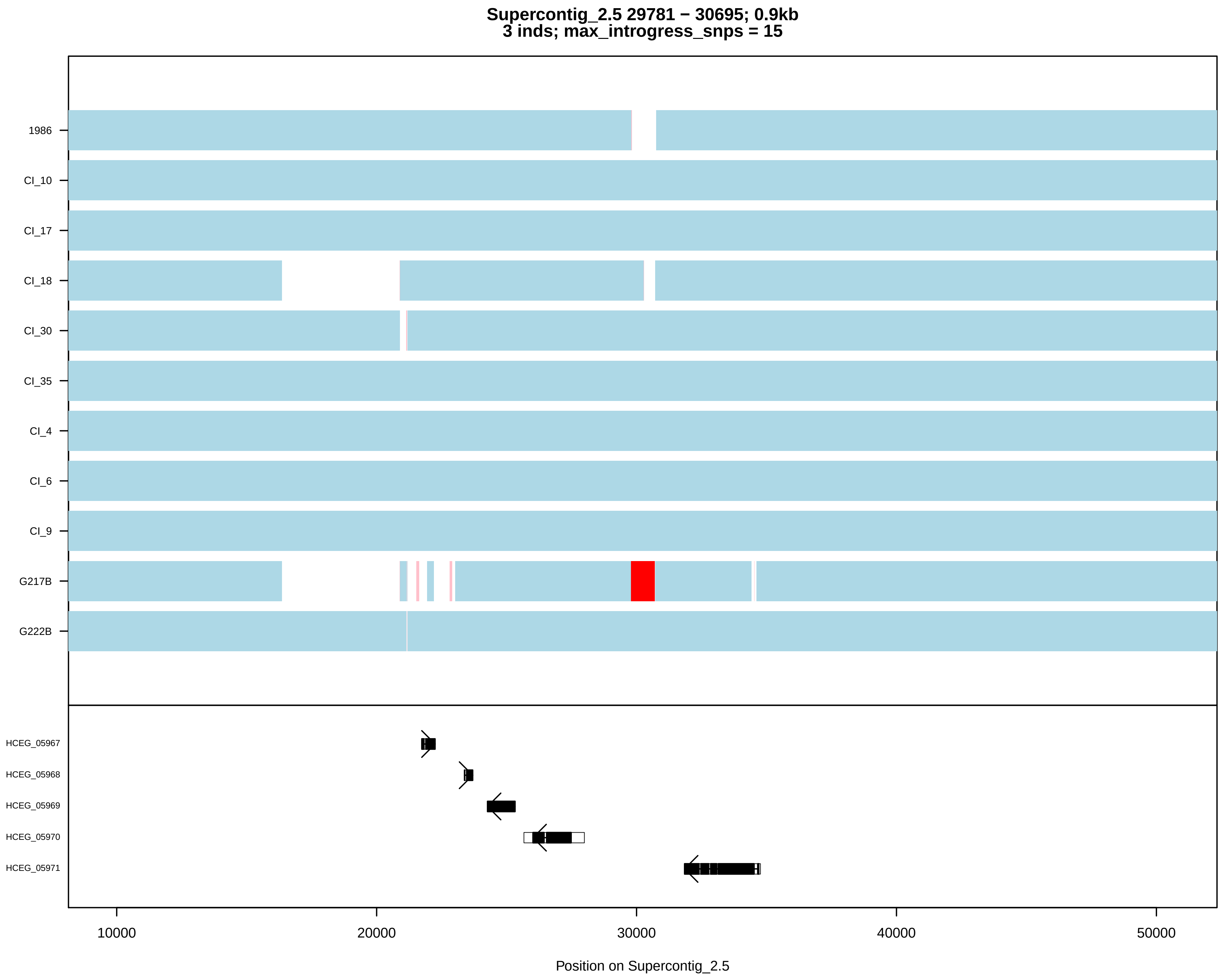

Supercontig_2.5 29781 − 30695; 0.9kb
3 inds; max_introgress_snps = 15
1986
CI_10
CI_17
CI_18
CI_30
CI_35
CI_4
CI_6
CI_9
G217B
G222B
HCEG_05967
HCEG_05968
HCEG_05969
HCEG_05970
HCEG_05971
10000
20000
30000
40000
50000
Position on Supercontig_2.5

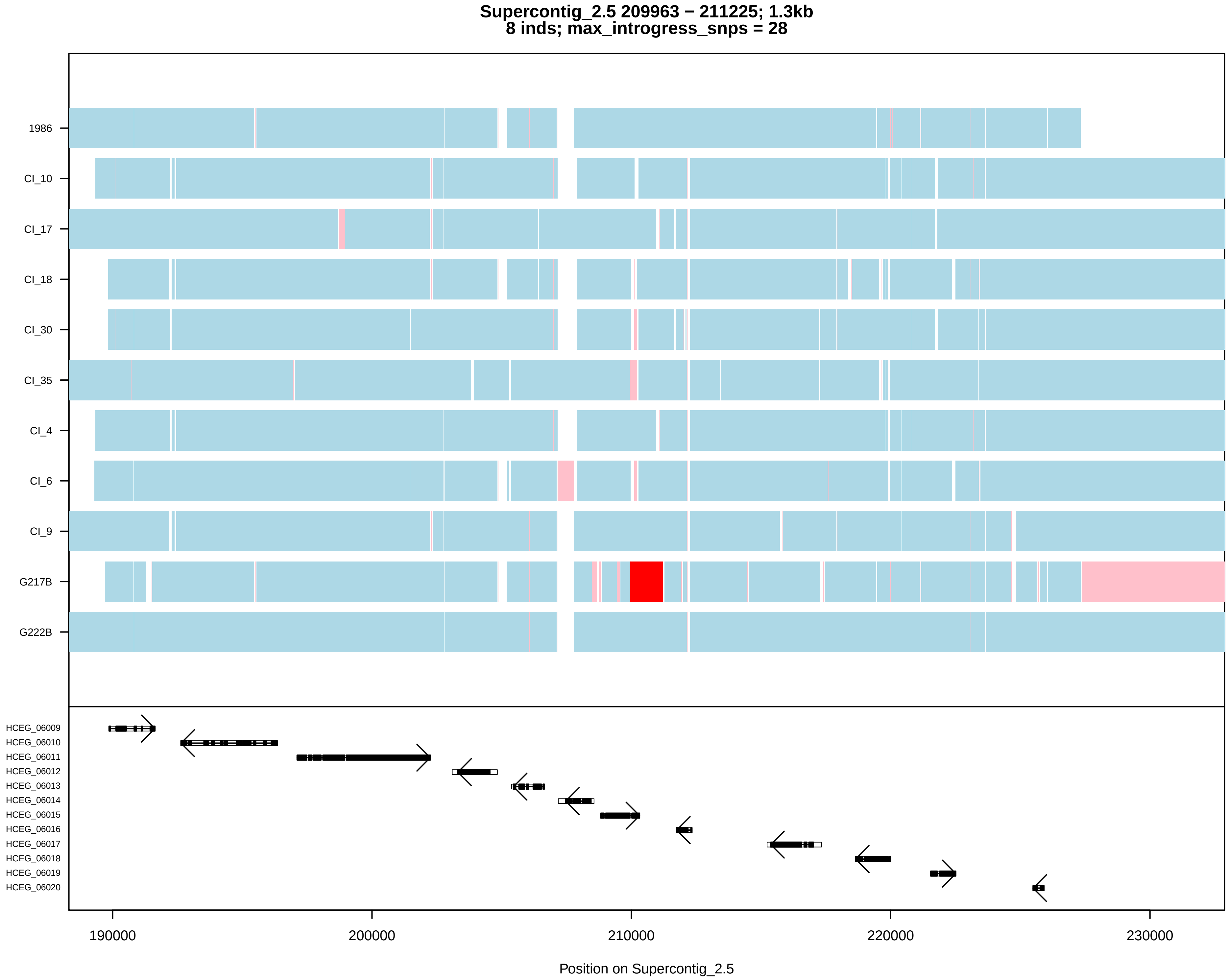

Supercontig_2.5 209963 − 211225; 1.3kb
8 inds; max_introgress_snps = 28
1986
CI_10
CI_17
CI_18
CI_30
CI_35
CI_4
CI_6
CI_9
G217B
G222B
HCEG_06009
HCEG_06010
HCEG_06011
HCEG_06012
HCEG_06013
HCEG_06014
HCEG_06015
HCEG_06016
HCEG_06017
HCEG_06018
HCEG_06019
HCEG_06020
190000
200000
210000
220000
230000
Position on Supercontig_2.5

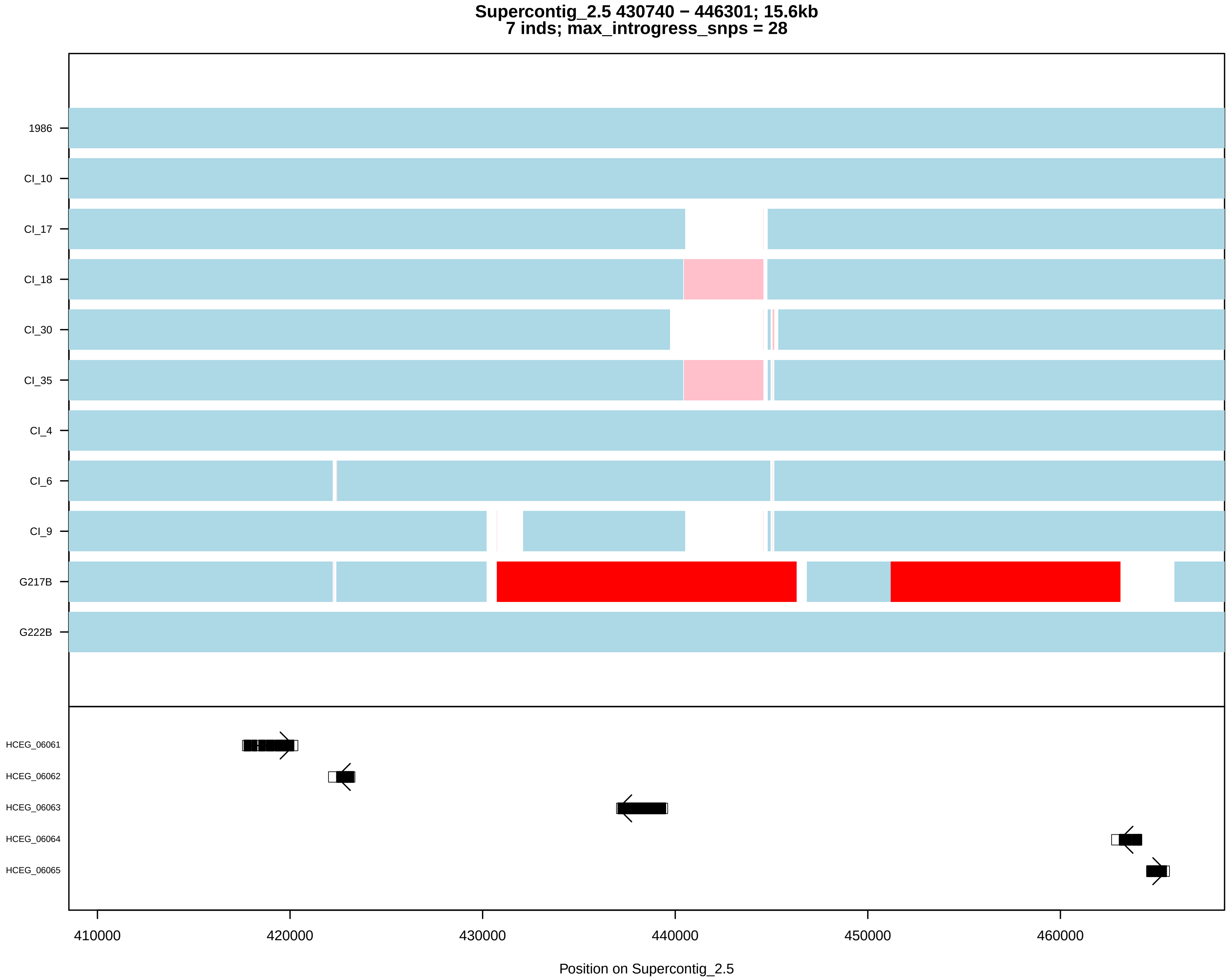

Supercontig_2.5 430740 − 446301; 15.6kb
7 inds; max_introgress_snps = 28
1986
CI_10
CI_17
CI_18
CI_30
CI_35
CI_4
CI_6
CI_9
G217B
G222B
HCEG_06061
HCEG_06062
HCEG_06063
HCEG_06064
HCEG_06065
410000
420000
430000
440000
450000
460000
Position on Supercontig_2.5

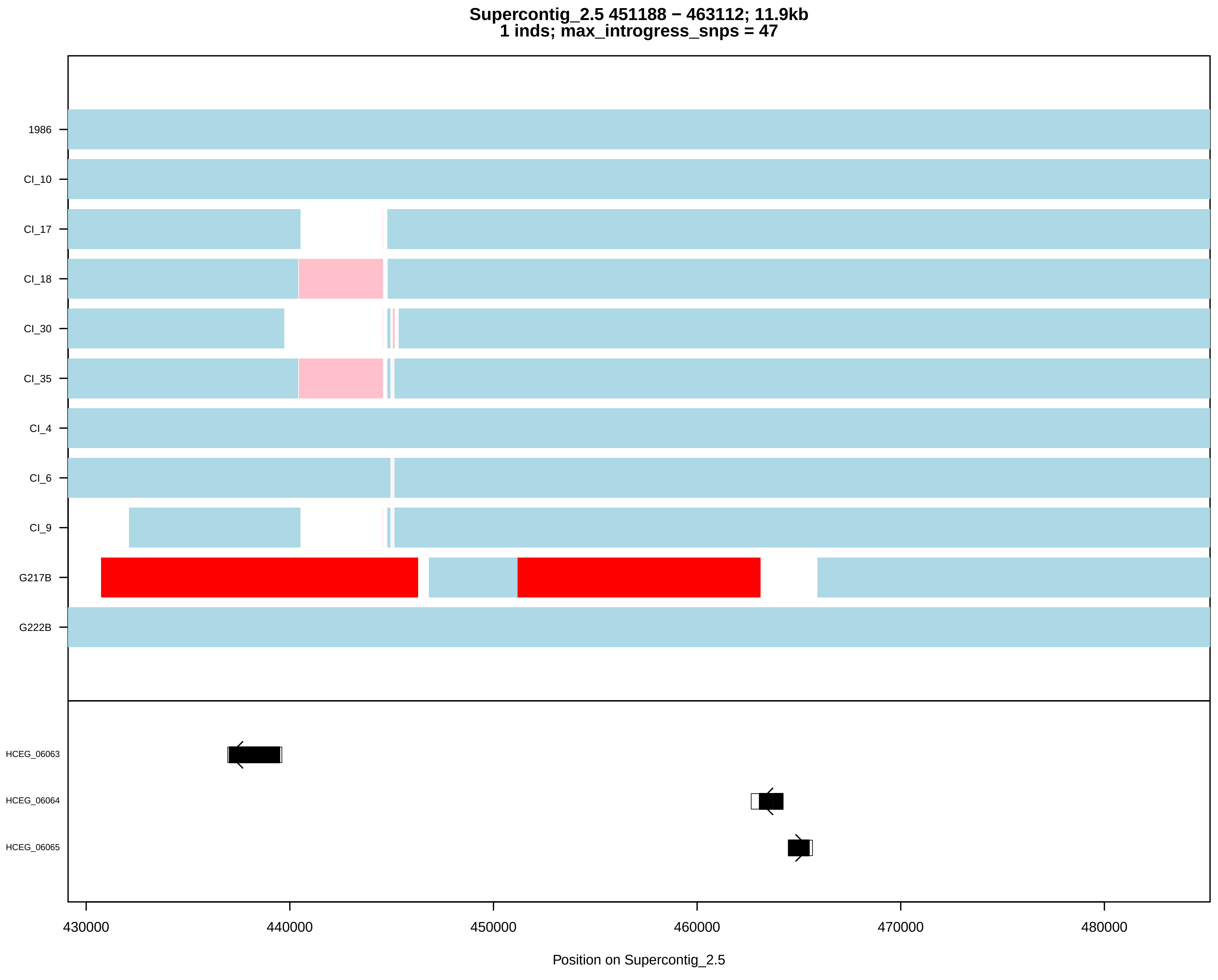
Supercontig_2.5 451188 − 463112; 11.9kb
1 inds; max_introgress_snps = 47
1986
CI_10
CI_17
CI_18
CI_30
CI_35
CI_4
CI_6
CI_9
G217B
G222B
HCEG_06063
HCEG_06064
HCEG_06065
430000
440000
450000
460000
470000
480000
Position on Supercontig_2.5

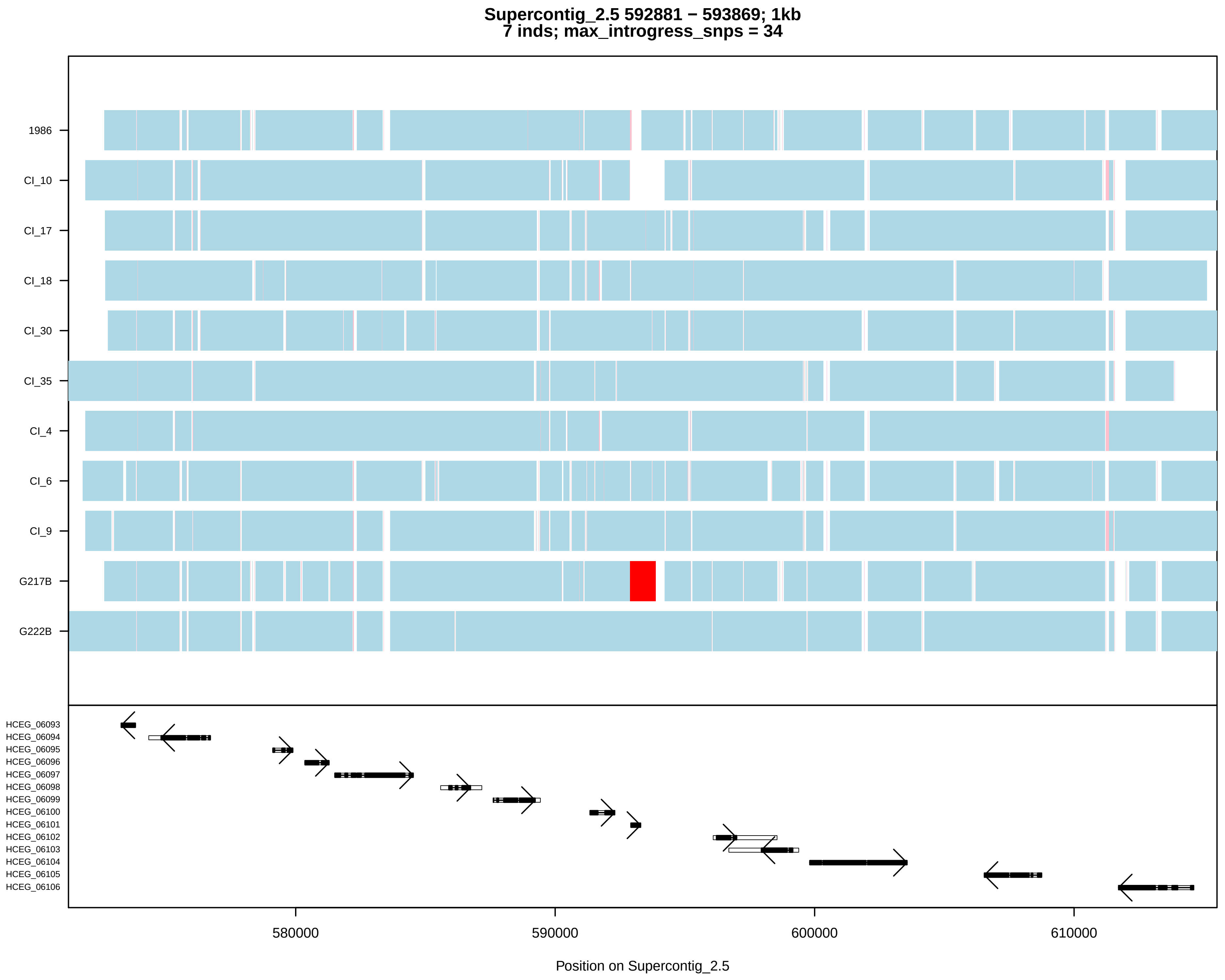
Supercontig_2.5 592881 − 593869; 1kb
7 inds; max_introgress_snps = 34
1986
CI_10
CI_17
CI_18
CI_30
CI_35
CI_4
CI_6
CI_9
G217B
G222B
HCEG_06093
HCEG_06094
HCEG_06095
HCEG_06096
HCEG_06097
HCEG_06098
HCEG_06099
HCEG_06100
HCEG_06101
HCEG_06102
HCEG_06103
HCEG_06104
HCEG_06105
HCEG_06106
580000
590000
600000
610000
Position on Supercontig_2.5

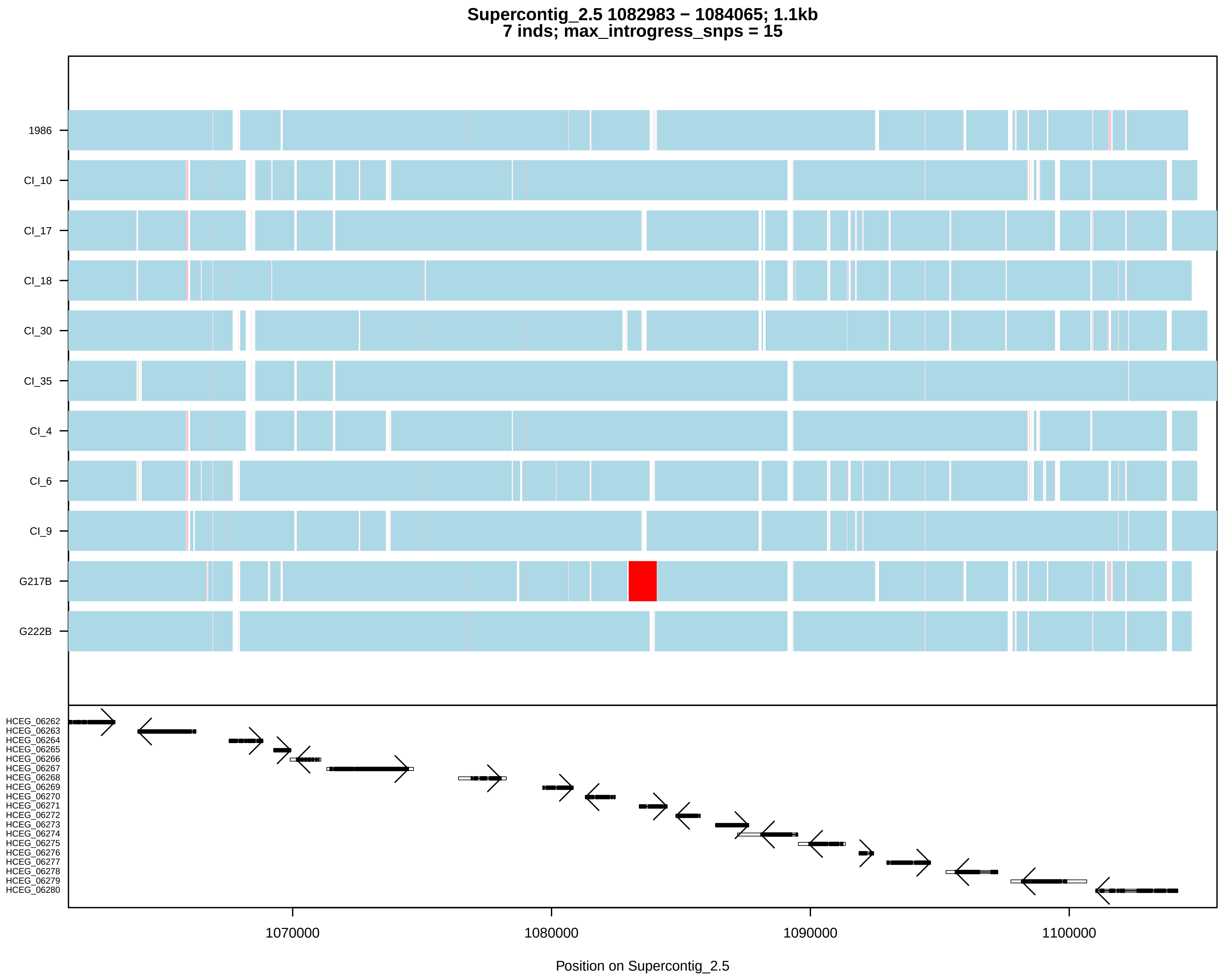

Supercontig_2.5 1082983 − 1084065; 1.1kb
7 inds; max_introgress_snps = 15
1986
CI_10
CI_17
CI_18
CI_30
CI_35
CI_4
CI_6
CI_9
G217B
G222B
HCEG_06262
HCEG_06263
HCEG_06264
HCEG_06265
HCEG_06266
HCEG_06267
HCEG_06268
HCEG_06269
HCEG_06270
HCEG_06271
HCEG_06272
HCEG_06273
HCEG_06274
HCEG_06275
HCEG_06276
HCEG_06277
HCEG_06278
HCEG_06279
HCEG_06280
1070000
1080000
1090000
1100000
Position on Supercontig_2.5

Supercontig_2.5 1220430 − 1222715; 2.3kb
1 inds; max_introgress_snps = 18

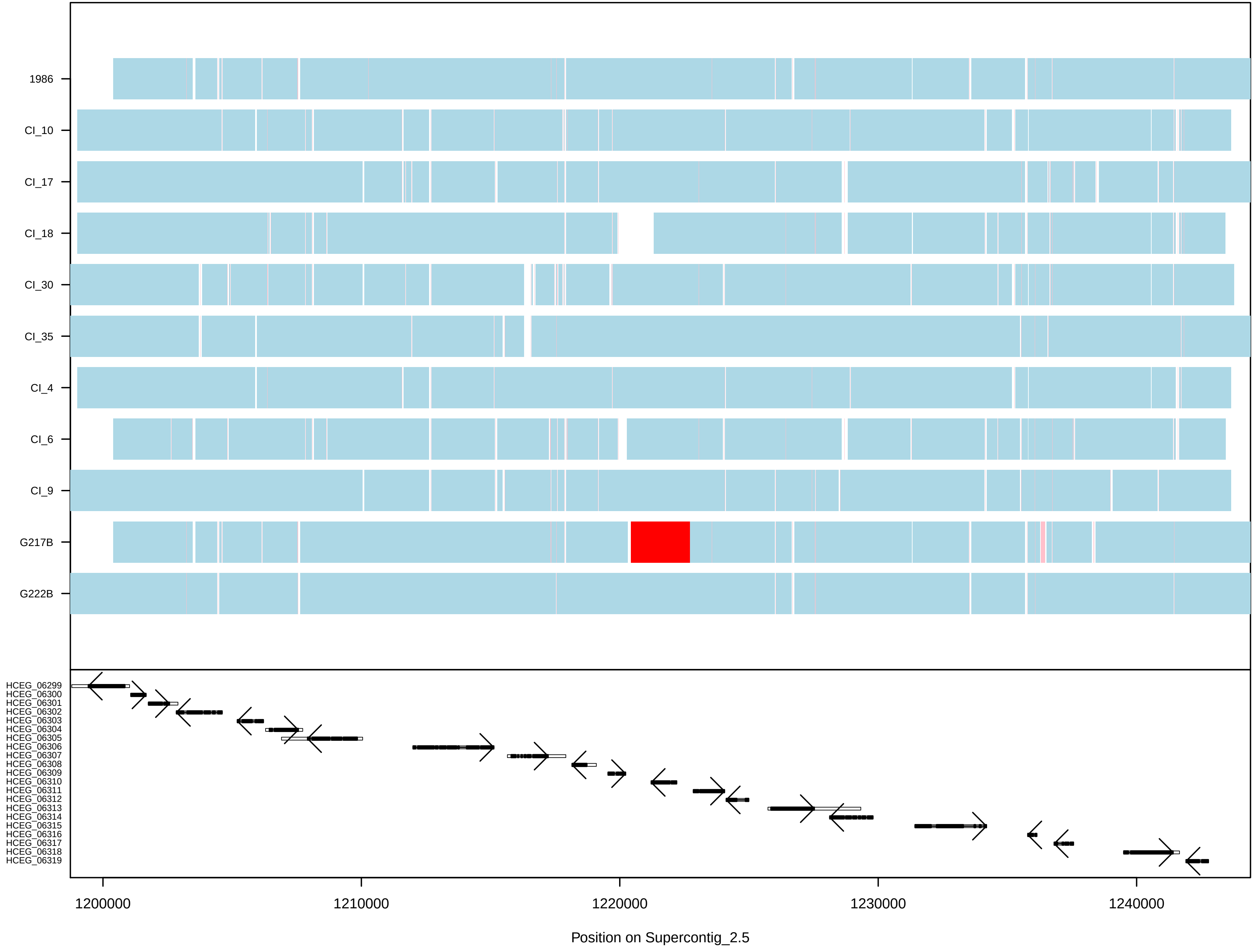

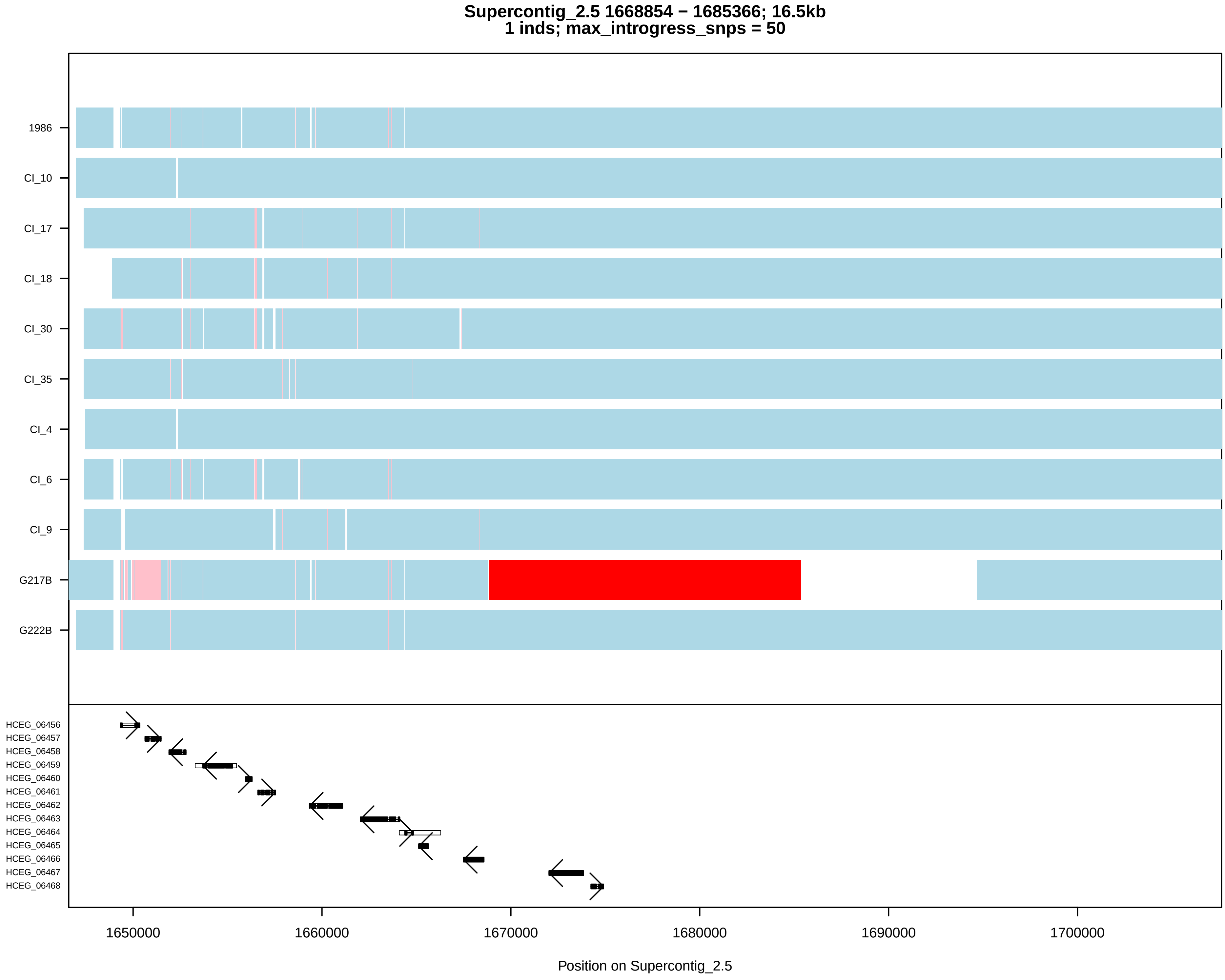
Supercontig_2.5 1668854 − 1685366; 16.5kb
1 inds; max_introgress_snps = 50
1986
CI_10
CI_17
CI_18
CI_30
CI_35
CI_4
CI_6
CI_9
G217B
G222B
HCEG_06456
HCEG_06457
HCEG_06458
HCEG_06459
HCEG_06460
HCEG_06461
HCEG_06462
HCEG_06463
HCEG_06464
HCEG_06465
HCEG_06466
HCEG_06467
HCEG_06468
1650000
1660000
1670000
1680000
1690000
1700000
Position on Supercontig_2.5

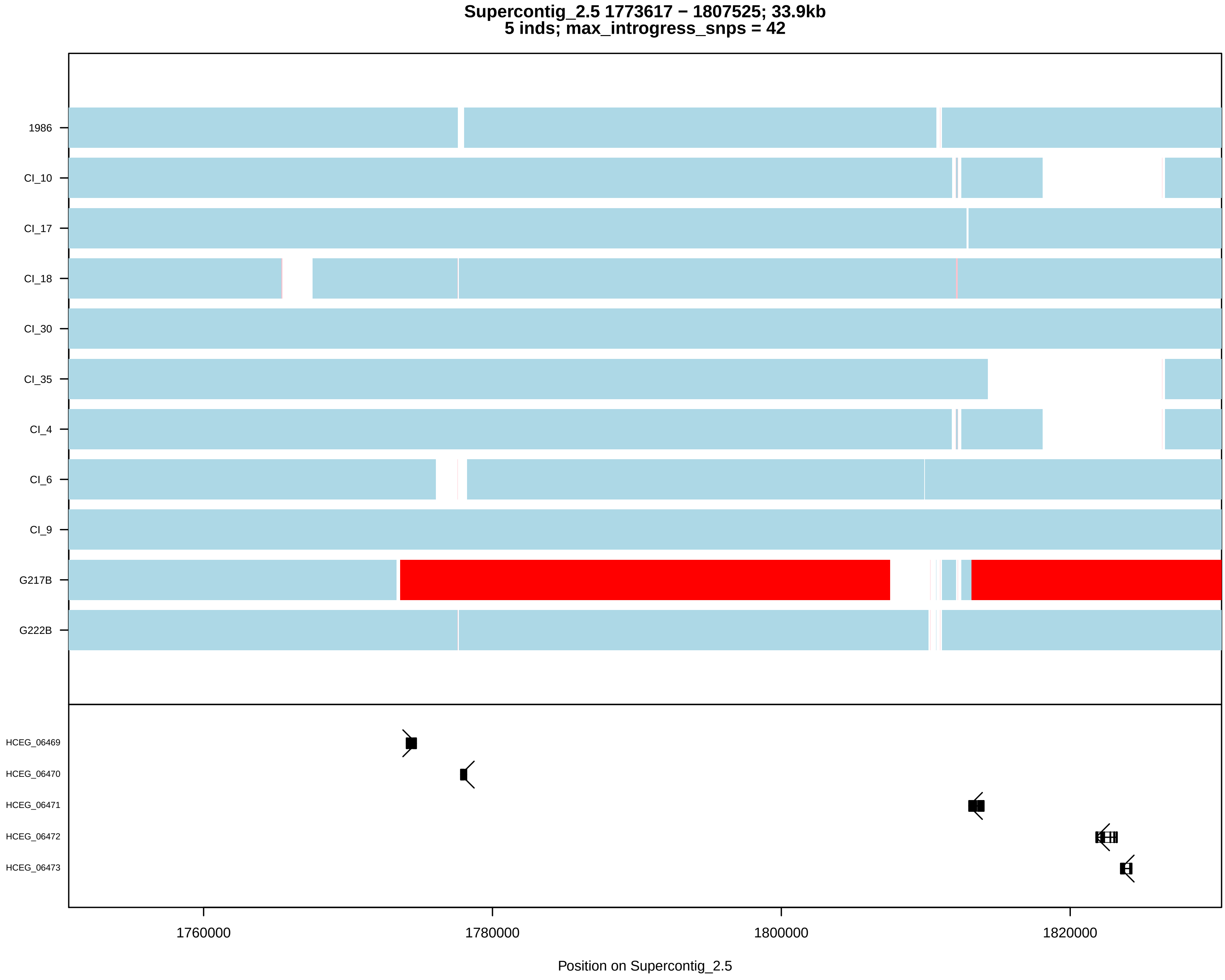

Supercontig_2.5 1773617 − 1807525; 33.9kb
5 inds; max_introgress_snps = 42
1986
CI_10
CI_17
CI_18
CI_30
CI_35
CI_4
CI_6
CI_9
G217B
G222B
HCEG_06469
HCEG_06470
HCEG_06471
HCEG_06472
HCEG_06473
1760000
1780000
1800000
1820000
Position on Supercontig_2.5

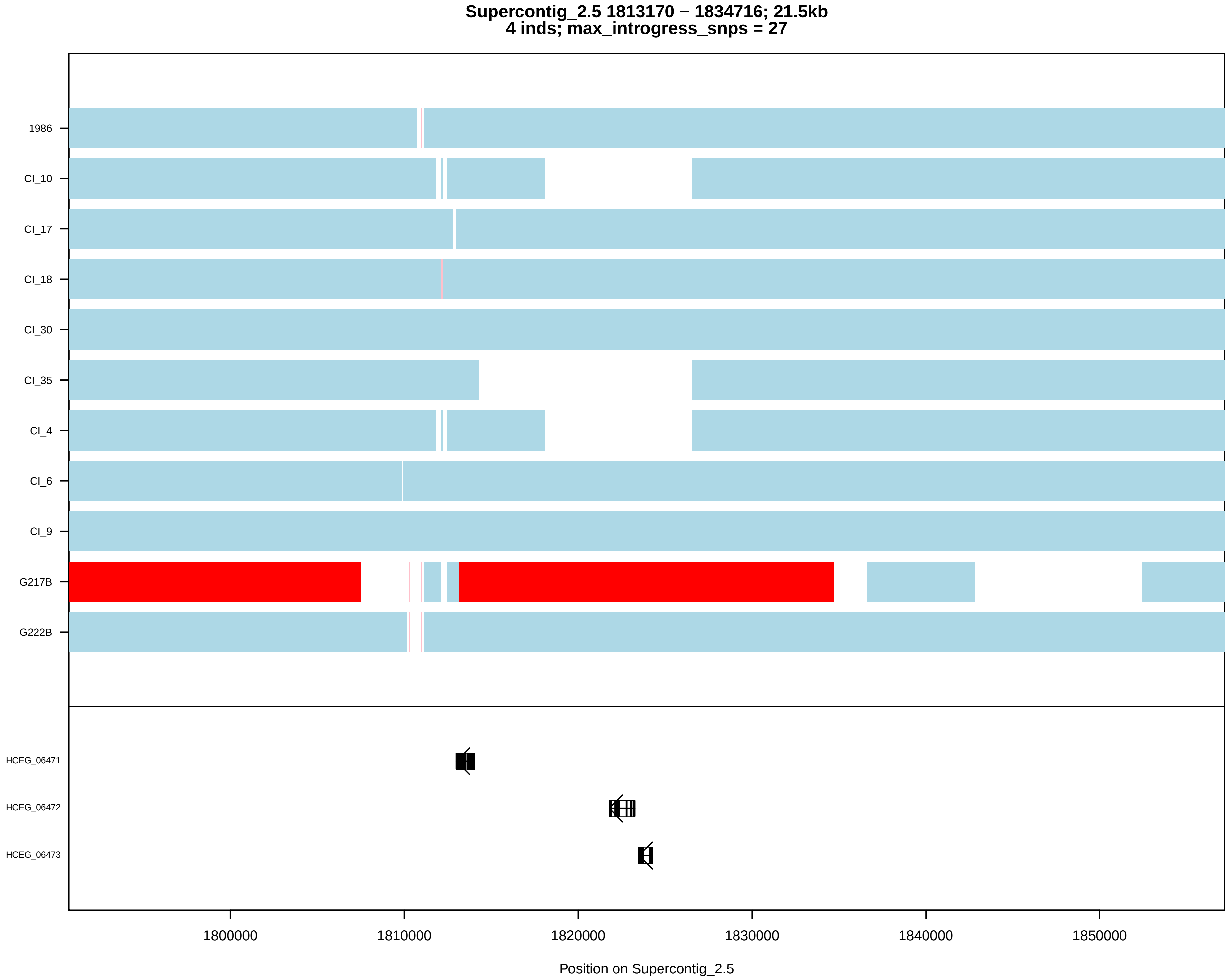

Supercontig_2.5 1813170 − 1834716; 21.5kb
4 inds; max_introgress_snps = 27
1986
CI_10
CI_17
CI_18
CI_30
CI_35
CI_4
CI_6
CI_9
G217B
G222B
HCEG_06471
HCEG_06472
HCEG_06473
1800000
1810000
1820000
1830000
1840000
1850000
Position on Supercontig_2.5

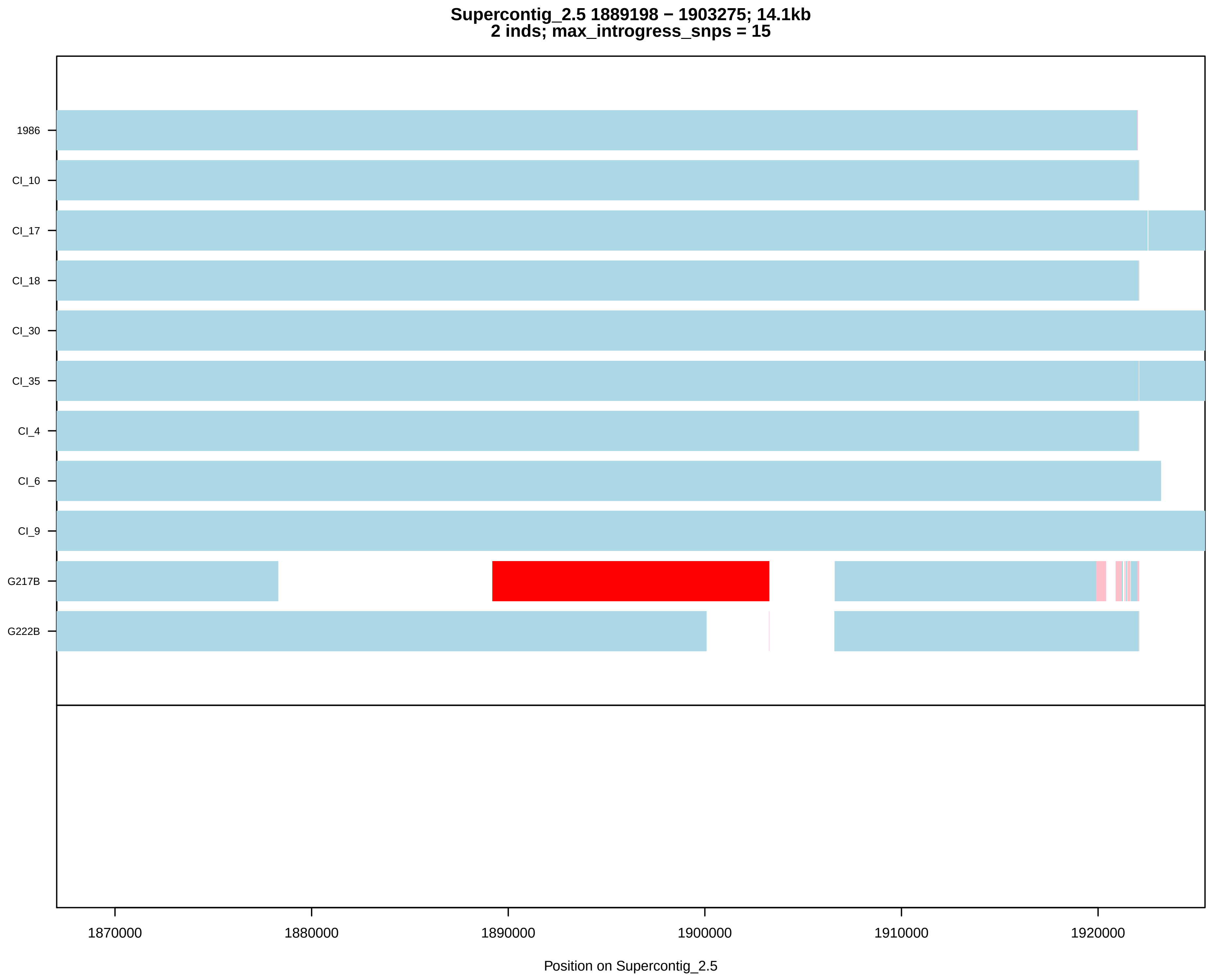

Supercontig_2.5 1889198 − 1903275; 14.1kb
2 inds; max_introgress_snps = 15
1986
CI_10
CI_17
CI_18
CI_30
CI_35
CI_4
CI_6
CI_9
G217B
G222B
1870000
1880000
1890000
1900000
1910000
1920000
Position on Supercontig_2.5

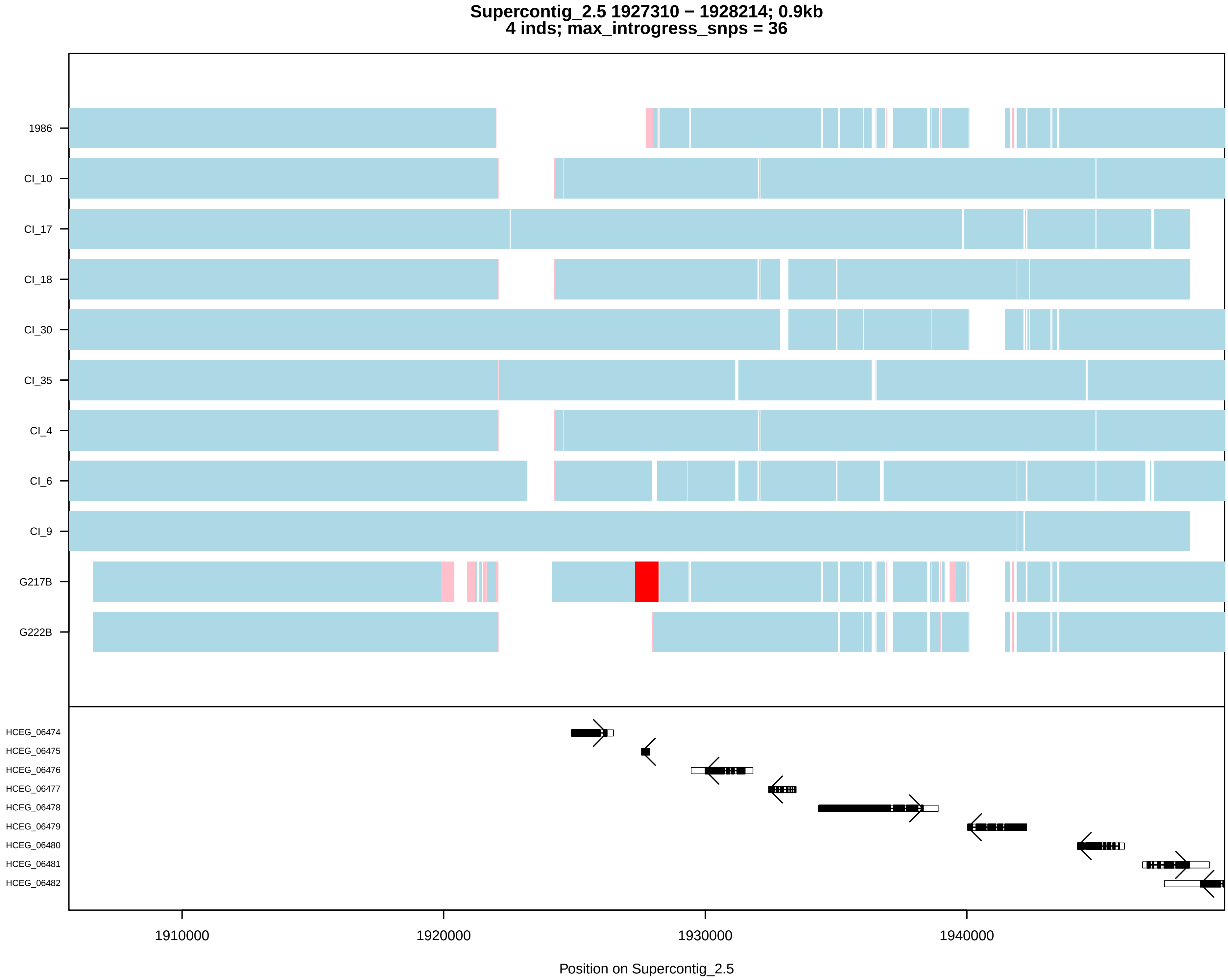
Supercontig_2.5 1927310 − 1928214; 0.9kb
4 inds; max_introgress_snps = 36
1986
CI_10
CI_17
CI_18
CI_30
CI_35
CI_4
CI_6
CI_9
G217B
G222B
HCEG_06474
HCEG_06475
HCEG_06476
HCEG_06477
HCEG_06478
HCEG_06479
HCEG_06480
HCEG_06481
HCEG_06482
1910000
1920000
1930000
1940000
Position on Supercontig_2.5

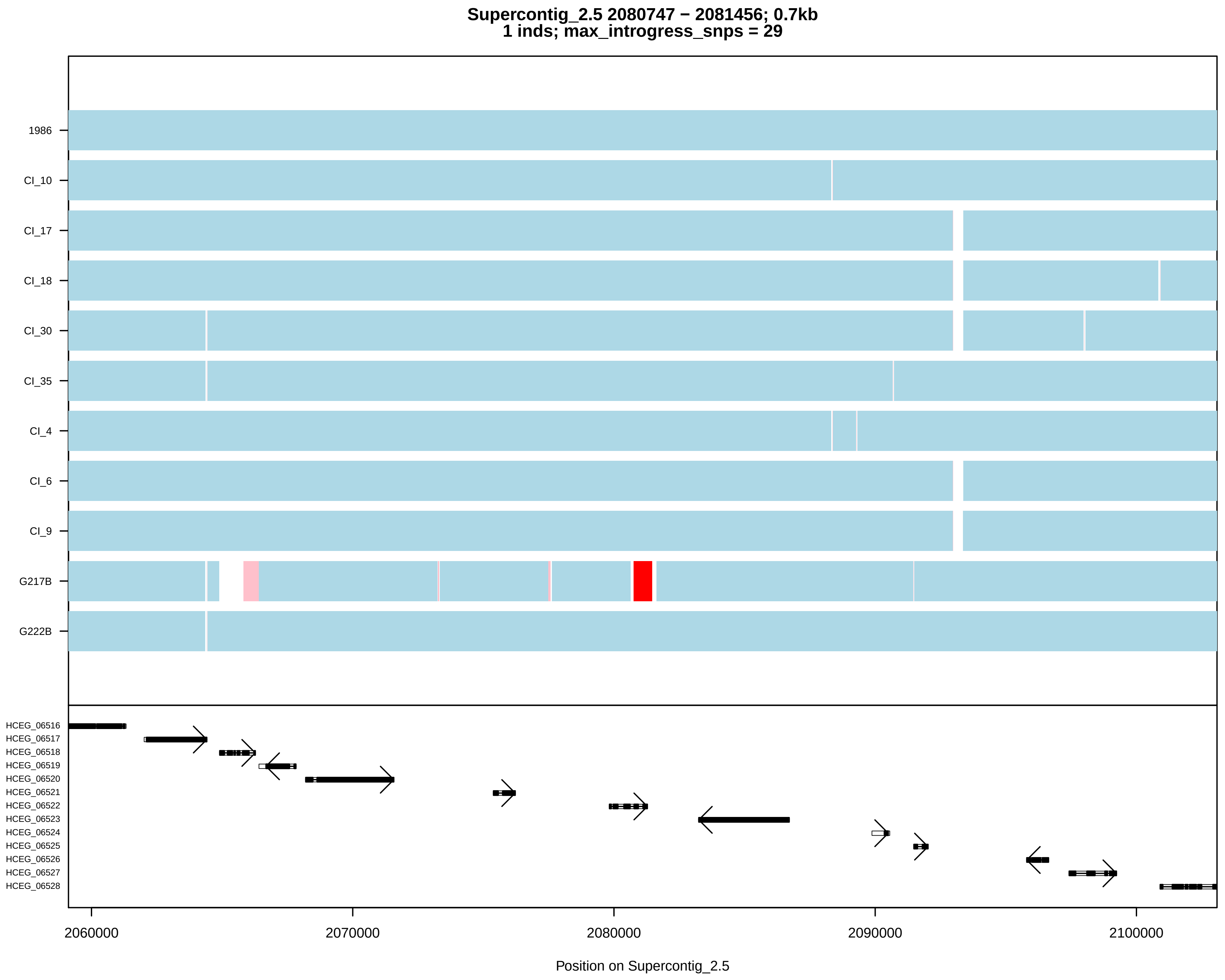

Supercontig_2.5 2080747 − 2081456; 0.7kb
1 inds; max_introgress_snps = 29
1986
CI_10
CI_17
CI_18
CI_30
CI_35
CI_4
CI_6
CI_9
G217B
G222B
HCEG_06516
HCEG_06517
HCEG_06518
HCEG_06519
HCEG_06520
HCEG_06521
HCEG_06522
HCEG_06523
HCEG_06524
HCEG_06525
HCEG_06526
HCEG_06527
HCEG_06528
2060000
2070000
2080000
2090000
2100000
Position on Supercontig_2.5

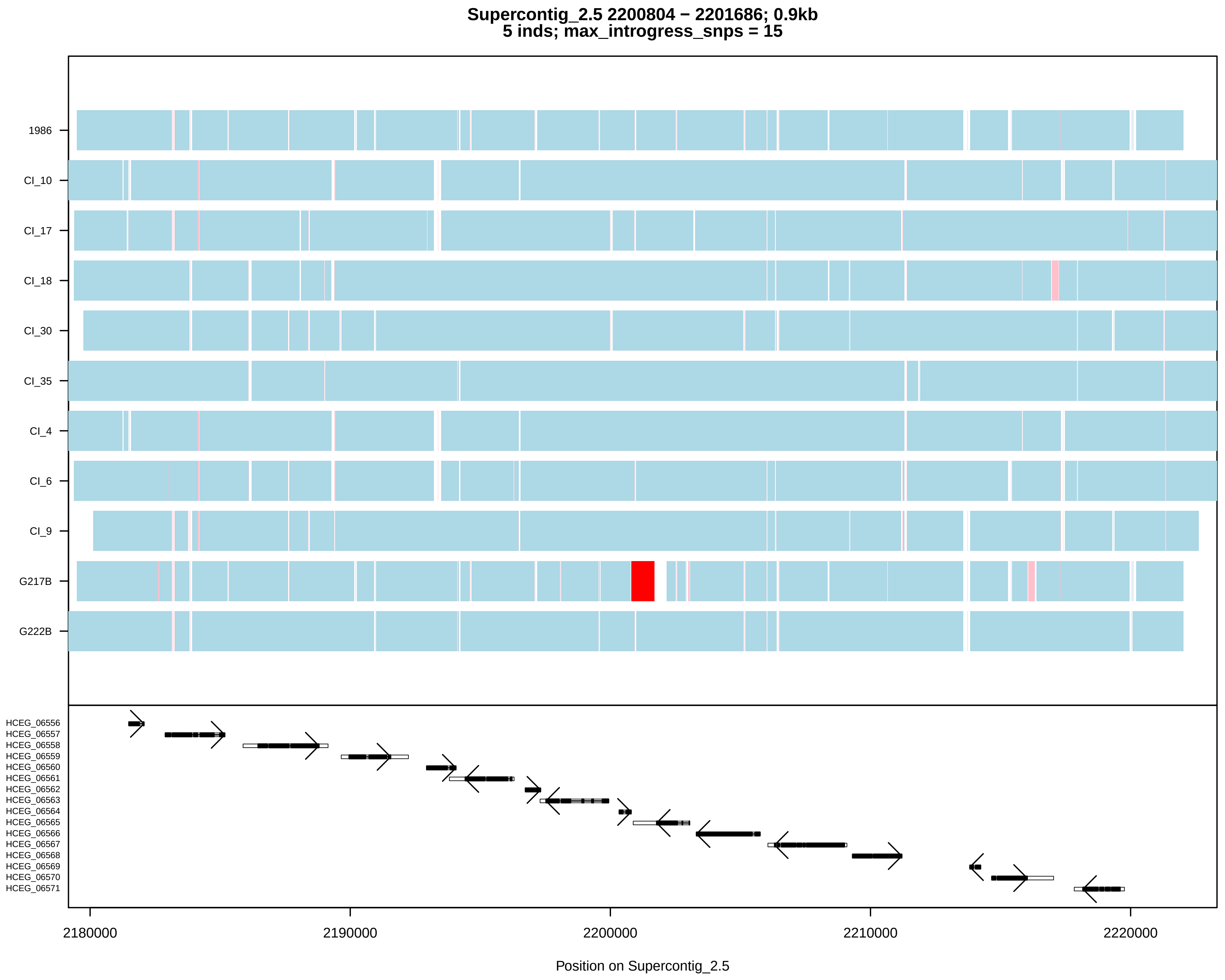
Supercontig_2.5 2200804 − 2201686; 0.9kb
5 inds; max_introgress_snps = 15
1986
CI_10
CI_17
CI_18
CI_30
CI_35
CI_4
CI_6
CI_9
G217B
G222B
HCEG_06556
HCEG_06557
HCEG_06558
HCEG_06559
HCEG_06560
HCEG_06561
HCEG_06562
HCEG_06563
HCEG_06564
HCEG_06565
HCEG_06566
HCEG_06567
HCEG_06568
HCEG_06569
HCEG_06570
HCEG_06571
2180000
2190000
2200000
2210000
2220000
Position on Supercontig_2.5

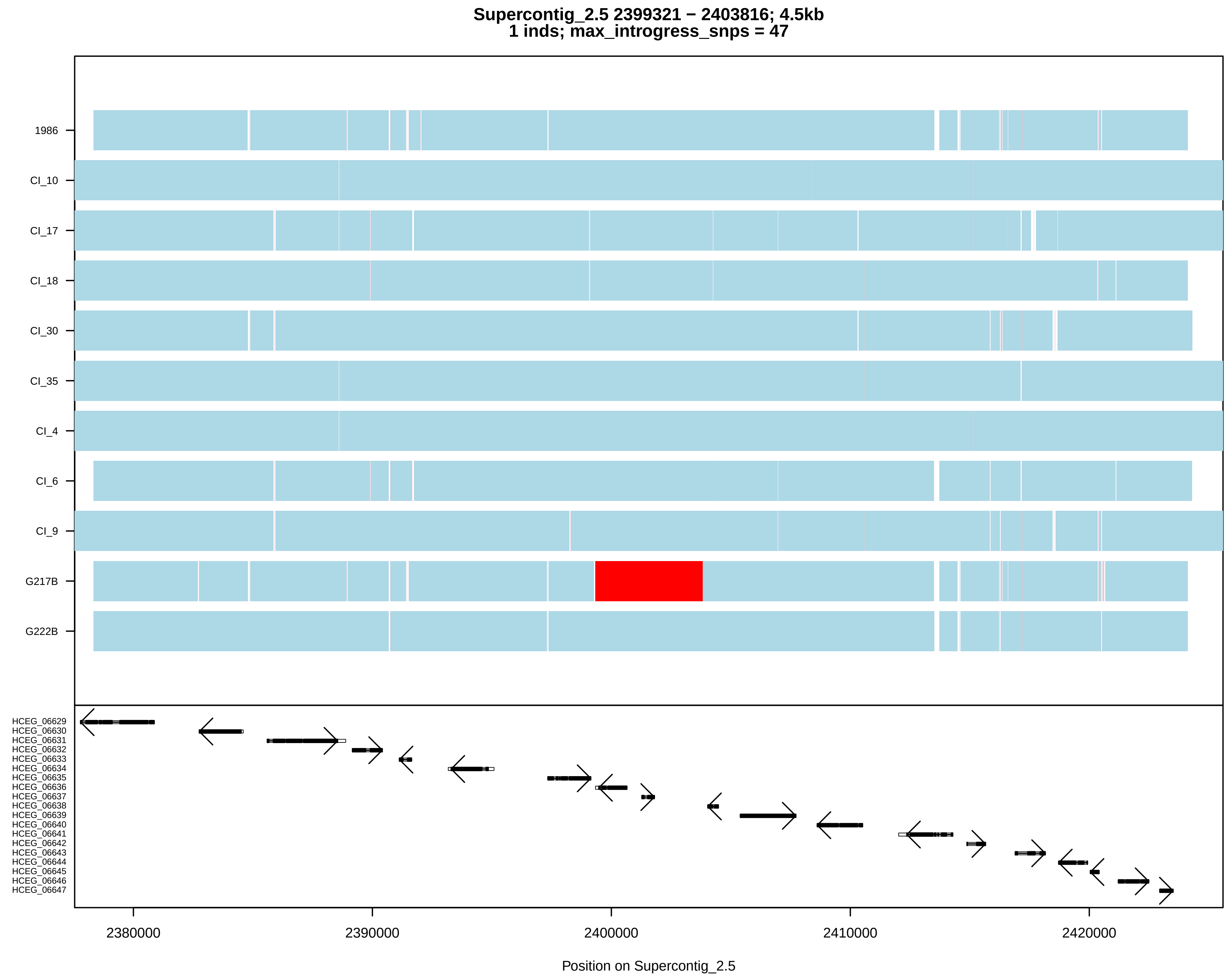

Supercontig_2.5 2399321 − 2403816; 4.5kb
1 inds; max_introgress_snps = 47
1986
CI_10
CI_17
CI_18
CI_30
CI_35
CI_4
CI_6
CI_9
G217B
G222B
HCEG_06629
HCEG_06630
HCEG_06631
HCEG_06632
HCEG_06633
HCEG_06634
HCEG_06635
HCEG_06636
HCEG_06637
HCEG_06638
HCEG_06639
HCEG_06640
HCEG_06641
HCEG_06642
HCEG_06643
HCEG_06644
HCEG_06645
HCEG_06646
HCEG_06647
2380000
2390000
2400000
2410000
2420000
Position on Supercontig_2.5

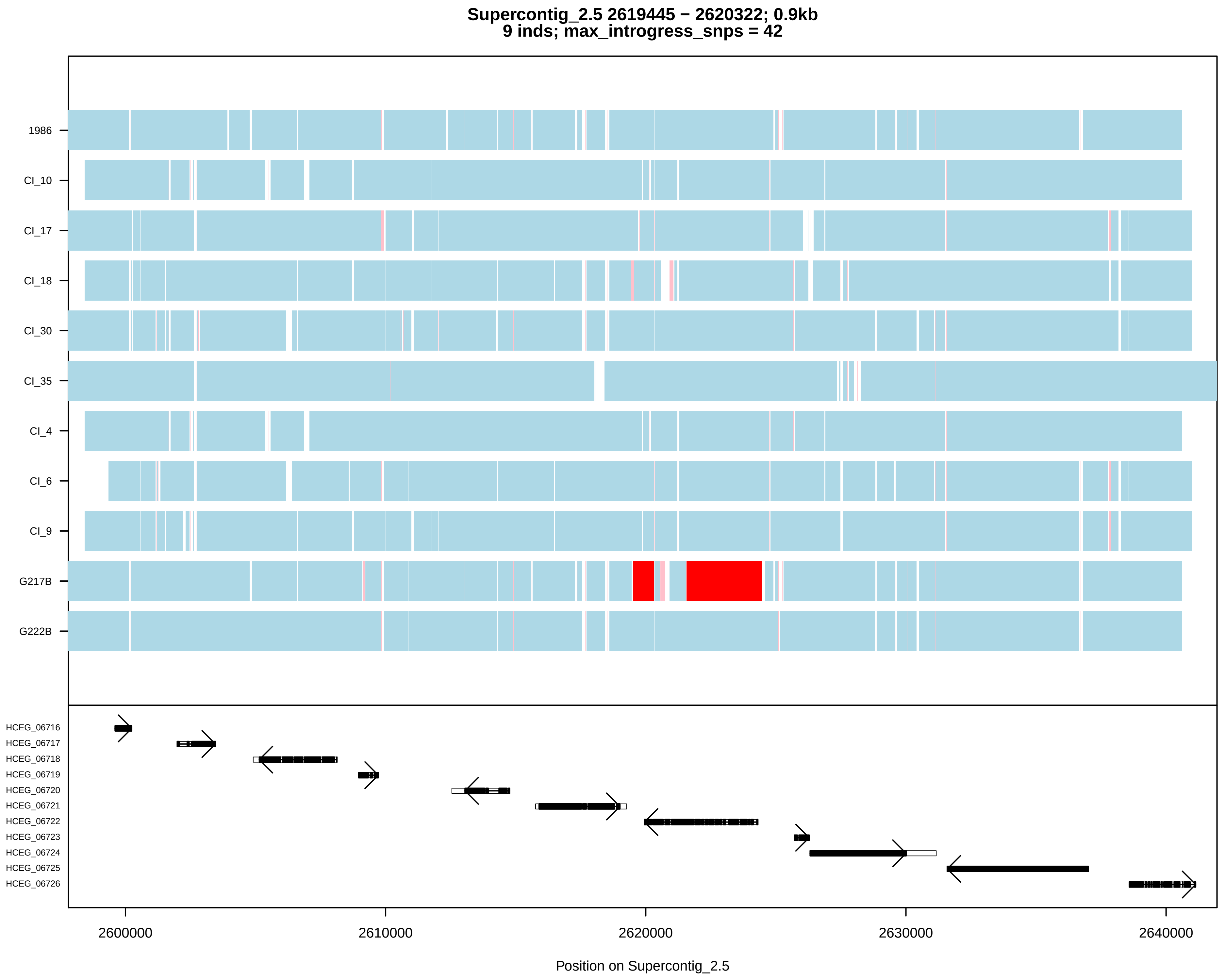
Supercontig_2.5 2619445 − 2620322; 0.9kb
9 inds; max_introgress_snps = 42
1986
CI_10
CI_17
CI_18
CI_30
CI_35
CI_4
CI_6
CI_9
G217B
G222B
HCEG_06716
HCEG_06717
HCEG_06718
HCEG_06719
HCEG_06720
HCEG_06721
HCEG_06722
HCEG_06723
HCEG_06724
HCEG_06725
HCEG_06726
2600000
2610000
2620000
2630000
2640000
Position on Supercontig_2.5

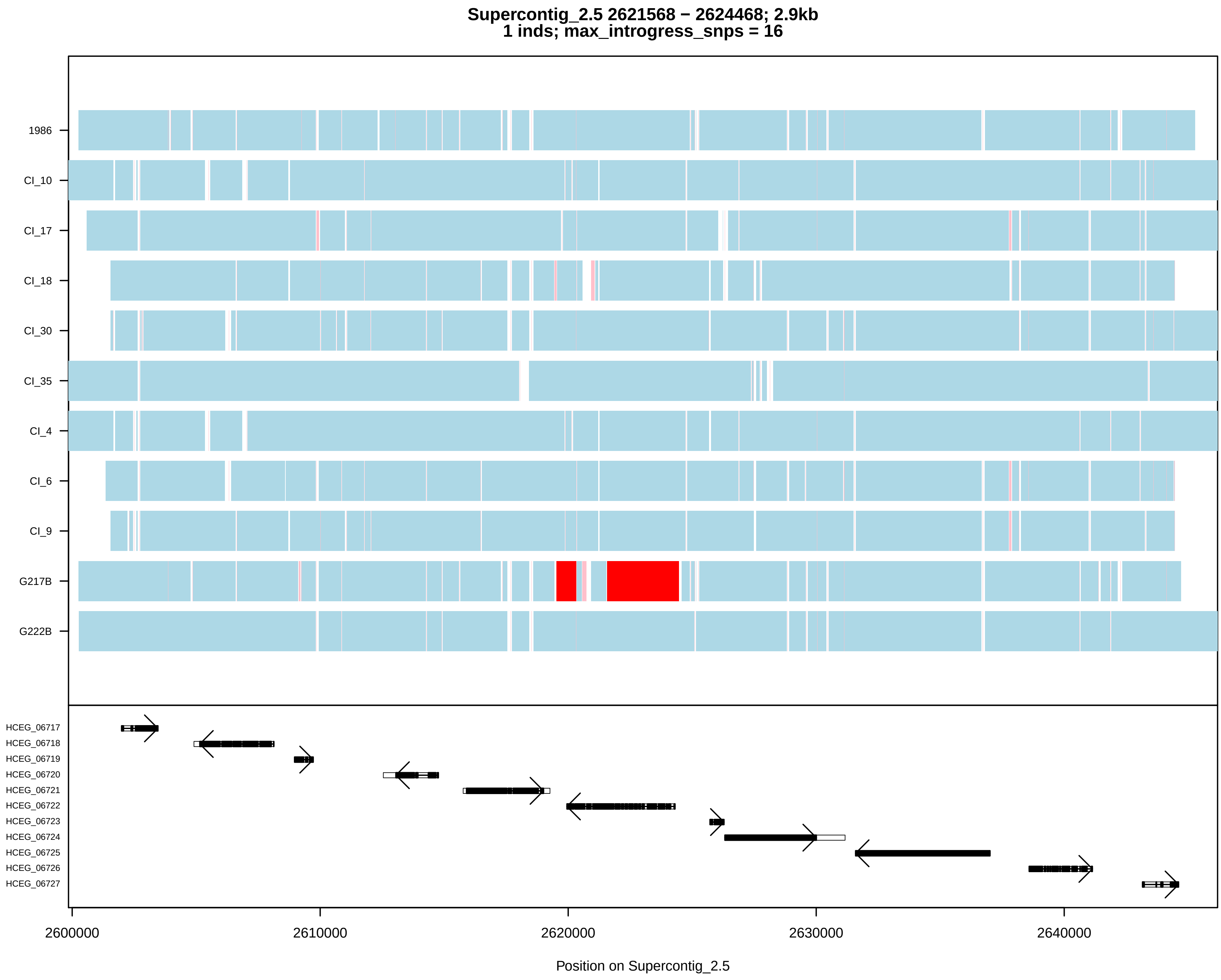

Supercontig_2.5 2621568 − 2624468; 2.9kb
1 inds; max_introgress_snps = 16
1986
CI_10
CI_17
CI_18
CI_30
CI_35
CI_4
CI_6
CI_9
G217B
G222B
HCEG_06717
HCEG_06718
HCEG_06719
HCEG_06720
HCEG_06721
HCEG_06722
HCEG_06723
HCEG_06724
HCEG_06725
HCEG_06726
HCEG_06727
2600000
2610000
2620000
2630000
2640000
Position on Supercontig_2.5

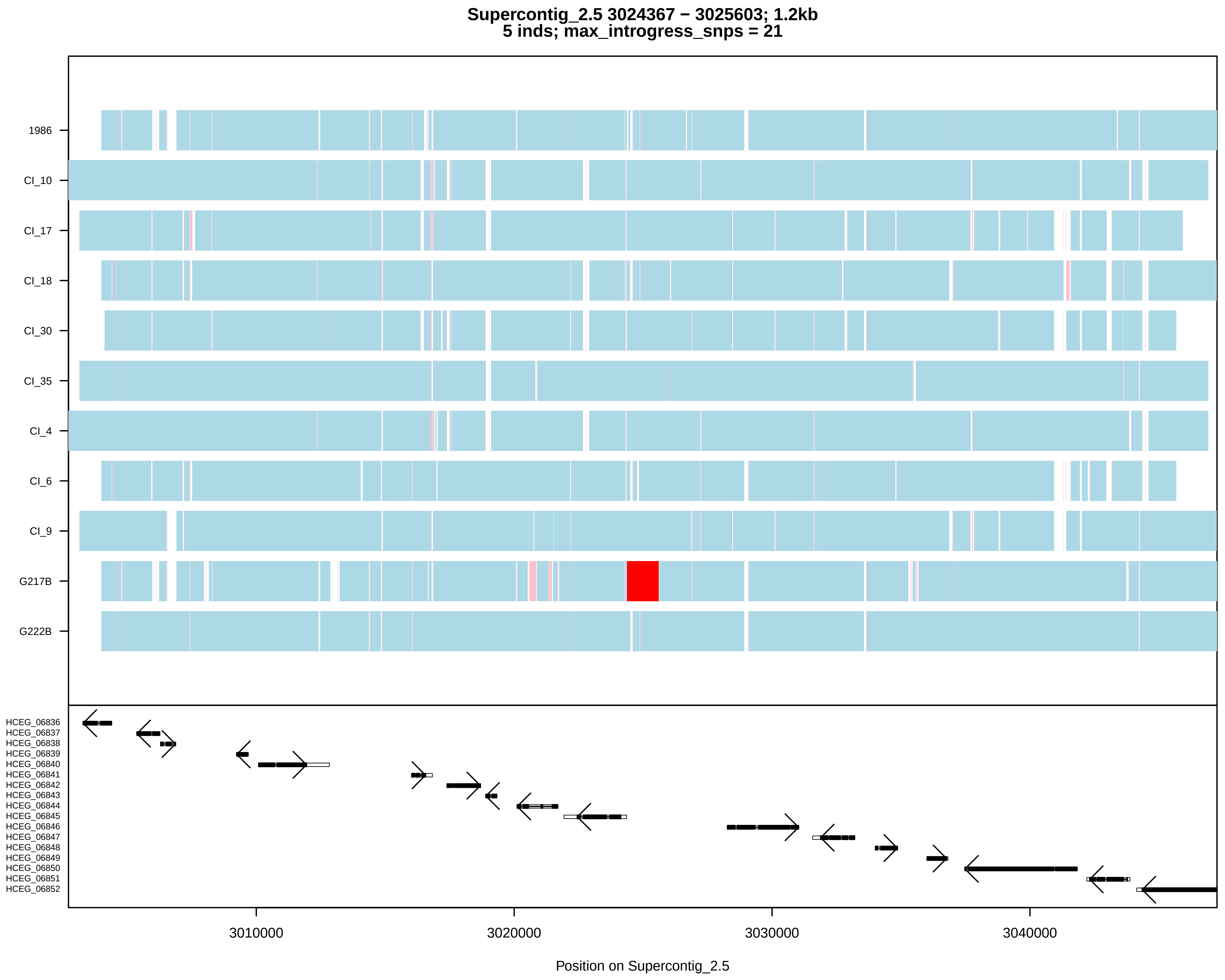

Supercontig_2.5 3024367 − 3025603; 1.2kb
5 inds; max_introgress_snps = 21
1986
CI_10
CI_17
CI_18
CI_30
CI_35
CI_4
CI_6
CI_9
G217B
G222B
HCEG_06836
HCEG_06837
HCEG_06838
HCEG_06839
HCEG_06840
HCEG_06841
HCEG_06842
HCEG_06843
HCEG_06844
HCEG_06845
HCEG_06846
HCEG_06847
HCEG_06848
HCEG_06849
HCEG_06850
HCEG_06851
HCEG_06852
3010000
3020000
3030000
3040000
Position on Supercontig_2.5

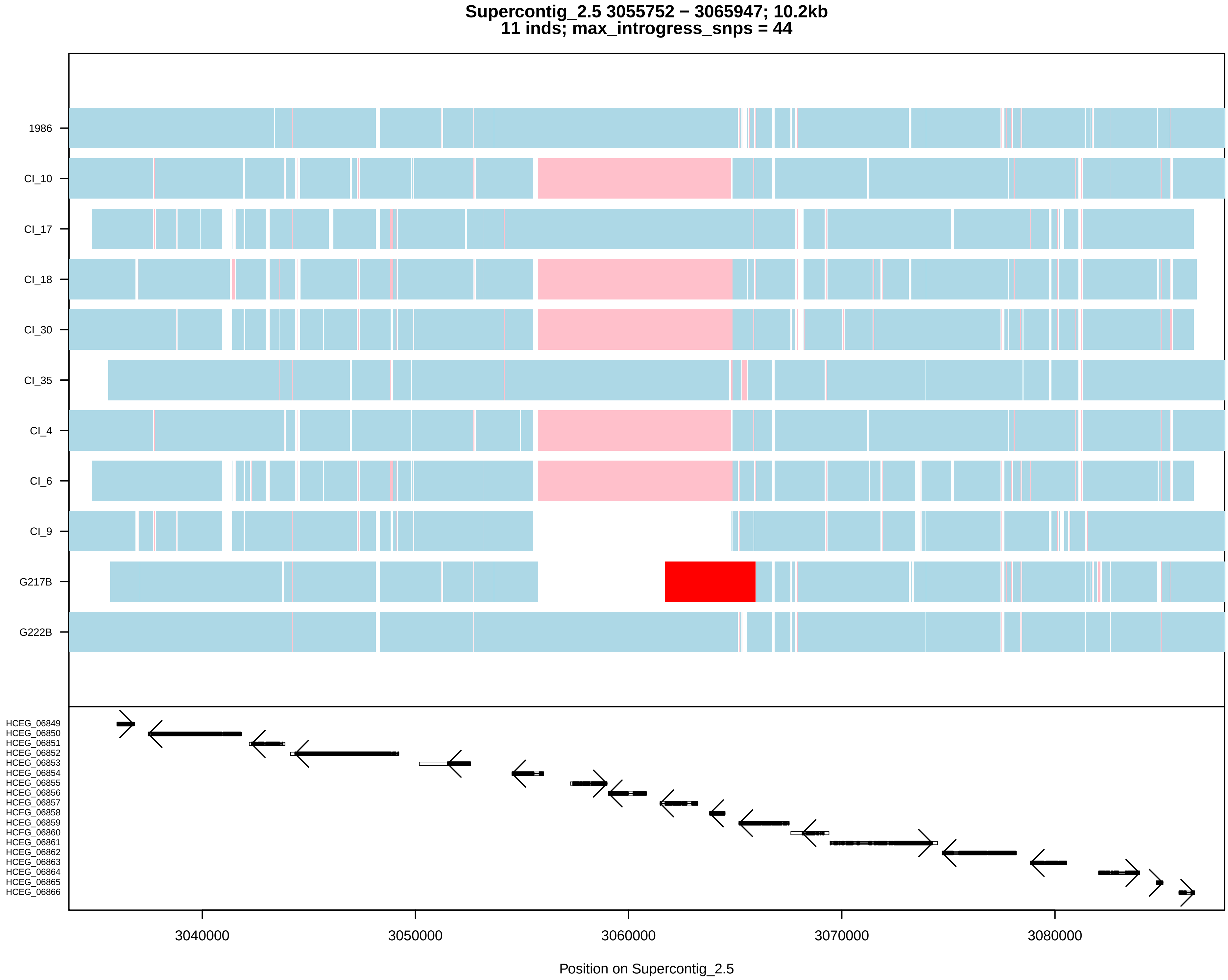
Supercontig_2.5 3055752 − 3065947; 10.2kb
11 inds; max_introgress_snps = 44
1986
CI_10
CI_17
CI_18
CI_30
CI_35
CI_4
CI_6
CI_9
G217B
G222B
HCEG_06849
HCEG_06850
HCEG_06851
HCEG_06852
HCEG_06853
HCEG_06854
HCEG_06855
HCEG_06856
HCEG_06857
HCEG_06858
HCEG_06859
HCEG_06860
HCEG_06861
HCEG_06862
HCEG_06863
HCEG_06864
HCEG_06865
HCEG_06866
3040000
3050000
3060000
3070000
3080000
Position on Supercontig_2.5

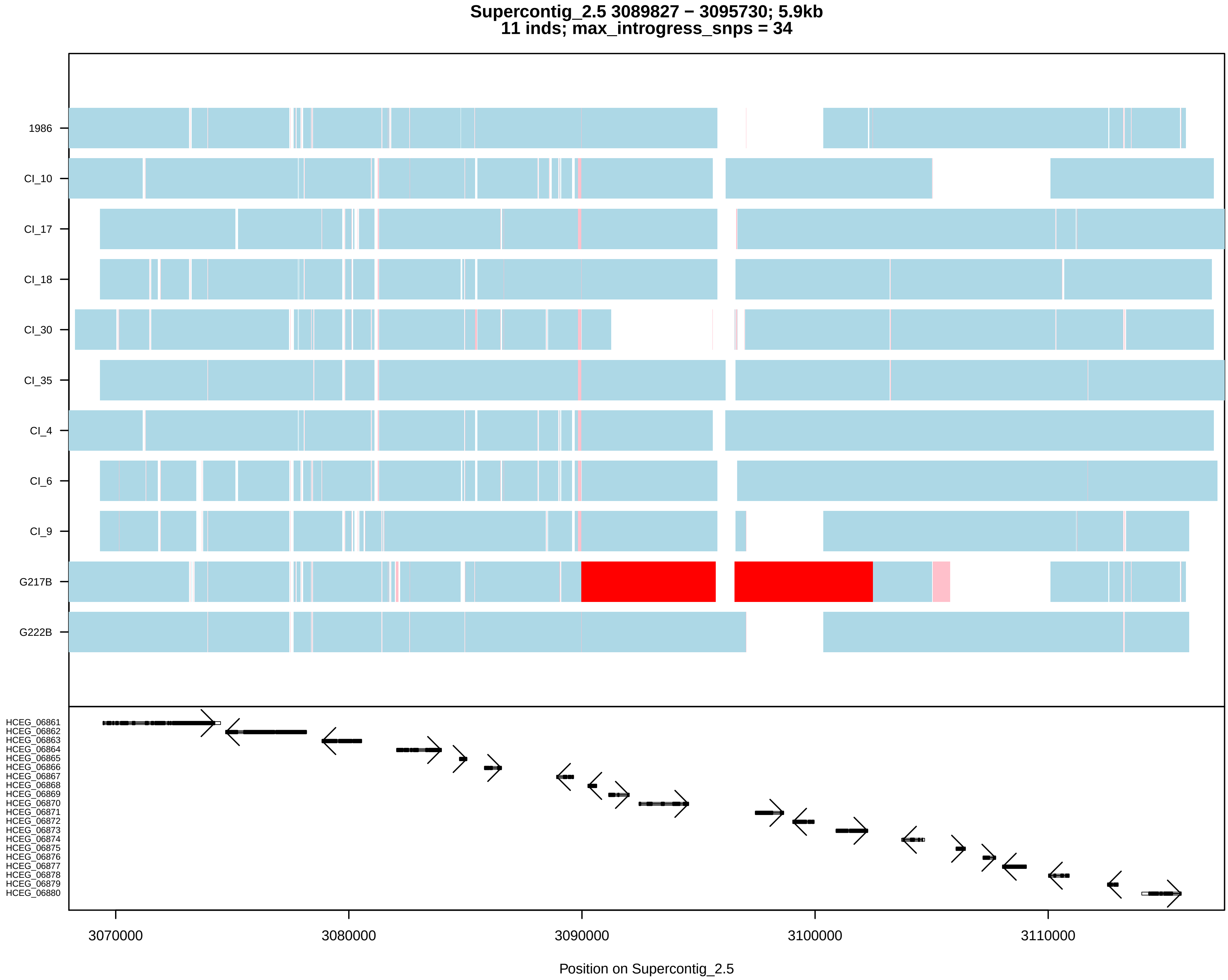

Supercontig_2.5 3089827 − 3095730; 5.9kb
11 inds; max_introgress_snps = 34
1986
CI_10
CI_17
CI_18
CI_30
CI_35
CI_4
CI_6
CI_9
G217B
G222B
HCEG_06861
HCEG_06862
HCEG_06863
HCEG_06864
HCEG_06865
HCEG_06866
HCEG_06867
HCEG_06868
HCEG_06869
HCEG_06870
HCEG_06871
HCEG_06872
HCEG_06873
HCEG_06874
HCEG_06875
HCEG_06876
HCEG_06877
HCEG_06878
HCEG_06879
HCEG_06880
3070000
3080000
3090000
3100000
3110000
Position on Supercontig_2.5

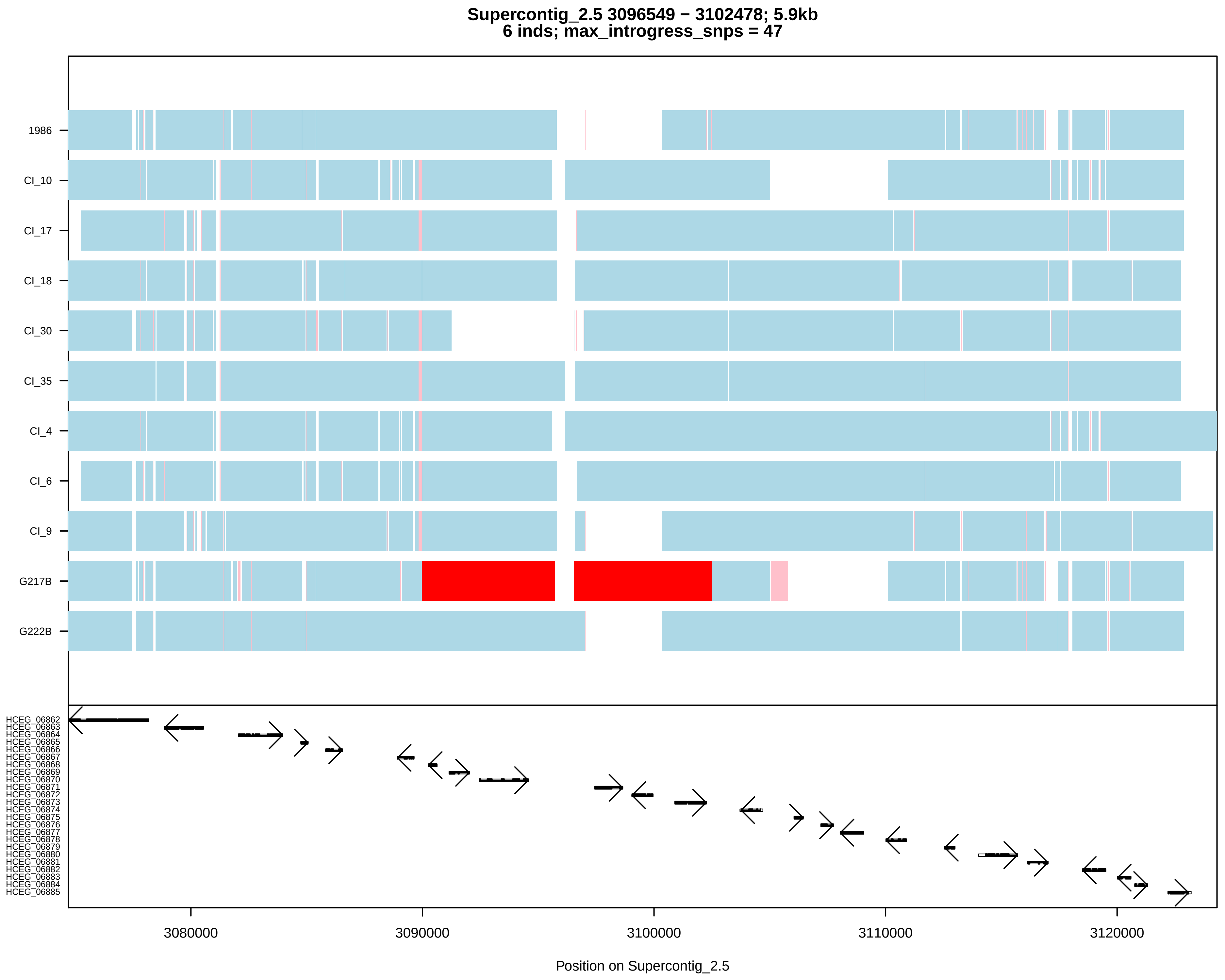
Supercontig_2.5 3096549 − 3102478; 5.9kb
6 inds; max_introgress_snps = 47
1986
CI_10
CI_17
CI_18
CI_30
CI_35
CI_4
CI_6
CI_9
G217B
G222B
HCEG_06862
HCEG_06863
HCEG_06864
HCEG_06865
HCEG_06866
HCEG_06867
HCEG_06868
HCEG_06869
HCEG_06870
HCEG_06871
HCEG_06872
HCEG_06873
HCEG_06874
HCEG_06875
HCEG_06876
HCEG_06877
HCEG_06878
HCEG_06879
HCEG_06880
HCEG_06881
HCEG_06882
HCEG_06883
HCEG_06884
HCEG_06885
3080000
3090000
3100000
3110000
3120000
Position on Supercontig_2.5

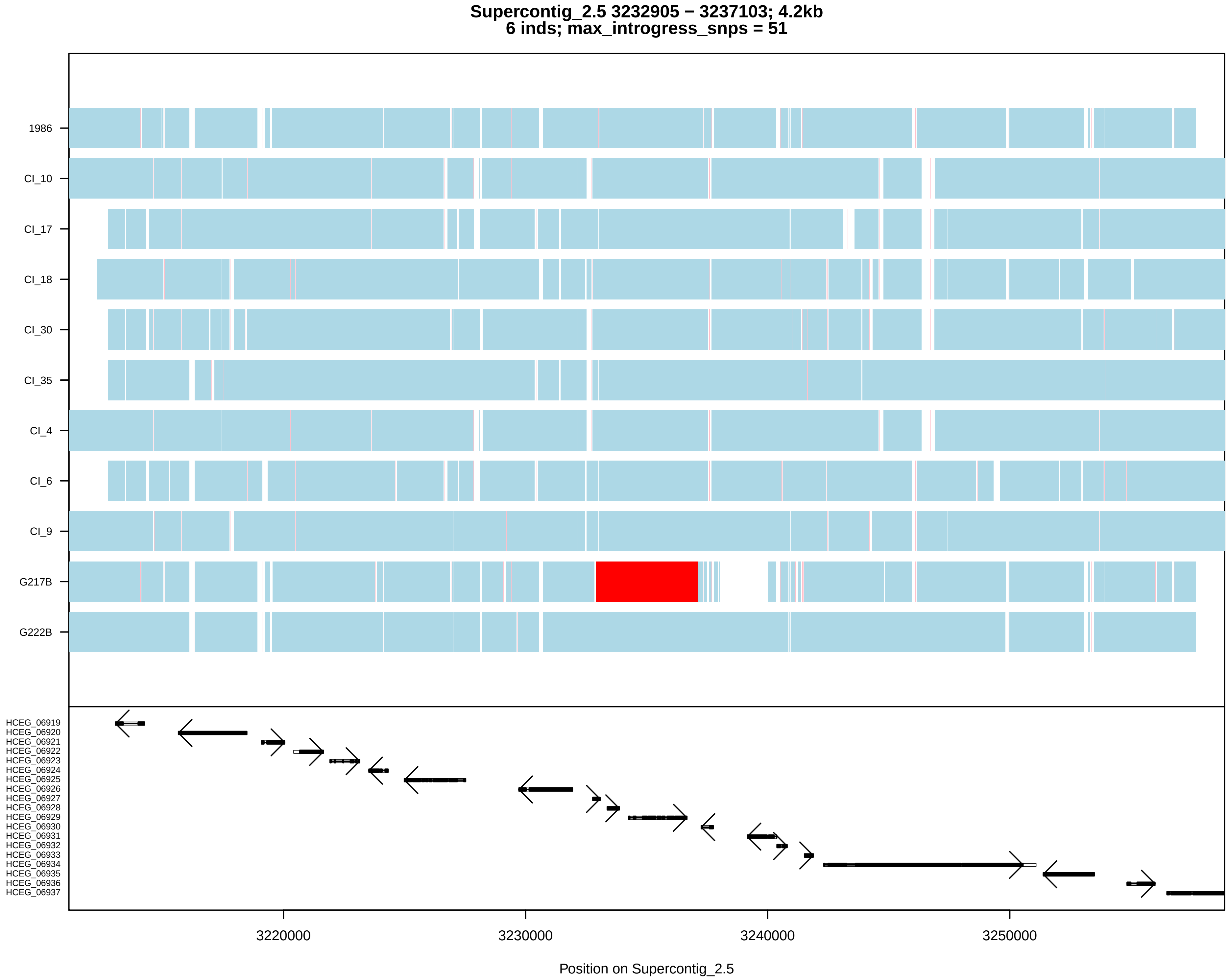

Supercontig_2.5 3232905 − 3237103; 4.2kb
6 inds; max_introgress_snps = 51
1986
CI_10
CI_17
CI_18
CI_30
CI_35
CI_4
CI_6
CI_9
G217B
G222B
HCEG_06919
HCEG_06920
HCEG_06921
HCEG_06922
HCEG_06923
HCEG_06924
HCEG_06925
HCEG_06926
HCEG_06927
HCEG_06928
HCEG_06929
HCEG_06930
HCEG_06931
HCEG_06932
HCEG_06933
HCEG_06934
HCEG_06935
HCEG_06936
HCEG_06937
3220000
3230000
3240000
3250000
Position on Supercontig_2.5

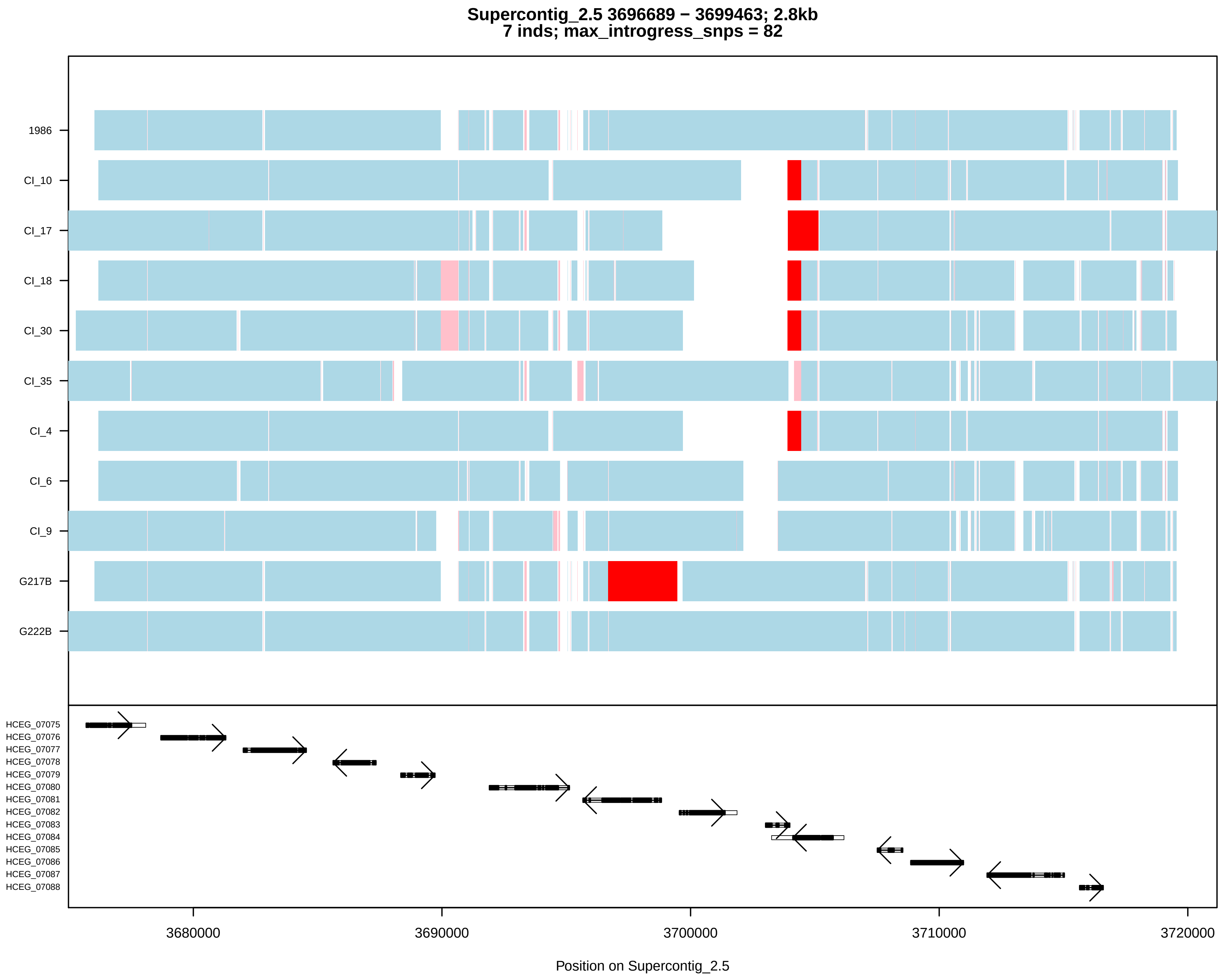

Supercontig_2.5 3696689 − 3699463; 2.8kb
7 inds; max_introgress_snps = 82
1986
CI_10
CI_17
CI_18
CI_30
CI_35
CI_4
CI_6
CI_9
G217B
G222B
HCEG_07075
HCEG_07076
HCEG_07077
HCEG_07078
HCEG_07079
HCEG_07080
HCEG_07081
HCEG_07082
HCEG_07083
HCEG_07084
HCEG_07085
HCEG_07086
HCEG_07087
HCEG_07088
3680000
3690000
3700000
3710000
3720000
Position on Supercontig_2.5

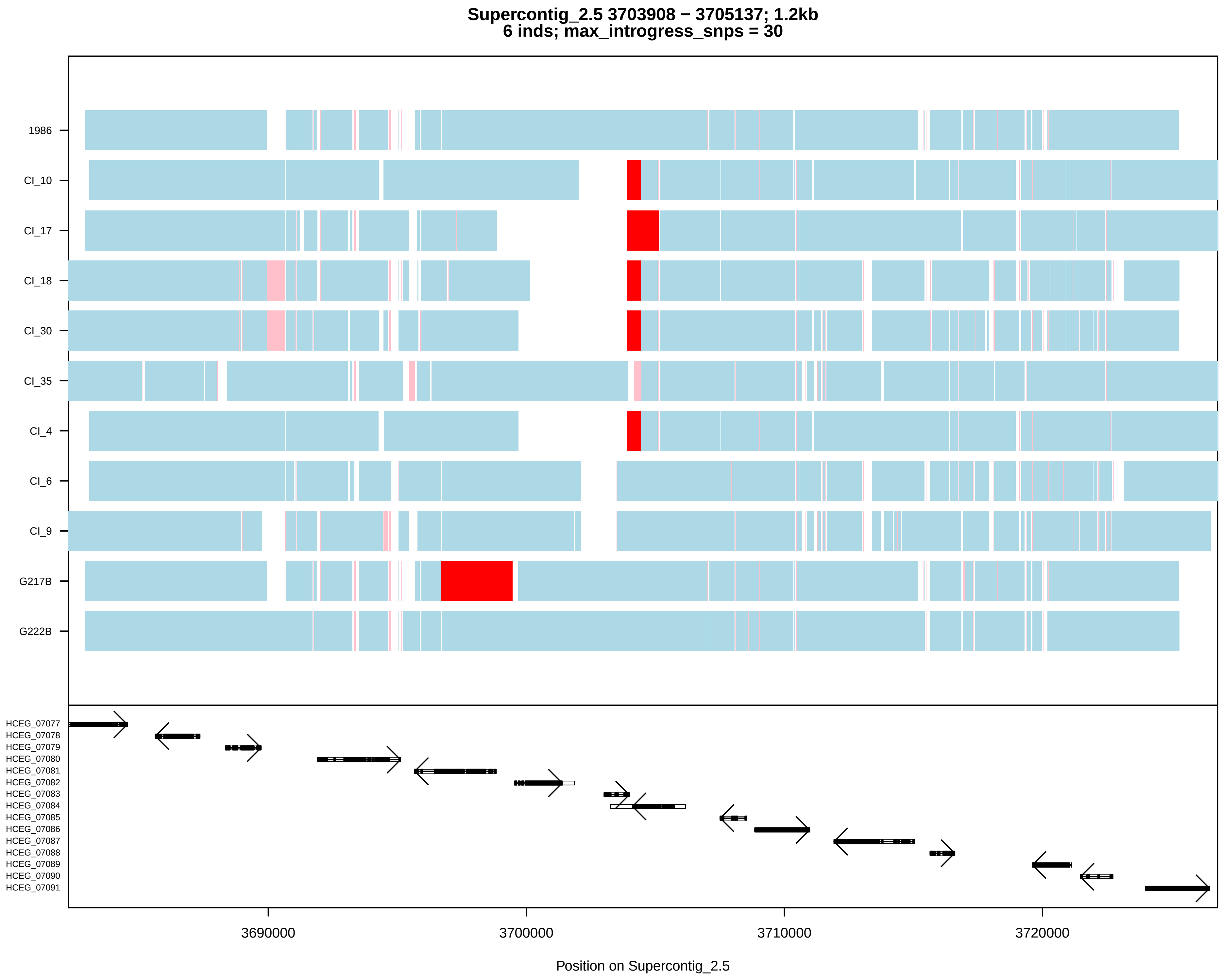
Supercontig_2.5 3703908 − 3705137; 1.2kb
6 inds; max_introgress_snps = 30
1986
CI_10
CI_17
CI_18
CI_30
CI_35
CI_4
CI_6
CI_9
G217B
G222B
HCEG_07077
HCEG_07078
HCEG_07079
HCEG_07080
HCEG_07081
HCEG_07082
HCEG_07083
HCEG_07084
HCEG_07085
HCEG_07086
HCEG_07087
HCEG_07088
HCEG_07089
HCEG_07090
HCEG_07091
3690000
3700000
3710000
3720000
Position on Supercontig_2.5

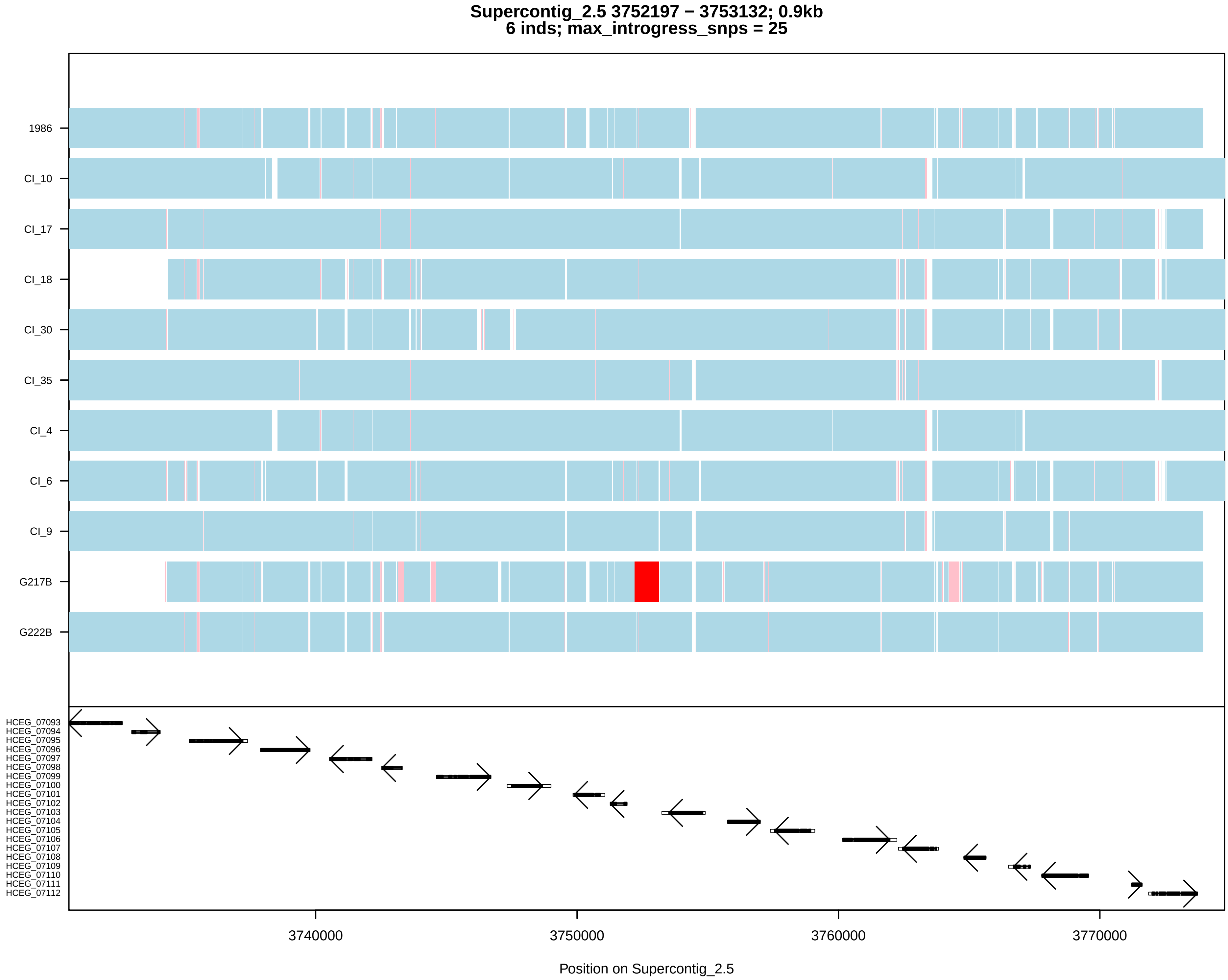

Supercontig_2.5 3752197 − 3753132; 0.9kb
6 inds; max_introgress_snps = 25
1986
CI_10
CI_17
CI_18
CI_30
CI_35
CI_4
CI_6
CI_9
G217B
G222B
HCEG_07093
HCEG_07094
HCEG_07095
HCEG_07096
HCEG_07097
HCEG_07098
HCEG_07099
HCEG_07100
HCEG_07101
HCEG_07102
HCEG_07103
HCEG_07104
HCEG_07105
HCEG_07106
HCEG_07107
HCEG_07108
HCEG_07109
HCEG_07110
HCEG_07111
HCEG_07112
3740000
3750000
3760000
3770000
Position on Supercontig_2.5

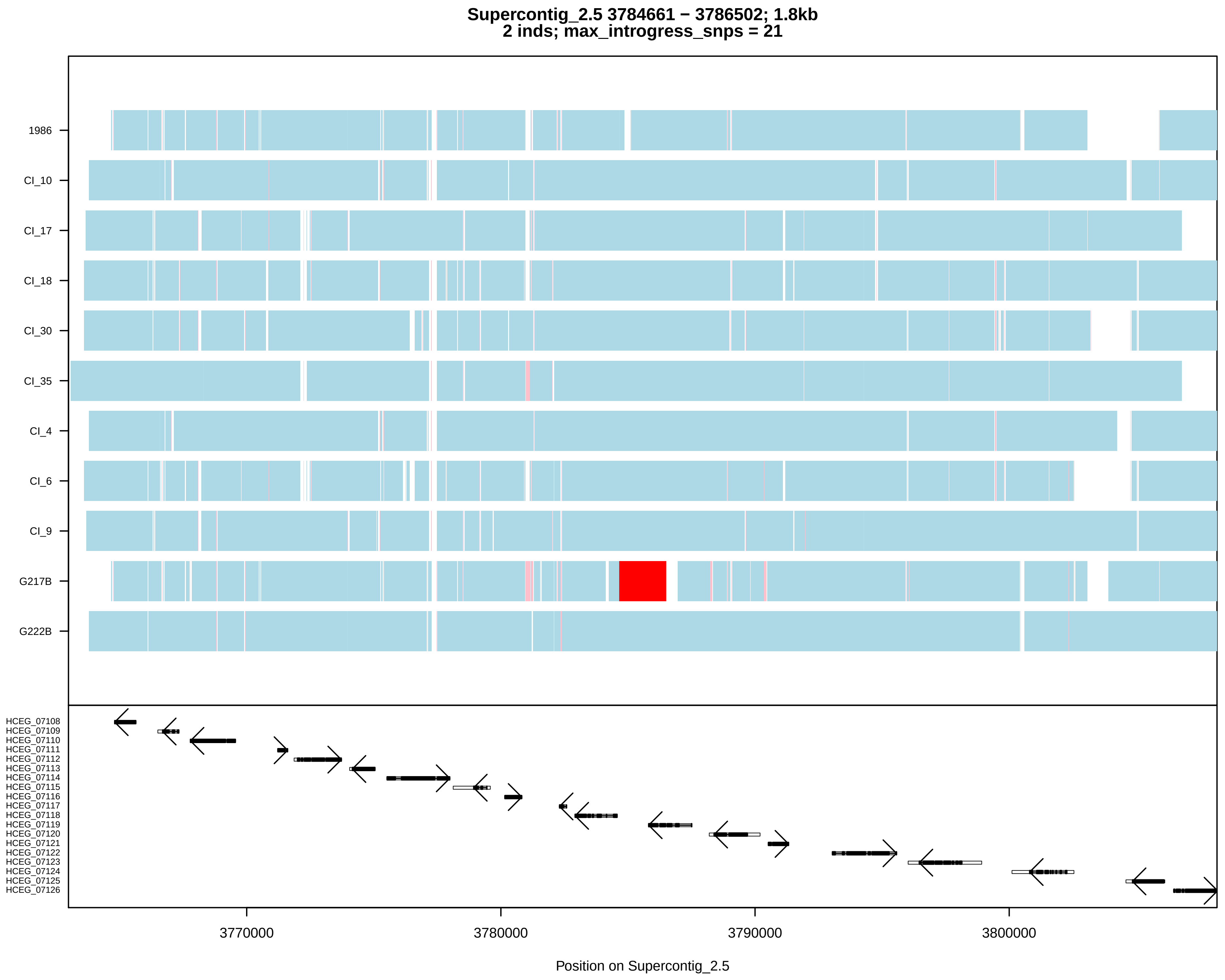

Supercontig_2.5 3784661 − 3786502; 1.8kb
2 inds; max_introgress_snps = 21
1986
CI_10
CI_17
CI_18
CI_30
CI_35
CI_4
CI_6
CI_9
G217B
G222B
HCEG_07108
HCEG_07109
HCEG_07110
HCEG_07111
HCEG_07112
HCEG_07113
HCEG_07114
HCEG_07115
HCEG_07116
HCEG_07117
HCEG_07118
HCEG_07119
HCEG_07120
HCEG_07121
HCEG_07122
HCEG_07123
HCEG_07124
HCEG_07125
HCEG_07126
3770000
3780000
3790000
3800000
Position on Supercontig_2.5

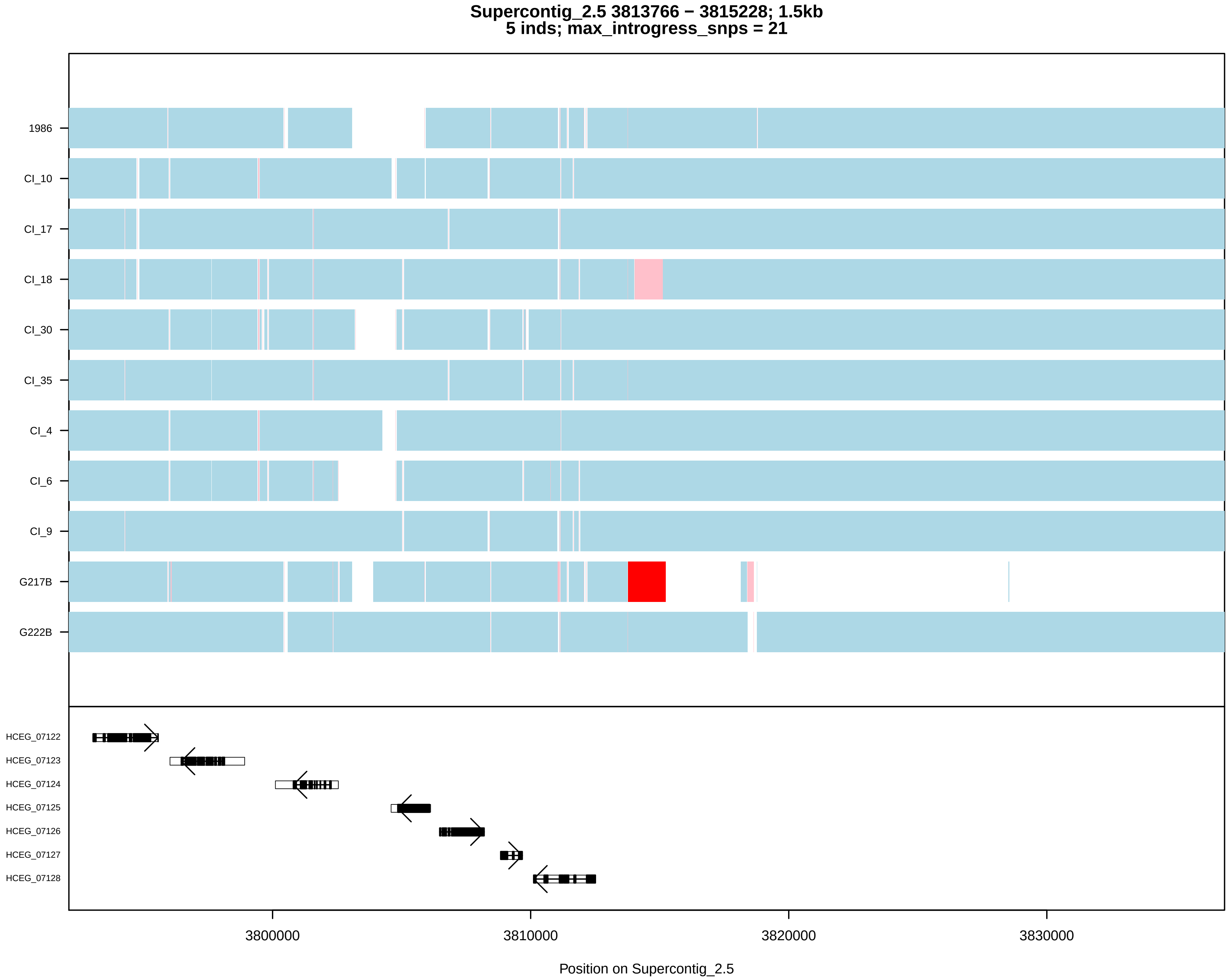
Supercontig_2.5 3813766 − 3815228; 1.5kb
5 inds; max_introgress_snps = 21
1986
CI_10
CI_17
CI_18
CI_30
CI_35
CI_4
CI_6
CI_9
G217B
G222B
HCEG_07122
HCEG_07123
HCEG_07124
HCEG_07125
HCEG_07126
HCEG_07127
HCEG_07128
3800000
3810000
3820000
3830000
Position on Supercontig_2.5

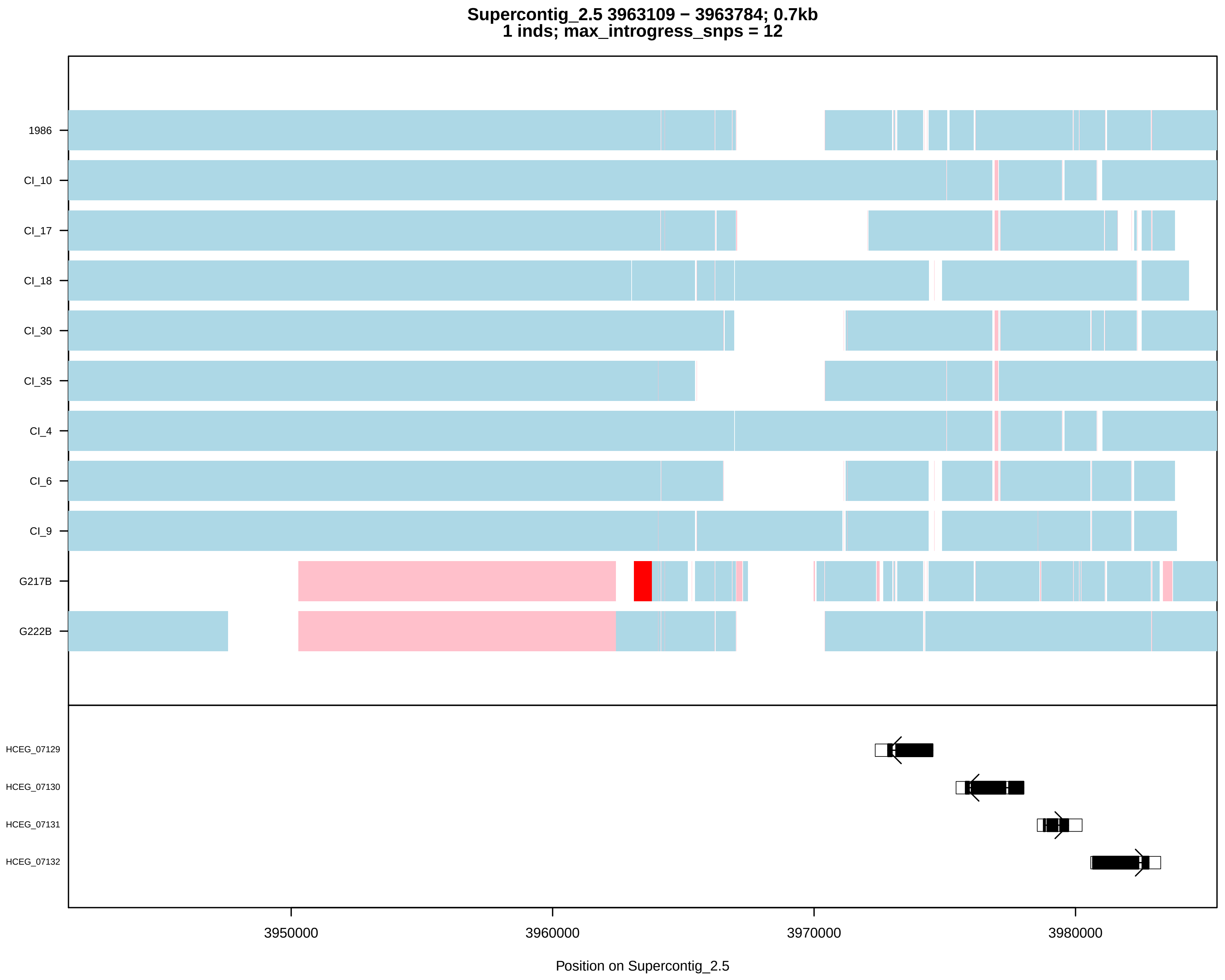

Supercontig_2.5 3963109 − 3963784; 0.7kb
1 inds; max_introgress_snps = 12
1986
CI_10
CI_17
CI_18
CI_30
CI_35
CI_4
CI_6
CI_9
G217B
G222B
HCEG_07129
HCEG_07130
HCEG_07131
HCEG_07132
3950000
3960000
3970000
3980000
Position on Supercontig_2.5

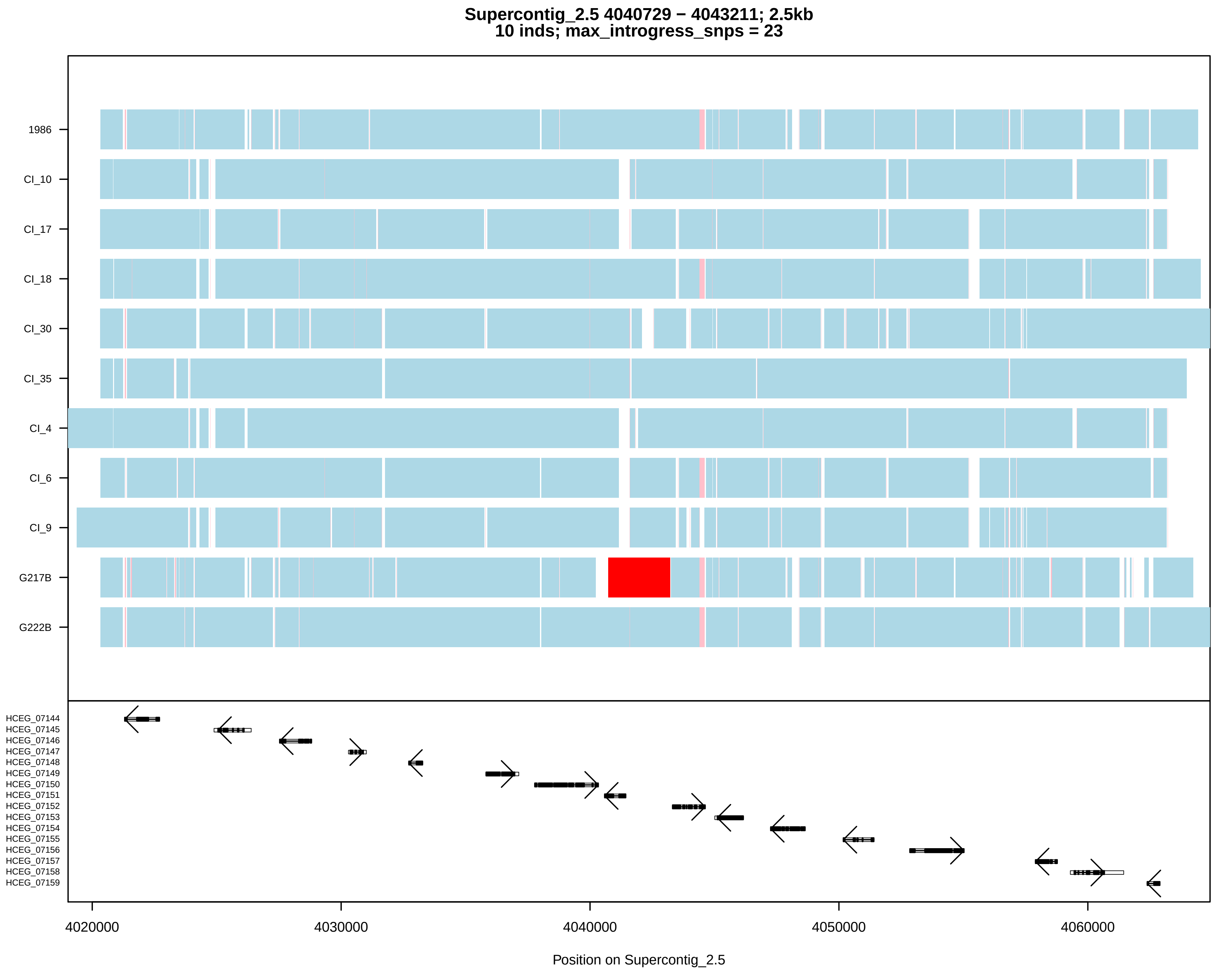
Supercontig_2.5 4040729 − 4043211; 2.5kb
10 inds; max_introgress_snps = 23
1986
CI_10
CI_17
CI_18
CI_30
CI_35
CI_4
CI_6
CI_9
G217B
G222B
HCEG_07144
HCEG_07145
HCEG_07146
HCEG_07147
HCEG_07148
HCEG_07149
HCEG_07150
HCEG_07151
HCEG_07152
HCEG_07153
HCEG_07154
HCEG_07155
HCEG_07156
HCEG_07157
HCEG_07158
HCEG_07159
4020000
4030000
4040000
4050000
4060000
Position on Supercontig_2.5

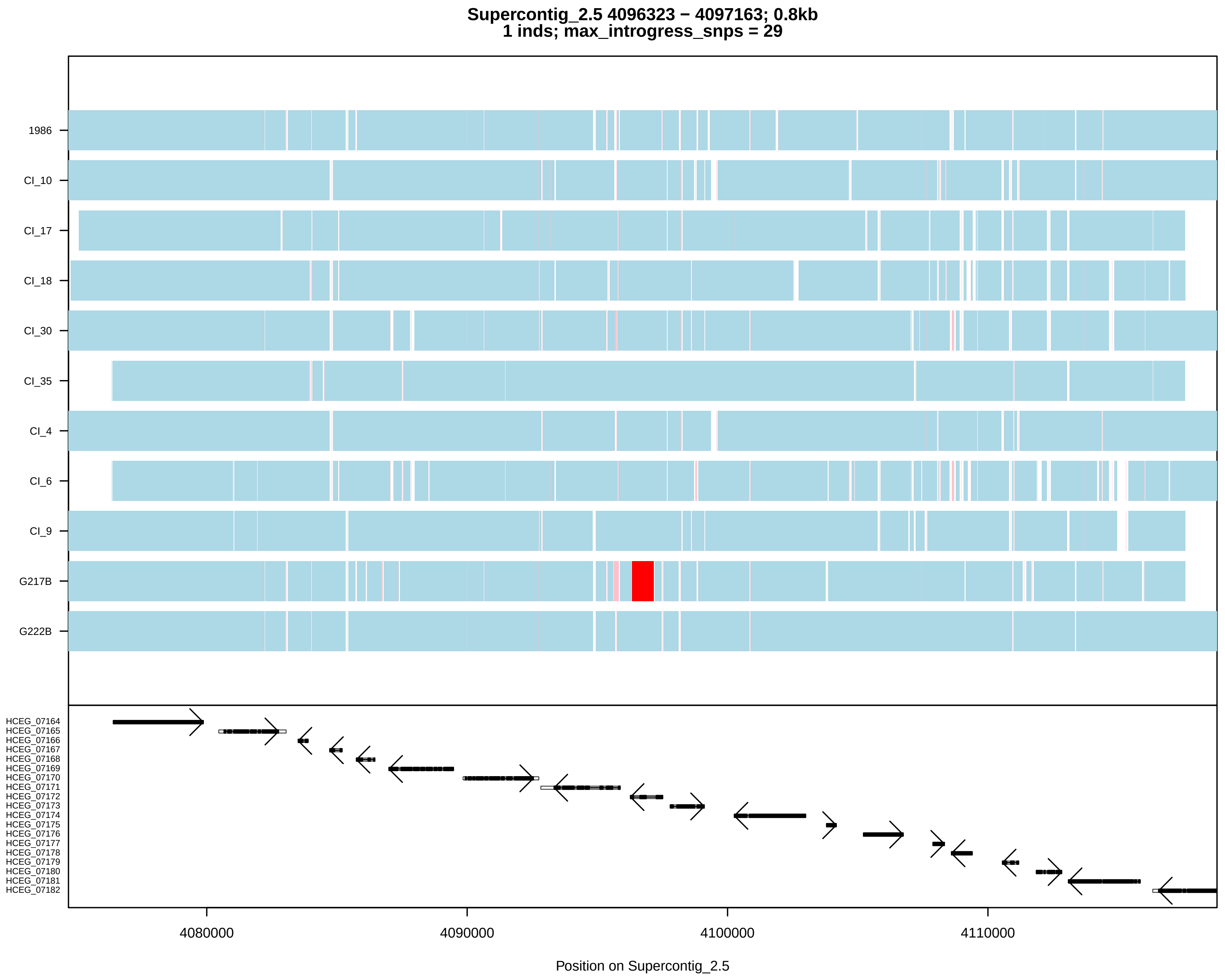
Supercontig_2.5 4096323 − 4097163; 0.8kb
1 inds; max_introgress_snps = 29
1986
CI_10
CI_17
CI_18
CI_30
CI_35
CI_4
CI_6
CI_9
G217B
G222B
HCEG_07164
HCEG_07165
HCEG_07166
HCEG_07167
HCEG_07168
HCEG_07169
HCEG_07170
HCEG_07171
HCEG_07172
HCEG_07173
HCEG_07174
HCEG_07175
HCEG_07176
HCEG_07177
HCEG_07178
HCEG_07179
HCEG_07180
HCEG_07181
HCEG_07182
4080000
4090000
4100000
4110000
Position on Supercontig_2.5

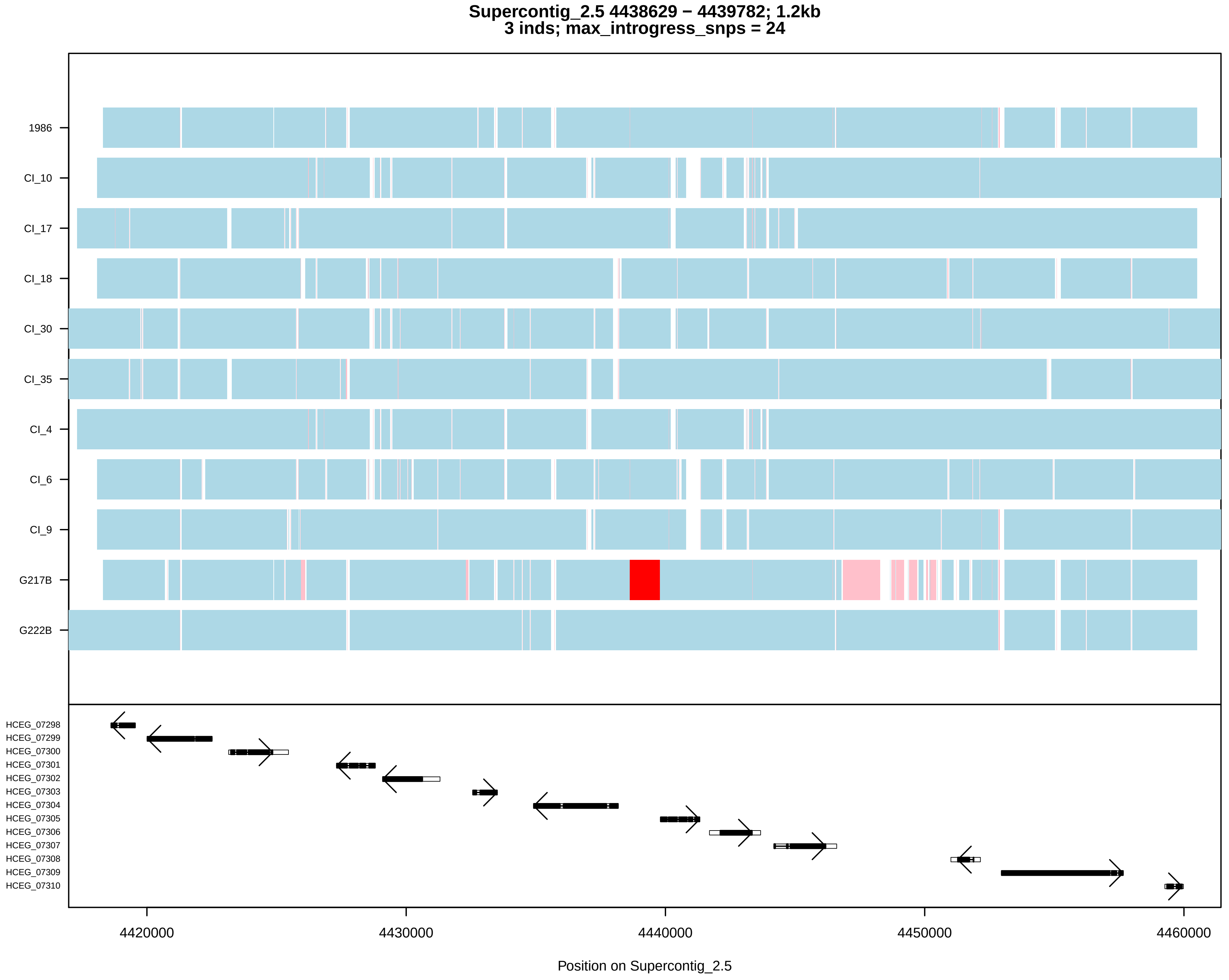

Supercontig_2.5 4438629 − 4439782; 1.2kb
3 inds; max_introgress_snps = 24
1986
CI_10
CI_17
CI_18
CI_30
CI_35
CI_4
CI_6
CI_9
G217B
G222B
HCEG_07298
HCEG_07299
HCEG_07300
HCEG_07301
HCEG_07302
HCEG_07303
HCEG_07304
HCEG_07305
HCEG_07306
HCEG_07307
HCEG_07308
HCEG_07309
HCEG_07310
4420000
4430000
4440000
4450000
4460000
Position on Supercontig_2.5

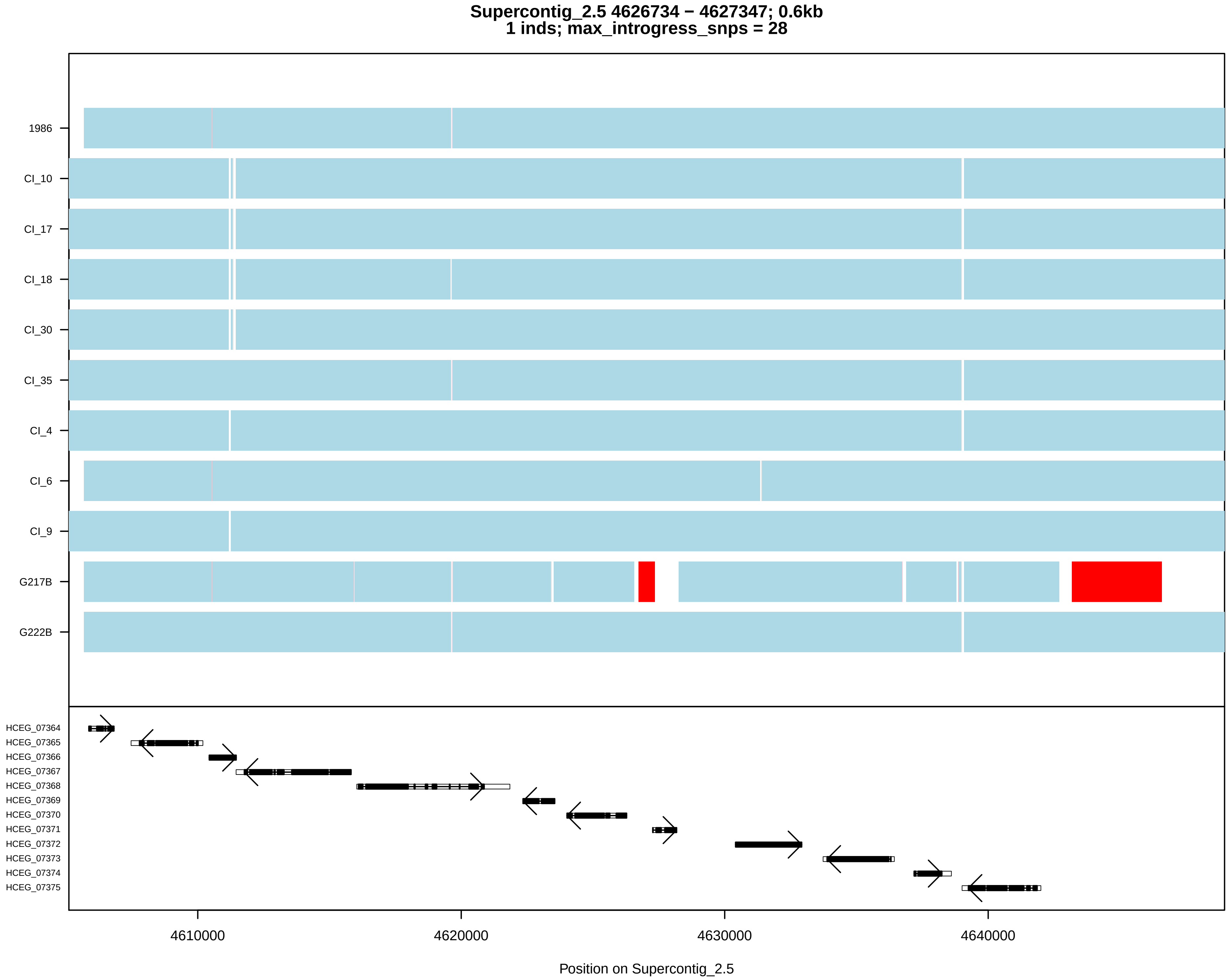

Supercontig_2.5 4626734 − 4627347; 0.6kb
1 inds; max_introgress_snps = 28
1986
CI_10
CI_17
CI_18
CI_30
CI_35
CI_4
CI_6
CI_9
G217B
G222B
HCEG_07364
HCEG_07365
HCEG_07366
HCEG_07367
HCEG_07368
HCEG_07369
HCEG_07370
HCEG_07371
HCEG_07372
HCEG_07373
HCEG_07374
HCEG_07375
4610000
4620000
4630000
4640000
Position on Supercontig_2.5

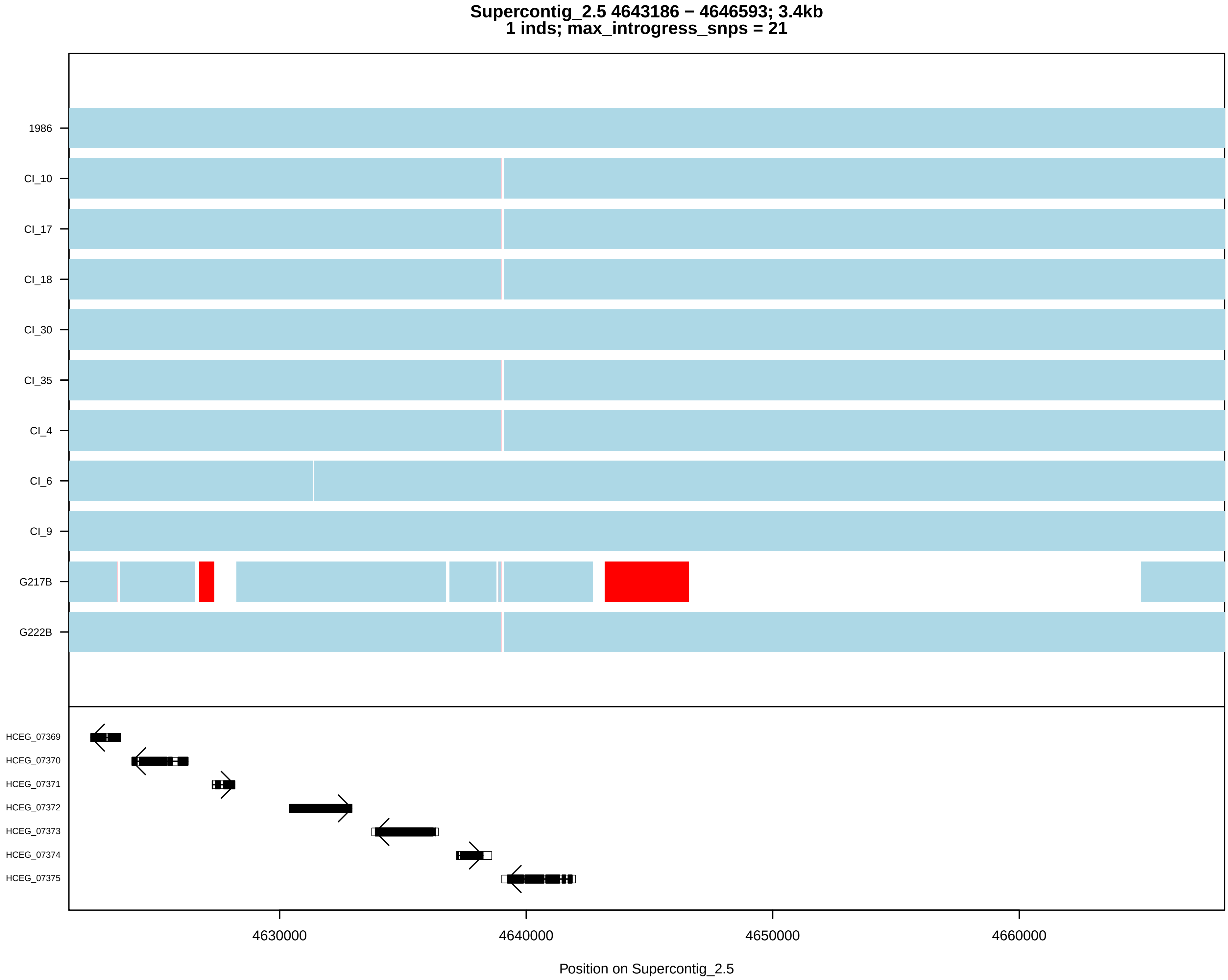
Supercontig_2.5 4643186 − 4646593; 3.4kb
1 inds; max_introgress_snps = 21
1986
CI_10
CI_17
CI_18
CI_30
CI_35
CI_4
CI_6
CI_9
G217B
G222B
HCEG_07369
HCEG_07370
HCEG_07371
HCEG_07372
HCEG_07373
HCEG_07374
HCEG_07375
4630000
4640000
4650000
4660000
Position on Supercontig_2.5

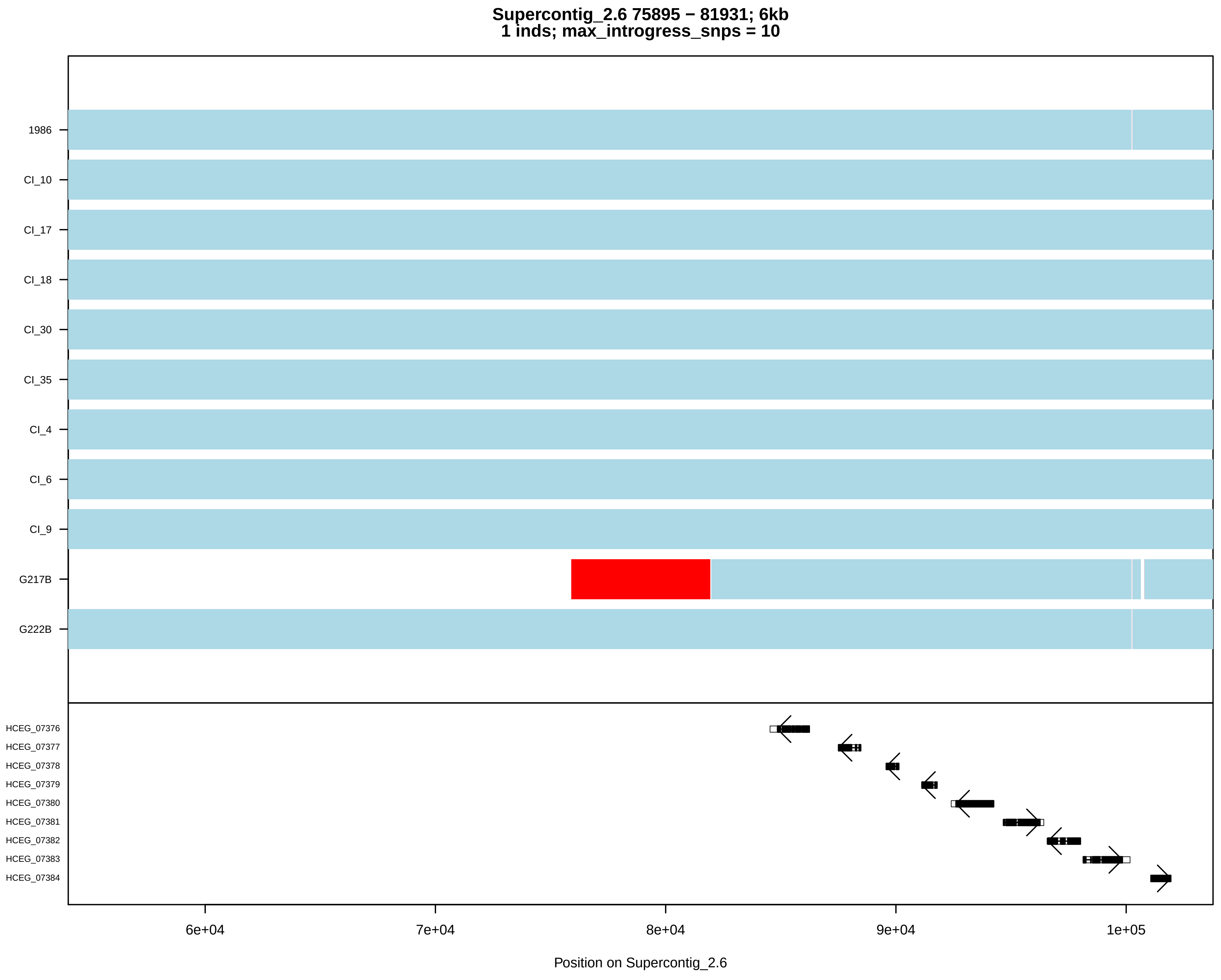

Supercontig_2.6 75895 − 81931; 6kb
1 inds; max_introgress_snps = 10
1986
CI_10
CI_17
CI_18
CI_30
CI_35
CI_4
CI_6
CI_9
G217B
G222B
HCEG_07376
HCEG_07377
HCEG_07378
HCEG_07379
HCEG_07380
HCEG_07381
HCEG_07382
HCEG_07383
HCEG_07384
6e+04
7e+04
8e+04
9e+04
1e+05
Position on Supercontig_2.6

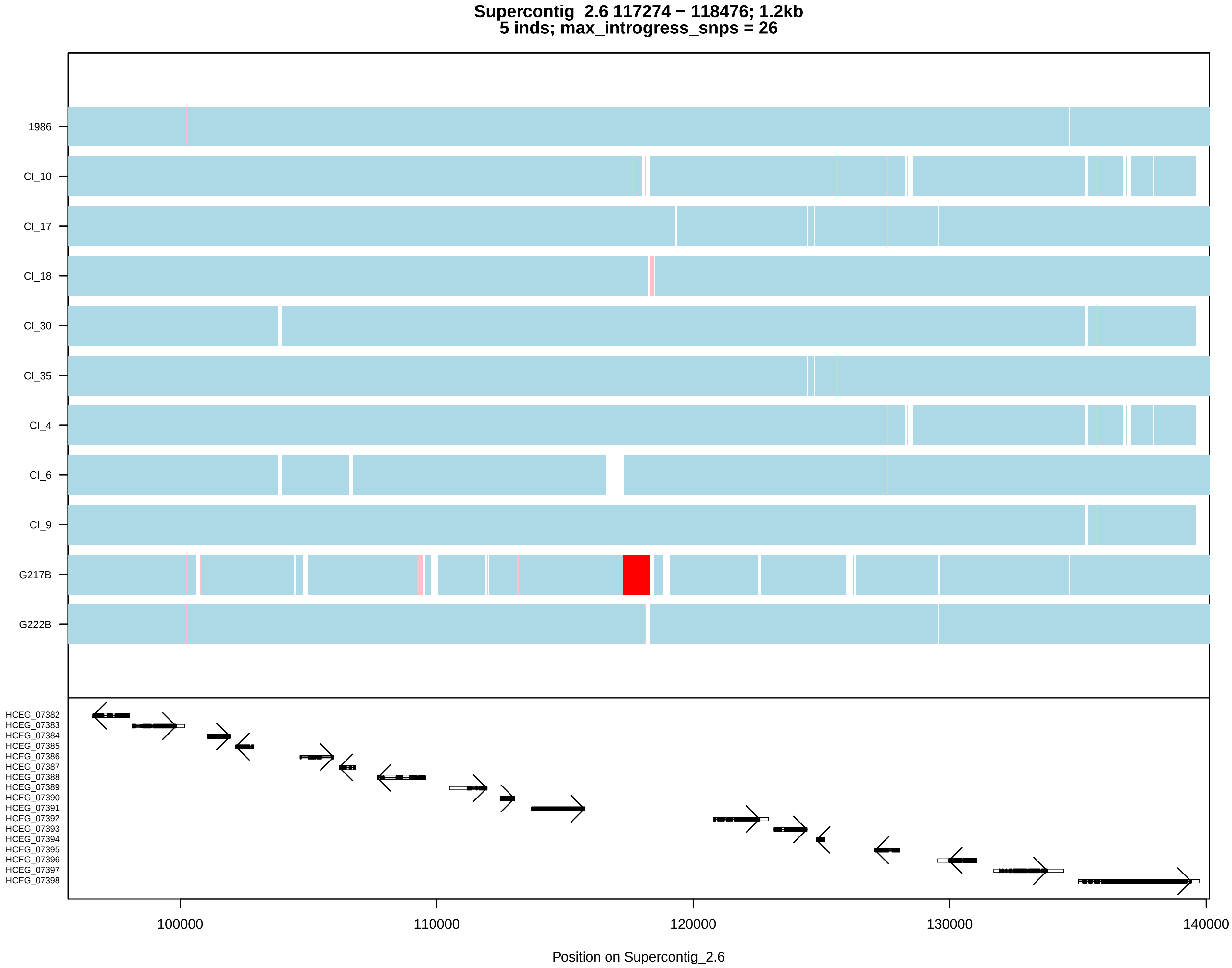

Supercontig_2.6 117274 − 118476; 1.2kb
5 inds; max_introgress_snps = 26
1986
CI_10
CI_17
CI_18
CI_30
CI_35
CI_4
CI_6
CI_9
G217B
G222B
HCEG_07382
HCEG_07383
HCEG_07384
HCEG_07385
HCEG_07386
HCEG_07387
HCEG_07388
HCEG_07389
HCEG_07390
HCEG_07391
HCEG_07392
HCEG_07393
HCEG_07394
HCEG_07395
HCEG_07396
HCEG_07397
HCEG_07398
100000
110000
120000
130000
140000
Position on Supercontig_2.6

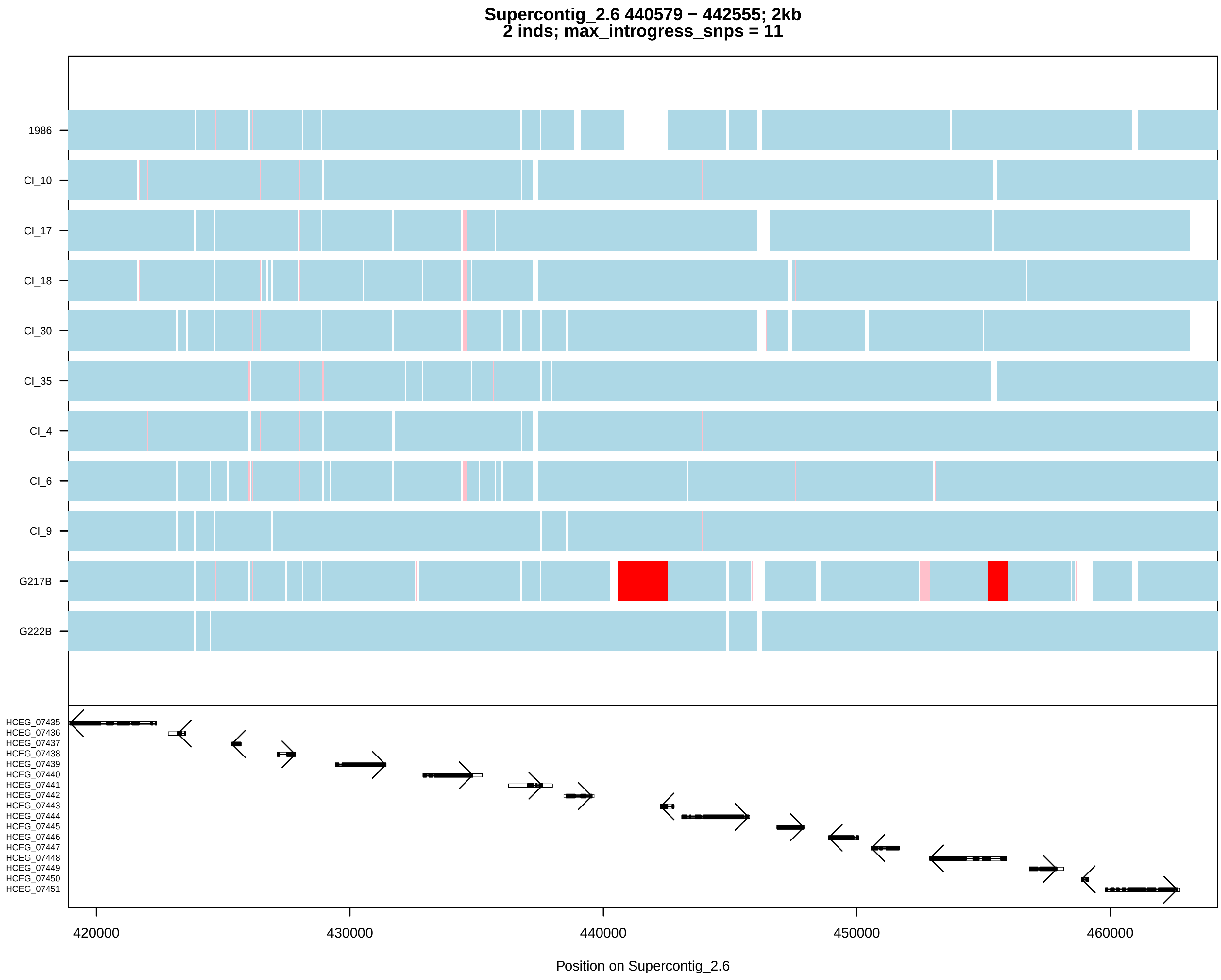

Supercontig_2.6 440579 − 442555; 2kb
2 inds; max_introgress_snps = 11
1986
CI_10
CI_17
CI_18
CI_30
CI_35
CI_4
CI_6
CI_9
G217B
G222B
HCEG_07435
HCEG_07436
HCEG_07437
HCEG_07438
HCEG_07439
HCEG_07440
HCEG_07441
HCEG_07442
HCEG_07443
HCEG_07444
HCEG_07445
HCEG_07446
HCEG_07447
HCEG_07448
HCEG_07449
HCEG_07450
HCEG_07451
420000
430000
440000
450000
460000
Position on Supercontig_2.6

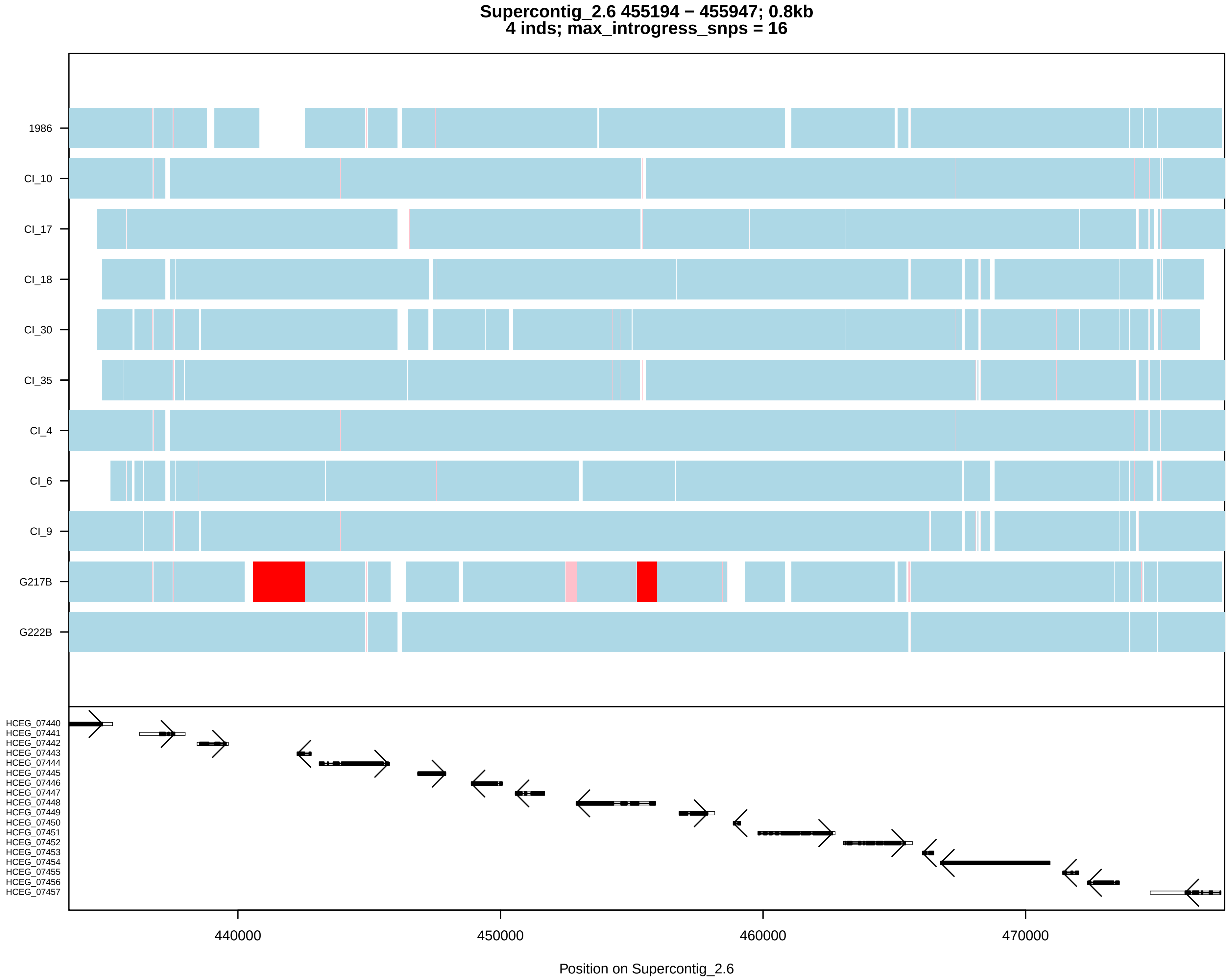
Supercontig_2.6 455194 − 455947; 0.8kb
4 inds; max_introgress_snps = 16
1986
CI_10
CI_17
CI_18
CI_30
CI_35
CI_4
CI_6
CI_9
G217B
G222B
HCEG_07440
HCEG_07441
HCEG_07442
HCEG_07443
HCEG_07444
HCEG_07445
HCEG_07446
HCEG_07447
HCEG_07448
HCEG_07449
HCEG_07450
HCEG_07451
HCEG_07452
HCEG_07453
HCEG_07454
HCEG_07455
HCEG_07456
HCEG_07457
440000
450000
460000
470000
Position on Supercontig_2.6

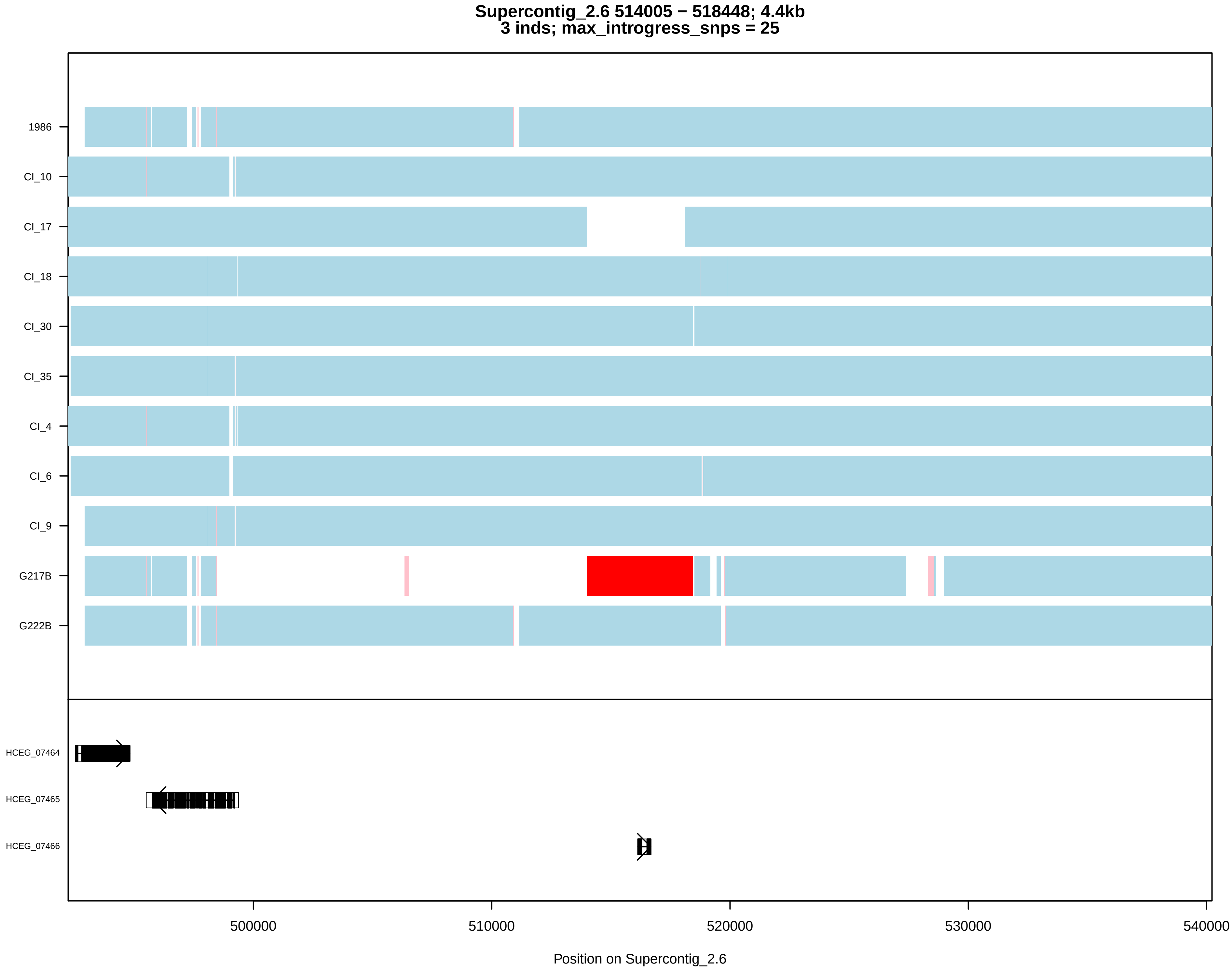
Supercontig_2.6 514005 − 518448; 4.4kb
3 inds; max_introgress_snps = 25
1986
CI_10
CI_17
CI_18
CI_30
CI_35
CI_4
CI_6
CI_9
G217B
G222B
HCEG_07464
HCEG_07465
HCEG_07466
500000
510000
520000
530000
540000
Position on Supercontig_2.6

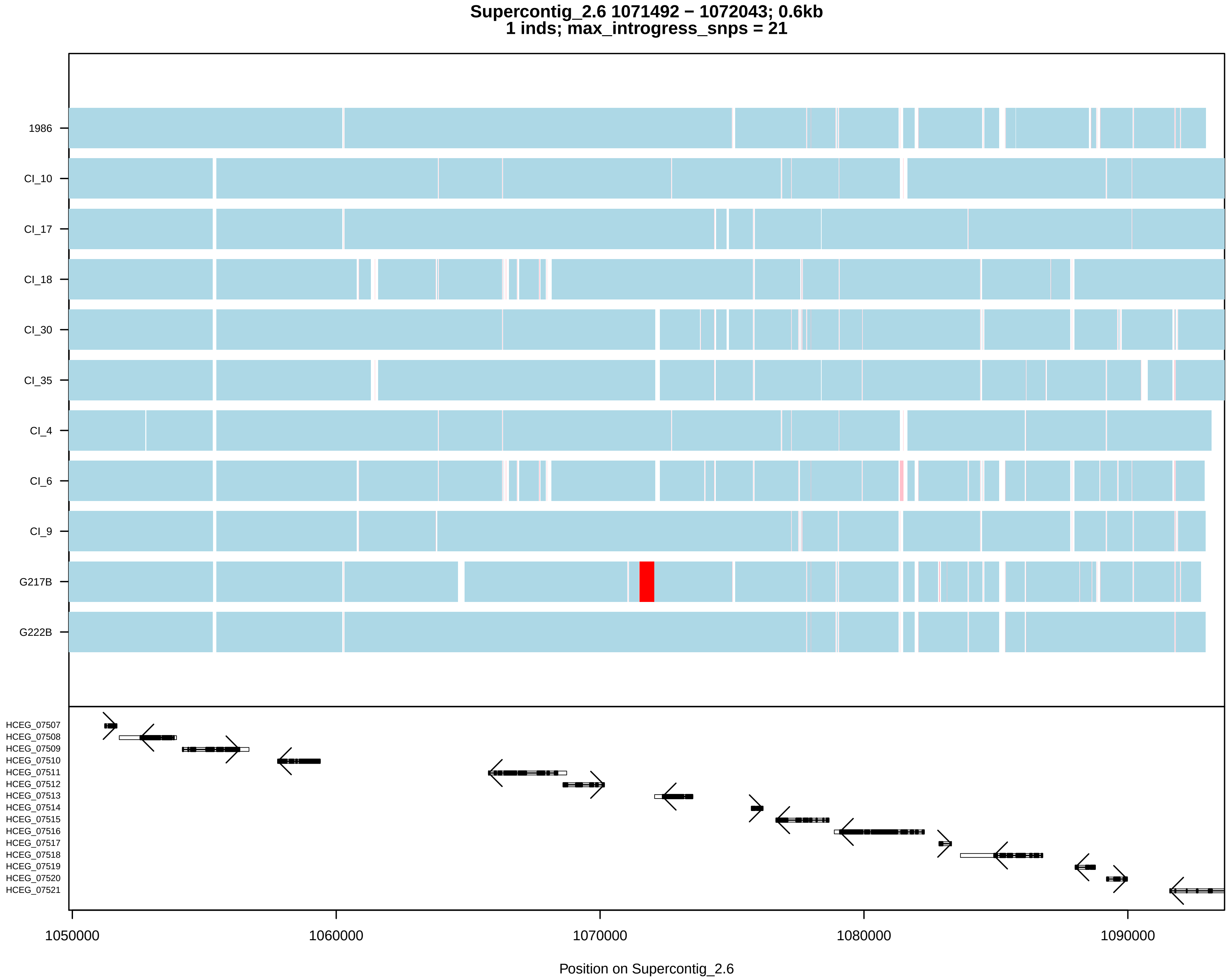
Supercontig_2.6 1071492 − 1072043; 0.6kb
1 inds; max_introgress_snps = 21
1986
CI_10
CI_17
CI_18
CI_30
CI_35
CI_4
CI_6
CI_9
G217B
G222B
HCEG_07507
HCEG_07508
HCEG_07509
HCEG_07510
HCEG_07511
HCEG_07512
HCEG_07513
HCEG_07514
HCEG_07515
HCEG_07516
HCEG_07517
HCEG_07518
HCEG_07519
HCEG_07520
HCEG_07521
1050000
1060000
1070000
1080000
1090000
Position on Supercontig_2.6

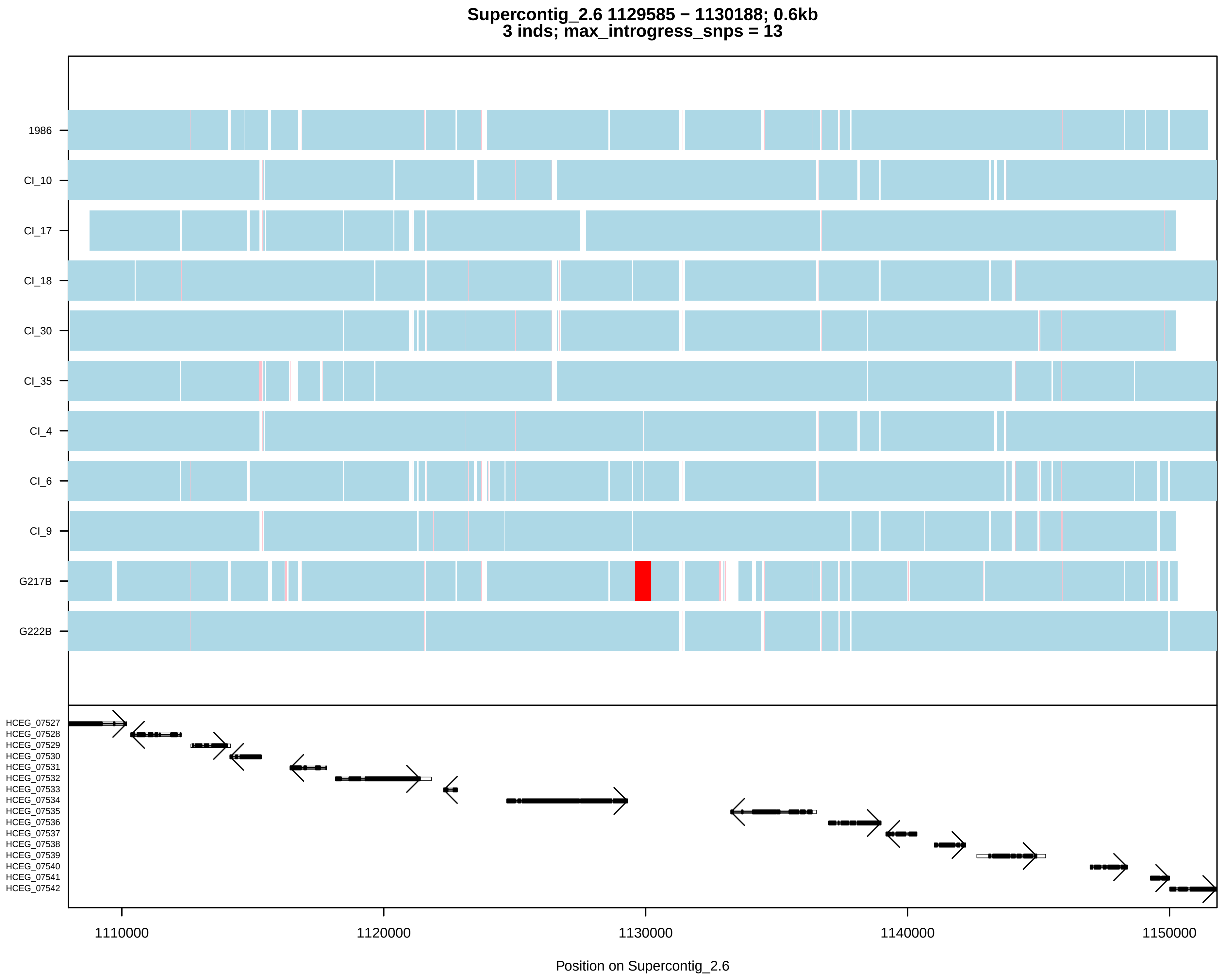

Supercontig_2.6 1129585 − 1130188; 0.6kb
3 inds; max_introgress_snps = 13
1986
CI_10
CI_17
CI_18
CI_30
CI_35
CI_4
CI_6
CI_9
G217B
G222B
HCEG_07527
HCEG_07528
HCEG_07529
HCEG_07530
HCEG_07531
HCEG_07532
HCEG_07533
HCEG_07534
HCEG_07535
HCEG_07536
HCEG_07537
HCEG_07538
HCEG_07539
HCEG_07540
HCEG_07541
HCEG_07542
1110000
1120000
1130000
1140000
1150000
Position on Supercontig_2.6

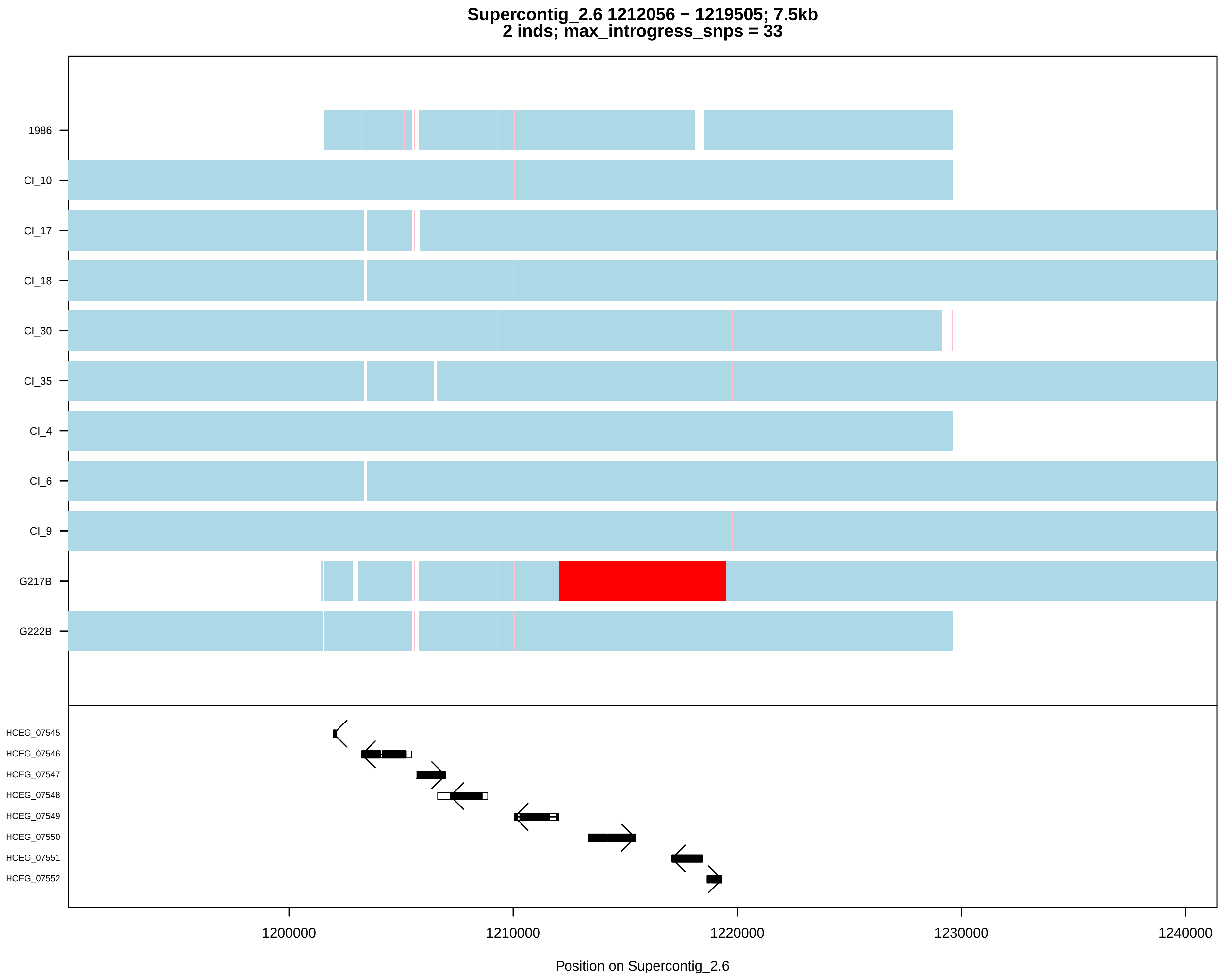

Supercontig_2.6 1212056 − 1219505; 7.5kb
2 inds; max_introgress_snps = 33
1986
CI_10
CI_17
CI_18
CI_30
CI_35
CI_4
CI_6
CI_9
G217B
G222B
HCEG_07545
HCEG_07546
HCEG_07547
HCEG_07548
HCEG_07549
HCEG_07550
HCEG_07551
HCEG_07552
1200000
1210000
1220000
1230000
1240000
Position on Supercontig_2.6

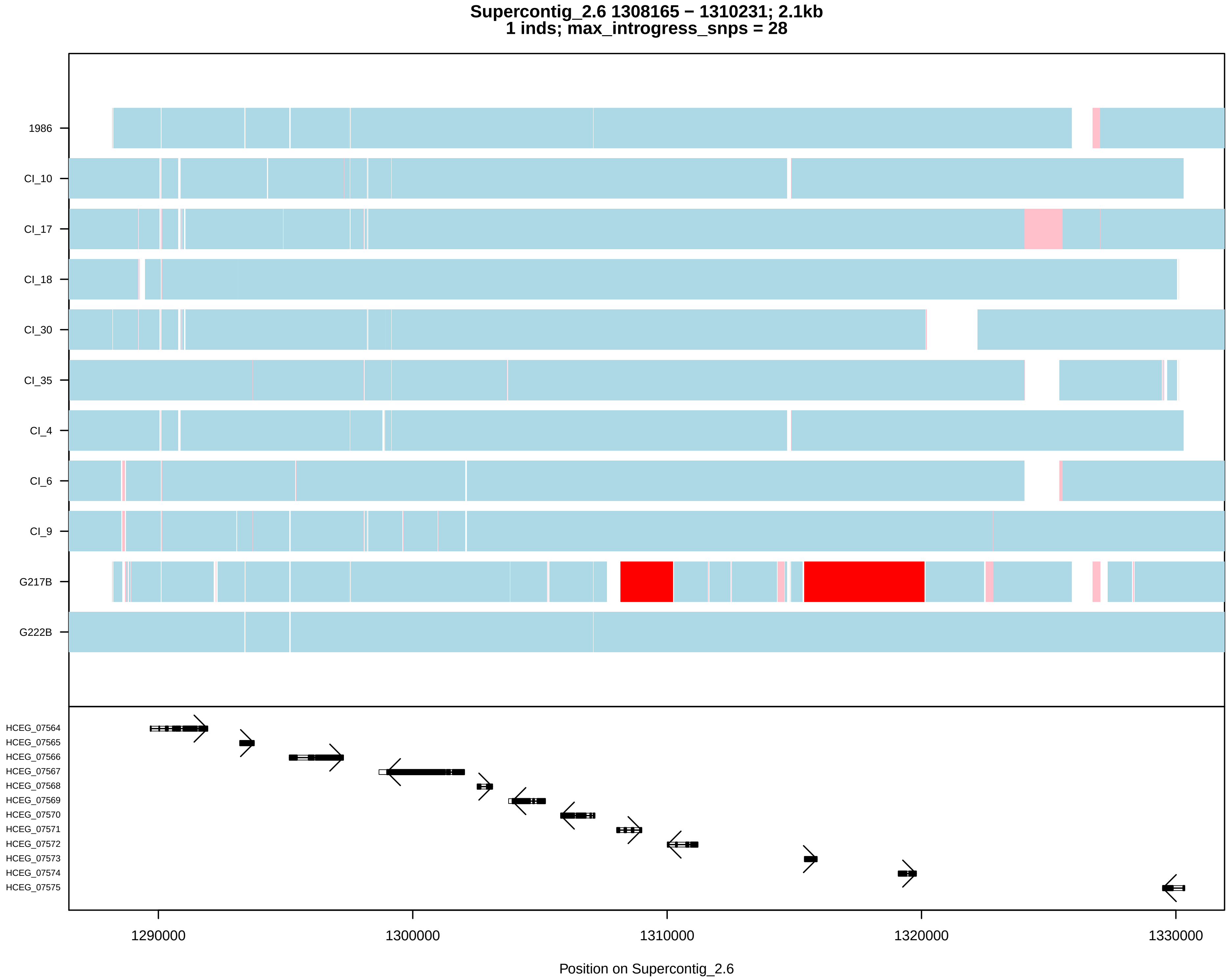

Supercontig_2.6 1308165 − 1310231; 2.1kb
1 inds; max_introgress_snps = 28
1986
CI_10
CI_17
CI_18
CI_30
CI_35
CI_4
CI_6
CI_9
G217B
G222B
HCEG_07564
HCEG_07565
HCEG_07566
HCEG_07567
HCEG_07568
HCEG_07569
HCEG_07570
HCEG_07571
HCEG_07572
HCEG_07573
HCEG_07574
HCEG_07575
1290000
1300000
1310000
1320000
1330000
Position on Supercontig_2.6

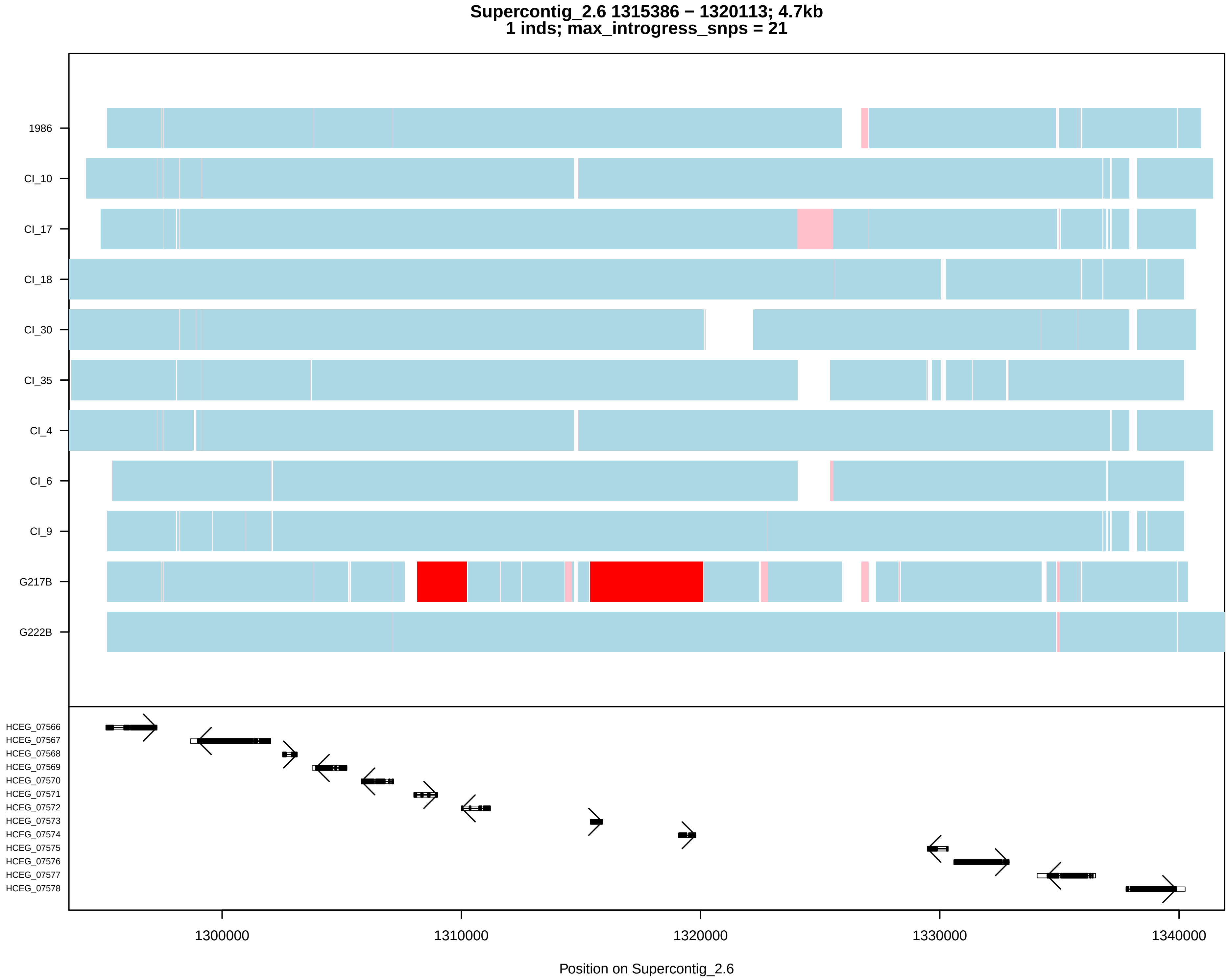

Supercontig_2.6 1315386 − 1320113; 4.7kb
1 inds; max_introgress_snps = 21
1986
CI_10
CI_17
CI_18
CI_30
CI_35
CI_4
CI_6
CI_9
G217B
G222B
HCEG_07566
HCEG_07567
HCEG_07568
HCEG_07569
HCEG_07570
HCEG_07571
HCEG_07572
HCEG_07573
HCEG_07574
HCEG_07575
HCEG_07576
HCEG_07577
HCEG_07578
1300000
1310000
1320000
1330000
1340000
Position on Supercontig_2.6

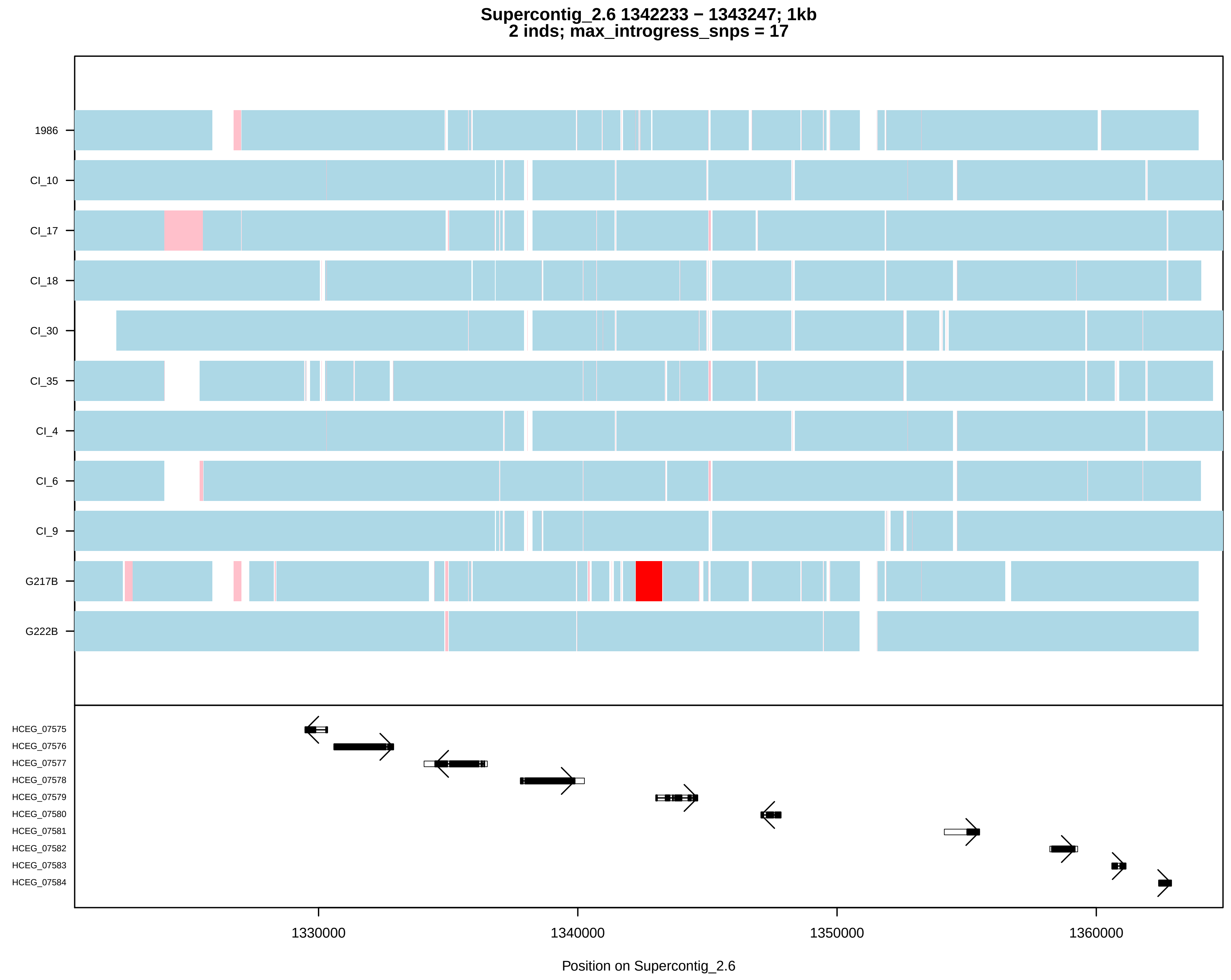

Supercontig_2.6 1342233 − 1343247; 1kb
2 inds; max_introgress_snps = 17
1986
CI_10
CI_17
CI_18
CI_30
CI_35
CI_4
CI_6
CI_9
G217B
G222B
HCEG_07575
HCEG_07576
HCEG_07577
HCEG_07578
HCEG_07579
HCEG_07580
HCEG_07581
HCEG_07582
HCEG_07583
HCEG_07584
1330000
1340000
1350000
1360000
Position on Supercontig_2.6

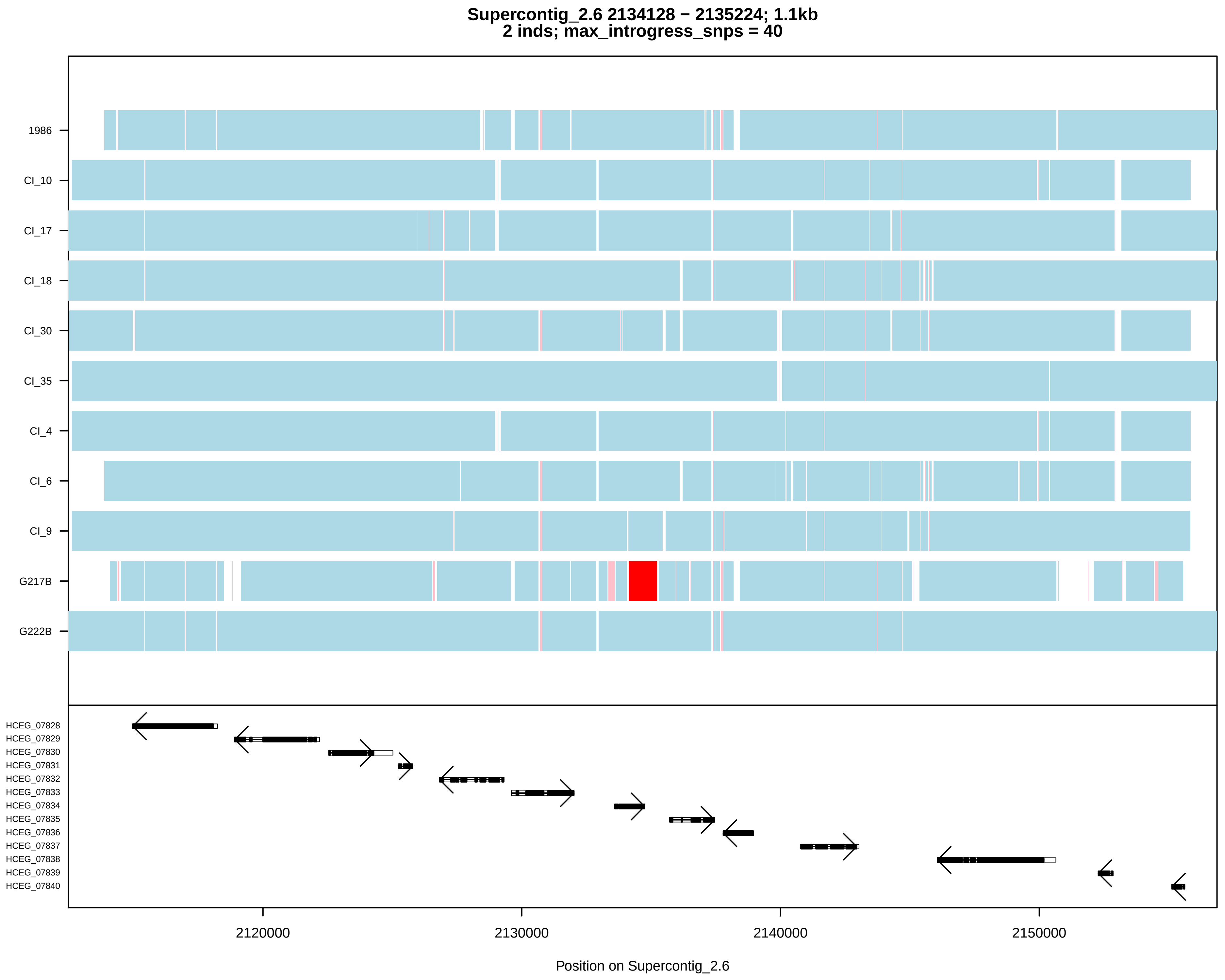

Supercontig_2.6 2134128 − 2135224; 1.1kb
2 inds; max_introgress_snps = 40
1986
CI_10
CI_17
CI_18
CI_30
CI_35
CI_4
CI_6
CI_9
G217B
G222B
HCEG_07828
HCEG_07829
HCEG_07830
HCEG_07831
HCEG_07832
HCEG_07833
HCEG_07834
HCEG_07835
HCEG_07836
HCEG_07837
HCEG_07838
HCEG_07839
HCEG_07840
2120000
2130000
2140000
2150000
Position on Supercontig_2.6

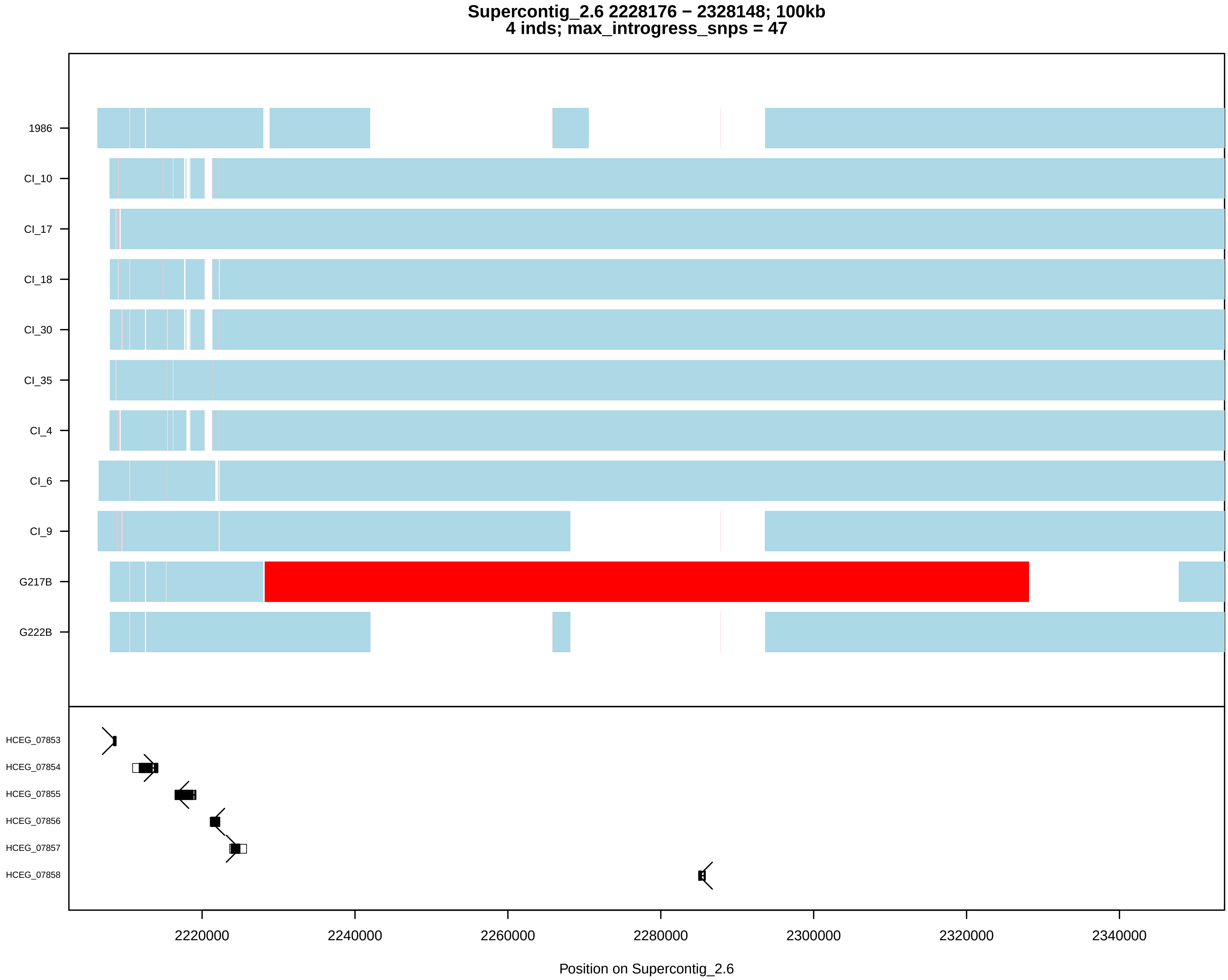
Supercontig_2.6 2228176 − 2328148; 100kb
4 inds; max_introgress_snps = 47
1986
CI_10
CI_17
CI_18
CI_30
CI_35
CI_4
CI_6
CI_9
G217B
G222B
HCEG_07853
HCEG_07854
HCEG_07855
HCEG_07856
HCEG_07857
HCEG_07858
2220000
2240000
2260000
2280000
2300000
2320000
2340000
Position on Supercontig_2.6

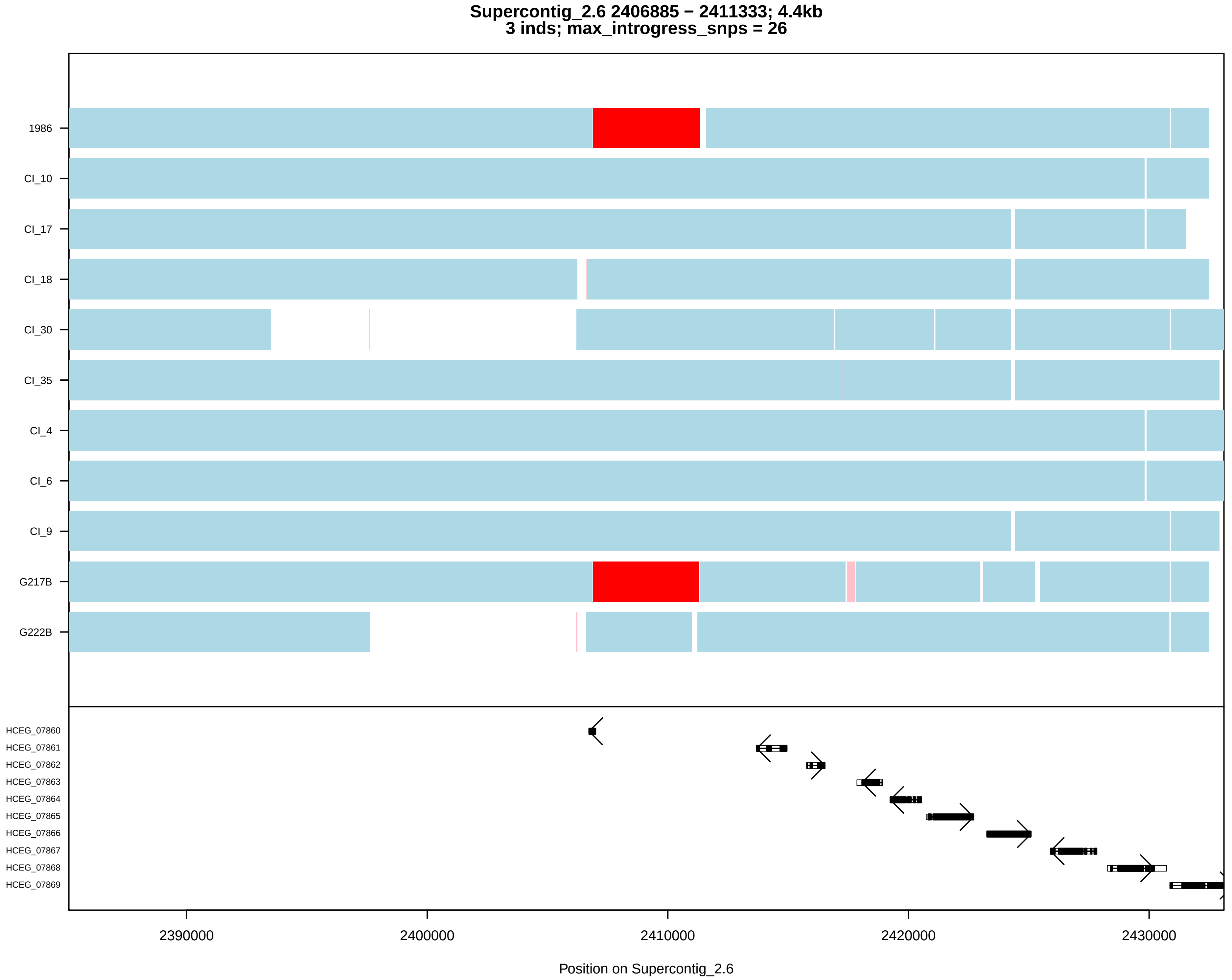
Supercontig_2.6 2406885 − 2411333; 4.4kb
3 inds; max_introgress_snps = 26
1986
CI_10
CI_17
CI_18
CI_30
CI_35
CI_4
CI_6
CI_9
G217B
G222B
HCEG_07860
HCEG_07861
HCEG_07862
HCEG_07863
HCEG_07864
HCEG_07865
HCEG_07866
HCEG_07867
HCEG_07868
HCEG_07869
2390000
2400000
2410000
2420000
2430000
Position on Supercontig_2.6

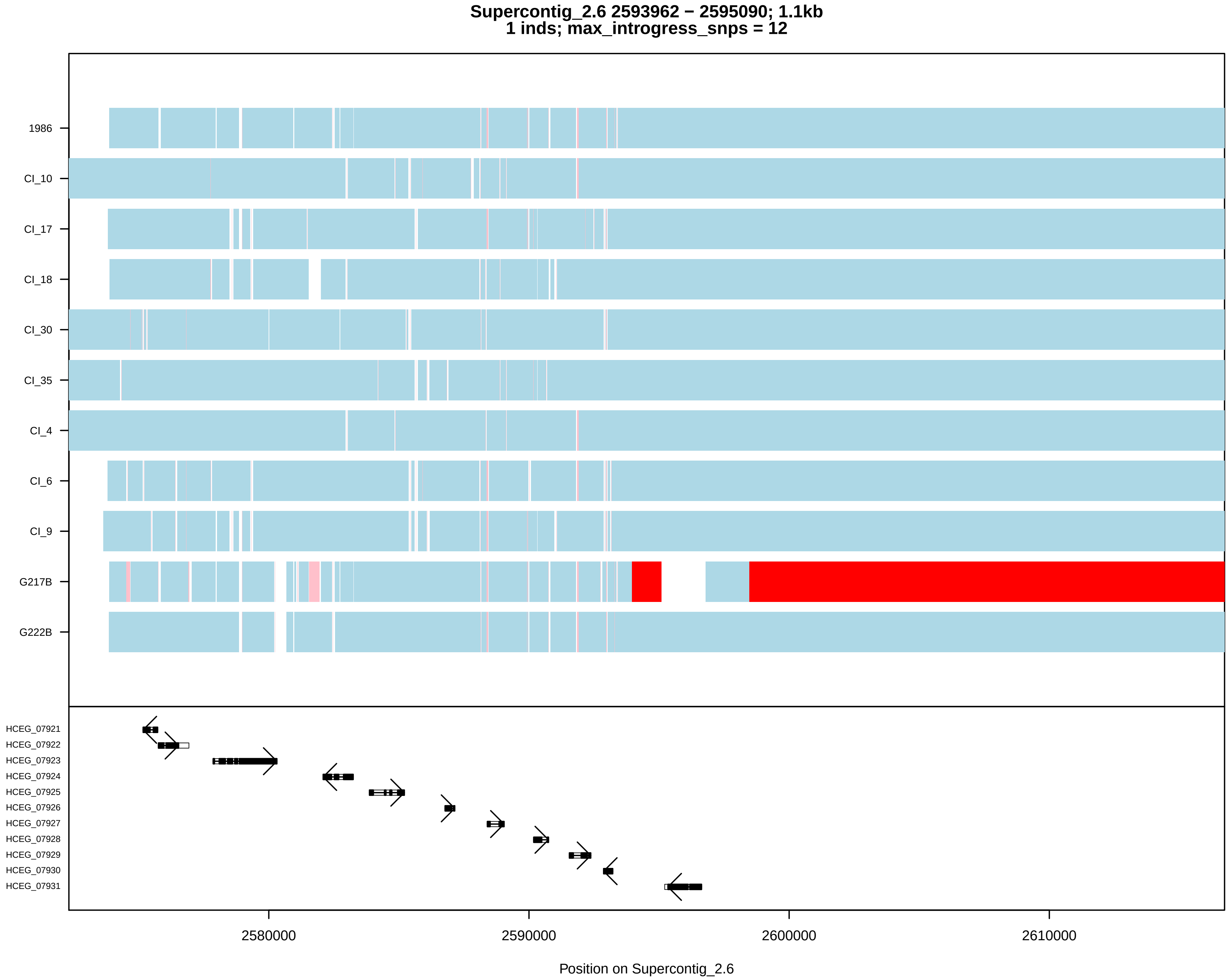
Supercontig_2.6 2593962 − 2595090; 1.1kb
1 inds; max_introgress_snps = 12
1986
CI_10
CI_17
CI_18
CI_30
CI_35
CI_4
CI_6
CI_9
G217B
G222B
HCEG_07921
HCEG_07922
HCEG_07923
HCEG_07924
HCEG_07925
HCEG_07926
HCEG_07927
HCEG_07928
HCEG_07929
HCEG_07930
HCEG_07931
2580000
2590000
2600000
2610000
Position on Supercontig_2.6

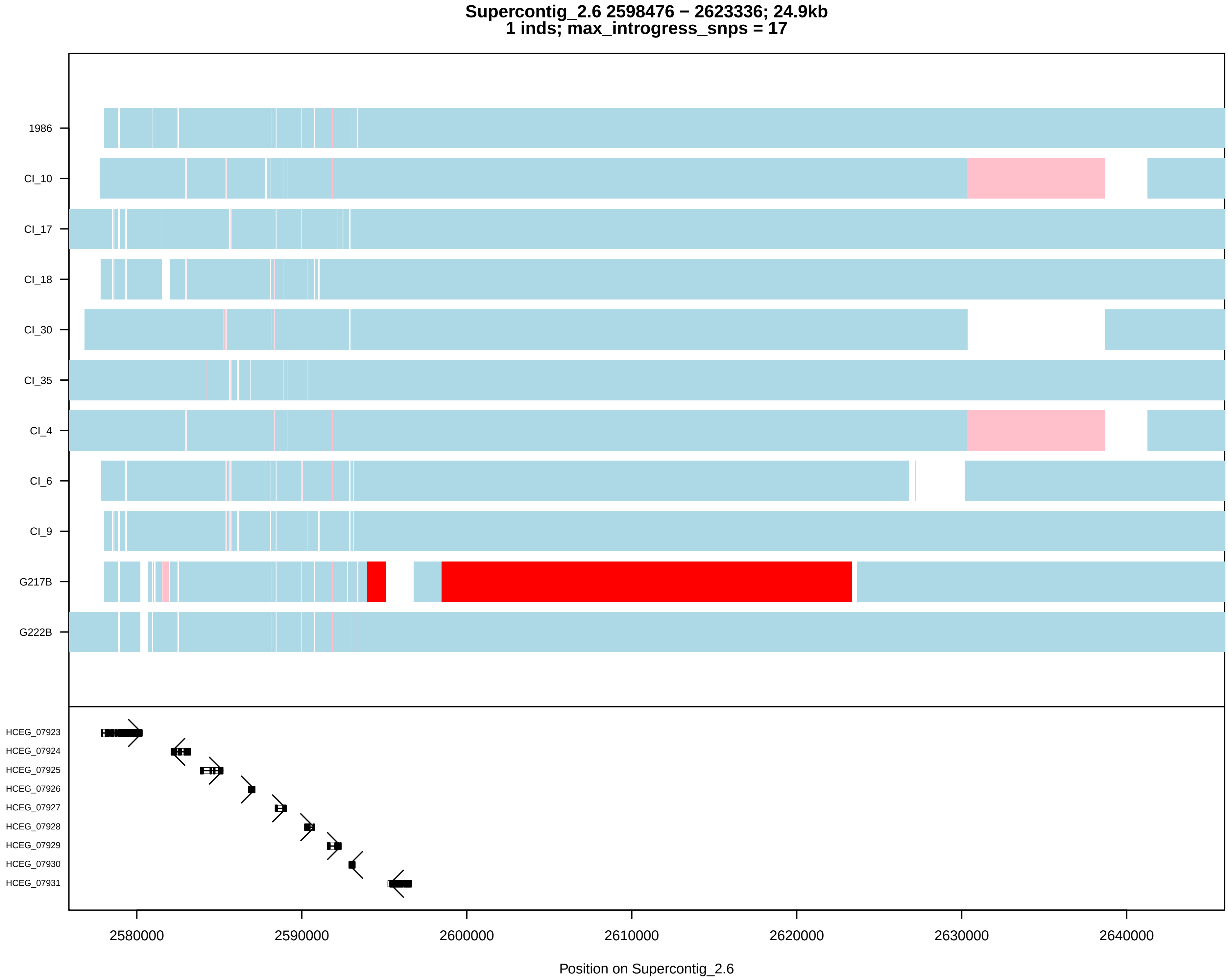

Supercontig_2.6 2598476 − 2623336; 24.9kb
1 inds; max_introgress_snps = 17
1986
CI_10
CI_17
CI_18
CI_30
CI_35
CI_4
CI_6
CI_9
G217B
G222B
HCEG_07923
HCEG_07924
HCEG_07925
HCEG_07926
HCEG_07927
HCEG_07928
HCEG_07929
HCEG_07930
HCEG_07931
2580000
2590000
2600000
2610000
2620000
2630000
2640000
Position on Supercontig_2.6

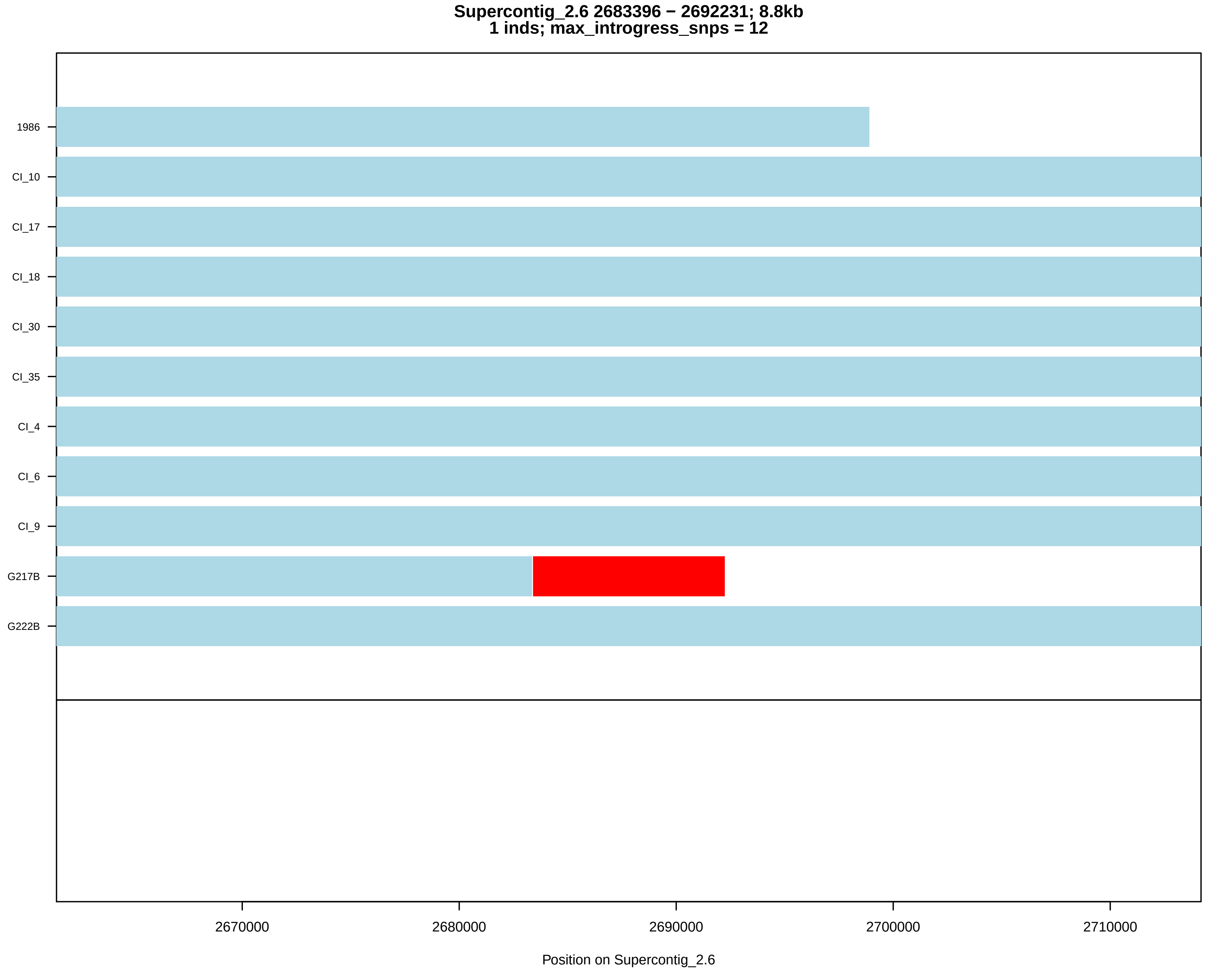
Supercontig_2.6 2683396 − 2692231; 8.8kb
1 inds; max_introgress_snps = 12
1986
CI_10
CI_17
CI_18
CI_30
CI_35
CI_4
CI_6
CI_9
G217B
G222B
2670000
2680000
2690000
2700000
2710000
Position on Supercontig_2.6

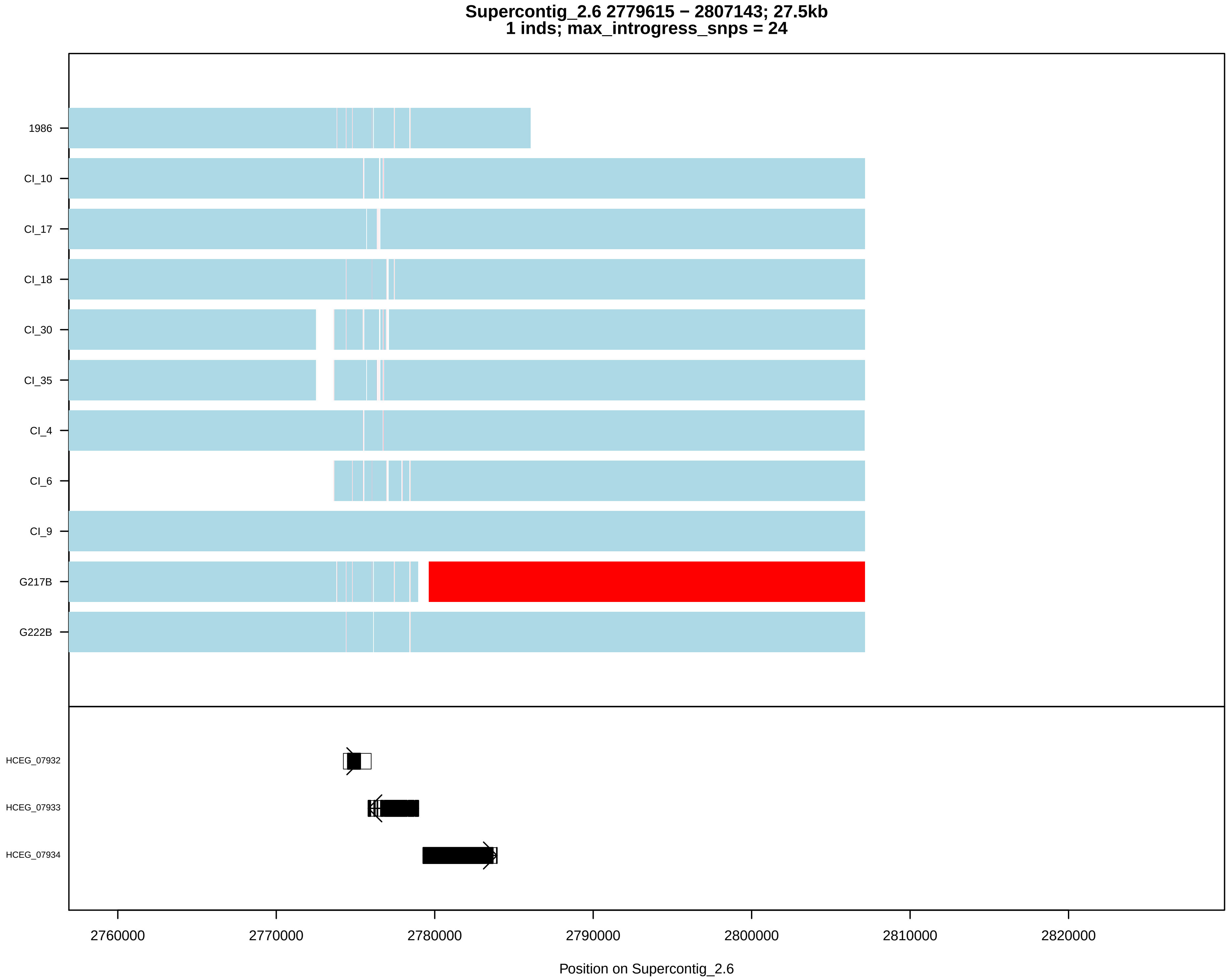

Supercontig_2.6 2779615 − 2807143; 27.5kb
1 inds; max_introgress_snps = 24
1986
CI_10
CI_17
CI_18
CI_30
CI_35
CI_4
CI_6
CI_9
G217B
G222B
HCEG_07932
HCEG_07933
HCEG_07934
2760000
2770000
2780000
2790000
2800000
2810000
2820000
Position on Supercontig_2.6

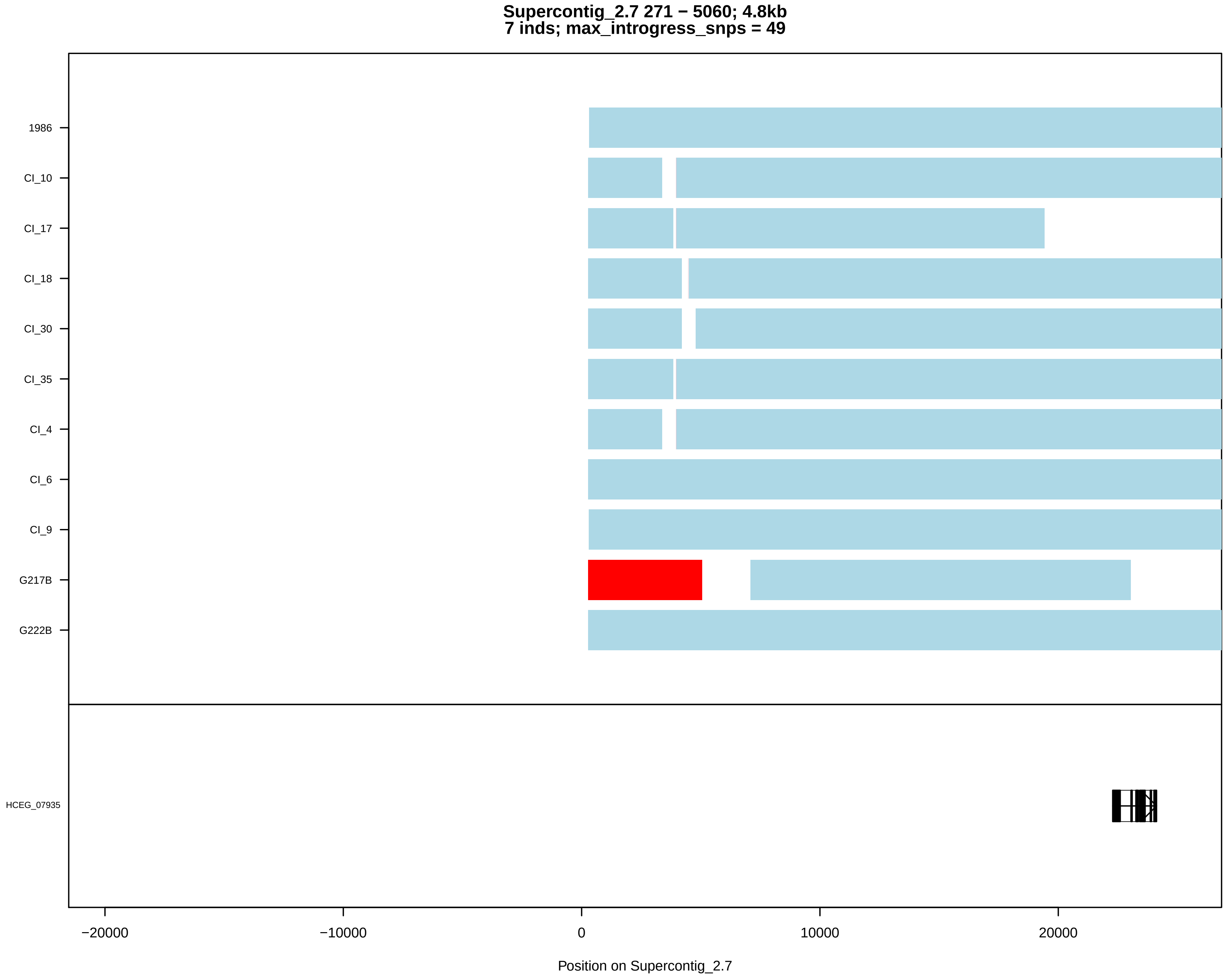
Supercontig_2.7 271 − 5060; 4.8kb
7 inds; max_introgress_snps = 49
1986
CI_10
CI_17
CI_18
CI_30
CI_35
CI_4
CI_6
CI_9
G217B
G222B
HCEG_07935
−20000
−10000
0
10000
20000
Position on Supercontig_2.7

Supercontig_2.7 25198 − 28511; 3.3kb
4 inds; max_introgress_snps = 31

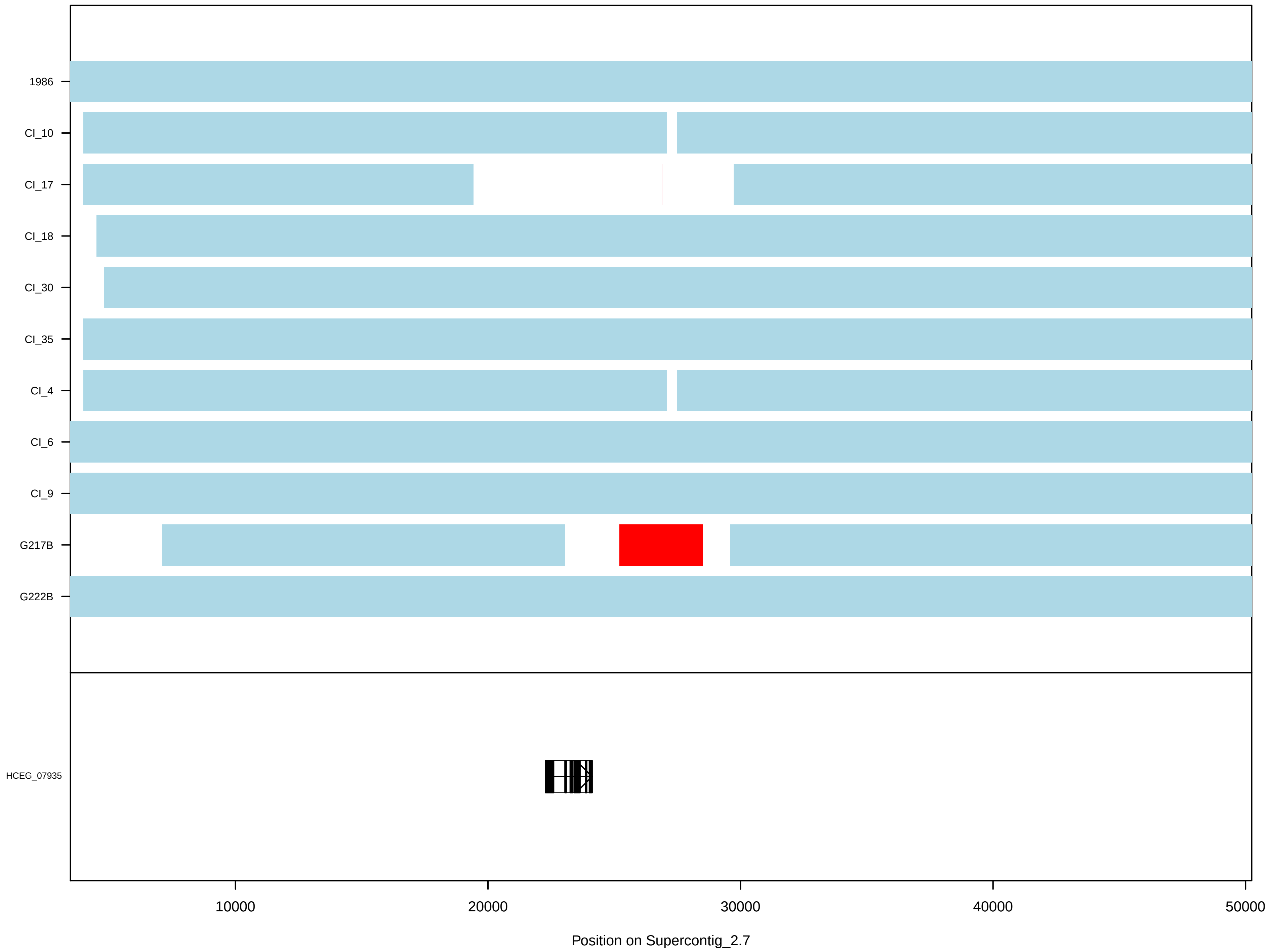

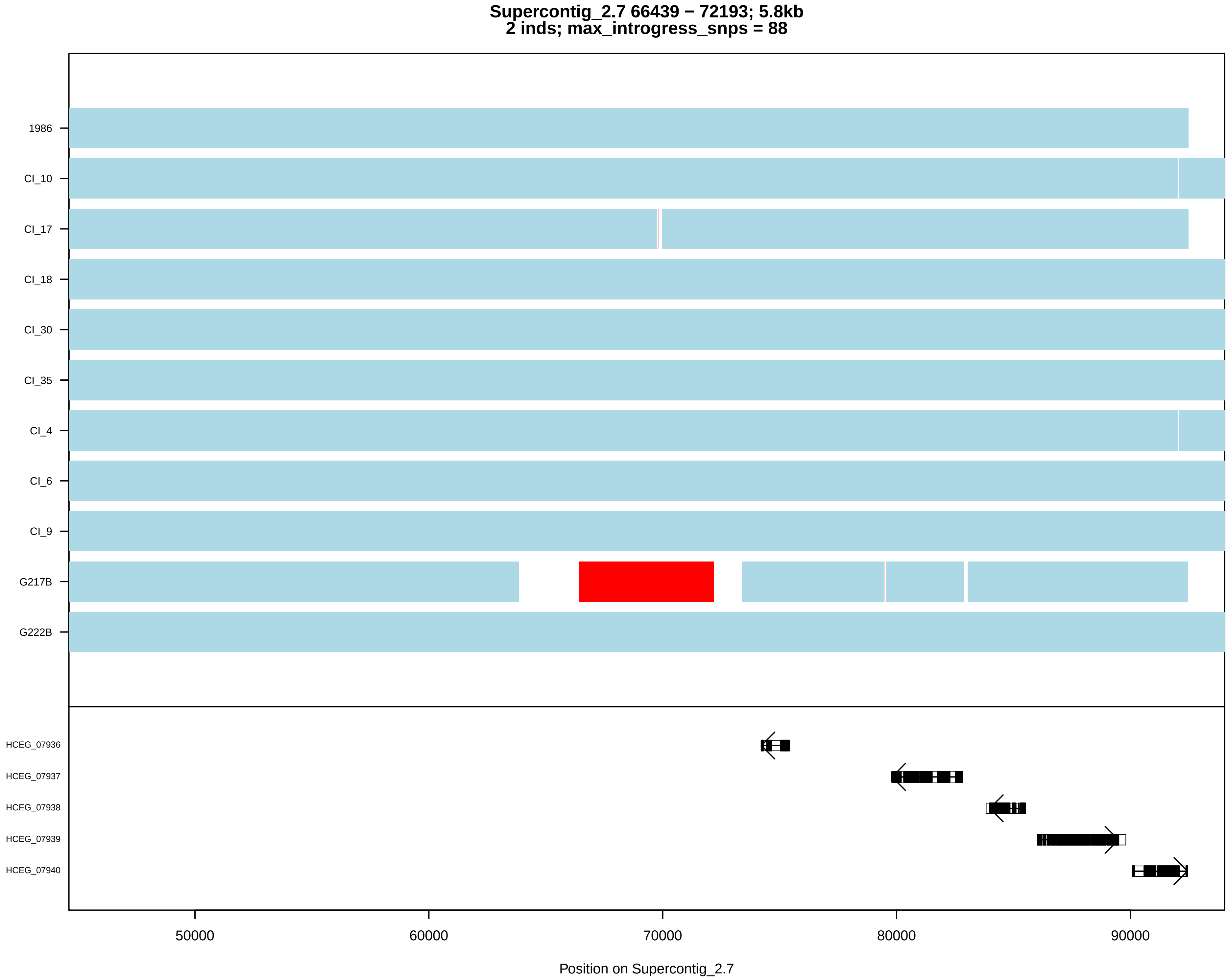

Supercontig_2.7 66439 − 72193; 5.8kb
2 inds; max_introgress_snps = 88
1986
CI_10
CI_17
CI_18
CI_30
CI_35
CI_4
CI_6
CI_9
G217B
G222B
HCEG_07936
HCEG_07937
HCEG_07938
HCEG_07939
HCEG_07940
50000
60000
70000
80000
90000
Position on Supercontig_2.7

Supercontig_2.7 343217 − 343999; 0.8kb
6 inds; max_introgress_snps = 13

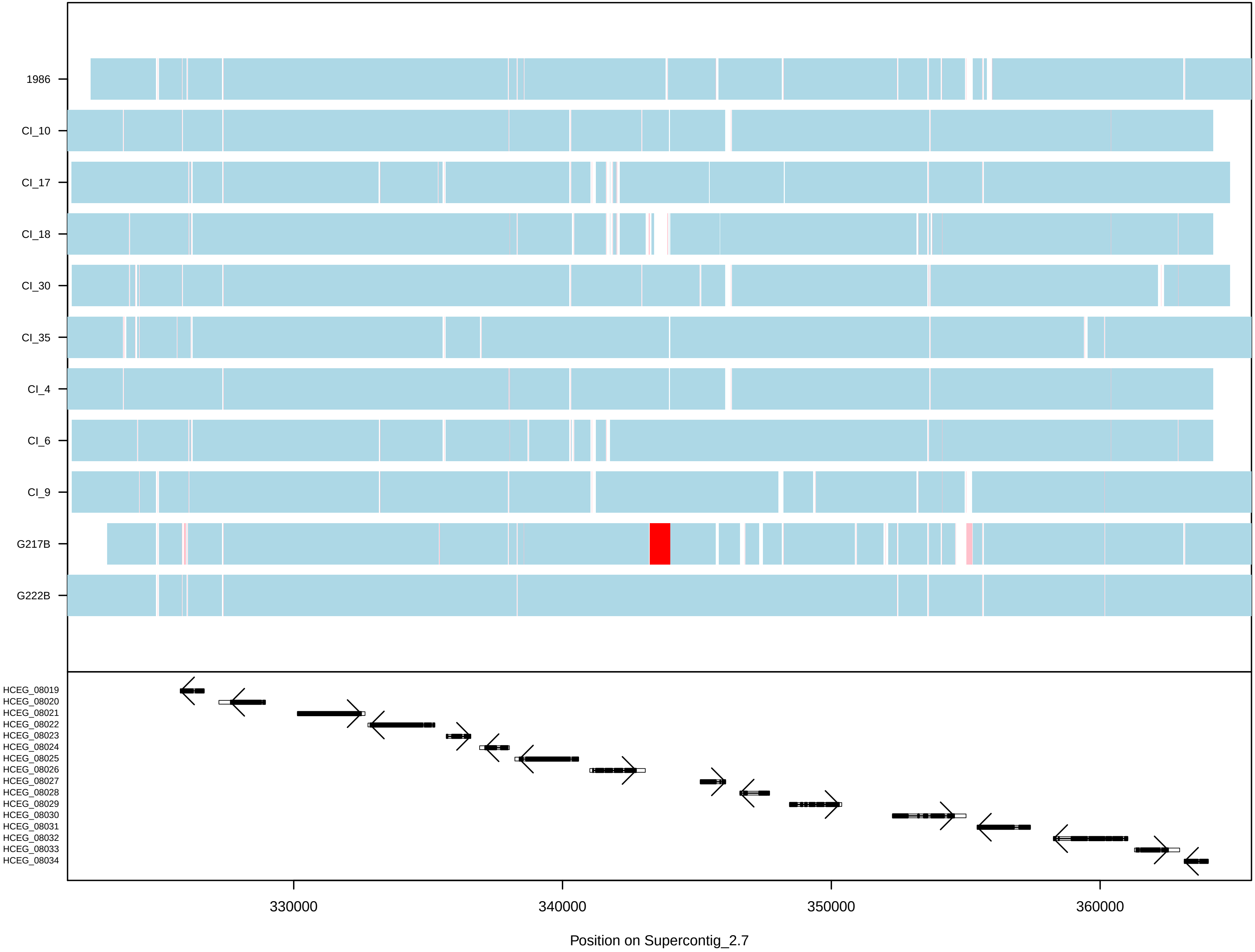

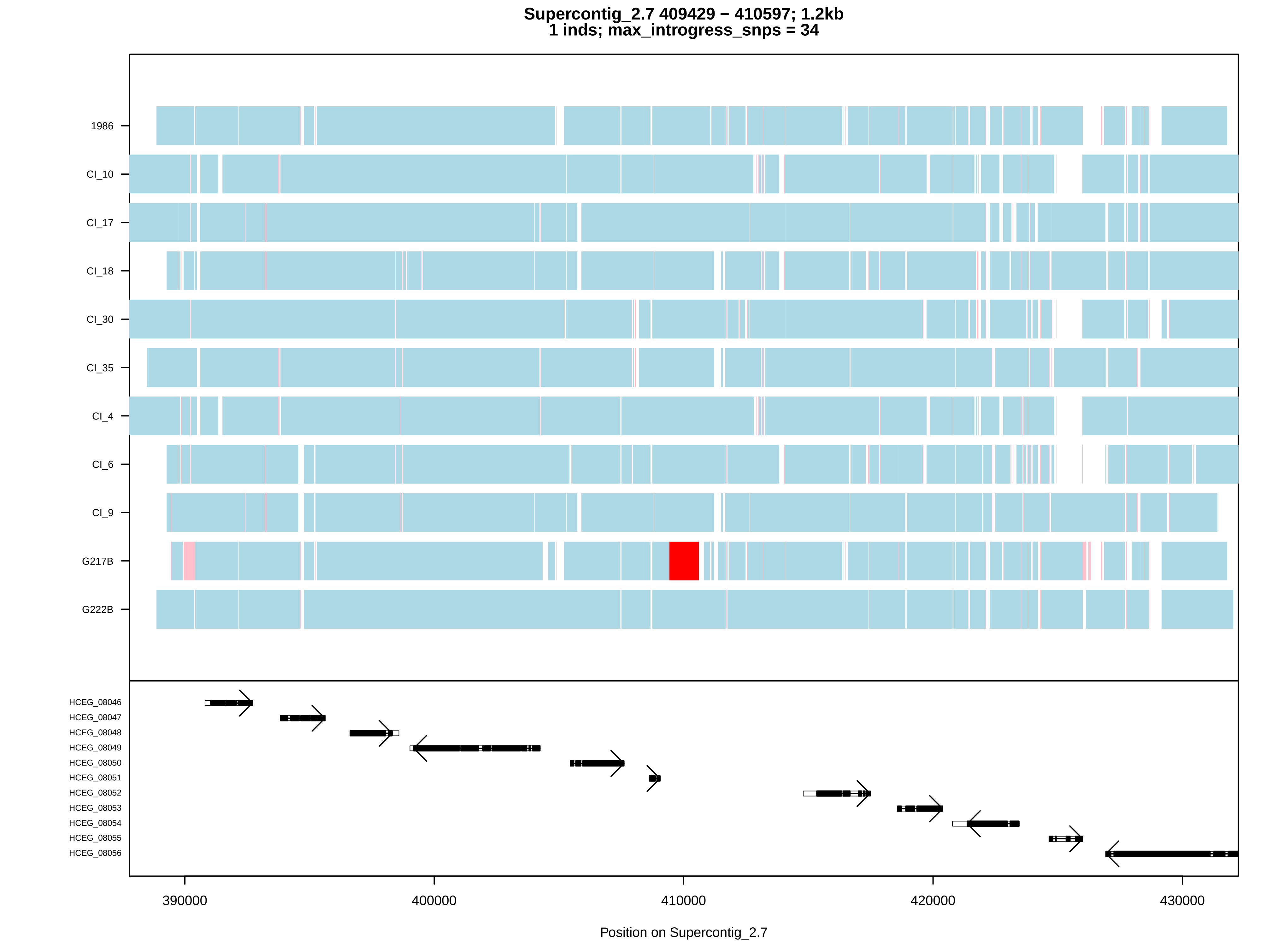
Supercontig_2.7 409429 − 410597; 1.2kb
1 inds; max_introgress_snps = 34
1986
CI_10
CI_17
CI_18
CI_30
CI_35
CI_4
CI_6
CI_9
G217B
G222B
HCEG_08046
HCEG_08047
HCEG_08048
HCEG_08049
HCEG_08050
HCEG_08051
HCEG_08052
HCEG_08053
HCEG_08054
HCEG_08055
HCEG_08056
390000
400000
410000
420000
430000
Position on Supercontig_2.7

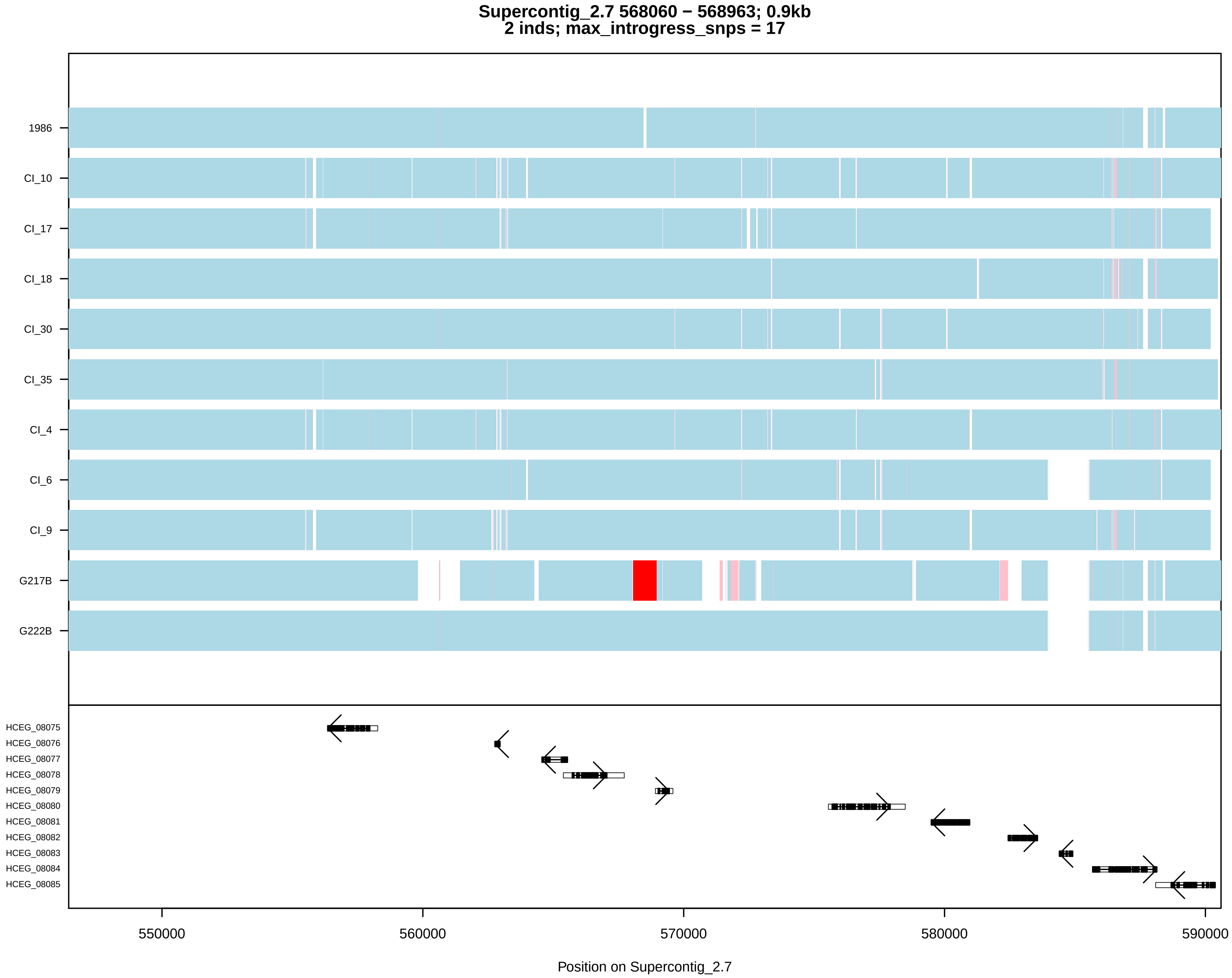

Supercontig_2.7 568060 − 568963; 0.9kb
2 inds; max_introgress_snps = 17
1986
CI_10
CI_17
CI_18
CI_30
CI_35
CI_4
CI_6
CI_9
G217B
G222B
HCEG_08075
HCEG_08076
HCEG_08077
HCEG_08078
HCEG_08079
HCEG_08080
HCEG_08081
HCEG_08082
HCEG_08083
HCEG_08084
HCEG_08085
550000
560000
570000
580000
590000
Position on Supercontig_2.7

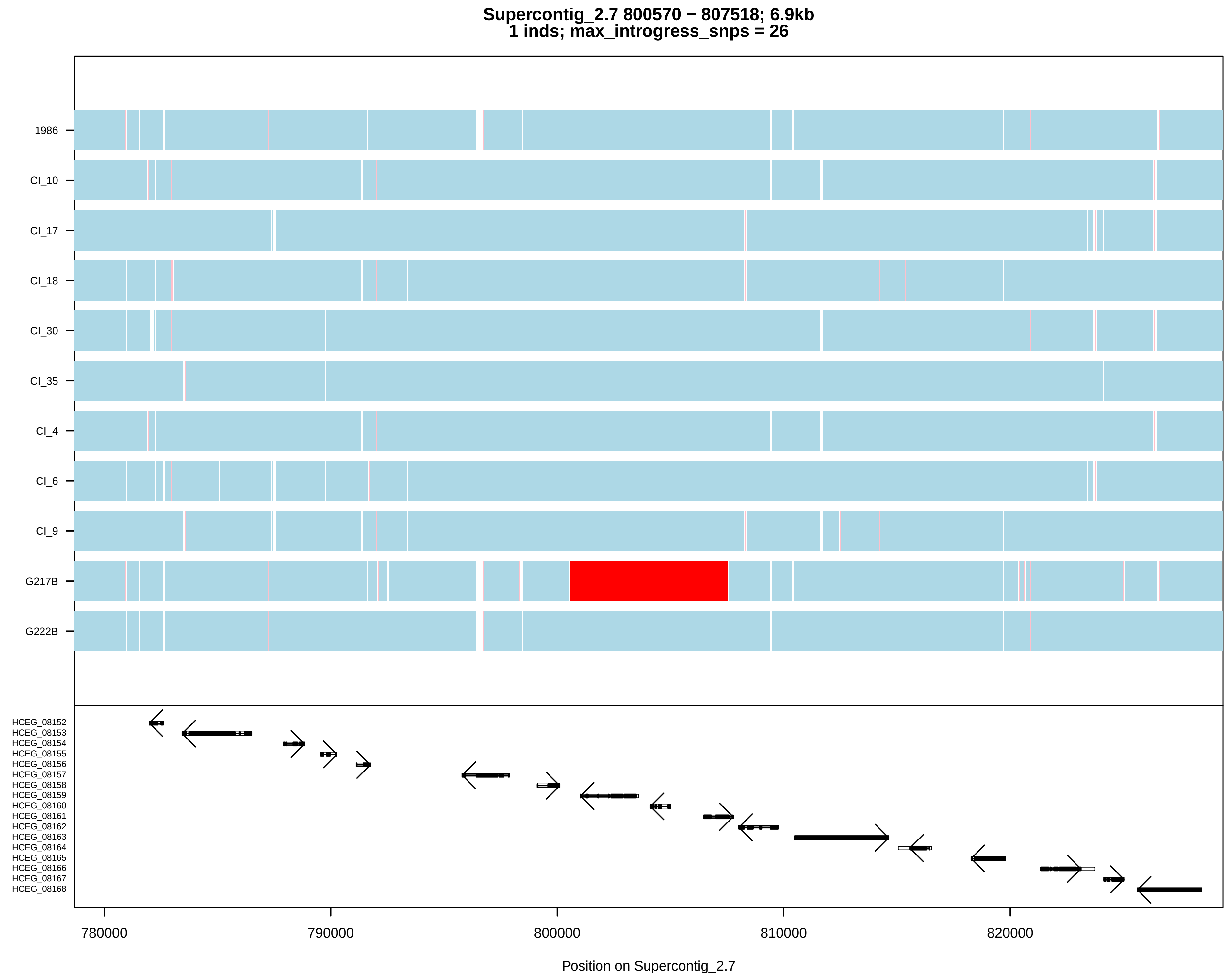

Supercontig_2.7 800570 − 807518; 6.9kb
1 inds; max_introgress_snps = 26
1986
CI_10
CI_17
CI_18
CI_30
CI_35
CI_4
CI_6
CI_9
G217B
G222B
HCEG_08152
HCEG_08153
HCEG_08154
HCEG_08155
HCEG_08156
HCEG_08157
HCEG_08158
HCEG_08159
HCEG_08160
HCEG_08161
HCEG_08162
HCEG_08163
HCEG_08164
HCEG_08165
HCEG_08166
HCEG_08167
HCEG_08168
780000
790000
800000
810000
820000
Position on Supercontig_2.7

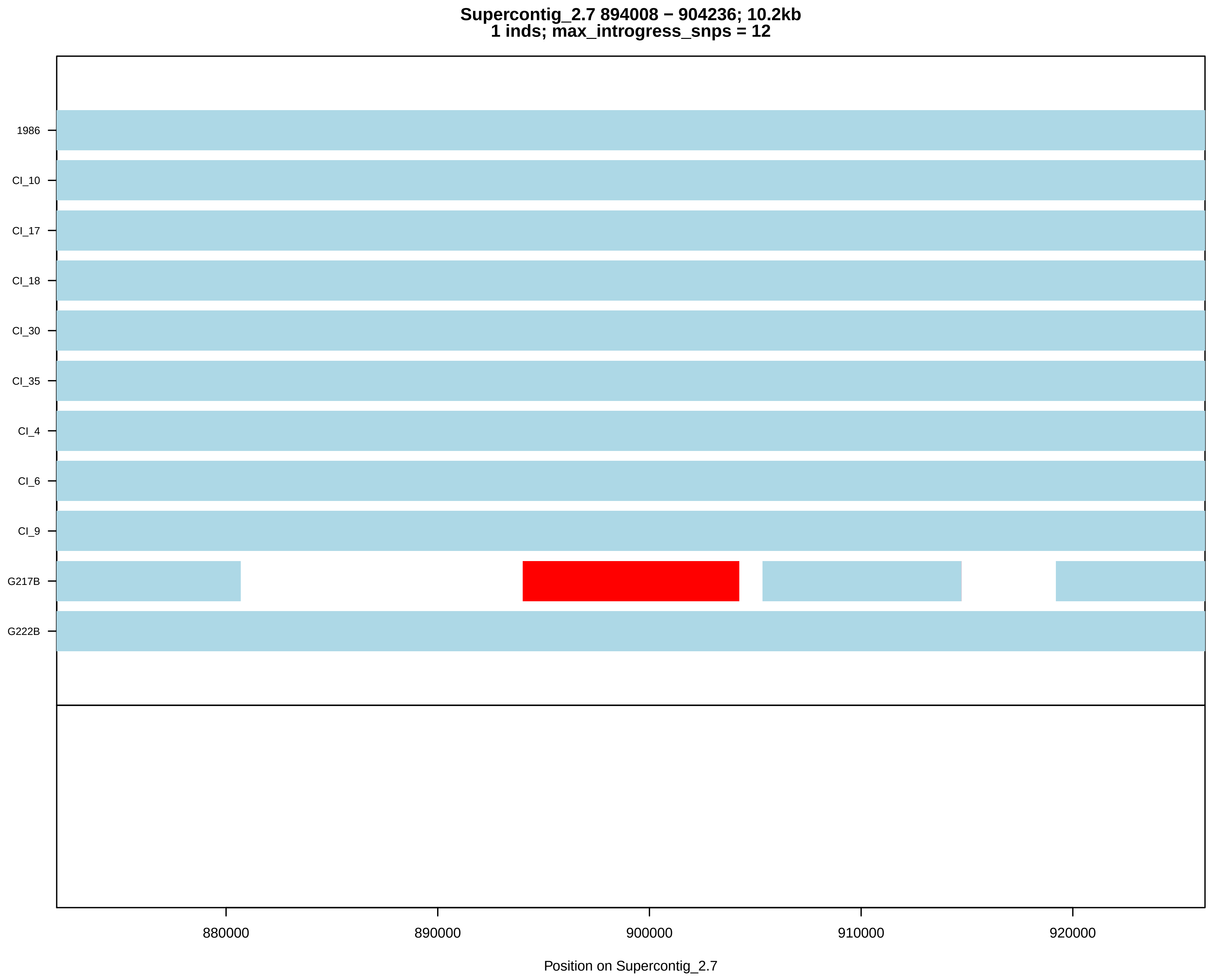

Supercontig_2.7 894008 − 904236; 10.2kb
1 inds; max_introgress_snps = 12
1986
CI_10
CI_17
CI_18
CI_30
CI_35
CI_4
CI_6
CI_9
G217B
G222B
880000
890000
900000
910000
920000
Position on Supercontig_2.7

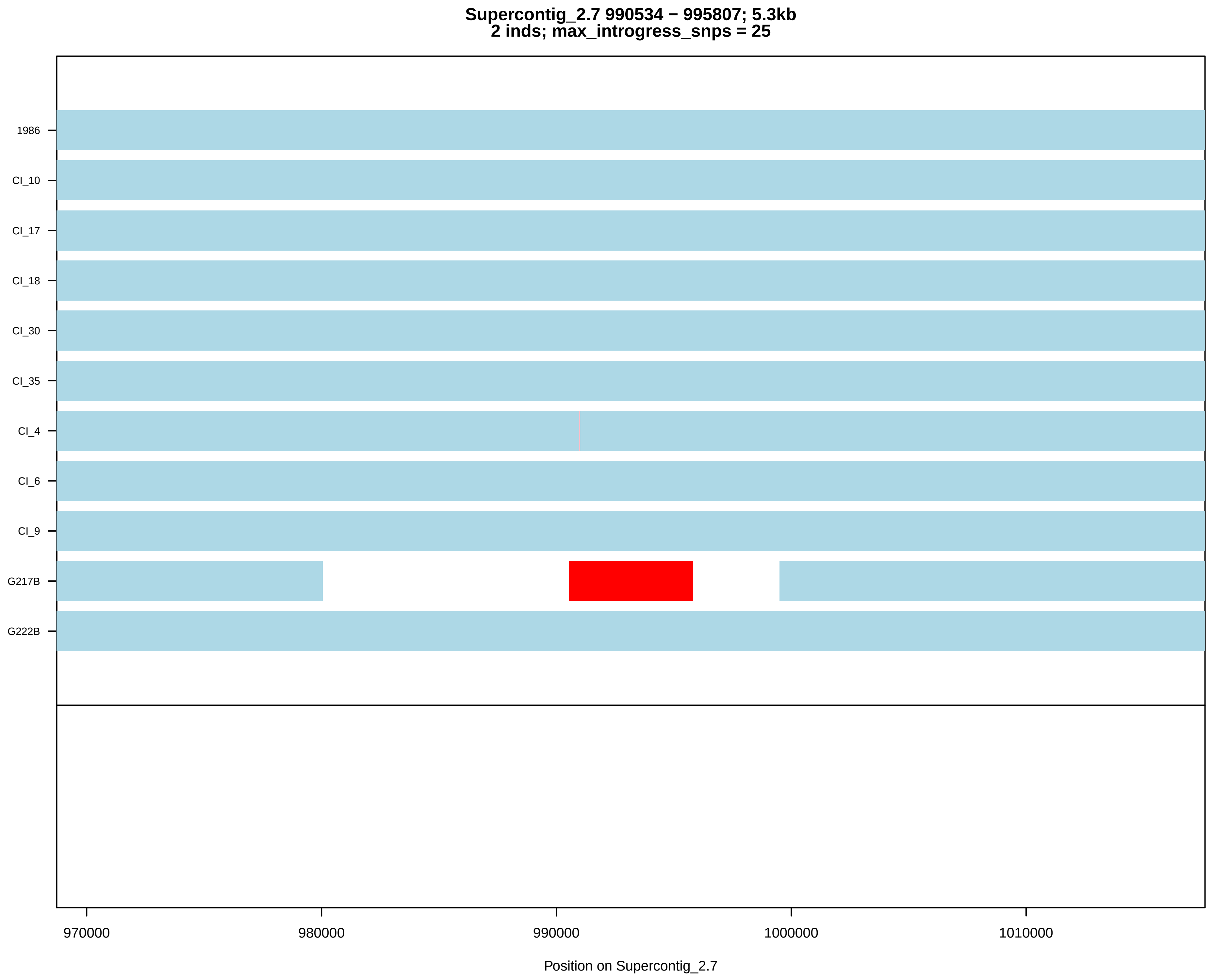
Supercontig_2.7 990534 − 995807; 5.3kb
2 inds; max_introgress_snps = 25
1986
CI_10
CI_17
CI_18
CI_30
CI_35
CI_4
CI_6
CI_9
G217B
G222B
970000
980000
990000
1000000
1010000
Position on Supercontig_2.7

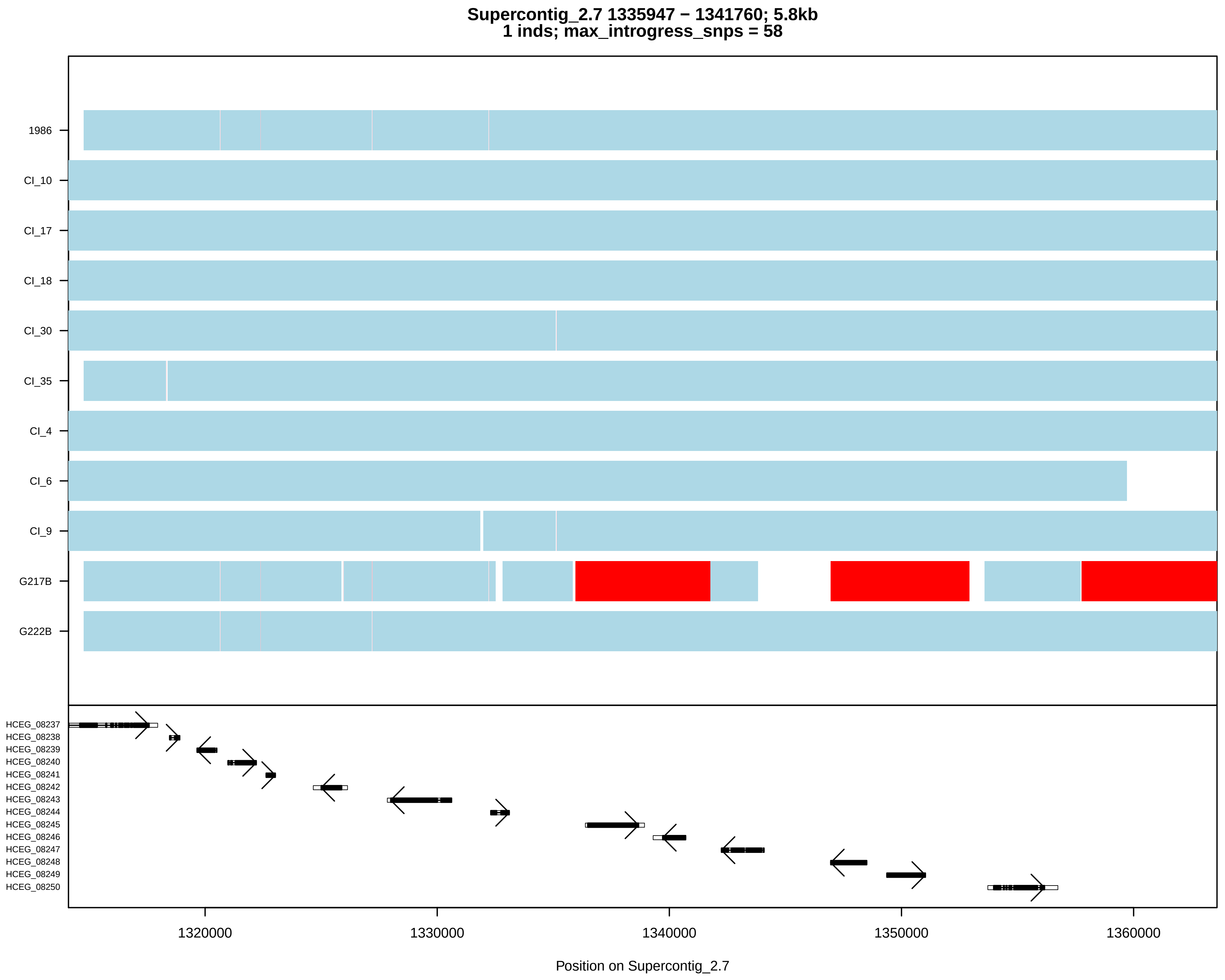
Supercontig_2.7 1335947 − 1341760; 5.8kb
1 inds; max_introgress_snps = 58
1986
CI_10
CI_17
CI_18
CI_30
CI_35
CI_4
CI_6
CI_9
G217B
G222B
HCEG_08237
HCEG_08238
HCEG_08239
HCEG_08240
HCEG_08241
HCEG_08242
HCEG_08243
HCEG_08244
HCEG_08245
HCEG_08246
HCEG_08247
HCEG_08248
HCEG_08249
HCEG_08250
1320000
1330000
1340000
1350000
1360000
Position on Supercontig_2.7

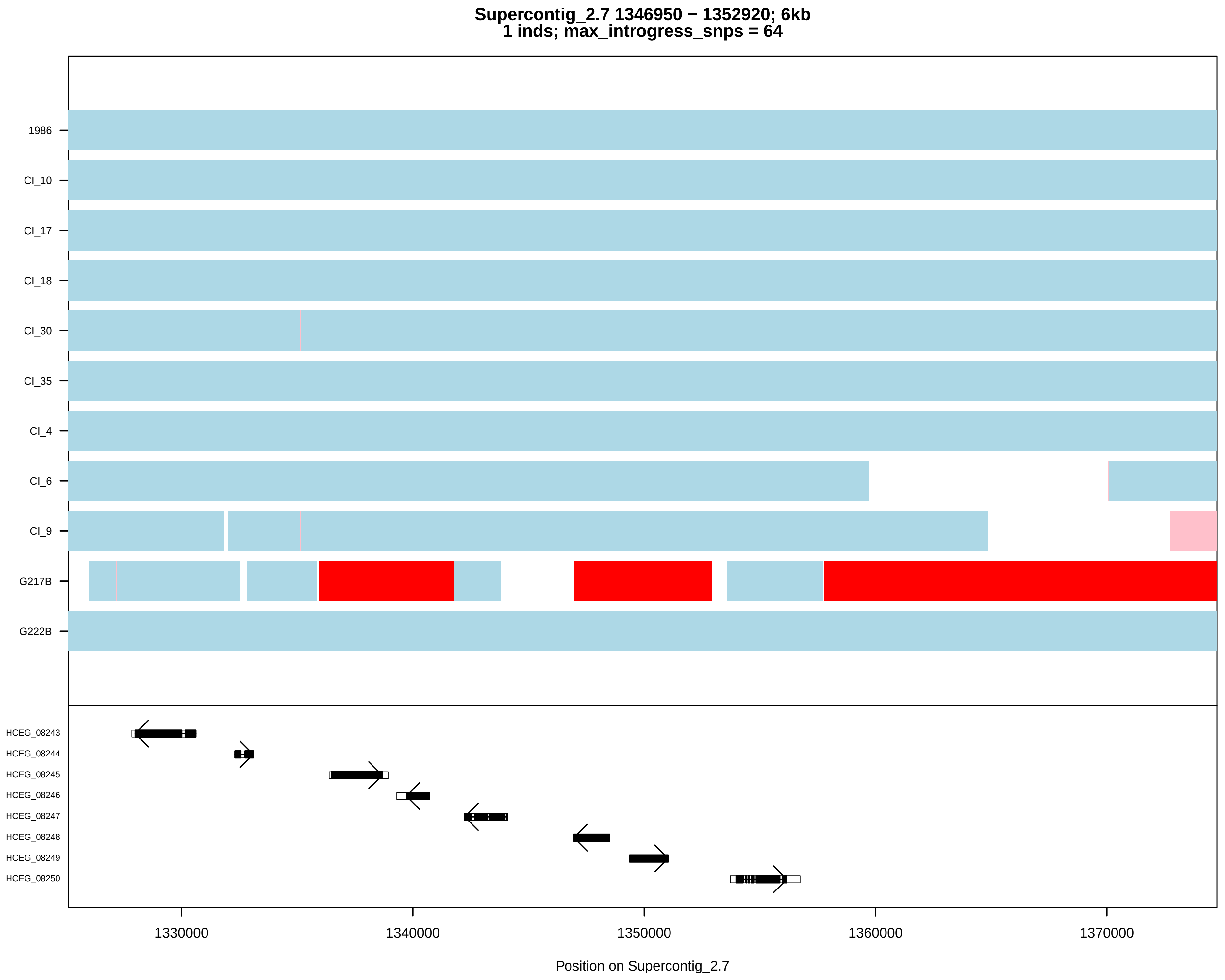

Supercontig_2.7 1346950 − 1352920; 6kb
1 inds; max_introgress_snps = 64
1986
CI_10
CI_17
CI_18
CI_30
CI_35
CI_4
CI_6
CI_9
G217B
G222B
HCEG_08243
HCEG_08244
HCEG_08245
HCEG_08246
HCEG_08247
HCEG_08248
HCEG_08249
HCEG_08250
1330000
1340000
1350000
1360000
1370000
Position on Supercontig_2.7

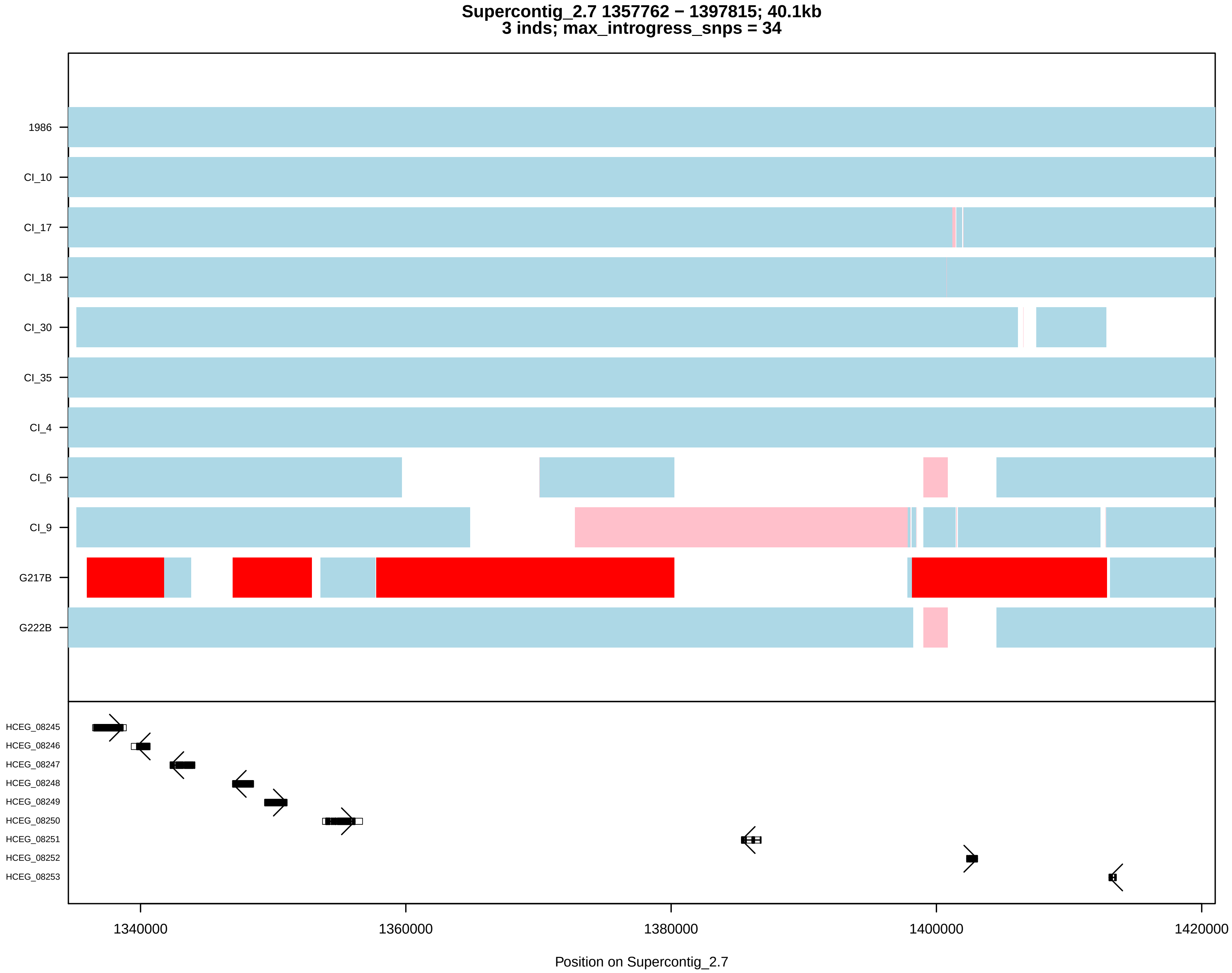

Supercontig_2.7 1357762 − 1397815; 40.1kb
3 inds; max_introgress_snps = 34
1986
CI_10
CI_17
CI_18
CI_30
CI_35
CI_4
CI_6
CI_9
G217B
G222B
HCEG_08245
HCEG_08246
HCEG_08247
HCEG_08248
HCEG_08249
HCEG_08250
HCEG_08251
HCEG_08252
HCEG_08253
1340000
1360000
1380000
1400000
1420000
Position on Supercontig_2.7

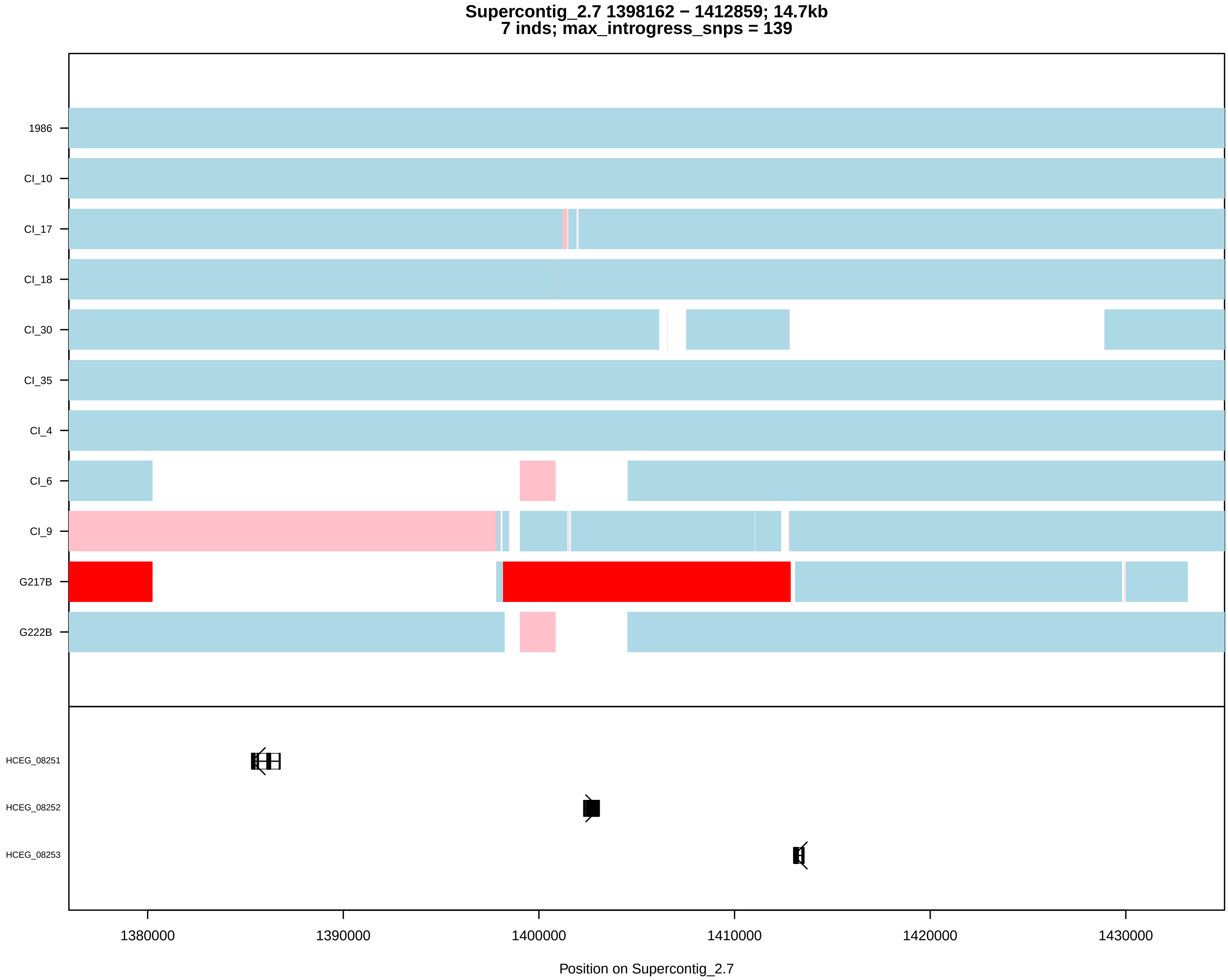

Supercontig_2.7 1398162 − 1412859; 14.7kb
7 inds; max_introgress_snps = 139
1986
CI_10
CI_17
CI_18
CI_30
CI_35
CI_4
CI_6
CI_9
G217B
G222B
HCEG_08251
HCEG_08252
HCEG_08253
1380000
1390000
1400000
1410000
1420000
1430000
Position on Supercontig_2.7

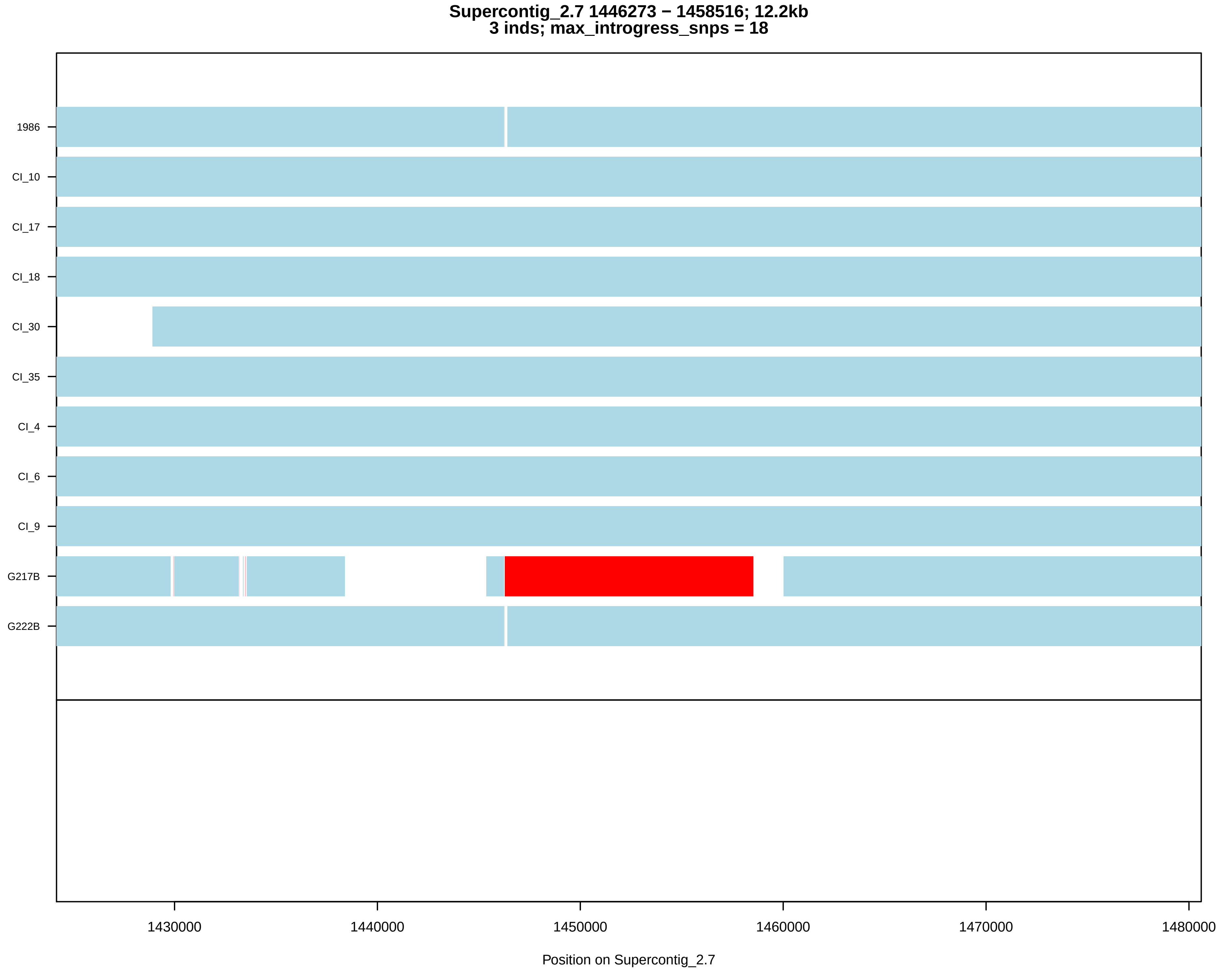
Supercontig_2.7 1446273 − 1458516; 12.2kb
3 inds; max_introgress_snps = 18
1986
CI_10
CI_17
CI_18
CI_30
CI_35
CI_4
CI_6
CI_9
G217B
G222B
1430000
1440000
1450000
1460000
1470000
1480000
Position on Supercontig_2.7

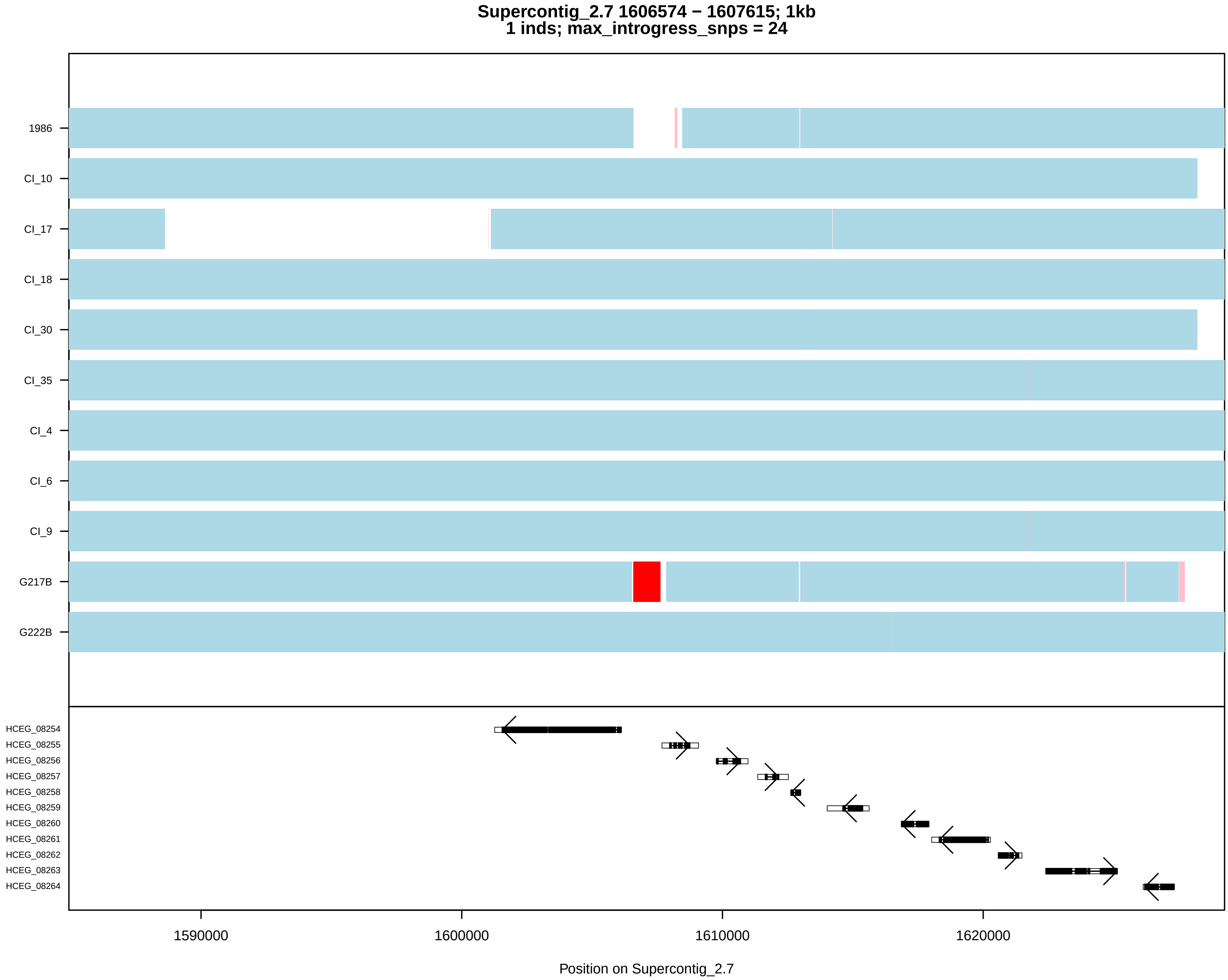

Supercontig_2.7 1606574 − 1607615; 1kb
1 inds; max_introgress_snps = 24
1986
CI_10
CI_17
CI_18
CI_30
CI_35
CI_4
CI_6
CI_9
G217B
G222B
HCEG_08254
HCEG_08255
HCEG_08256
HCEG_08257
HCEG_08258
HCEG_08259
HCEG_08260
HCEG_08261
HCEG_08262
HCEG_08263
HCEG_08264
1590000
1600000
1610000
1620000
Position on Supercontig_2.7

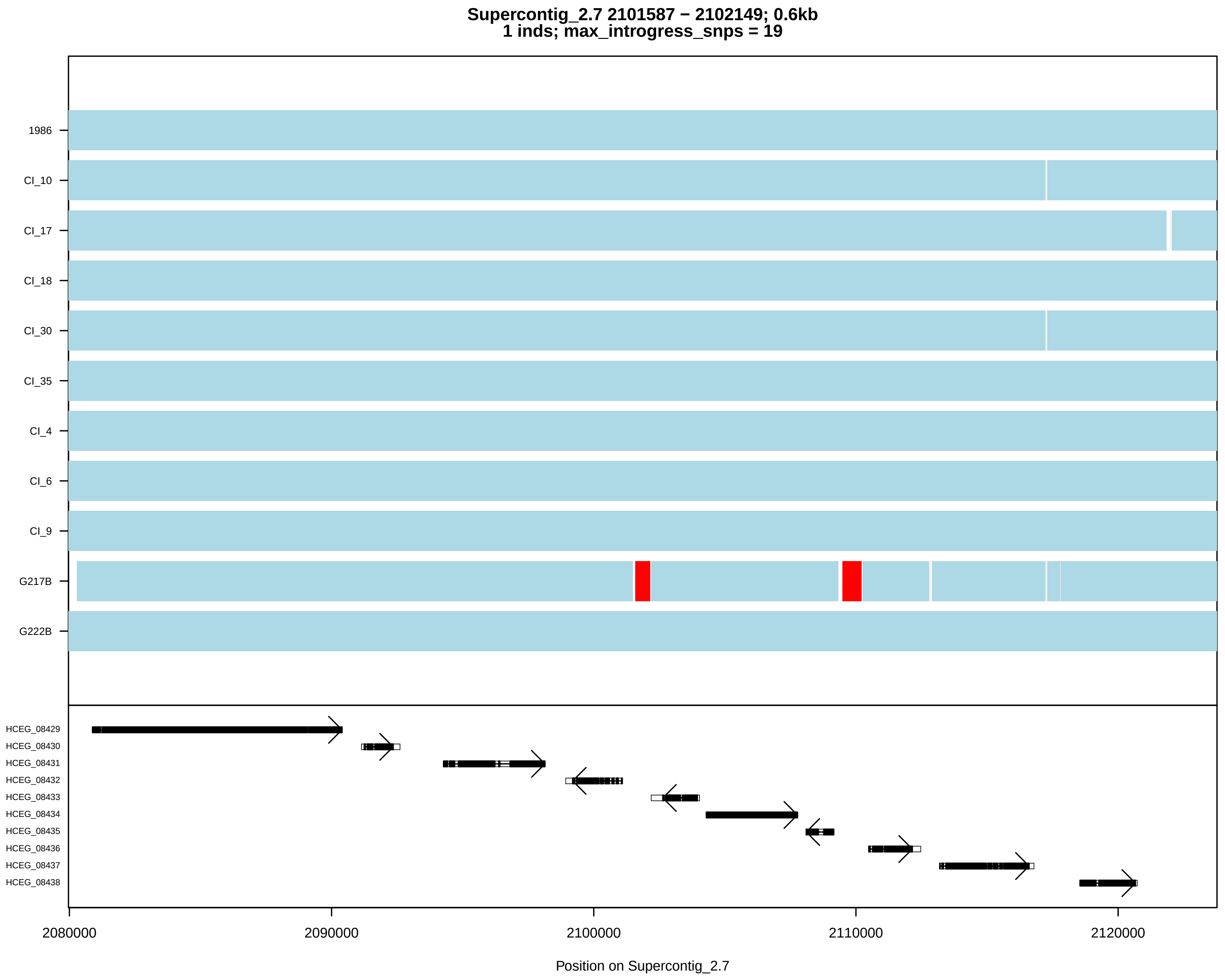
Supercontig_2.7 2101587 − 2102149; 0.6kb
1 inds; max_introgress_snps = 19
1986
CI_10
CI_17
CI_18
CI_30
CI_35
CI_4
CI_6
CI_9
G217B
G222B
HCEG_08429
HCEG_08430
HCEG_08431
HCEG_08432
HCEG_08433
HCEG_08434
HCEG_08435
HCEG_08436
HCEG_08437
HCEG_08438
2080000
2090000
2100000
2110000
2120000
Position on Supercontig_2.7

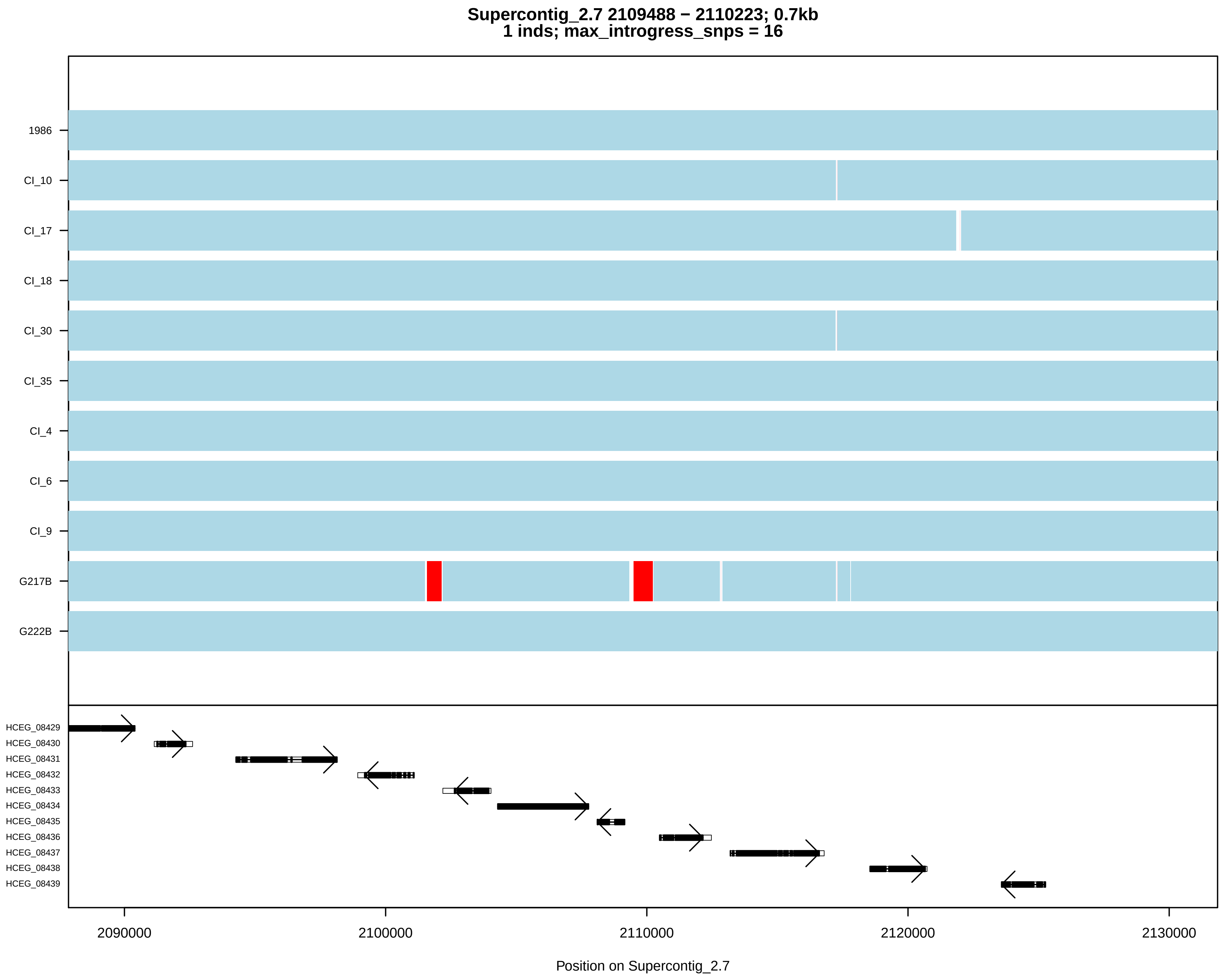

Supercontig_2.7 2109488 − 2110223; 0.7kb
1 inds; max_introgress_snps = 16
1986
CI_10
CI_17
CI_18
CI_30
CI_35
CI_4
CI_6
CI_9
G217B
G222B
HCEG_08429
HCEG_08430
HCEG_08431
HCEG_08432
HCEG_08433
HCEG_08434
HCEG_08435
HCEG_08436
HCEG_08437
HCEG_08438
HCEG_08439
2090000
2100000
2110000
2120000
2130000
Position on Supercontig_2.7

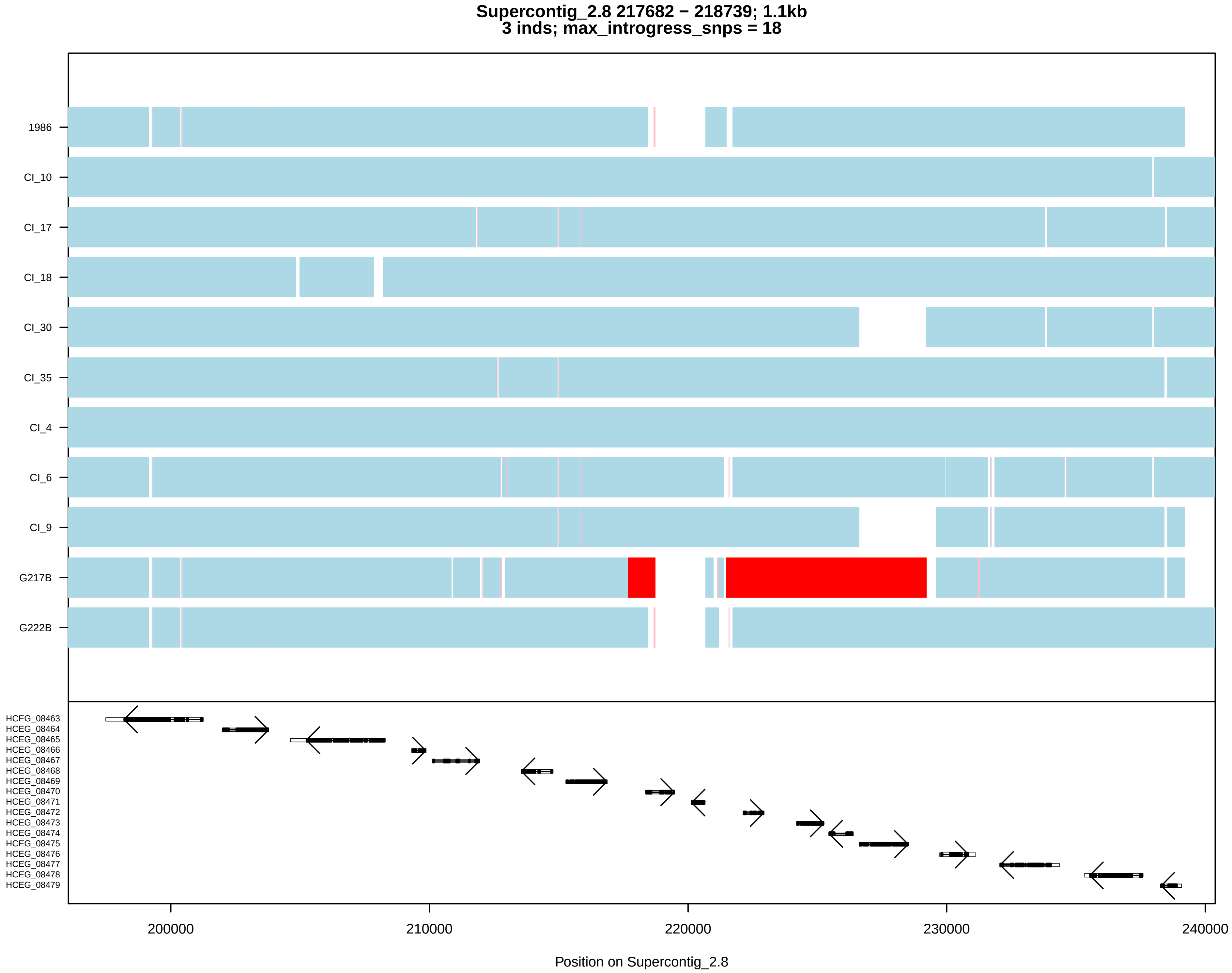

Supercontig_2.8 217682 − 218739; 1.1kb
3 inds; max_introgress_snps = 18
1986
CI_10
CI_17
CI_18
CI_30
CI_35
CI_4
CI_6
CI_9
G217B
G222B
HCEG_08463
HCEG_08464
HCEG_08465
HCEG_08466
HCEG_08467
HCEG_08468
HCEG_08469
HCEG_08470
HCEG_08471
HCEG_08472
HCEG_08473
HCEG_08474
HCEG_08475
HCEG_08476
HCEG_08477
HCEG_08478
HCEG_08479
200000
210000
220000
230000
240000
Position on Supercontig_2.8

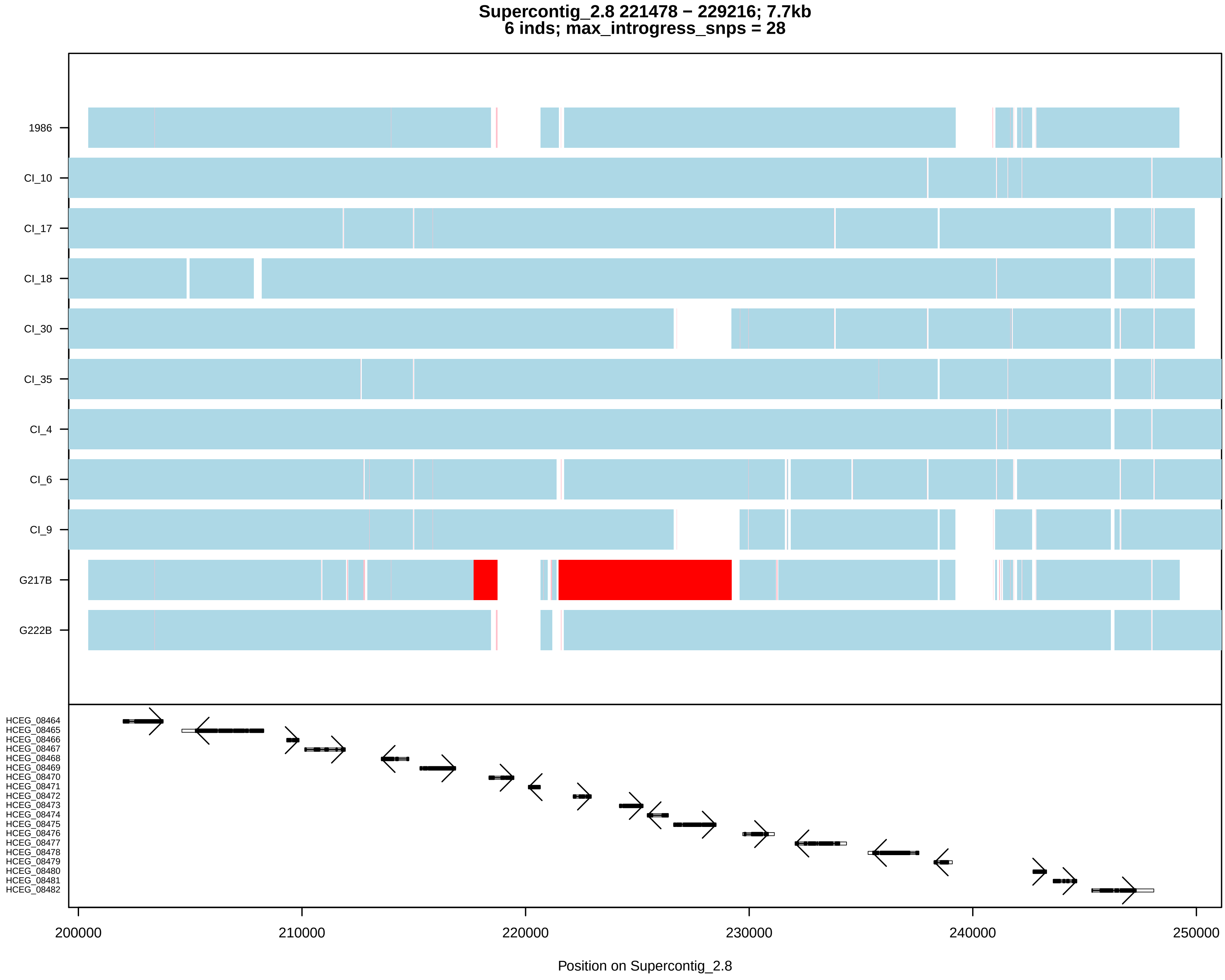

Supercontig_2.8 221478 − 229216; 7.7kb
6 inds; max_introgress_snps = 28
1986
CI_10
CI_17
CI_18
CI_30
CI_35
CI_4
CI_6
CI_9
G217B
G222B
HCEG_08464
HCEG_08465
HCEG_08466
HCEG_08467
HCEG_08468
HCEG_08469
HCEG_08470
HCEG_08471
HCEG_08472
HCEG_08473
HCEG_08474
HCEG_08475
HCEG_08476
HCEG_08477
HCEG_08478
HCEG_08479
HCEG_08480
HCEG_08481
HCEG_08482
200000
210000
220000
230000
240000
250000
Position on Supercontig_2.8

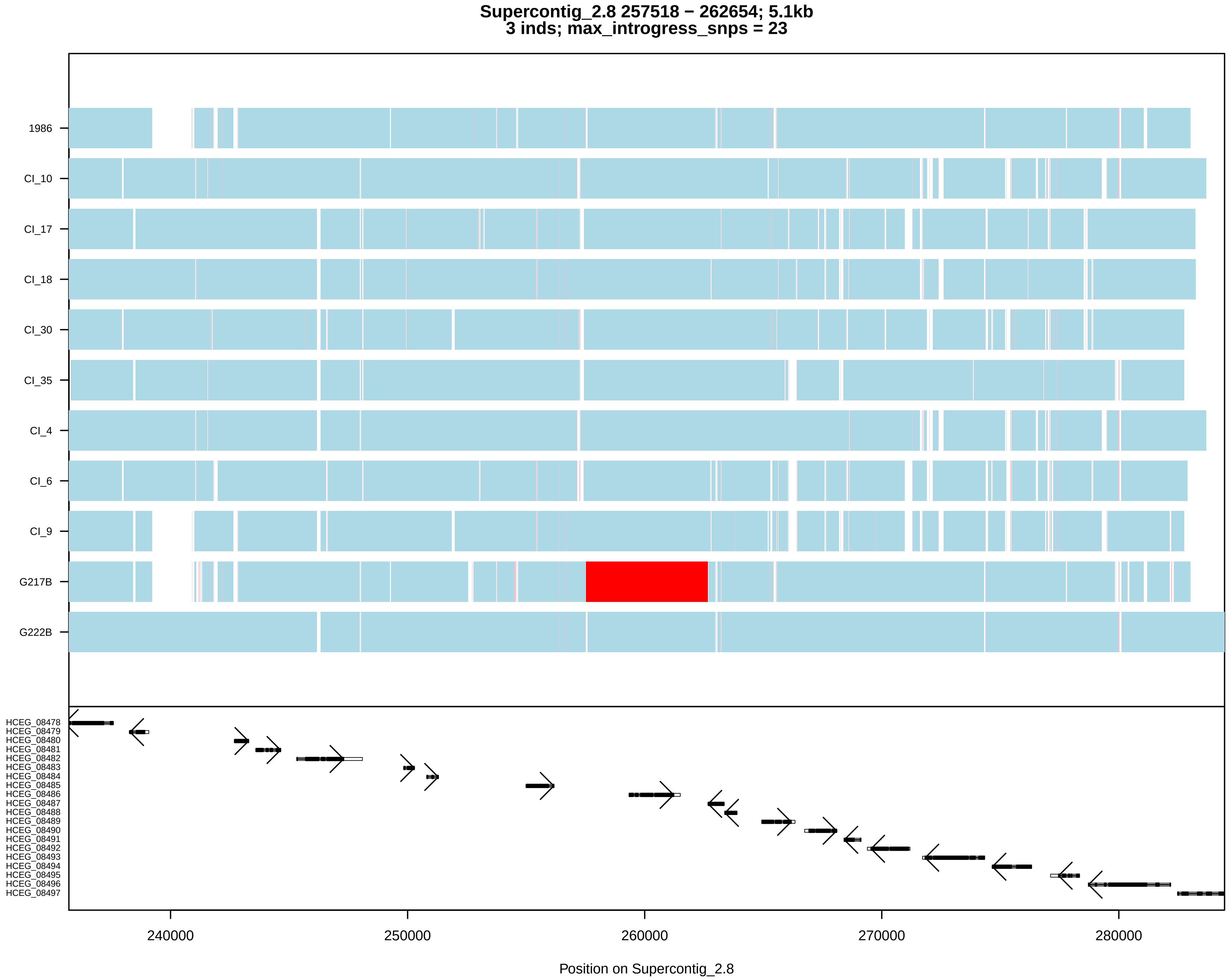
Supercontig_2.8 257518 − 262654; 5.1kb
3 inds; max_introgress_snps = 23
1986
CI_10
CI_17
CI_18
CI_30
CI_35
CI_4
CI_6
CI_9
G217B
G222B
HCEG_08478
HCEG_08479
HCEG_08480
HCEG_08481
HCEG_08482
HCEG_08483
HCEG_08484
HCEG_08485
HCEG_08486
HCEG_08487
HCEG_08488
HCEG_08489
HCEG_08490
HCEG_08491
HCEG_08492
HCEG_08493
HCEG_08494
HCEG_08495
HCEG_08496
HCEG_08497
240000
250000
260000
270000
280000
Position on Supercontig_2.8

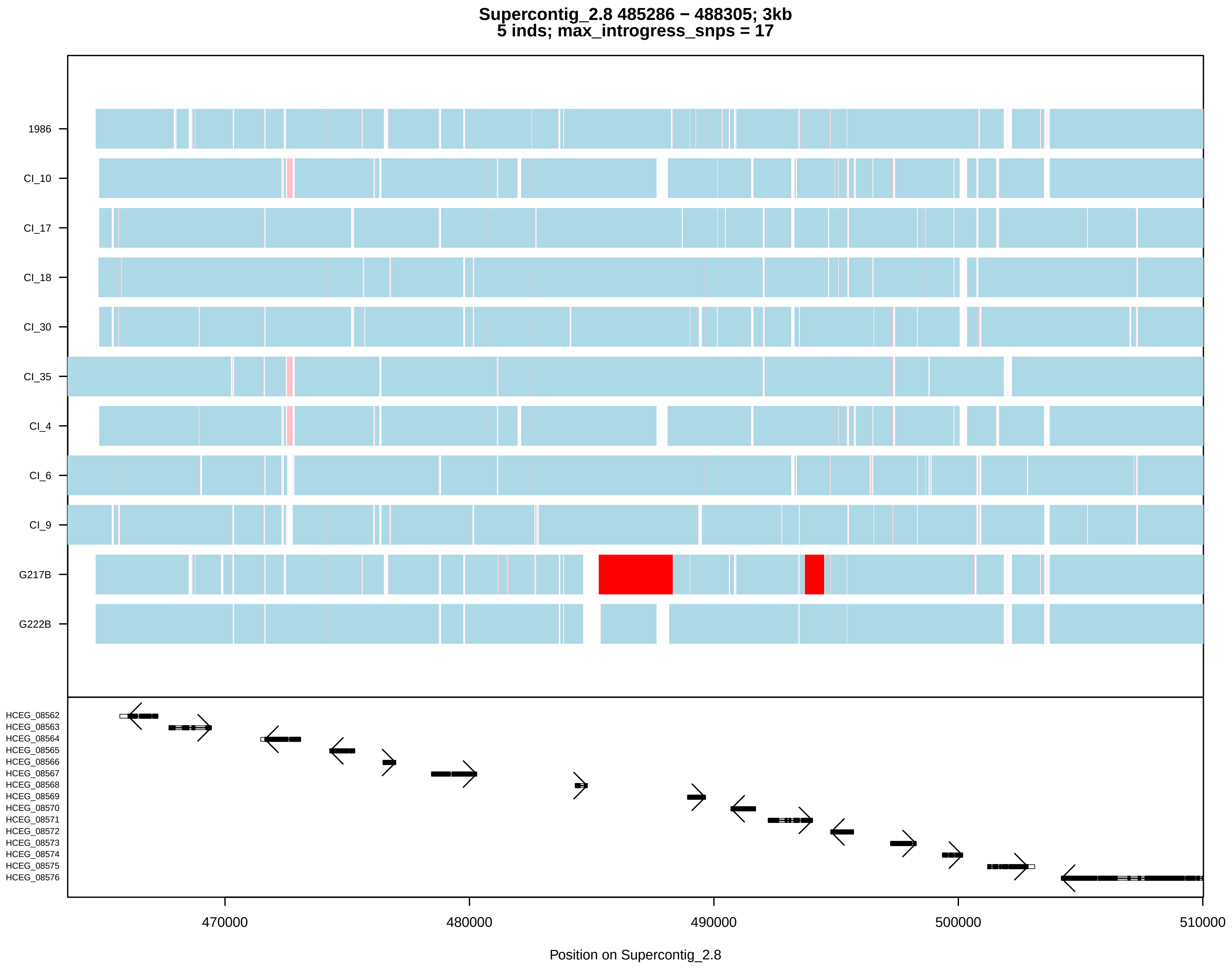
Supercontig_2.8 485286 − 488305; 3kb
5 inds; max_introgress_snps = 17
1986
CI_10
CI_17
CI_18
CI_30
CI_35
CI_4
CI_6
CI_9
G217B
G222B
HCEG_08562
HCEG_08563
HCEG_08564
HCEG_08565
HCEG_08566
HCEG_08567
HCEG_08568
HCEG_08569
HCEG_08570
HCEG_08571
HCEG_08572
HCEG_08573
HCEG_08574
HCEG_08575
HCEG_08576
470000
480000
490000
500000
510000
Position on Supercontig_2.8

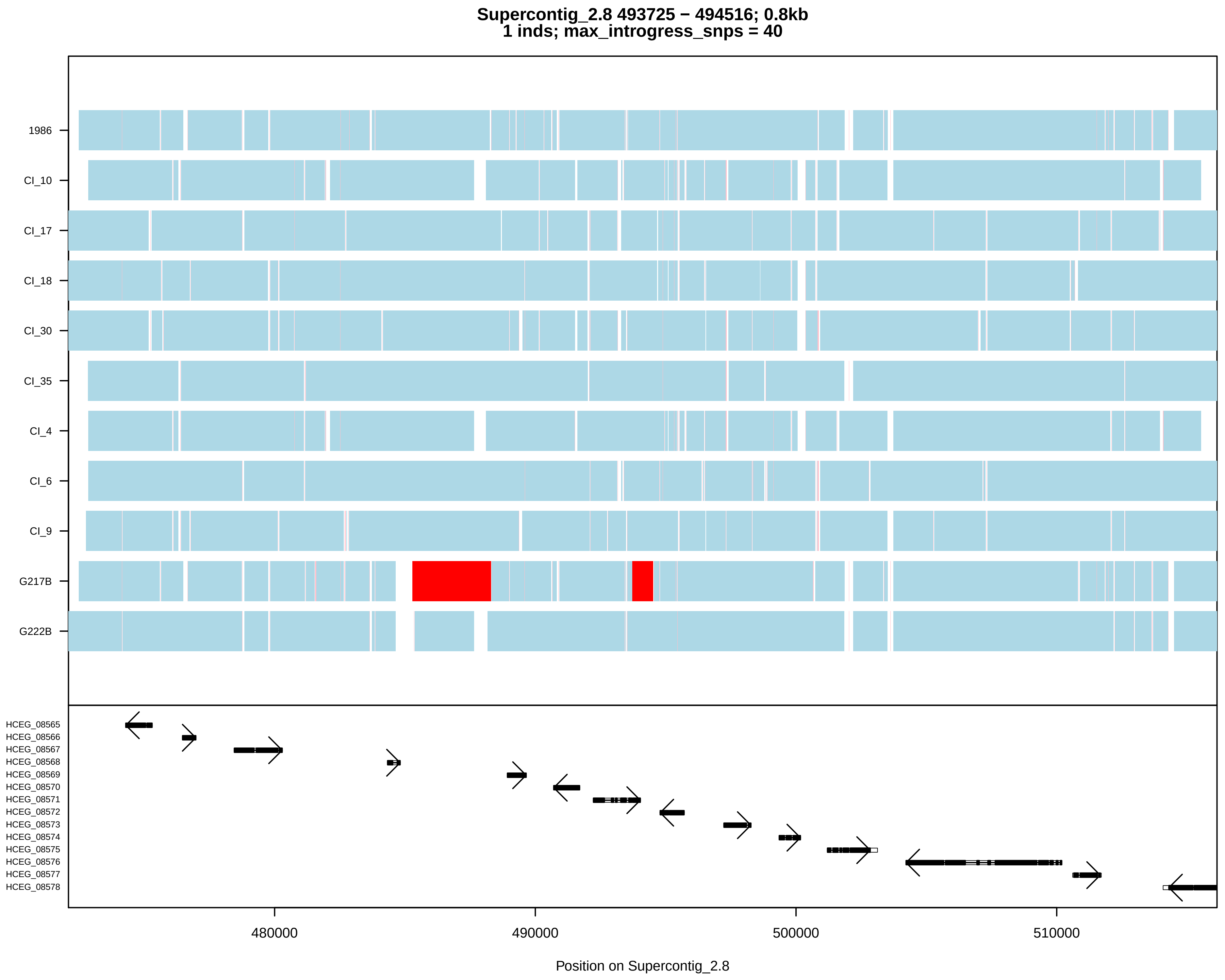

Supercontig_2.8 493725 − 494516; 0.8kb
1 inds; max_introgress_snps = 40
1986
CI_10
CI_17
CI_18
CI_30
CI_35
CI_4
CI_6
CI_9
G217B
G222B
HCEG_08565
HCEG_08566
HCEG_08567
HCEG_08568
HCEG_08569
HCEG_08570
HCEG_08571
HCEG_08572
HCEG_08573
HCEG_08574
HCEG_08575
HCEG_08576
HCEG_08577
HCEG_08578
480000
490000
500000
510000
Position on Supercontig_2.8

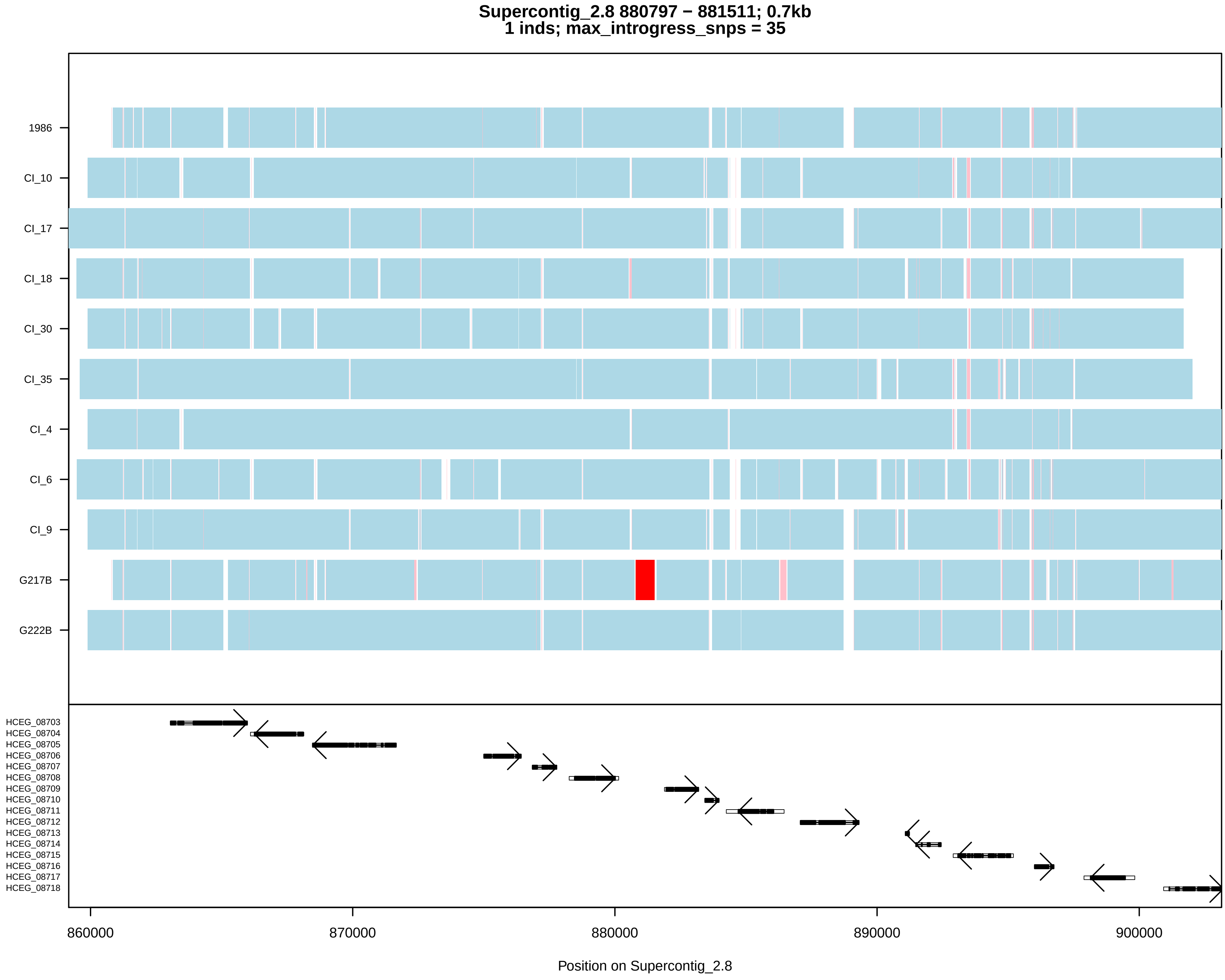
Supercontig_2.8 880797 − 881511; 0.7kb
1 inds; max_introgress_snps = 35
1986
CI_10
CI_17
CI_18
CI_30
CI_35
CI_4
CI_6
CI_9
G217B
G222B
HCEG_08703
HCEG_08704
HCEG_08705
HCEG_08706
HCEG_08707
HCEG_08708
HCEG_08709
HCEG_08710
HCEG_08711
HCEG_08712
HCEG_08713
HCEG_08714
HCEG_08715
HCEG_08716
HCEG_08717
HCEG_08718
860000
870000
880000
890000
900000
Position on Supercontig_2.8

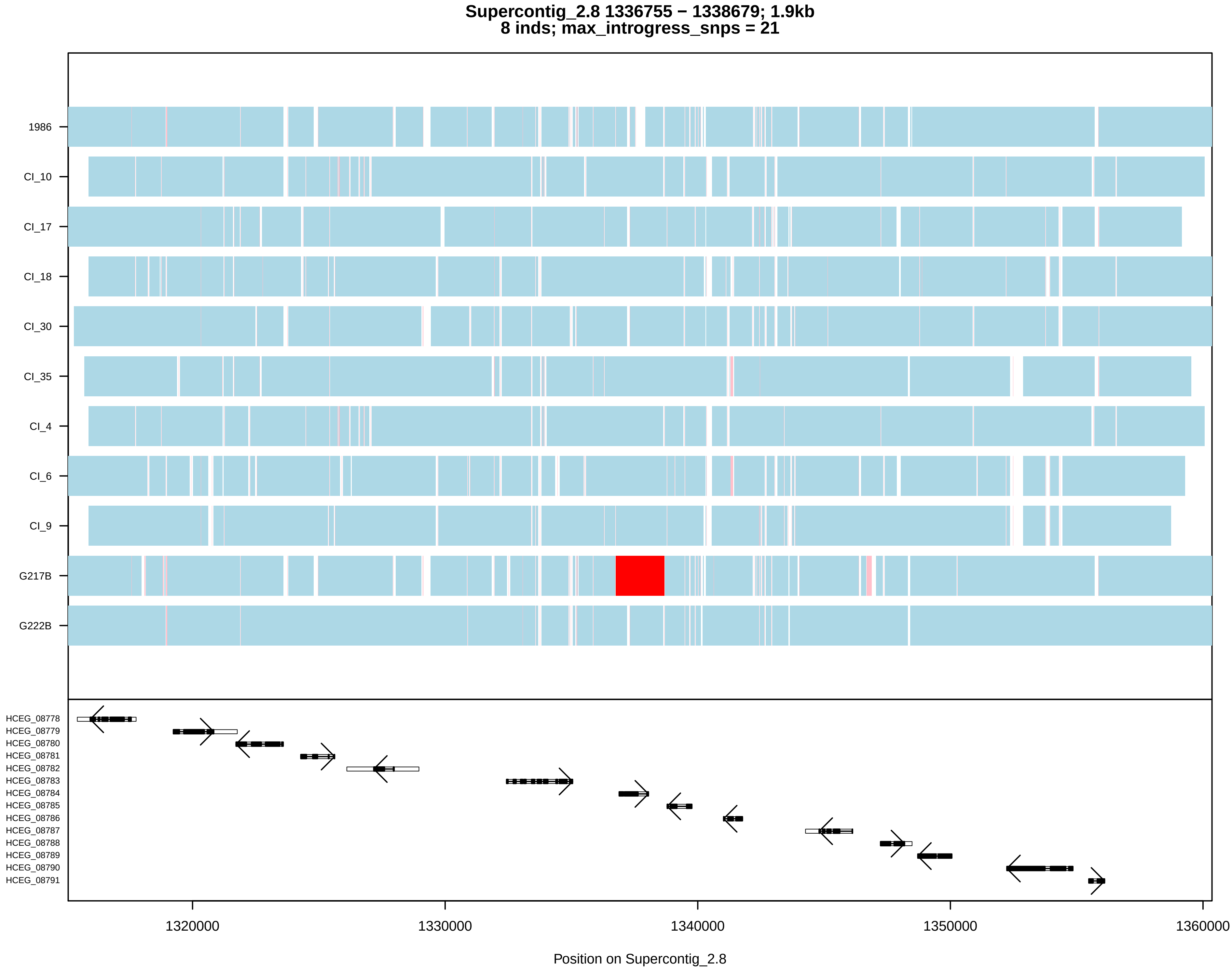

Supercontig_2.8 1336755 − 1338679; 1.9kb
8 inds; max_introgress_snps = 21
1986
CI_10
CI_17
CI_18
CI_30
CI_35
CI_4
CI_6
CI_9
G217B
G222B
HCEG_08778
HCEG_08779
HCEG_08780
HCEG_08781
HCEG_08782
HCEG_08783
HCEG_08784
HCEG_08785
HCEG_08786
HCEG_08787
HCEG_08788
HCEG_08789
HCEG_08790
HCEG_08791
1320000
1330000
1340000
1350000
1360000
Position on Supercontig_2.8

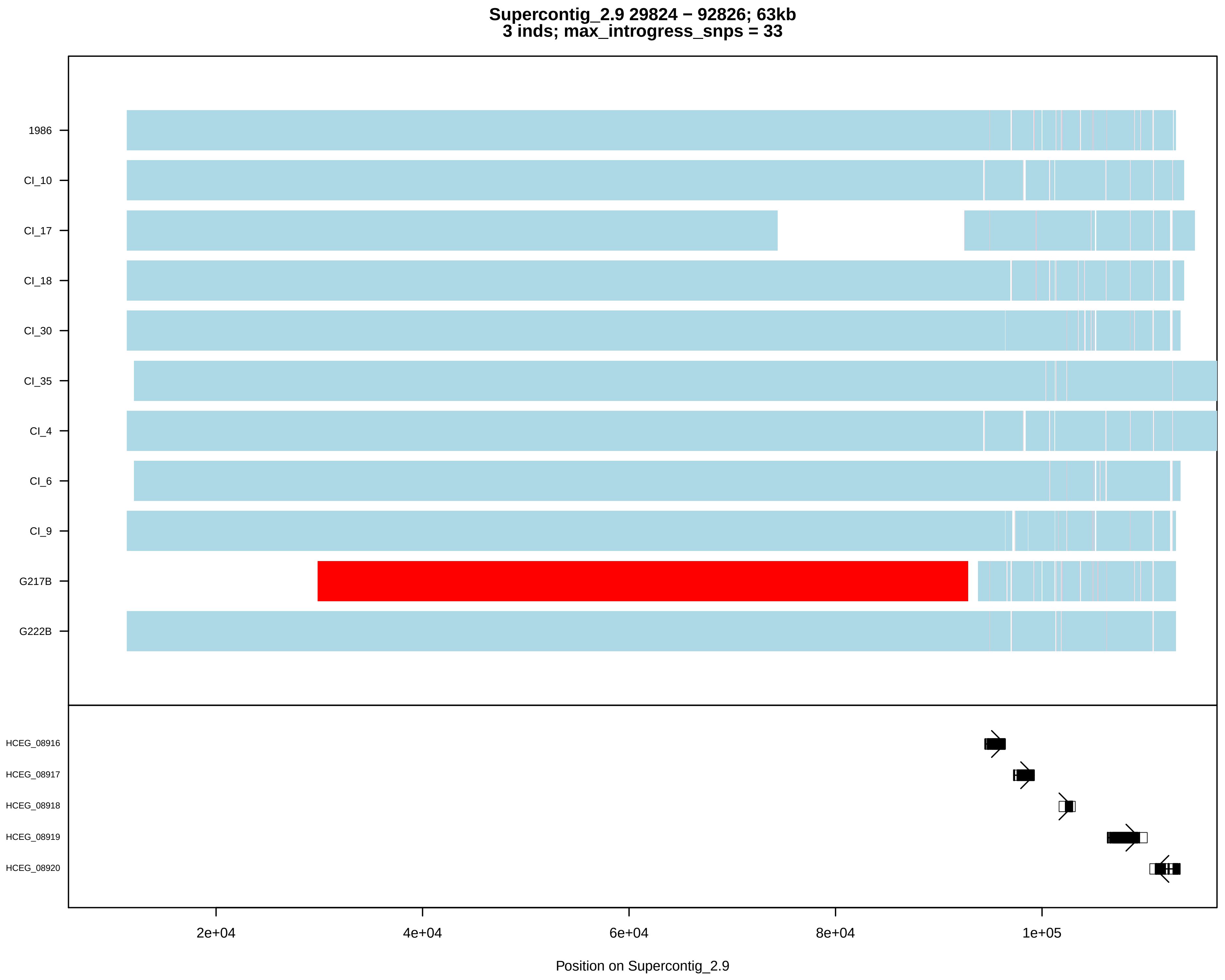

Supercontig_2.9 29824 − 92826; 63kb
3 inds; max_introgress_snps = 33
1986
CI_10
CI_17
CI_18
CI_30
CI_35
CI_4
CI_6
CI_9
G217B
G222B
HCEG_08916
HCEG_08917
HCEG_08918
HCEG_08919
HCEG_08920
2e+04
4e+04
6e+04
8e+04
1e+05
Position on Supercontig_2.9

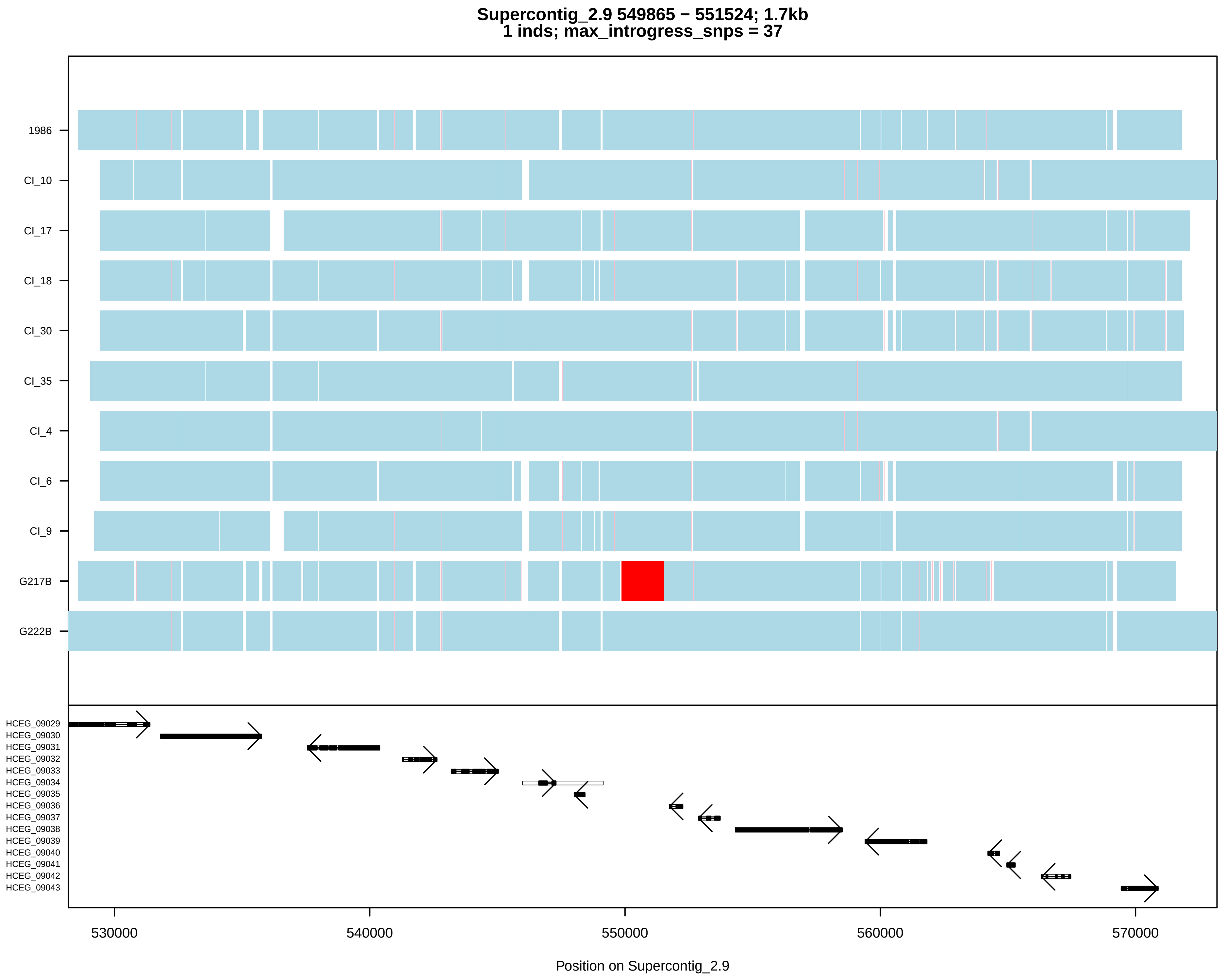
Supercontig_2.9 549865 − 551524; 1.7kb
1 inds; max_introgress_snps = 37
1986
CI_10
CI_17
CI_18
CI_30
CI_35
CI_4
CI_6
CI_9
G217B
G222B
HCEG_09029
HCEG_09030
HCEG_09031
HCEG_09032
HCEG_09033
HCEG_09034
HCEG_09035
HCEG_09036
HCEG_09037
HCEG_09038
HCEG_09039
HCEG_09040
HCEG_09041
HCEG_09042
HCEG_09043
530000
540000
550000
560000
570000
Position on Supercontig_2.9

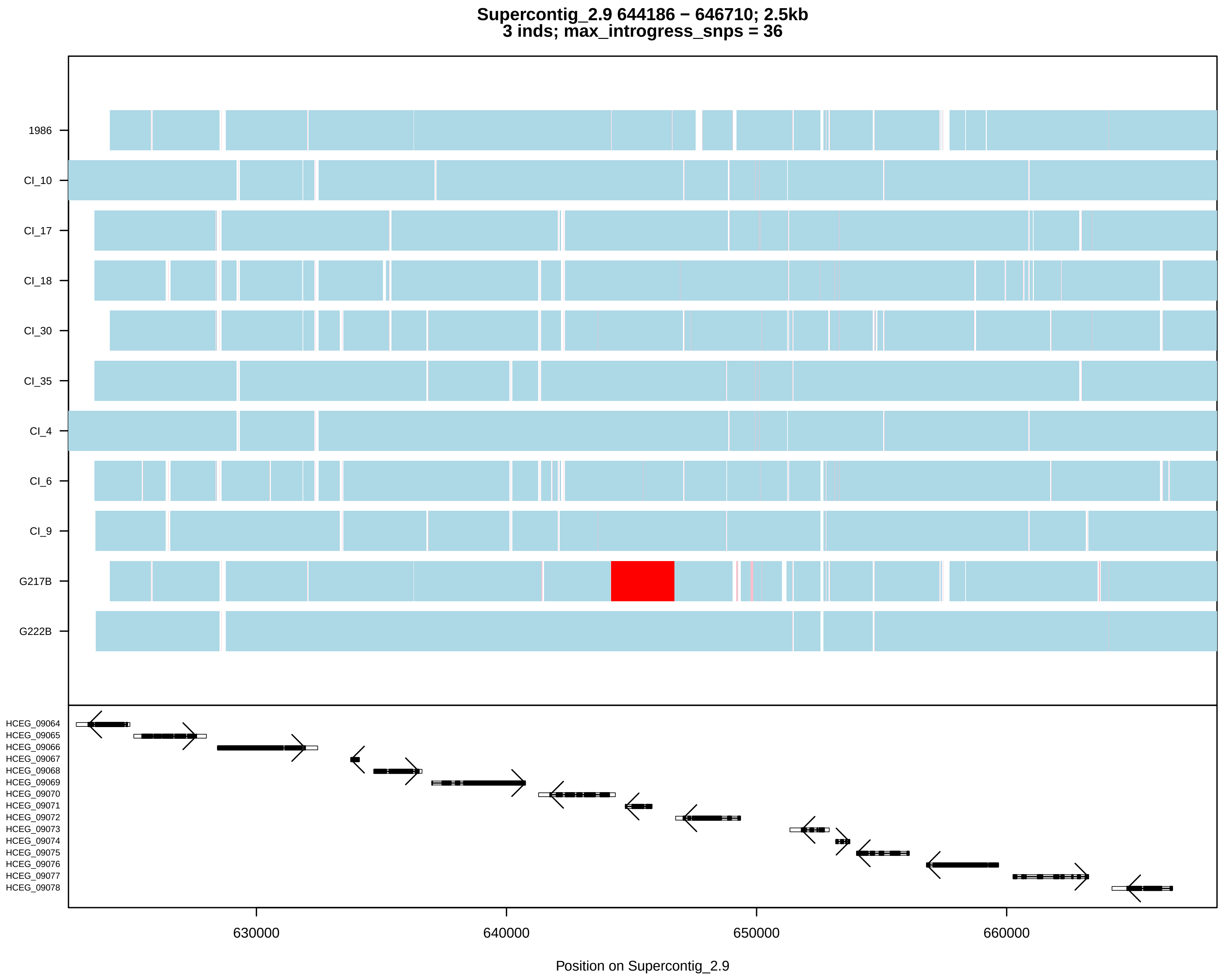

Supercontig_2.9 644186 − 646710; 2.5kb
3 inds; max_introgress_snps = 36
1986
CI_10
CI_17
CI_18
CI_30
CI_35
CI_4
CI_6
CI_9
G217B
G222B
HCEG_09064
HCEG_09065
HCEG_09066
HCEG_09067
HCEG_09068
HCEG_09069
HCEG_09070
HCEG_09071
HCEG_09072
HCEG_09073
HCEG_09074
HCEG_09075
HCEG_09076
HCEG_09077
HCEG_09078
630000
640000
650000
660000
Position on Supercontig_2.9

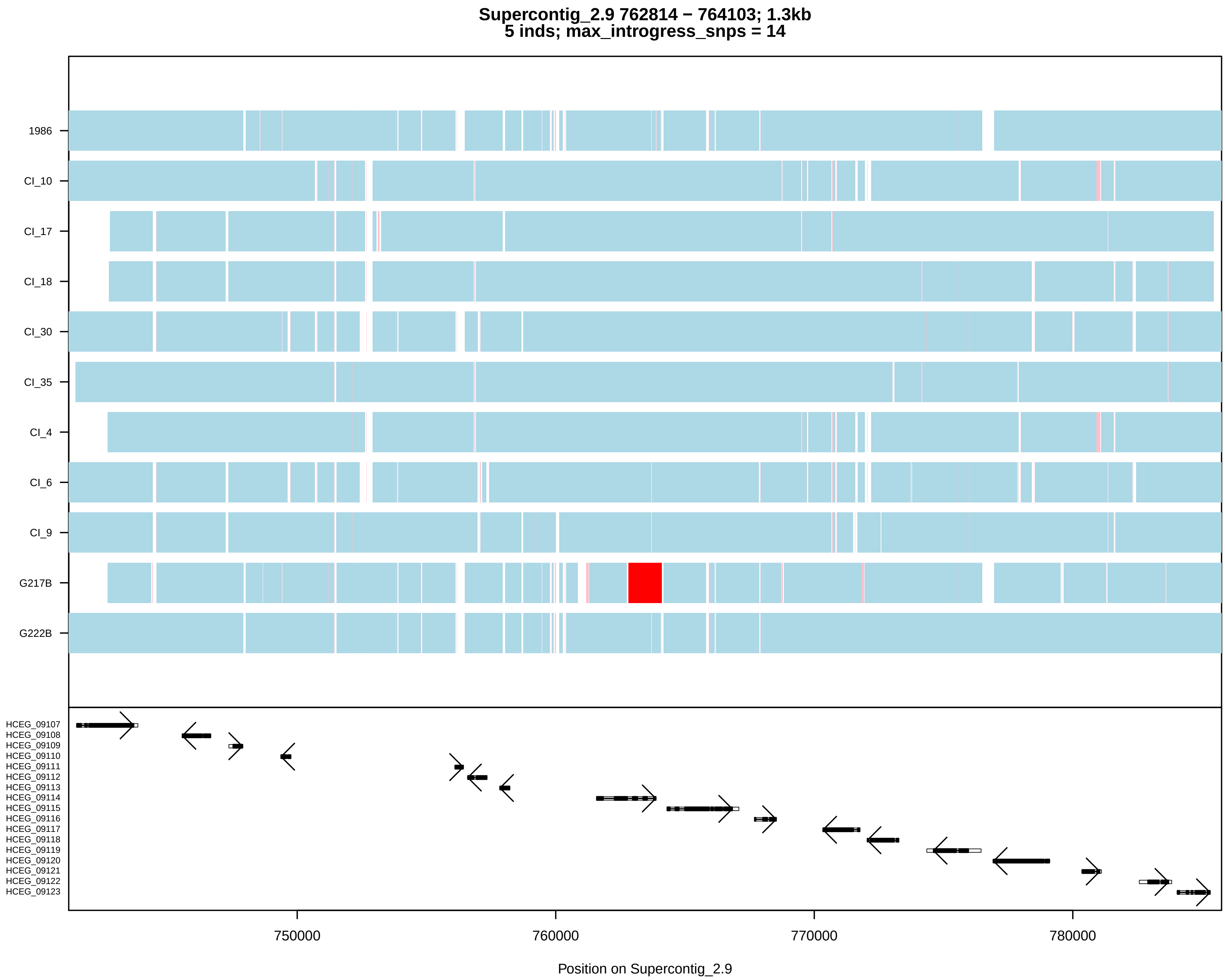
Supercontig_2.9 762814 − 764103; 1.3kb
5 inds; max_introgress_snps = 14
1986
CI_10
CI_17
CI_18
CI_30
CI_35
CI_4
CI_6
CI_9
G217B
G222B
HCEG_09107
HCEG_09108
HCEG_09109
HCEG_09110
HCEG_09111
HCEG_09112
HCEG_09113
HCEG_09114
HCEG_09115
HCEG_09116
HCEG_09117
HCEG_09118
HCEG_09119
HCEG_09120
HCEG_09121
HCEG_09122
HCEG_09123
750000
760000
770000
780000
Position on Supercontig_2.9

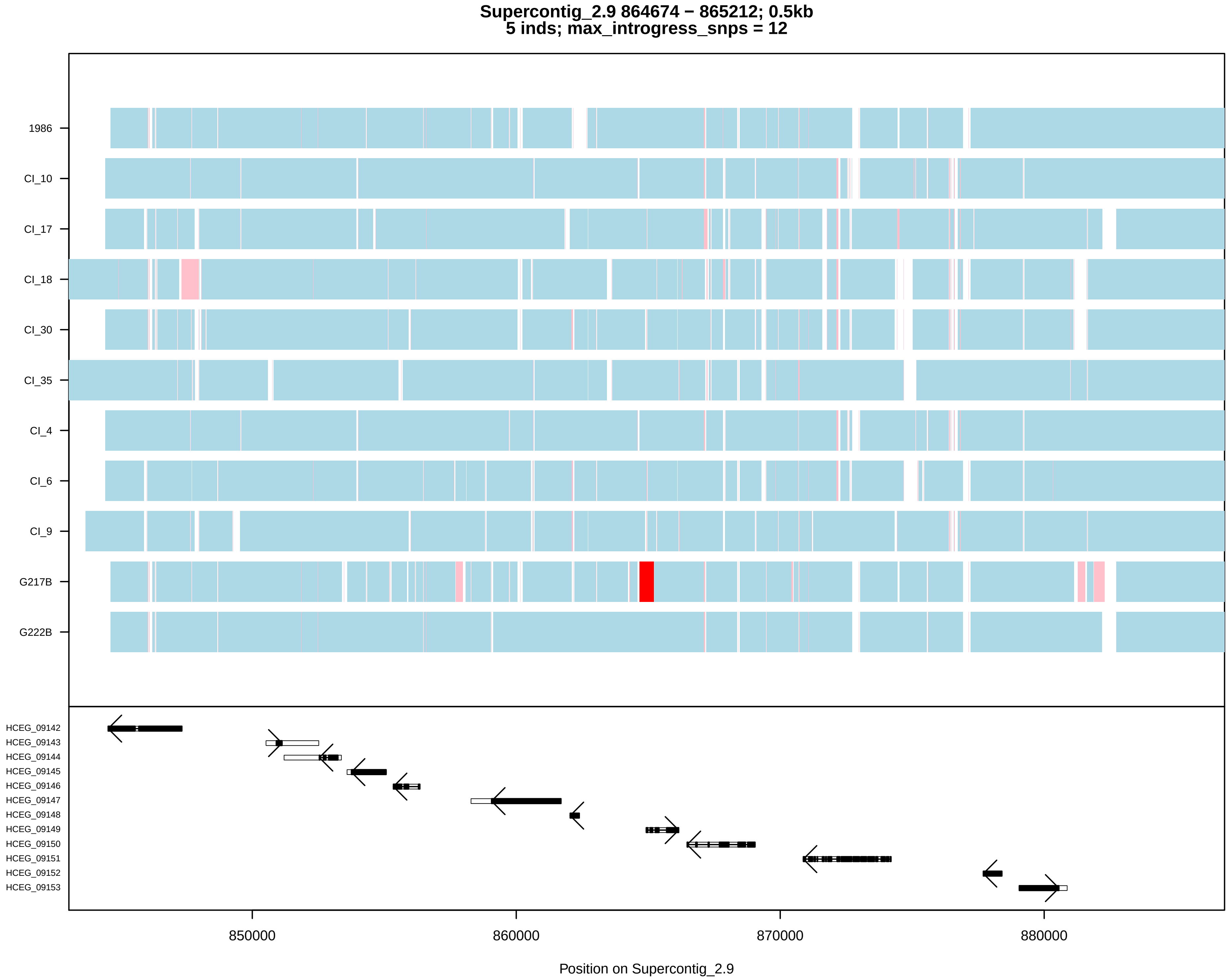

Supercontig_2.9 864674 − 865212; 0.5kb
5 inds; max_introgress_snps = 12
1986
CI_10
CI_17
CI_18
CI_30
CI_35
CI_4
CI_6
CI_9
G217B
G222B
HCEG_09142
HCEG_09143
HCEG_09144
HCEG_09145
HCEG_09146
HCEG_09147
HCEG_09148
HCEG_09149
HCEG_09150
HCEG_09151
HCEG_09152
HCEG_09153
850000
860000
870000
880000
Position on Supercontig_2.9

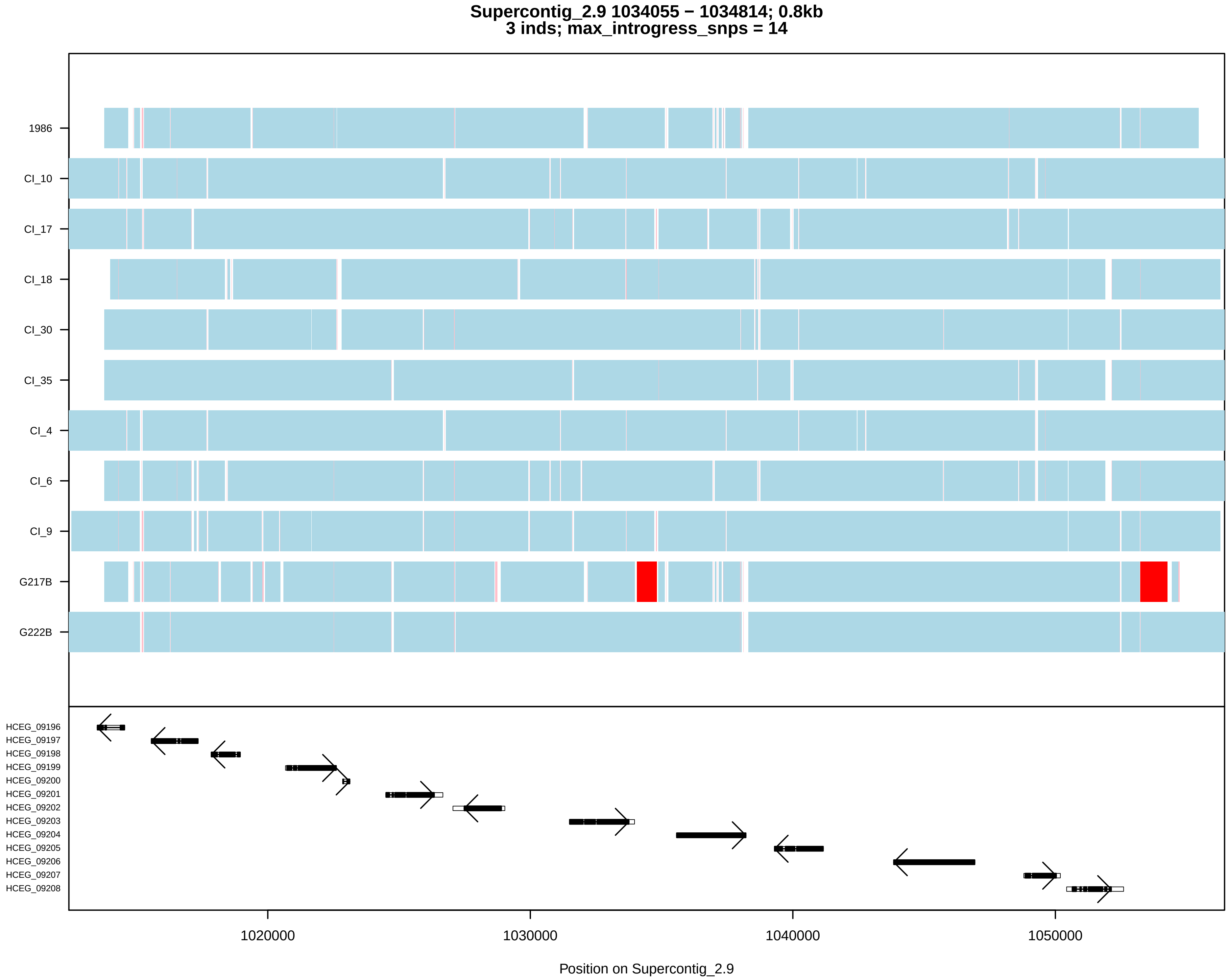
Supercontig_2.9 1034055 − 1034814; 0.8kb
3 inds; max_introgress_snps = 14
1986
CI_10
CI_17
CI_18
CI_30
CI_35
CI_4
CI_6
CI_9
G217B
G222B
HCEG_09196
HCEG_09197
HCEG_09198
HCEG_09199
HCEG_09200
HCEG_09201
HCEG_09202
HCEG_09203
HCEG_09204
HCEG_09205
HCEG_09206
HCEG_09207
HCEG_09208
1020000
1030000
1040000
1050000
Position on Supercontig_2.9

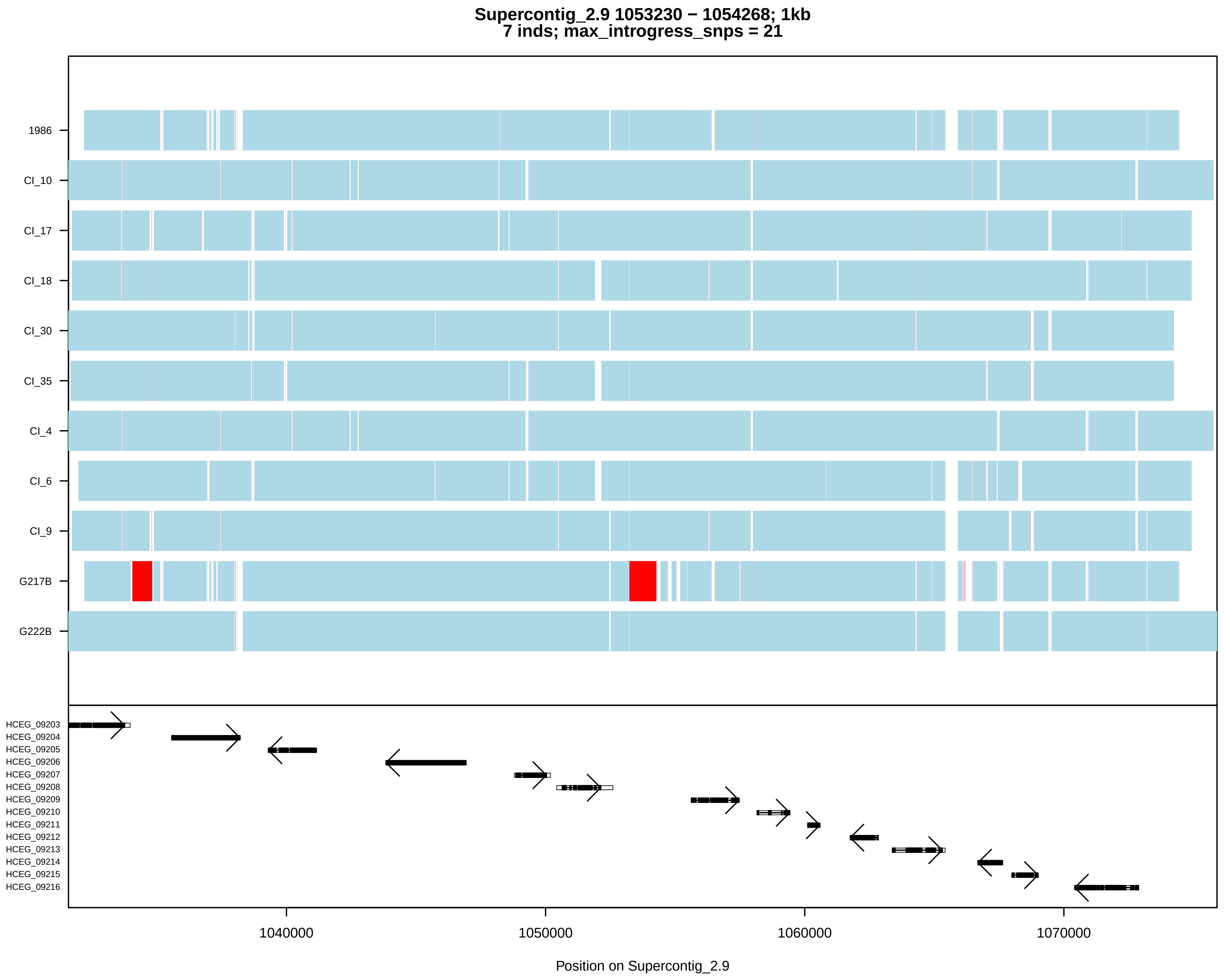

Supercontig_2.9 1053230 − 1054268; 1kb
7 inds; max_introgress_snps = 21
1986
CI_10
CI_17
CI_18
CI_30
CI_35
CI_4
CI_6
CI_9
G217B
G222B
HCEG_09203
HCEG_09204
HCEG_09205
HCEG_09206
HCEG_09207
HCEG_09208
HCEG_09209
HCEG_09210
HCEG_09211
HCEG_09212
HCEG_09213
HCEG_09214
HCEG_09215
HCEG_09216
1040000
1050000
1060000
1070000
Position on Supercontig_2.9

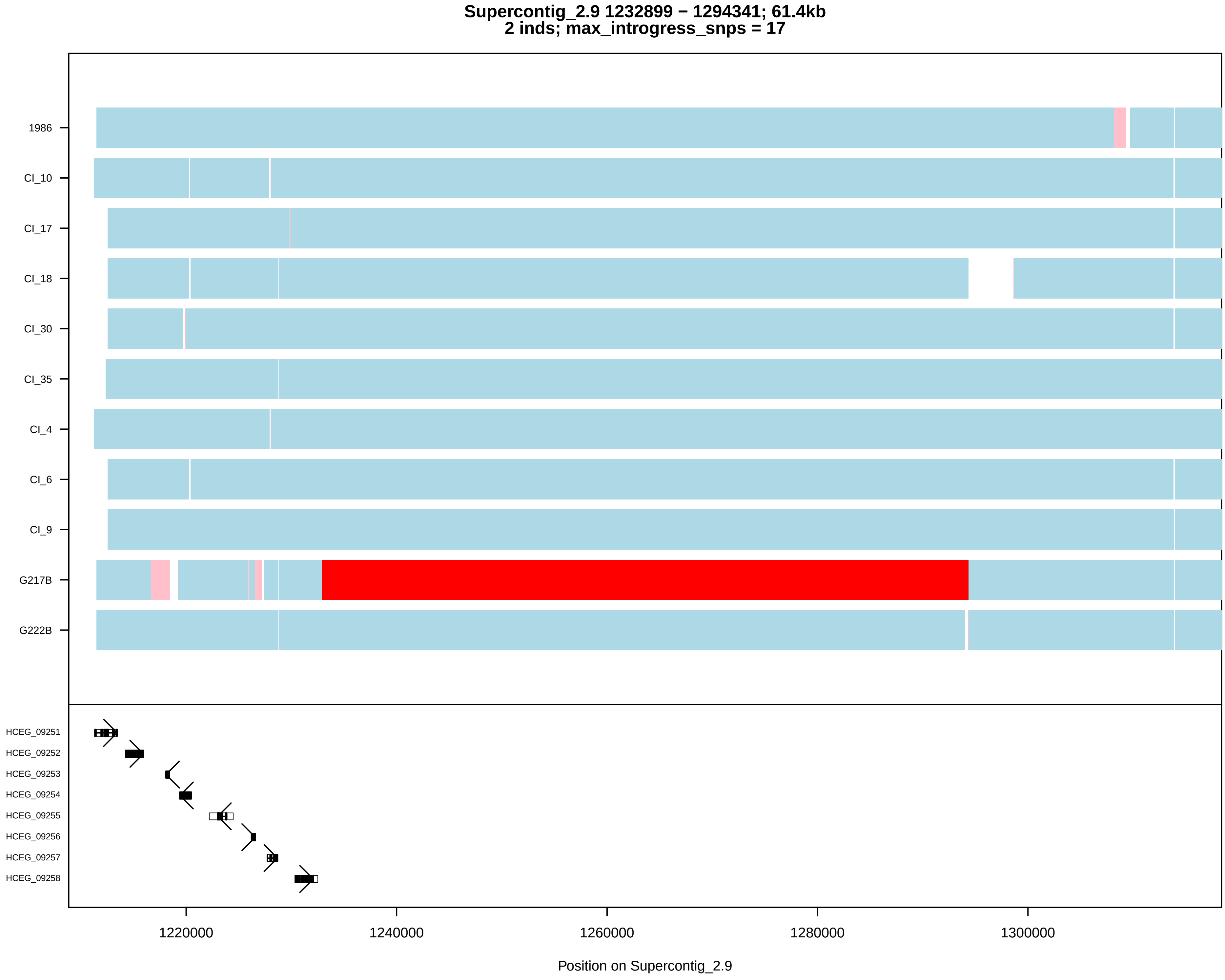
Supercontig_2.9 1232899 − 1294341; 61.4kb
2 inds; max_introgress_snps = 17
1986
CI_10
CI_17
CI_18
CI_30
CI_35
CI_4
CI_6
CI_9
G217B
G222B
HCEG_09251
HCEG_09252
HCEG_09253
HCEG_09254
HCEG_09255
HCEG_09256
HCEG_09257
HCEG_09258
1220000
1240000
1260000
1280000
1300000
Position on Supercontig_2.9

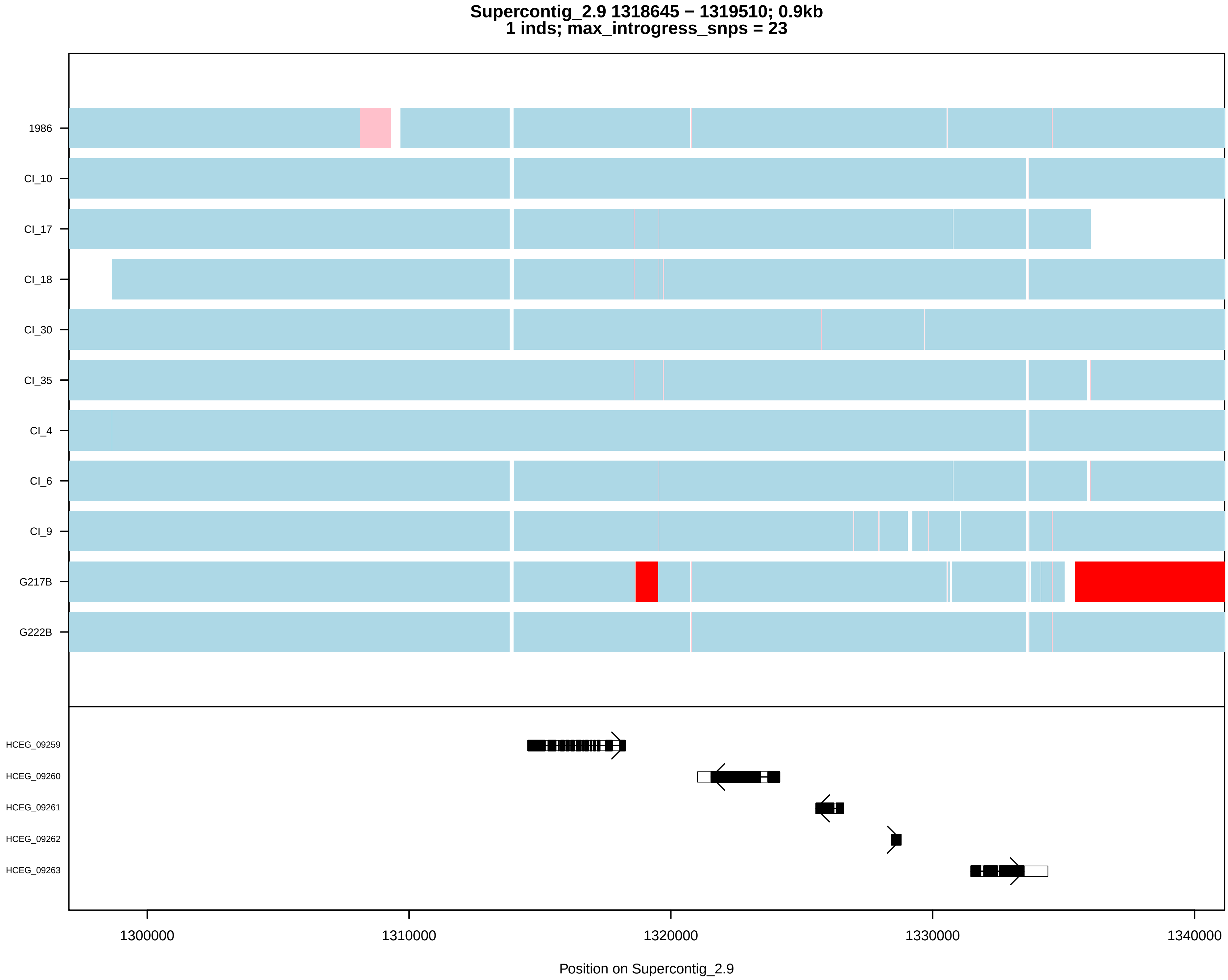

Supercontig_2.9 1318645 − 1319510; 0.9kb
1 inds; max_introgress_snps = 23
1986
CI_10
CI_17
CI_18
CI_30
CI_35
CI_4
CI_6
CI_9
G217B
G222B
HCEG_09259
HCEG_09260
HCEG_09261
HCEG_09262
HCEG_09263
1300000
1310000
1320000
1330000
1340000
Position on Supercontig_2.9

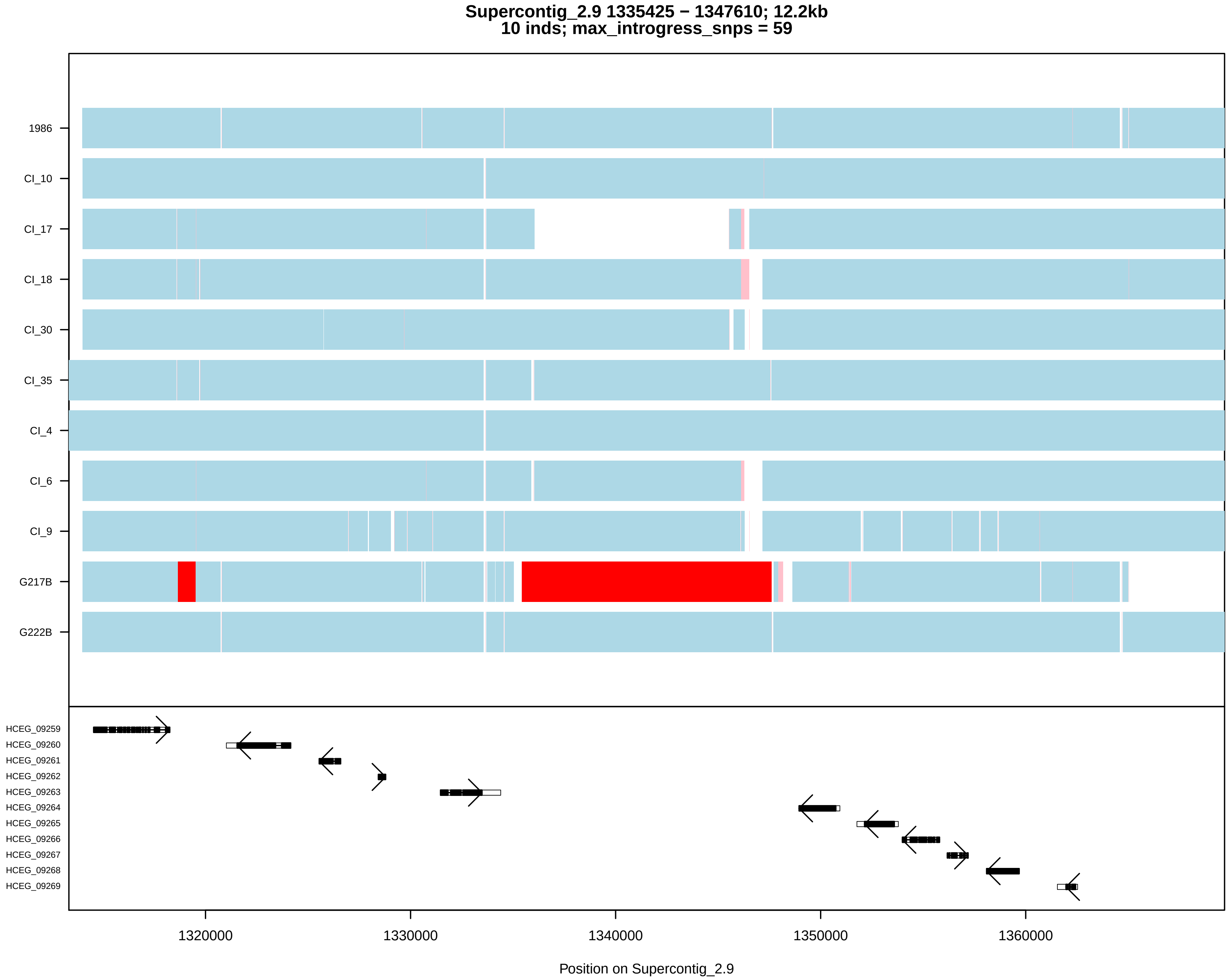

Supercontig_2.9 1335425 − 1347610; 12.2kb
10 inds; max_introgress_snps = 59
1986
CI_10
CI_17
CI_18
CI_30
CI_35
CI_4
CI_6
CI_9
G217B
G222B
HCEG_09259
HCEG_09260
HCEG_09261
HCEG_09262
HCEG_09263
HCEG_09264
HCEG_09265
HCEG_09266
HCEG_09267
HCEG_09268
HCEG_09269
1320000
1330000
1340000
1350000
1360000
Position on Supercontig_2.9

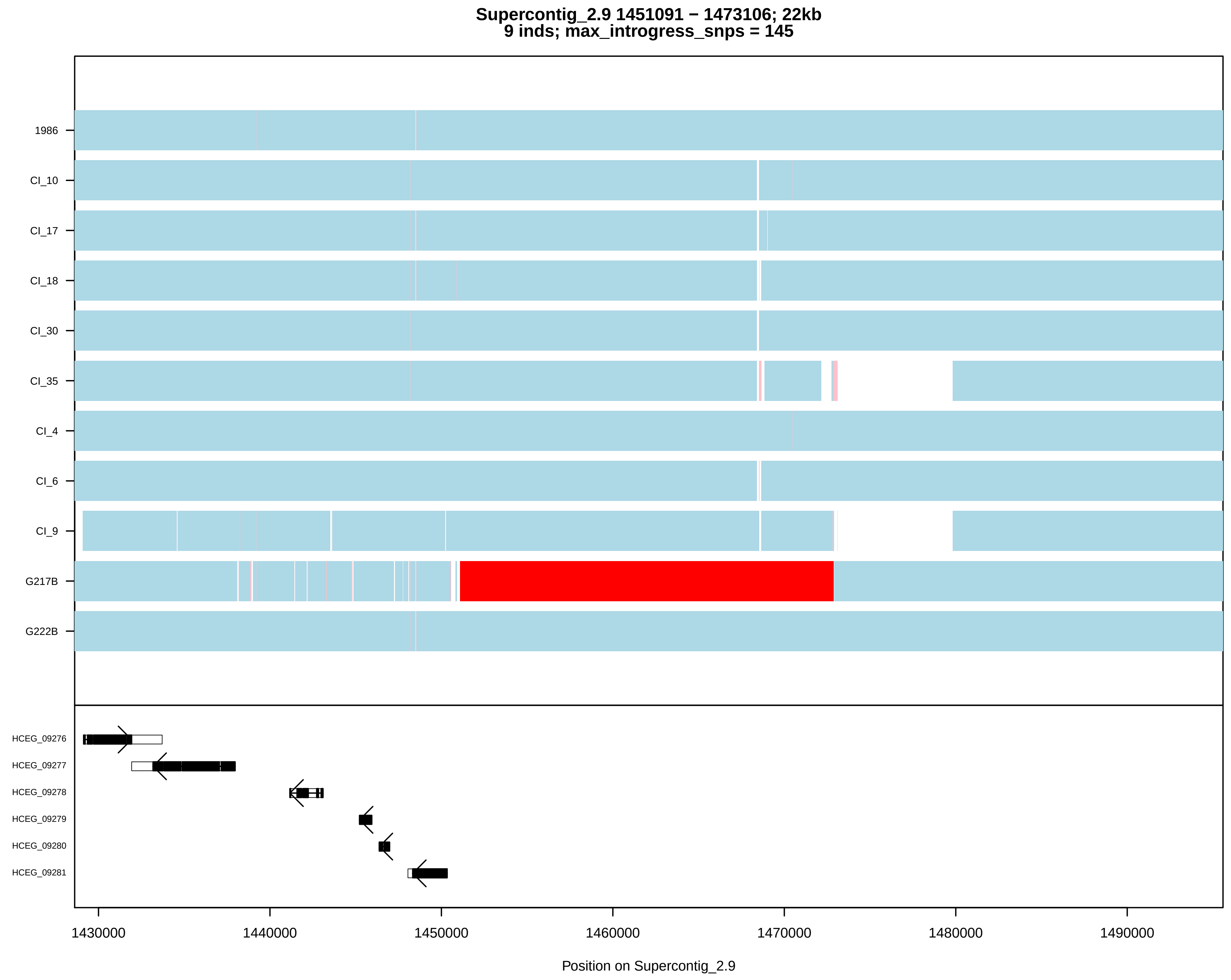
Supercontig_2.9 1451091 − 1473106; 22kb
9 inds; max_introgress_snps = 145
1986
CI_10
CI_17
CI_18
CI_30
CI_35
CI_4
CI_6
CI_9
G217B
G222B
HCEG_09276
HCEG_09277
HCEG_09278
HCEG_09279
HCEG_09280
HCEG_09281
1430000
1440000
1450000
1460000
1470000
1480000
1490000
Position on Supercontig_2.9

Supercontig_2.9 1496192 − 1507388; 11.2kb
2 inds; max_introgress_snps = 23

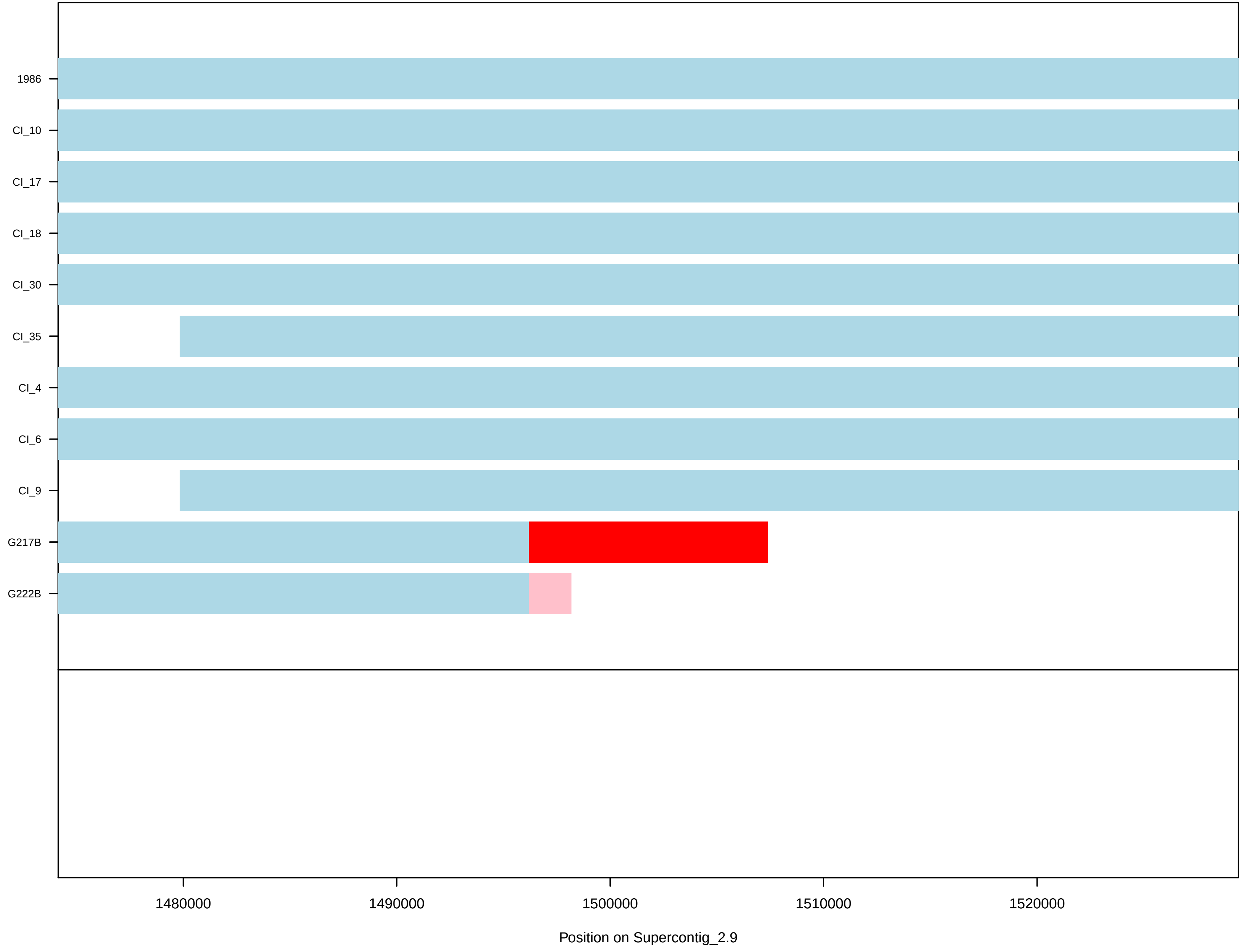

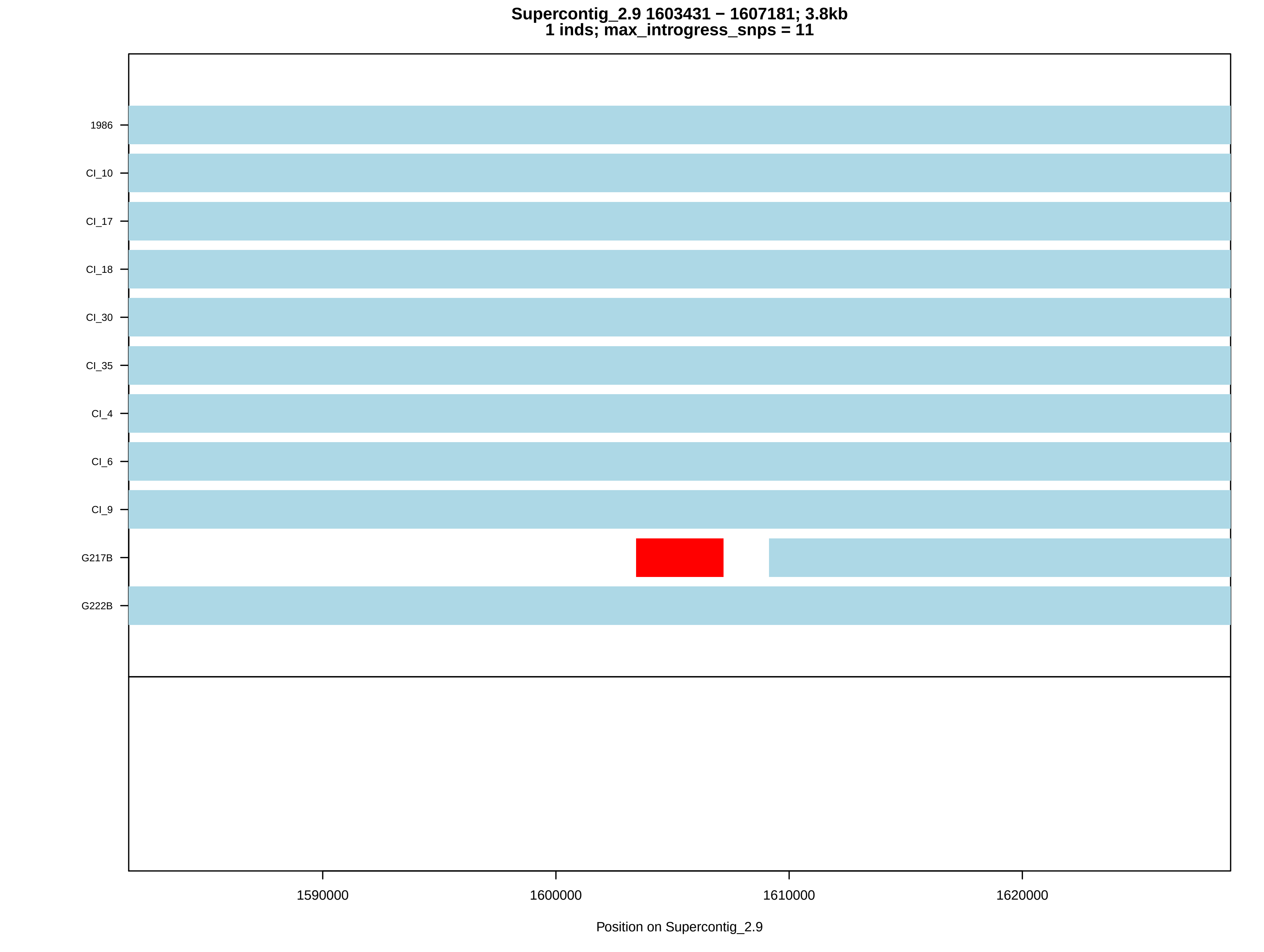
Supercontig_2.9 1603431 − 1607181; 3.8kb
1 inds; max_introgress_snps = 11
1986
CI_10
CI_17
CI_18
CI_30
CI_35
CI_4
CI_6
CI_9
G217B
G222B
1590000
1600000
1610000
1620000
Position on Supercontig_2.9
