# Supplementary material for: Recent admixture between species of the fungal pathogen Histoplasma
Source: Evol Lett. 2018 Jun 22;2(3):210–20. doi: 10.1002/evl3.59 (PMC6121842; doi:10.1002/evl3.59)
Supplement: Supplementary file 1 — FILE S1. Inferred introgressions from H. ohiense into H. mississippiense inferred using Int‐HMM. Blue markers represent the H. mississippiense background; red blocks are introgressions from H. ohiense. Each of the 152 pages of the file shows an introgression with its relevant information (position, size, number of SNPS used to infer it —max_introgress_snps—). ‘i ind’ refers to the number of individuals (i) that show any evidence for shared ancestry in the shown genomic window. Sections of the genome with no evidence for introgression are not shown. The only isolate that shows evidence for extensive introgression is H. mississippiense WU24. [file EVL3-2-210-s001.pdf]

Supercontig_2.1 8047 − 72899; 64.9kb
2 inds; max_introgress_snps = 23

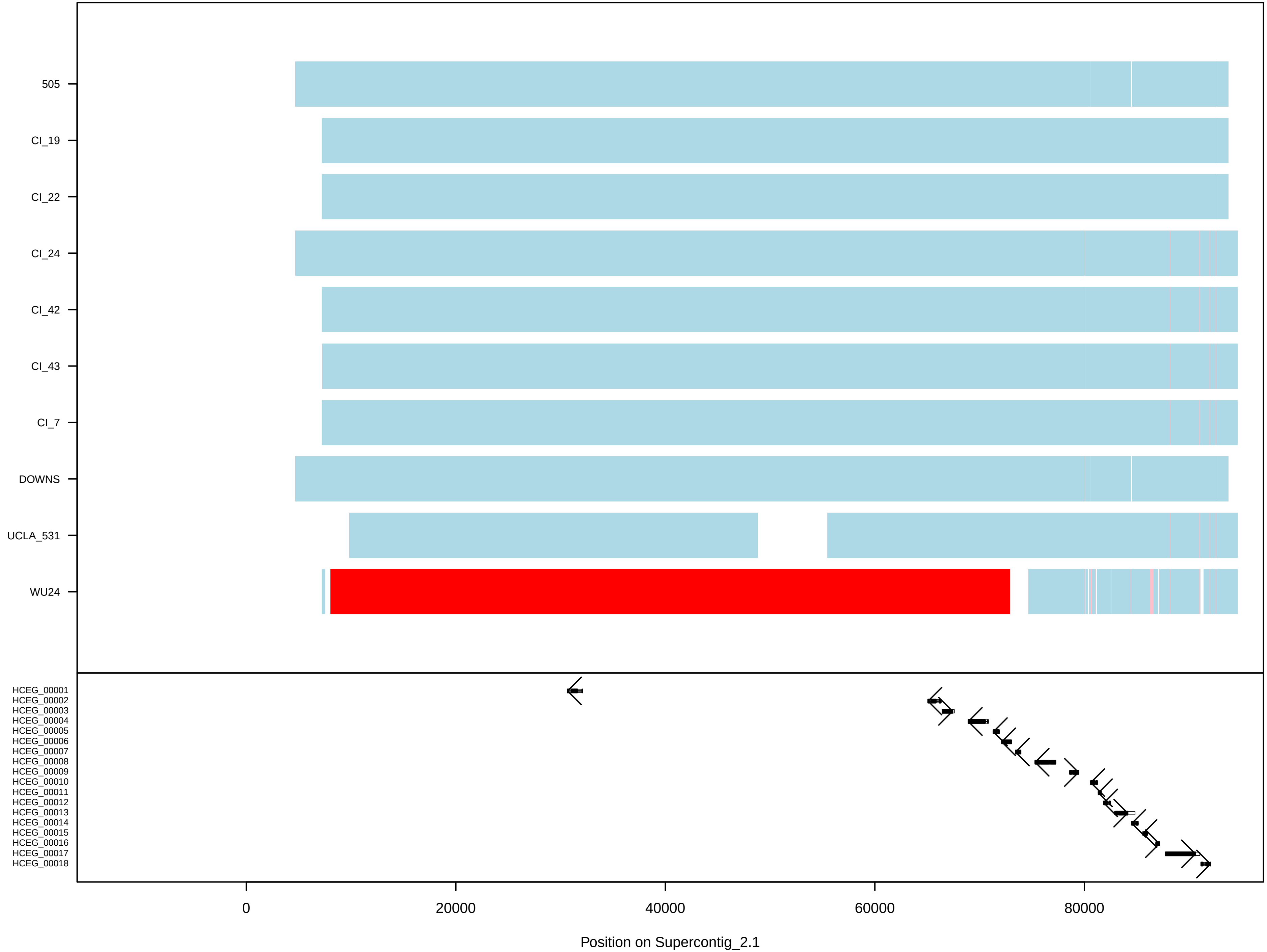

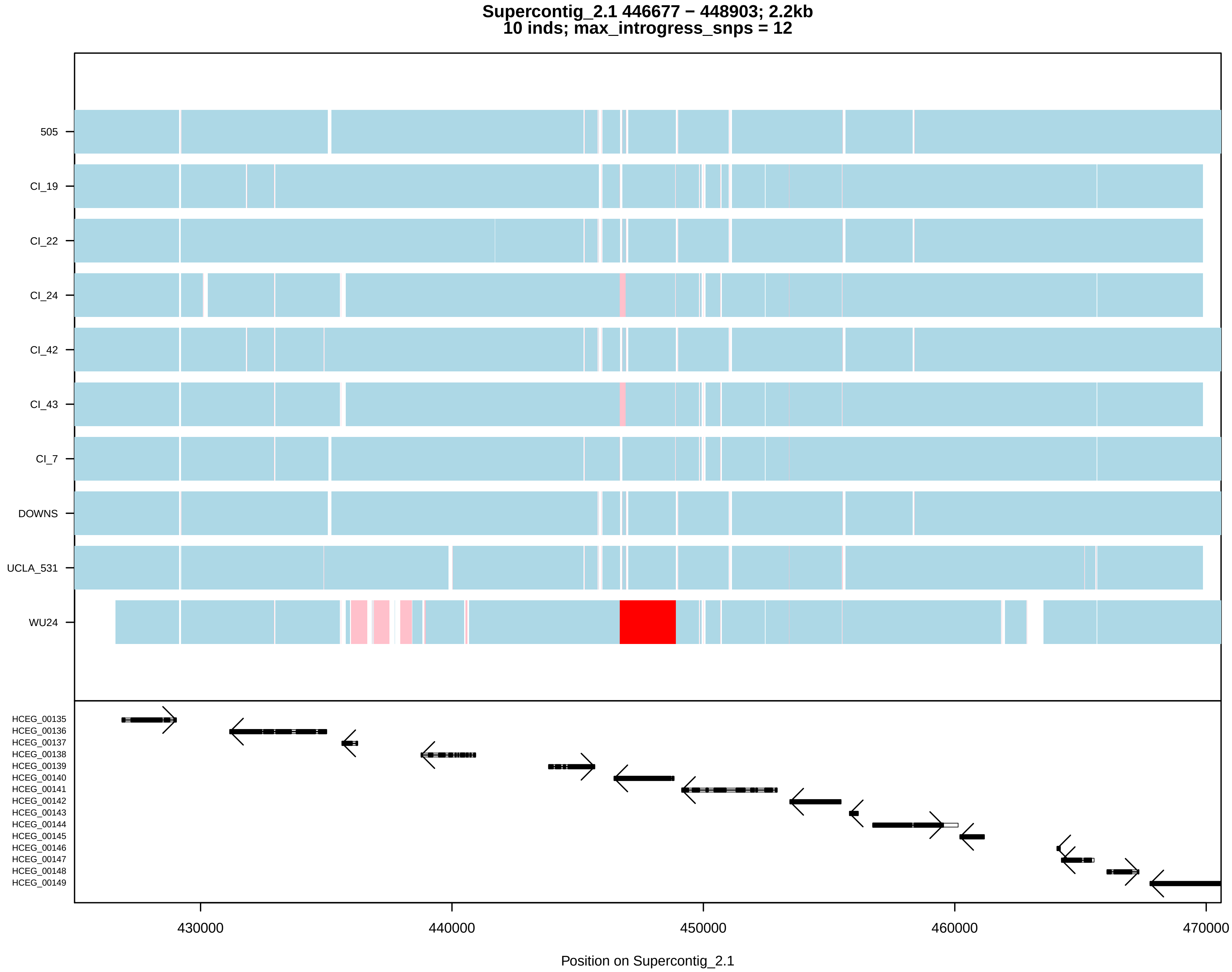

Supercontig_2.1 446677 − 448903; 2.2kb
10 inds; max_introgress_snps = 12
505
CI_19
CI_22
CI_24
CI_42
CI_43
CI_7
DOWNS
UCLA_531
WU24
HCEG_00135
HCEG_00136
HCEG_00137
HCEG_00138
HCEG_00139
HCEG_00140
HCEG_00141
HCEG_00142
HCEG_00143
HCEG_00144
HCEG_00145
HCEG_00146
HCEG_00147
HCEG_00148
HCEG_00149
430000
440000
450000
460000
470000
Position on Supercontig_2.1

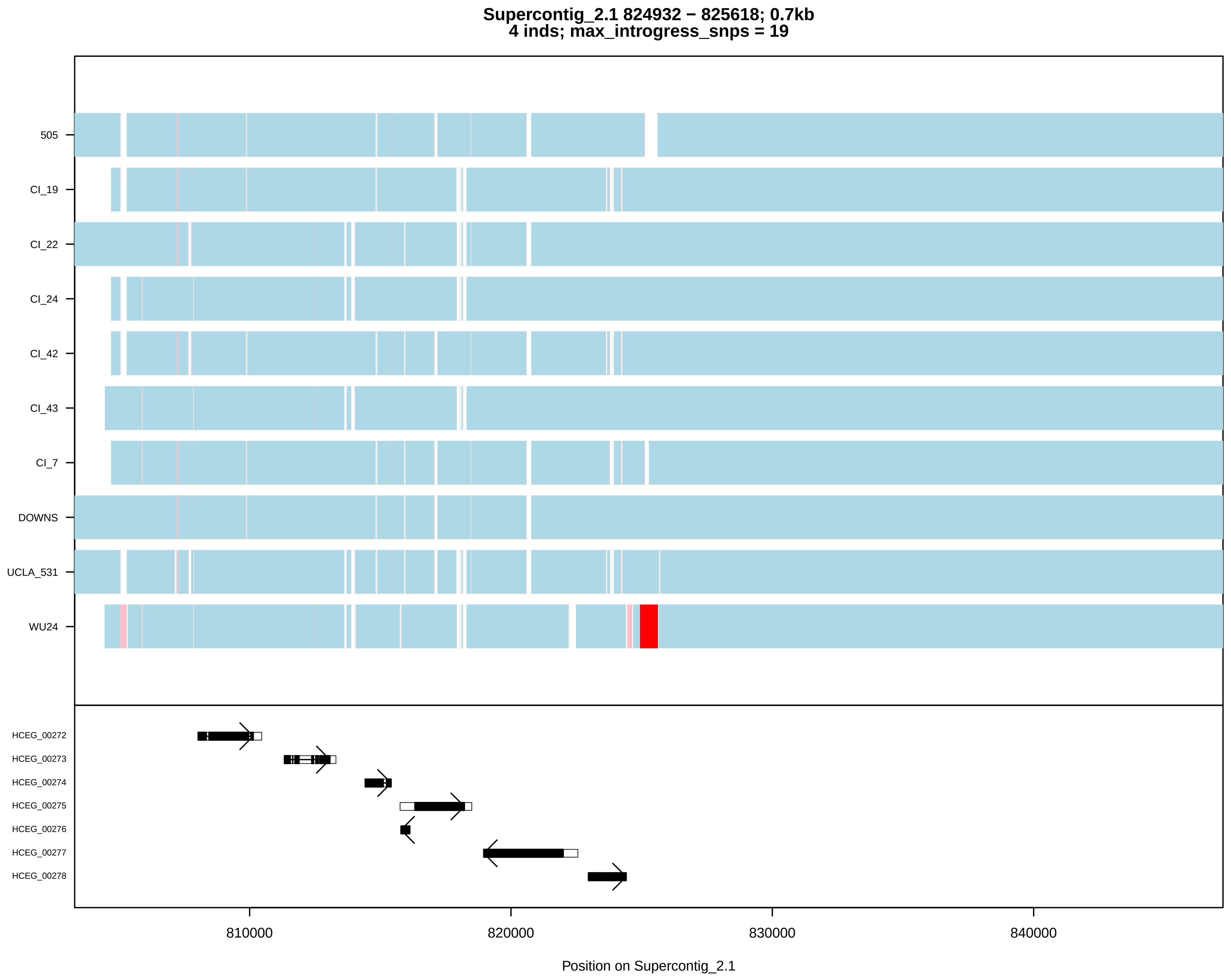
Supercontig_2.1 824932 − 825618; 0.7kb
4 inds; max_introgress_snps = 19
505
CI_19
CI_22
CI_24
CI_42
CI_43
CI_7
DOWNS
UCLA_531
WU24
HCEG_00272
HCEG_00273
HCEG_00274
HCEG_00275
HCEG_00276
HCEG_00277
HCEG_00278
810000
820000
830000
840000
Position on Supercontig_2.1

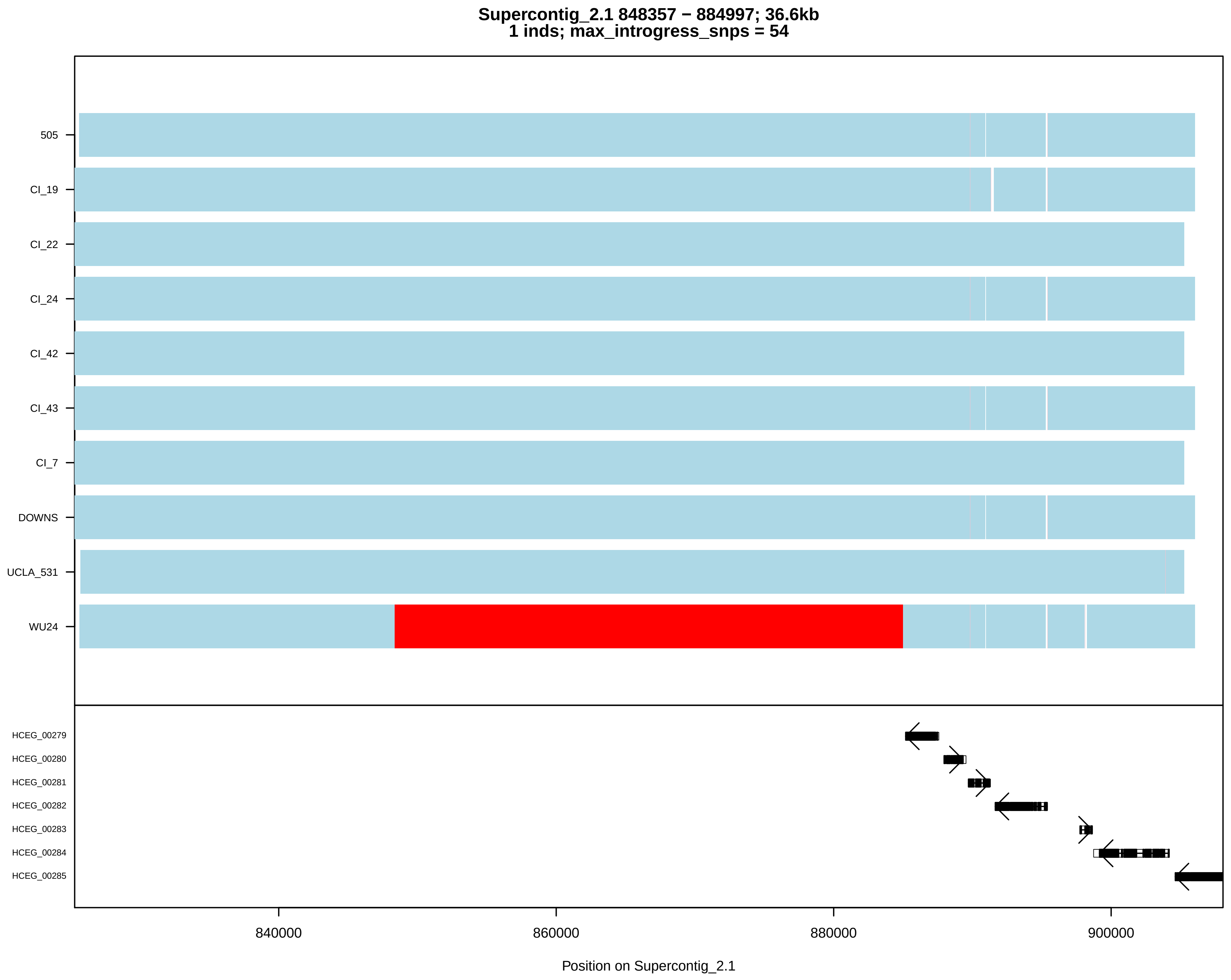
Supercontig_2.1 848357 − 884997; 36.6kb
1 inds; max_introgress_snps = 54
505
CI_19
CI_22
CI_24
CI_42
CI_43
CI_7
DOWNS
UCLA_531
WU24
HCEG_00279
HCEG_00280
HCEG_00281
HCEG_00282
HCEG_00283
HCEG_00284
HCEG_00285
840000
860000
880000
900000
Position on Supercontig_2.1

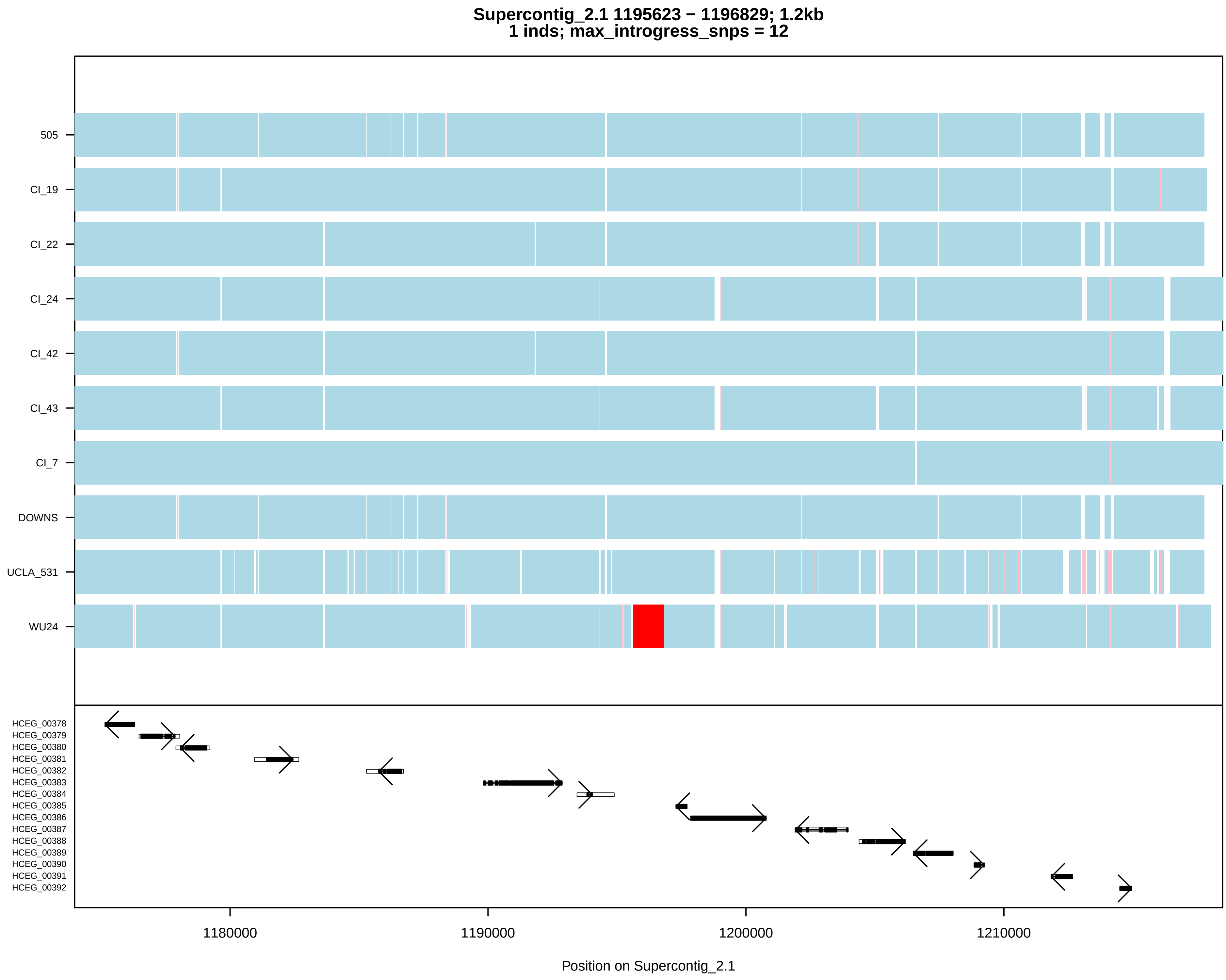
Supercontig_2.1 1195623 − 1196829; 1.2kb
1 inds; max_introgress_snps = 12
505
CI_19
CI_22
CI_24
CI_42
CI_43
CI_7
DOWNS
UCLA_531
WU24
HCEG_00378
HCEG_00379
HCEG_00380
HCEG_00381
HCEG_00382
HCEG_00383
HCEG_00384
HCEG_00385
HCEG_00386
HCEG_00387
HCEG_00388
HCEG_00389
HCEG_00390
HCEG_00391
HCEG_00392
1180000
1190000
1200000
1210000
Position on Supercontig_2.1

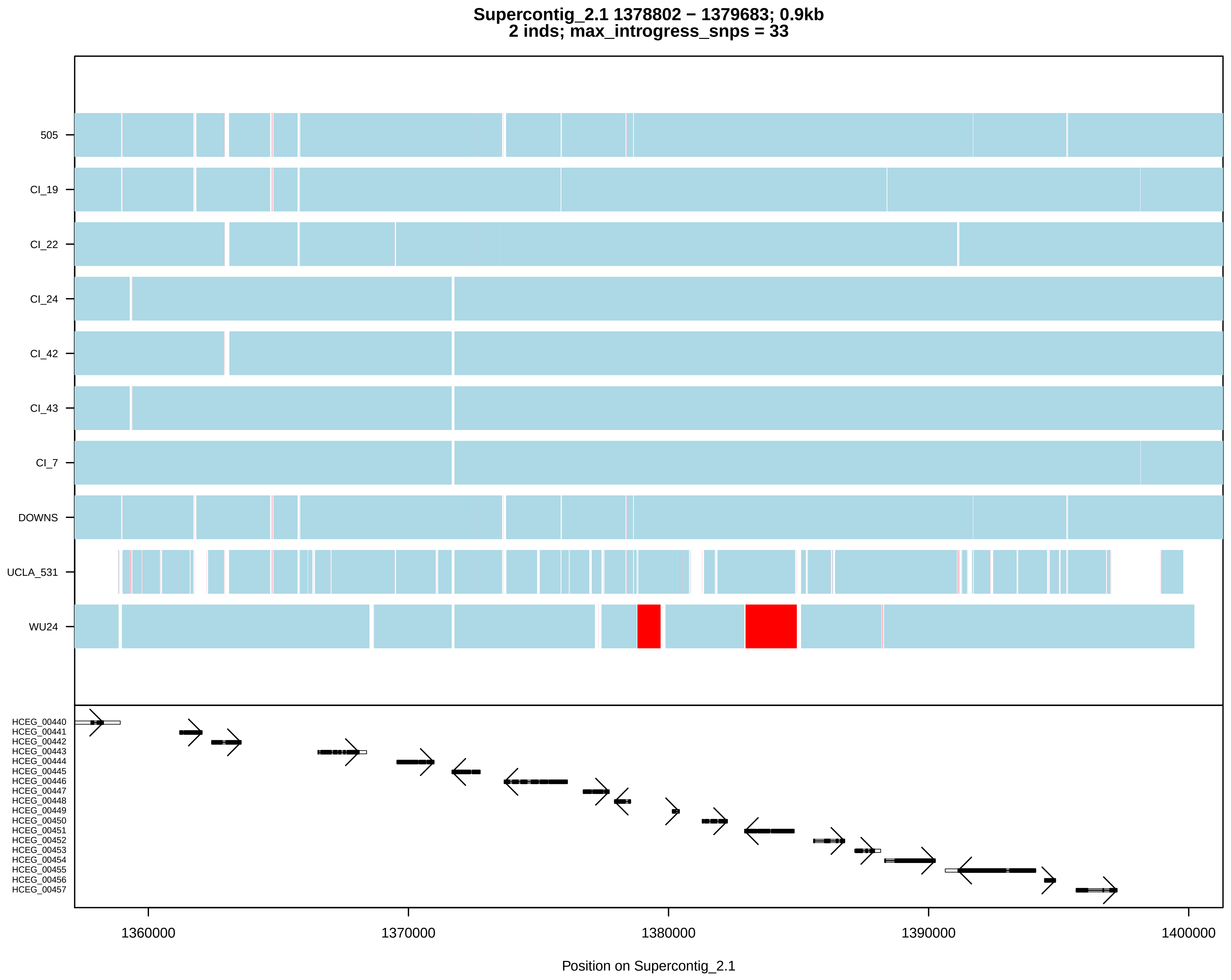
Supercontig_2.1 1378802 − 1379683; 0.9kb
2 inds; max_introgress_snps = 33
505
CI_19
CI_22
CI_24
CI_42
CI_43
CI_7
DOWNS
UCLA_531
WU24
HCEG_00440
HCEG_00441
HCEG_00442
HCEG_00443
HCEG_00444
HCEG_00445
HCEG_00446
HCEG_00447
HCEG_00448
HCEG_00449
HCEG_00450
HCEG_00451
HCEG_00452
HCEG_00453
HCEG_00454
HCEG_00455
HCEG_00456
HCEG_00457
1360000
1370000
1380000
1390000
1400000
Position on Supercontig_2.1

Supercontig_2.1 1382960 − 1384922; 2kb
2 inds; max_introgress_snps = 47

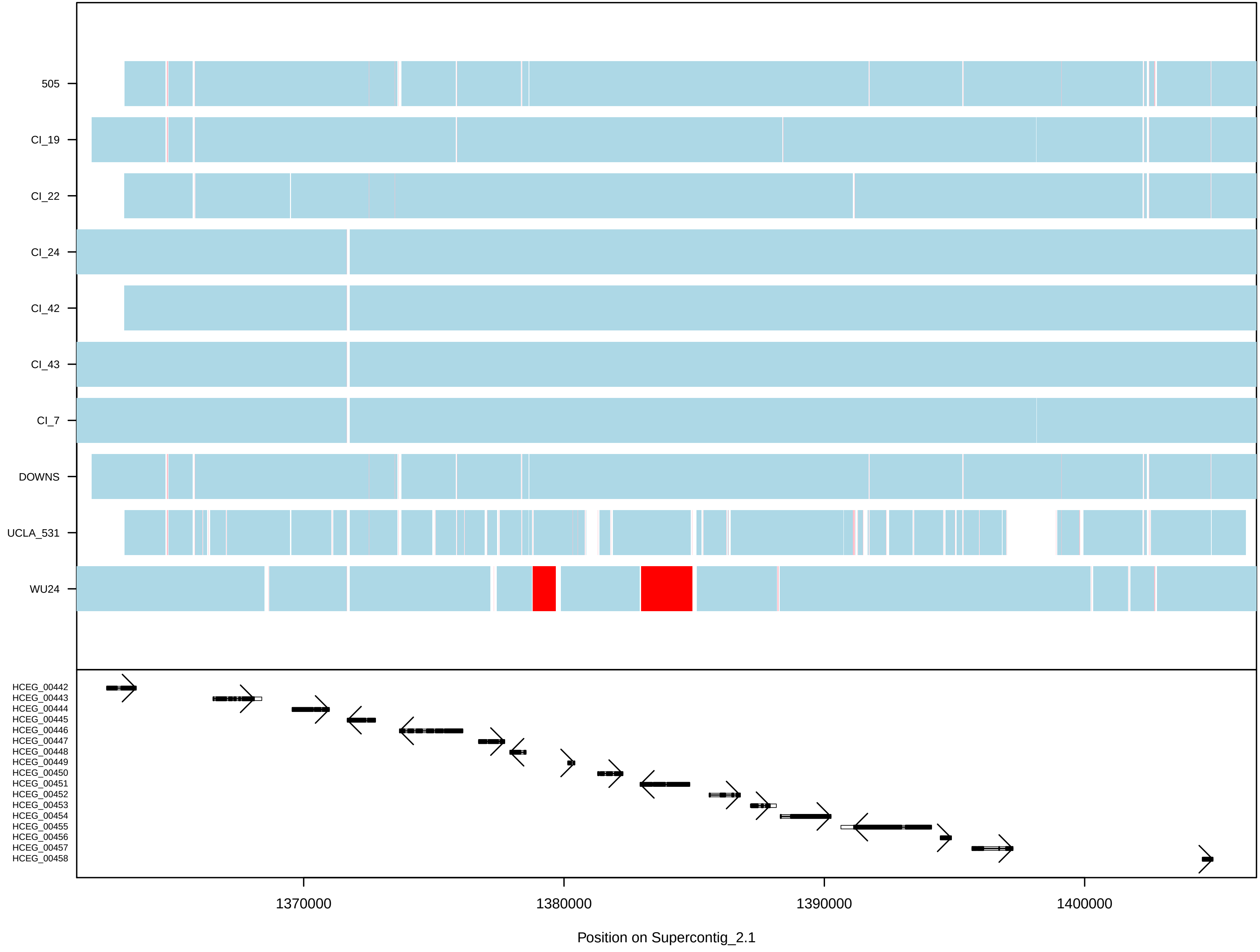

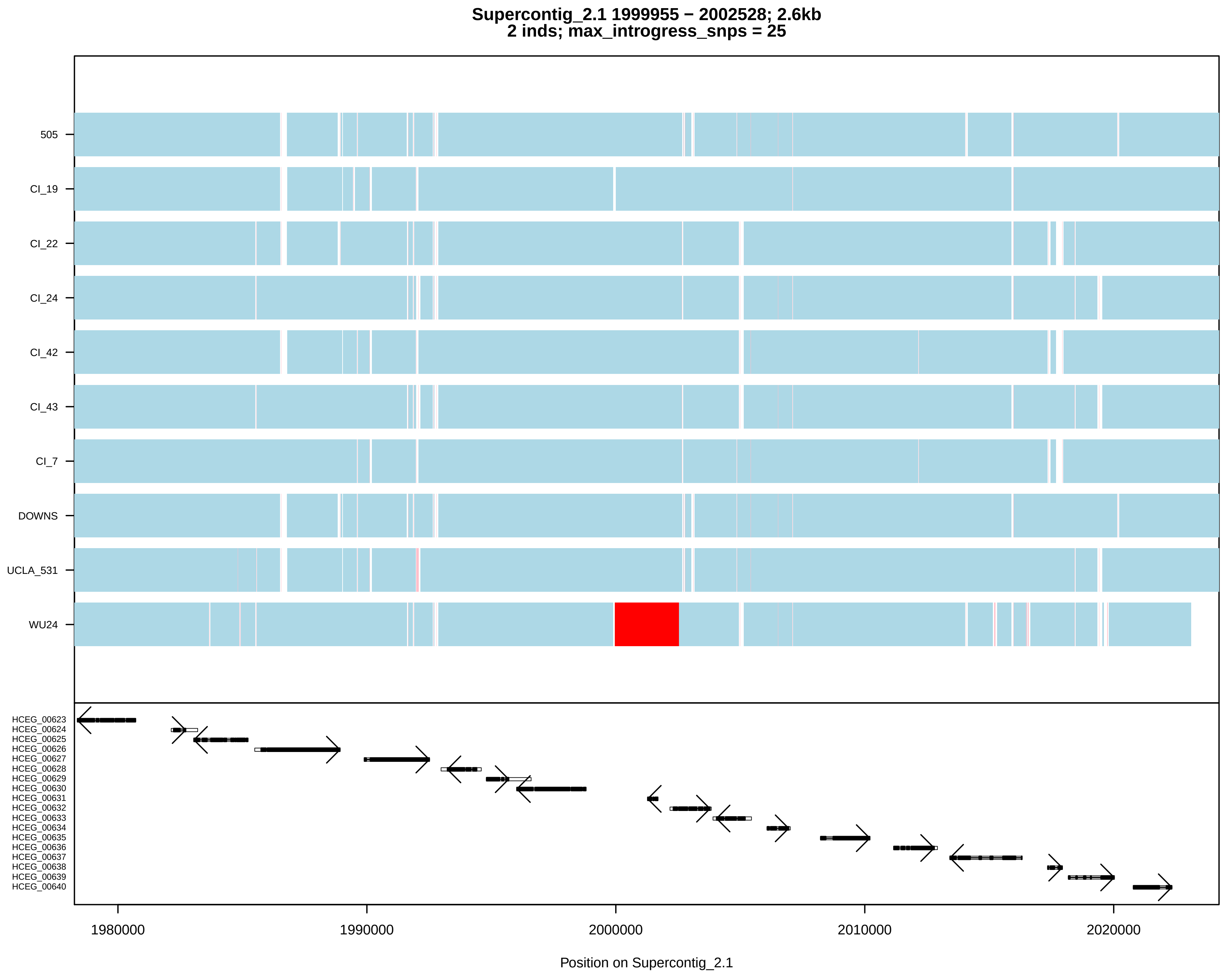

Supercontig_2.1 1999955 − 2002528; 2.6kb
2 inds; max_introgress_snps = 25
505
CI_19
CI_22
CI_24
CI_42
CI_43
CI_7
DOWNS
UCLA_531
WU24
HCEG_00623
HCEG_00624
HCEG_00625
HCEG_00626
HCEG_00627
HCEG_00628
HCEG_00629
HCEG_00630
HCEG_00631
HCEG_00632
HCEG_00633
HCEG_00634
HCEG_00635
HCEG_00636
HCEG_00637
HCEG_00638
HCEG_00639
HCEG_00640
1980000
1990000
2000000
2010000
2020000
Position on Supercontig_2.1

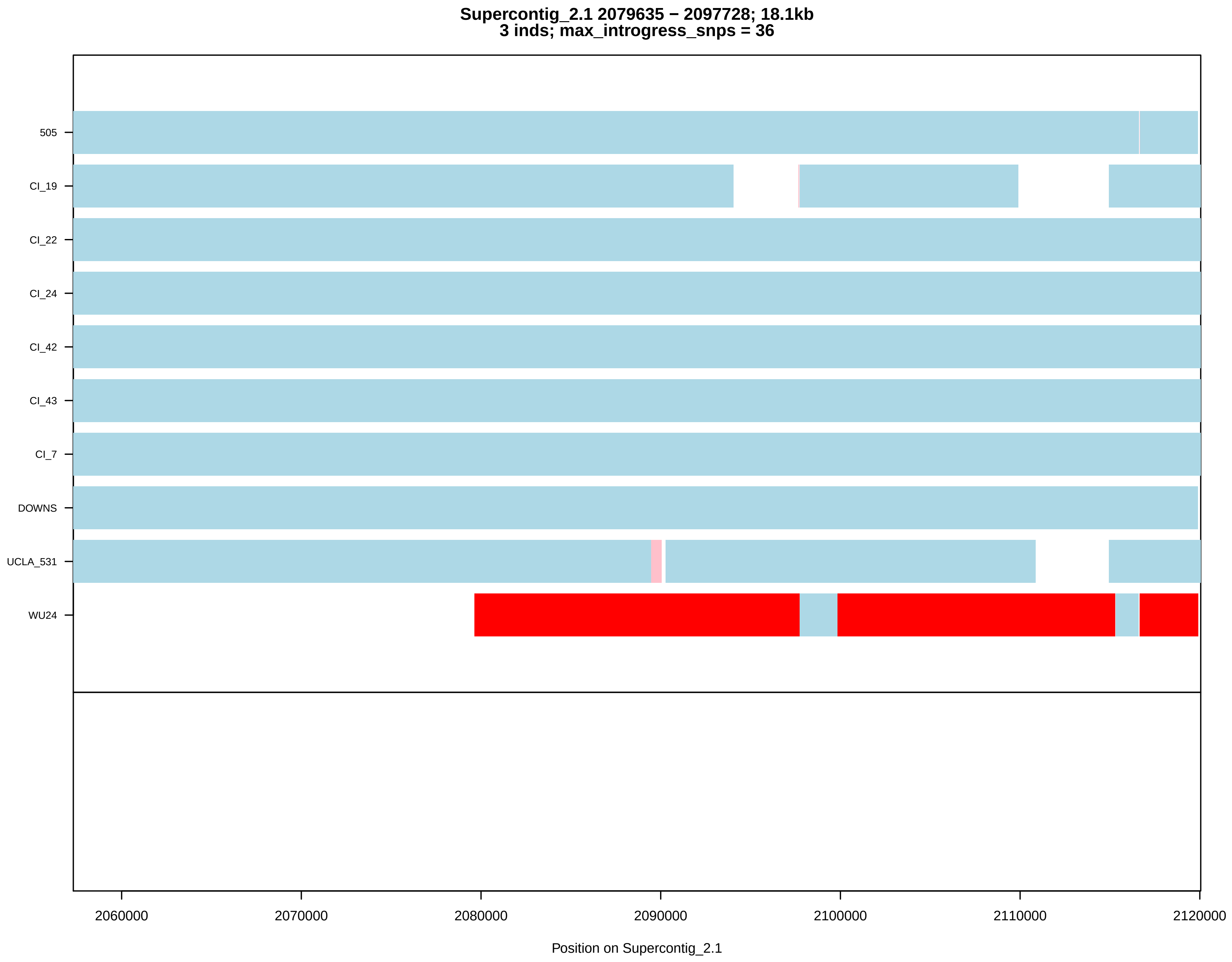
Supercontig_2.1 2079635 − 2097728; 18.1kb
3 inds; max_introgress_snps = 36
505
CI_19
CI_22
CI_24
CI_42
CI_43
CI_7
DOWNS
UCLA_531
WU24
2060000
2070000
2080000
2090000
2100000
2110000
2120000
Position on Supercontig_2.1

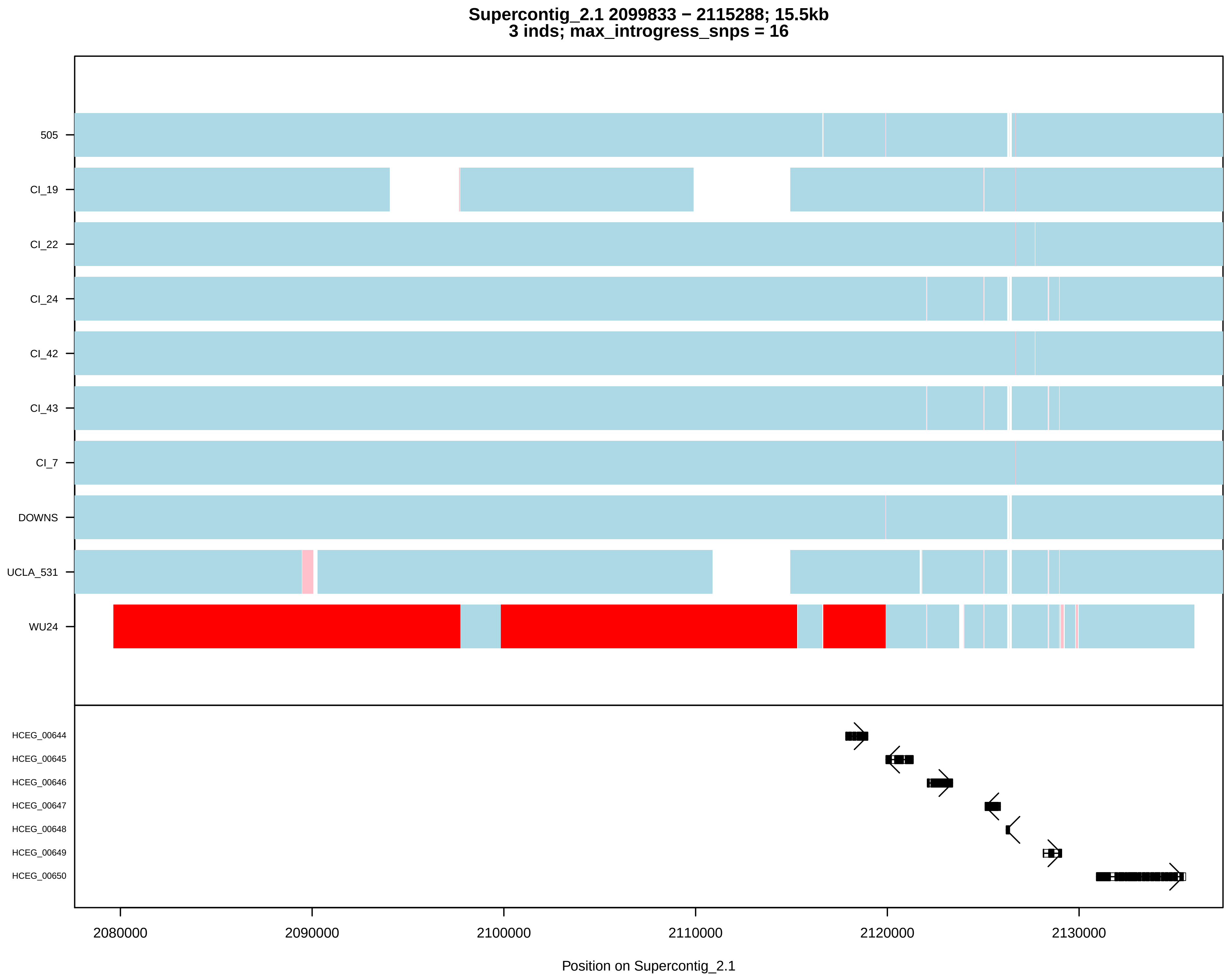
Supercontig_2.1 2099833 − 2115288; 15.5kb
3 inds; max_introgress_snps = 16
505
CI_19
CI_22
CI_24
CI_42
CI_43
CI_7
DOWNS
UCLA_531
WU24
HCEG_00644
HCEG_00645
HCEG_00646
HCEG_00647
HCEG_00648
HCEG_00649
HCEG_00650
2080000
2090000
2100000
2110000
2120000
2130000
Position on Supercontig_2.1

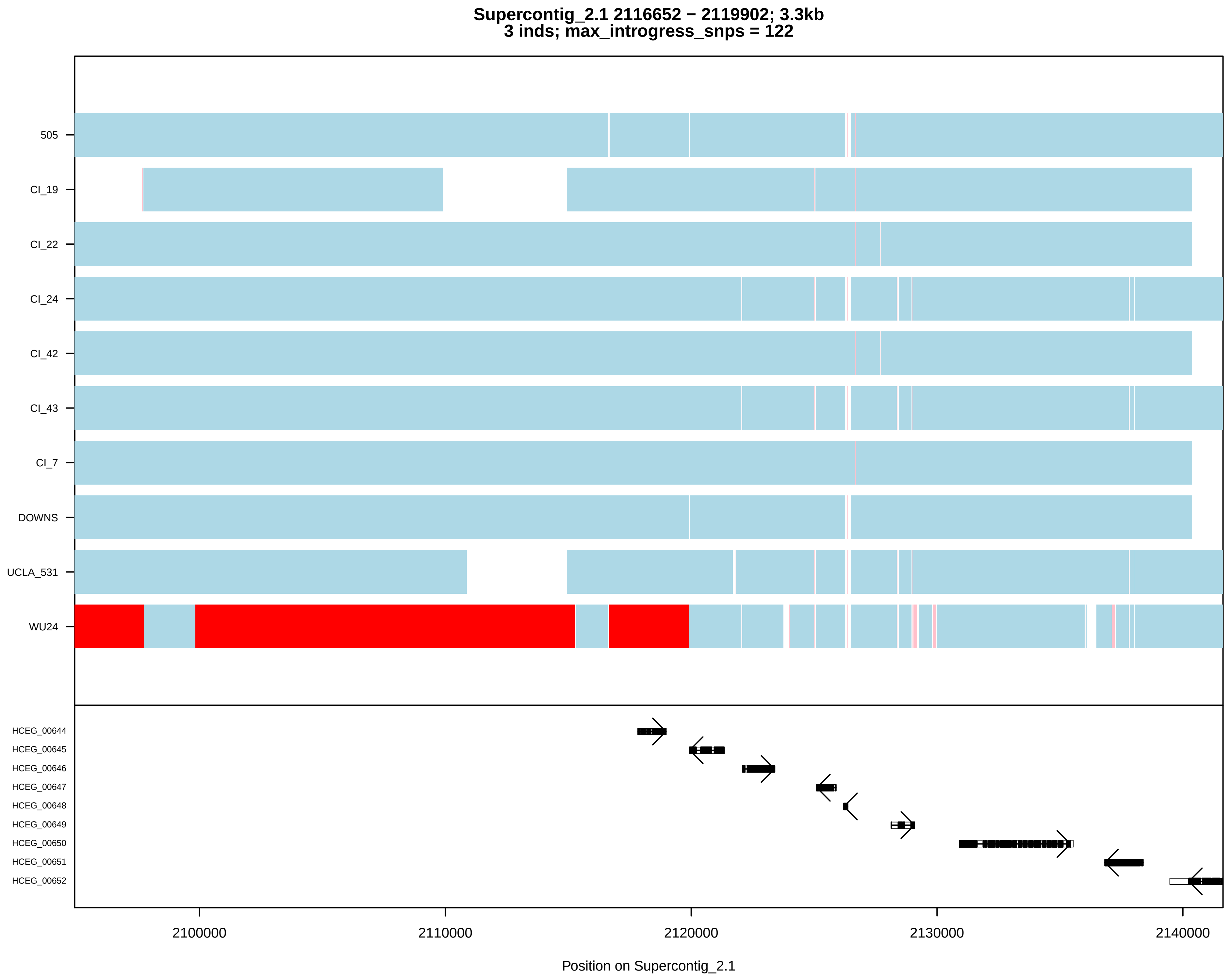
Supercontig_2.1 2116652 − 2119902; 3.3kb
3 inds; max_introgress_snps = 122
505
CI_19
CI_22
CI_24
CI_42
CI_43
CI_7
DOWNS
UCLA_531
WU24
HCEG_00644
HCEG_00645
HCEG_00646
HCEG_00647
HCEG_00648
HCEG_00649
HCEG_00650
HCEG_00651
HCEG_00652
2100000
2110000
2120000
2130000
2140000
Position on Supercontig_2.1

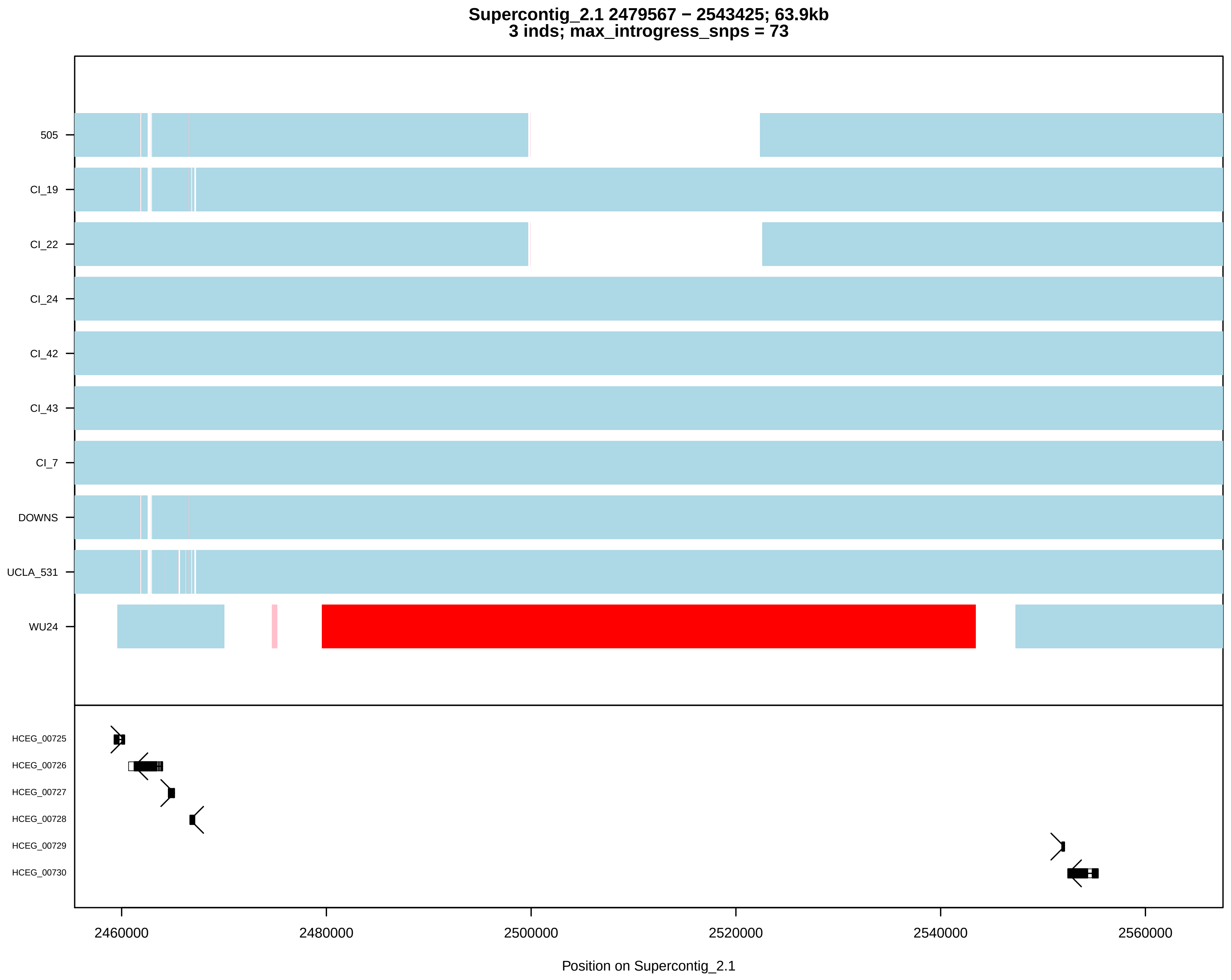

Supercontig_2.1 2479567 − 2543425; 63.9kb
3 inds; max_introgress_snps = 73
505
CI_19
CI_22
CI_24
CI_42
CI_43
CI_7
DOWNS
UCLA_531
WU24
HCEG_00725
HCEG_00726
HCEG_00727
HCEG_00728
HCEG_00729
HCEG_00730
2460000
2480000
2500000
2520000
2540000
2560000
Position on Supercontig_2.1

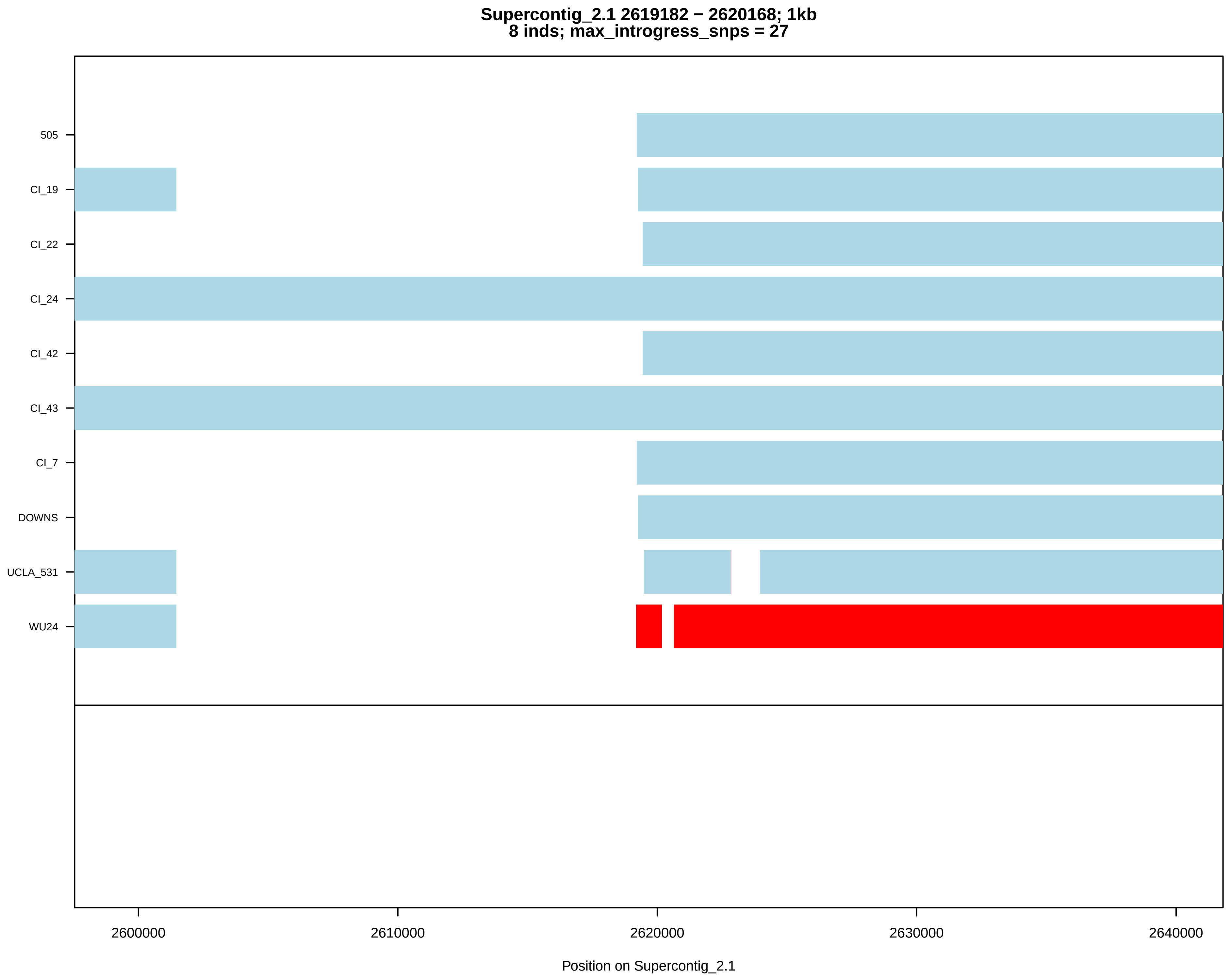

Supercontig_2.1 2619182 − 2620168; 1kb
8 inds; max_introgress_snps = 27
505
CI_19
CI_22
CI_24
CI_42
CI_43
CI_7
DOWNS
UCLA_531
WU24
2600000
2610000
2620000
2630000
2640000
Position on Supercontig_2.1

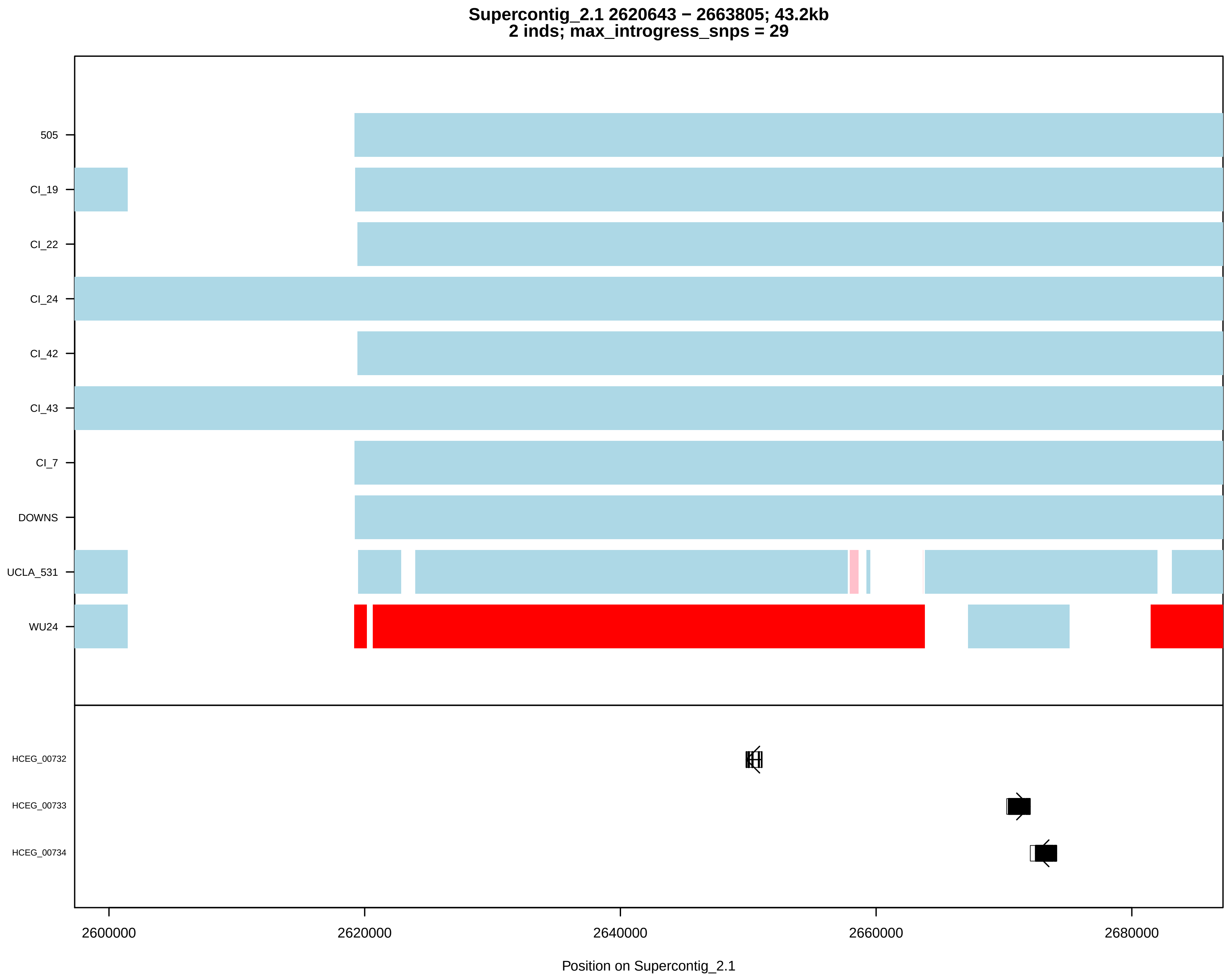

Supercontig_2.1 2620643 − 2663805; 43.2kb
2 inds; max_introgress_snps = 29
505
CI_19
CI_22
CI_24
CI_42
CI_43
CI_7
DOWNS
UCLA_531
WU24
HCEG_00732
HCEG_00733
HCEG_00734
2600000
2620000
2640000
2660000
2680000
Position on Supercontig_2.1

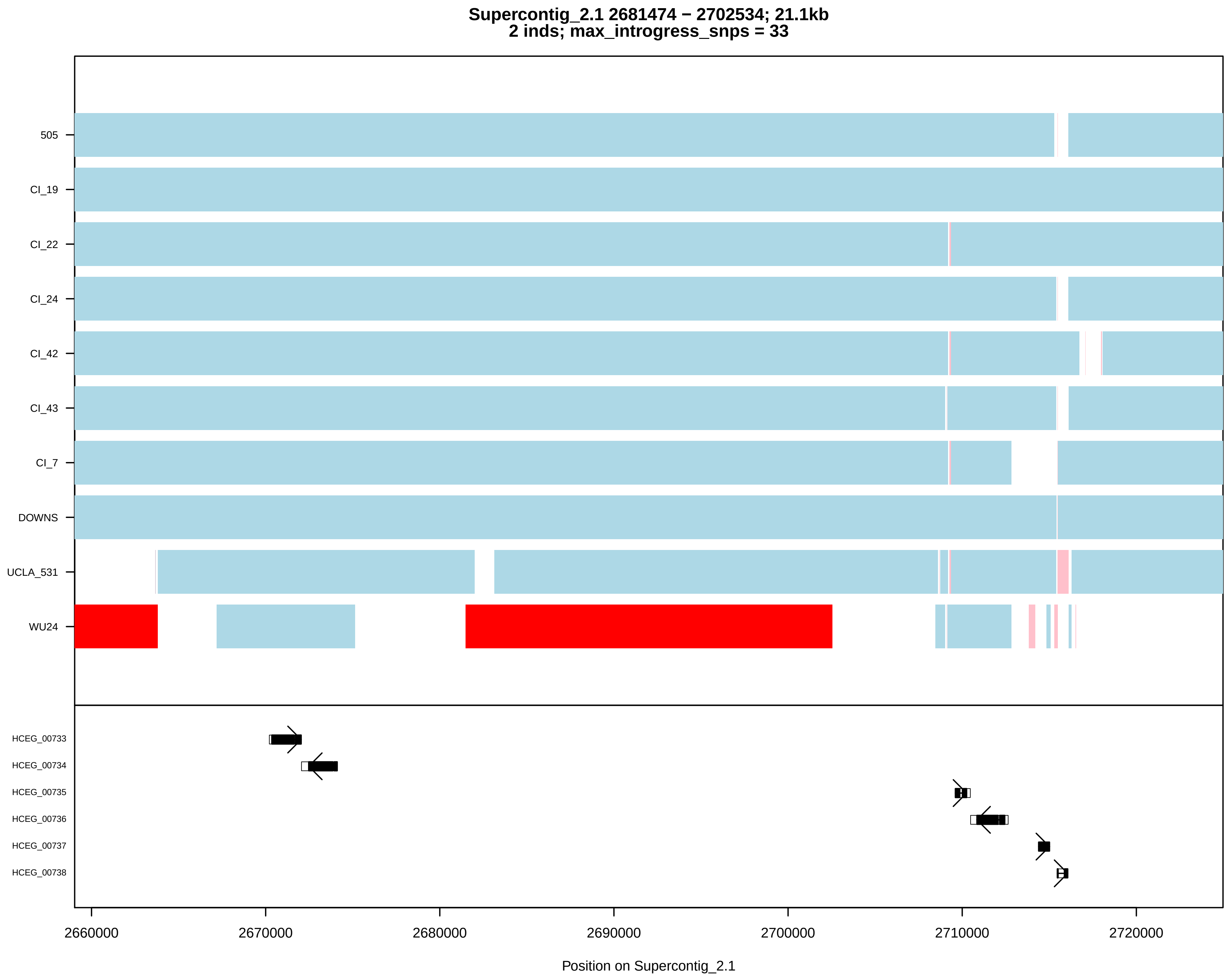

Supercontig_2.1 2681474 − 2702534; 21.1kb
2 inds; max_introgress_snps = 33
505
CI_19
CI_22
CI_24
CI_42
CI_43
CI_7
DOWNS
UCLA_531
WU24
HCEG_00733
HCEG_00734
HCEG_00735
HCEG_00736
HCEG_00737
HCEG_00738
2660000
2670000
2680000
2690000
2700000
2710000
2720000
Position on Supercontig_2.1

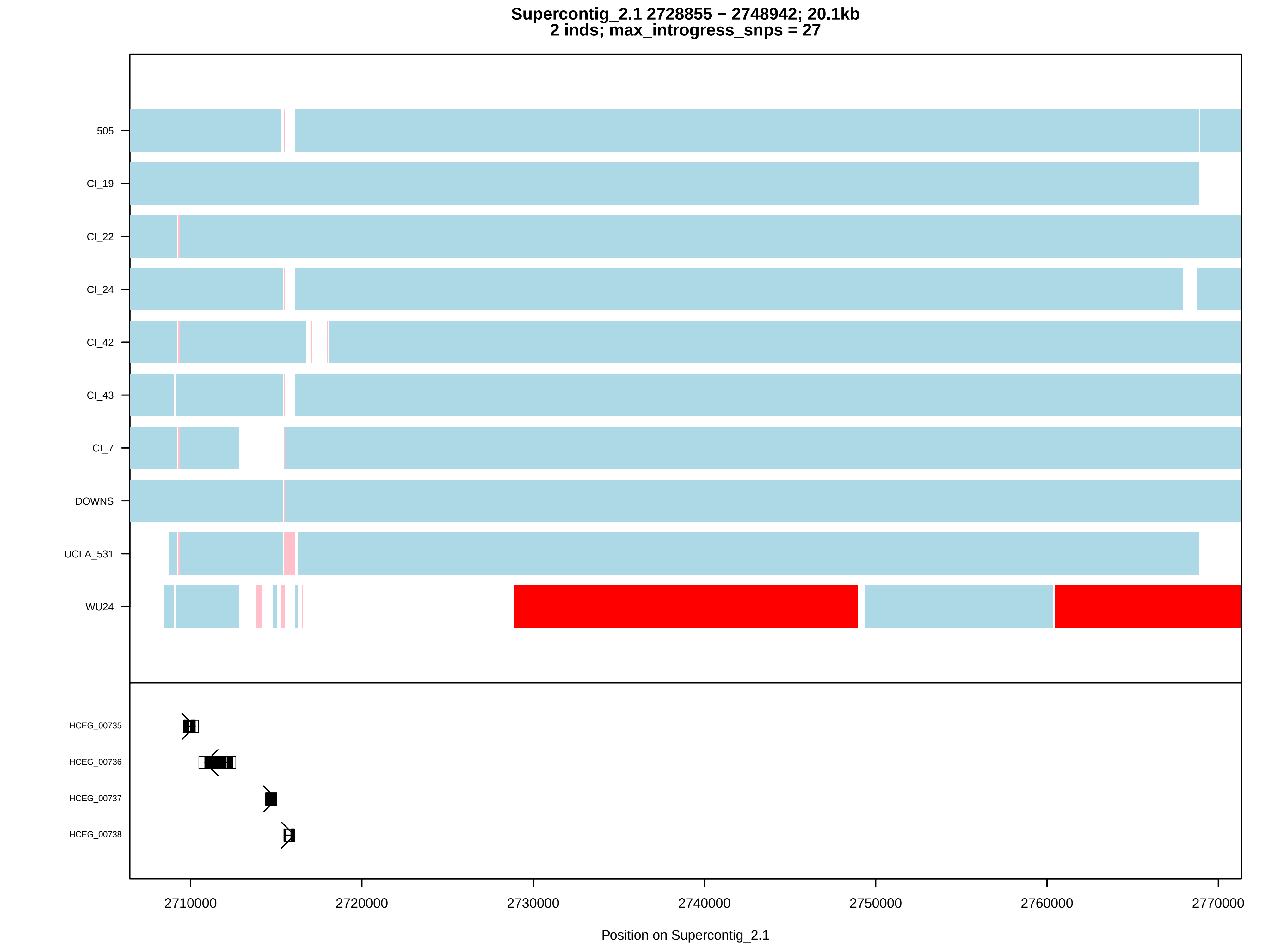

Supercontig_2.1 2728855 − 2748942; 20.1kb
2 inds; max_introgress_snps = 27
505
CI_19
CI_22
CI_24
CI_42
CI_43
CI_7
DOWNS
UCLA_531
WU24
HCEG_00735
HCEG_00736
HCEG_00737
HCEG_00738
2710000
2720000
2730000
2740000
2750000
2760000
2770000
Position on Supercontig_2.1

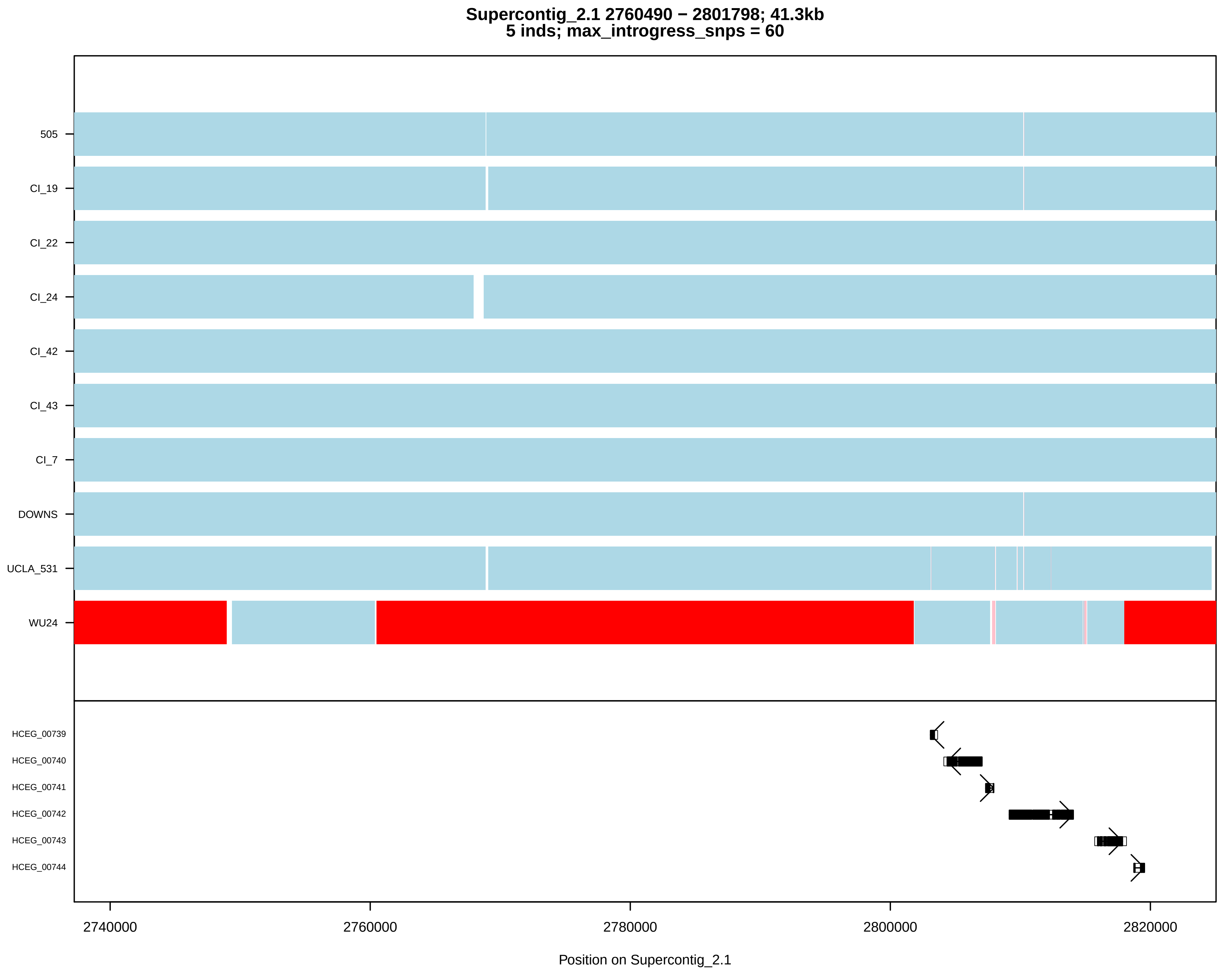

Supercontig_2.1 2760490 − 2801798; 41.3kb
5 inds; max_introgress_snps = 60
505
CI_19
CI_22
CI_24
CI_42
CI_43
CI_7
DOWNS
UCLA_531
WU24
HCEG_00739
HCEG_00740
HCEG_00741
HCEG_00742
HCEG_00743
HCEG_00744
2740000
2760000
2780000
2800000
2820000
Position on Supercontig_2.1

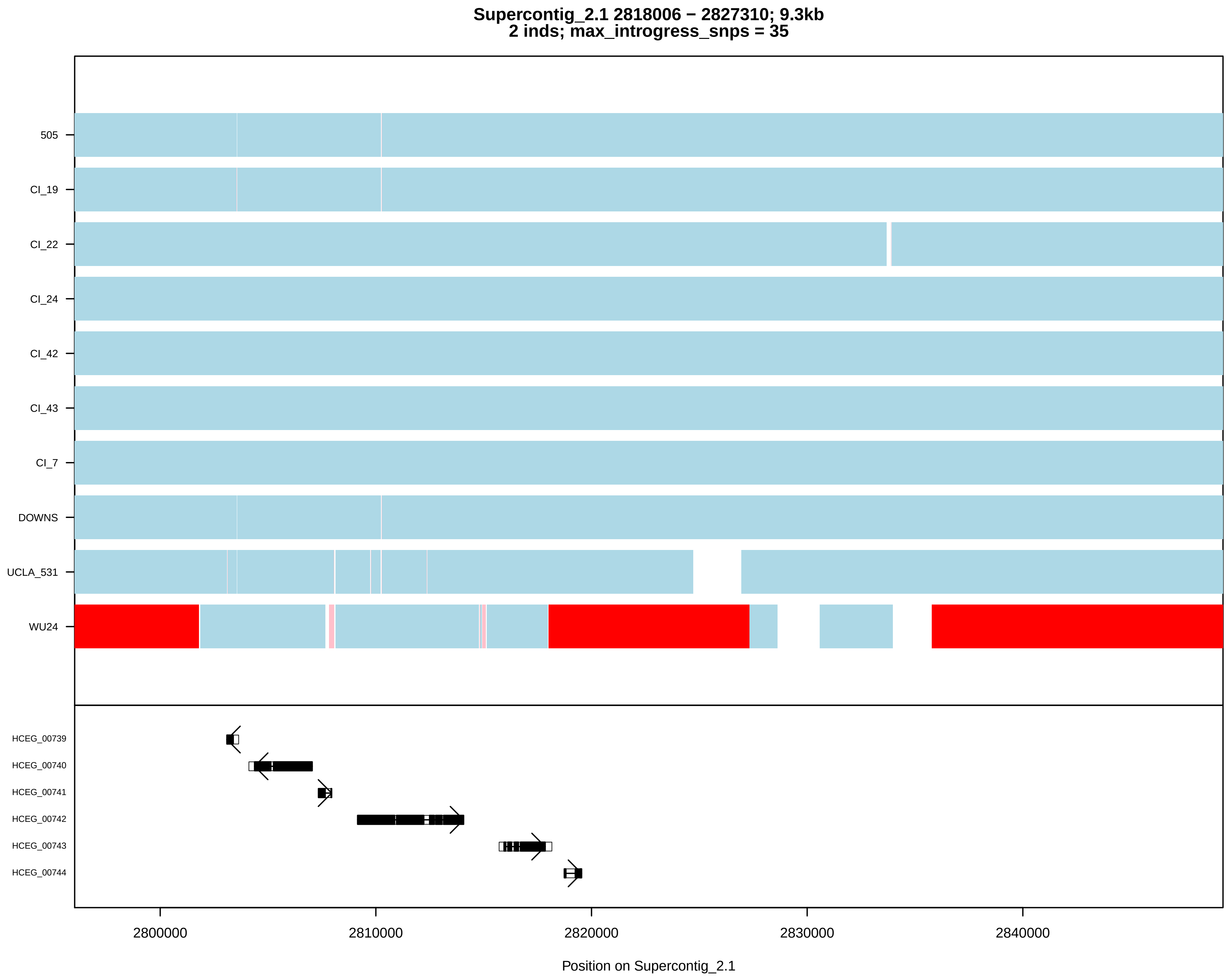
Supercontig_2.1 2818006 − 2827310; 9.3kb
2 inds; max_introgress_snps = 35
505
CI_19
CI_22
CI_24
CI_42
CI_43
CI_7
DOWNS
UCLA_531
WU24
HCEG_00739
HCEG_00740
HCEG_00741
HCEG_00742
HCEG_00743
HCEG_00744
2800000
2810000
2820000
2830000
2840000
Position on Supercontig_2.1

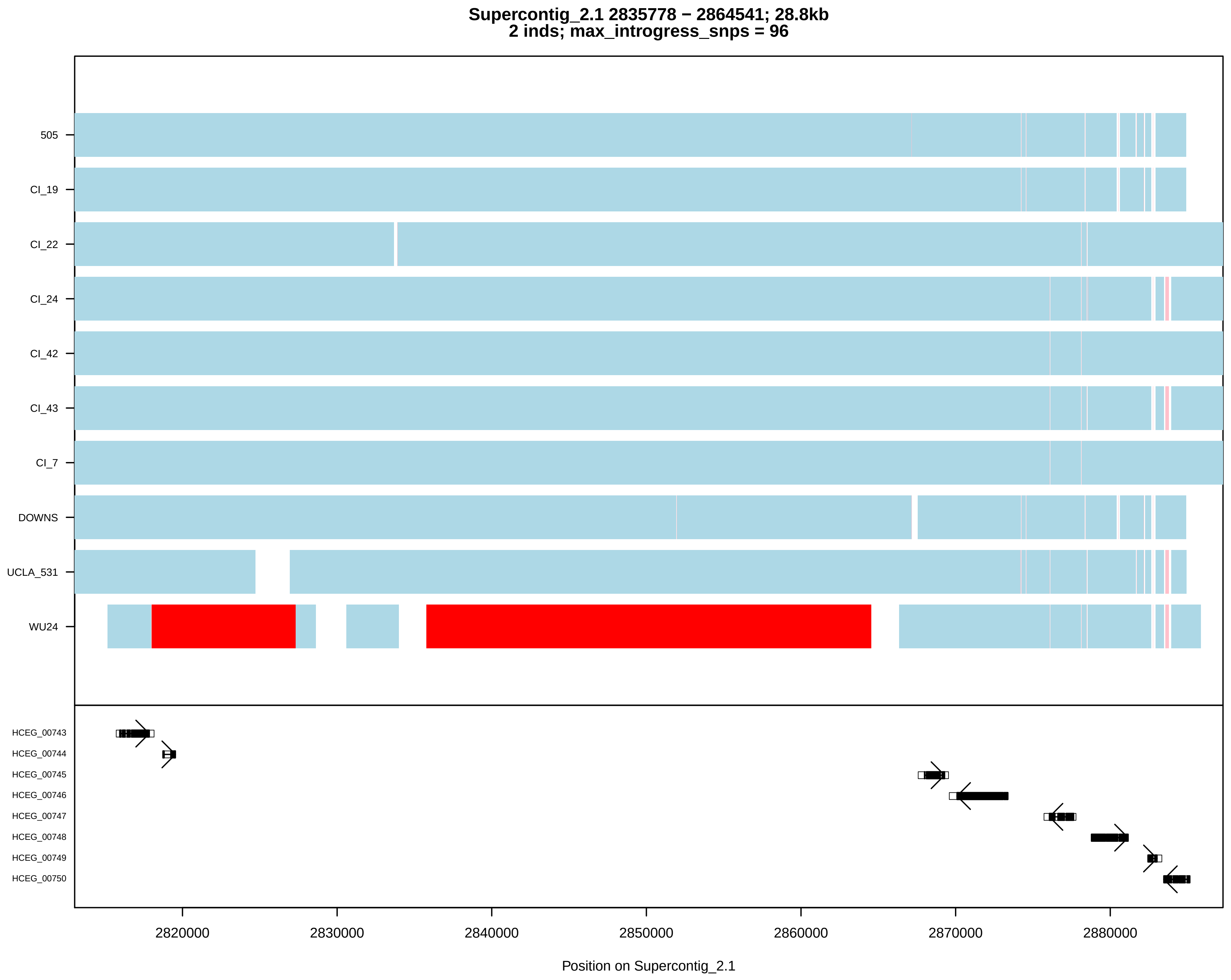

Supercontig_2.1 2835778 − 2864541; 28.8kb
2 inds; max_introgress_snps = 96
505
CI_19
CI_22
CI_24
CI_42
CI_43
CI_7
DOWNS
UCLA_531
WU24
HCEG_00743
HCEG_00744
HCEG_00745
HCEG_00746
HCEG_00747
HCEG_00748
HCEG_00749
HCEG_00750
2820000
2830000
2840000
2850000
2860000
2870000
2880000
Position on Supercontig_2.1

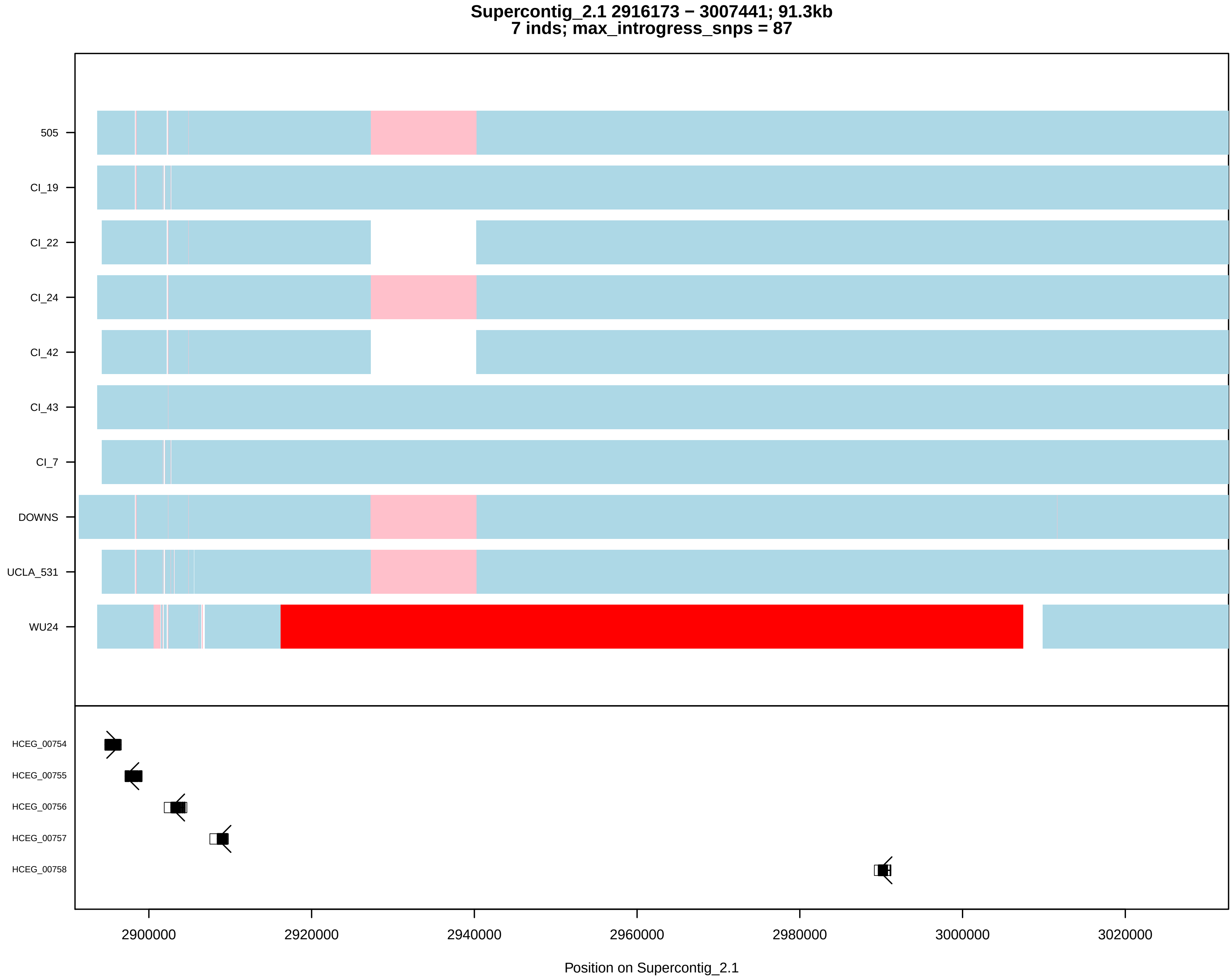

Supercontig_2.1 2916173 − 3007441; 91.3kb
7 inds; max_introgress_snps = 87
505
CI_19
CI_22
CI_24
CI_42
CI_43
CI_7
DOWNS
UCLA_531
WU24
HCEG_00754
HCEG_00755
HCEG_00756
HCEG_00757
HCEG_00758
2900000
2920000
2940000
2960000
2980000
3000000
3020000
Position on Supercontig_2.1

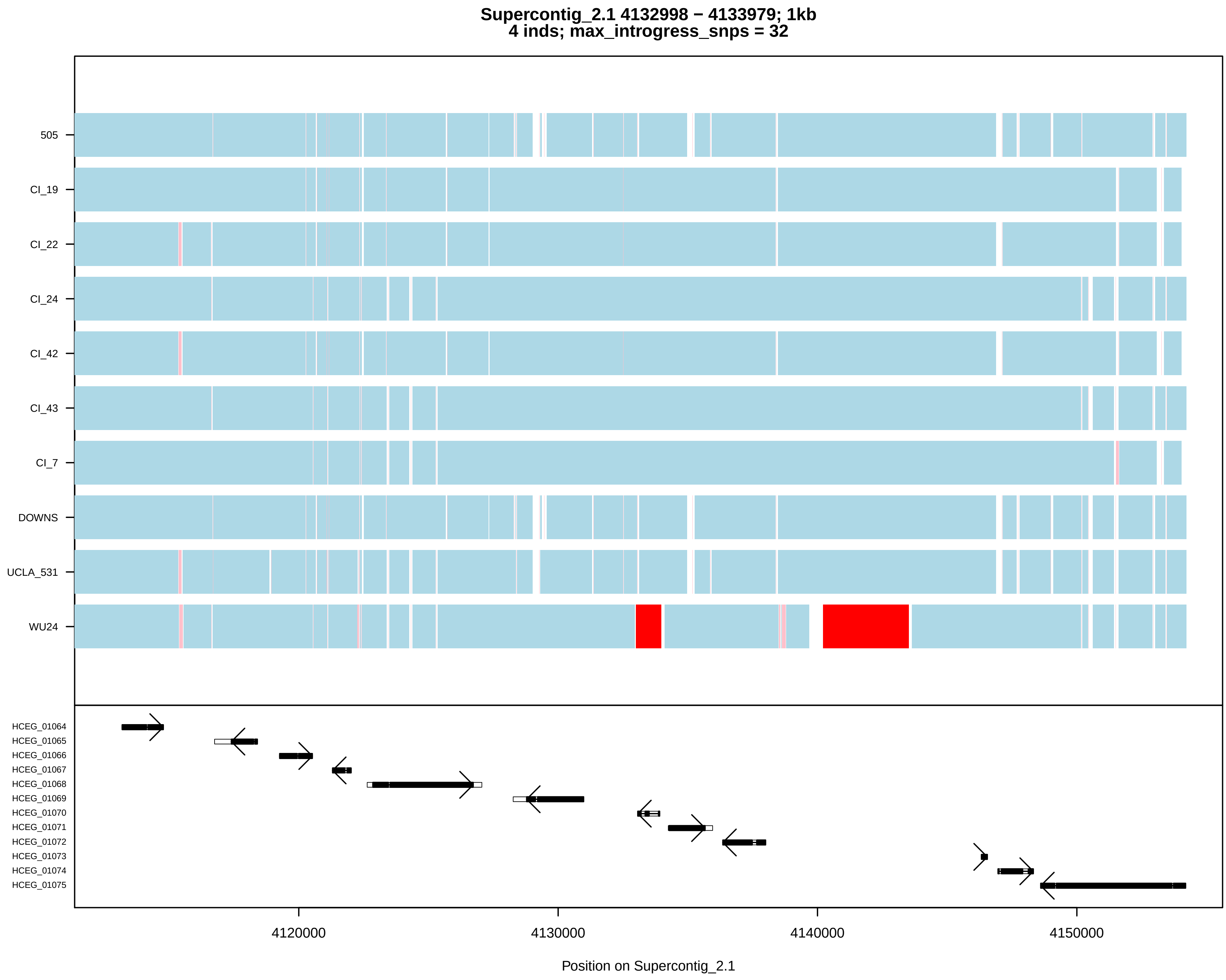

Supercontig_2.1 4132998 − 4133979; 1kb
4 inds; max_introgress_snps = 32
505
CI_19
CI_22
CI_24
CI_42
CI_43
CI_7
DOWNS
UCLA_531
WU24
HCEG_01064
HCEG_01065
HCEG_01066
HCEG_01067
HCEG_01068
HCEG_01069
HCEG_01070
HCEG_01071
HCEG_01072
HCEG_01073
HCEG_01074
HCEG_01075
4120000
4130000
4140000
4150000
Position on Supercontig_2.1

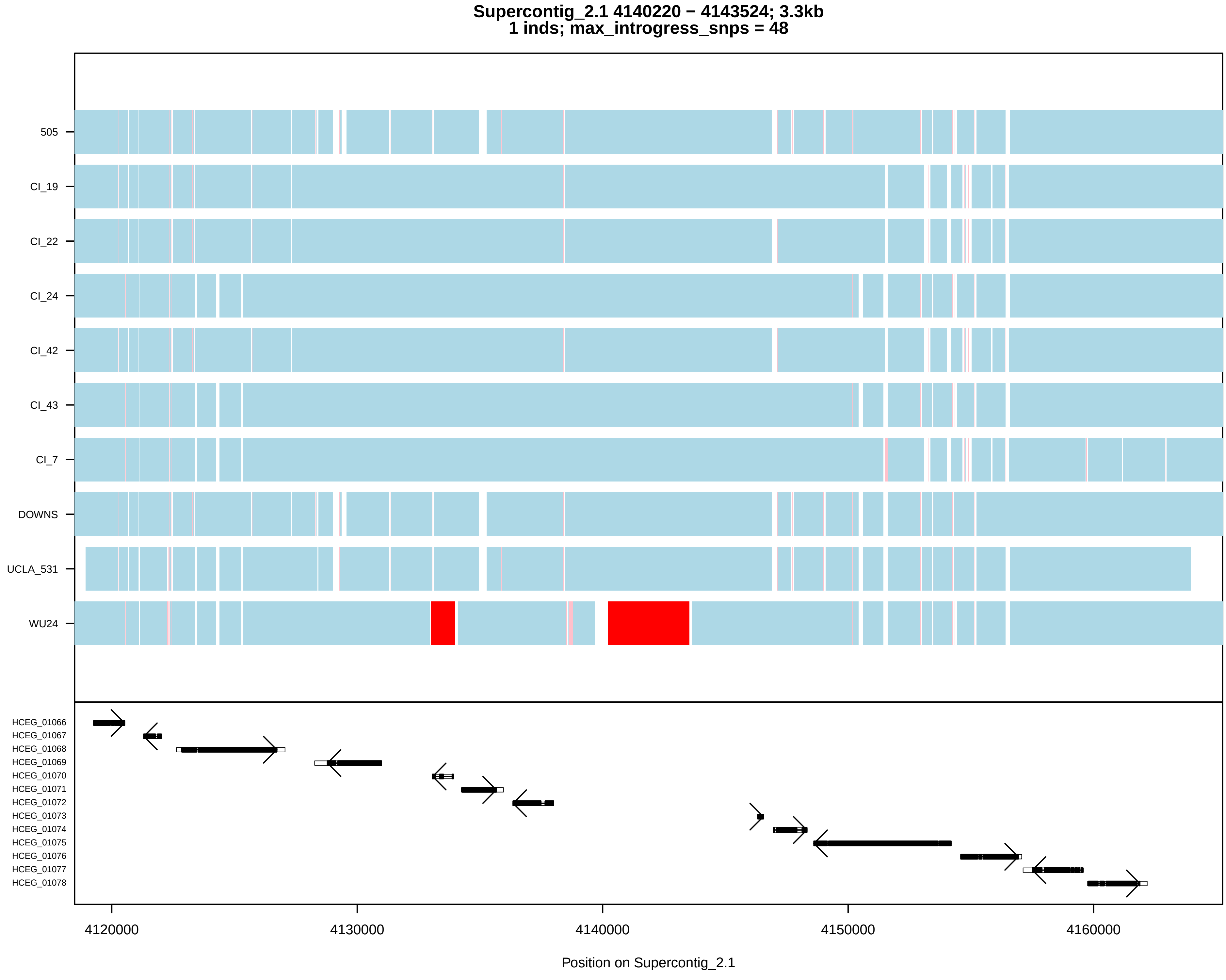

Supercontig_2.1 4140220 − 4143524; 3.3kb
1 inds; max_introgress_snps = 48
505
CI_19
CI_22
CI_24
CI_42
CI_43
CI_7
DOWNS
UCLA_531
WU24
HCEG_01066
HCEG_01067
HCEG_01068
HCEG_01069
HCEG_01070
HCEG_01071
HCEG_01072
HCEG_01073
HCEG_01074
HCEG_01075
HCEG_01076
HCEG_01077
HCEG_01078
4120000
4130000
4140000
4150000
4160000
Position on Supercontig_2.1

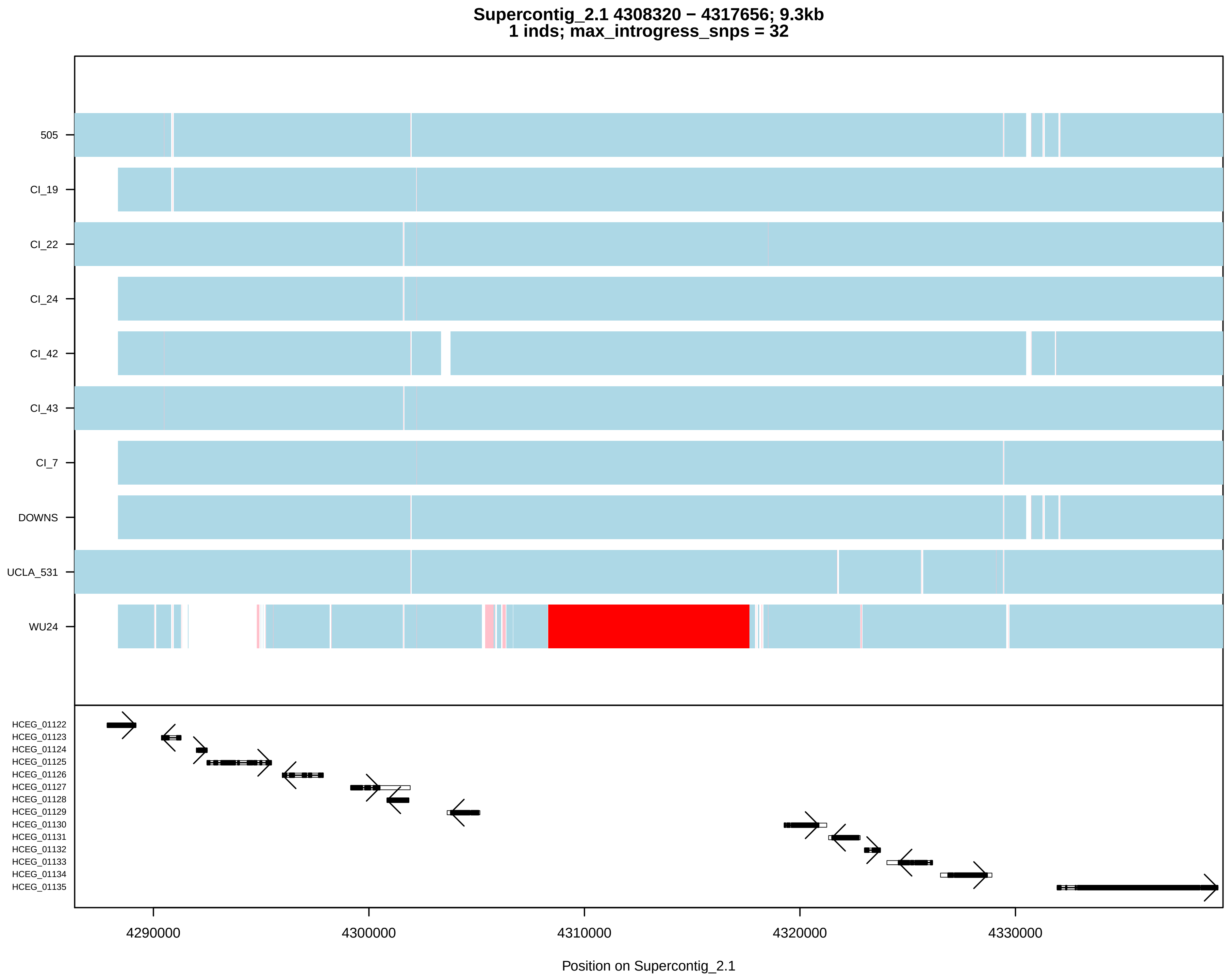
Supercontig_2.1 4308320 − 4317656; 9.3kb
1 inds; max_introgress_snps = 32
505
CI_19
CI_22
CI_24
CI_42
CI_43
CI_7
DOWNS
UCLA_531
WU24
HCEG_01122
HCEG_01123
HCEG_01124
HCEG_01125
HCEG_01126
HCEG_01127
HCEG_01128
HCEG_01129
HCEG_01130
HCEG_01131
HCEG_01132
HCEG_01133
HCEG_01134
HCEG_01135
4290000
4300000
4310000
4320000
4330000
Position on Supercontig_2.1

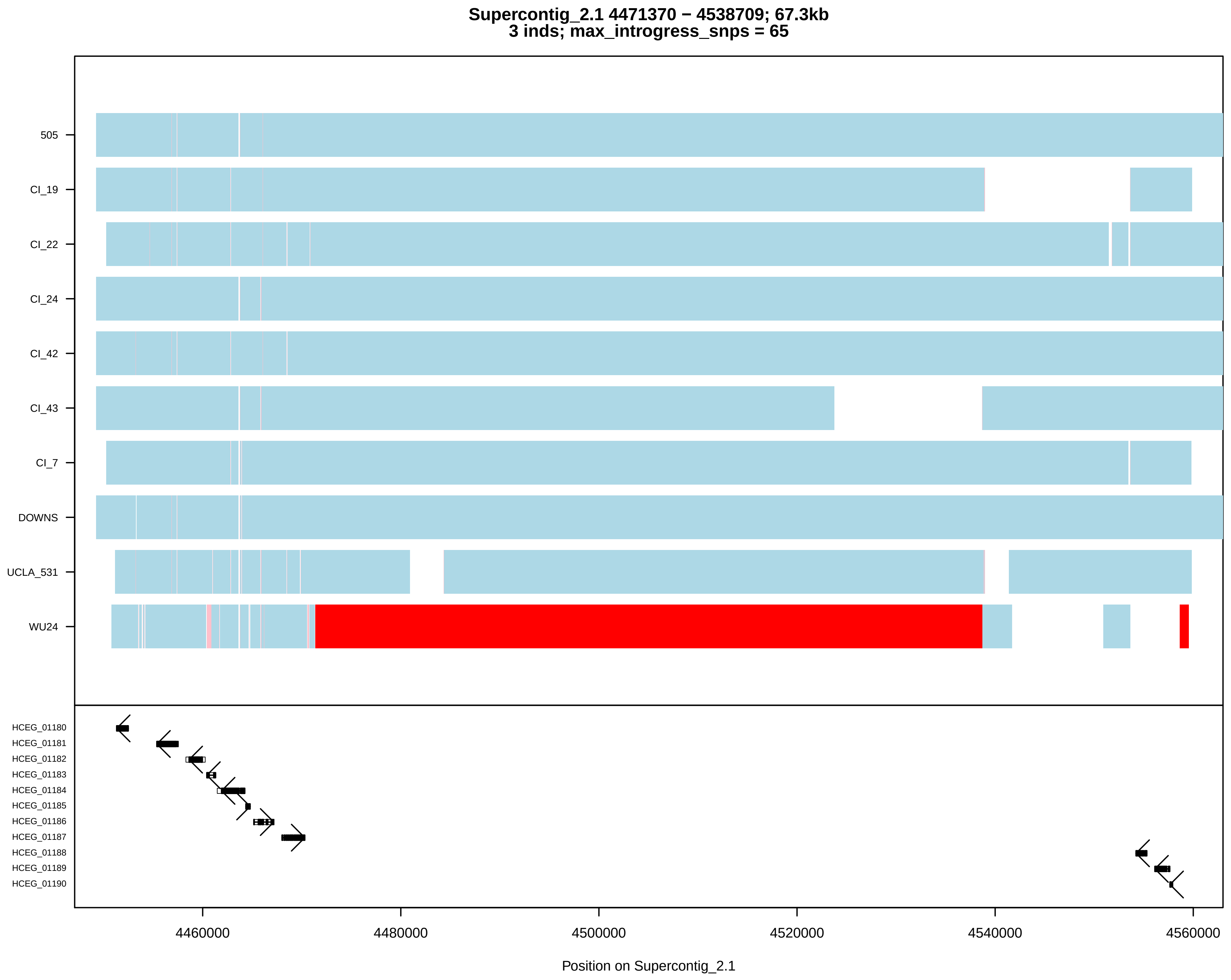

Supercontig_2.1 4471370 − 4538709; 67.3kb
3 inds; max_introgress_snps = 65
505
CI_19
CI_22
CI_24
CI_42
CI_43
CI_7
DOWNS
UCLA_531
WU24
HCEG_01180
HCEG_01181
HCEG_01182
HCEG_01183
HCEG_01184
HCEG_01185
HCEG_01186
HCEG_01187
HCEG_01188
HCEG_01189
HCEG_01190
4460000
4480000
4500000
4520000
4540000
4560000
Position on Supercontig_2.1

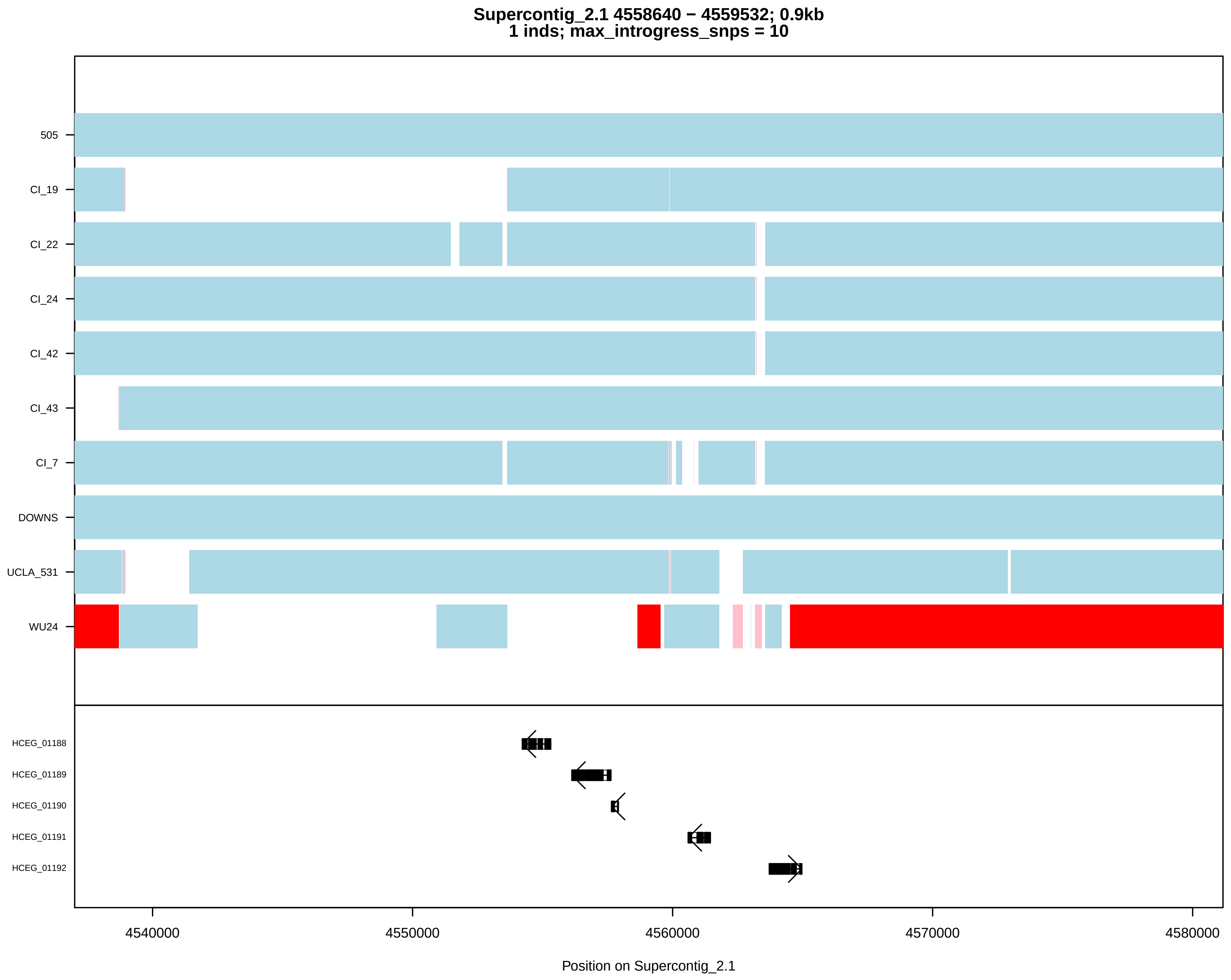

Supercontig_2.1 4558640 − 4559532; 0.9kb
1 inds; max_introgress_snps = 10
505
CI_19
CI_22
CI_24
CI_42
CI_43
CI_7
DOWNS
UCLA_531
WU24
HCEG_01188
HCEG_01189
HCEG_01190
HCEG_01191
HCEG_01192
4540000
4550000
4560000
4570000
4580000
Position on Supercontig_2.1

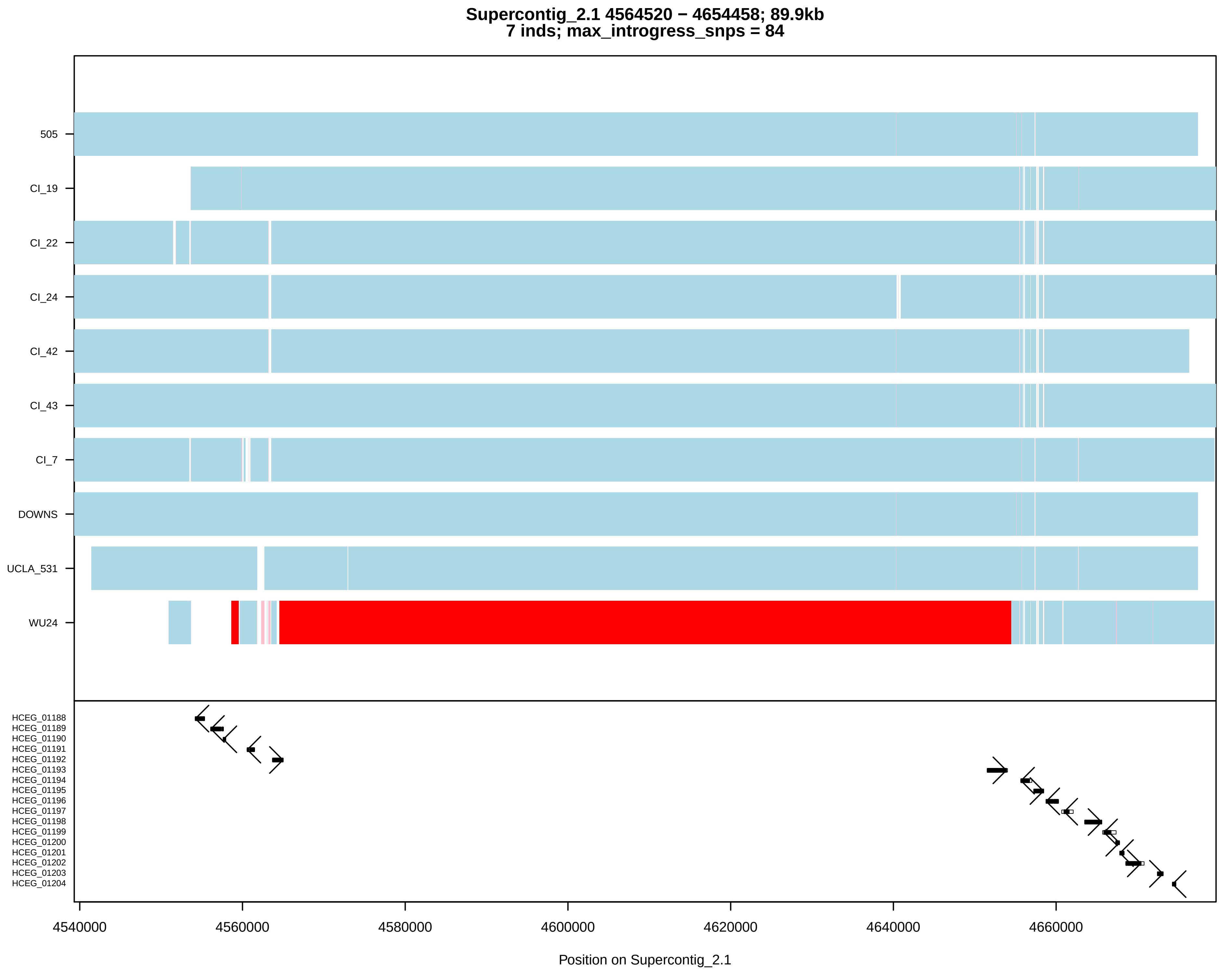
Supercontig_2.1 4564520 − 4654458; 89.9kb
7 inds; max_introgress_snps = 84
505
CI_19
CI_22
CI_24
CI_42
CI_43
CI_7
DOWNS
UCLA_531
WU24
HCEG_01188
HCEG_01189
HCEG_01190
HCEG_01191
HCEG_01192
HCEG_01193
HCEG_01194
HCEG_01195
HCEG_01196
HCEG_01197
HCEG_01198
HCEG_01199
HCEG_01200
HCEG_01201
HCEG_01202
HCEG_01203
HCEG_01204
4540000
4560000
4580000
4600000
4620000
4640000
4660000
Position on Supercontig_2.1

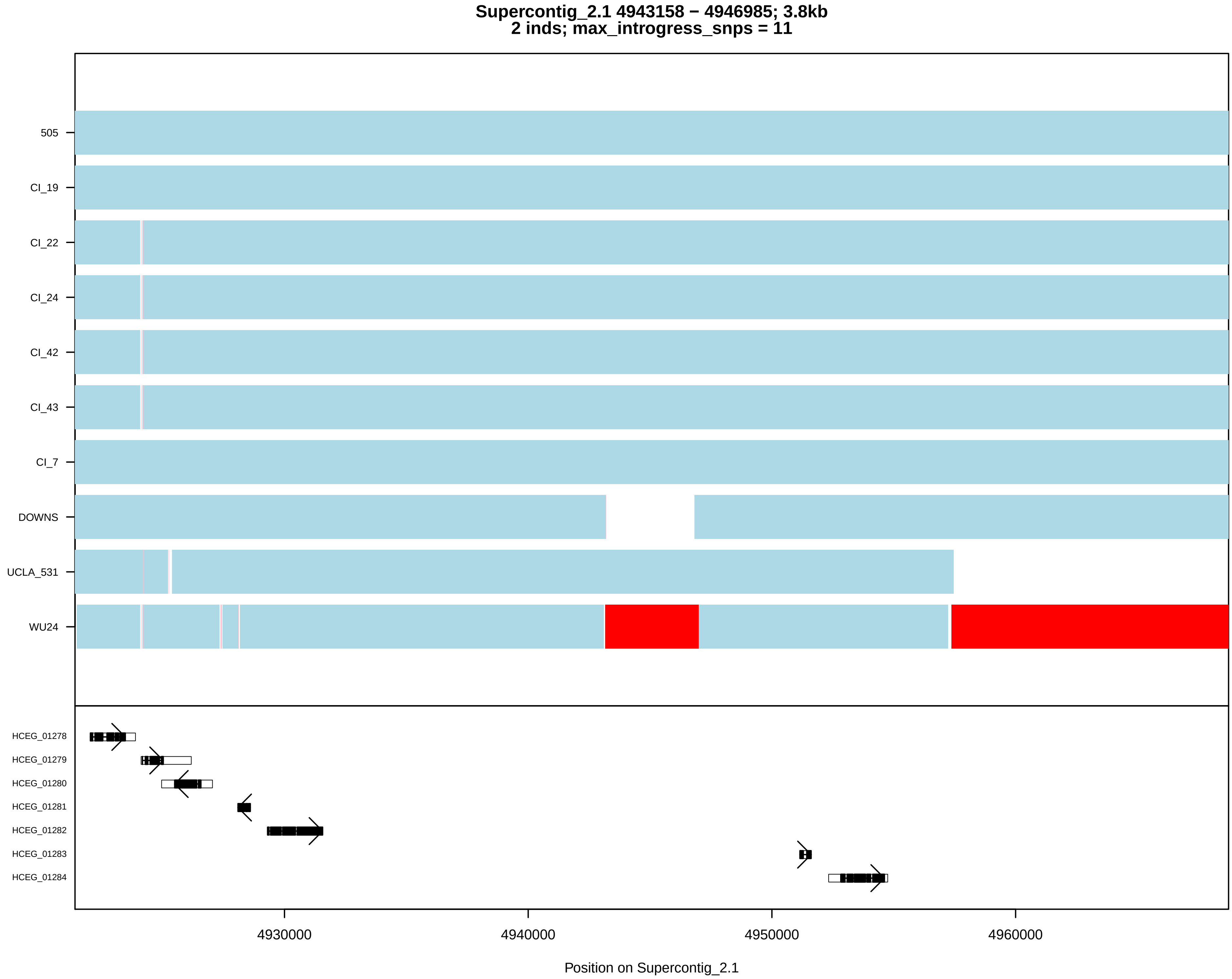

Supercontig_2.1 4943158 − 4946985; 3.8kb
2 inds; max_introgress_snps = 11
505
CI_19
CI_22
CI_24
CI_42
CI_43
CI_7
DOWNS
UCLA_531
WU24
HCEG_01278
HCEG_01279
HCEG_01280
HCEG_01281
HCEG_01282
HCEG_01283
HCEG_01284
4930000
4940000
4950000
4960000
Position on Supercontig_2.1

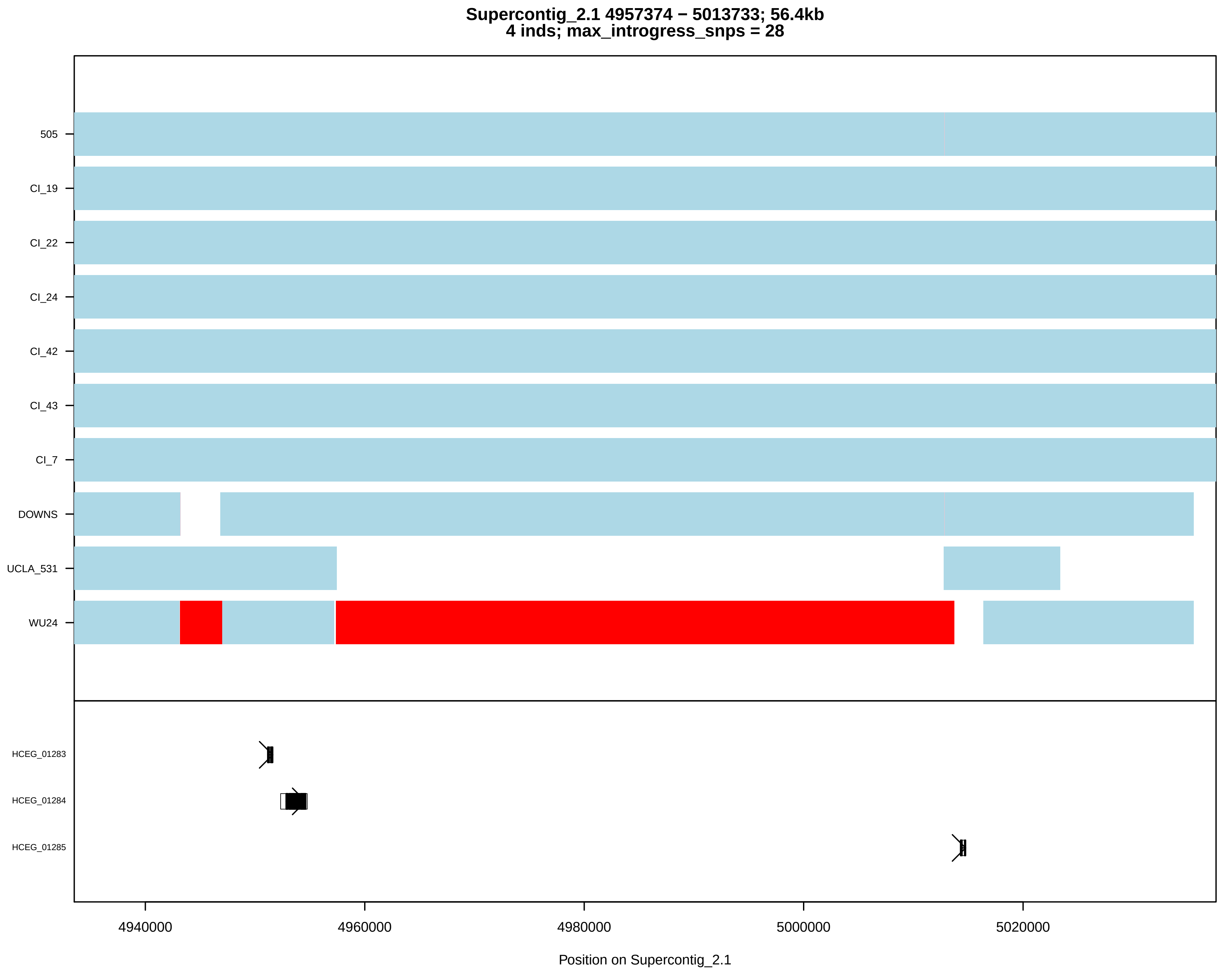

Supercontig_2.1 4957374 − 5013733; 56.4kb
4 inds; max_introgress_snps = 28
505
CI_19
CI_22
CI_24
CI_42
CI_43
CI_7
DOWNS
UCLA_531
WU24
HCEG_01283
HCEG_01284
HCEG_01285
4940000
4960000
4980000
5000000
5020000
Position on Supercontig_2.1

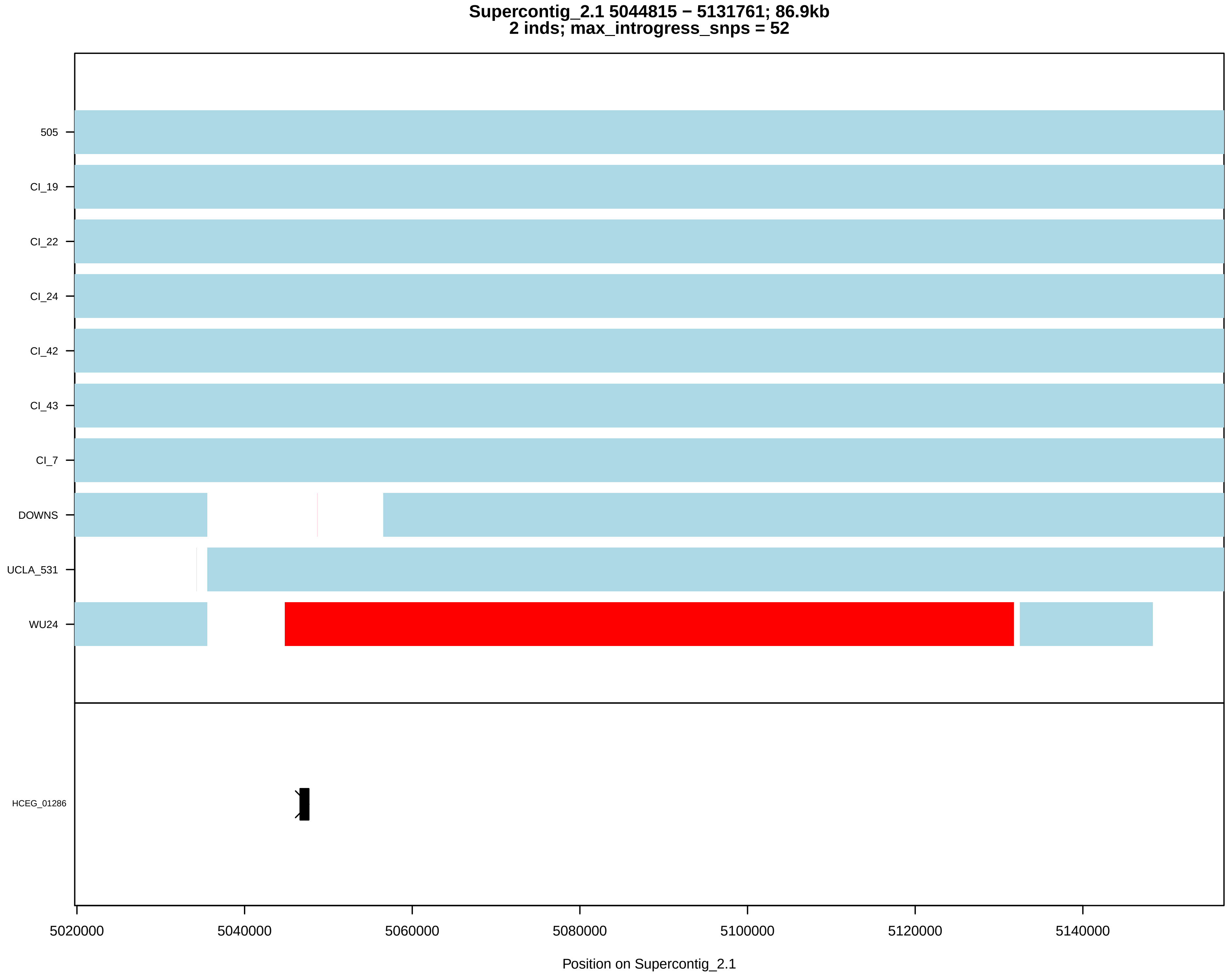

Supercontig_2.1 5044815 − 5131761; 86.9kb
2 inds; max_introgress_snps = 52
505
CI_19
CI_22
CI_24
CI_42
CI_43
CI_7
DOWNS
UCLA_531
WU24
HCEG_01286
5020000
5040000
5060000
5080000
5100000
5120000
5140000
Position on Supercontig_2.1

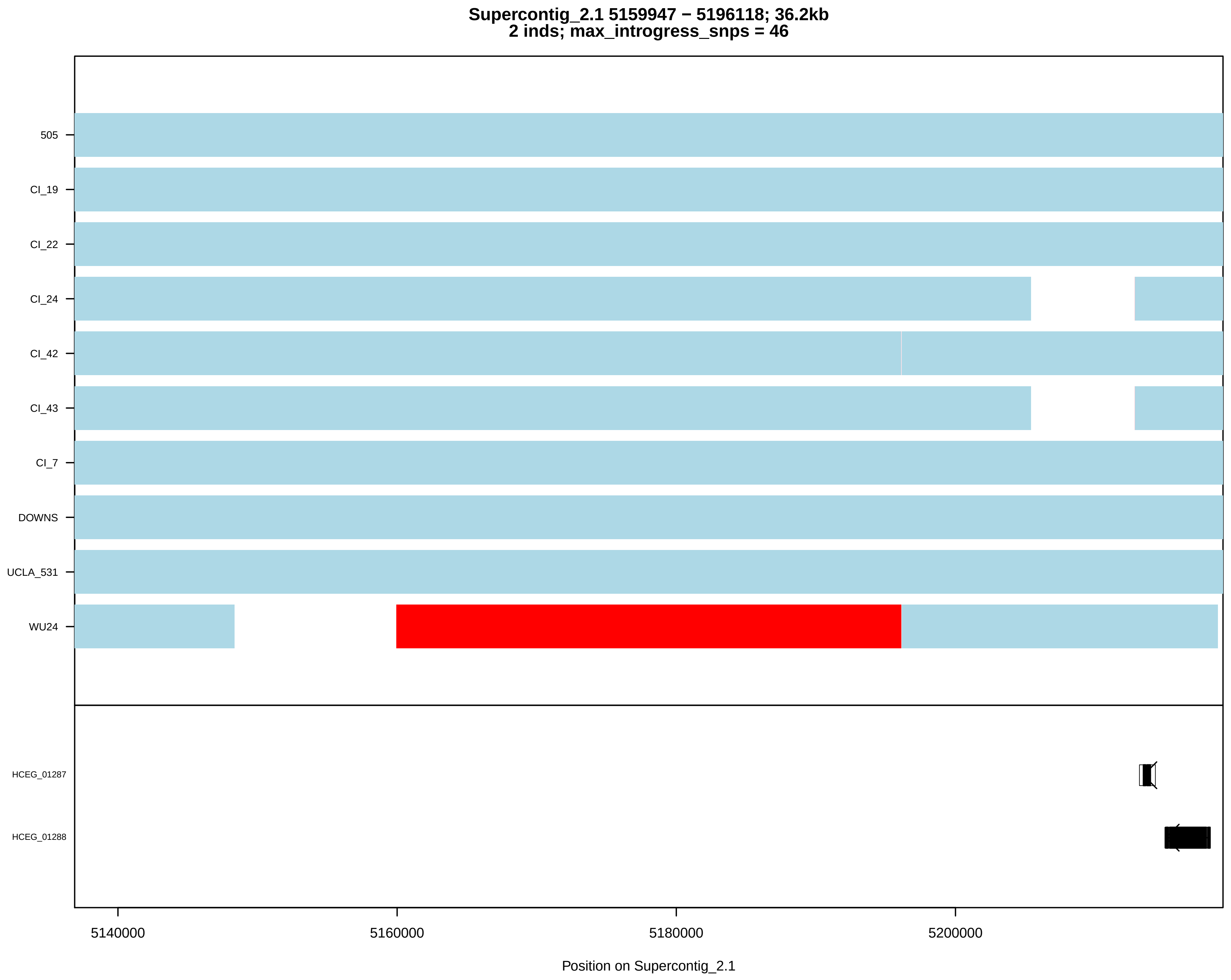

Supercontig_2.1 5159947 − 5196118; 36.2kb
2 inds; max_introgress_snps = 46
505
CI_19
CI_22
CI_24
CI_42
CI_43
CI_7
DOWNS
UCLA_531
WU24
HCEG_01287
HCEG_01288
5140000
5160000
5180000
5200000
Position on Supercontig_2.1

Supercontig_2.1 5252303 − 5440198; 187.9kb
1 inds; max_introgress_snps = 76

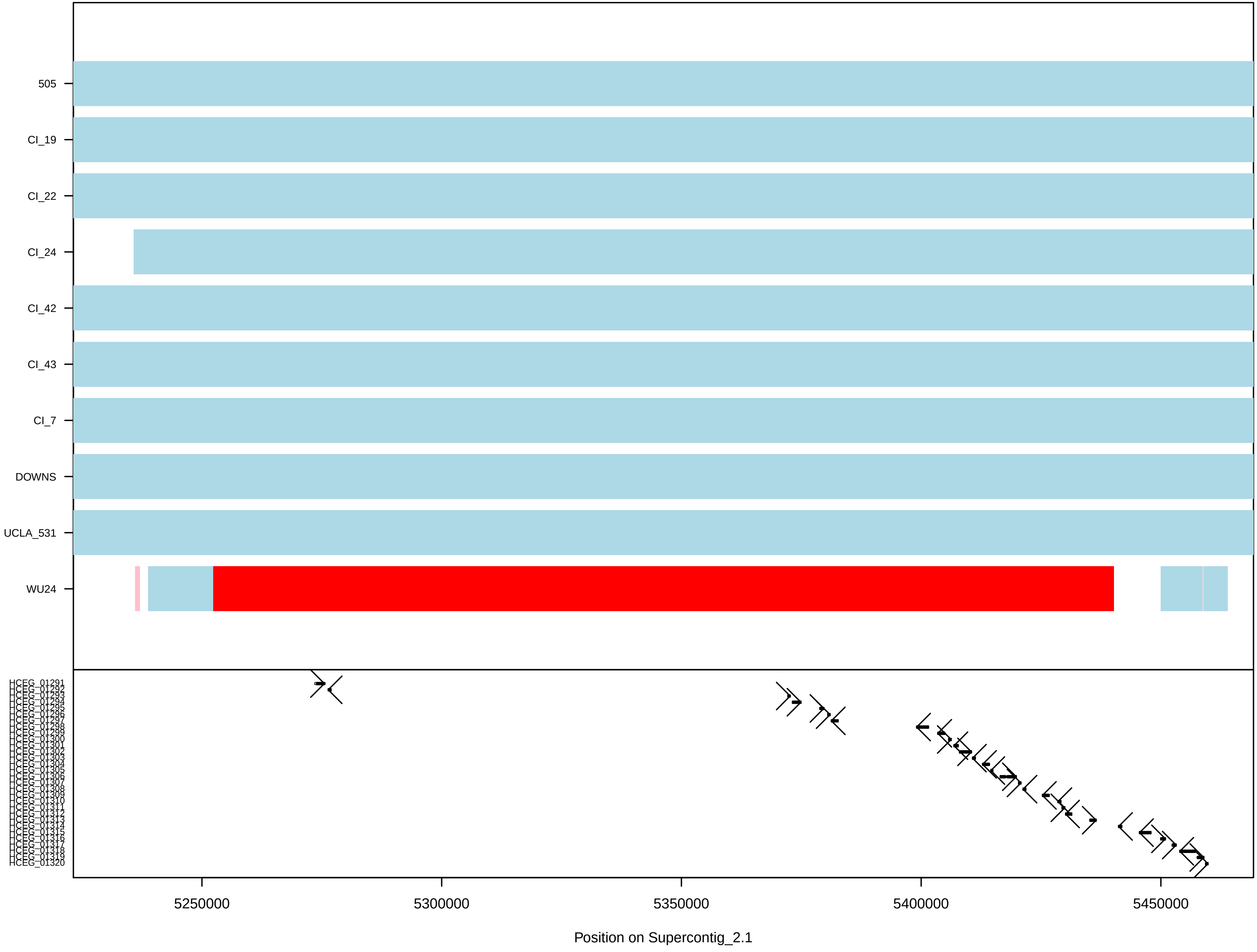

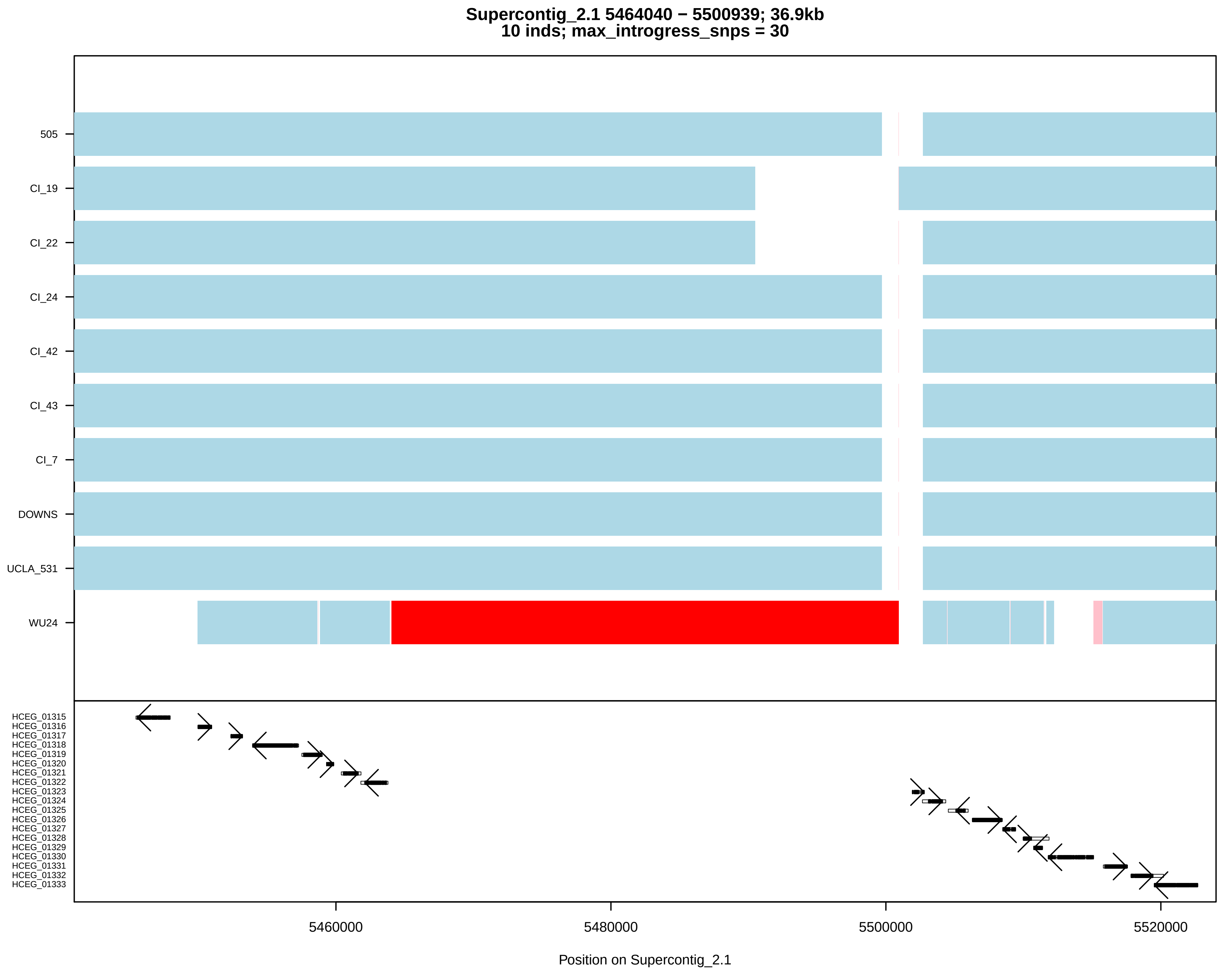
Supercontig_2.1 5464040 − 5500939; 36.9kb
10 inds; max_introgress_snps = 30
505
CI_19
CI_22
CI_24
CI_42
CI_43
CI_7
DOWNS
UCLA_531
WU24
HCEG_01315
HCEG_01316
HCEG_01317
HCEG_01318
HCEG_01319
HCEG_01320
HCEG_01321
HCEG_01322
HCEG_01323
HCEG_01324
HCEG_01325
HCEG_01326
HCEG_01327
HCEG_01328
HCEG_01329
HCEG_01330
HCEG_01331
HCEG_01332
HCEG_01333
5460000
5480000
5500000
5520000
Position on Supercontig_2.1

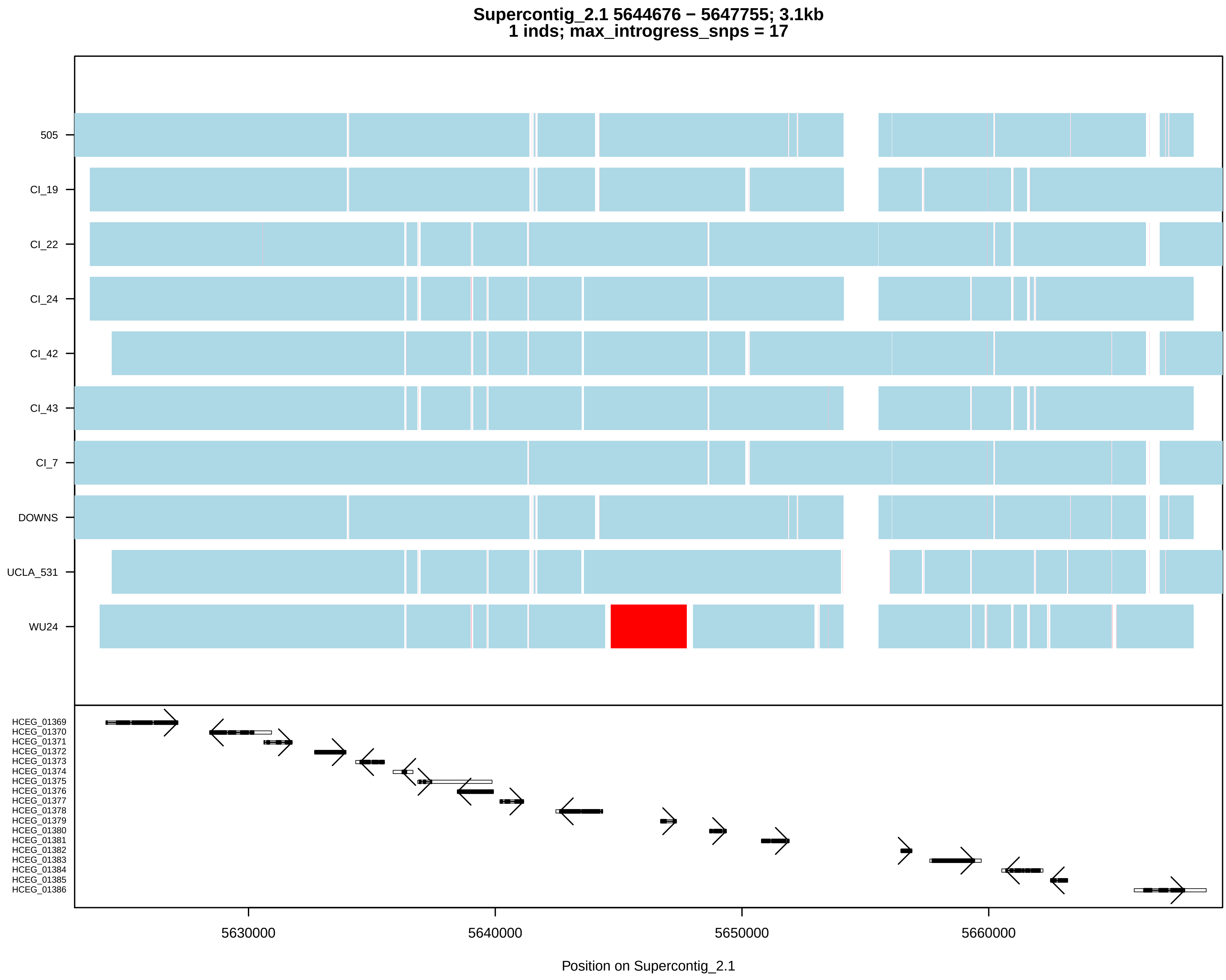
Supercontig_2.1 5644676 − 5647755; 3.1kb
1 inds; max_introgress_snps = 17
505
CI_19
CI_22
CI_24
CI_42
CI_43
CI_7
DOWNS
UCLA_531
WU24
HCEG_01369
HCEG_01370
HCEG_01371
HCEG_01372
HCEG_01373
HCEG_01374
HCEG_01375
HCEG_01376
HCEG_01377
HCEG_01378
HCEG_01379
HCEG_01380
HCEG_01381
HCEG_01382
HCEG_01383
HCEG_01384
HCEG_01385
HCEG_01386
5630000
5640000
5650000
5660000
Position on Supercontig_2.1

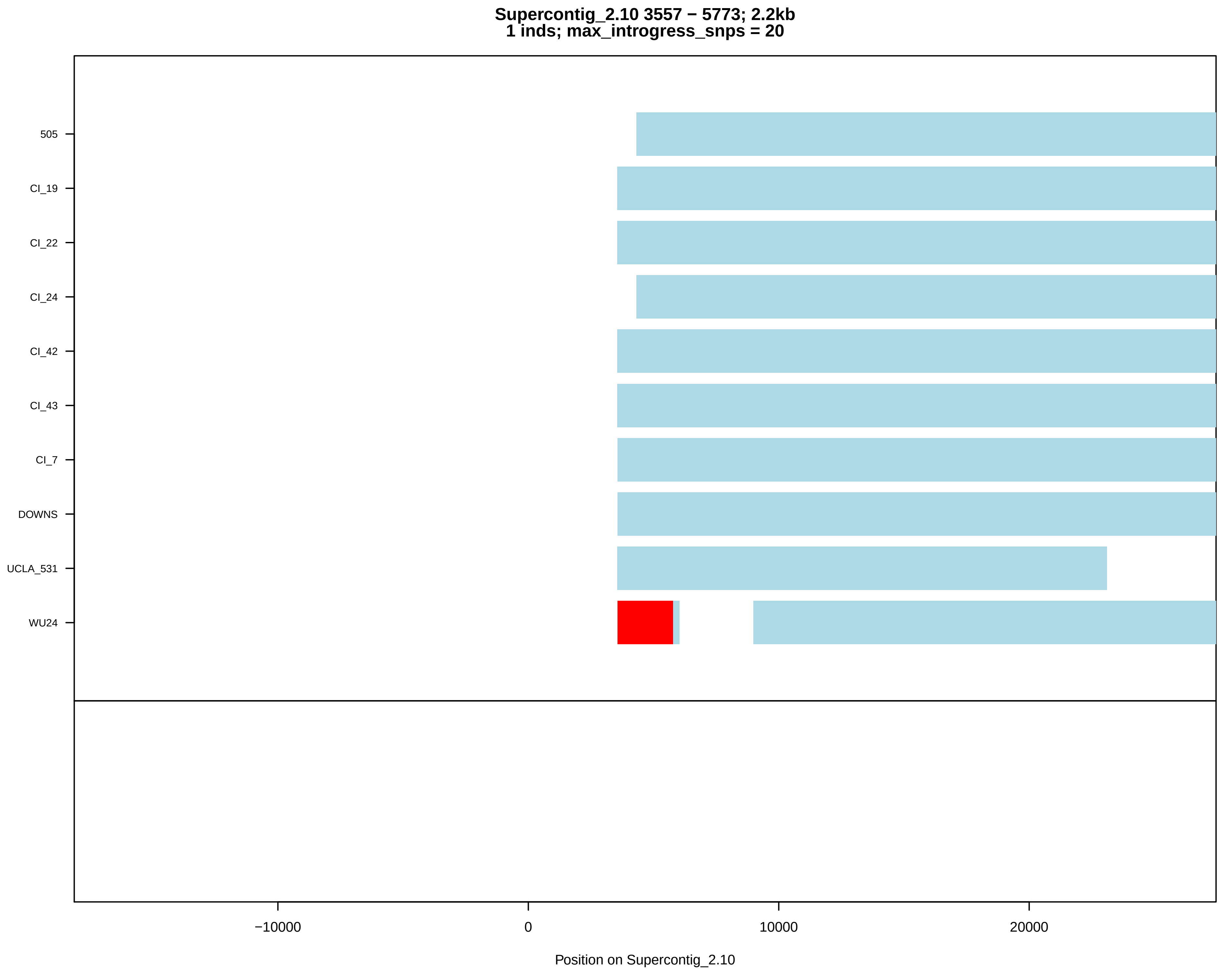

Supercontig_2.10 3557 − 5773; 2.2kb
1 inds; max_introgress_snps = 20
505
CI_19
CI_22
CI_24
CI_42
CI_43
CI_7
DOWNS
UCLA_531
WU24
−10000
0
10000
20000
Position on Supercontig_2.10

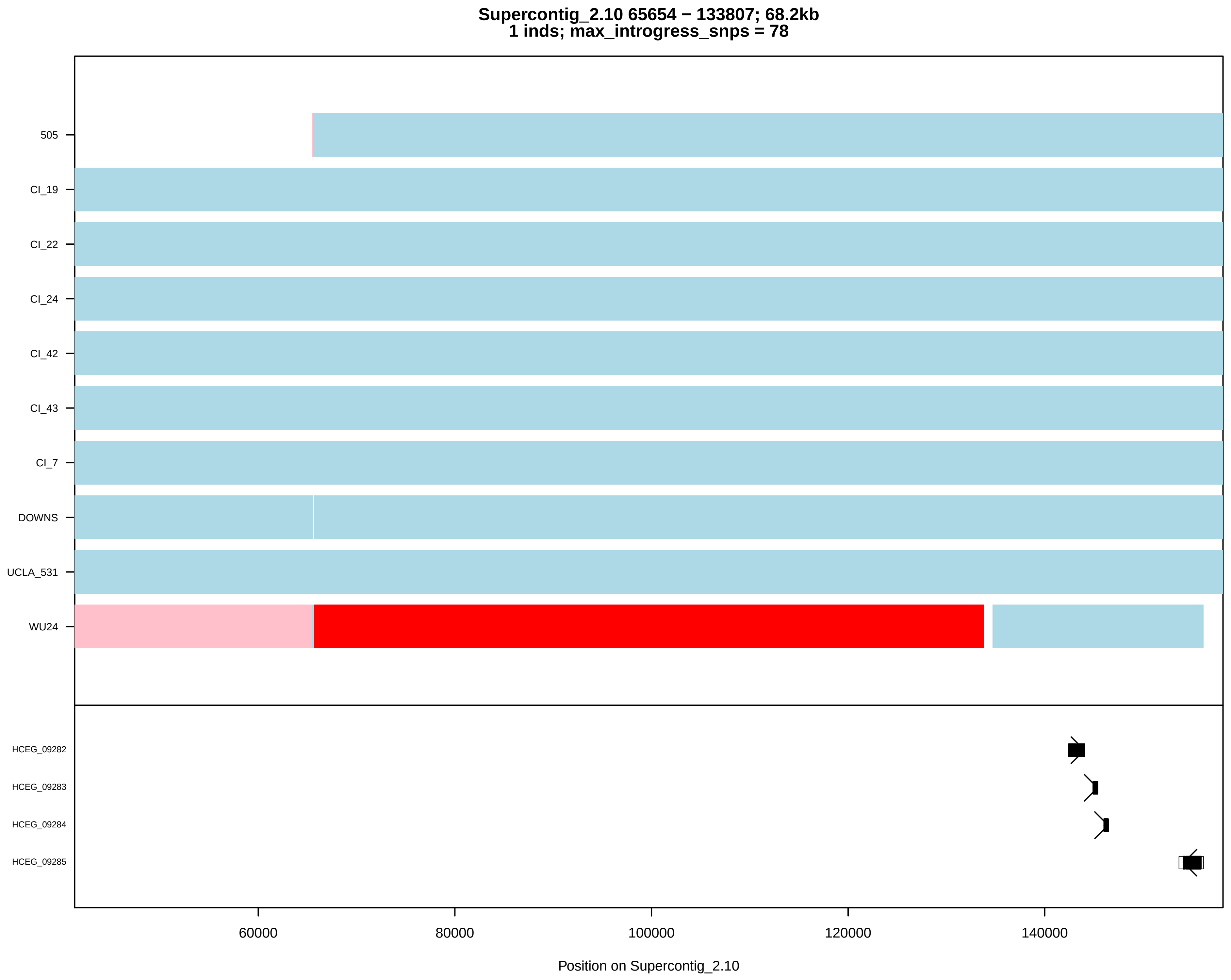

Supercontig_2.10 65654 − 133807; 68.2kb
1 inds; max_introgress_snps = 78
505
CI_19
CI_22
CI_24
CI_42
CI_43
CI_7
DOWNS
UCLA_531
WU24
HCEG_09282
HCEG_09283
HCEG_09284
HCEG_09285
60000
80000
100000
120000
140000
Position on Supercontig_2.10

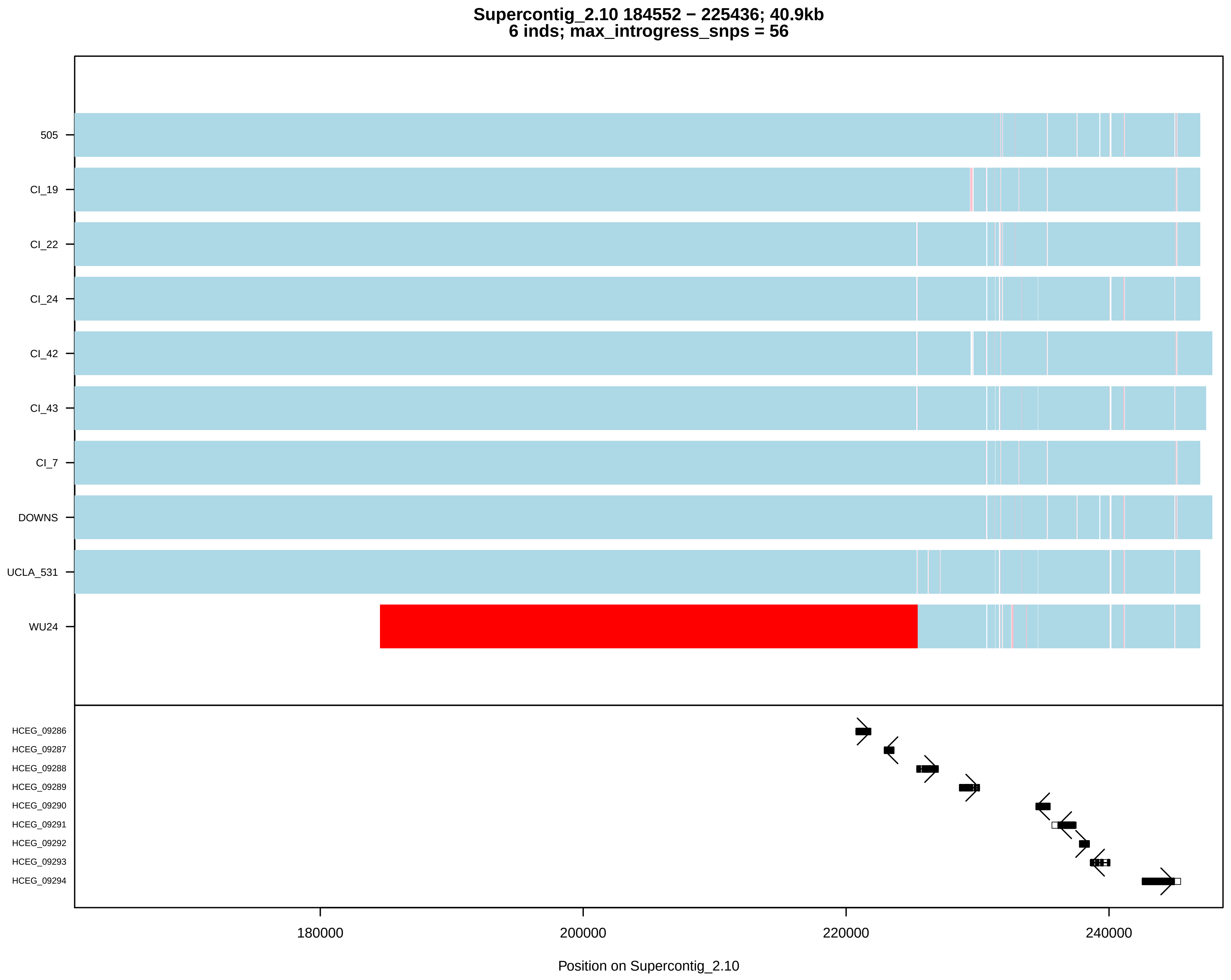
Supercontig_2.10 184552 − 225436; 40.9kb
6 inds; max_introgress_snps = 56
505
CI_19
CI_22
CI_24
CI_42
CI_43
CI_7
DOWNS
UCLA_531
WU24
HCEG_09286
HCEG_09287
HCEG_09288
HCEG_09289
HCEG_09290
HCEG_09291
HCEG_09292
HCEG_09293
HCEG_09294
180000
200000
220000
240000
Position on Supercontig_2.10

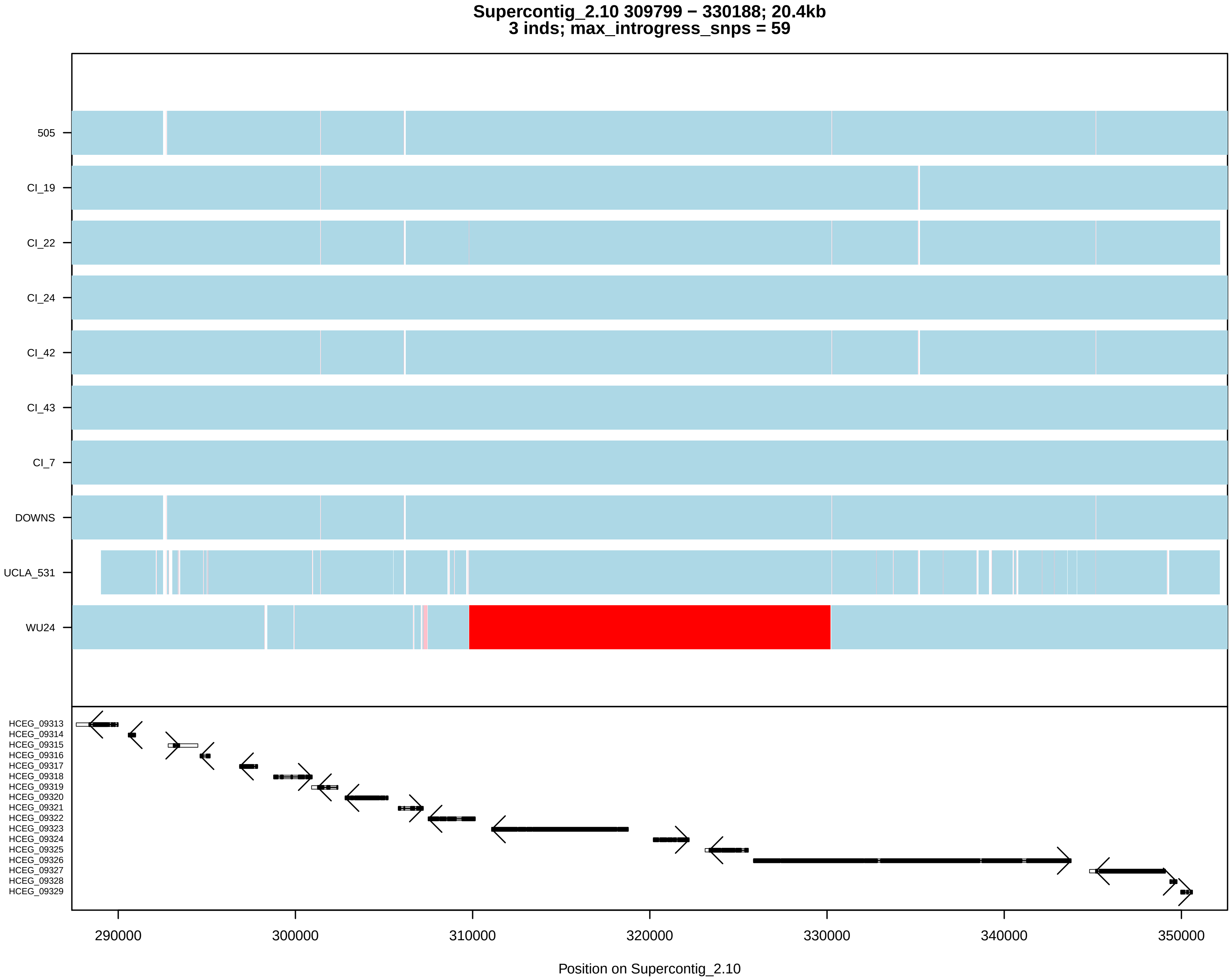
Supercontig_2.10 309799 − 330188; 20.4kb
3 inds; max_introgress_snps = 59
505
CI_19
CI_22
CI_24
CI_42
CI_43
CI_7
DOWNS
UCLA_531
WU24
HCEG_09313
HCEG_09314
HCEG_09315
HCEG_09316
HCEG_09317
HCEG_09318
HCEG_09319
HCEG_09320
HCEG_09321
HCEG_09322
HCEG_09323
HCEG_09324
HCEG_09325
HCEG_09326
HCEG_09327
HCEG_09328
HCEG_09329
290000
300000
310000
320000
330000
340000
350000
Position on Supercontig_2.10

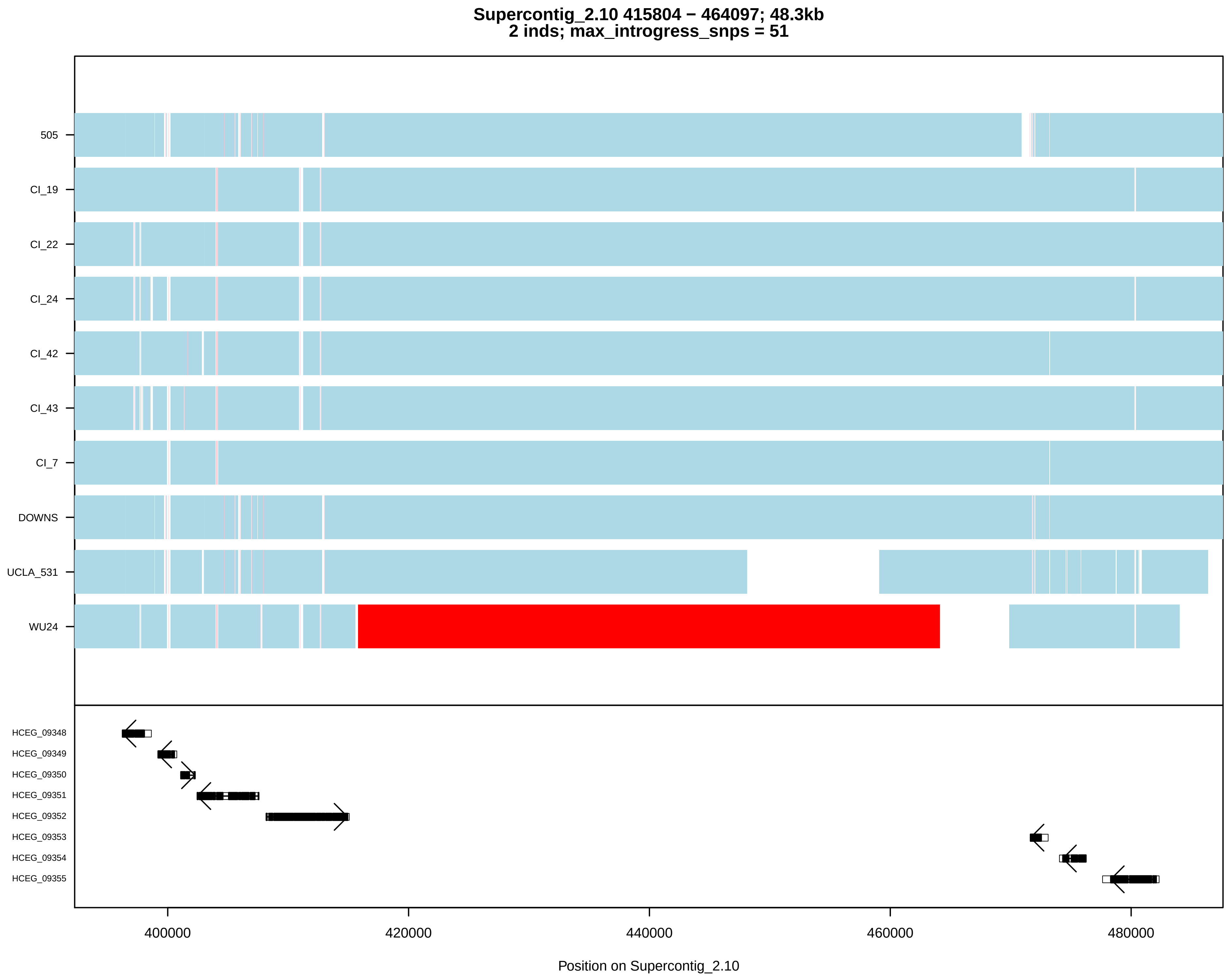
Supercontig_2.10 415804 − 464097; 48.3kb
2 inds; max_introgress_snps = 51
505
CI_19
CI_22
CI_24
CI_42
CI_43
CI_7
DOWNS
UCLA_531
WU24
HCEG_09348
HCEG_09349
HCEG_09350
HCEG_09351
HCEG_09352
HCEG_09353
HCEG_09354
HCEG_09355
400000
420000
440000
460000
480000
Position on Supercontig_2.10

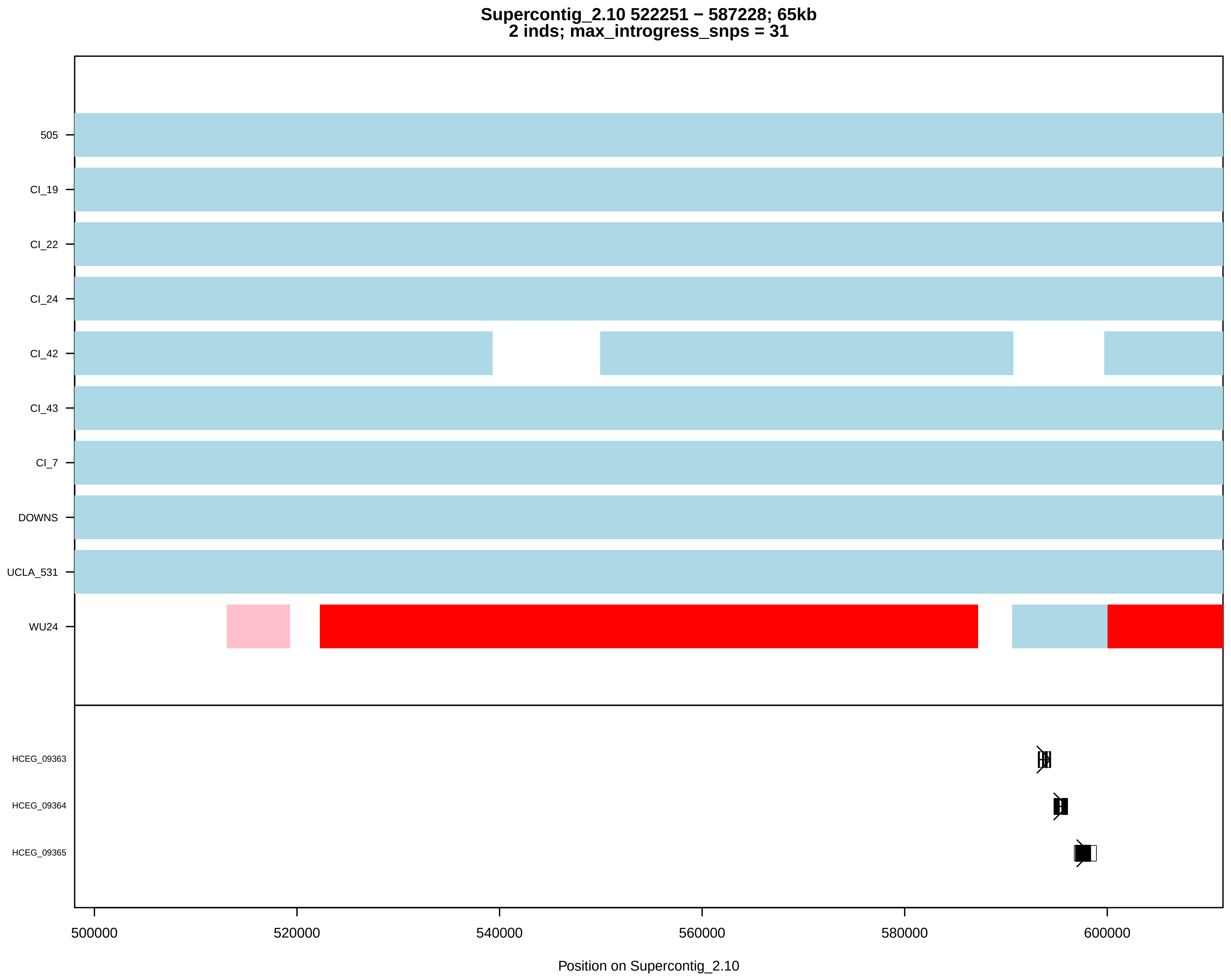
Supercontig_2.10 522251 − 587228; 65kb
2 inds; max_introgress_snps = 31
505
CI_19
CI_22
CI_24
CI_42
CI_43
CI_7
DOWNS
UCLA_531
WU24
HCEG_09363
HCEG_09364
HCEG_09365
500000
520000
540000
560000
580000
600000
Position on Supercontig_2.10

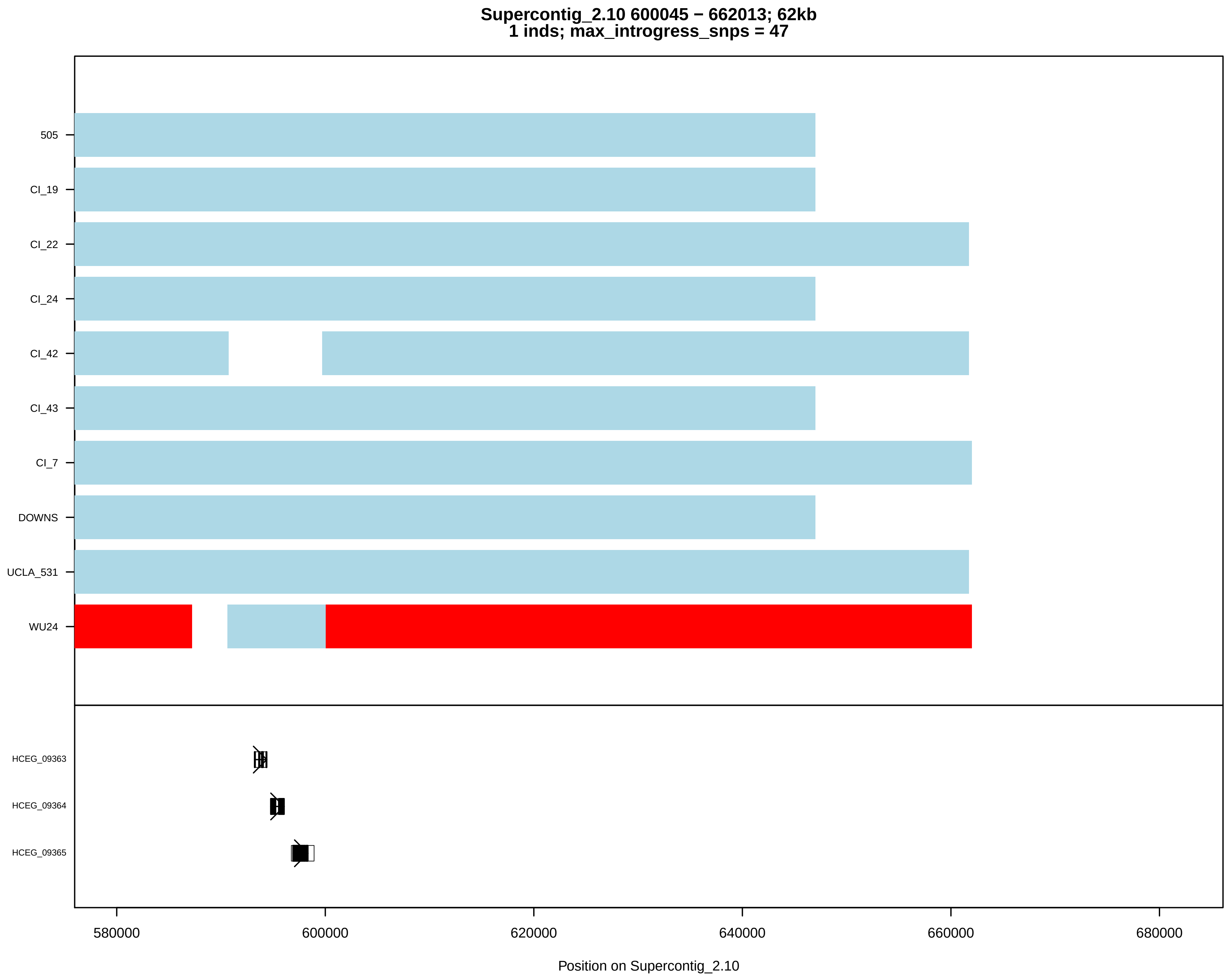

Supercontig_2.10 600045 − 662013; 62kb
1 inds; max_introgress_snps = 47
505
CI_19
CI_22
CI_24
CI_42
CI_43
CI_7
DOWNS
UCLA_531
WU24
HCEG_09363
HCEG_09364
HCEG_09365
580000
600000
620000
640000
660000
680000
Position on Supercontig_2.10

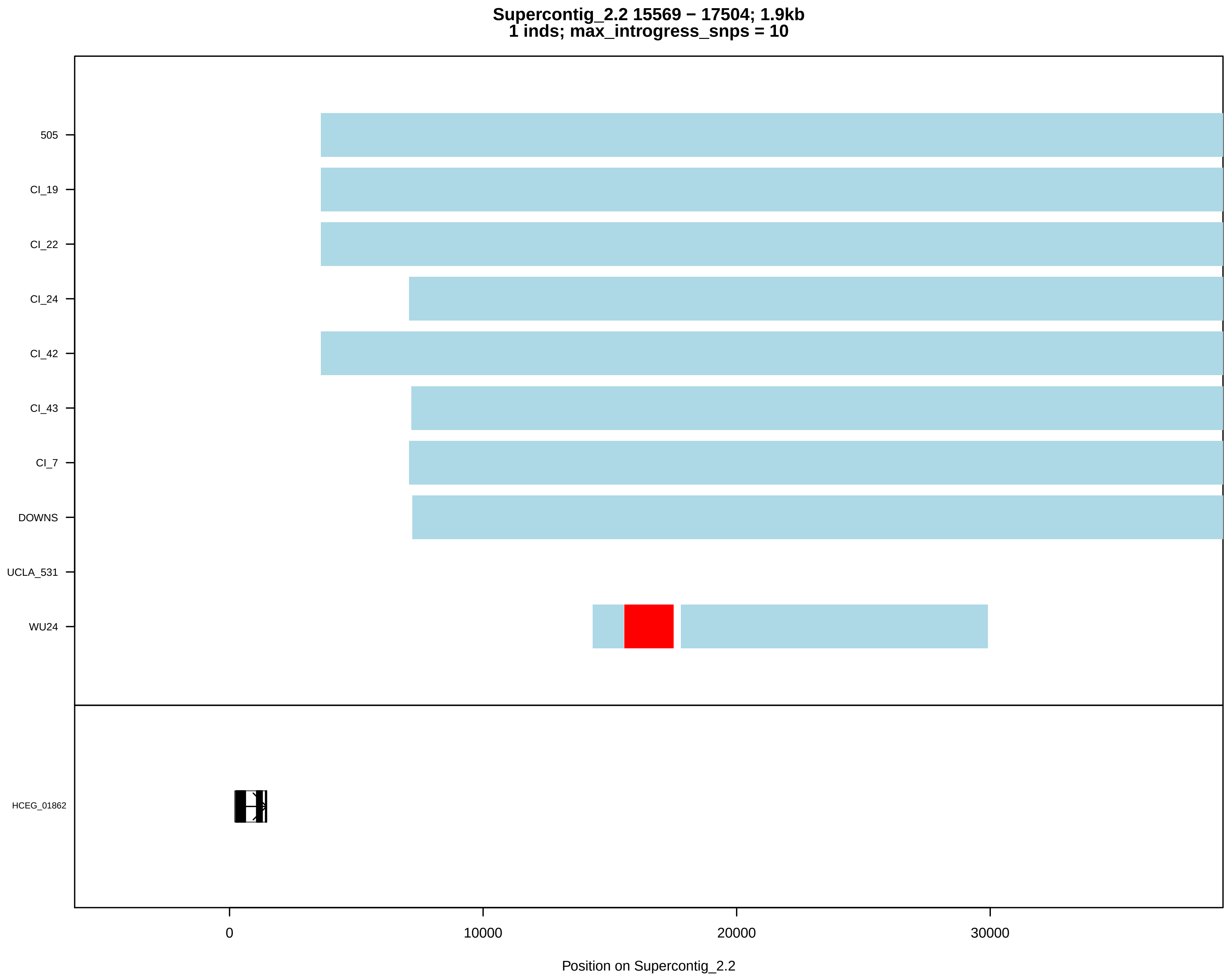
Supercontig_2.2 15569 − 17504; 1.9kb
1 inds; max_introgress_snps = 10
505
CI_19
CI_22
CI_24
CI_42
CI_43
CI_7
DOWNS
UCLA_531
WU24
HCEG_01862
0
10000
20000
30000
Position on Supercontig_2.2

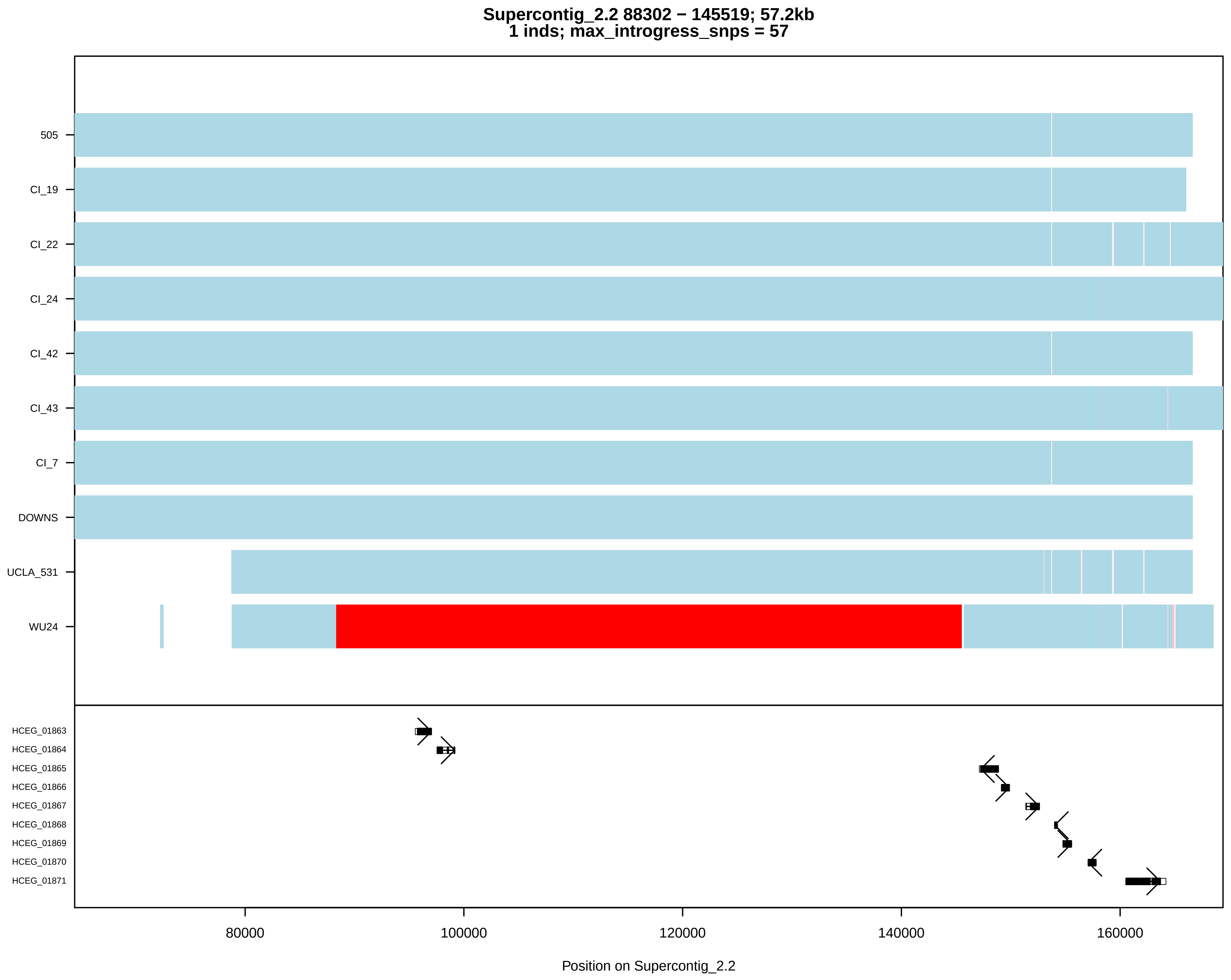

Supercontig_2.2 88302 − 145519; 57.2kb
1 inds; max_introgress_snps = 57
505
CI_19
CI_22
CI_24
CI_42
CI_43
CI_7
DOWNS
UCLA_531
WU24
HCEG_01863
HCEG_01864
HCEG_01865
HCEG_01866
HCEG_01867
HCEG_01868
HCEG_01869
HCEG_01870
HCEG_01871
80000
100000
120000
140000
160000
Position on Supercontig_2.2

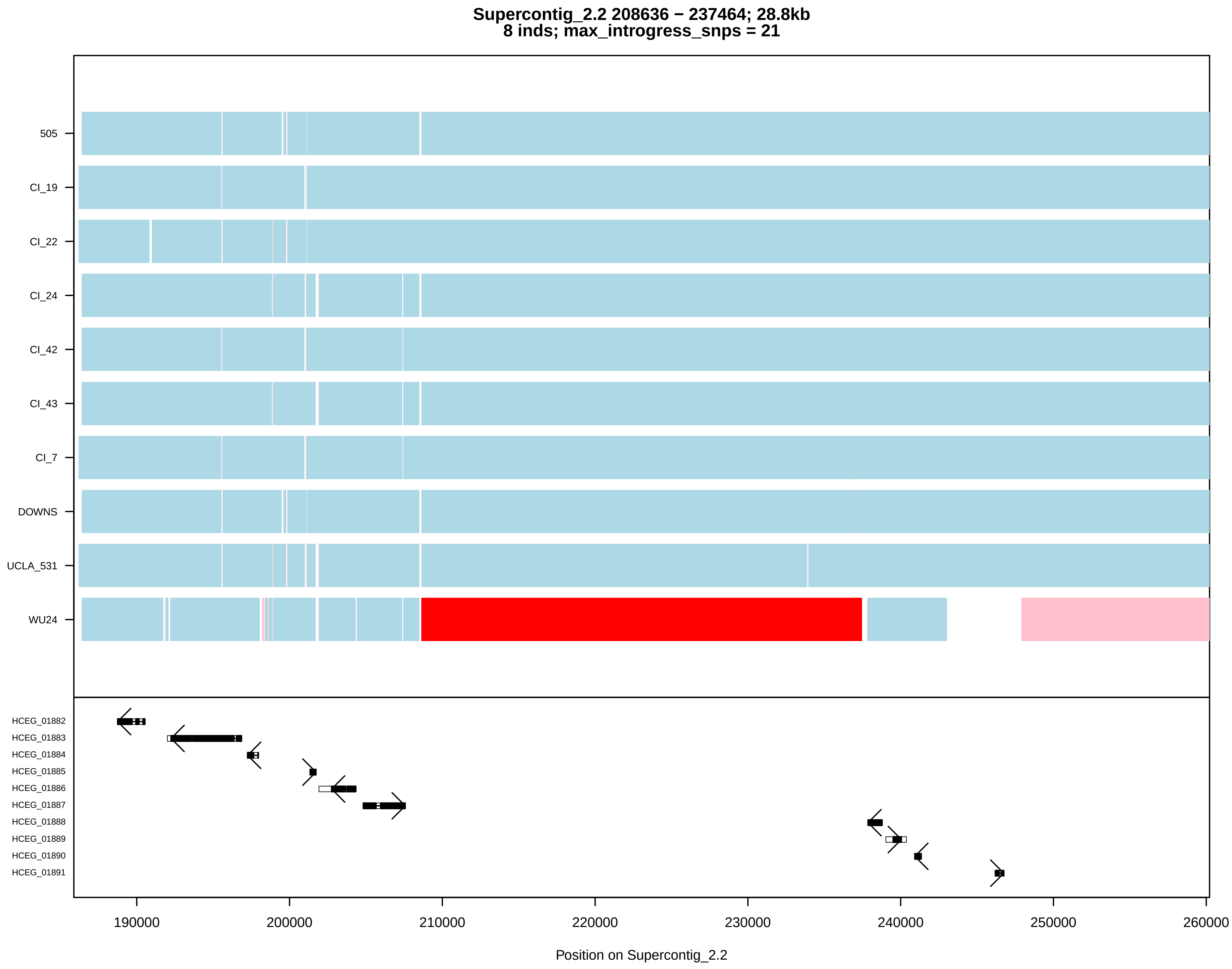

Supercontig_2.2 208636 − 237464; 28.8kb
8 inds; max_introgress_snps = 21
505
CI_19
CI_22
CI_24
CI_42
CI_43
CI_7
DOWNS
UCLA_531
WU24
HCEG_01882
HCEG_01883
HCEG_01884
HCEG_01885
HCEG_01886
HCEG_01887
HCEG_01888
HCEG_01889
HCEG_01890
HCEG_01891
190000
200000
210000
220000
230000
240000
250000
260000
Position on Supercontig_2.2

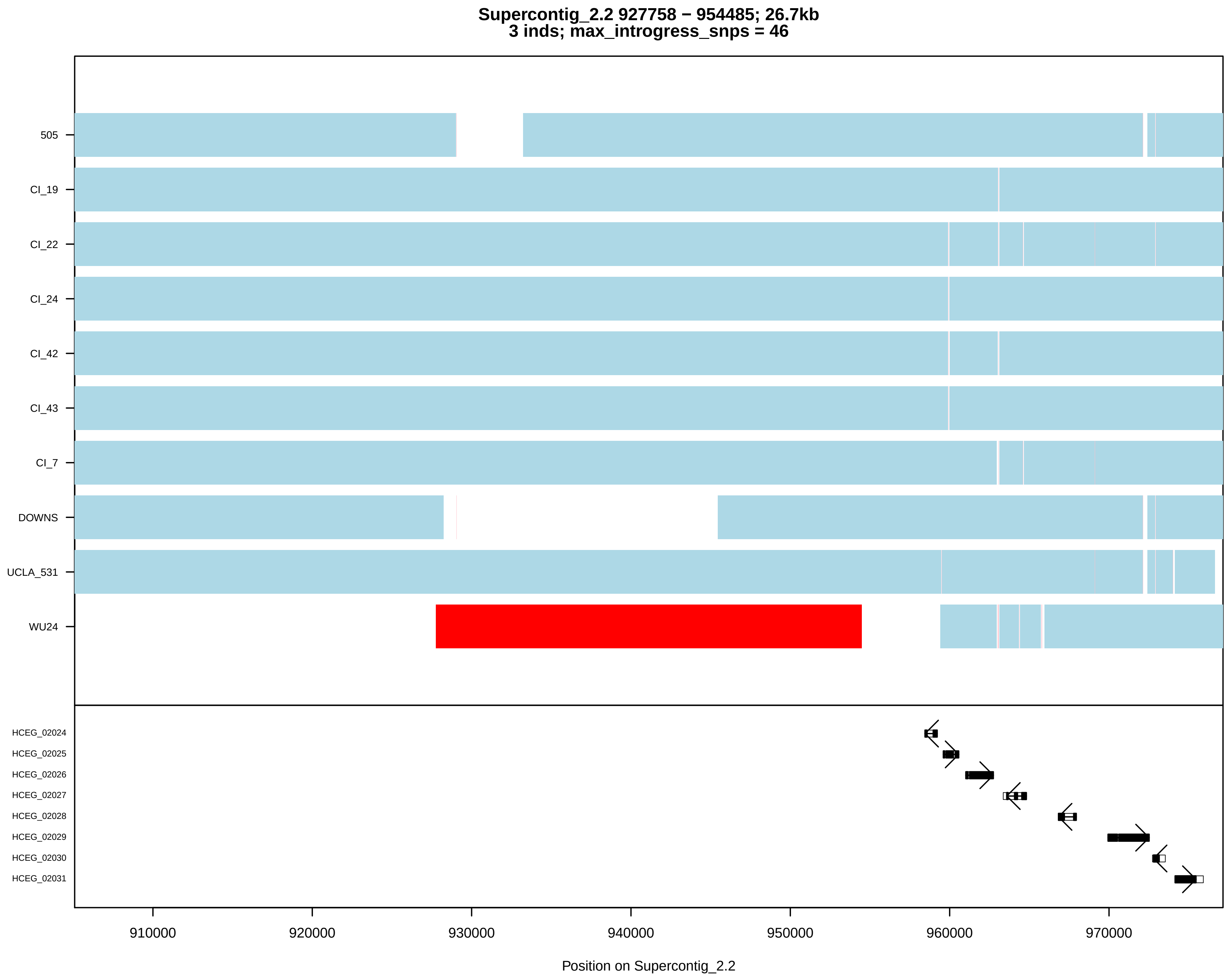

Supercontig_2.2 927758 − 954485; 26.7kb
3 inds; max_introgress_snps = 46
505
CI_19
CI_22
CI_24
CI_42
CI_43
CI_7
DOWNS
UCLA_531
WU24
HCEG_02024
HCEG_02025
HCEG_02026
HCEG_02027
HCEG_02028
HCEG_02029
HCEG_02030
HCEG_02031
910000
920000
930000
940000
950000
960000
970000
Position on Supercontig_2.2

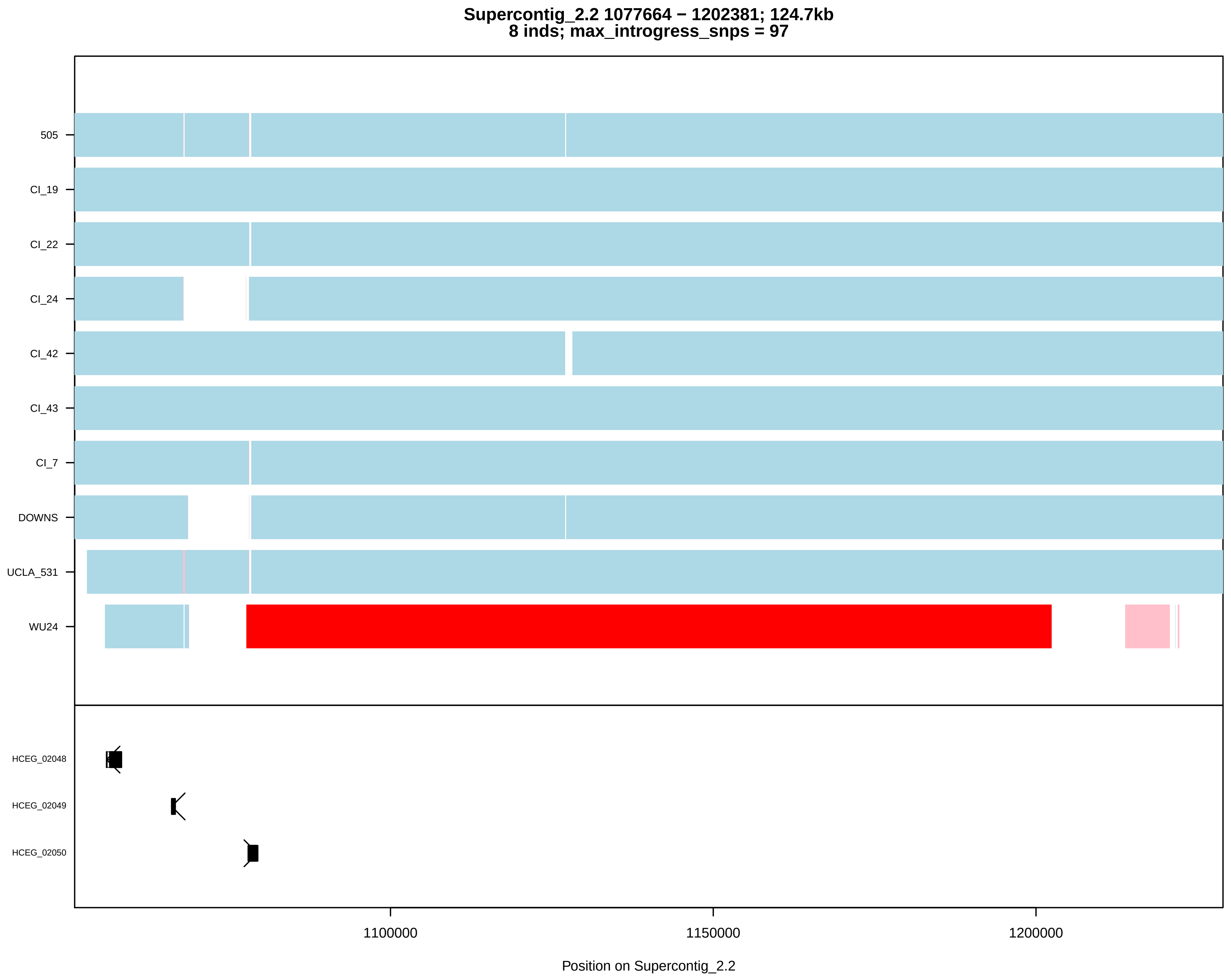
Supercontig_2.2 1077664 − 1202381; 124.7kb
8 inds; max_introgress_snps = 97
505
CI_19
CI_22
CI_24
CI_42
CI_43
CI_7
DOWNS
UCLA_531
WU24
HCEG_02048
HCEG_02049
HCEG_02050
1100000
1150000
1200000
Position on Supercontig_2.2

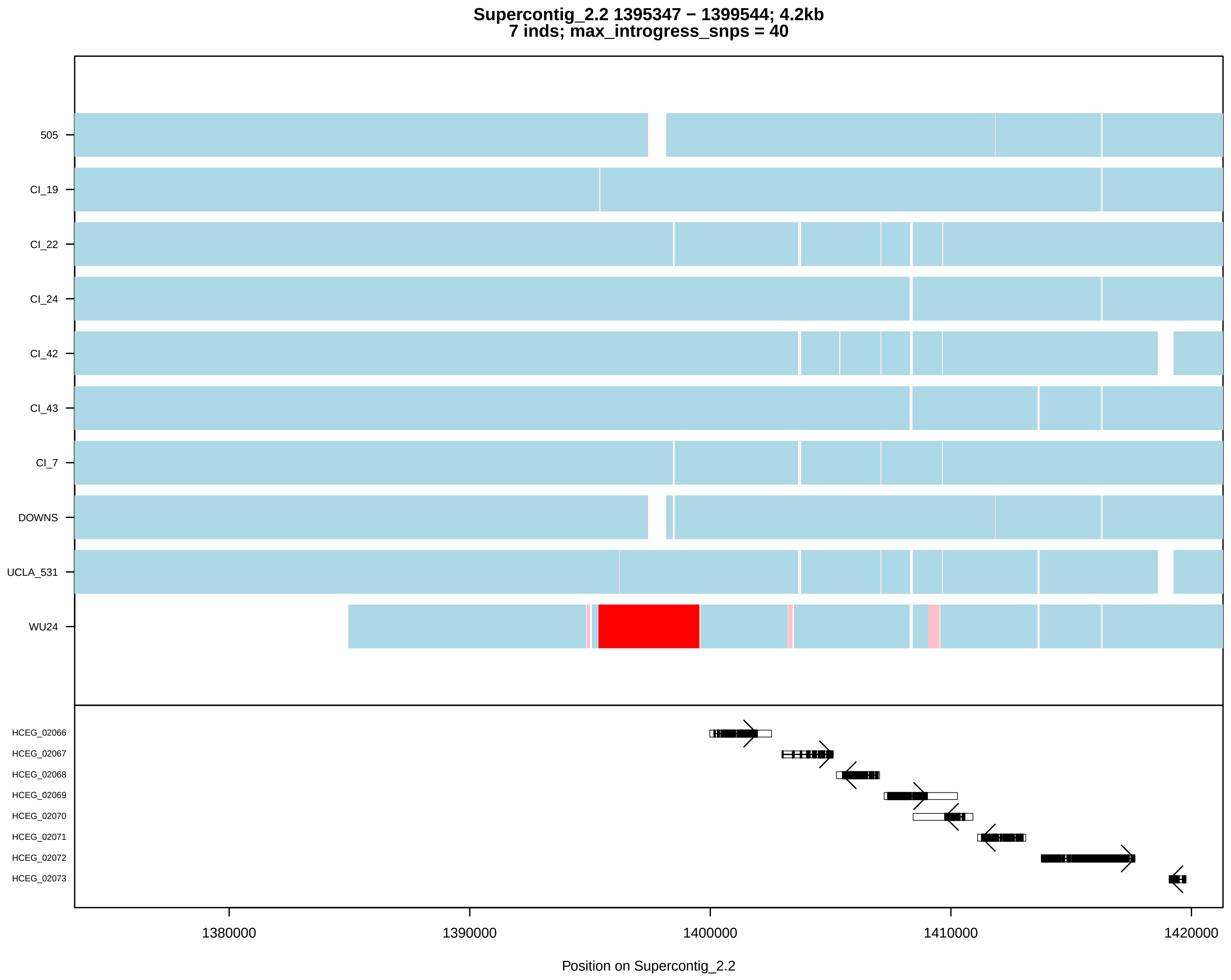

Supercontig_2.2 1395347 − 1399544; 4.2kb
7 inds; max_introgress_snps = 40
505
CI_19
CI_22
CI_24
CI_42
CI_43
CI_7
DOWNS
UCLA_531
WU24
HCEG_02066
HCEG_02067
HCEG_02068
HCEG_02069
HCEG_02070
HCEG_02071
HCEG_02072
HCEG_02073
1380000
1390000
1400000
1410000
1420000
Position on Supercontig_2.2

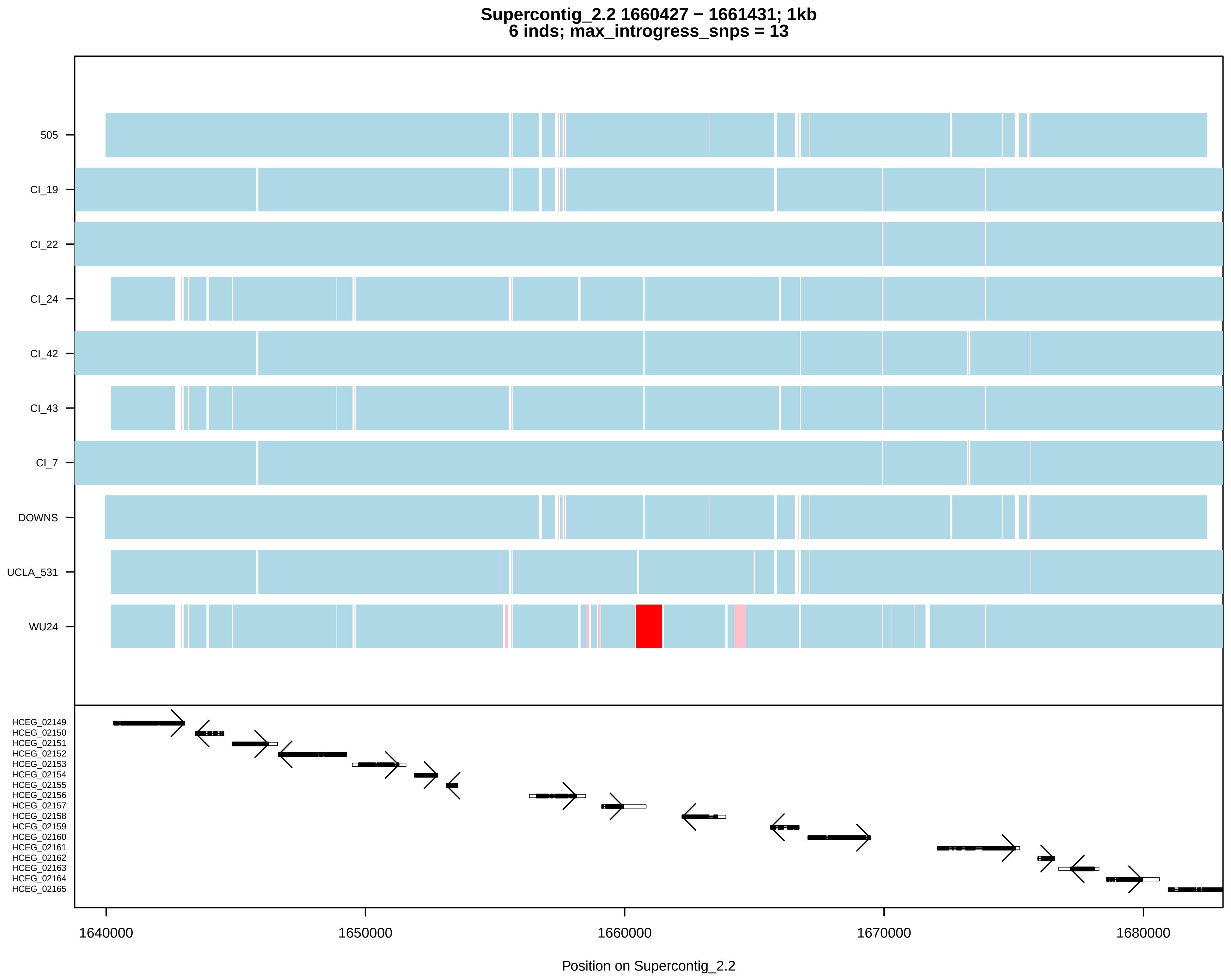
Supercontig_2.2 1660427 − 1661431; 1kb
6 inds; max_introgress_snps = 13
505
CI_19
CI_22
CI_24
CI_42
CI_43
CI_7
DOWNS
UCLA_531
WU24
HCEG_02149
HCEG_02150
HCEG_02151
HCEG_02152
HCEG_02153
HCEG_02154
HCEG_02155
HCEG_02156
HCEG_02157
HCEG_02158
HCEG_02159
HCEG_02160
HCEG_02161
HCEG_02162
HCEG_02163
HCEG_02164
HCEG_02165
1640000
1650000
1660000
1670000
1680000
Position on Supercontig_2.2

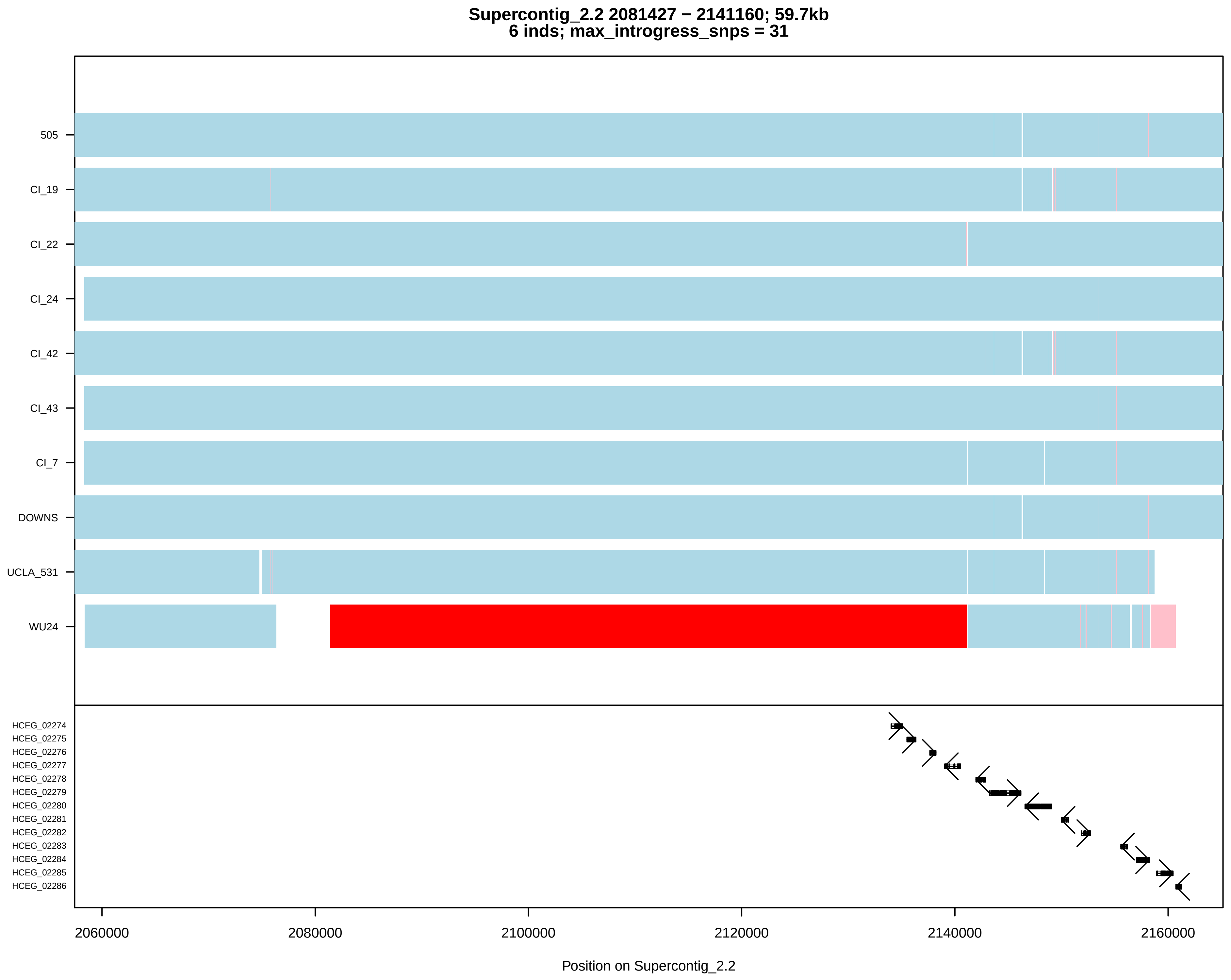

Supercontig_2.2 2081427 − 2141160; 59.7kb
6 inds; max_introgress_snps = 31
505
CI_19
CI_22
CI_24
CI_42
CI_43
CI_7
DOWNS
UCLA_531
WU24
HCEG_02274
HCEG_02275
HCEG_02276
HCEG_02277
HCEG_02278
HCEG_02279
HCEG_02280
HCEG_02281
HCEG_02282
HCEG_02283
HCEG_02284
HCEG_02285
HCEG_02286
2060000
2080000
2100000
2120000
2140000
2160000
Position on Supercontig_2.2

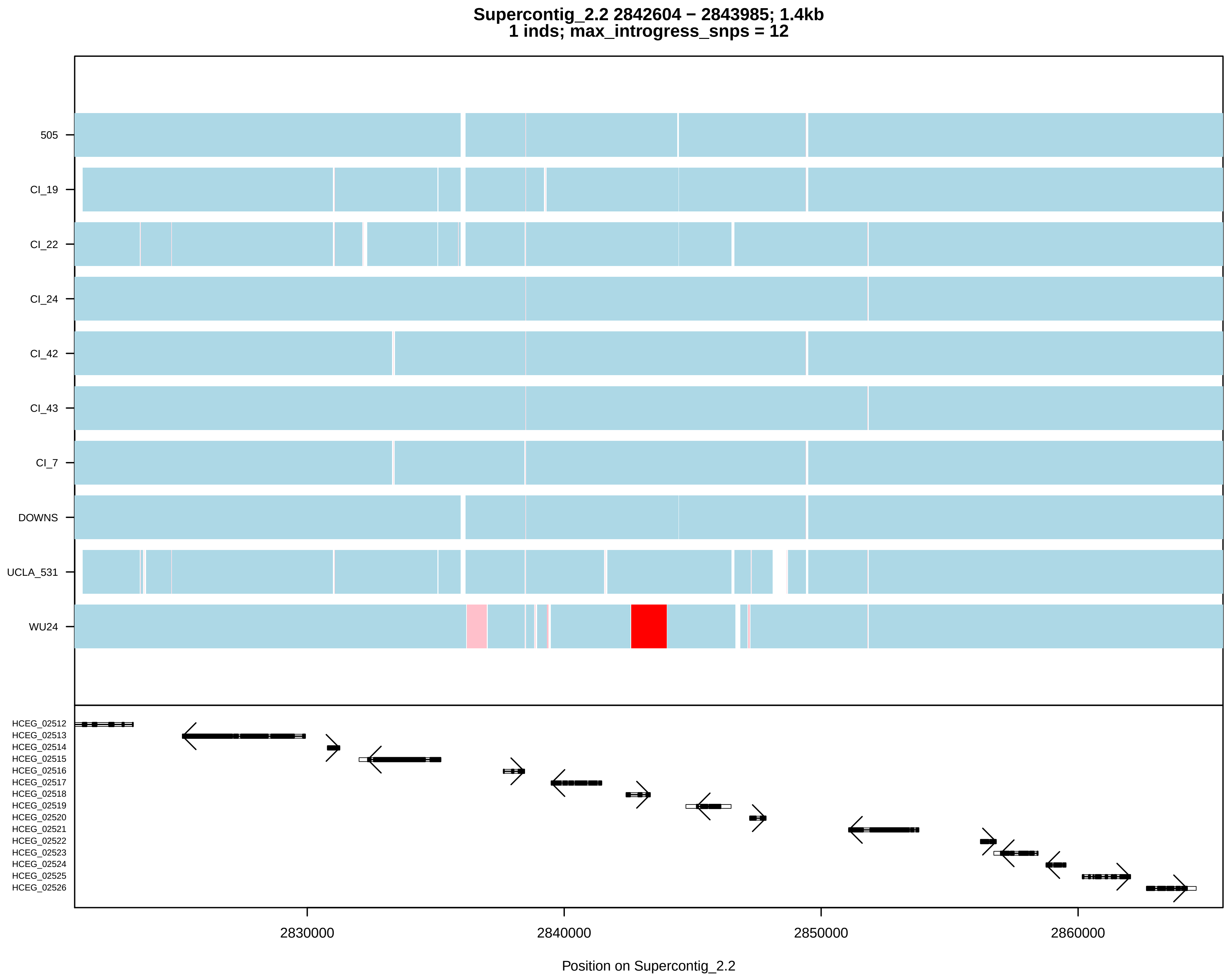
Supercontig_2.2 2842604 − 2843985; 1.4kb
1 inds; max_introgress_snps = 12
505
CI_19
CI_22
CI_24
CI_42
CI_43
CI_7
DOWNS
UCLA_531
WU24
HCEG_02512
HCEG_02513
HCEG_02514
HCEG_02515
HCEG_02516
HCEG_02517
HCEG_02518
HCEG_02519
HCEG_02520
HCEG_02521
HCEG_02522
HCEG_02523
HCEG_02524
HCEG_02525
HCEG_02526
2830000
2840000
2850000
2860000
Position on Supercontig_2.2

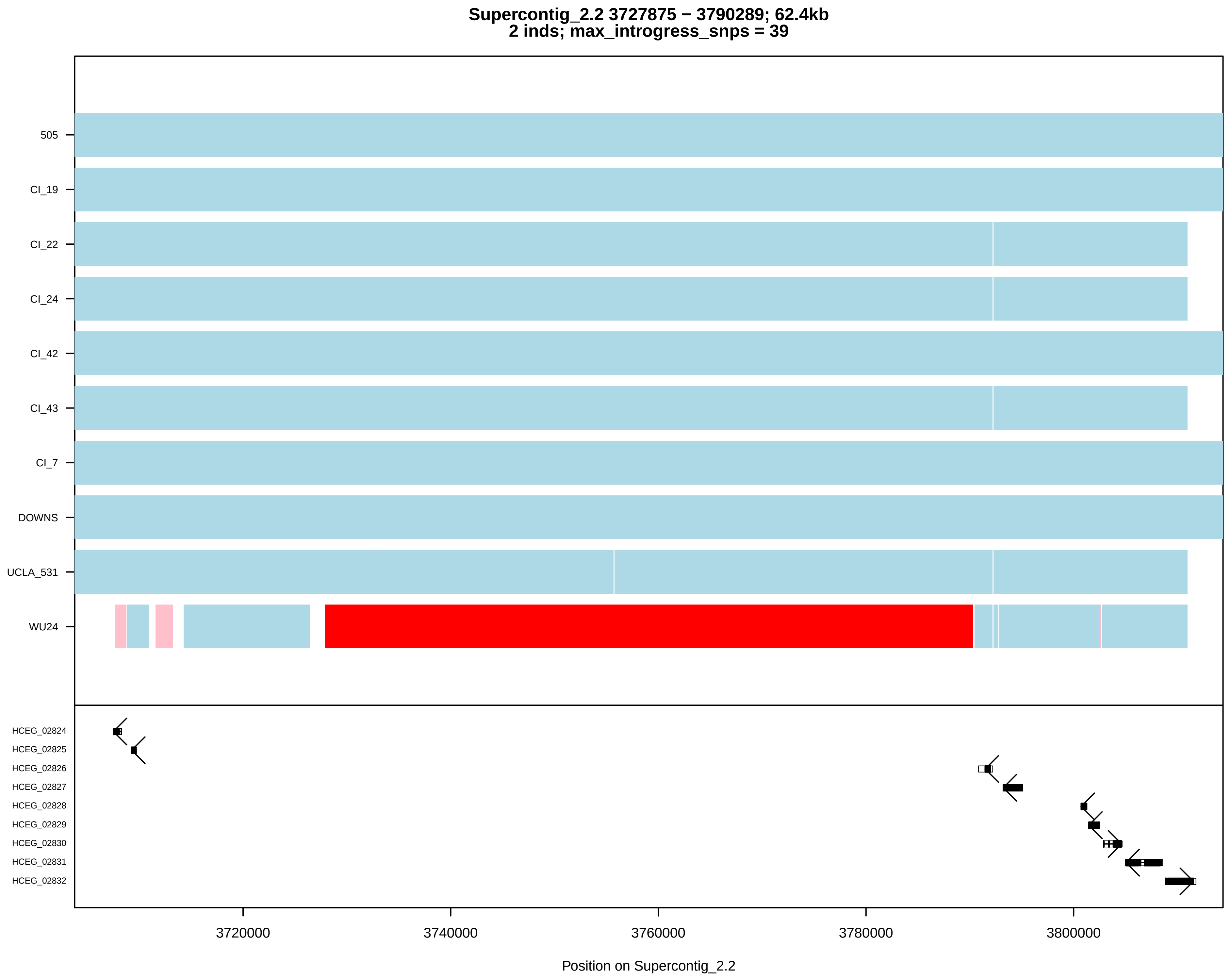
Supercontig_2.2 3727875 − 3790289; 62.4kb
2 inds; max_introgress_snps = 39
505
CI_19
CI_22
CI_24
CI_42
CI_43
CI_7
DOWNS
UCLA_531
WU24
HCEG_02824
HCEG_02825
HCEG_02826
HCEG_02827
HCEG_02828
HCEG_02829
HCEG_02830
HCEG_02831
HCEG_02832
3720000
3740000
3760000
3780000
3800000
Position on Supercontig_2.2

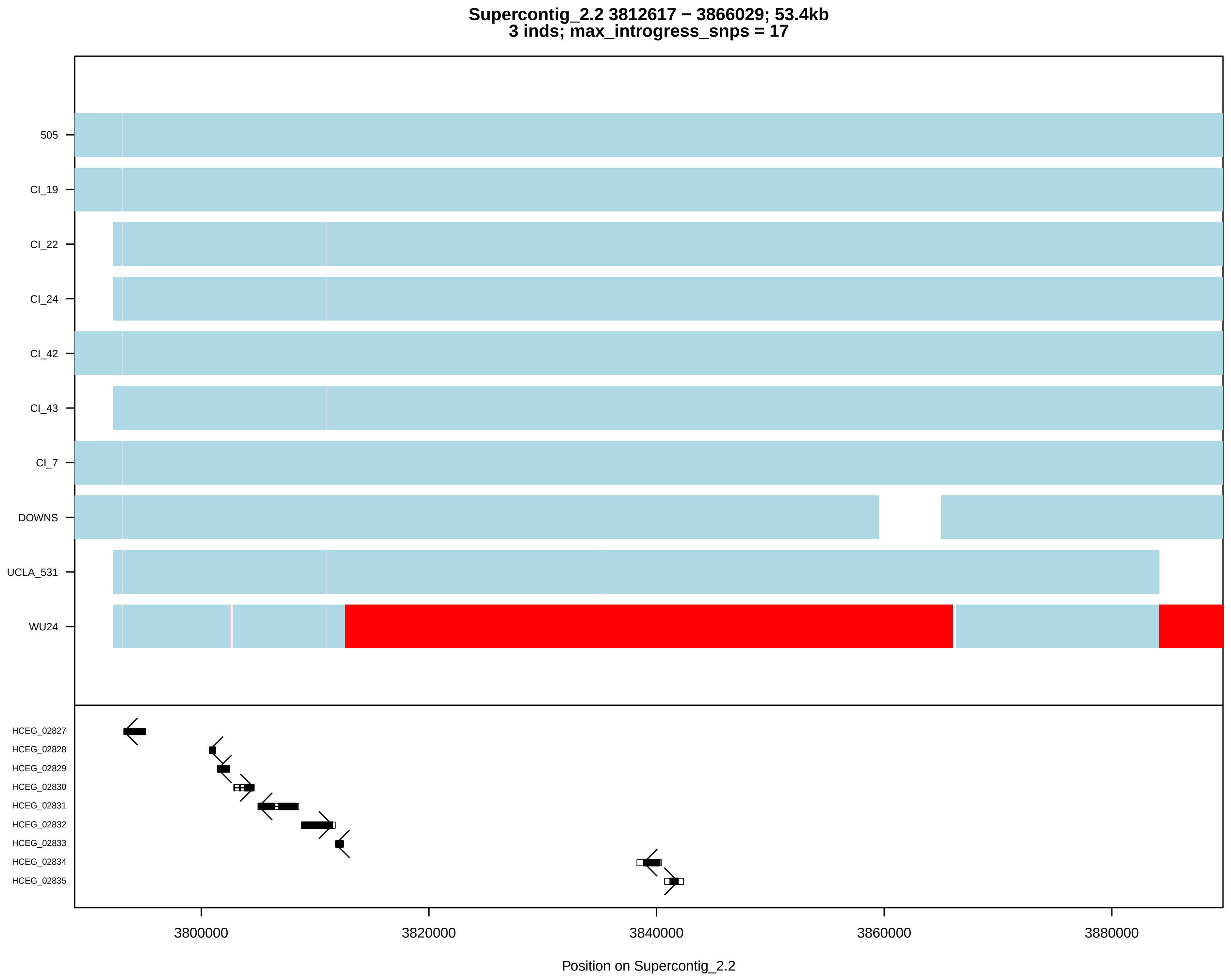

Supercontig_2.2 3812617 − 3866029; 53.4kb
3 inds; max_introgress_snps = 17
505
CI_19
CI_22
CI_24
CI_42
CI_43
CI_7
DOWNS
UCLA_531
WU24
HCEG_02827
HCEG_02828
HCEG_02829
HCEG_02830
HCEG_02831
HCEG_02832
HCEG_02833
HCEG_02834
HCEG_02835
3800000
3820000
3840000
3860000
3880000
Position on Supercontig_2.2

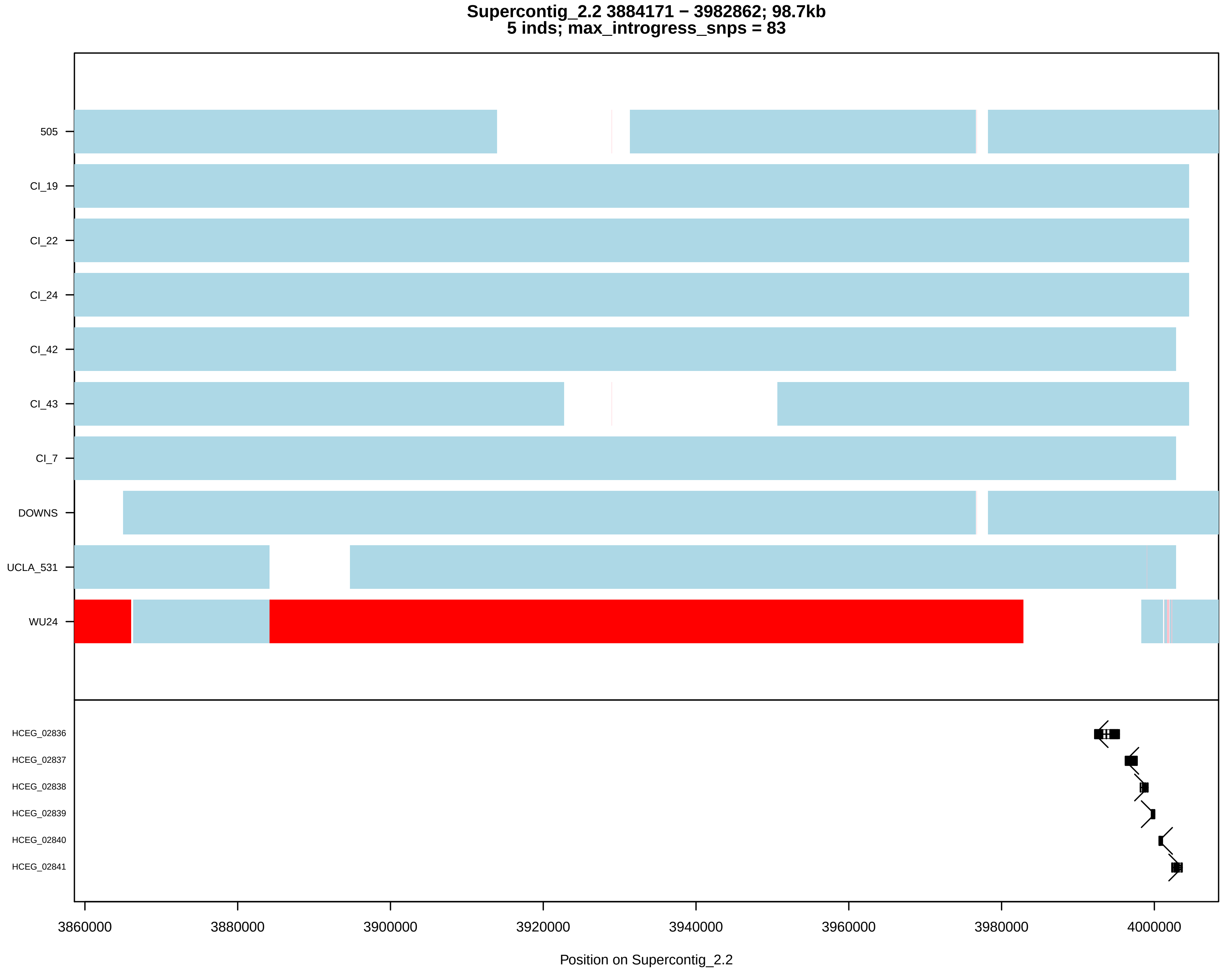
Supercontig_2.2 3884171 − 3982862; 98.7kb
5 inds; max_introgress_snps = 83
505
CI_19
CI_22
CI_24
CI_42
CI_43
CI_7
DOWNS
UCLA_531
WU24
HCEG_02836
HCEG_02837
HCEG_02838
HCEG_02839
HCEG_02840
HCEG_02841
3860000
3880000
3900000
3920000
3940000
3960000
3980000
4000000
Position on Supercontig_2.2

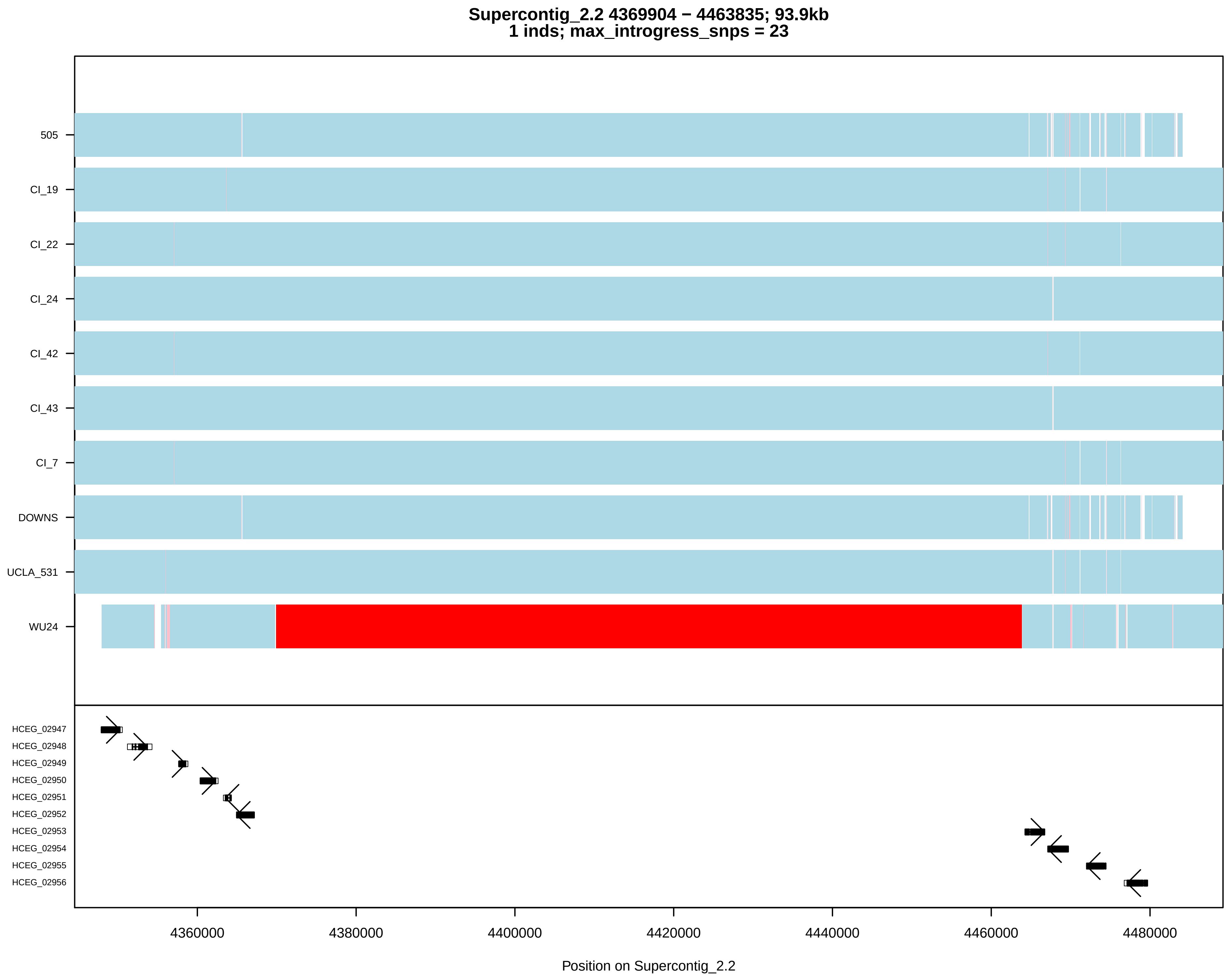

Supercontig_2.2 4369904 − 4463835; 93.9kb
1 inds; max_introgress_snps = 23
505
CI_19
CI_22
CI_24
CI_42
CI_43
CI_7
DOWNS
UCLA_531
WU24
HCEG_02947
HCEG_02948
HCEG_02949
HCEG_02950
HCEG_02951
HCEG_02952
HCEG_02953
HCEG_02954
HCEG_02955
HCEG_02956
4360000
4380000
4400000
4420000
4440000
4460000
4480000
Position on Supercontig_2.2

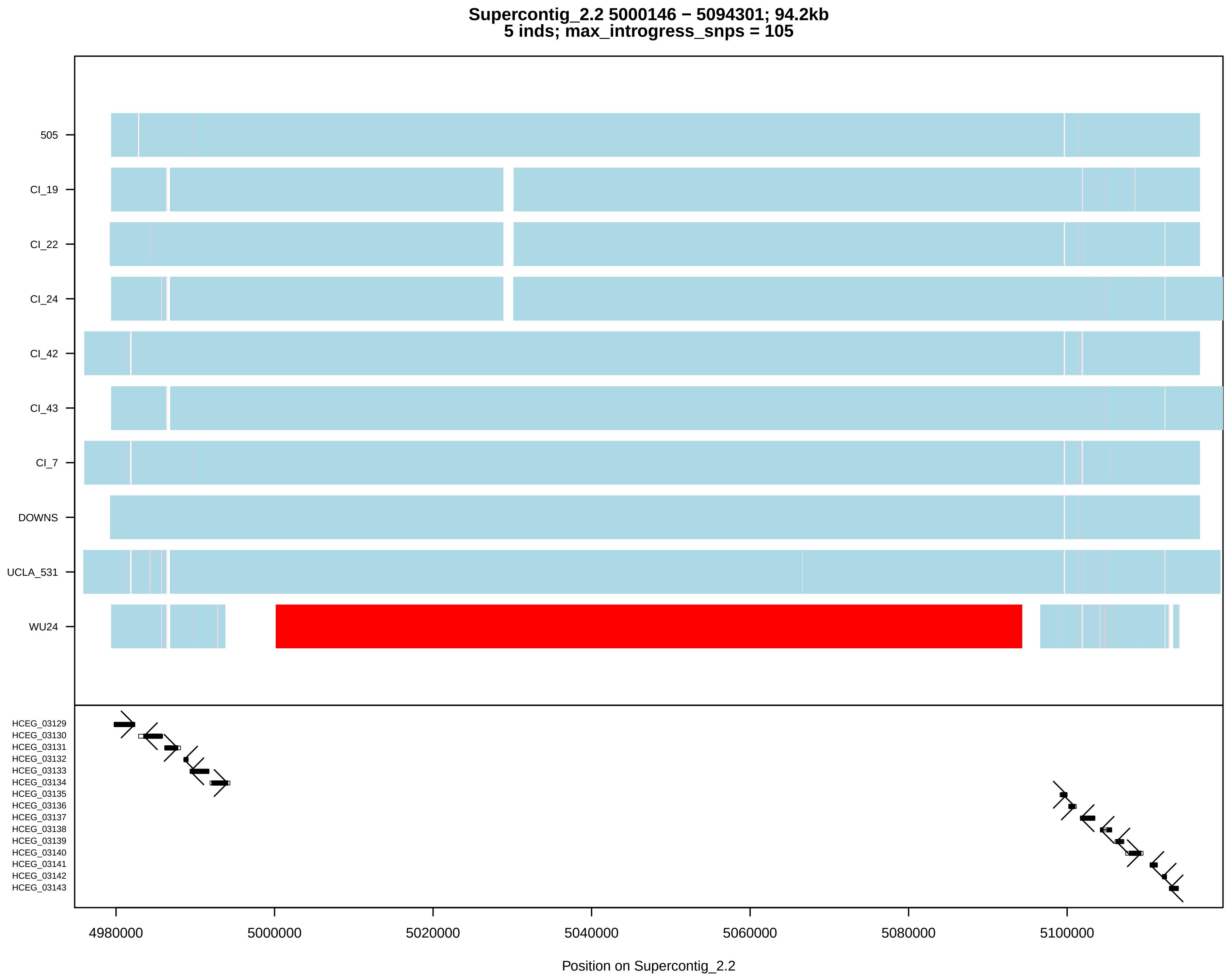
Supercontig_2.2 5000146 − 5094301; 94.2kb
5 inds; max_introgress_snps = 105
505
CI_19
CI_22
CI_24
CI_42
CI_43
CI_7
DOWNS
UCLA_531
WU24
HCEG_03129
HCEG_03130
HCEG_03131
HCEG_03132
HCEG_03133
HCEG_03134
HCEG_03135
HCEG_03136
HCEG_03137
HCEG_03138
HCEG_03139
HCEG_03140
HCEG_03141
HCEG_03142
HCEG_03143
4980000
5000000
5020000
5040000
5060000
5080000
5100000
Position on Supercontig_2.2

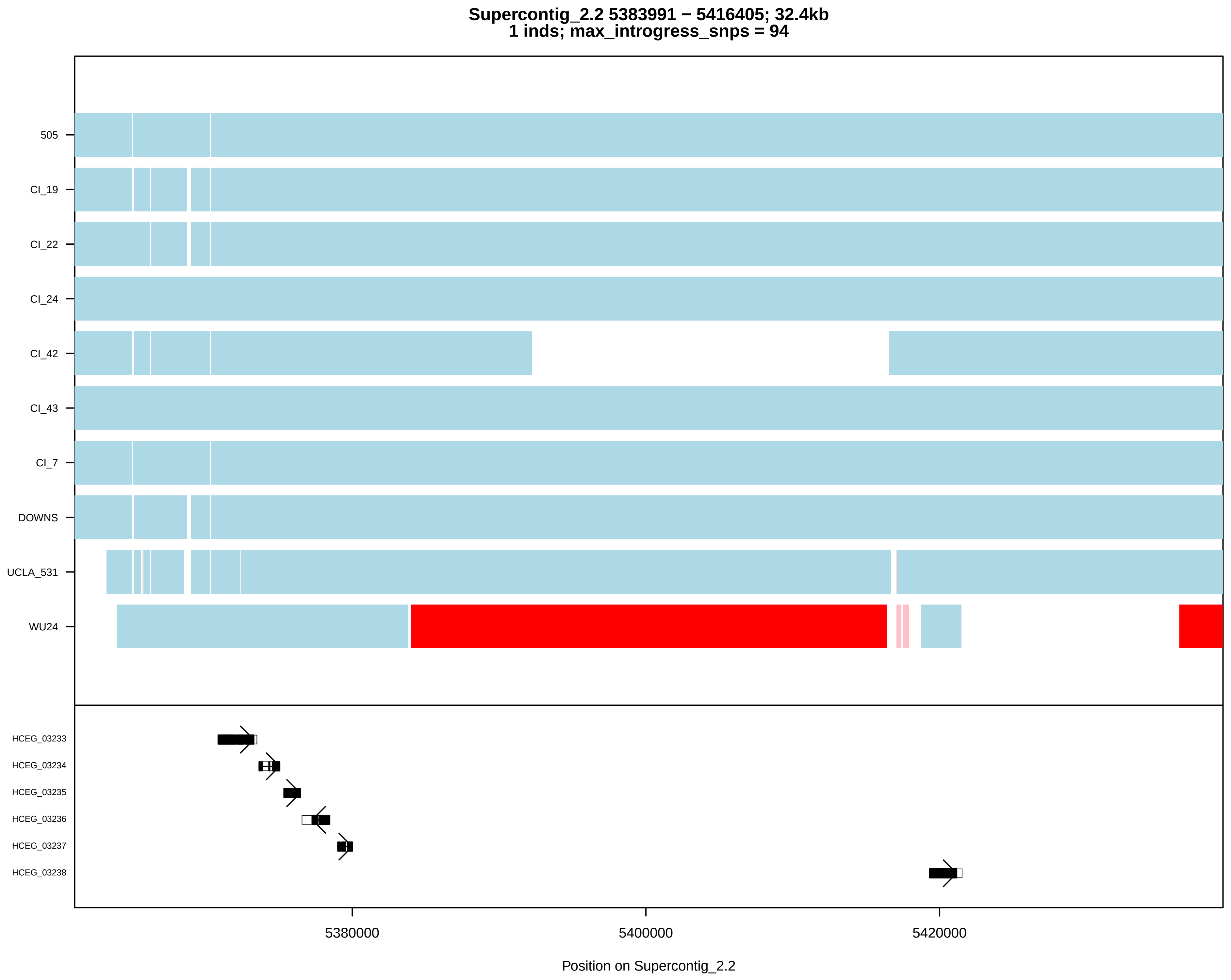
Supercontig_2.2 5383991 − 5416405; 32.4kb
1 inds; max_introgress_snps = 94
505
CI_19
CI_22
CI_24
CI_42
CI_43
CI_7
DOWNS
UCLA_531
WU24
HCEG_03233
HCEG_03234
HCEG_03235
HCEG_03236
HCEG_03237
HCEG_03238
5380000
5400000
5420000
Position on Supercontig_2.2

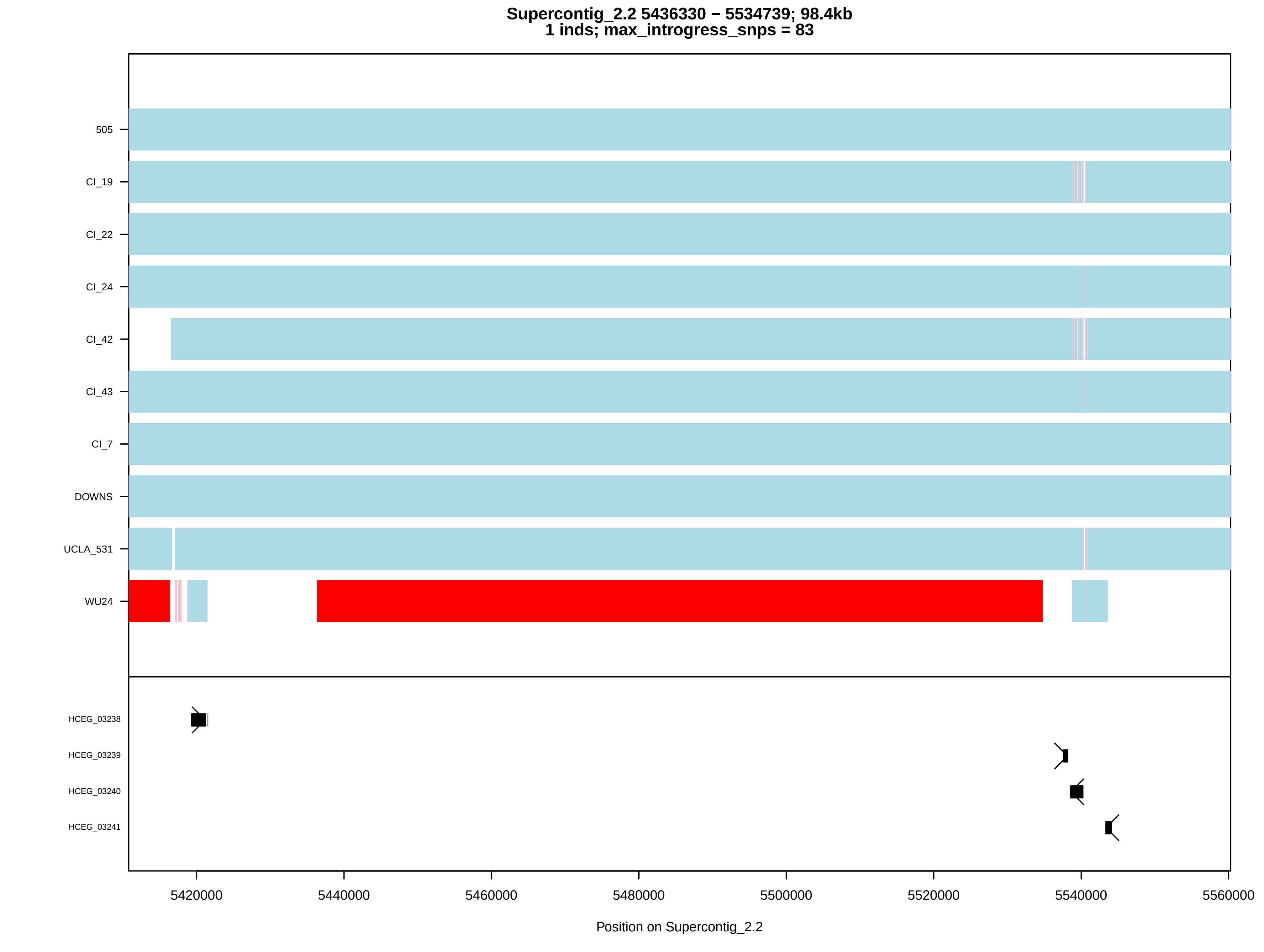

Supercontig_2.2 5436330 − 5534739; 98.4kb
1 inds; max_introgress_snps = 83
505
CI_19
CI_22
CI_24
CI_42
CI_43
CI_7
DOWNS
UCLA_531
WU24
HCEG_03238
HCEG_03239
HCEG_03240
HCEG_03241
5420000
5440000
5460000
5480000
5500000
5520000
5540000
5560000
Position on Supercontig_2.2

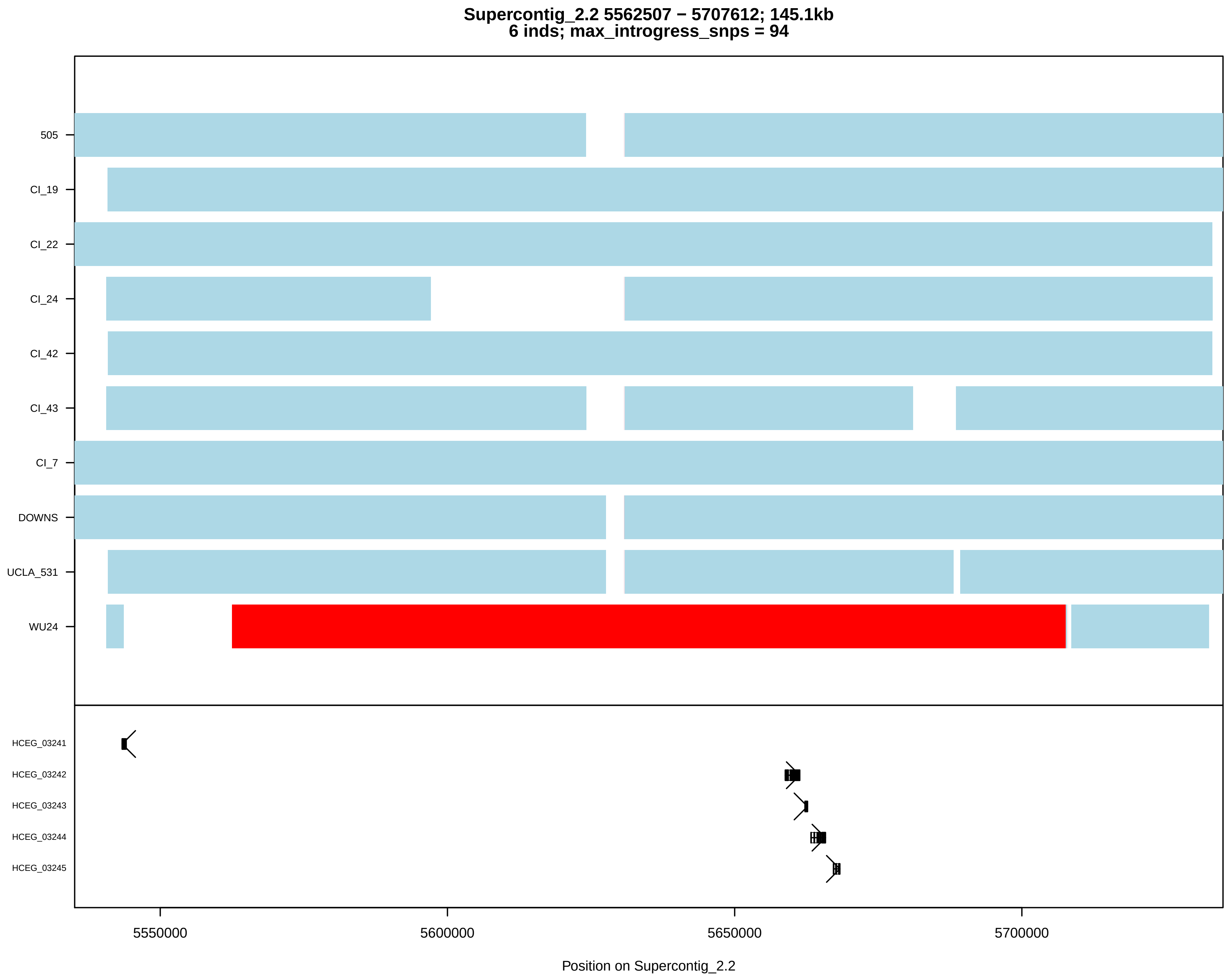
Supercontig_2.2 5562507 − 5707612; 145.1kb
6 inds; max_introgress_snps = 94
505
CI_19
CI_22
CI_24
CI_42
CI_43
CI_7
DOWNS
UCLA_531
WU24
HCEG_03241
HCEG_03242
HCEG_03243
HCEG_03244
HCEG_03245
5550000
5600000
5650000
5700000
Position on Supercontig_2.2

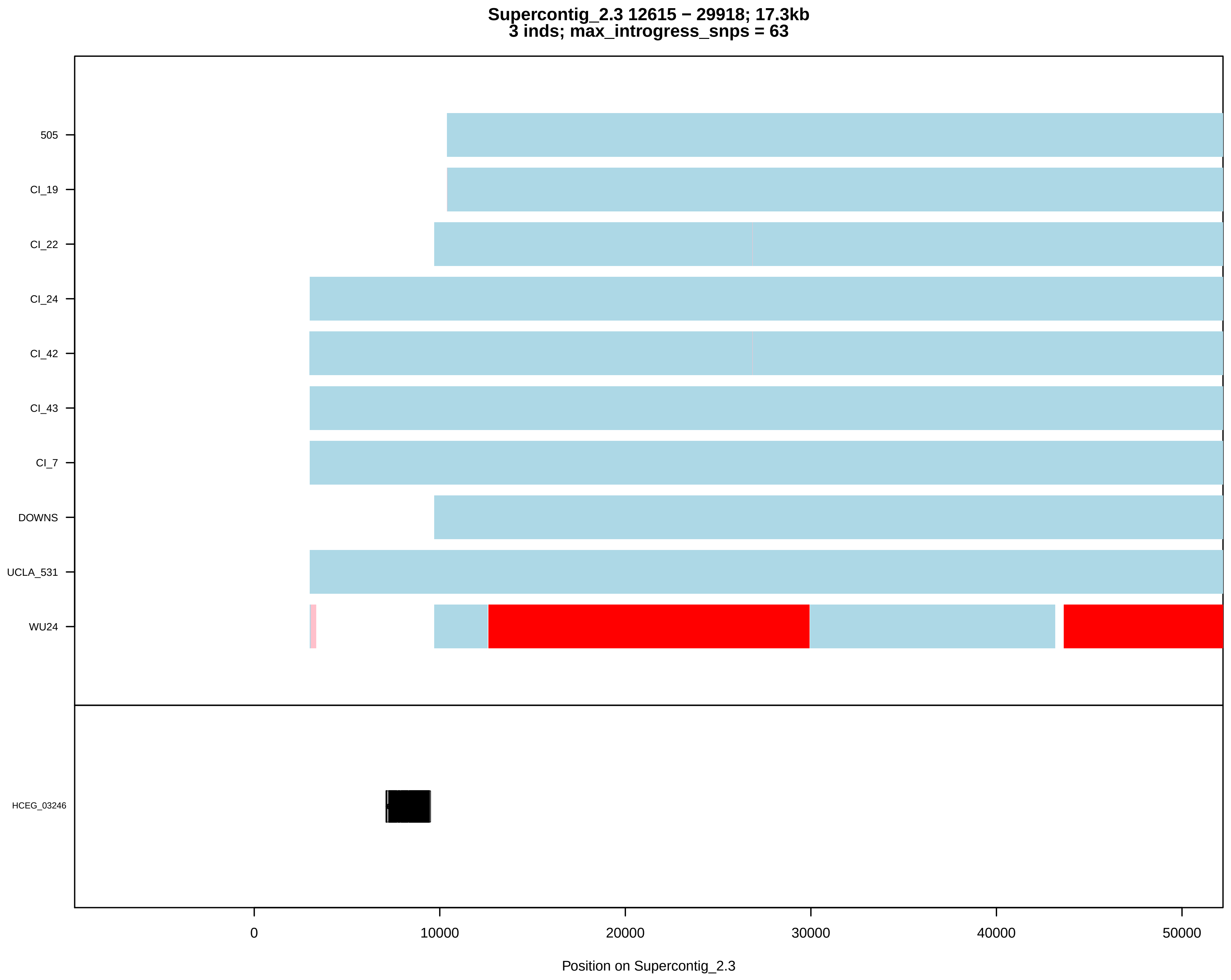

Supercontig_2.3 12615 − 29918; 17.3kb
3 inds; max_introgress_snps = 63
505
CI_19
CI_22
CI_24
CI_42
CI_43
CI_7
DOWNS
UCLA_531
WU24
HCEG_03246
0
10000
20000
30000
40000
50000
Position on Supercontig_2.3

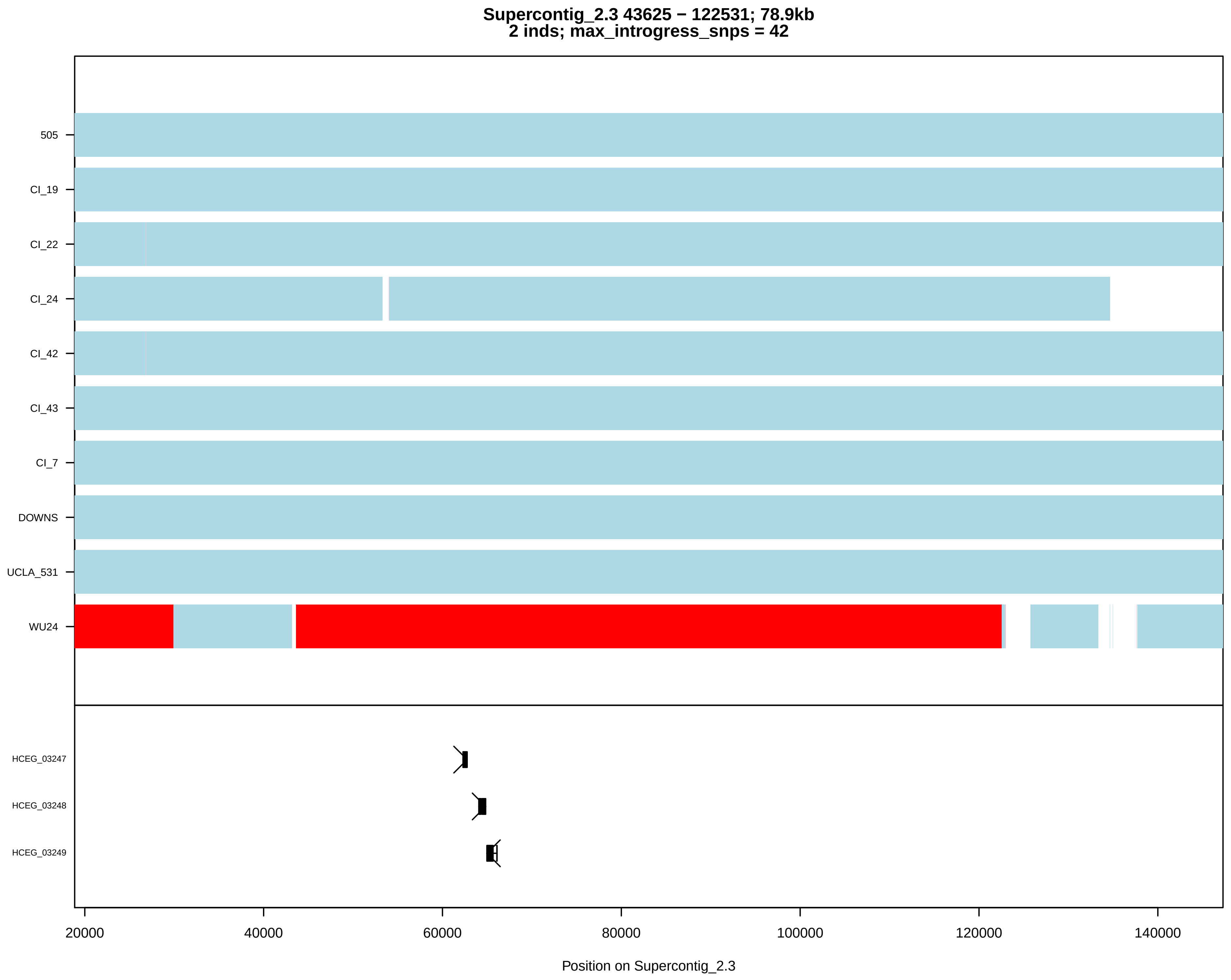

Supercontig_2.3 43625 − 122531; 78.9kb
2 inds; max_introgress_snps = 42
505
CI_19
CI_22
CI_24
CI_42
CI_43
CI_7
DOWNS
UCLA_531
WU24
HCEG_03247
HCEG_03248
HCEG_03249
20000
40000
60000
80000
100000
120000
140000
Position on Supercontig_2.3

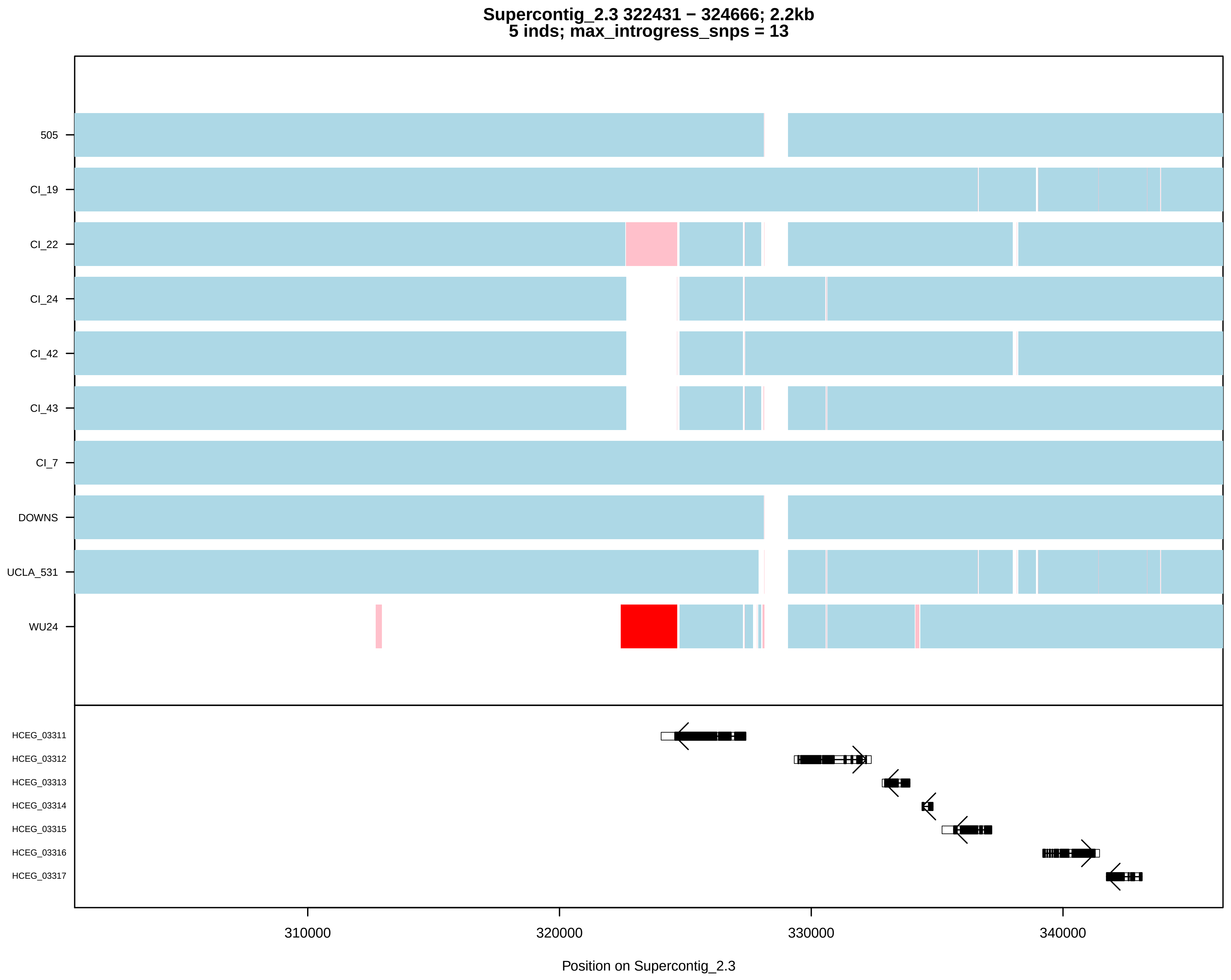
Supercontig_2.3 322431 − 324666; 2.2kb
5 inds; max_introgress_snps = 13
505
CI_19
CI_22
CI_24
CI_42
CI_43
CI_7
DOWNS
UCLA_531
WU24
HCEG_03311
HCEG_03312
HCEG_03313
HCEG_03314
HCEG_03315
HCEG_03316
HCEG_03317
310000
320000
330000
340000
Position on Supercontig_2.3

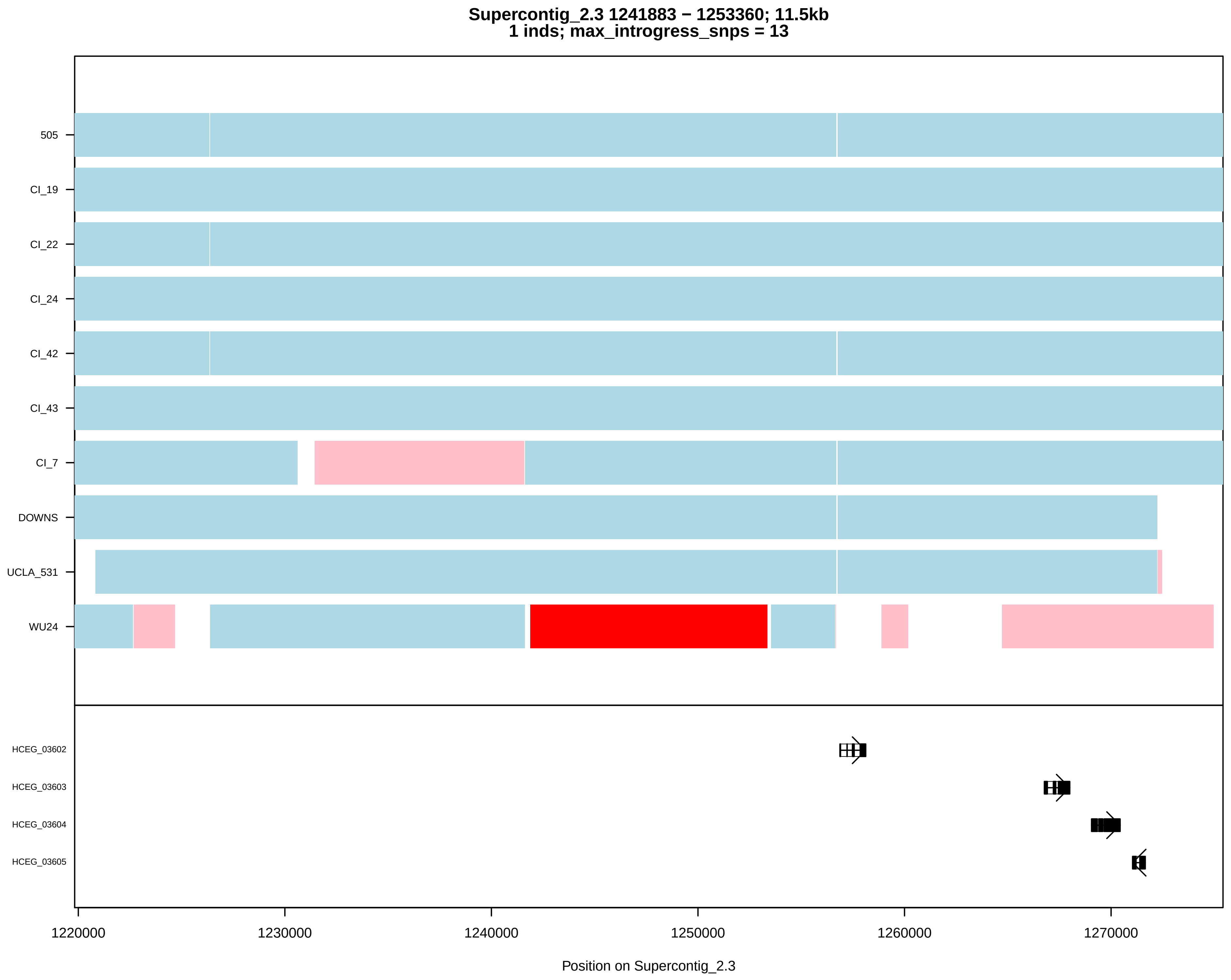
Supercontig_2.3 1241883 − 1253360; 11.5kb
1 inds; max_introgress_snps = 13
505
CI_19
CI_22
CI_24
CI_42
CI_43
CI_7
DOWNS
UCLA_531
WU24
HCEG_03602
HCEG_03603
HCEG_03604
HCEG_03605
1220000
1230000
1240000
1250000
1260000
1270000
Position on Supercontig_2.3

Supercontig_2.3 1499060 − 1501352; 2.3kb
1 inds; max_introgress_snps = 21

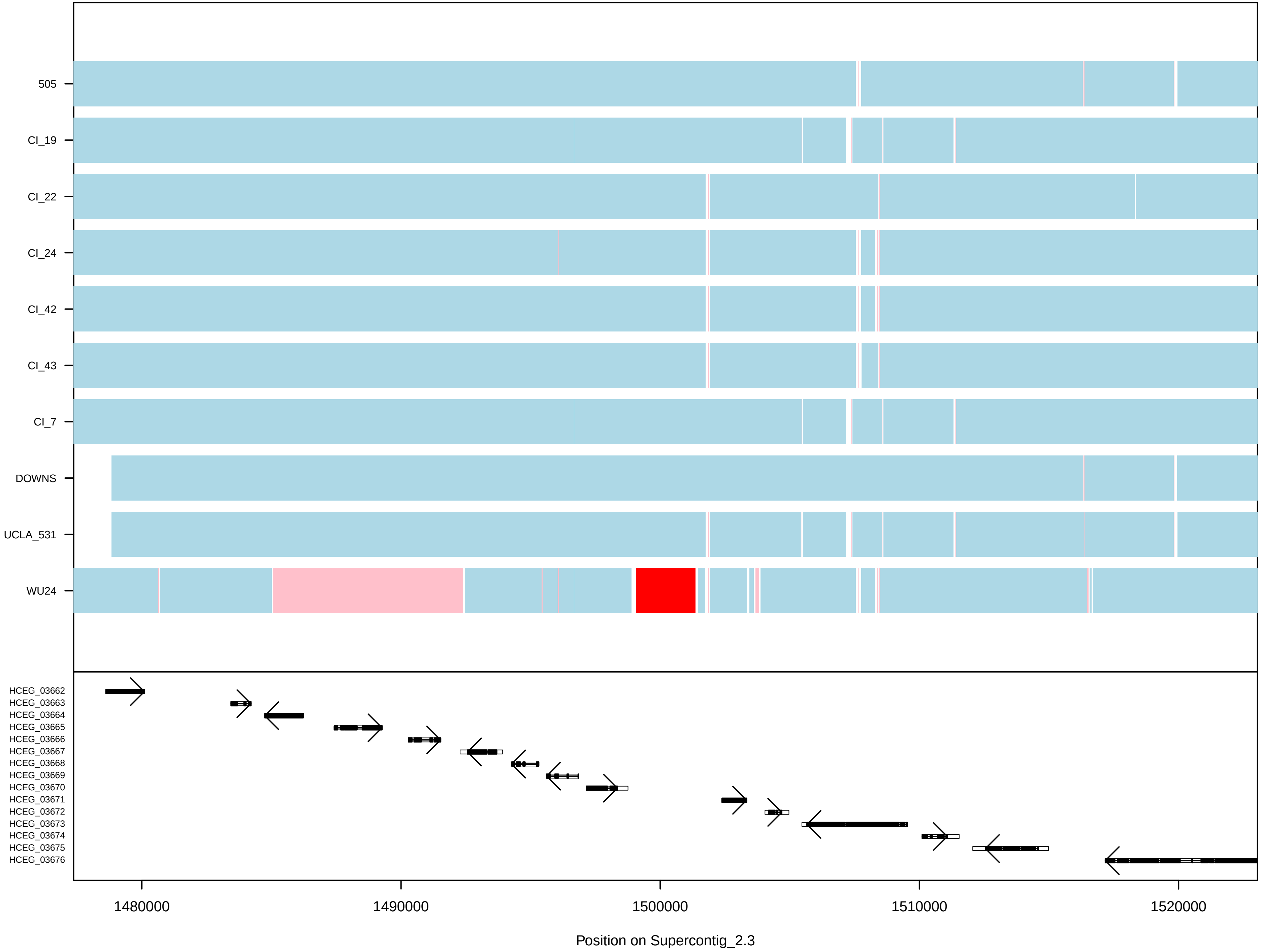

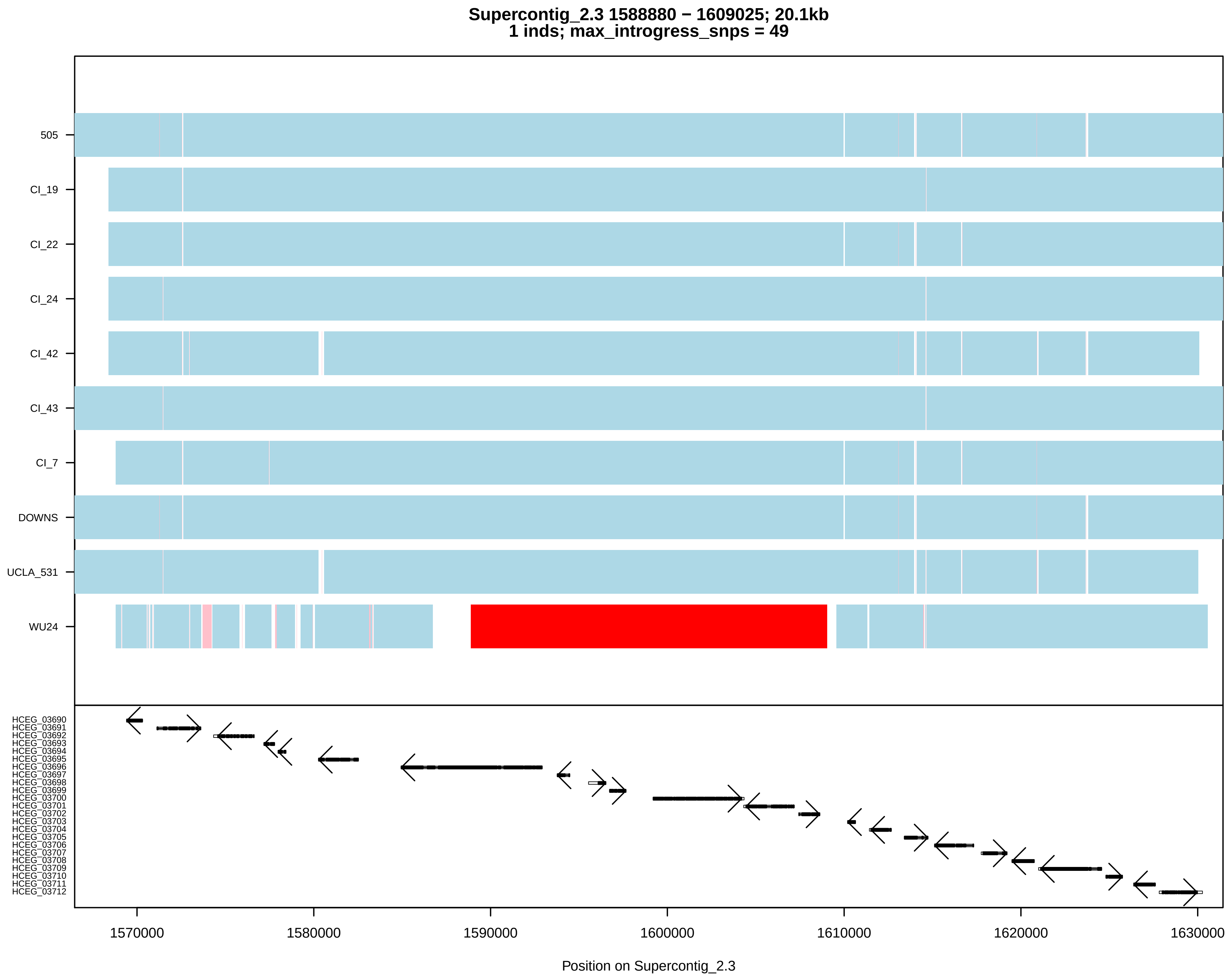

Supercontig_2.3 1588880 − 1609025; 20.1kb
1 inds; max_introgress_snps = 49
505
CI_19
CI_22
CI_24
CI_42
CI_43
CI_7
DOWNS
UCLA_531
WU24
HCEG_03690
HCEG_03691
HCEG_03692
HCEG_03693
HCEG_03694
HCEG_03695
HCEG_03696
HCEG_03697
HCEG_03698
HCEG_03699
HCEG_03700
HCEG_03701
HCEG_03702
HCEG_03703
HCEG_03704
HCEG_03705
HCEG_03706
HCEG_03707
HCEG_03708
HCEG_03709
HCEG_03710
HCEG_03711
HCEG_03712
1570000
1580000
1590000
1600000
1610000
1620000
1630000
Position on Supercontig_2.3

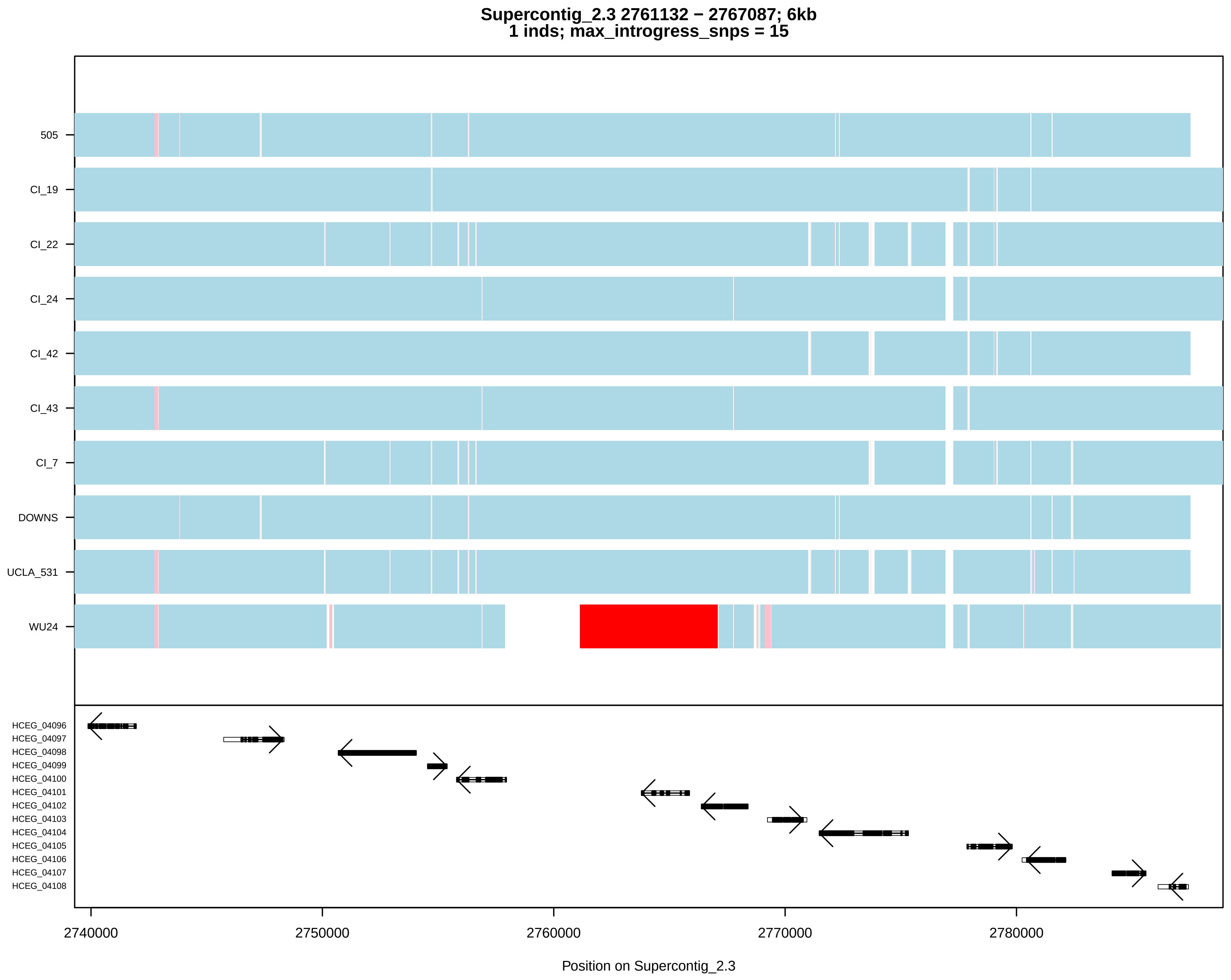
Supercontig_2.3 2761132 − 2767087; 6kb
1 inds; max_introgress_snps = 15
505
CI_19
CI_22
CI_24
CI_42
CI_43
CI_7
DOWNS
UCLA_531
WU24
HCEG_04096
HCEG_04097
HCEG_04098
HCEG_04099
HCEG_04100
HCEG_04101
HCEG_04102
HCEG_04103
HCEG_04104
HCEG_04105
HCEG_04106
HCEG_04107
HCEG_04108
2740000
2750000
2760000
2770000
2780000
Position on Supercontig_2.3

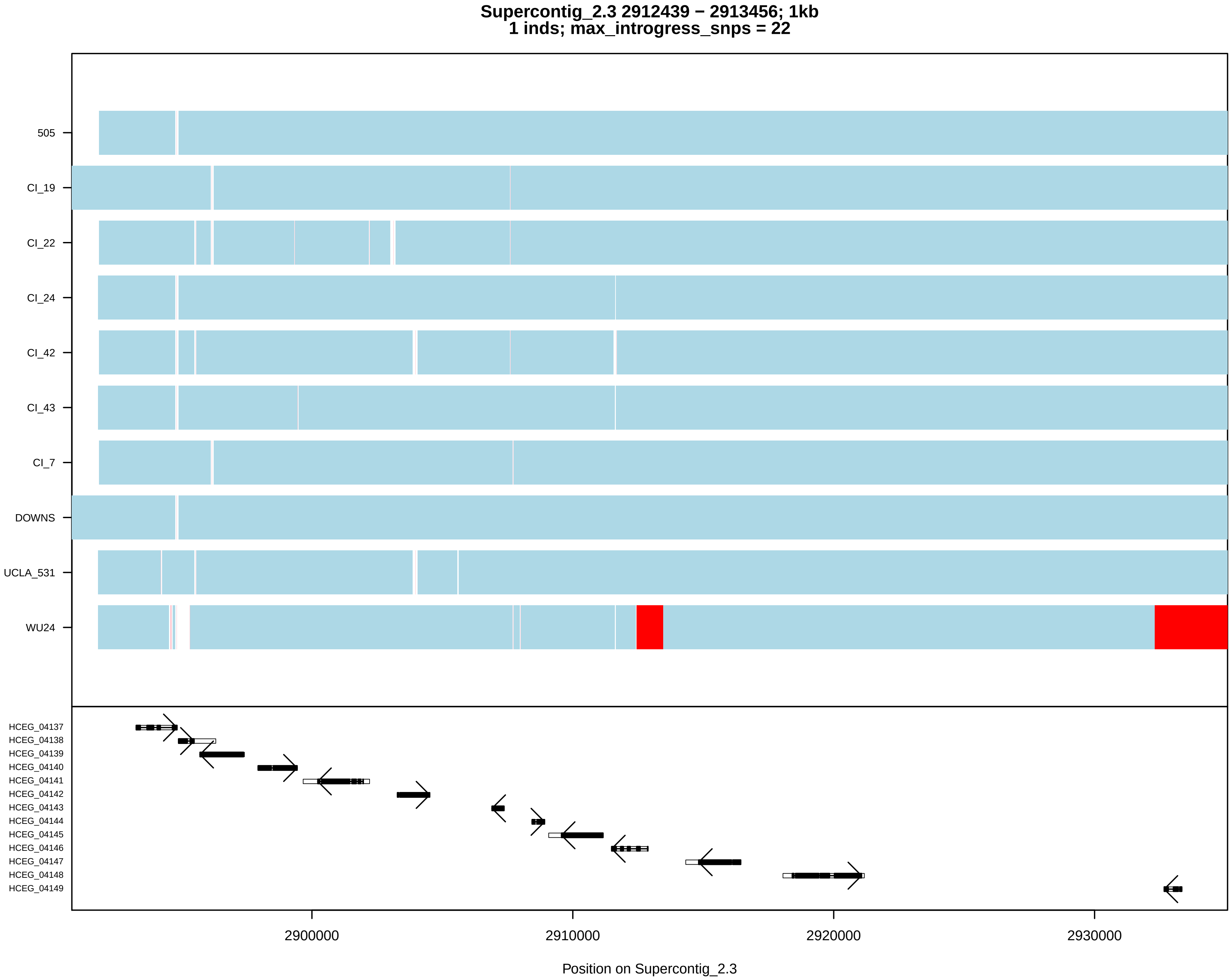
Supercontig_2.3 2912439 − 2913456; 1kb
1 inds; max_introgress_snps = 22
505
CI_19
CI_22
CI_24
CI_42
CI_43
CI_7
DOWNS
UCLA_531
WU24
HCEG_04137
HCEG_04138
HCEG_04139
HCEG_04140
HCEG_04141
HCEG_04142
HCEG_04143
HCEG_04144
HCEG_04145
HCEG_04146
HCEG_04147
HCEG_04148
HCEG_04149
2900000
2910000
2920000
2930000
Position on Supercontig_2.3

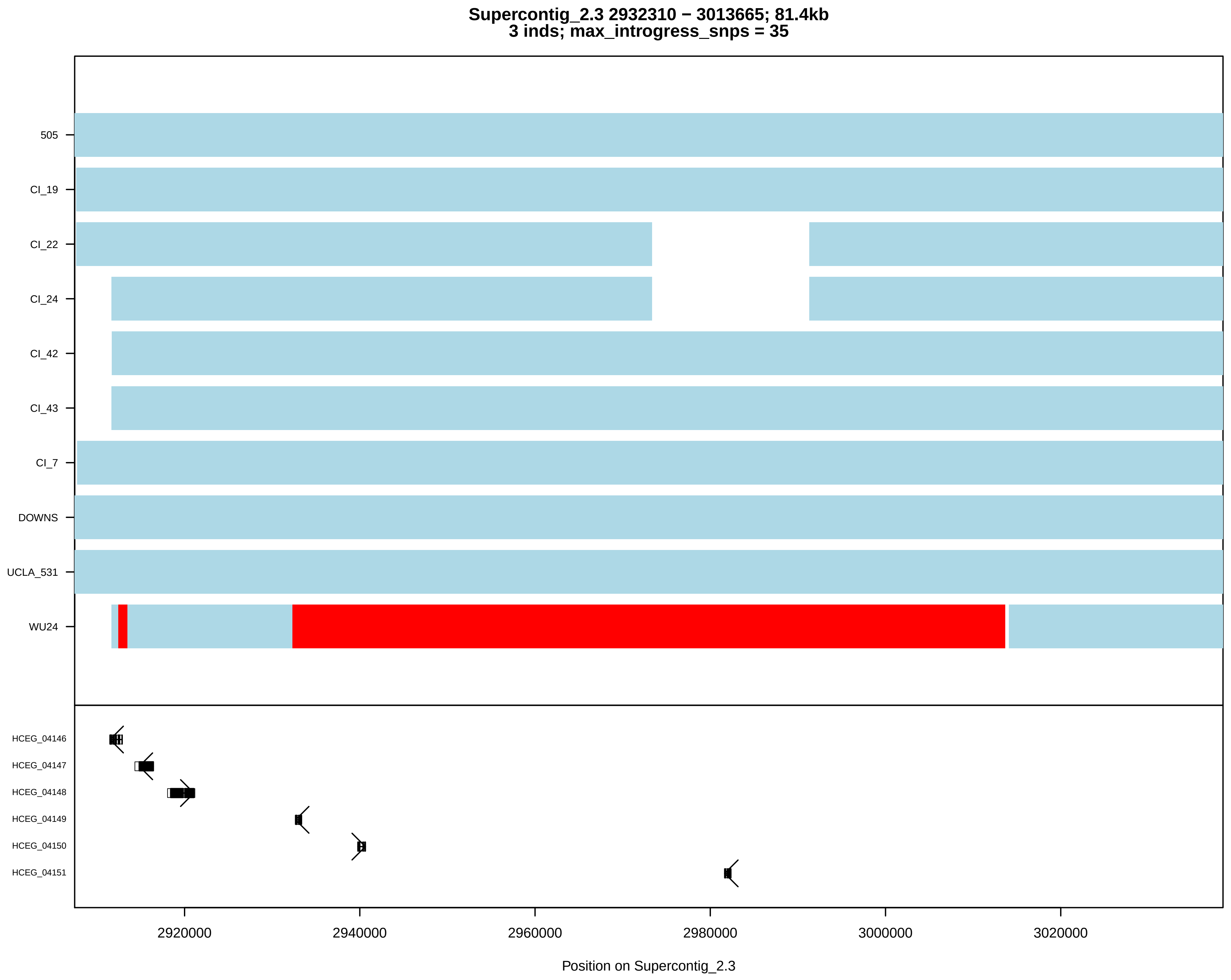
Supercontig_2.3 2932310 − 3013665; 81.4kb
3 inds; max_introgress_snps = 35
505
CI_19
CI_22
CI_24
CI_42
CI_43
CI_7
DOWNS
UCLA_531
WU24
HCEG_04146
HCEG_04147
HCEG_04148
HCEG_04149
HCEG_04150
HCEG_04151
2920000
2940000
2960000
2980000
3000000
3020000
Position on Supercontig_2.3

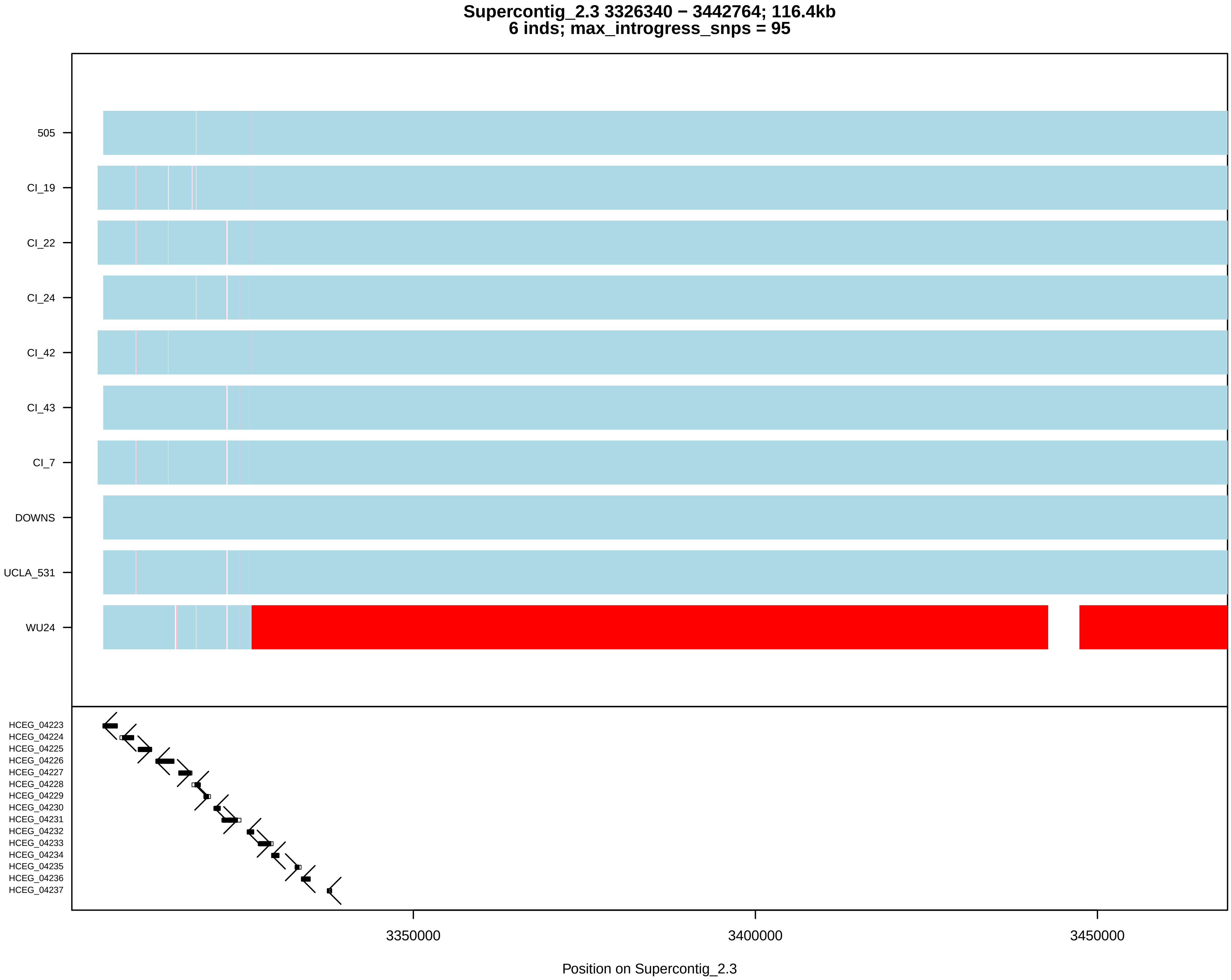

Supercontig_2.3 3326340 − 3442764; 116.4kb
6 inds; max_introgress_snps = 95
505
CI_19
CI_22
CI_24
CI_42
CI_43
CI_7
DOWNS
UCLA_531
WU24
HCEG_04223
HCEG_04224
HCEG_04225
HCEG_04226
HCEG_04227
HCEG_04228
HCEG_04229
HCEG_04230
HCEG_04231
HCEG_04232
HCEG_04233
HCEG_04234
HCEG_04235
HCEG_04236
HCEG_04237
3350000
3400000
3450000
Position on Supercontig_2.3

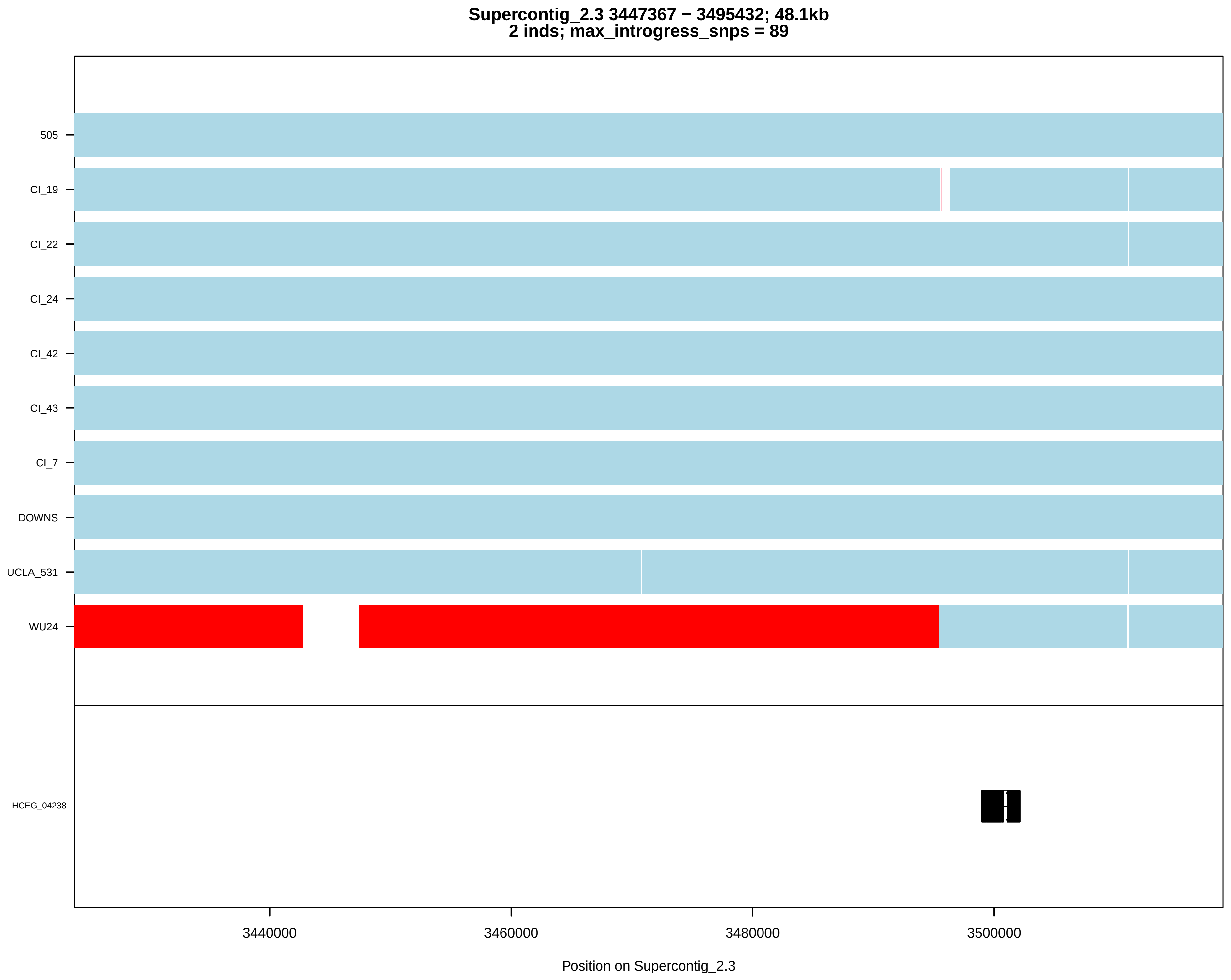
Supercontig_2.3 3447367 − 3495432; 48.1kb
2 inds; max_introgress_snps = 89
505
CI_19
CI_22
CI_24
CI_42
CI_43
CI_7
DOWNS
UCLA_531
WU24
HCEG_04238
3440000
3460000
3480000
3500000
Position on Supercontig_2.3

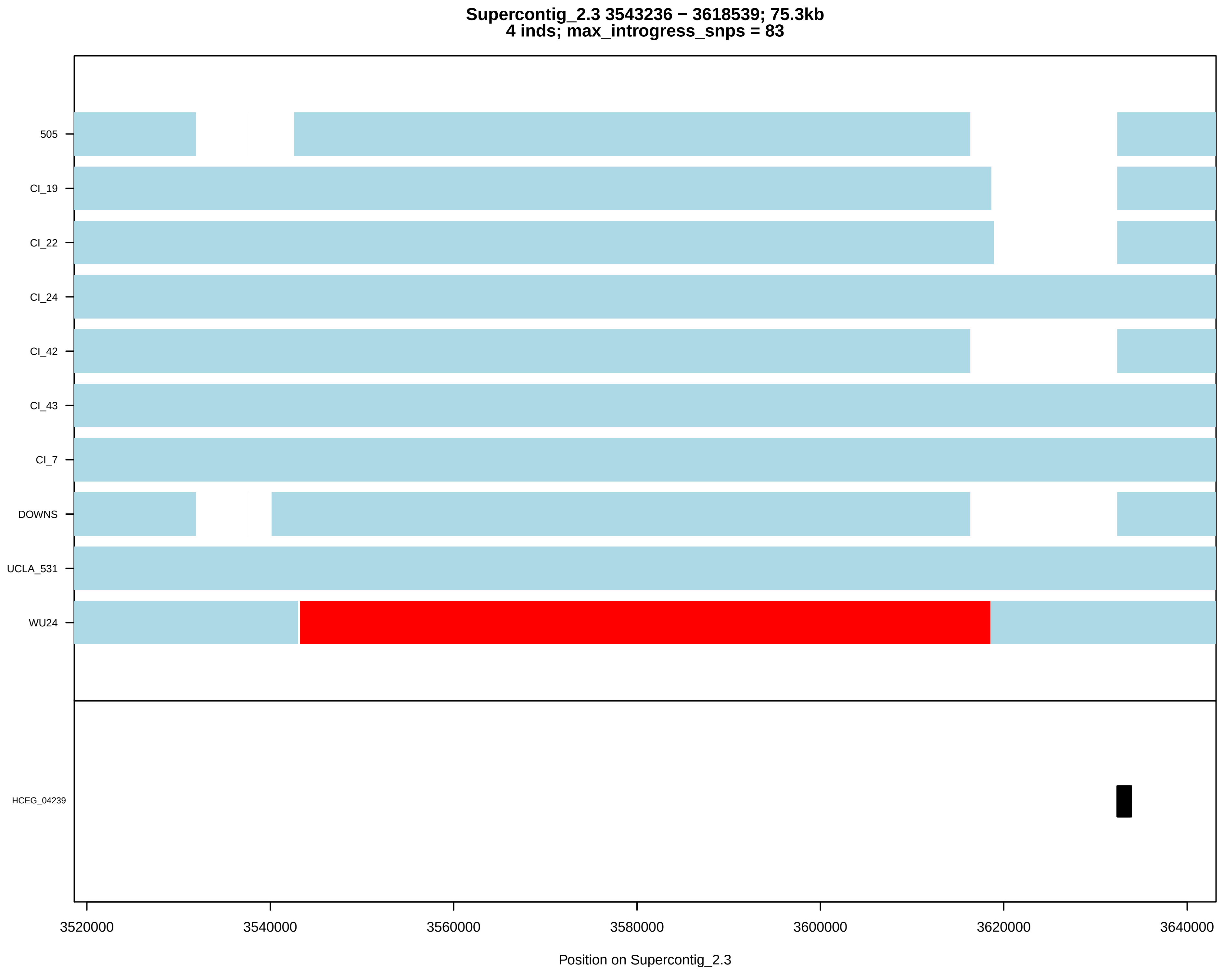

Supercontig_2.3 3543236 − 3618539; 75.3kb
4 inds; max_introgress_snps = 83
505
CI_19
CI_22
CI_24
CI_42
CI_43
CI_7
DOWNS
UCLA_531
WU24
HCEG_04239
3520000
3540000
3560000
3580000
3600000
3620000
3640000
Position on Supercontig_2.3

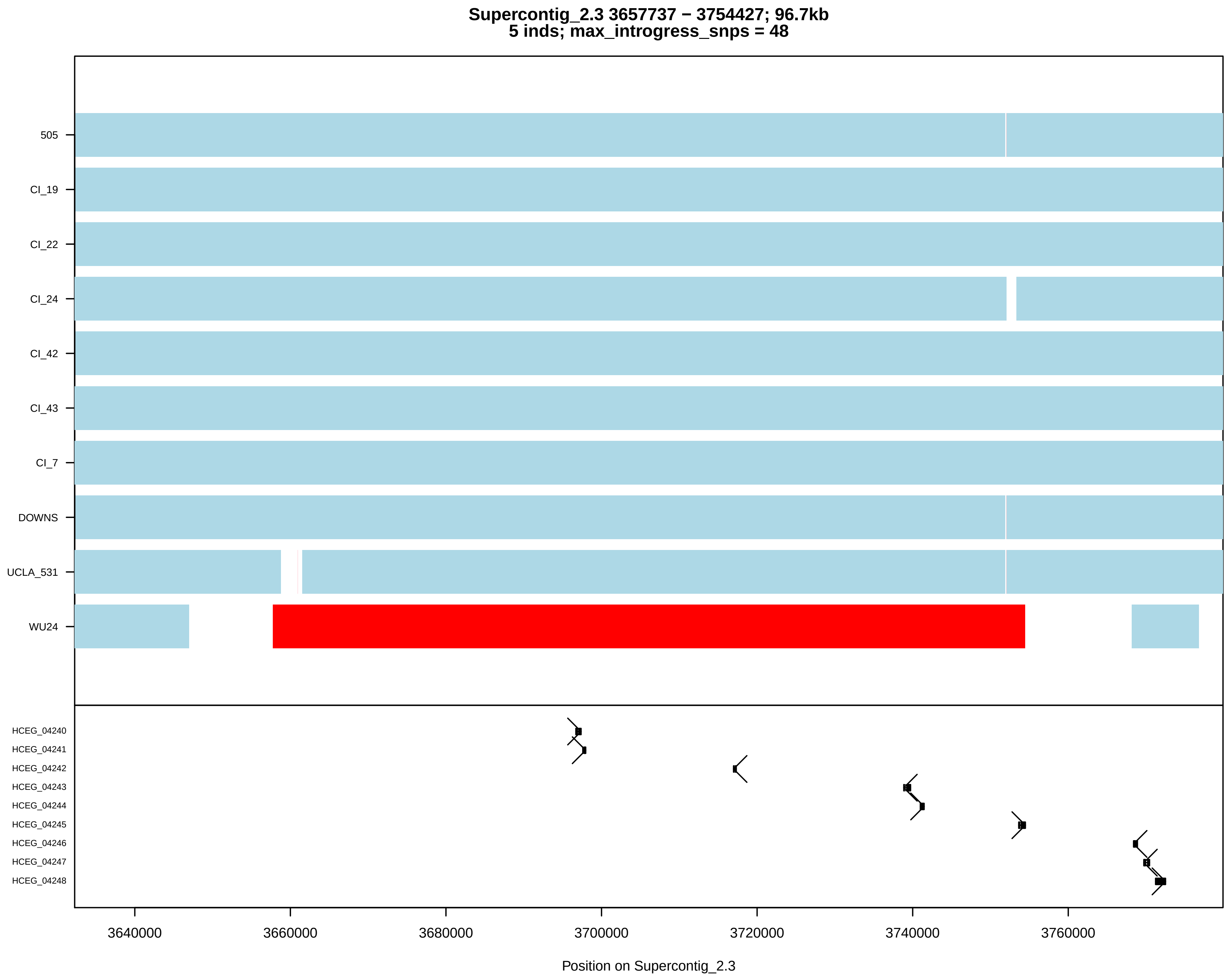
Supercontig_2.3 3657737 − 3754427; 96.7kb
5 inds; max_introgress_snps = 48
505
CI_19
CI_22
CI_24
CI_42
CI_43
CI_7
DOWNS
UCLA_531
WU24
HCEG_04240
HCEG_04241
HCEG_04242
HCEG_04243
HCEG_04244
HCEG_04245
HCEG_04246
HCEG_04247
HCEG_04248
3640000
3660000
3680000
3700000
3720000
3740000
3760000
Position on Supercontig_2.3

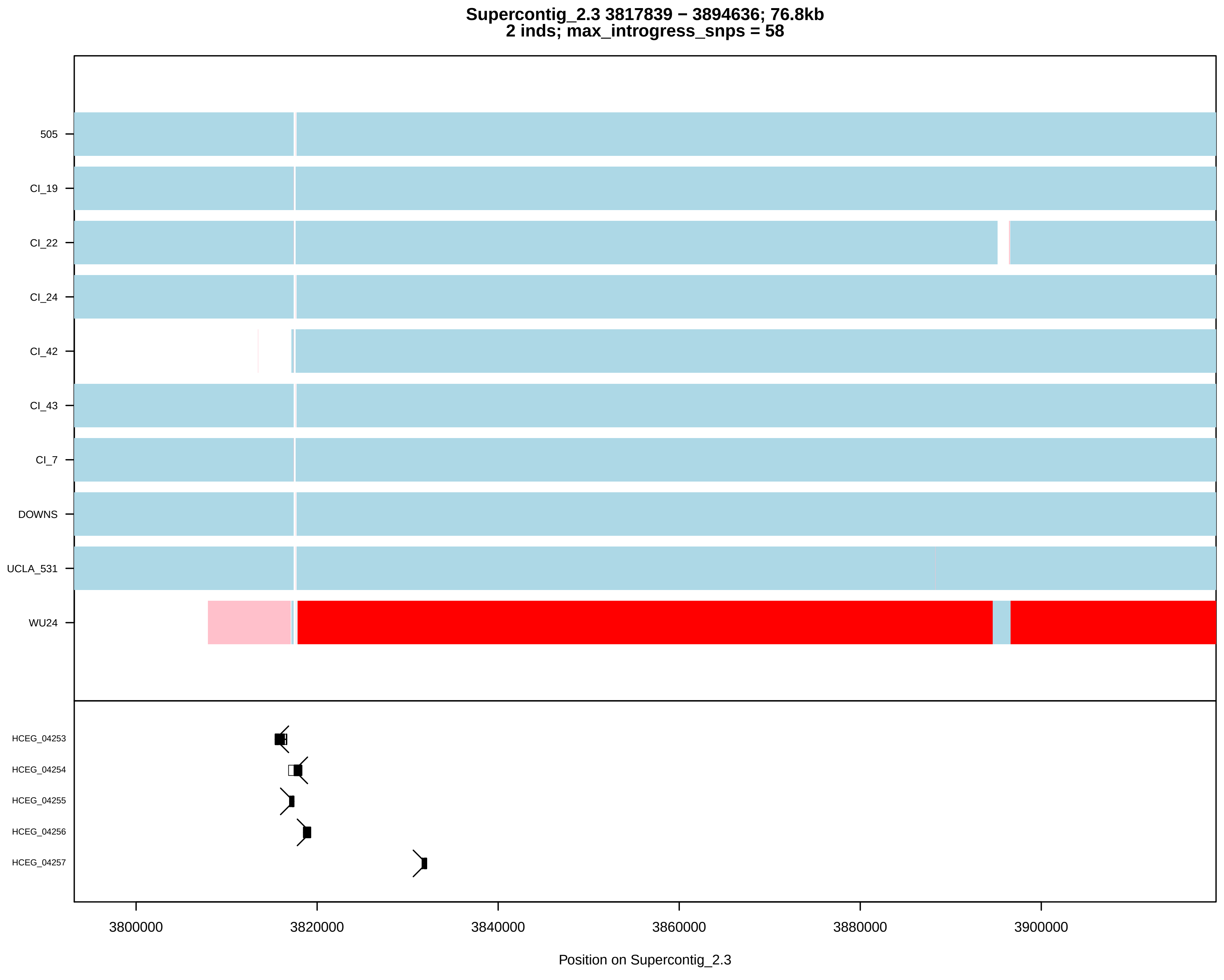

Supercontig_2.3 3817839 − 3894636; 76.8kb
2 inds; max_introgress_snps = 58
505
CI_19
CI_22
CI_24
CI_42
CI_43
CI_7
DOWNS
UCLA_531
WU24
HCEG_04253
HCEG_04254
HCEG_04255
HCEG_04256
HCEG_04257
3800000
3820000
3840000
3860000
3880000
3900000
Position on Supercontig_2.3

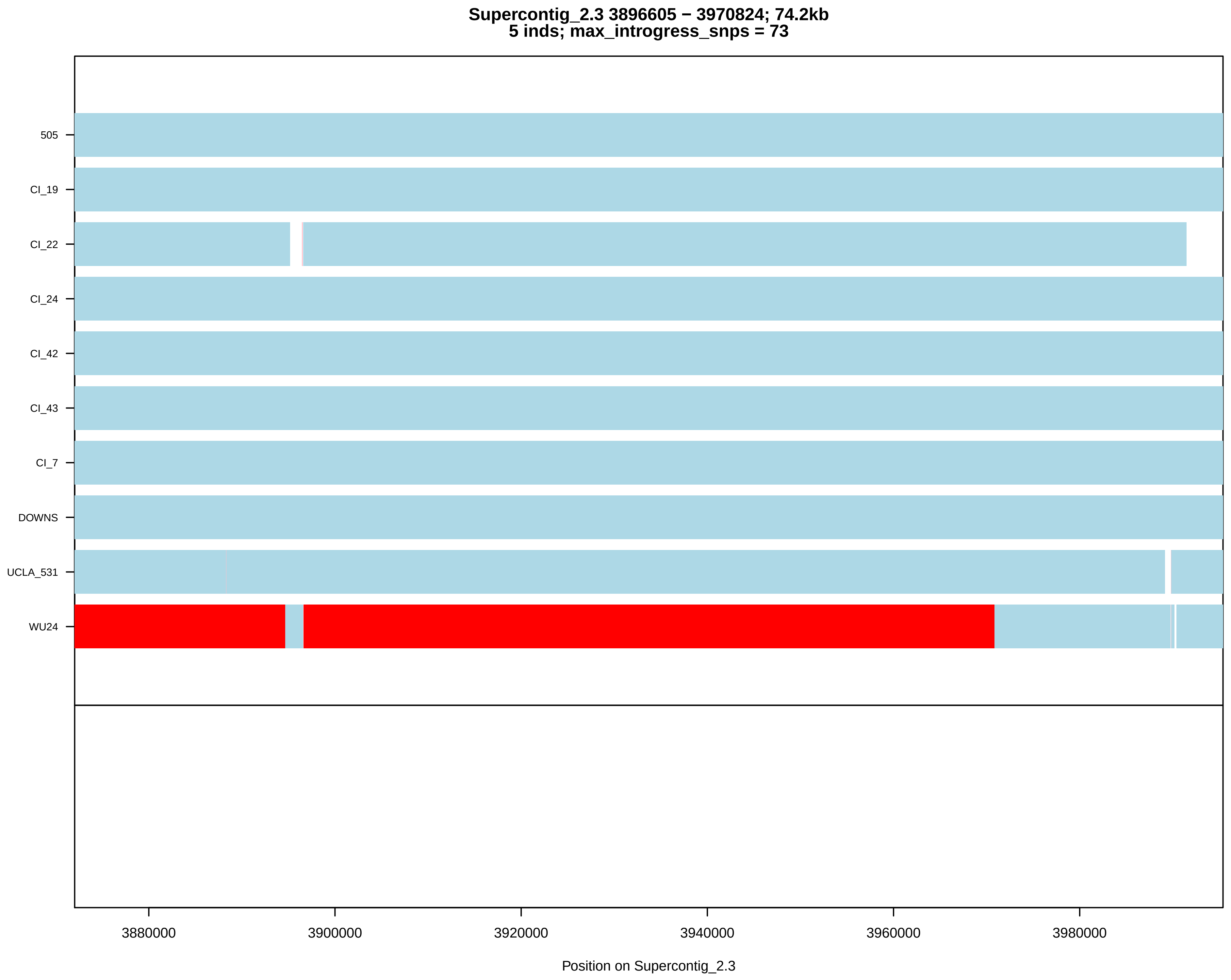
Supercontig_2.3 3896605 − 3970824; 74.2kb
5 inds; max_introgress_snps = 73
505
CI_19
CI_22
CI_24
CI_42
CI_43
CI_7
DOWNS
UCLA_531
WU24
3880000
3900000
3920000
3940000
3960000
3980000
Position on Supercontig_2.3

Supercontig_2.3 4080870 − 4117303; 36.4kb
2 inds; max_introgress_snps = 53

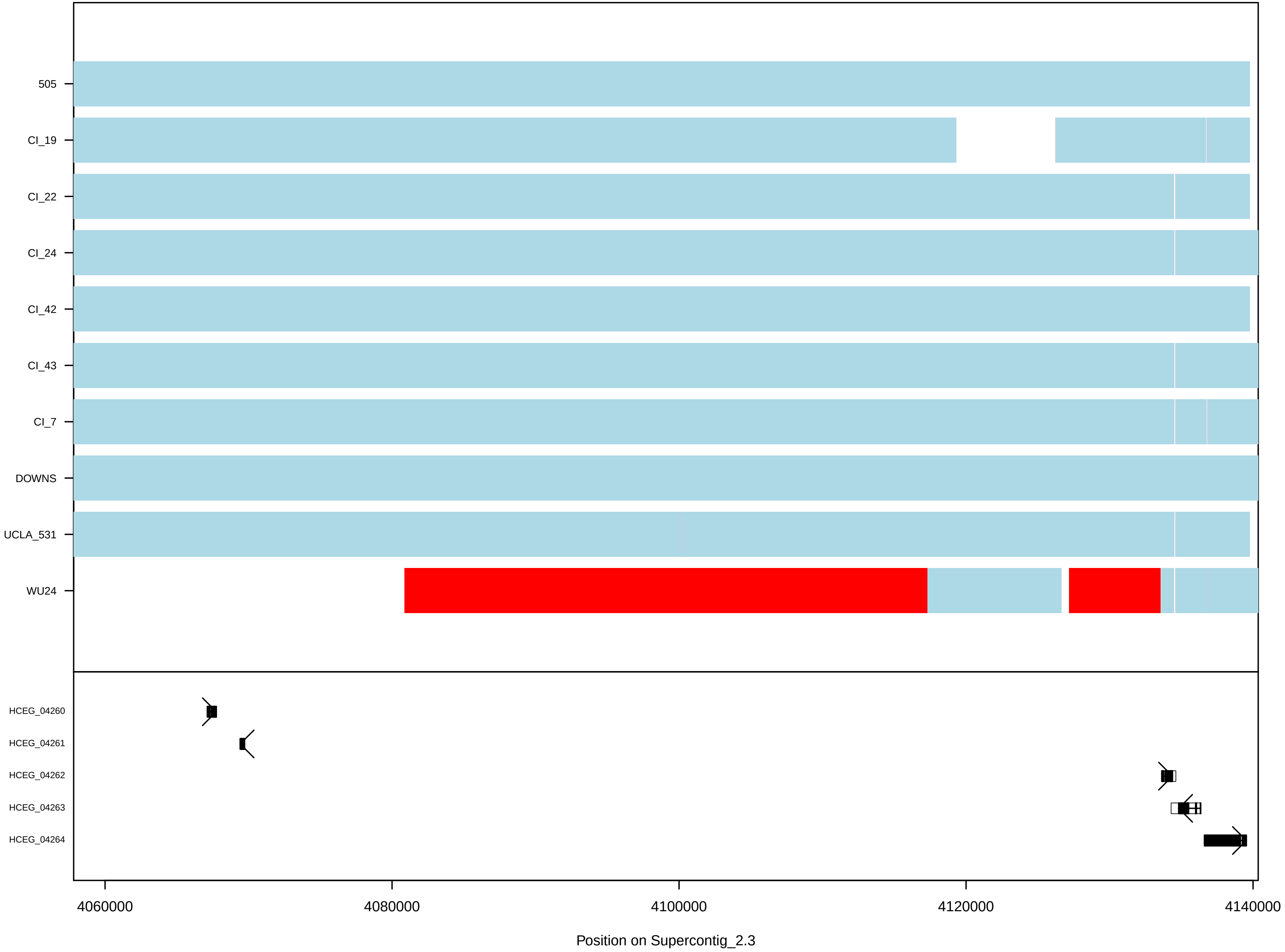

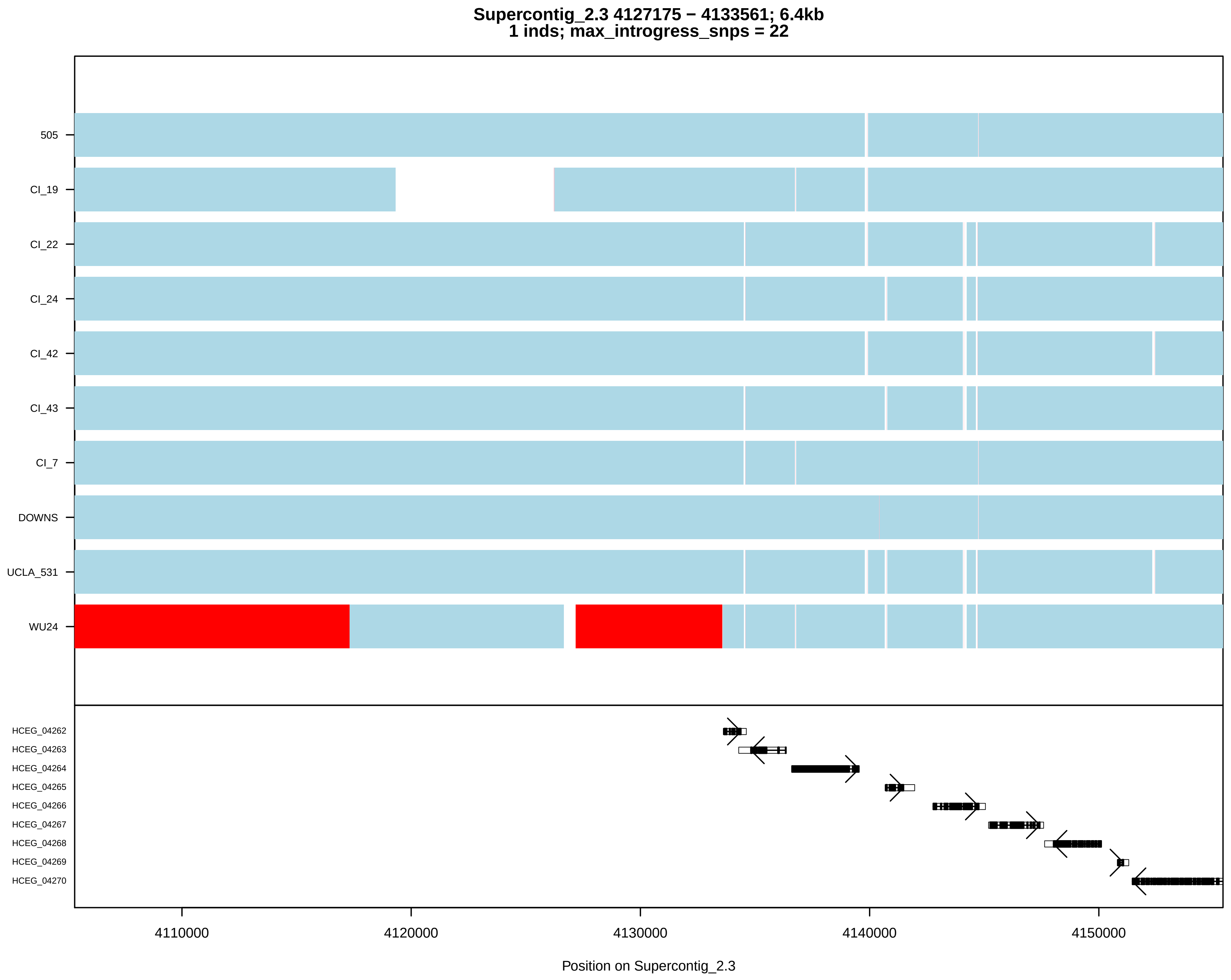

Supercontig_2.3 4127175 − 4133561; 6.4kb
1 inds; max_introgress_snps = 22
505
CI_19
CI_22
CI_24
CI_42
CI_43
CI_7
DOWNS
UCLA_531
WU24
HCEG_04262
HCEG_04263
HCEG_04264
HCEG_04265
HCEG_04266
HCEG_04267
HCEG_04268
HCEG_04269
HCEG_04270
4110000
4120000
4130000
4140000
4150000
Position on Supercontig_2.3

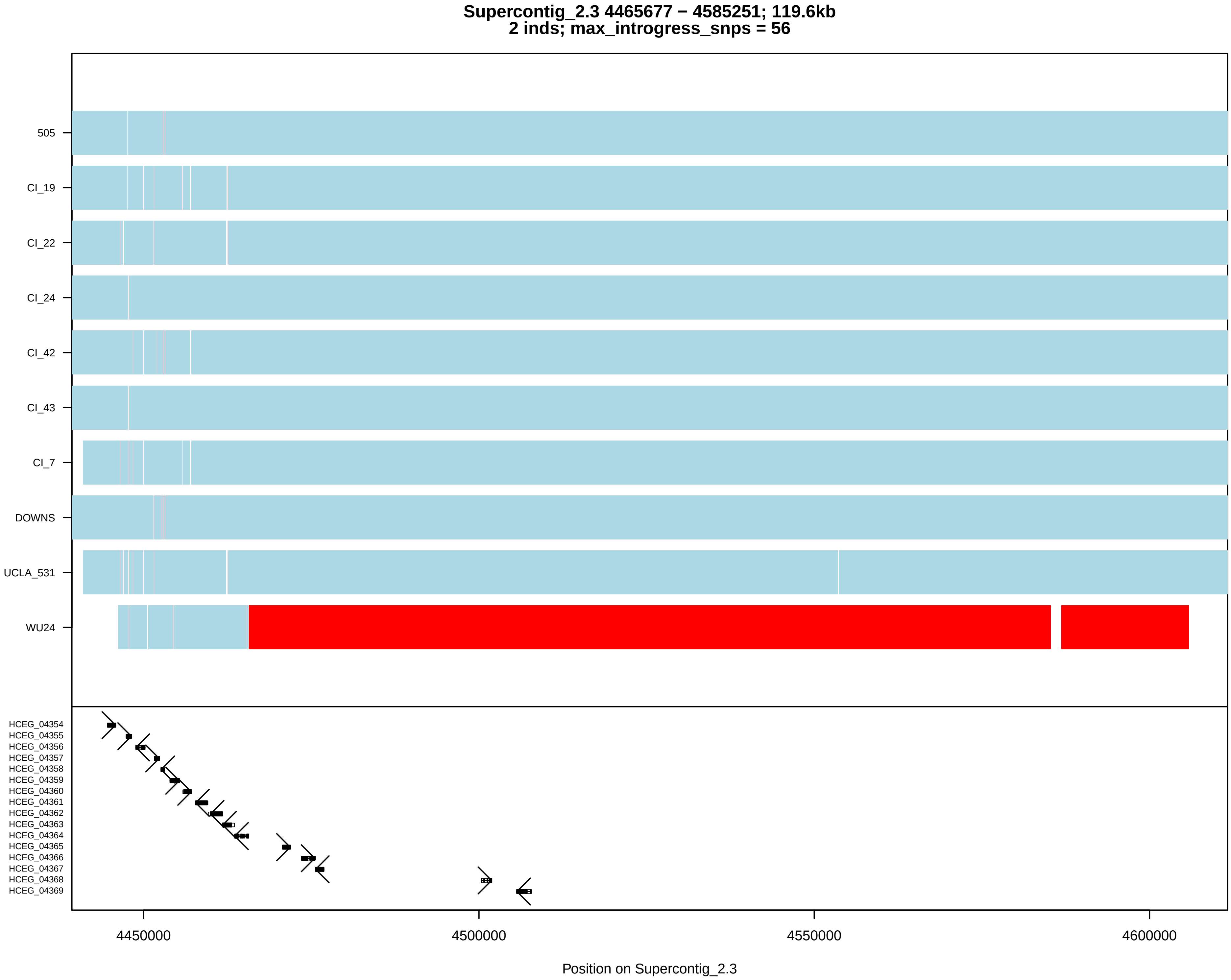
Supercontig_2.3 4465677 − 4585251; 119.6kb
2 inds; max_introgress_snps = 56
505
CI_19
CI_22
CI_24
CI_42
CI_43
CI_7
DOWNS
UCLA_531
WU24
HCEG_04354
HCEG_04355
HCEG_04356
HCEG_04357
HCEG_04358
HCEG_04359
HCEG_04360
HCEG_04361
HCEG_04362
HCEG_04363
HCEG_04364
HCEG_04365
HCEG_04366
HCEG_04367
HCEG_04368
HCEG_04369
4450000
4500000
4550000
4600000
Position on Supercontig_2.3

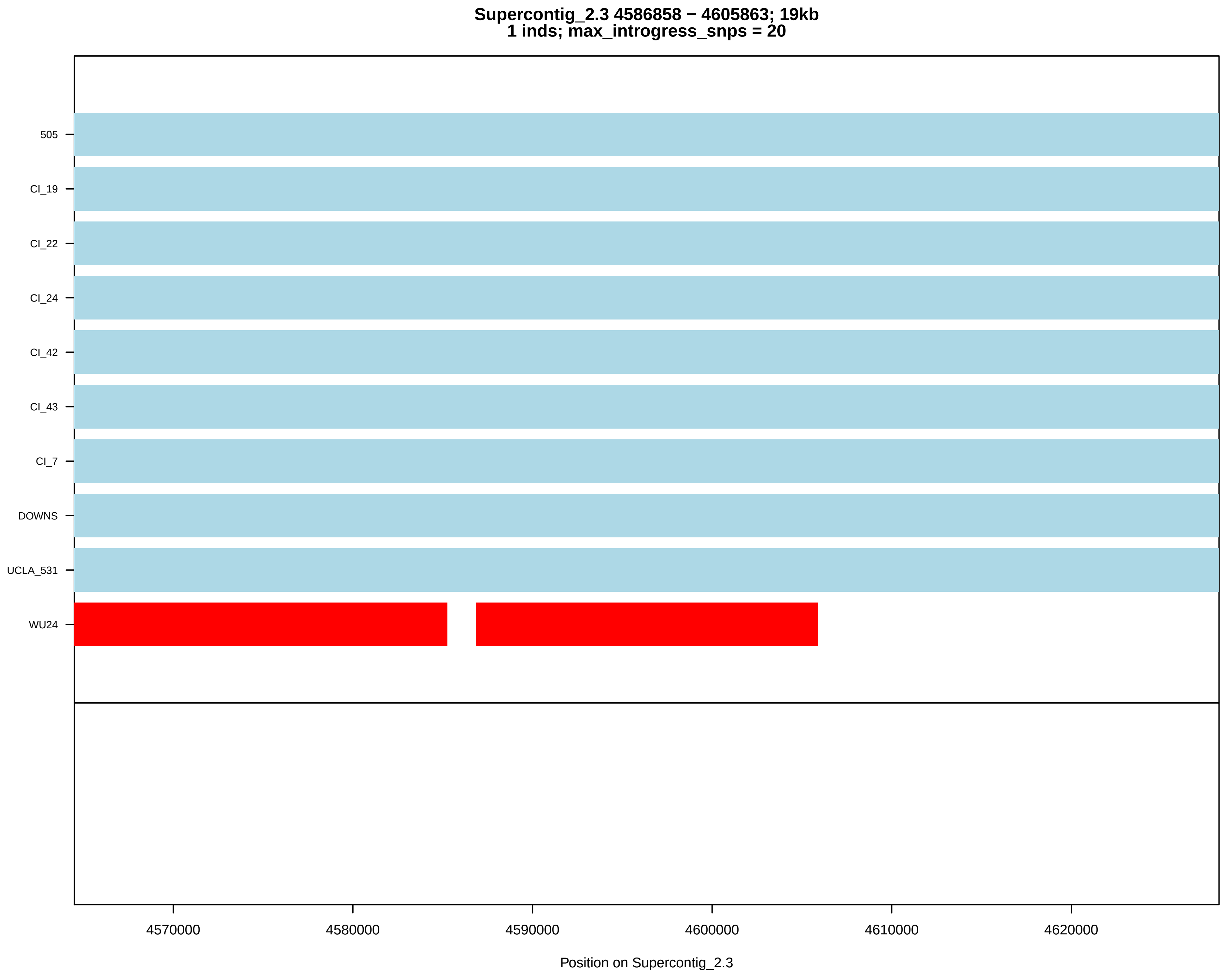
Supercontig_2.3 4586858 − 4605863; 19kb
1 inds; max_introgress_snps = 20
505
CI_19
CI_22
CI_24
CI_42
CI_43
CI_7
DOWNS
UCLA_531
WU24
4570000
4580000
4590000
4600000
4610000
4620000
Position on Supercontig_2.3

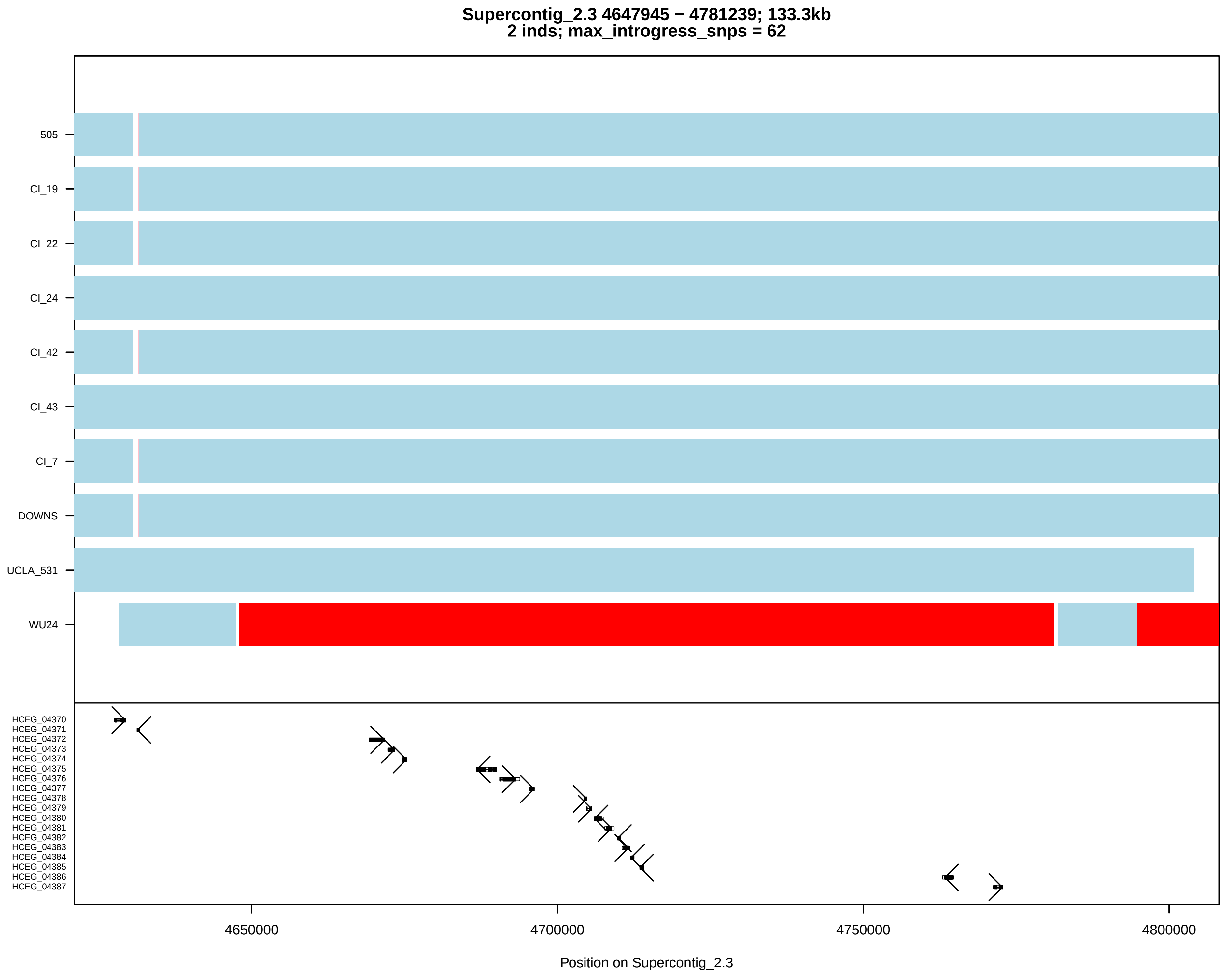
Supercontig_2.3 4647945 − 4781239; 133.3kb
2 inds; max_introgress_snps = 62
505
CI_19
CI_22
CI_24
CI_42
CI_43
CI_7
DOWNS
UCLA_531
WU24
HCEG_04370
HCEG_04371
HCEG_04372
HCEG_04373
HCEG_04374
HCEG_04375
HCEG_04376
HCEG_04377
HCEG_04378
HCEG_04379
HCEG_04380
HCEG_04381
HCEG_04382
HCEG_04383
HCEG_04384
HCEG_04385
HCEG_04386
HCEG_04387
4650000
4700000
4750000
4800000
Position on Supercontig_2.3

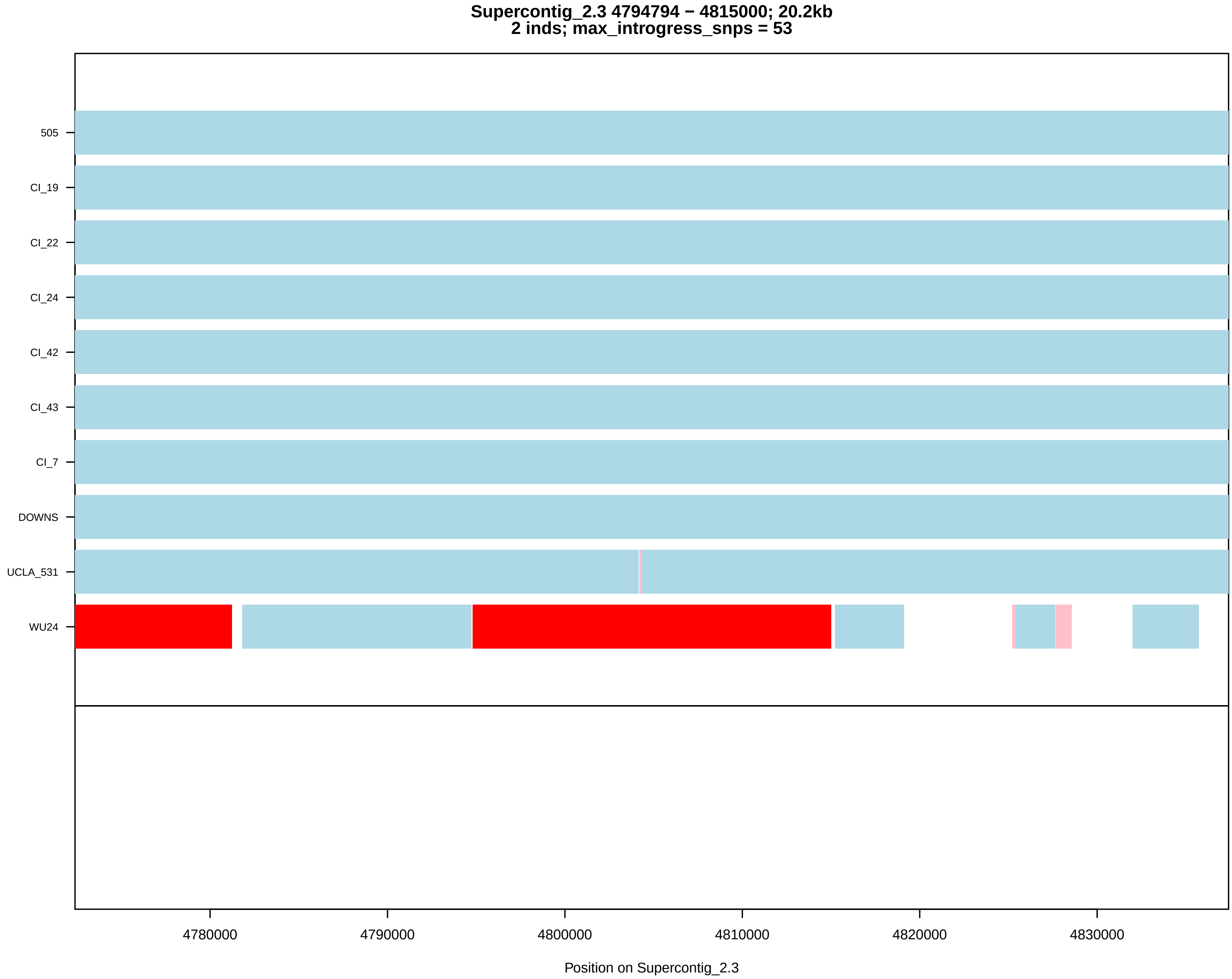
Supercontig_2.3 4794794 − 4815000; 20.2kb
2 inds; max_introgress_snps = 53
505
CI_19
CI_22
CI_24
CI_42
CI_43
CI_7
DOWNS
UCLA_531
WU24
4780000
4790000
4800000
4810000
4820000
4830000
Position on Supercontig_2.3

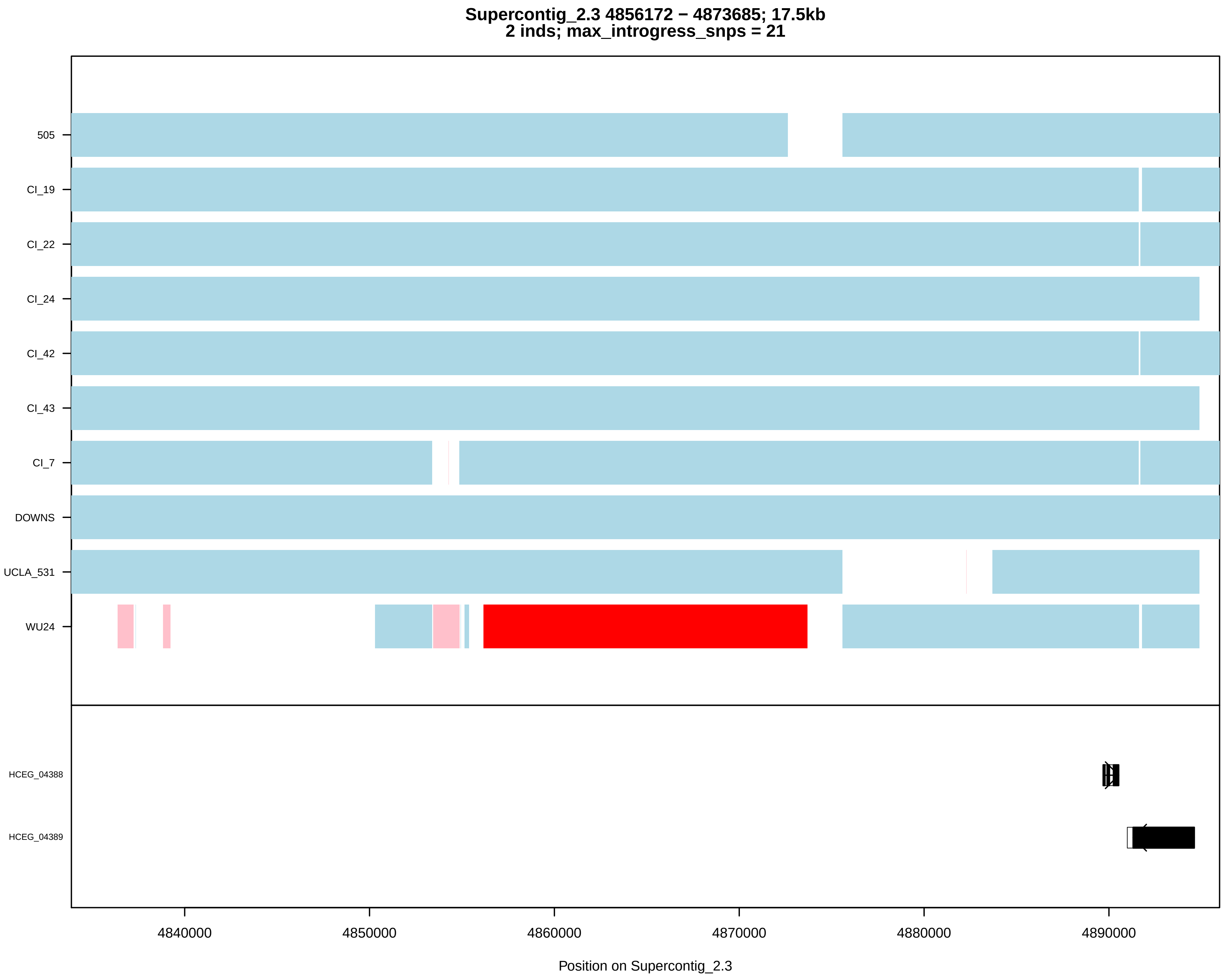

Supercontig_2.3 4856172 − 4873685; 17.5kb
2 inds; max_introgress_snps = 21
505
CI_19
CI_22
CI_24
CI_42
CI_43
CI_7
DOWNS
UCLA_531
WU24
HCEG_04388
HCEG_04389
4840000
4850000
4860000
4870000
4880000
4890000
Position on Supercontig_2.3

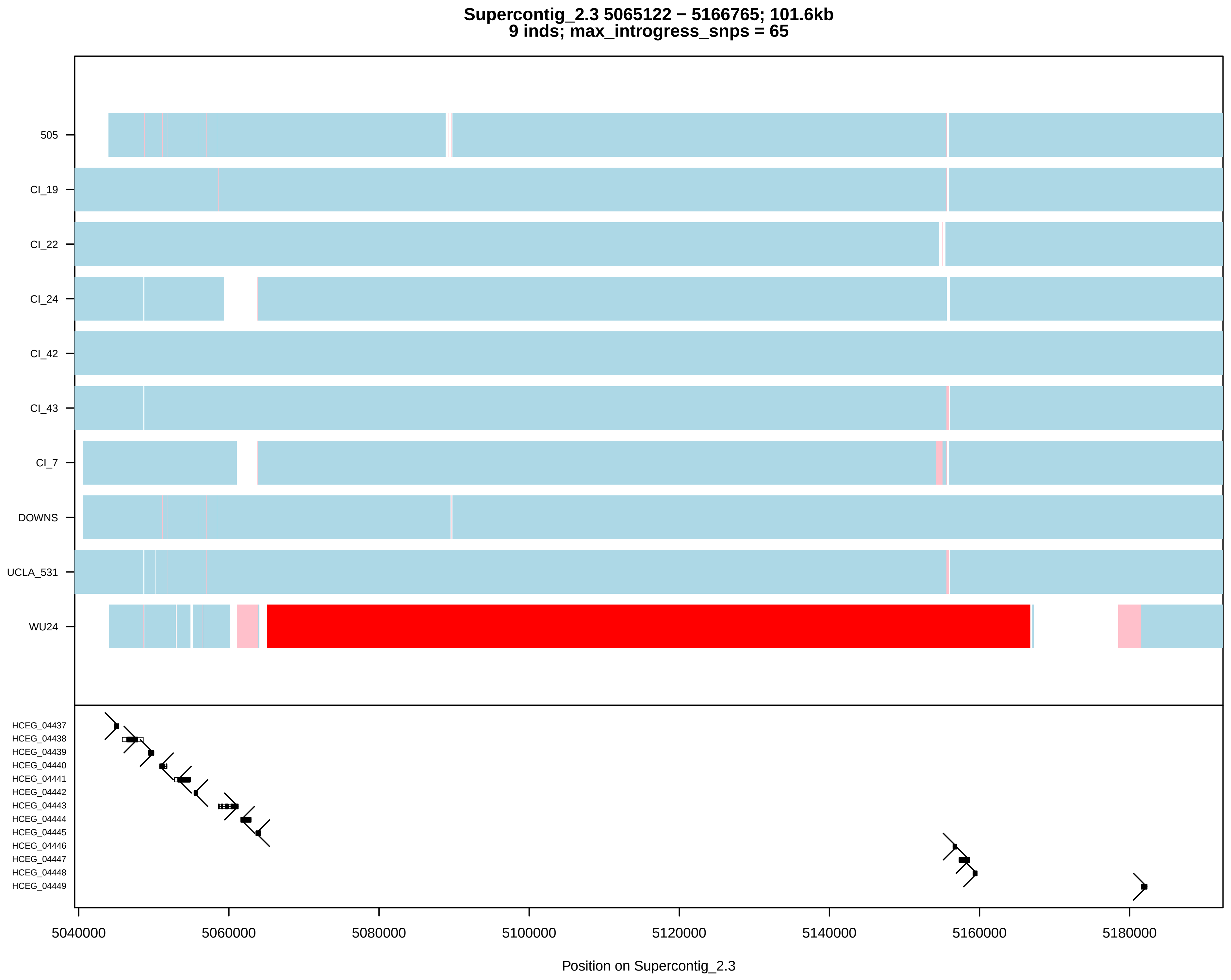
Supercontig_2.3 5065122 − 5166765; 101.6kb
9 inds; max_introgress_snps = 65
505
CI_19
CI_22
CI_24
CI_42
CI_43
CI_7
DOWNS
UCLA_531
WU24
HCEG_04437
HCEG_04438
HCEG_04439
HCEG_04440
HCEG_04441
HCEG_04442
HCEG_04443
HCEG_04444
HCEG_04445
HCEG_04446
HCEG_04447
HCEG_04448
HCEG_04449
5040000
5060000
5080000
5100000
5120000
5140000
5160000
5180000
Position on Supercontig_2.3

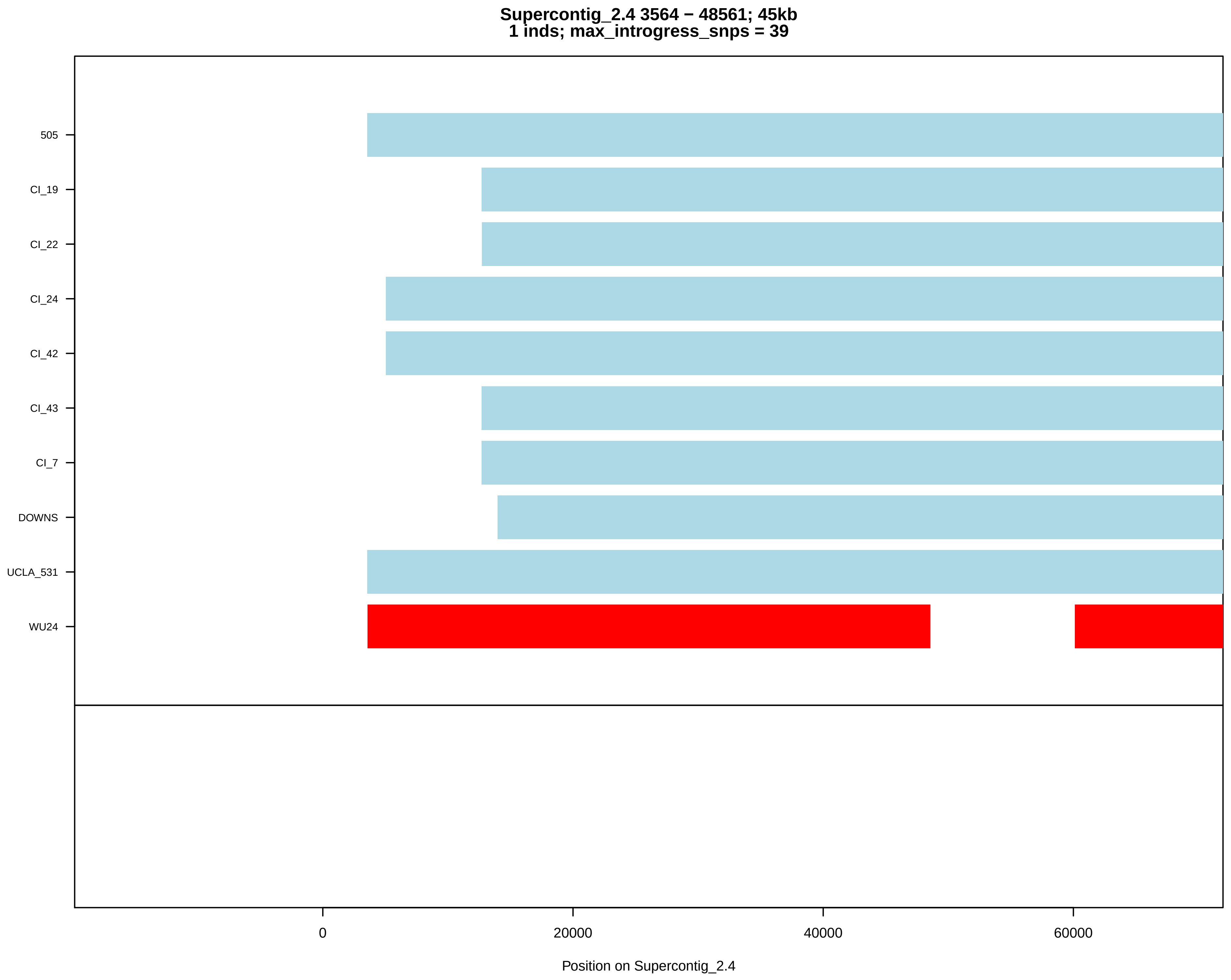
Supercontig_2.4 3564 − 48561; 45kb
1 inds; max_introgress_snps = 39
505
CI_19
CI_22
CI_24
CI_42
CI_43
CI_7
DOWNS
UCLA_531
WU24
0
20000
40000
60000
Position on Supercontig_2.4

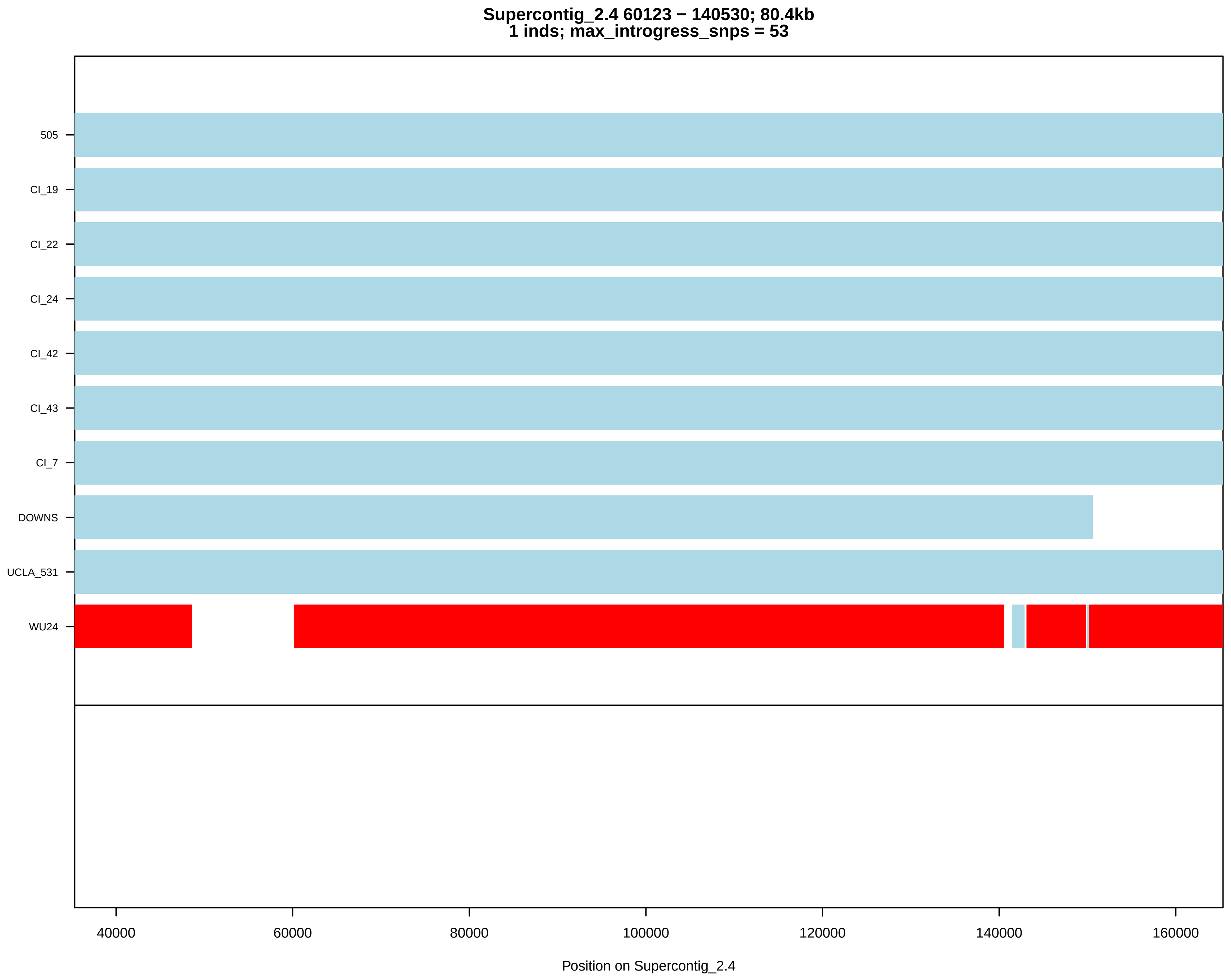
Supercontig_2.4 60123 − 140530; 80.4kb
1 inds; max_introgress_snps = 53
505
CI_19
CI_22
CI_24
CI_42
CI_43
CI_7
DOWNS
UCLA_531
WU24
40000
60000
80000
100000
120000
140000
160000
Position on Supercontig_2.4

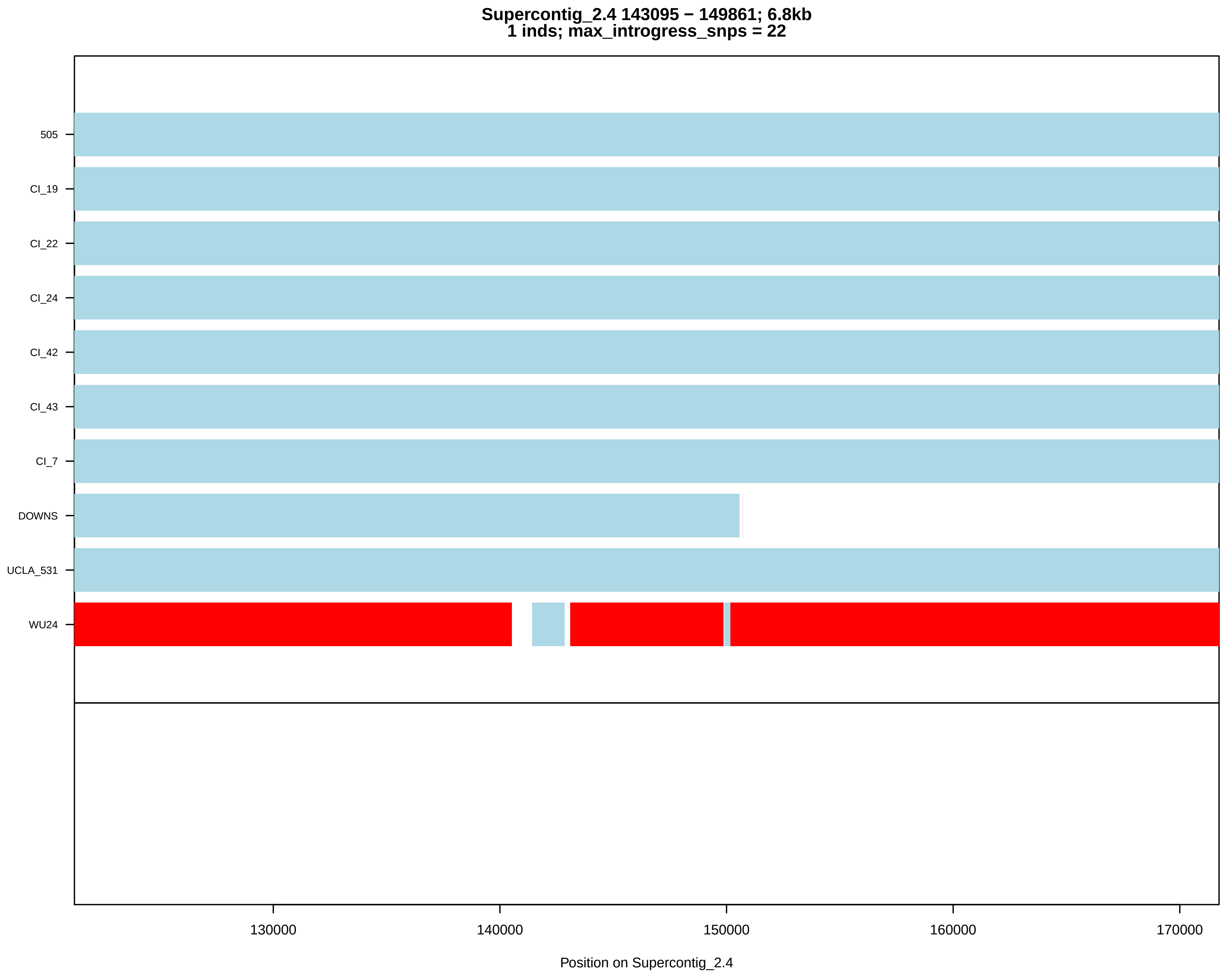
Supercontig_2.4 143095 − 149861; 6.8kb
1 inds; max_introgress_snps = 22
505
CI_19
CI_22
CI_24
CI_42
CI_43
CI_7
DOWNS
UCLA_531
WU24
130000
140000
150000
160000
170000
Position on Supercontig_2.4

Supercontig_2.4 150172 − 192429; 42.3kb
4 inds; max_introgress_snps = 46

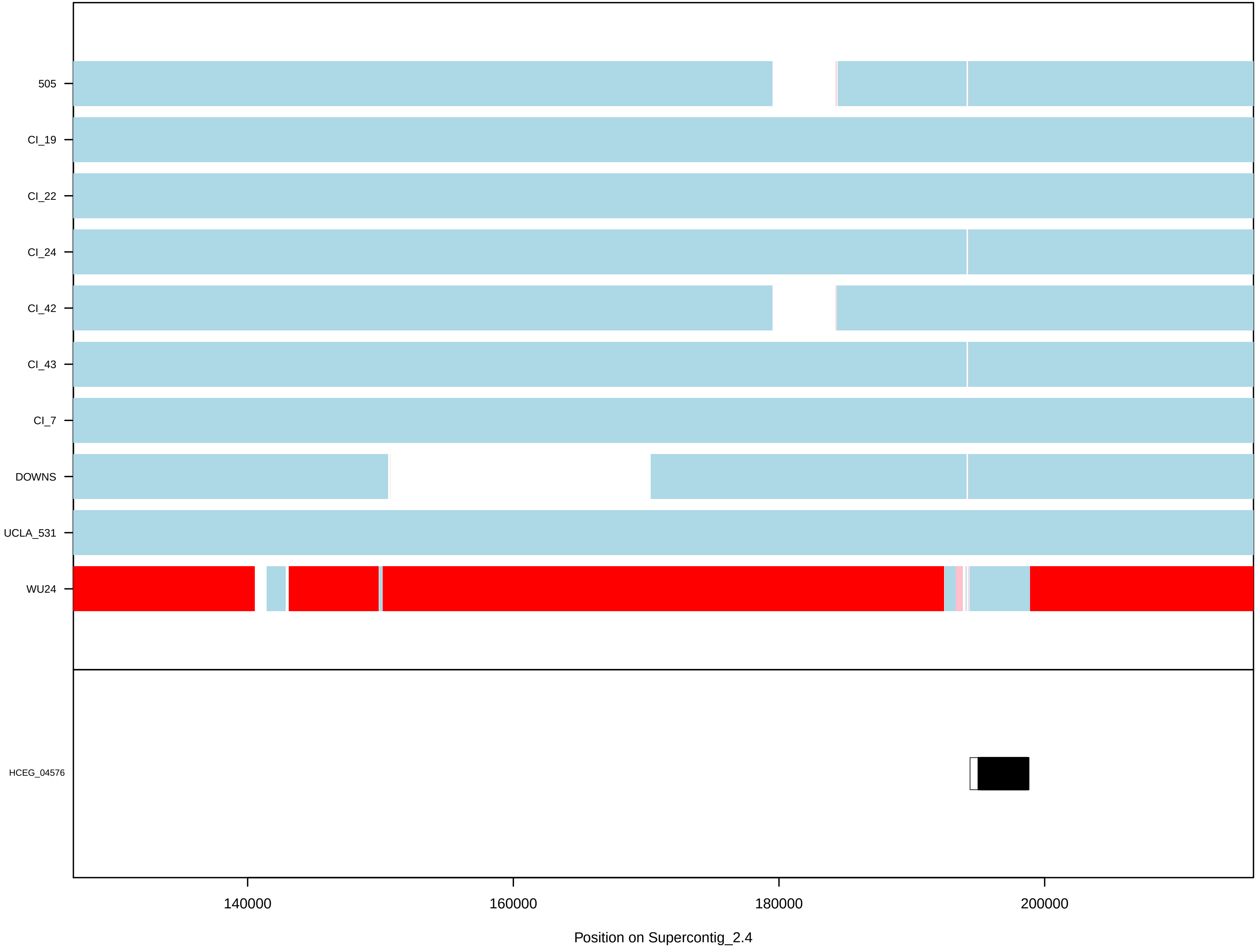

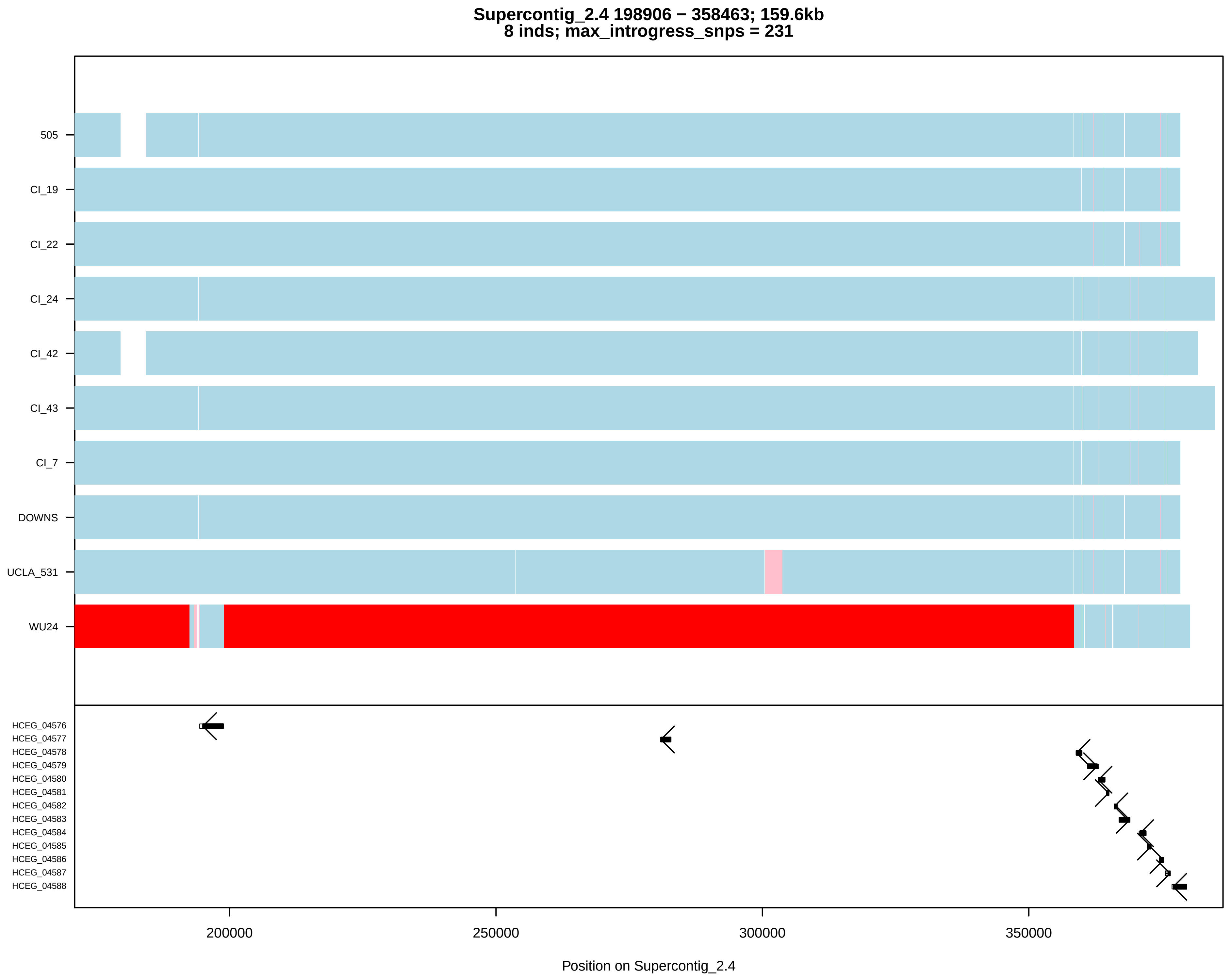
Supercontig_2.4 198906 − 358463; 159.6kb
8 inds; max_introgress_snps = 231
505
CI_19
CI_22
CI_24
CI_42
CI_43
CI_7
DOWNS
UCLA_531
WU24
HCEG_04576
HCEG_04577
HCEG_04578
HCEG_04579
HCEG_04580
HCEG_04581
HCEG_04582
HCEG_04583
HCEG_04584
HCEG_04585
HCEG_04586
HCEG_04587
HCEG_04588
200000
250000
300000
350000
Position on Supercontig_2.4

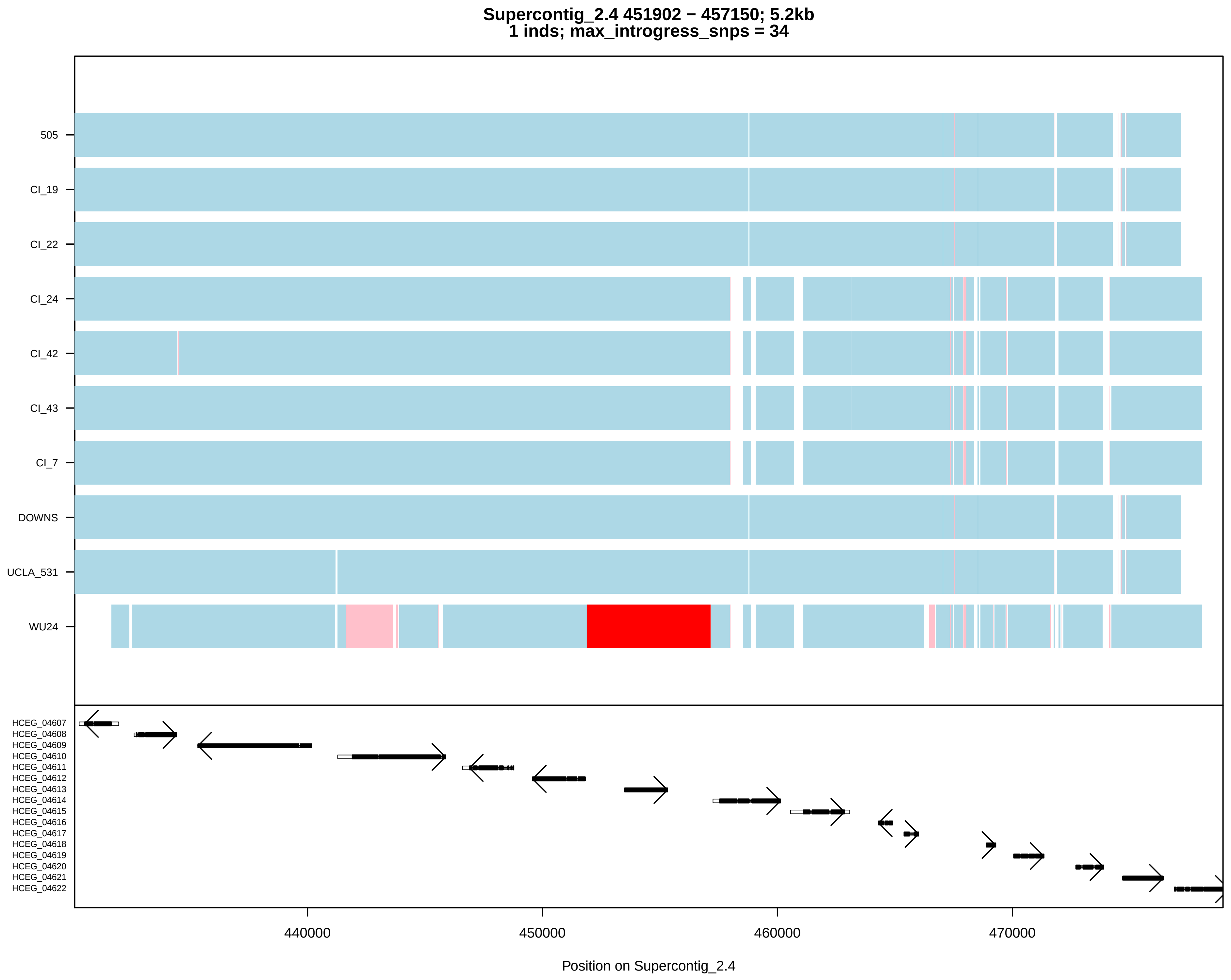

Supercontig_2.4 451902 − 457150; 5.2kb
1 inds; max_introgress_snps = 34
505
CI_19
CI_22
CI_24
CI_42
CI_43
CI_7
DOWNS
UCLA_531
WU24
HCEG_04607
HCEG_04608
HCEG_04609
HCEG_04610
HCEG_04611
HCEG_04612
HCEG_04613
HCEG_04614
HCEG_04615
HCEG_04616
HCEG_04617
HCEG_04618
HCEG_04619
HCEG_04620
HCEG_04621
HCEG_04622
440000
450000
460000
470000
Position on Supercontig_2.4

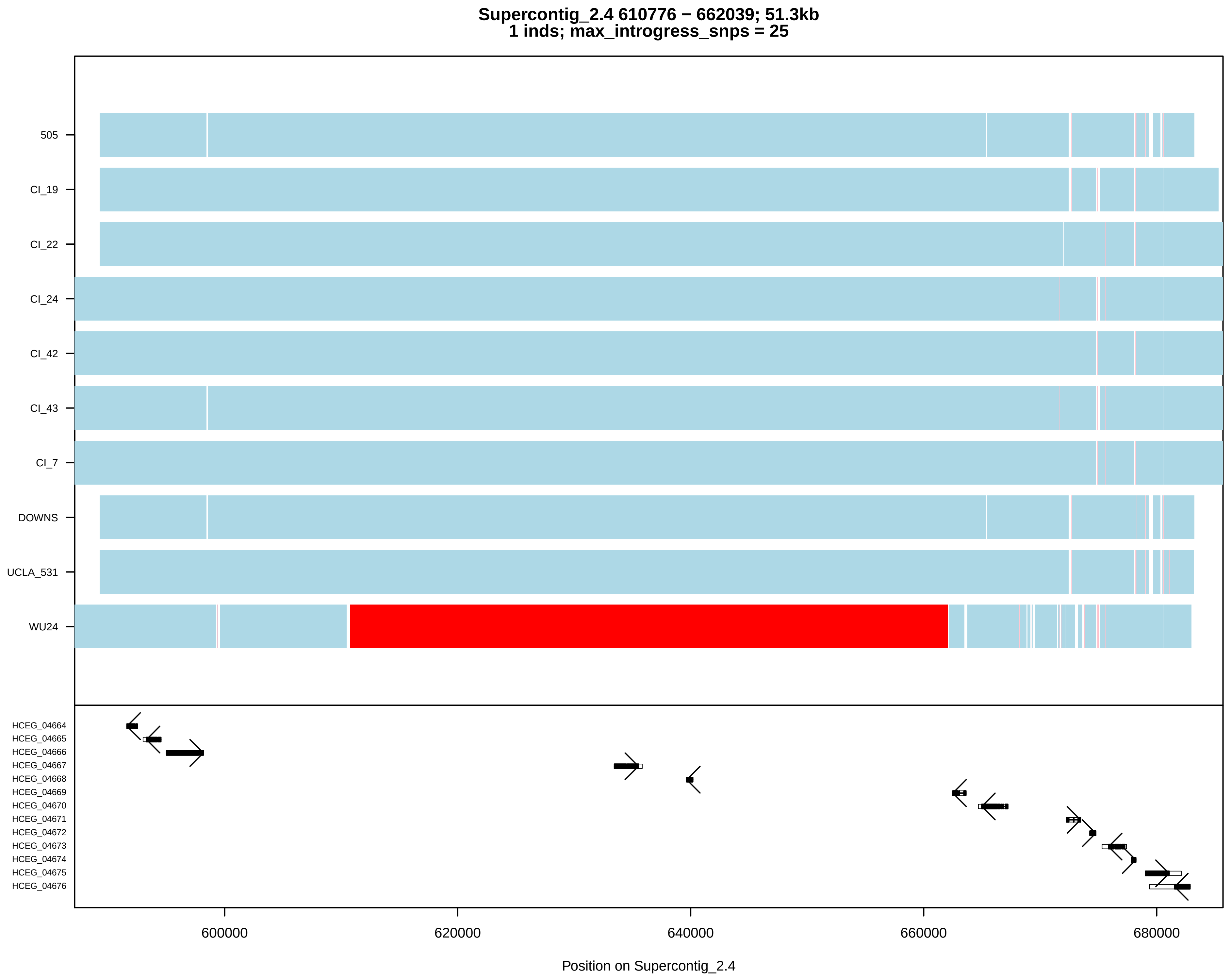

Supercontig_2.4 610776 − 662039; 51.3kb
1 inds; max_introgress_snps = 25
505
CI_19
CI_22
CI_24
CI_42
CI_43
CI_7
DOWNS
UCLA_531
WU24
HCEG_04664
HCEG_04665
HCEG_04666
HCEG_04667
HCEG_04668
HCEG_04669
HCEG_04670
HCEG_04671
HCEG_04672
HCEG_04673
HCEG_04674
HCEG_04675
HCEG_04676
600000
620000
640000
660000
680000
Position on Supercontig_2.4

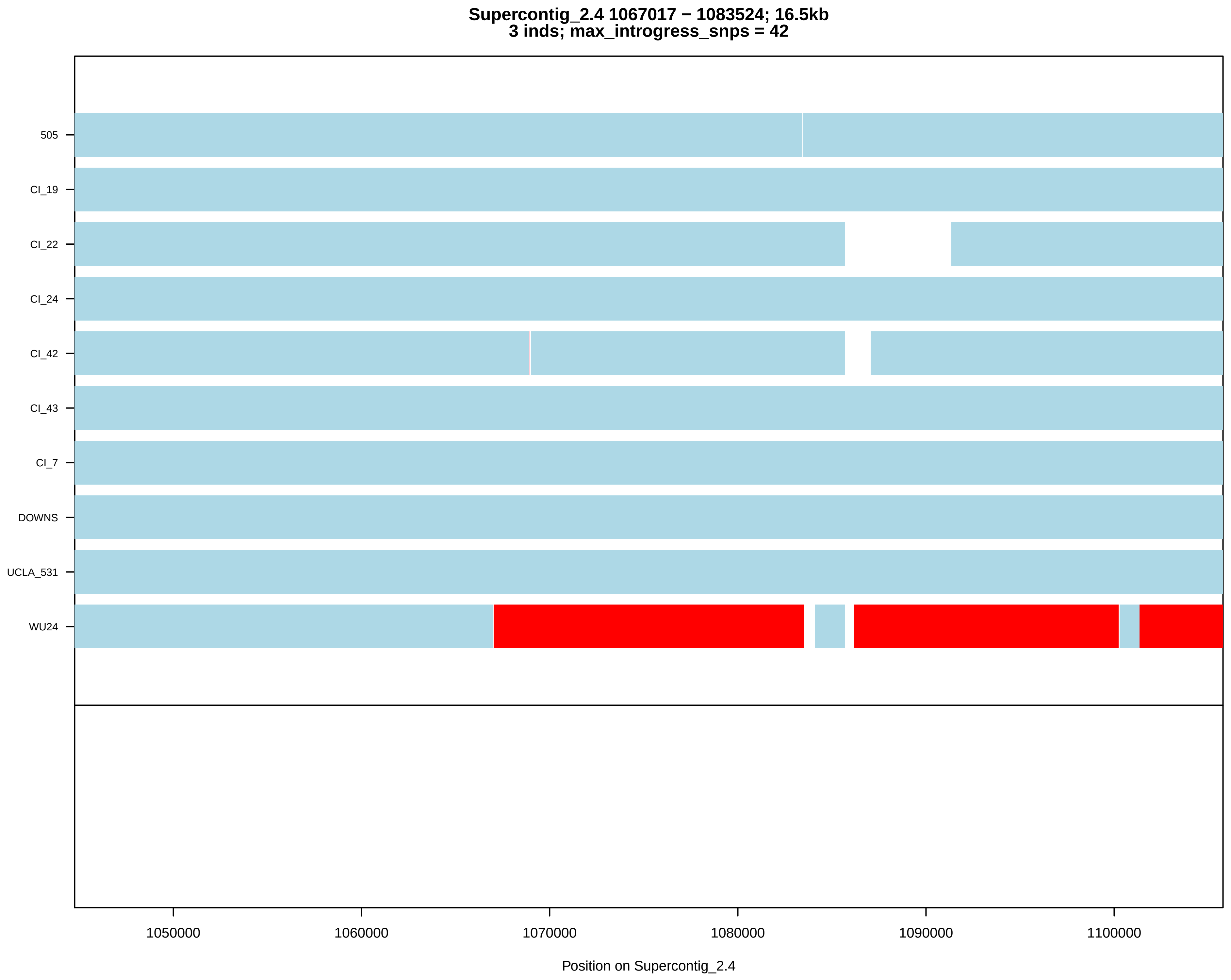
Supercontig_2.4 1067017 − 1083524; 16.5kb
3 inds; max_introgress_snps = 42
505
CI_19
CI_22
CI_24
CI_42
CI_43
CI_7
DOWNS
UCLA_531
WU24
1050000
1060000
1070000
1080000
1090000
1100000
Position on Supercontig_2.4

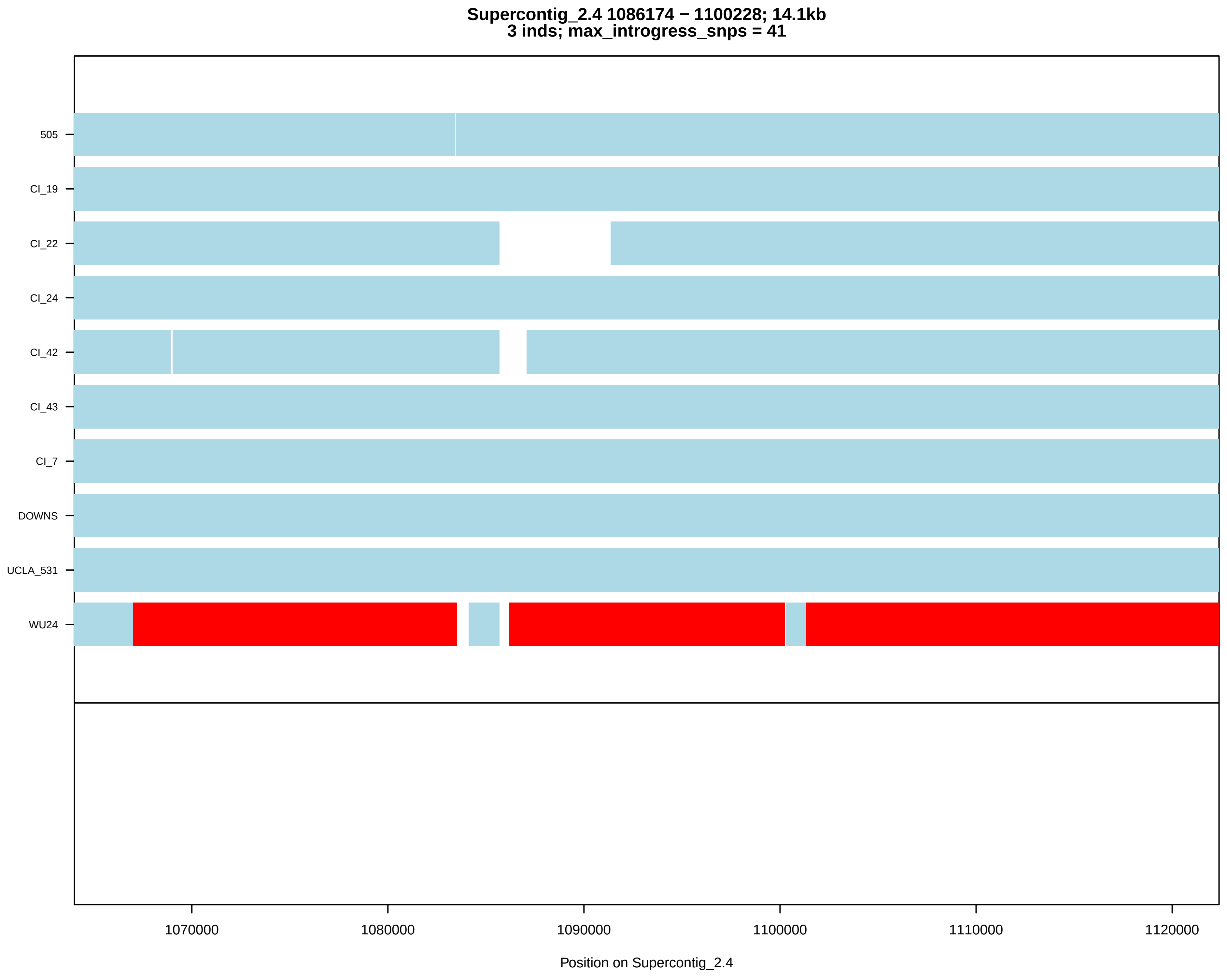

Supercontig_2.4 1086174 − 1100228; 14.1kb
3 inds; max_introgress_snps = 41
505
CI_19
CI_22
CI_24
CI_42
CI_43
CI_7
DOWNS
UCLA_531
WU24
1070000
1080000
1090000
1100000
1110000
1120000
Position on Supercontig_2.4

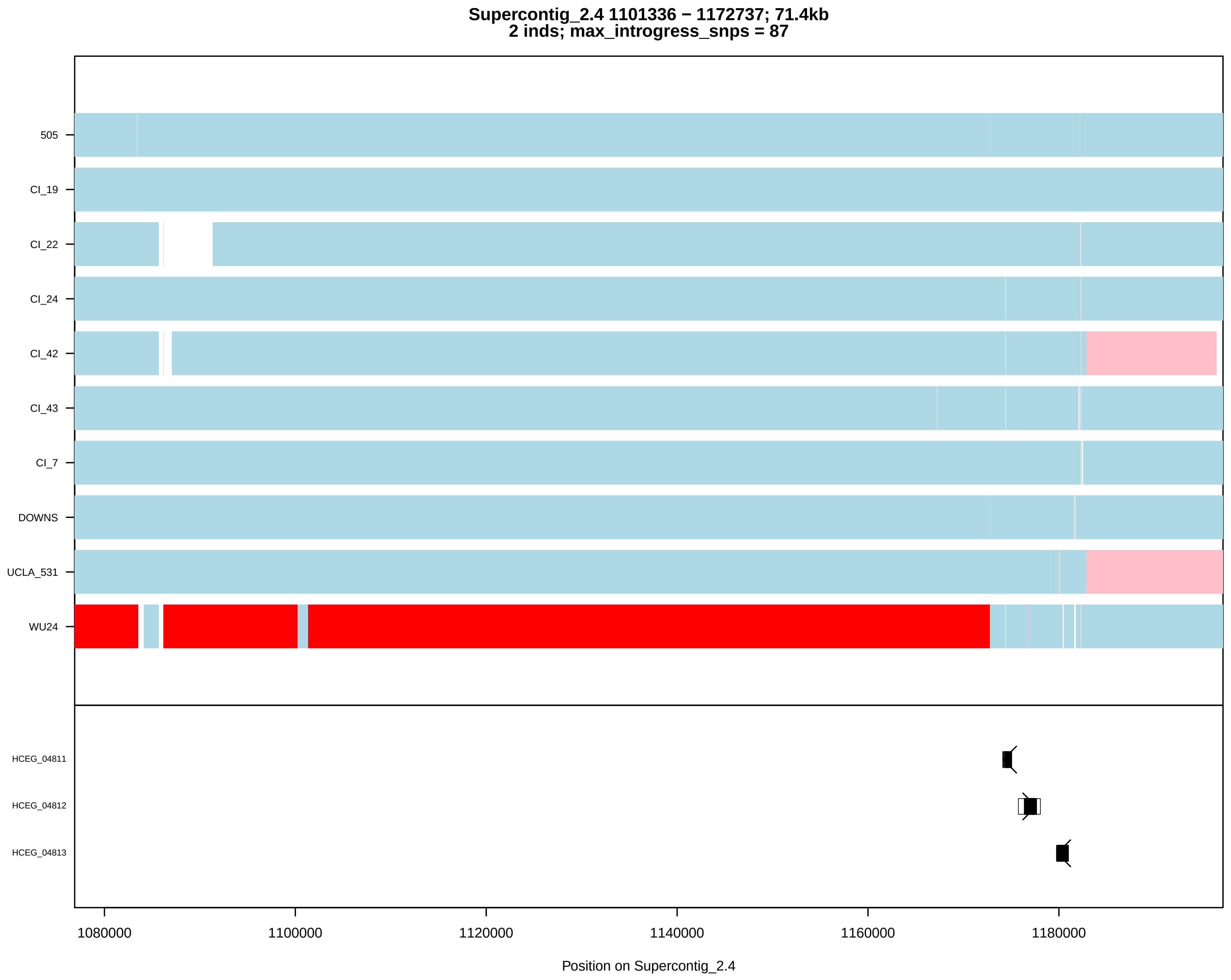
Supercontig_2.4 1101336 − 1172737; 71.4kb
2 inds; max_introgress_snps = 87
505
CI_19
CI_22
CI_24
CI_42
CI_43
CI_7
DOWNS
UCLA_531
WU24
HCEG_04811
HCEG_04812
HCEG_04813
1080000
1100000
1120000
1140000
1160000
1180000
Position on Supercontig_2.4

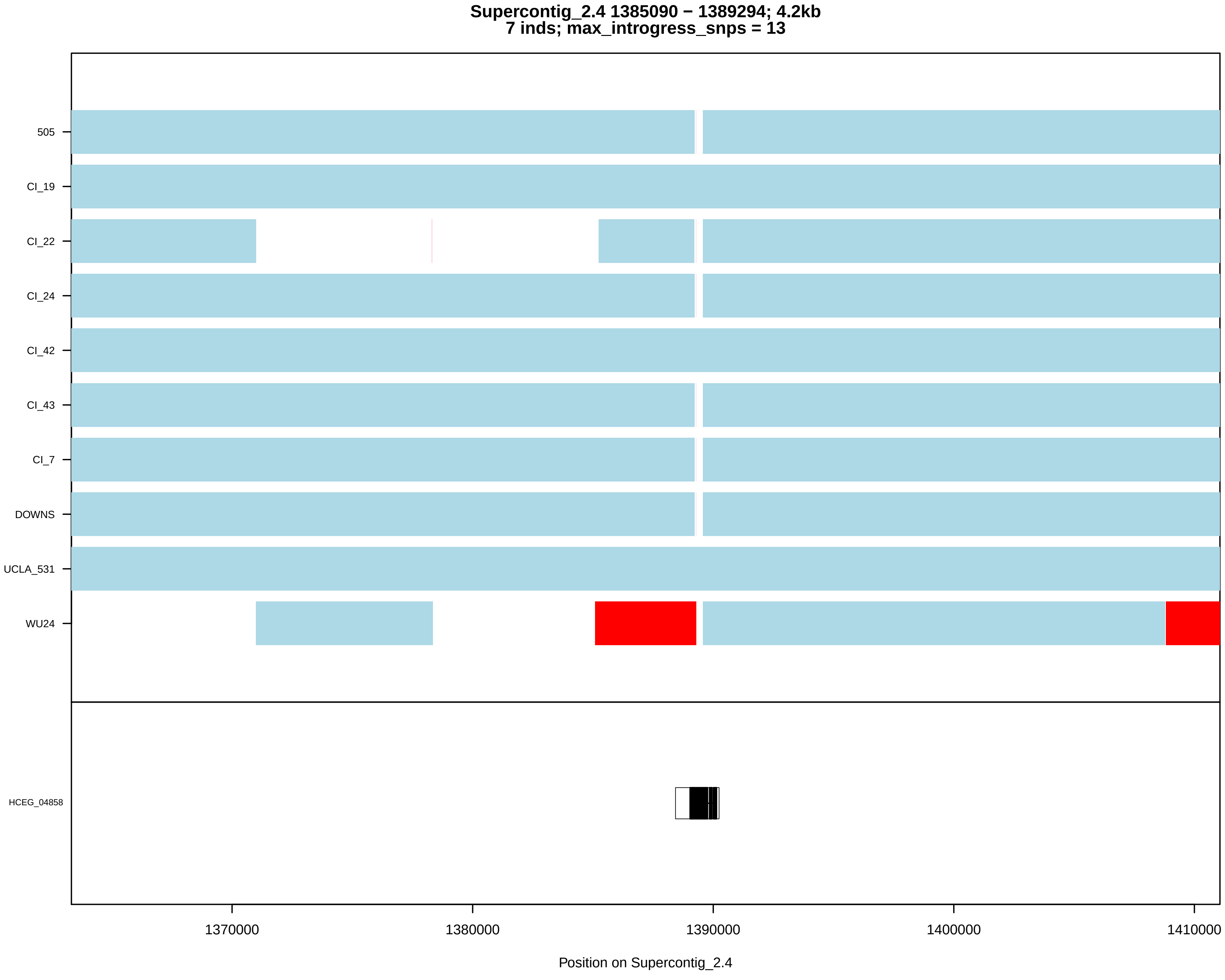

Supercontig_2.4 1385090 − 1389294; 4.2kb
7 inds; max_introgress_snps = 13
505
CI_19
CI_22
CI_24
CI_42
CI_43
CI_7
DOWNS
UCLA_531
WU24
HCEG_04858
1370000
1380000
1390000
1400000
1410000
Position on Supercontig_2.4

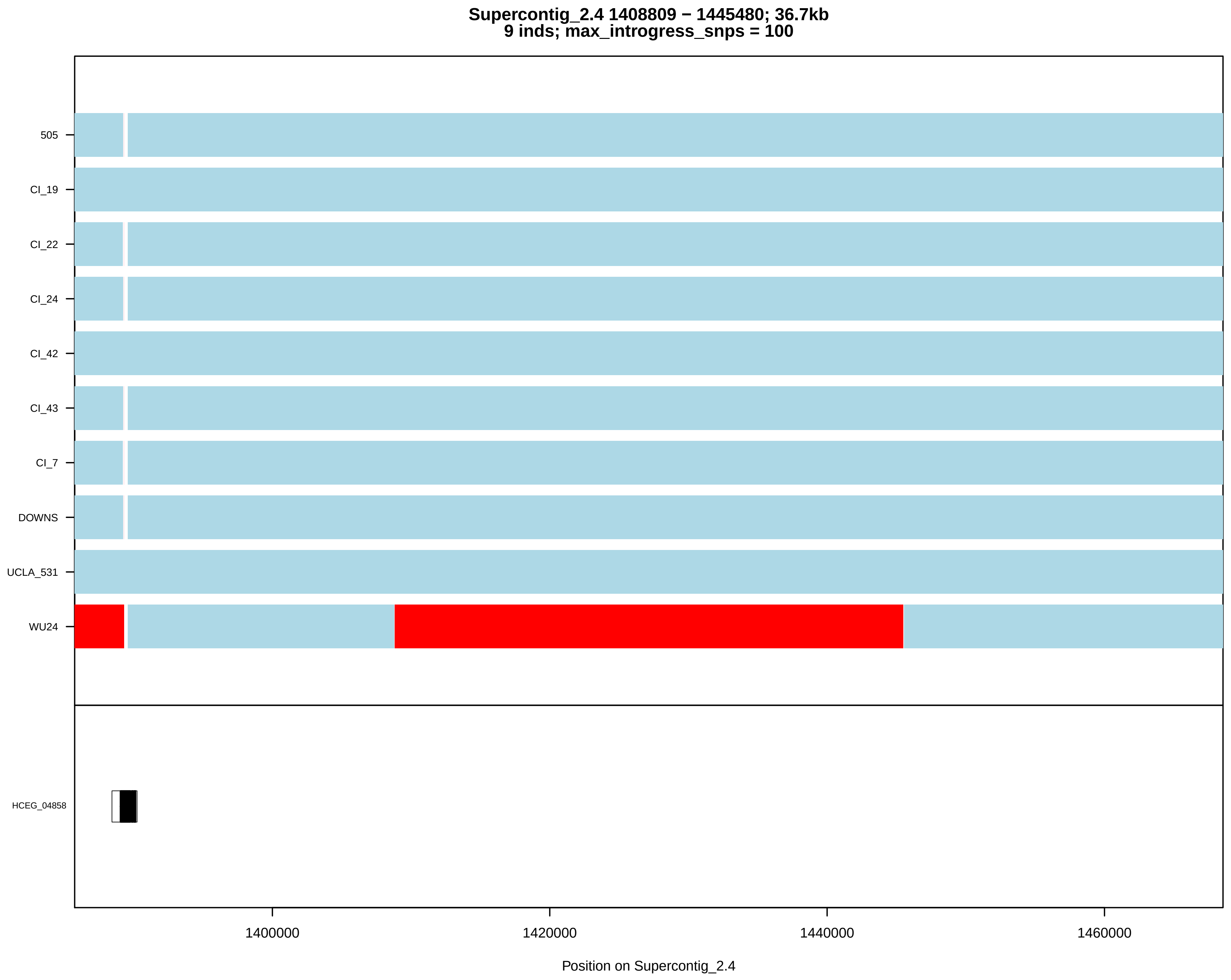

Supercontig_2.4 1408809 − 1445480; 36.7kb
9 inds; max_introgress_snps = 100
505
CI_19
CI_22
CI_24
CI_42
CI_43
CI_7
DOWNS
UCLA_531
WU24
HCEG_04858
1400000
1420000
1440000
1460000
Position on Supercontig_2.4

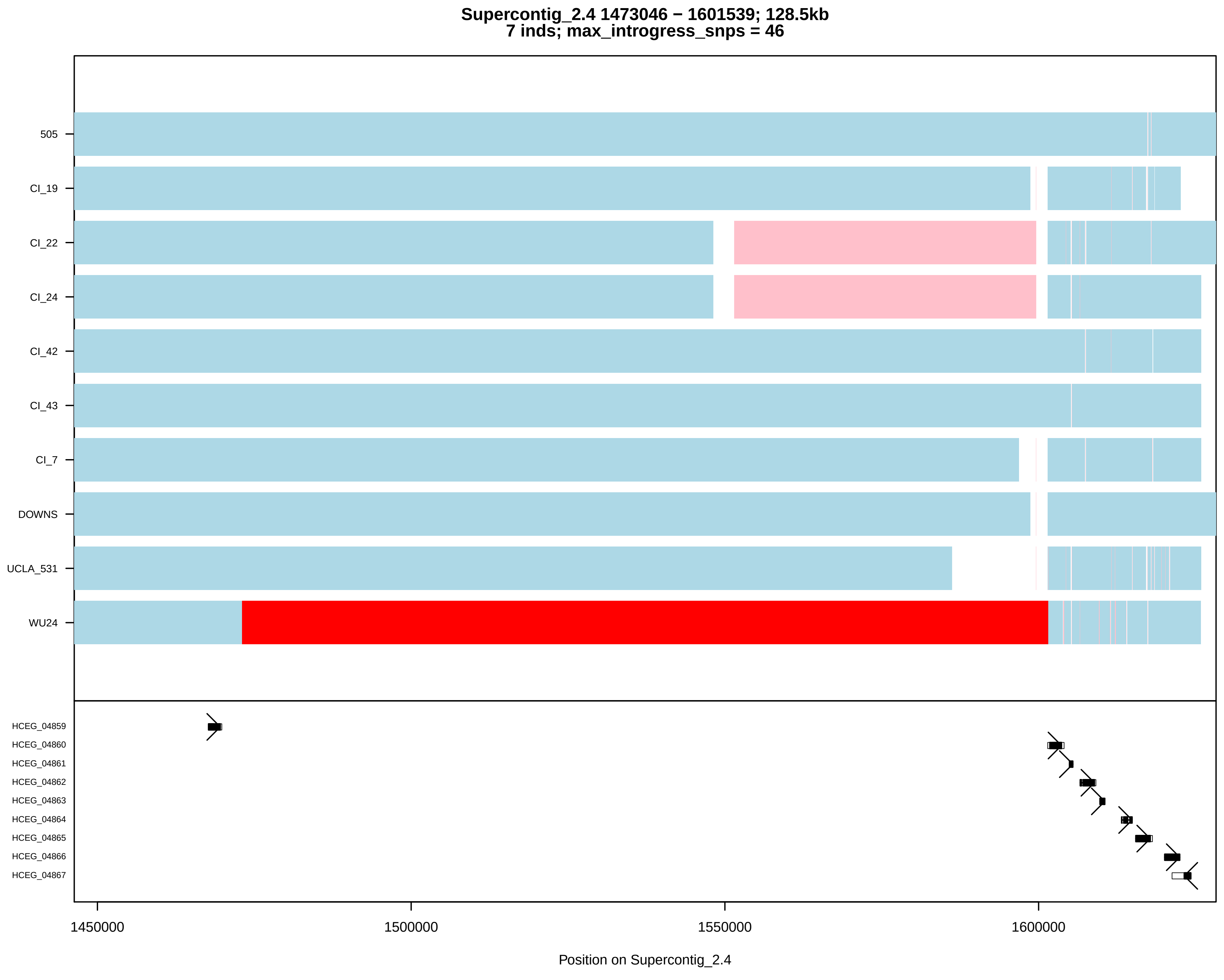

Supercontig_2.4 1473046 − 1601539; 128.5kb
7 inds; max_introgress_snps = 46
505
CI_19
CI_22
CI_24
CI_42
CI_43
CI_7
DOWNS
UCLA_531
WU24
HCEG_04859
HCEG_04860
HCEG_04861
HCEG_04862
HCEG_04863
HCEG_04864
HCEG_04865
HCEG_04866
HCEG_04867
1450000
1500000
1550000
1600000
Position on Supercontig_2.4

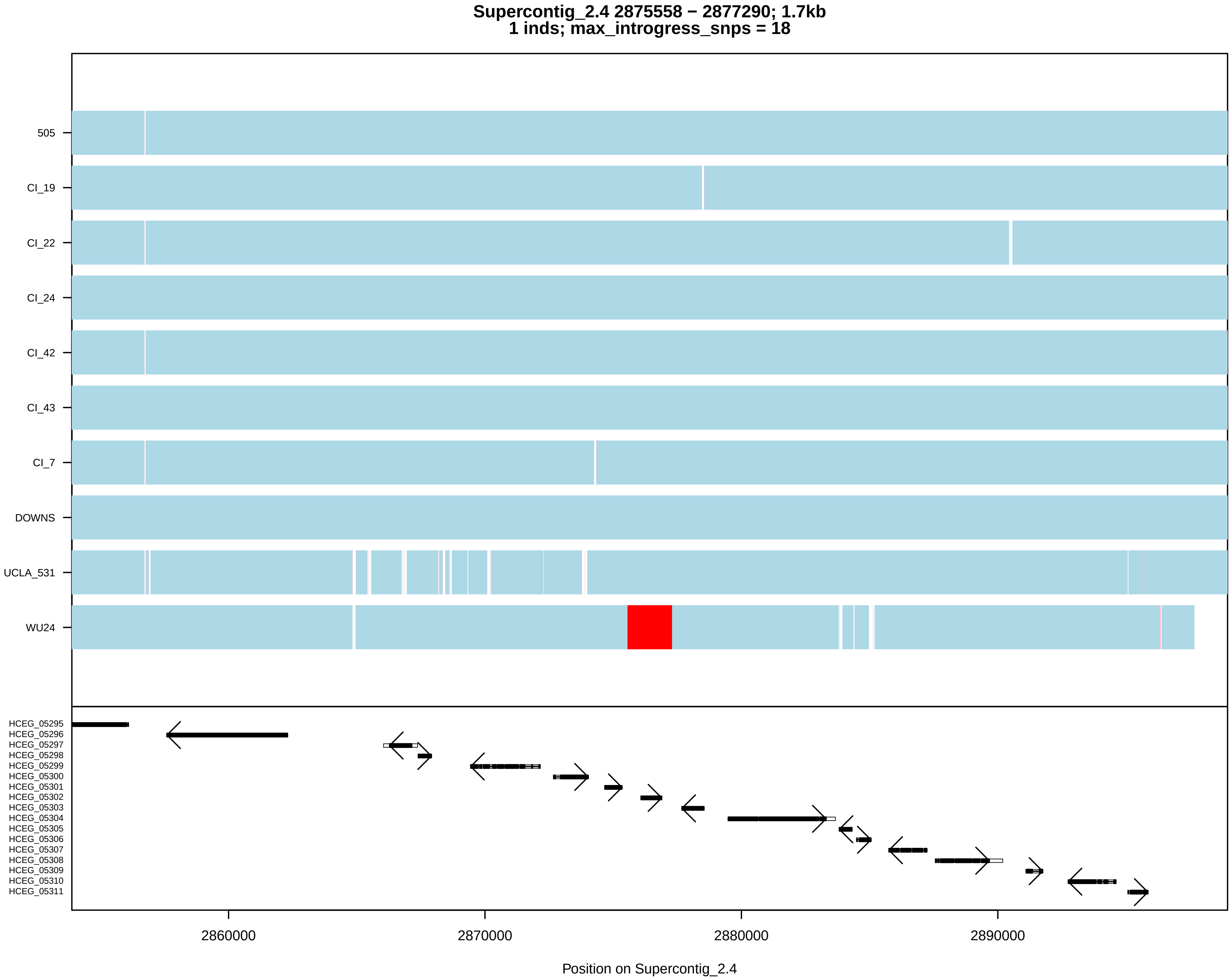

Supercontig_2.4 2875558 − 2877290; 1.7kb
1 inds; max_introgress_snps = 18
505
CI_19
CI_22
CI_24
CI_42
CI_43
CI_7
DOWNS
UCLA_531
WU24
HCEG_05295
HCEG_05296
HCEG_05297
HCEG_05298
HCEG_05299
HCEG_05300
HCEG_05301
HCEG_05302
HCEG_05303
HCEG_05304
HCEG_05305
HCEG_05306
HCEG_05307
HCEG_05308
HCEG_05309
HCEG_05310
HCEG_05311
2860000
2870000
2880000
2890000
Position on Supercontig_2.4

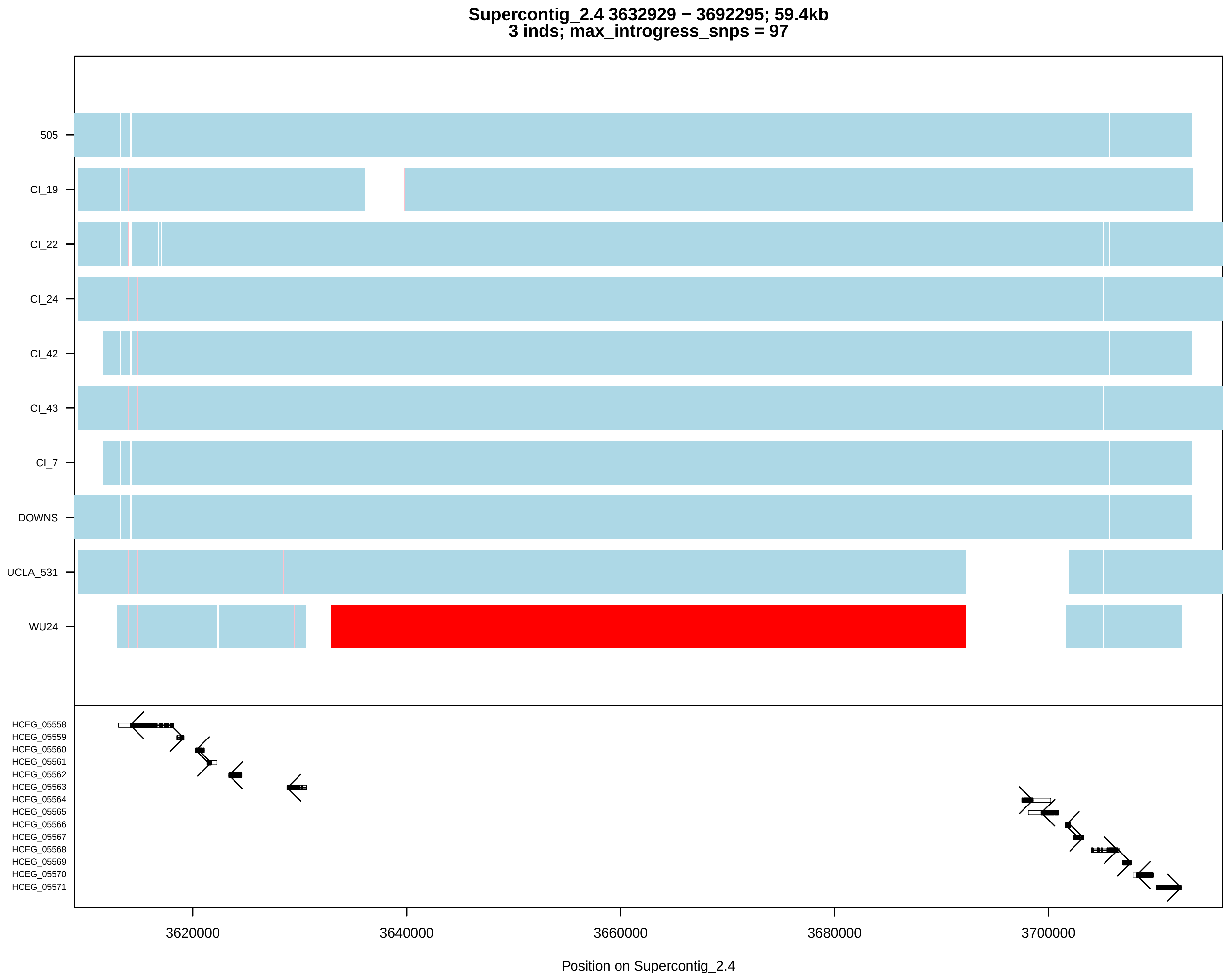

Supercontig_2.4 3632929 − 3692295; 59.4kb
3 inds; max_introgress_snps = 97
505
CI_19
CI_22
CI_24
CI_42
CI_43
CI_7
DOWNS
UCLA_531
WU24
HCEG_05558
HCEG_05559
HCEG_05560
HCEG_05561
HCEG_05562
HCEG_05563
HCEG_05564
HCEG_05565
HCEG_05566
HCEG_05567
HCEG_05568
HCEG_05569
HCEG_05570
HCEG_05571
3620000
3640000
3660000
3680000
3700000
Position on Supercontig_2.4

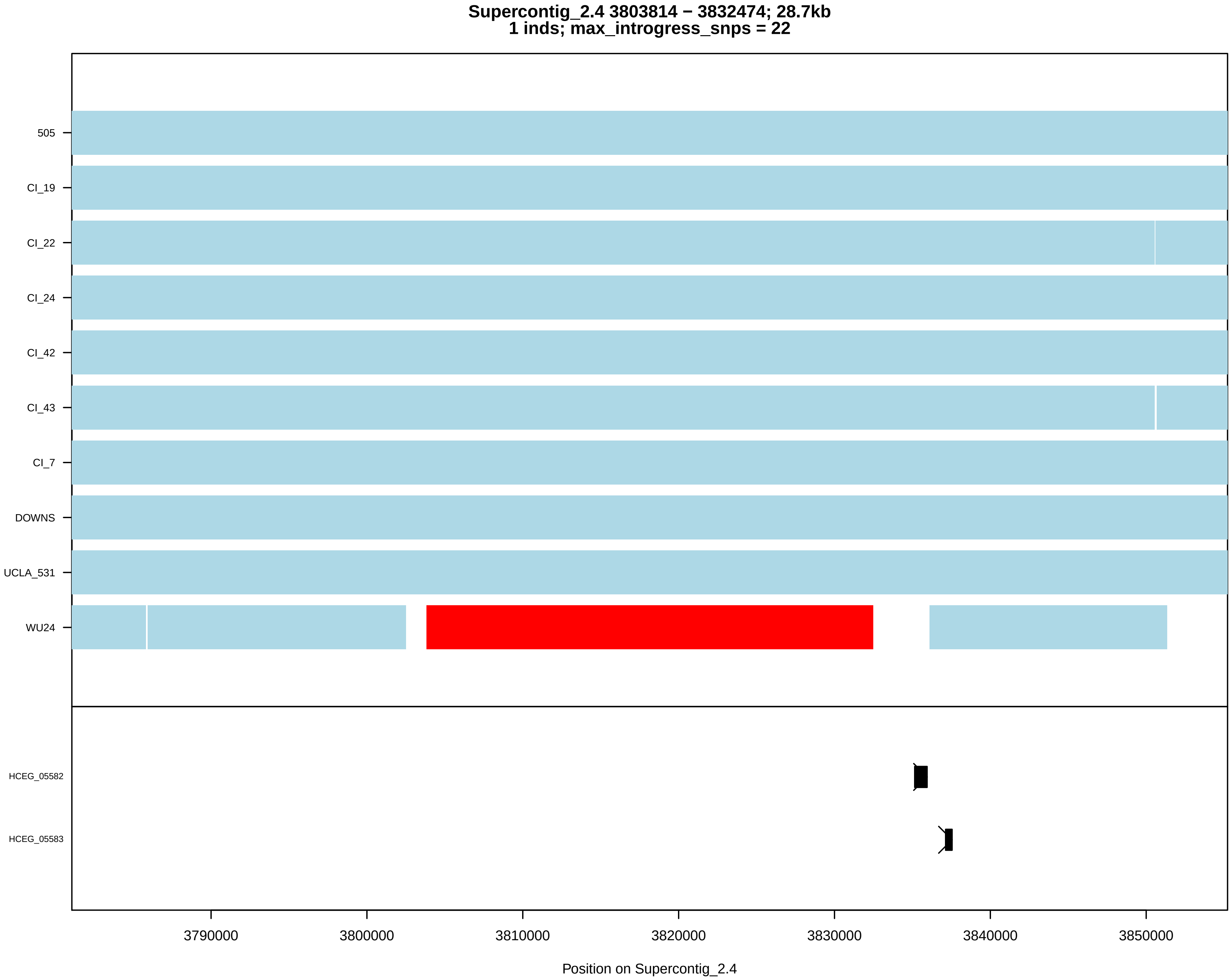
Supercontig_2.4 3803814 − 3832474; 28.7kb
1 inds; max_introgress_snps = 22
505
CI_19
CI_22
CI_24
CI_42
CI_43
CI_7
DOWNS
UCLA_531
WU24
HCEG_05582
HCEG_05583
3790000
3800000
3810000
3820000
3830000
3840000
3850000
Position on Supercontig_2.4

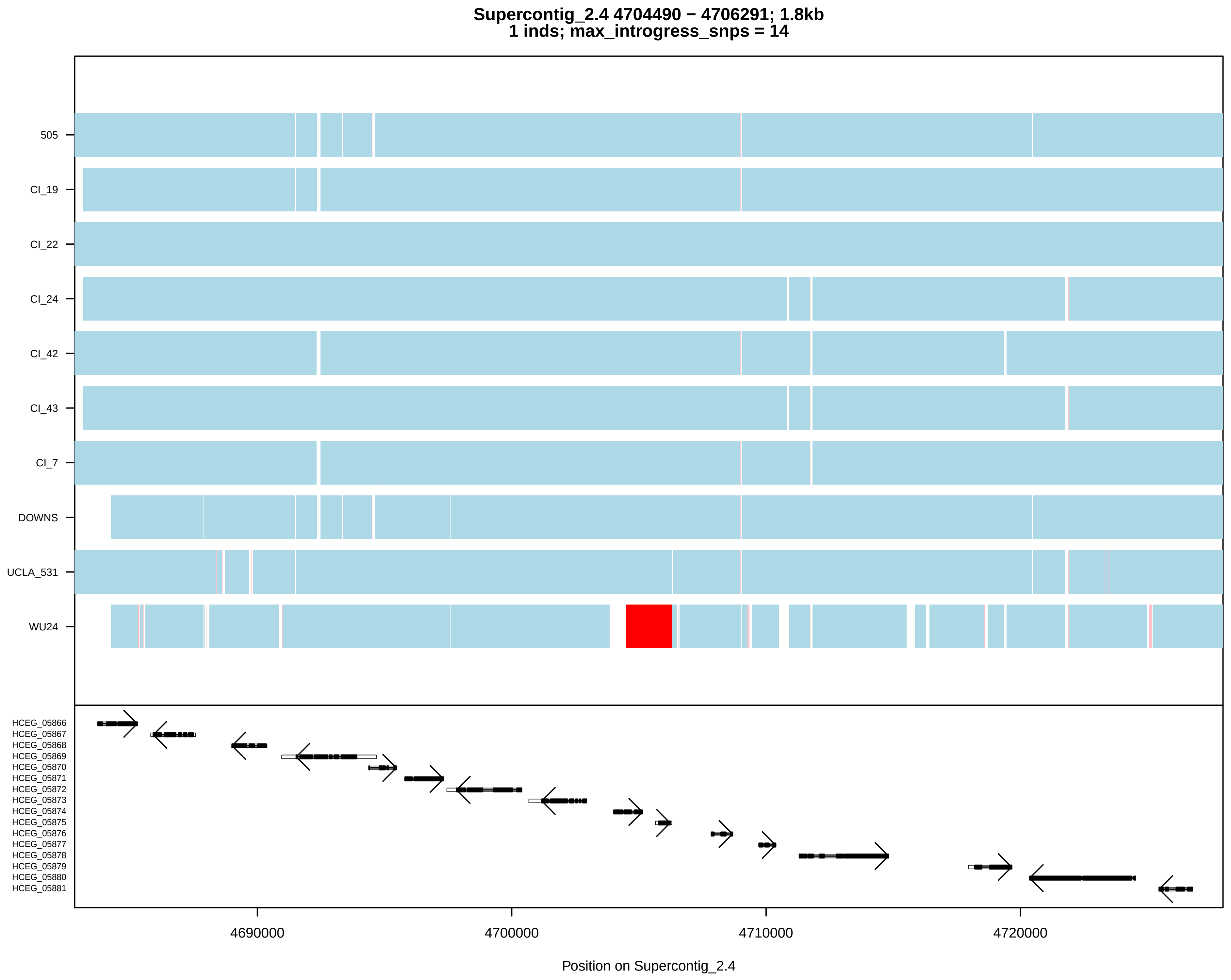

Supercontig_2.4 4704490 − 4706291; 1.8kb
1 inds; max_introgress_snps = 14
505
CI_19
CI_22
CI_24
CI_42
CI_43
CI_7
DOWNS
UCLA_531
WU24
HCEG_05866
HCEG_05867
HCEG_05868
HCEG_05869
HCEG_05870
HCEG_05871
HCEG_05872
HCEG_05873
HCEG_05874
HCEG_05875
HCEG_05876
HCEG_05877
HCEG_05878
HCEG_05879
HCEG_05880
HCEG_05881
4690000
4700000
4710000
4720000
Position on Supercontig_2.4

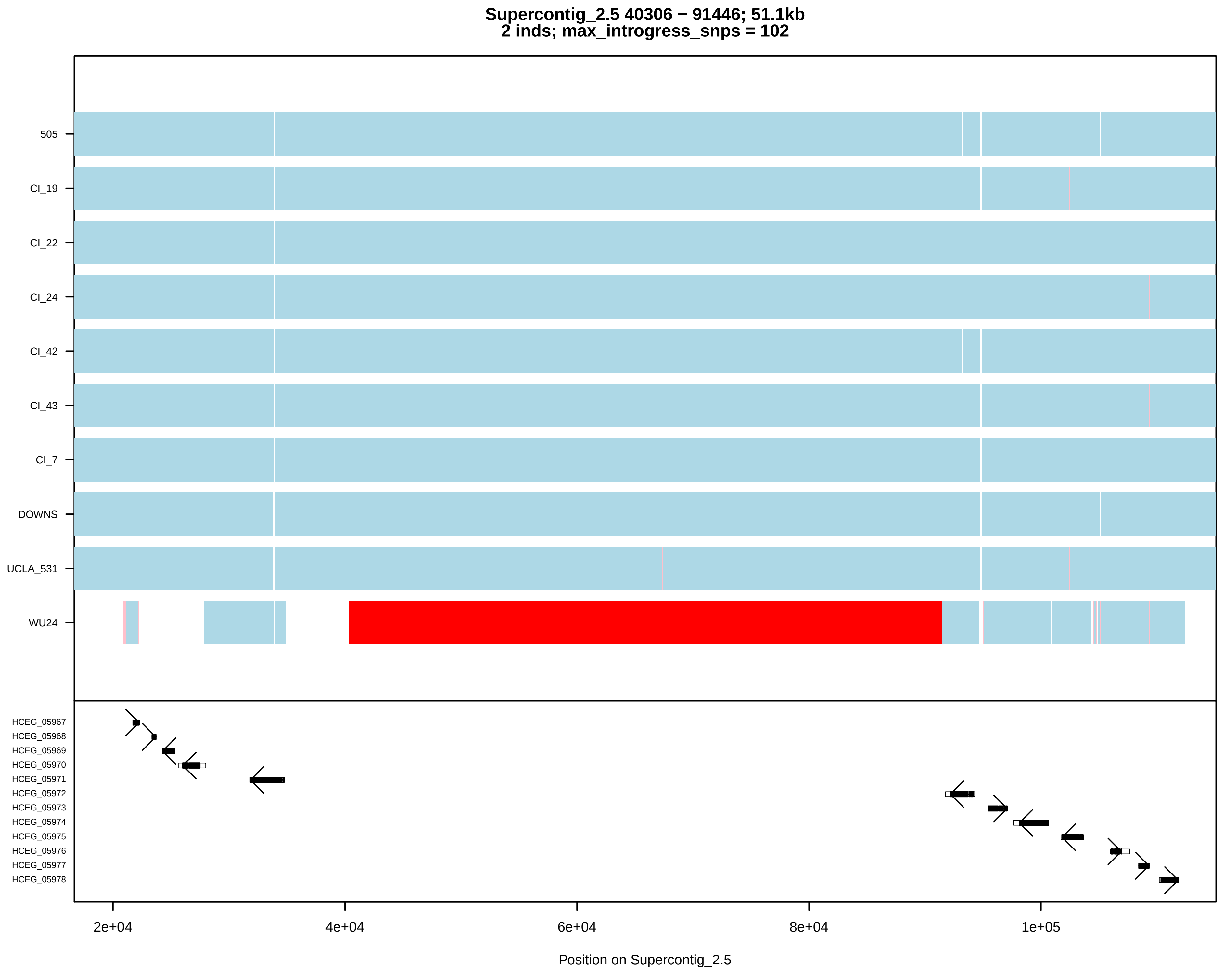

Supercontig_2.5 40306 − 91446; 51.1kb
2 inds; max_introgress_snps = 102
505
CI_19
CI_22
CI_24
CI_42
CI_43
CI_7
DOWNS
UCLA_531
WU24
HCEG_05967
HCEG_05968
HCEG_05969
HCEG_05970
HCEG_05971
HCEG_05972
HCEG_05973
HCEG_05974
HCEG_05975
HCEG_05976
HCEG_05977
HCEG_05978
2e+04
4e+04
6e+04
8e+04
1e+05
Position on Supercontig_2.5

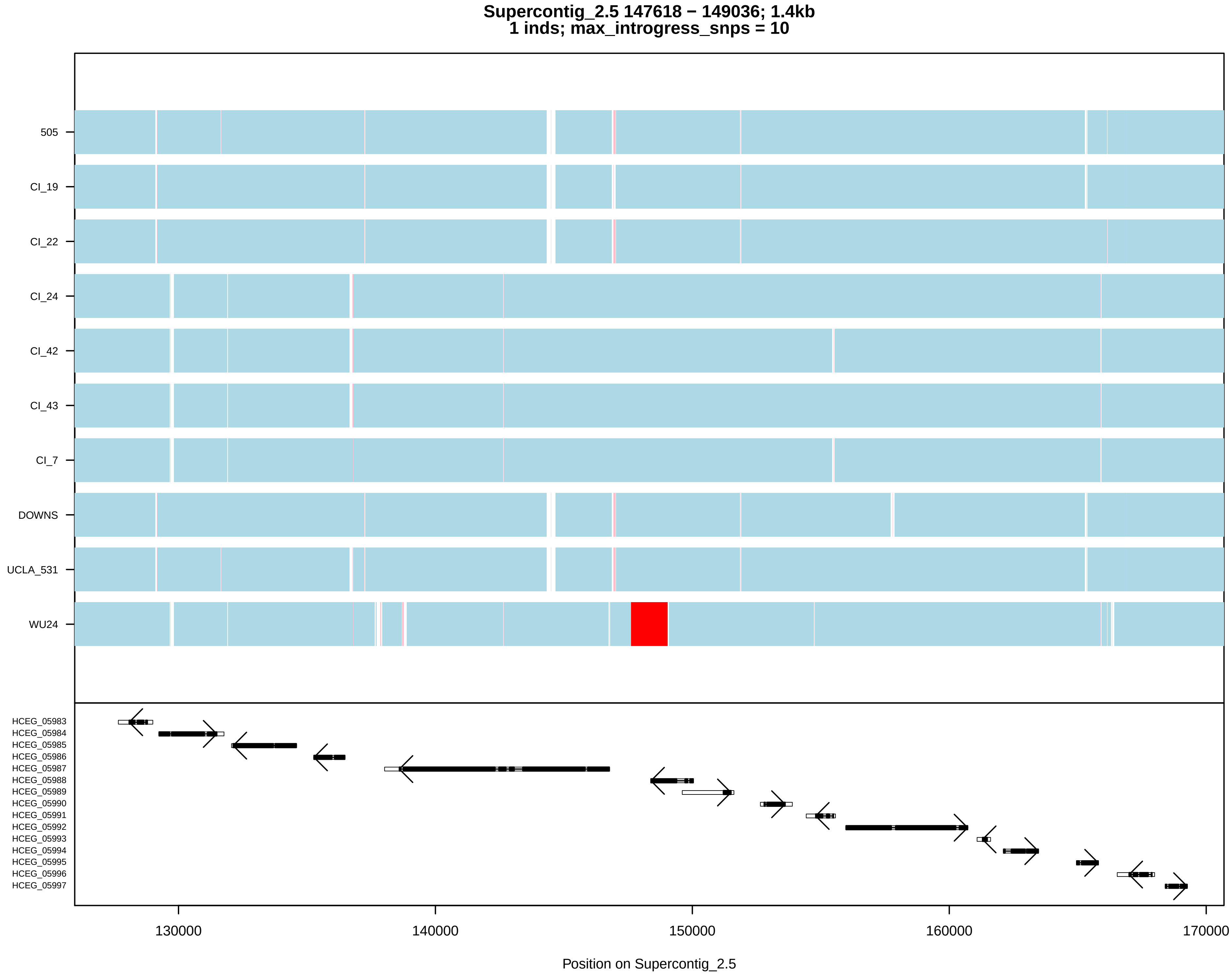

Supercontig_2.5 147618 − 149036; 1.4kb
1 inds; max_introgress_snps = 10
505
CI_19
CI_22
CI_24
CI_42
CI_43
CI_7
DOWNS
UCLA_531
WU24
HCEG_05983
HCEG_05984
HCEG_05985
HCEG_05986
HCEG_05987
HCEG_05988
HCEG_05989
HCEG_05990
HCEG_05991
HCEG_05992
HCEG_05993
HCEG_05994
HCEG_05995
HCEG_05996
HCEG_05997
130000
140000
150000
160000
170000
Position on Supercontig_2.5

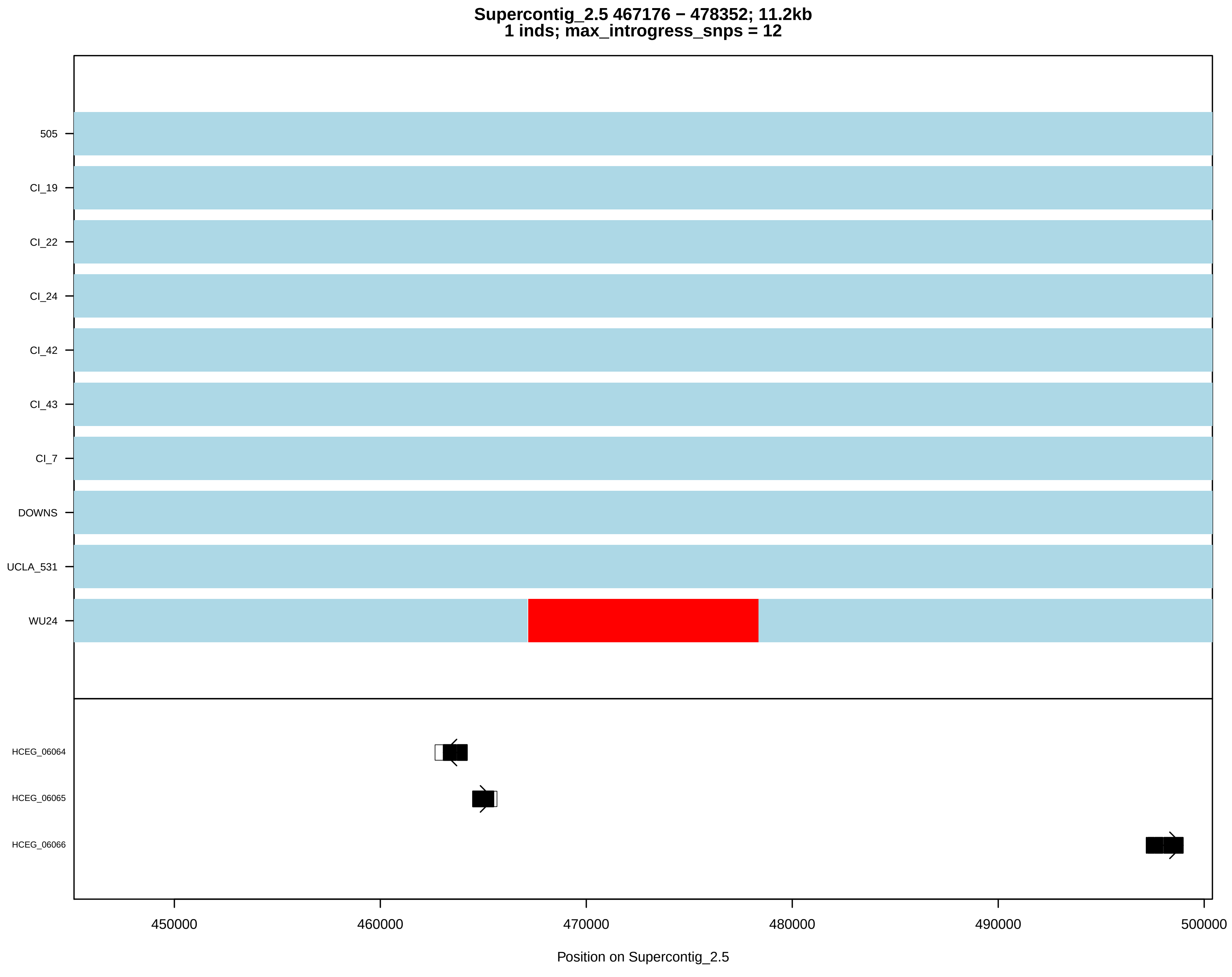

Supercontig_2.5 467176 − 478352; 11.2kb
1 inds; max_introgress_snps = 12
505
CI_19
CI_22
CI_24
CI_42
CI_43
CI_7
DOWNS
UCLA_531
WU24
HCEG_06064
HCEG_06065
HCEG_06066
450000
460000
470000
480000
490000
500000
Position on Supercontig_2.5

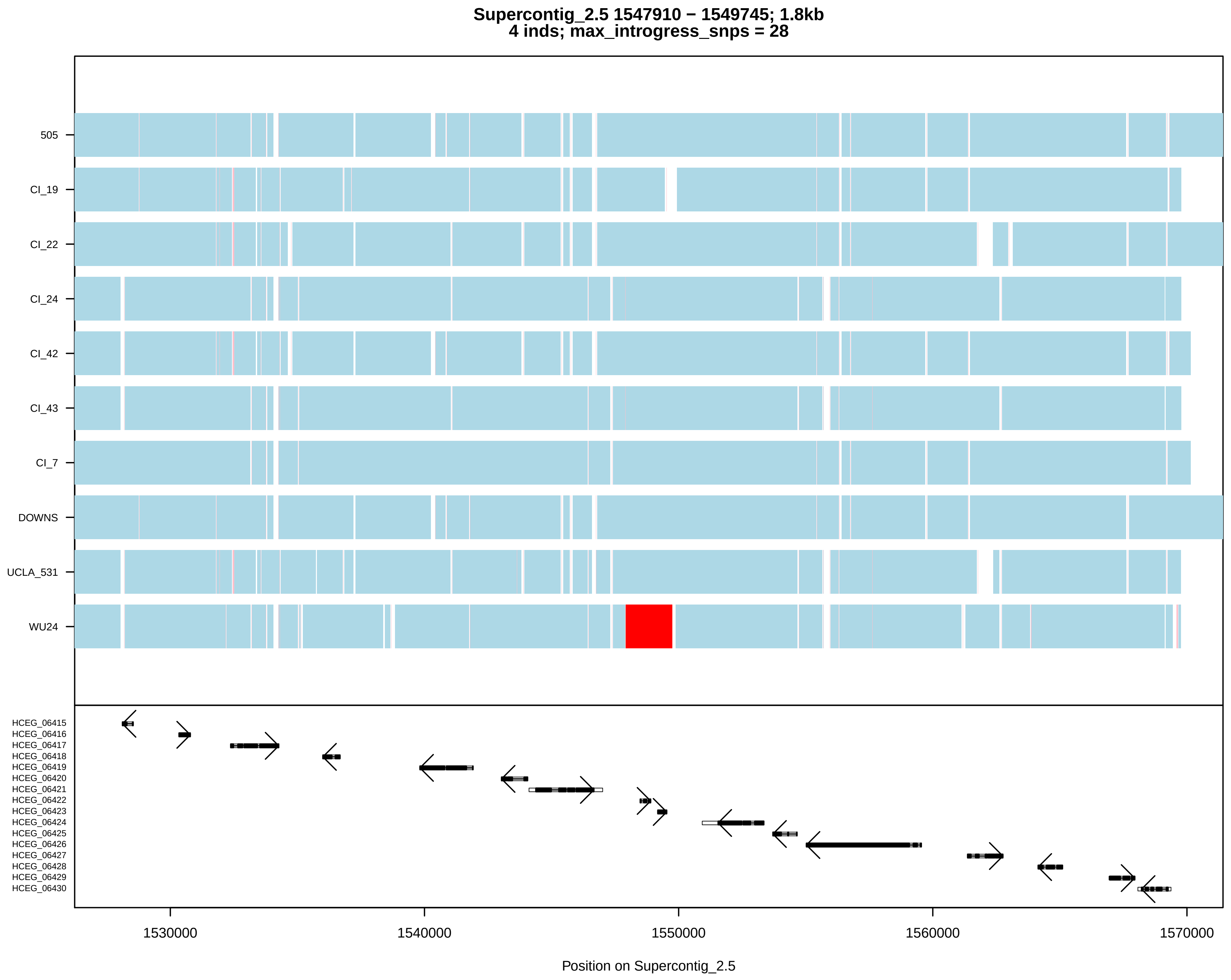

Supercontig_2.5 1547910 − 1549745; 1.8kb
4 inds; max_introgress_snps = 28
505
CI_19
CI_22
CI_24
CI_42
CI_43
CI_7
DOWNS
UCLA_531
WU24
HCEG_06415
HCEG_06416
HCEG_06417
HCEG_06418
HCEG_06419
HCEG_06420
HCEG_06421
HCEG_06422
HCEG_06423
HCEG_06424
HCEG_06425
HCEG_06426
HCEG_06427
HCEG_06428
HCEG_06429
HCEG_06430
1530000
1540000
1550000
1560000
1570000
Position on Supercontig_2.5

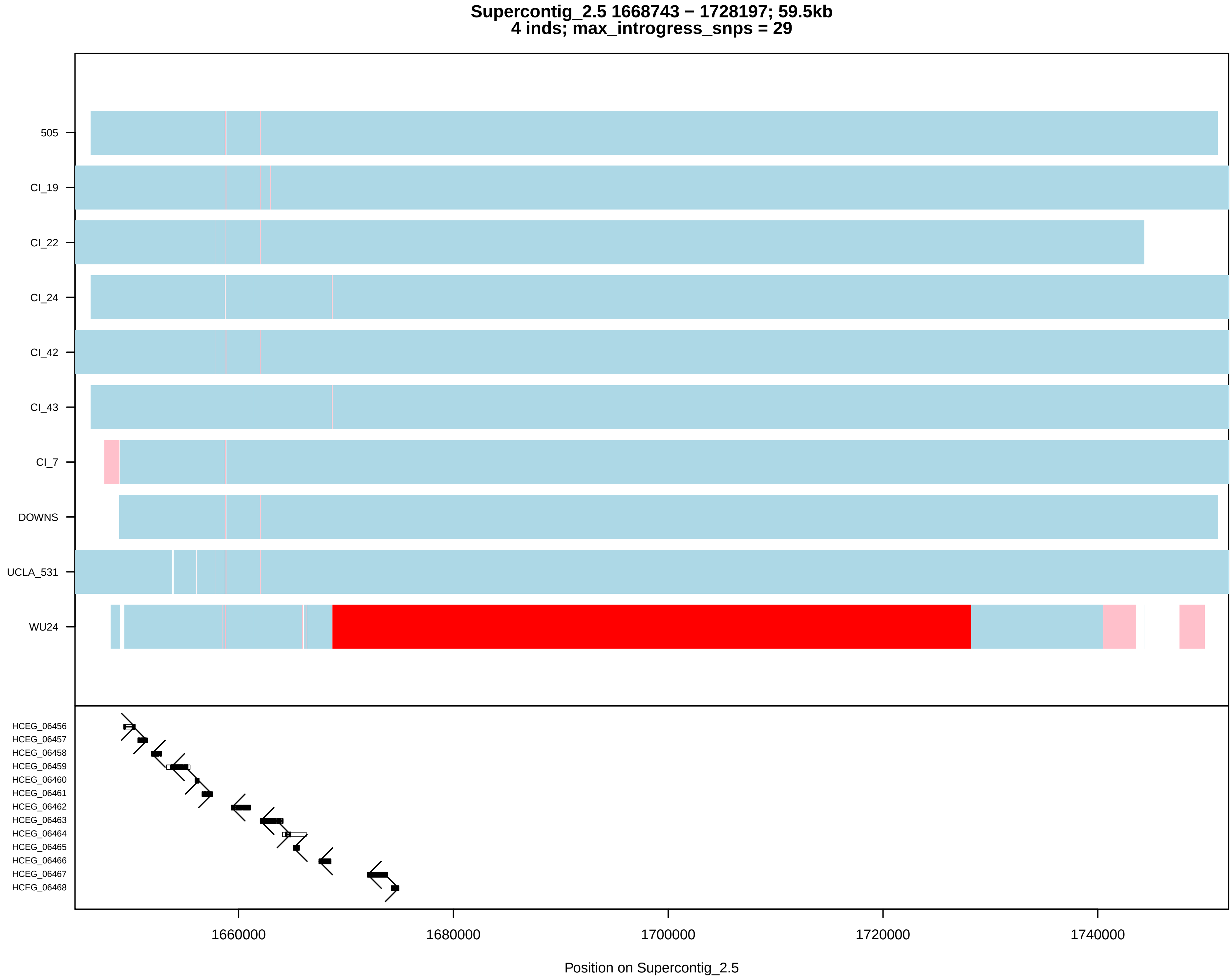

Supercontig_2.5 1668743 − 1728197; 59.5kb
4 inds; max_introgress_snps = 29
505
CI_19
CI_22
CI_24
CI_42
CI_43
CI_7
DOWNS
UCLA_531
WU24
HCEG_06456
HCEG_06457
HCEG_06458
HCEG_06459
HCEG_06460
HCEG_06461
HCEG_06462
HCEG_06463
HCEG_06464
HCEG_06465
HCEG_06466
HCEG_06467
HCEG_06468
1660000
1680000
1700000
1720000
1740000
Position on Supercontig_2.5

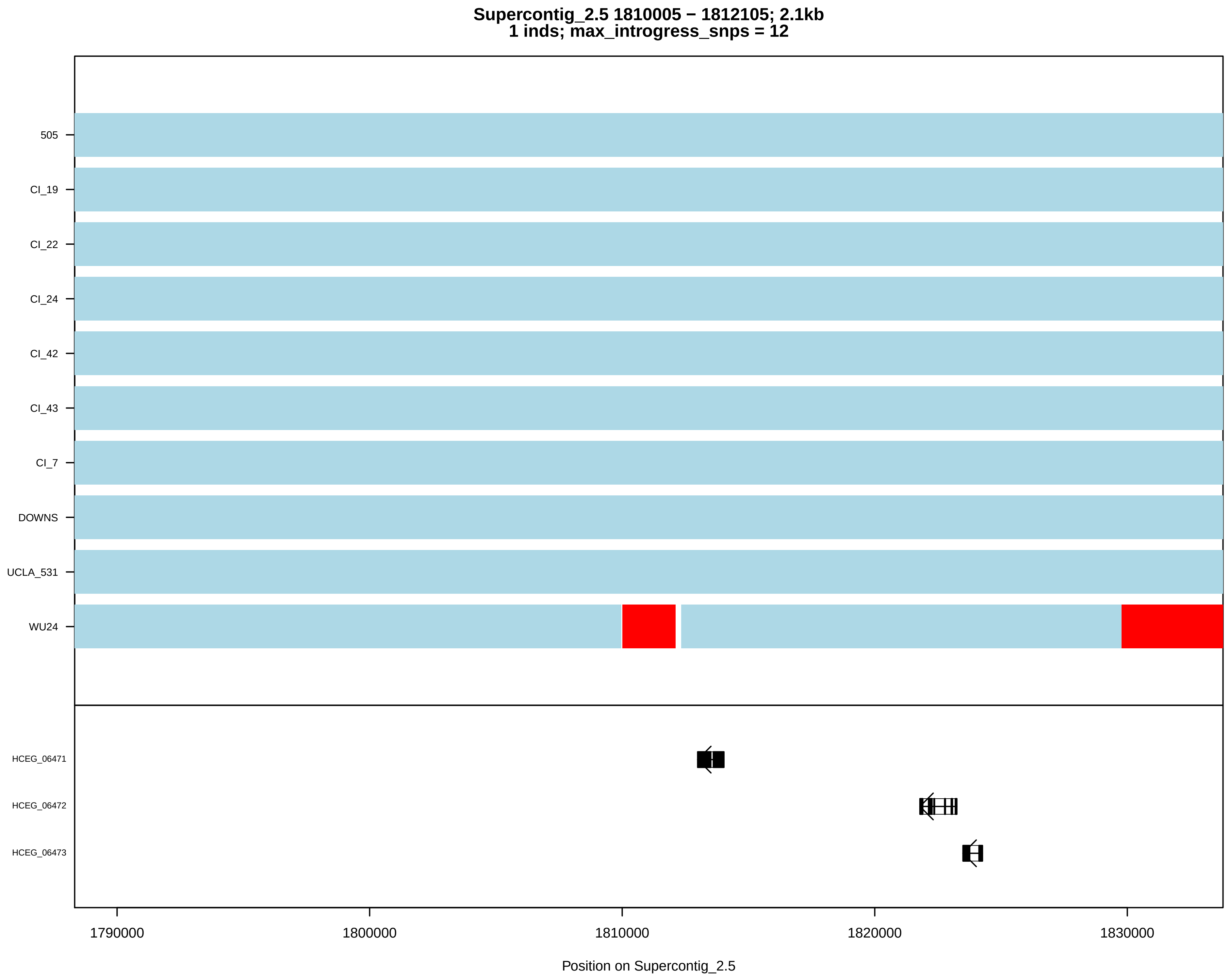
Supercontig_2.5 1810005 − 1812105; 2.1kb
1 inds; max_introgress_snps = 12
505
CI_19
CI_22
CI_24
CI_42
CI_43
CI_7
DOWNS
UCLA_531
WU24
HCEG_06471
HCEG_06472
HCEG_06473
1790000
1800000
1810000
1820000
1830000
Position on Supercontig_2.5

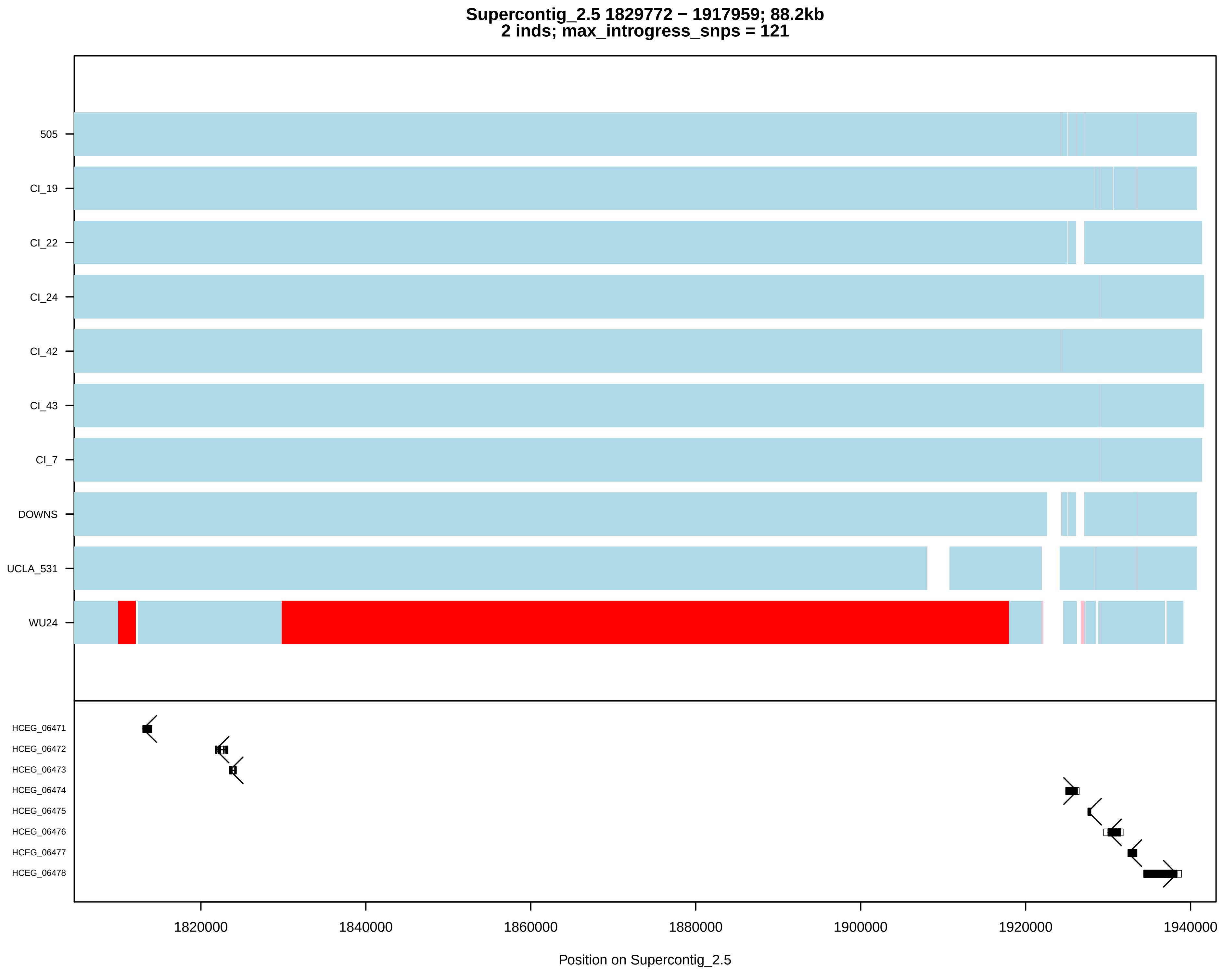
Supercontig_2.5 1829772 − 1917959; 88.2kb
2 inds; max_introgress_snps = 121
505
CI_19
CI_22
CI_24
CI_42
CI_43
CI_7
DOWNS
UCLA_531
WU24
HCEG_06471
HCEG_06472
HCEG_06473
HCEG_06474
HCEG_06475
HCEG_06476
HCEG_06477
HCEG_06478
1820000
1840000
1860000
1880000
1900000
1920000
1940000
Position on Supercontig_2.5

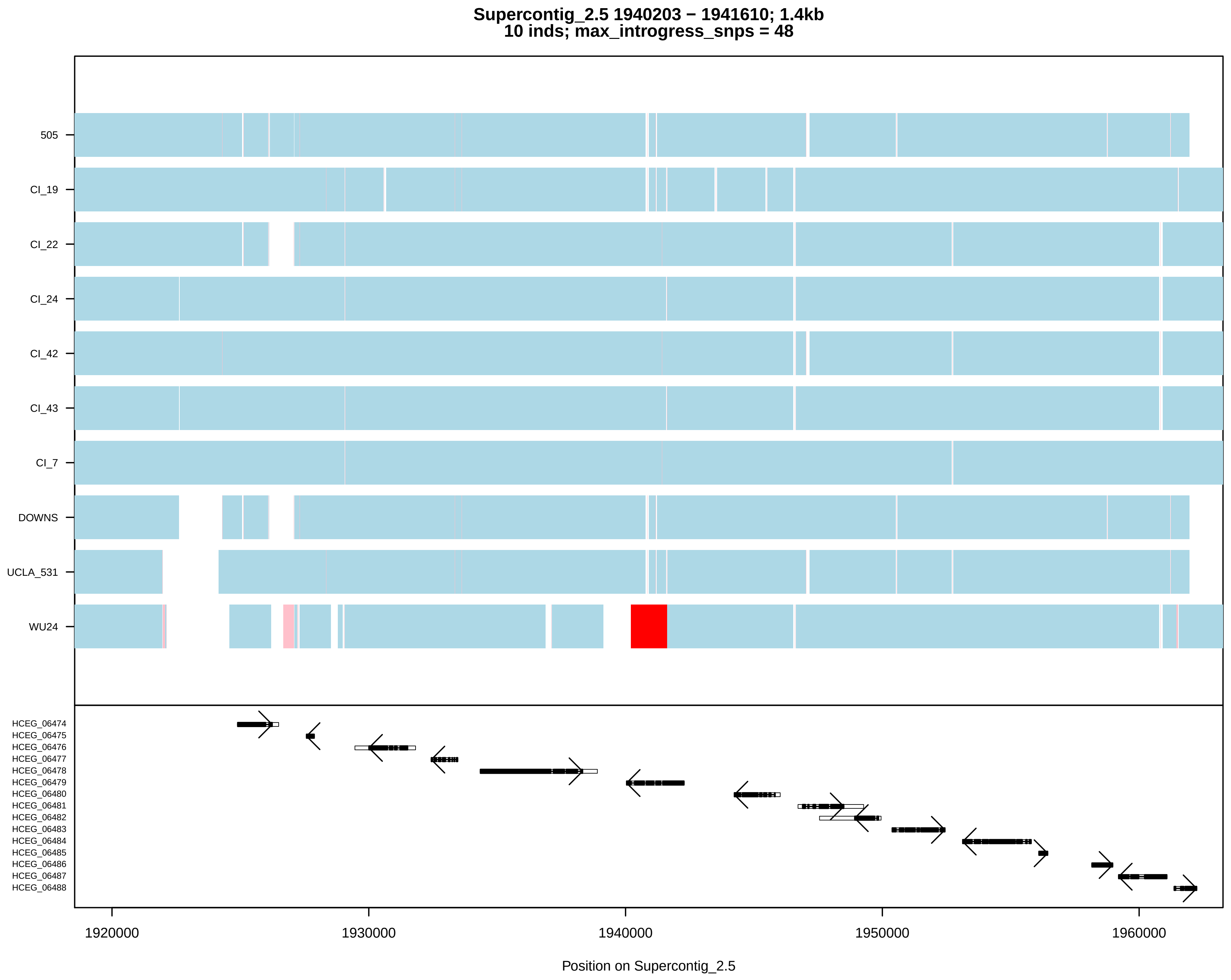
Supercontig_2.5 1940203 − 1941610; 1.4kb
10 inds; max_introgress_snps = 48
505
CI_19
CI_22
CI_24
CI_42
CI_43
CI_7
DOWNS
UCLA_531
WU24
HCEG_06474
HCEG_06475
HCEG_06476
HCEG_06477
HCEG_06478
HCEG_06479
HCEG_06480
HCEG_06481
HCEG_06482
HCEG_06483
HCEG_06484
HCEG_06485
HCEG_06486
HCEG_06487
HCEG_06488
1920000
1930000
1940000
1950000
1960000
Position on Supercontig_2.5

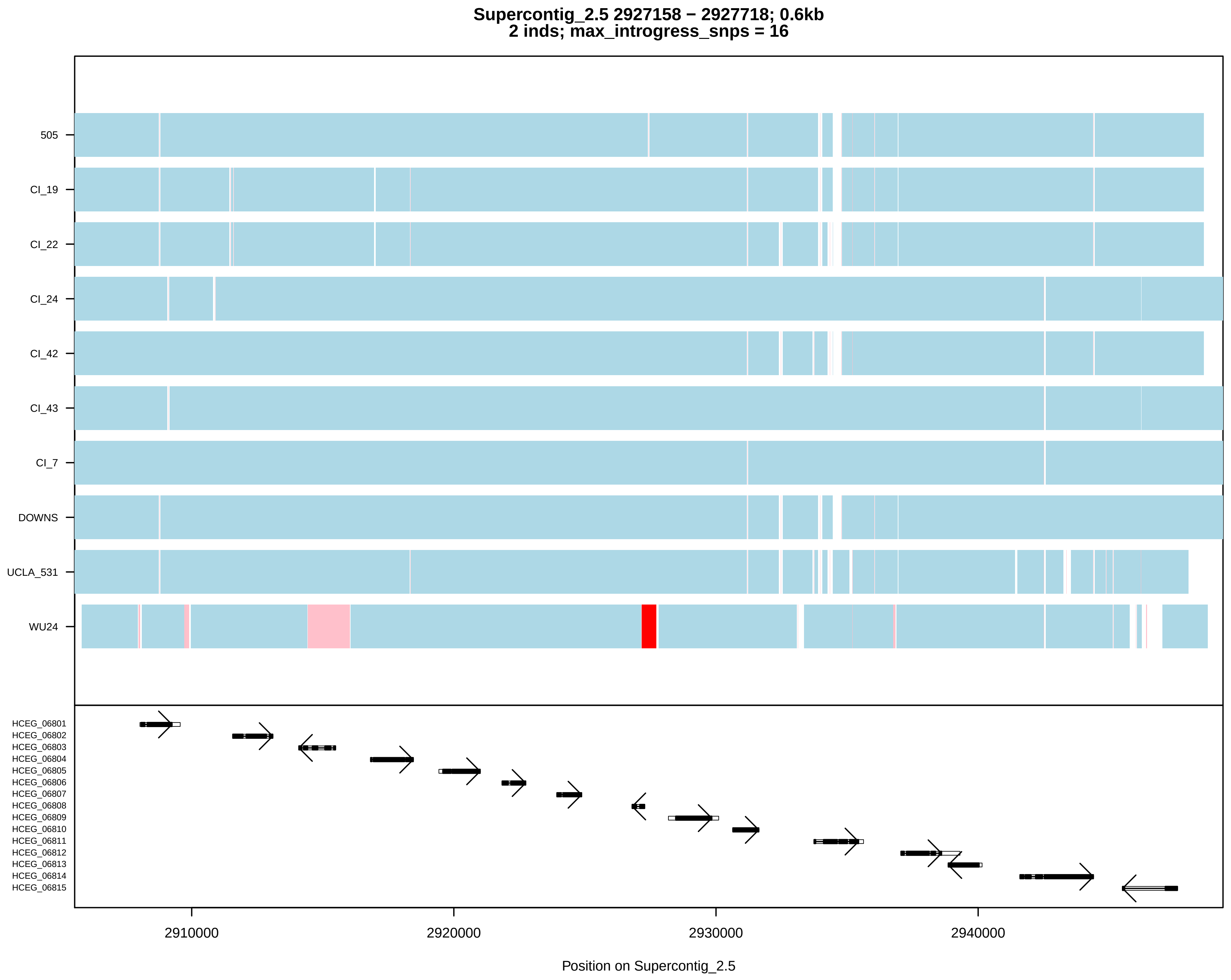

Supercontig_2.5 2927158 − 2927718; 0.6kb
2 inds; max_introgress_snps = 16
505
CI_19
CI_22
CI_24
CI_42
CI_43
CI_7
DOWNS
UCLA_531
WU24
HCEG_06801
HCEG_06802
HCEG_06803
HCEG_06804
HCEG_06805
HCEG_06806
HCEG_06807
HCEG_06808
HCEG_06809
HCEG_06810
HCEG_06811
HCEG_06812
HCEG_06813
HCEG_06814
HCEG_06815
2910000
2920000
2930000
2940000
Position on Supercontig_2.5

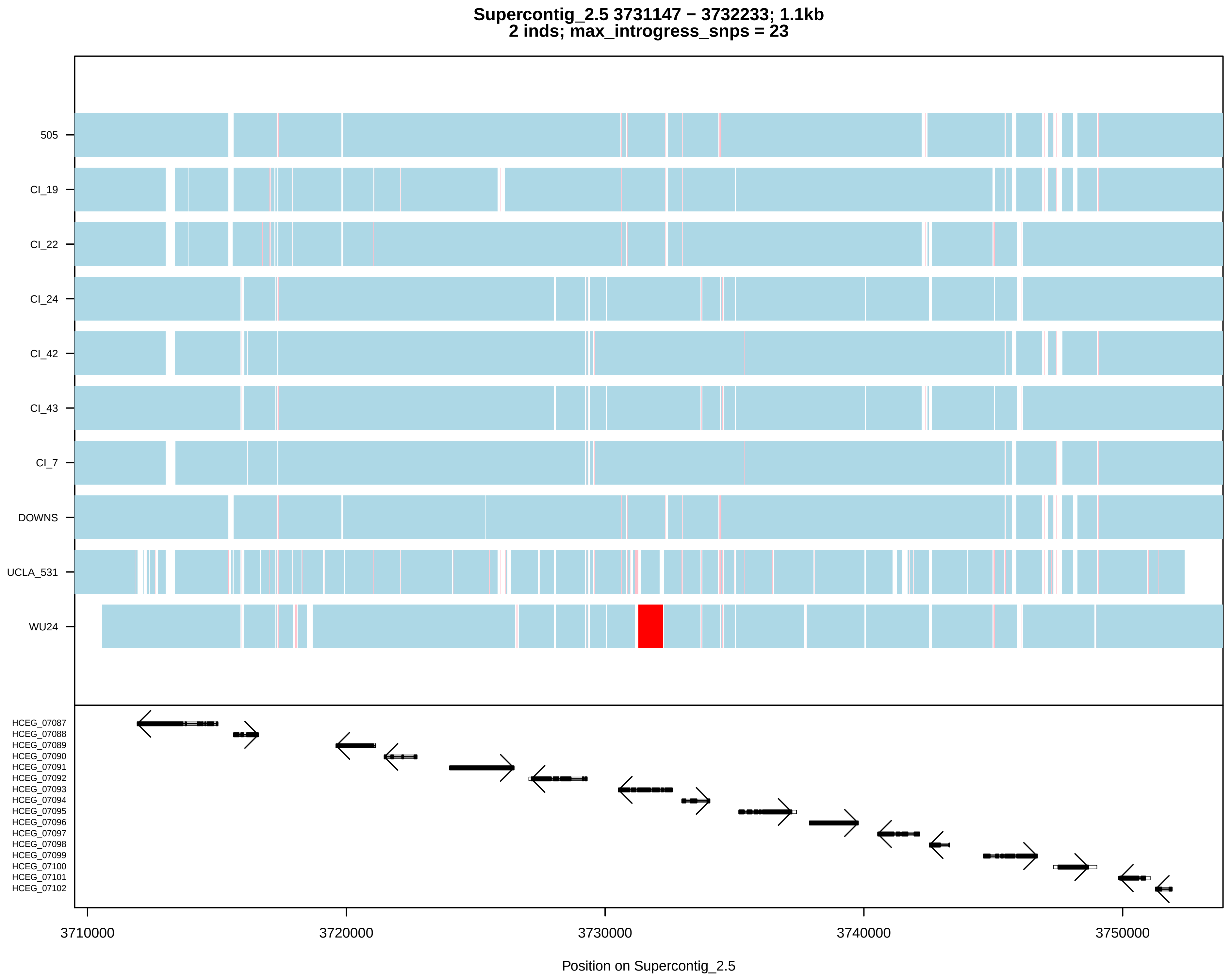
Supercontig_2.5 3731147 − 3732233; 1.1kb
2 inds; max_introgress_snps = 23
505
CI_19
CI_22
CI_24
CI_42
CI_43
CI_7
DOWNS
UCLA_531
WU24
HCEG_07087
HCEG_07088
HCEG_07089
HCEG_07090
HCEG_07091
HCEG_07092
HCEG_07093
HCEG_07094
HCEG_07095
HCEG_07096
HCEG_07097
HCEG_07098
HCEG_07099
HCEG_07100
HCEG_07101
HCEG_07102
3710000
3720000
3730000
3740000
3750000
Position on Supercontig_2.5

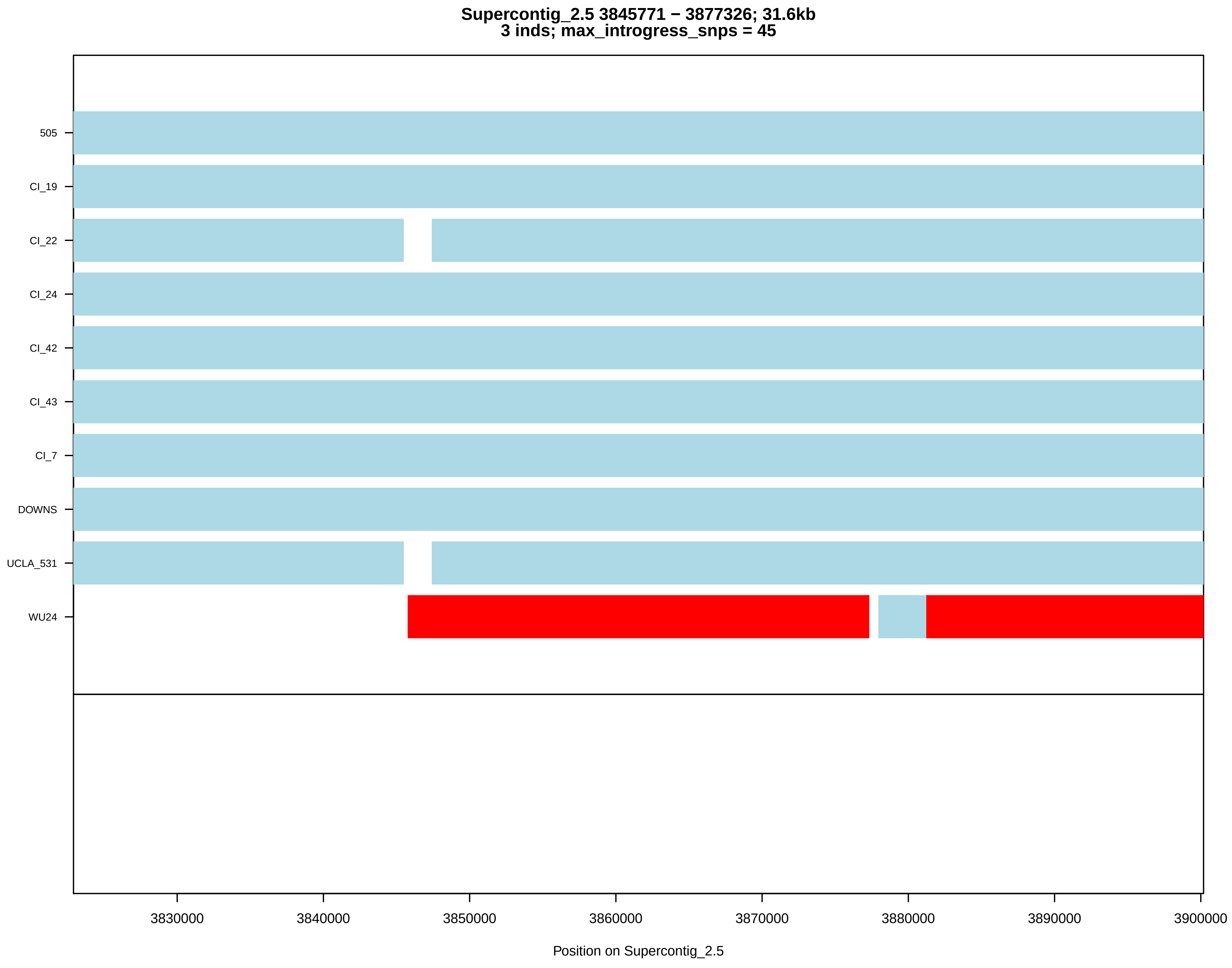
Supercontig_2.5 3845771 − 3877326; 31.6kb
3 inds; max_introgress_snps = 45
505
CI_19
CI_22
CI_24
CI_42
CI_43
CI_7
DOWNS
UCLA_531
WU24
3830000
3840000
3850000
3860000
3870000
3880000
3890000
3900000
Position on Supercontig_2.5

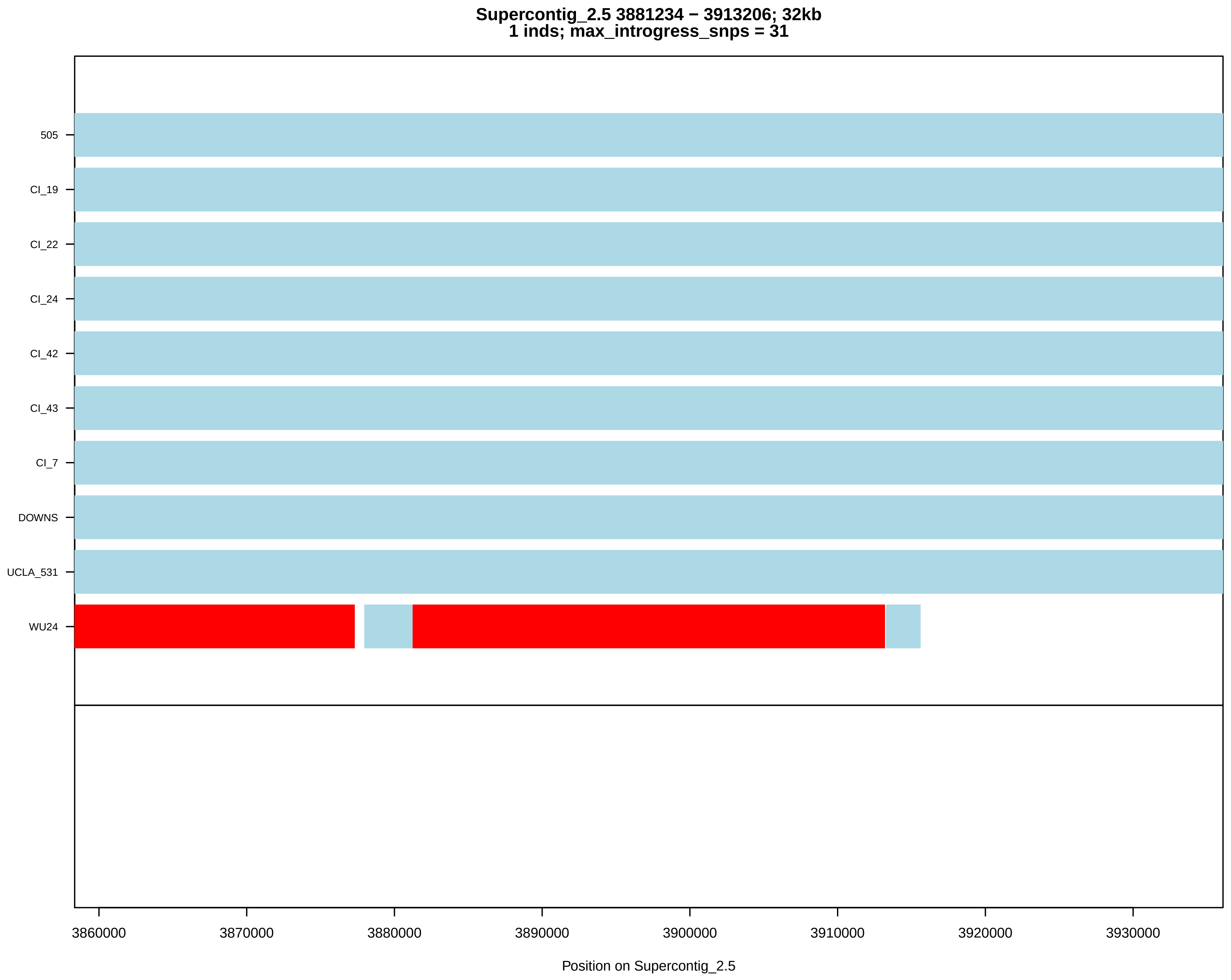

Supercontig_2.5 3881234 − 3913206; 32kb
1 inds; max_introgress_snps = 31
505
CI_19
CI_22
CI_24
CI_42
CI_43
CI_7
DOWNS
UCLA_531
WU24
3860000
3870000
3880000
3890000
3900000
3910000
3920000
3930000
Position on Supercontig_2.5

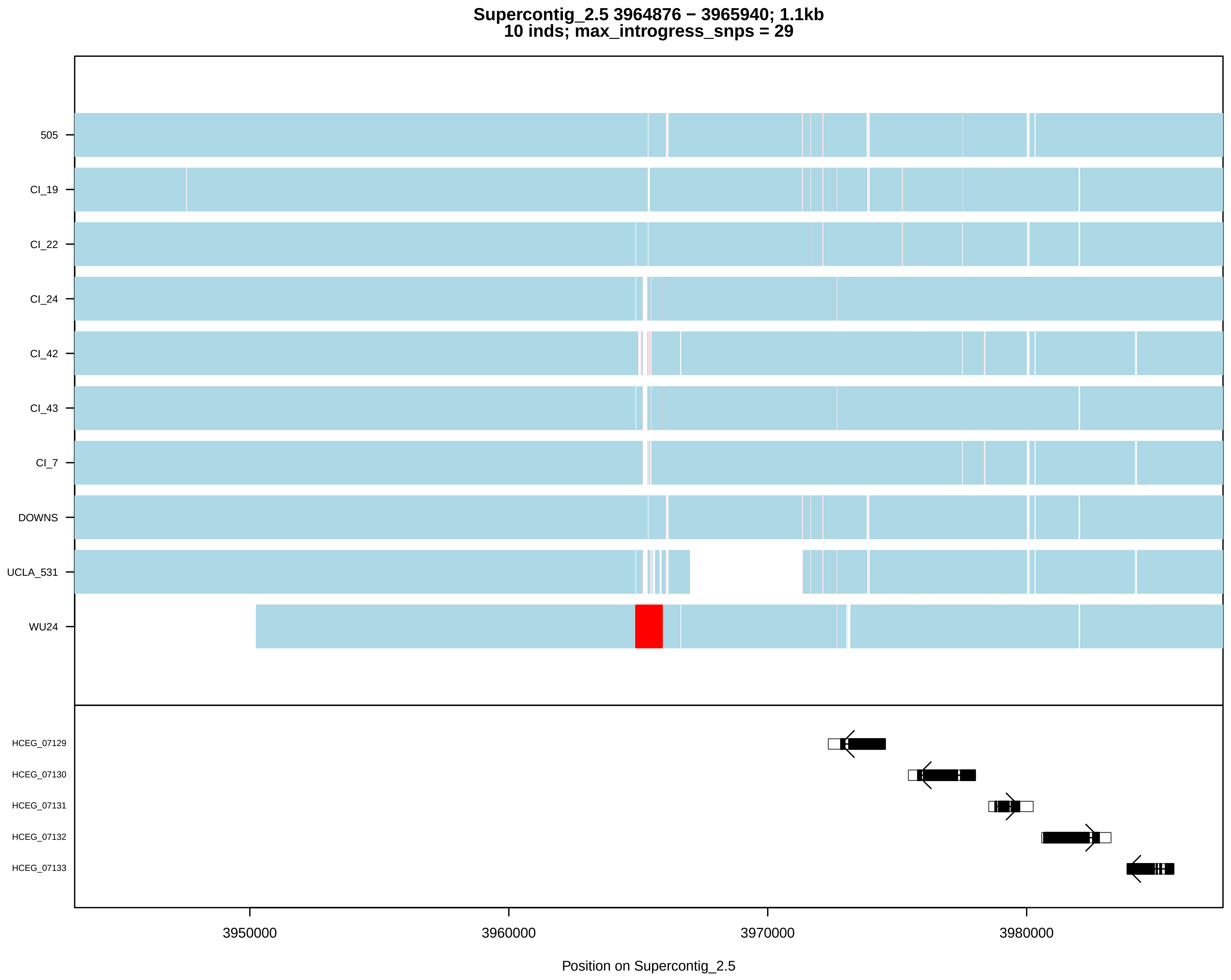

Supercontig_2.5 3964876 − 3965940; 1.1kb
10 inds; max_introgress_snps = 29
505
CI_19
CI_22
CI_24
CI_42
CI_43
CI_7
DOWNS
UCLA_531
WU24
HCEG_07129
HCEG_07130
HCEG_07131
HCEG_07132
HCEG_07133
3950000
3960000
3970000
3980000
Position on Supercontig_2.5

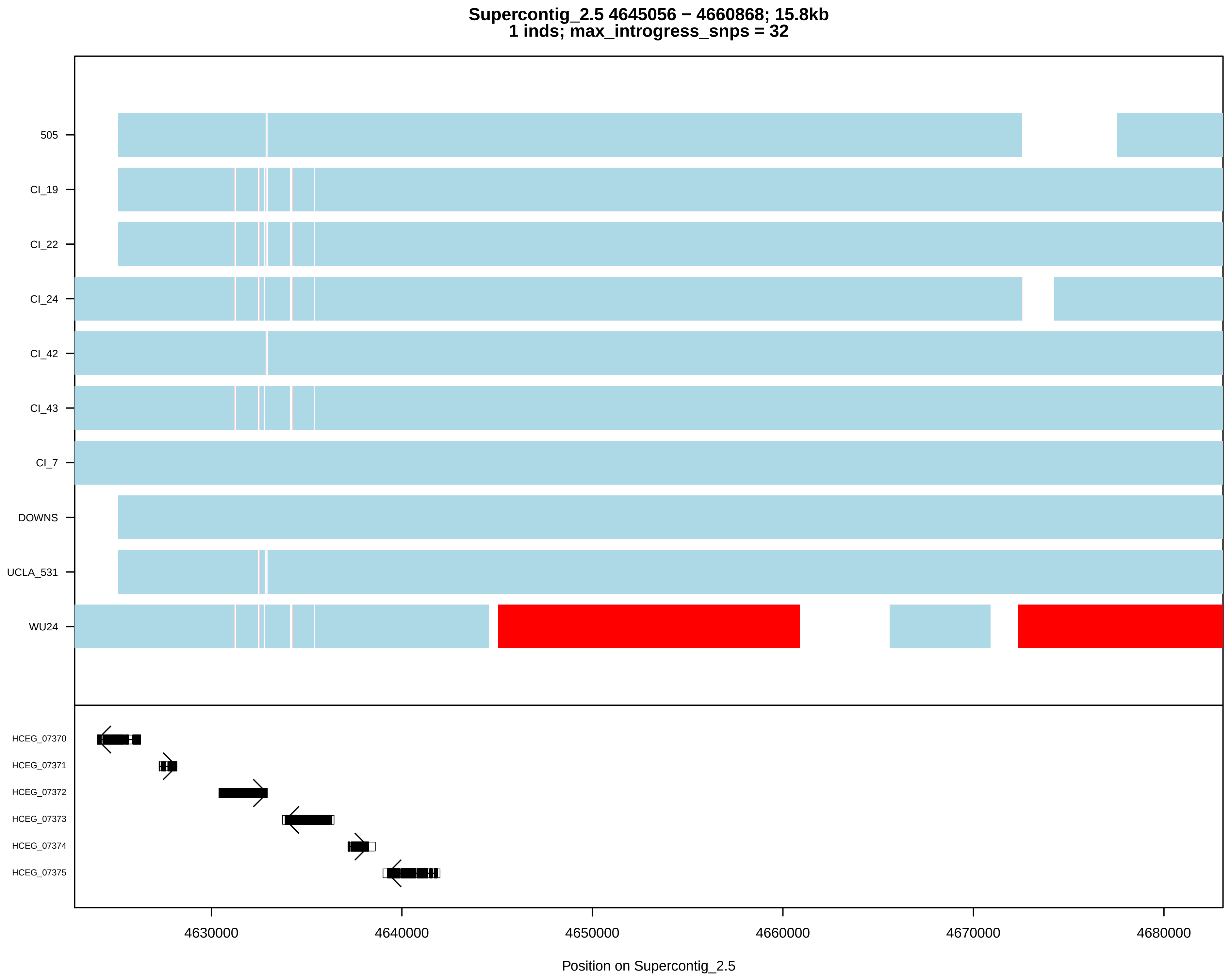

Supercontig_2.5 4645056 − 4660868; 15.8kb
1 inds; max_introgress_snps = 32
505
CI_19
CI_22
CI_24
CI_42
CI_43
CI_7
DOWNS
UCLA_531
WU24
HCEG_07370
HCEG_07371
HCEG_07372
HCEG_07373
HCEG_07374
HCEG_07375
4630000
4640000
4650000
4660000
4670000
4680000
Position on Supercontig_2.5

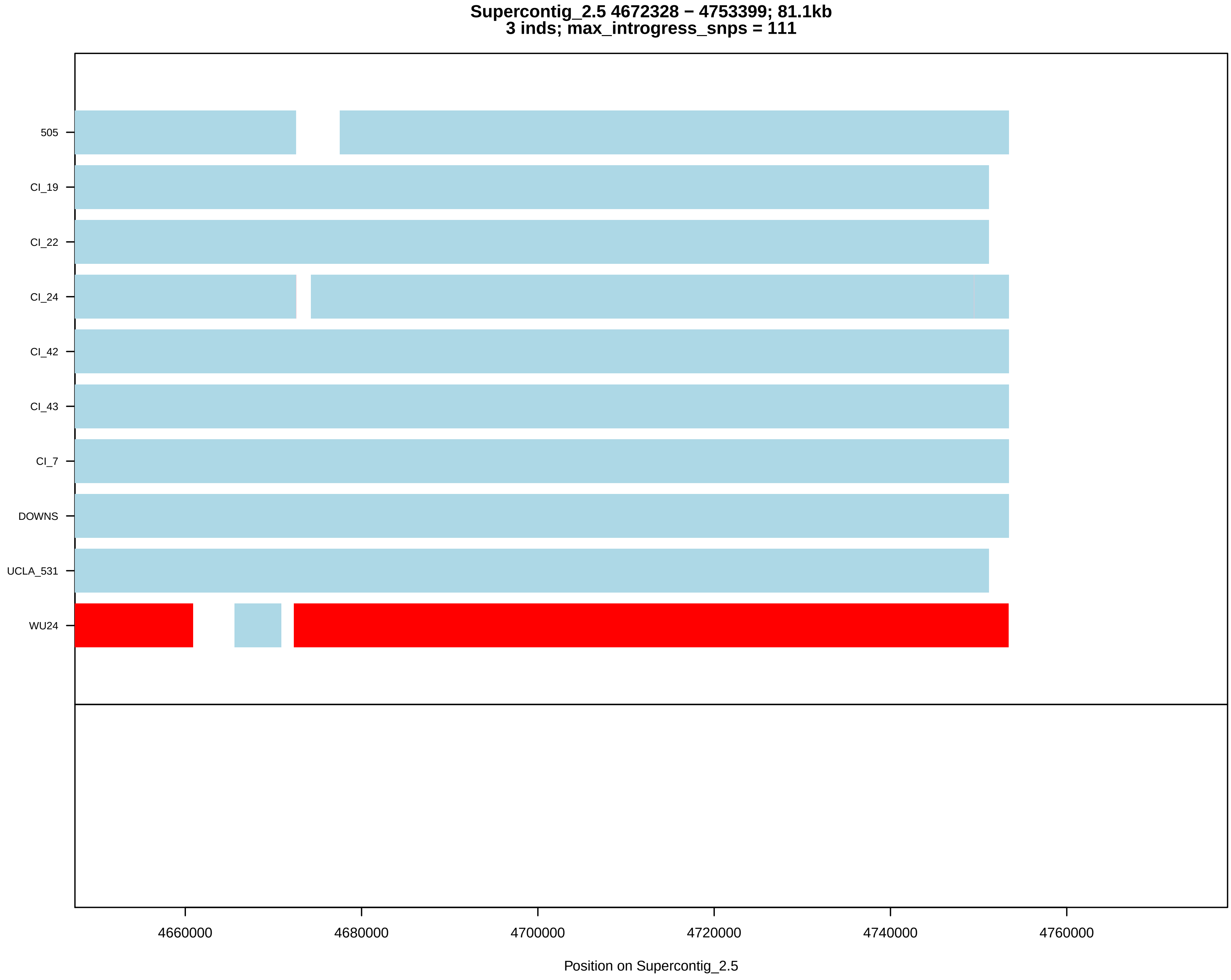
Supercontig_2.5 4672328 − 4753399; 81.1kb
3 inds; max_introgress_snps = 111
505
CI_19
CI_22
CI_24
CI_42
CI_43
CI_7
DOWNS
UCLA_531
WU24
4660000
4680000
4700000
4720000
4740000
4760000
Position on Supercontig_2.5

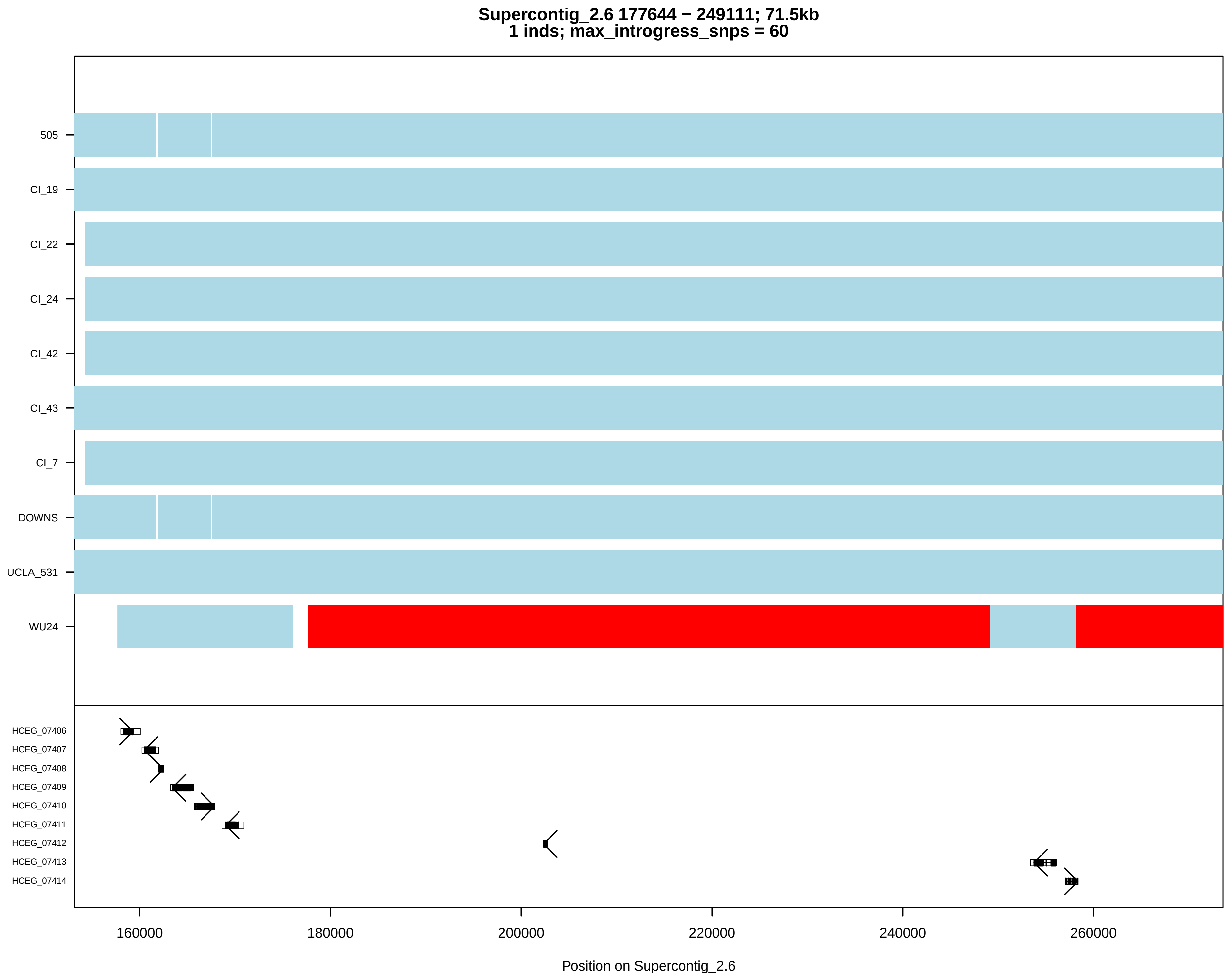

Supercontig_2.6 177644 − 249111; 71.5kb
1 inds; max_introgress_snps = 60
505
CI_19
CI_22
CI_24
CI_42
CI_43
CI_7
DOWNS
UCLA_531
WU24
HCEG_07406
HCEG_07407
HCEG_07408
HCEG_07409
HCEG_07410
HCEG_07411
HCEG_07412
HCEG_07413
HCEG_07414
160000
180000
200000
220000
240000
260000
Position on Supercontig_2.6

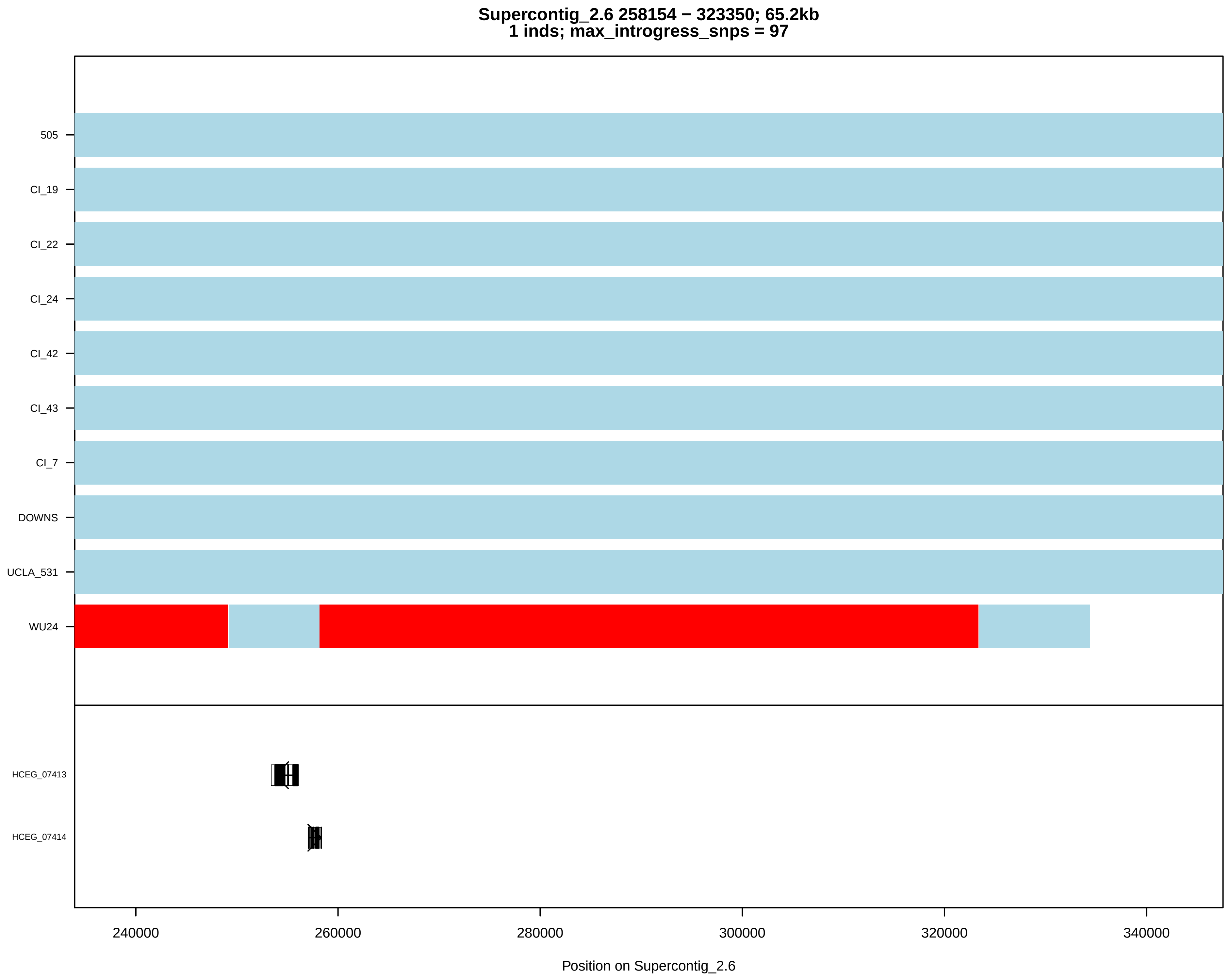

Supercontig_2.6 258154 − 323350; 65.2kb
1 inds; max_introgress_snps = 97
505
CI_19
CI_22
CI_24
CI_42
CI_43
CI_7
DOWNS
UCLA_531
WU24
HCEG_07413
HCEG_07414
240000
260000
280000
300000
320000
340000
Position on Supercontig_2.6

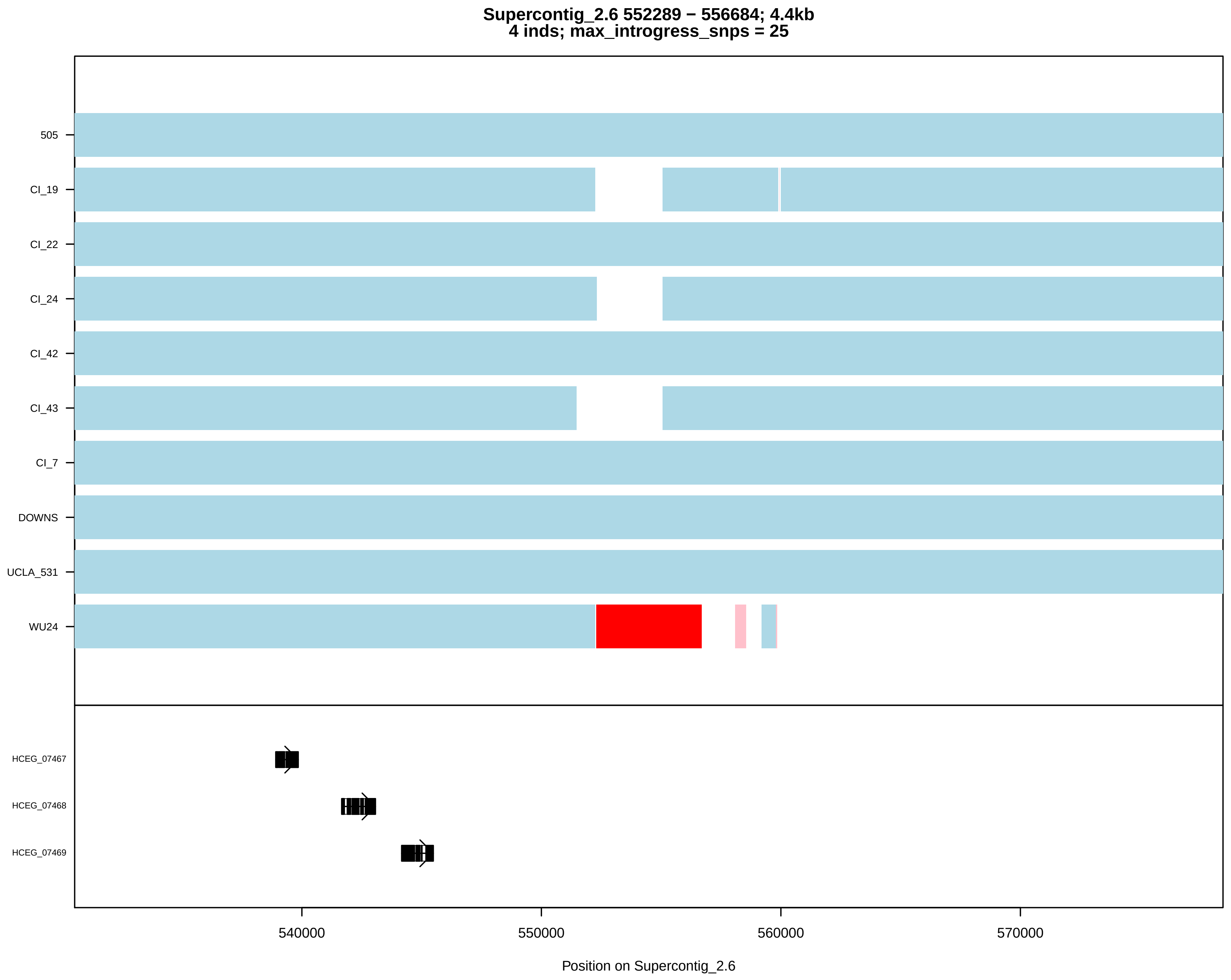

Supercontig_2.6 552289 − 556684; 4.4kb
4 inds; max_introgress_snps = 25
505
CI_19
CI_22
CI_24
CI_42
CI_43
CI_7
DOWNS
UCLA_531
WU24
HCEG_07467
HCEG_07468
HCEG_07469
540000
550000
560000
570000
Position on Supercontig_2.6

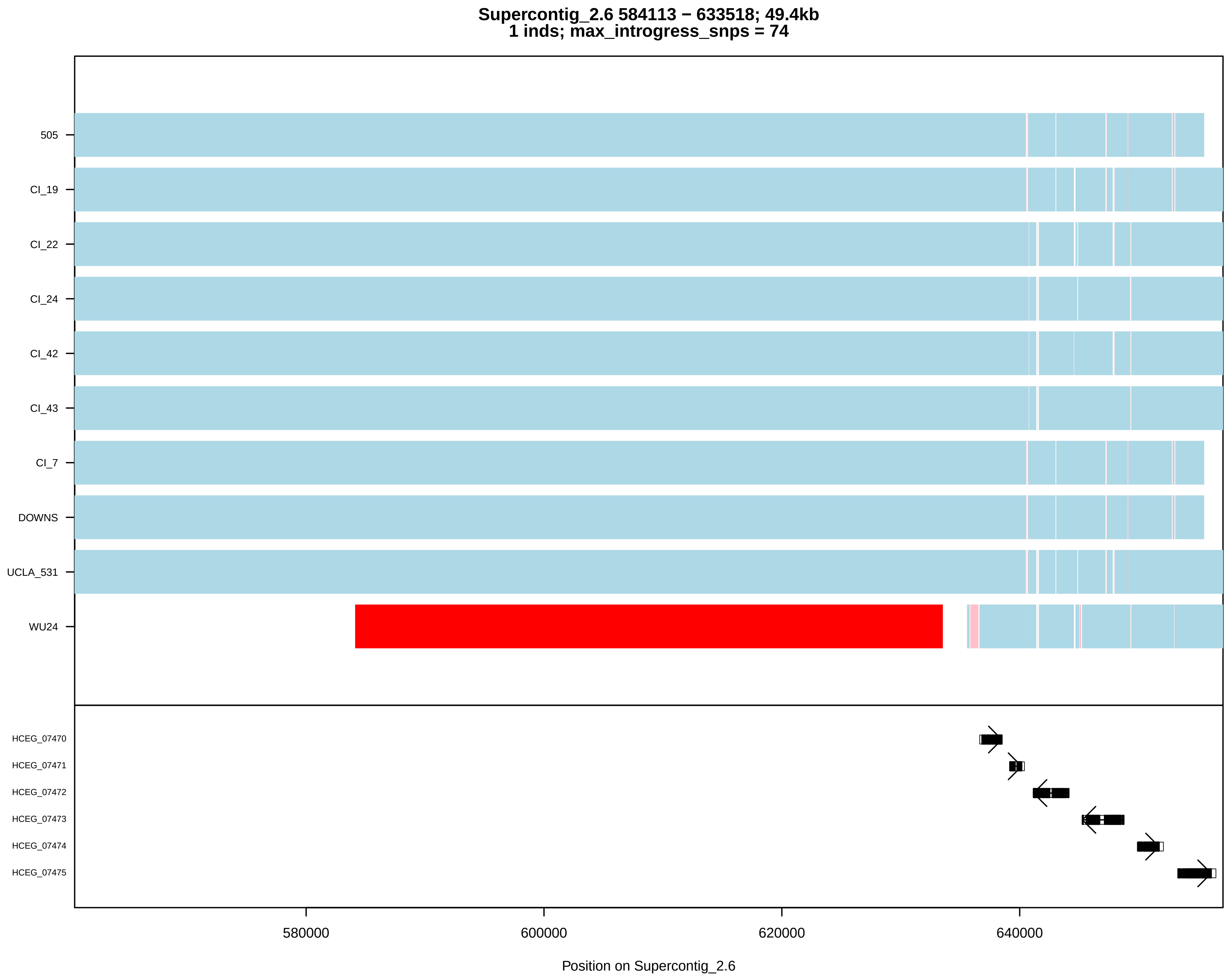

Supercontig_2.6 584113 − 633518; 49.4kb
1 inds; max_introgress_snps = 74
505
CI_19
CI_22
CI_24
CI_42
CI_43
CI_7
DOWNS
UCLA_531
WU24
HCEG_07470
HCEG_07471
HCEG_07472
HCEG_07473
HCEG_07474
HCEG_07475
580000
600000
620000
640000
Position on Supercontig_2.6

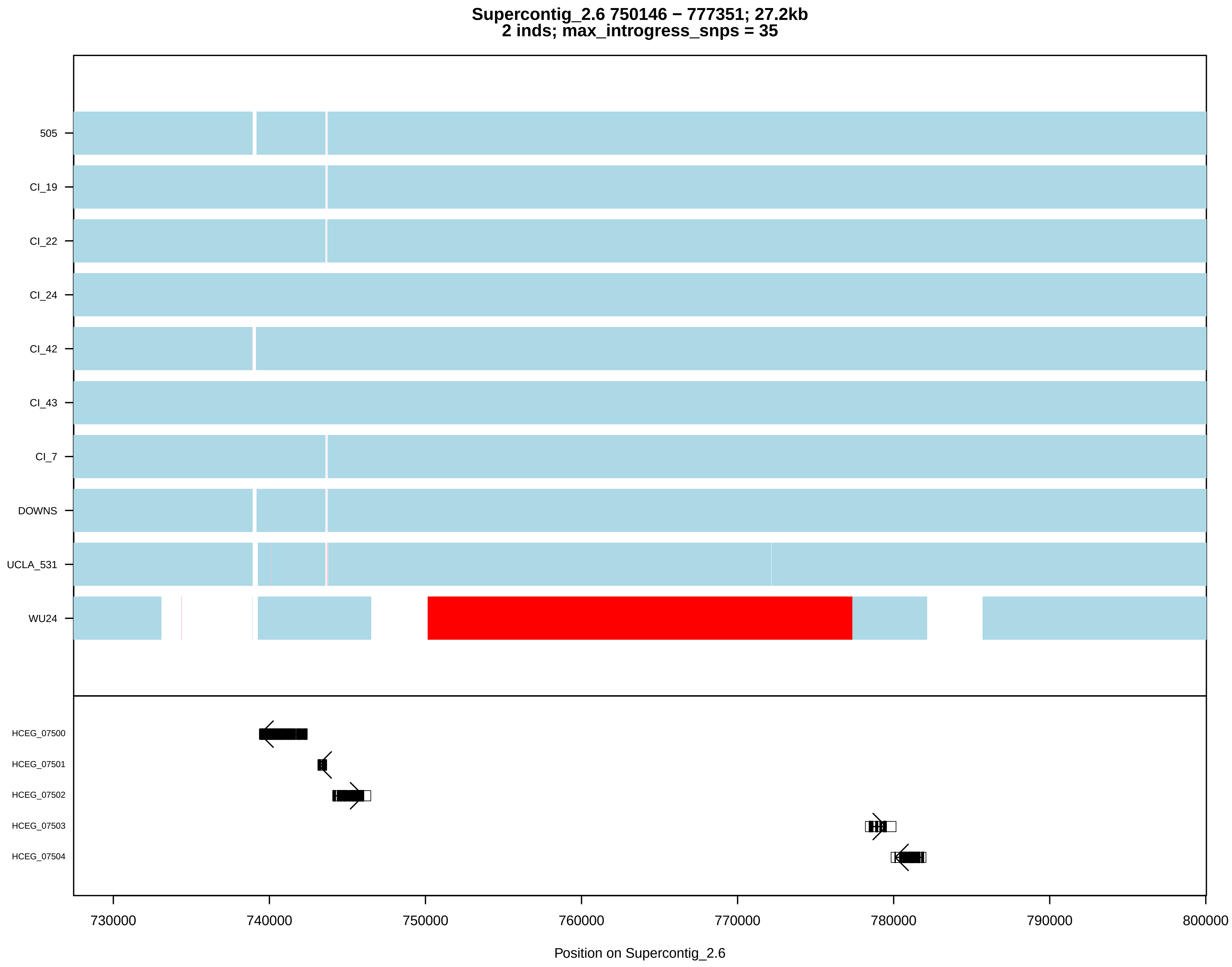
Supercontig_2.6 750146 − 777351; 27.2kb
2 inds; max_introgress_snps = 35
505
CI_19
CI_22
CI_24
CI_42
CI_43
CI_7
DOWNS
UCLA_531
WU24
HCEG_07500
HCEG_07501
HCEG_07502
HCEG_07503
HCEG_07504
730000
740000
750000
760000
770000
780000
790000
800000
Position on Supercontig_2.6

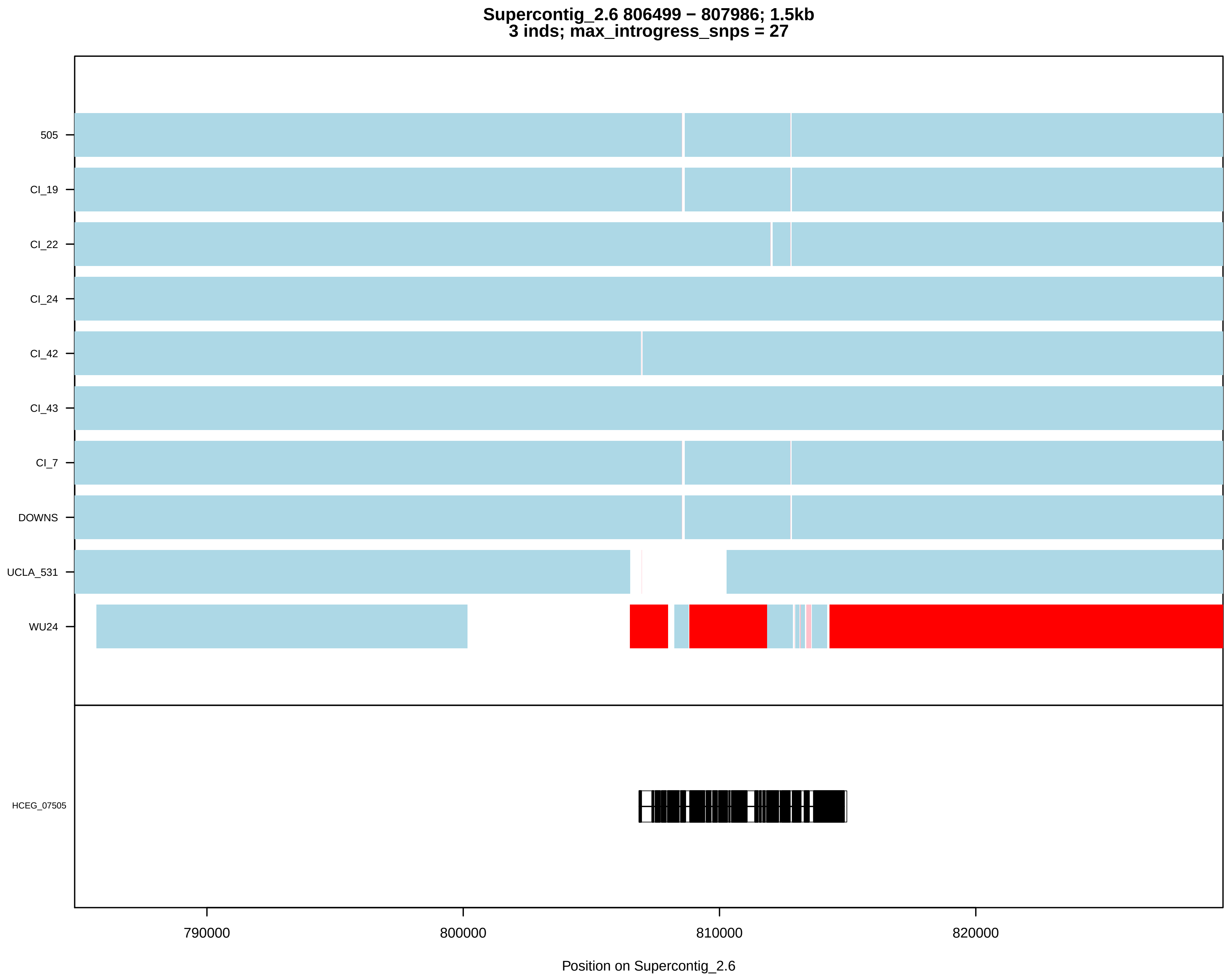

Supercontig_2.6 806499 − 807986; 1.5kb
3 inds; max_introgress_snps = 27
505
CI_19
CI_22
CI_24
CI_42
CI_43
CI_7
DOWNS
UCLA_531
WU24
HCEG_07505
790000
800000
810000
820000
Position on Supercontig_2.6

Supercontig_2.6 808830 − 811858; 3kb
1 inds; max_introgress_snps = 36

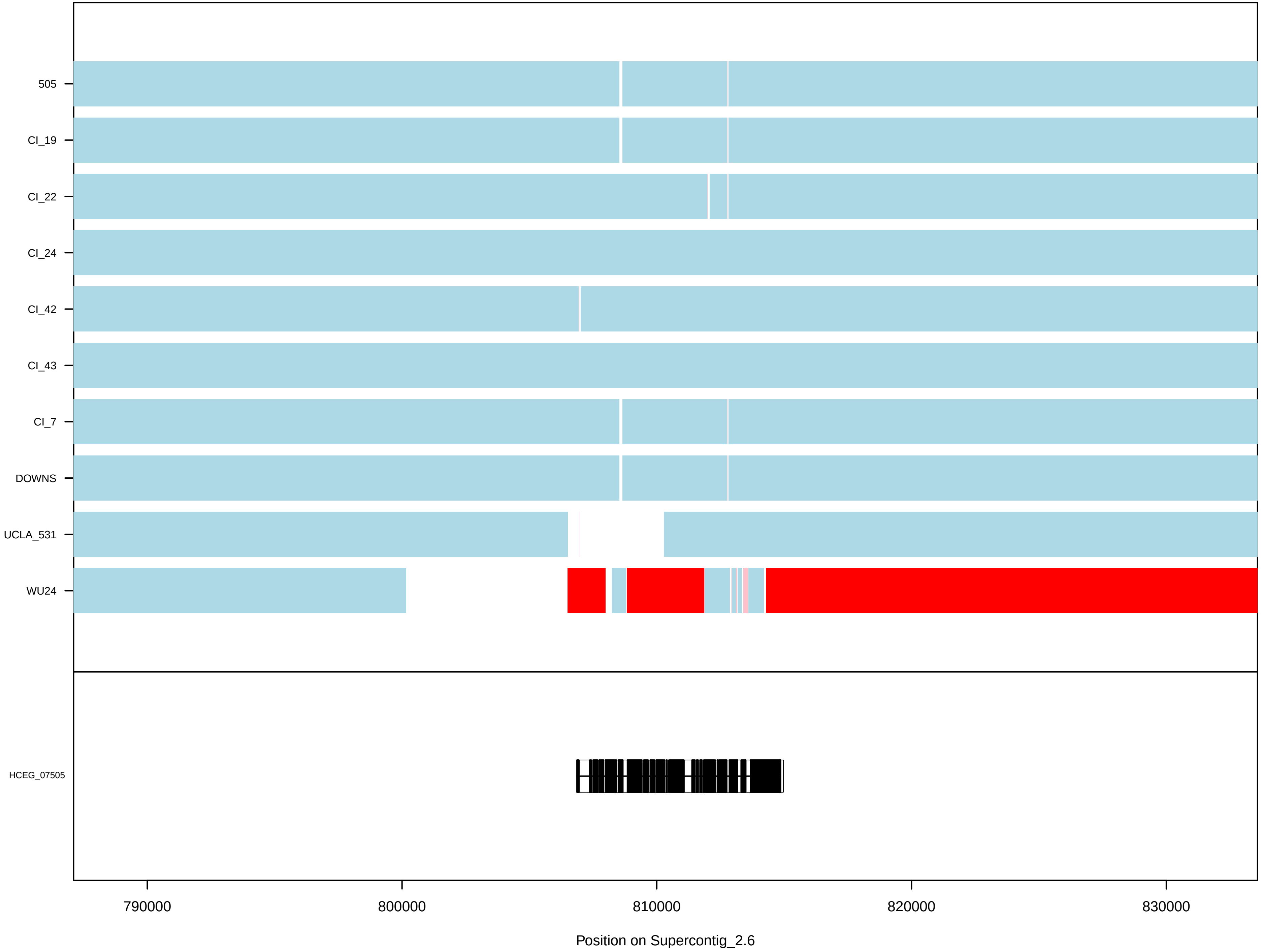

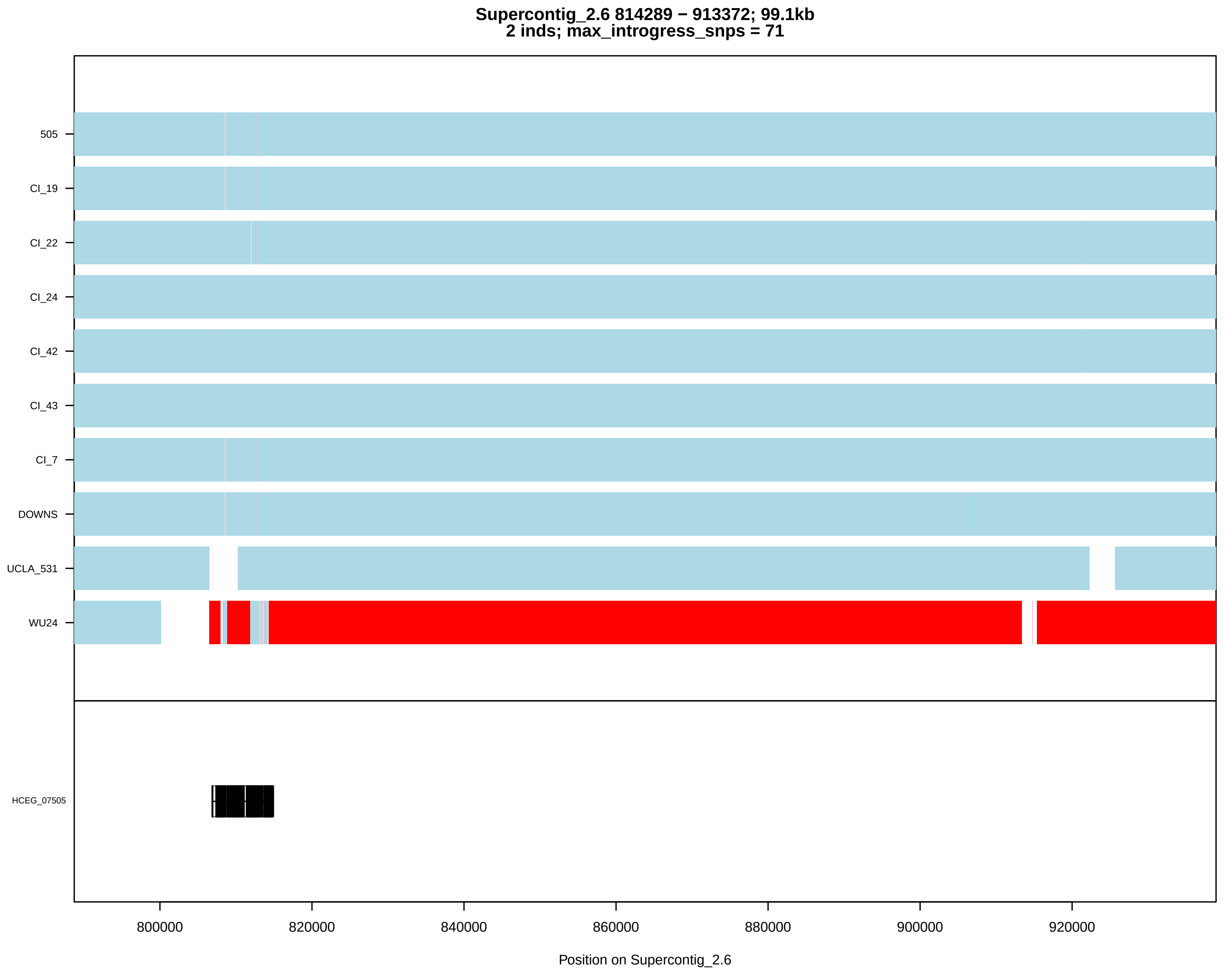
Supercontig_2.6 814289 − 913372; 99.1kb
2 inds; max_introgress_snps = 71
505
CI_19
CI_22
CI_24
CI_42
CI_43
CI_7
DOWNS
UCLA_531
WU24
HCEG_07505
800000
820000
840000
860000
880000
900000
920000
Position on Supercontig_2.6

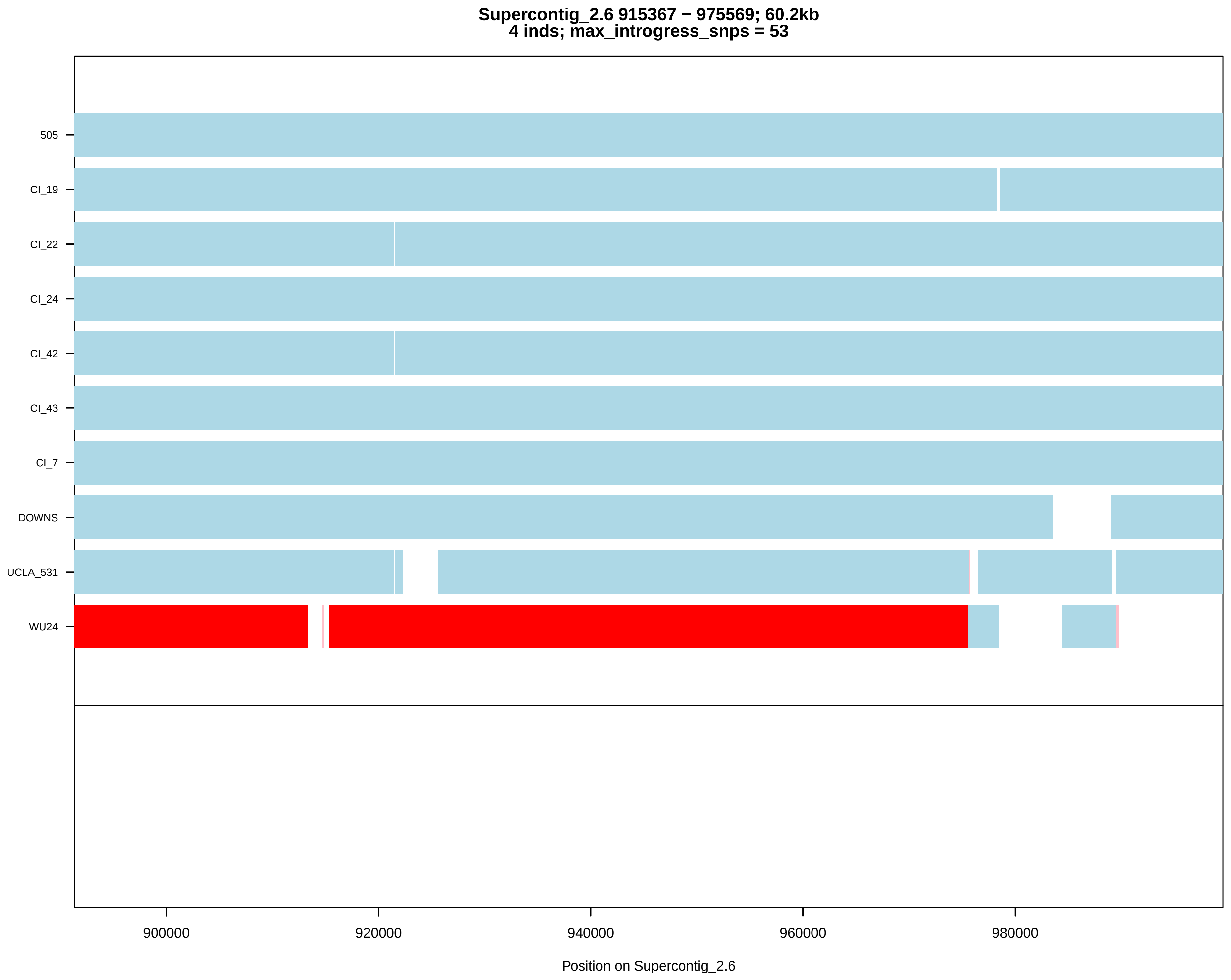
Supercontig_2.6 915367 − 975569; 60.2kb
4 inds; max_introgress_snps = 53
505
CI_19
CI_22
CI_24
CI_42
CI_43
CI_7
DOWNS
UCLA_531
WU24
900000
920000
940000
960000
980000
Position on Supercontig_2.6

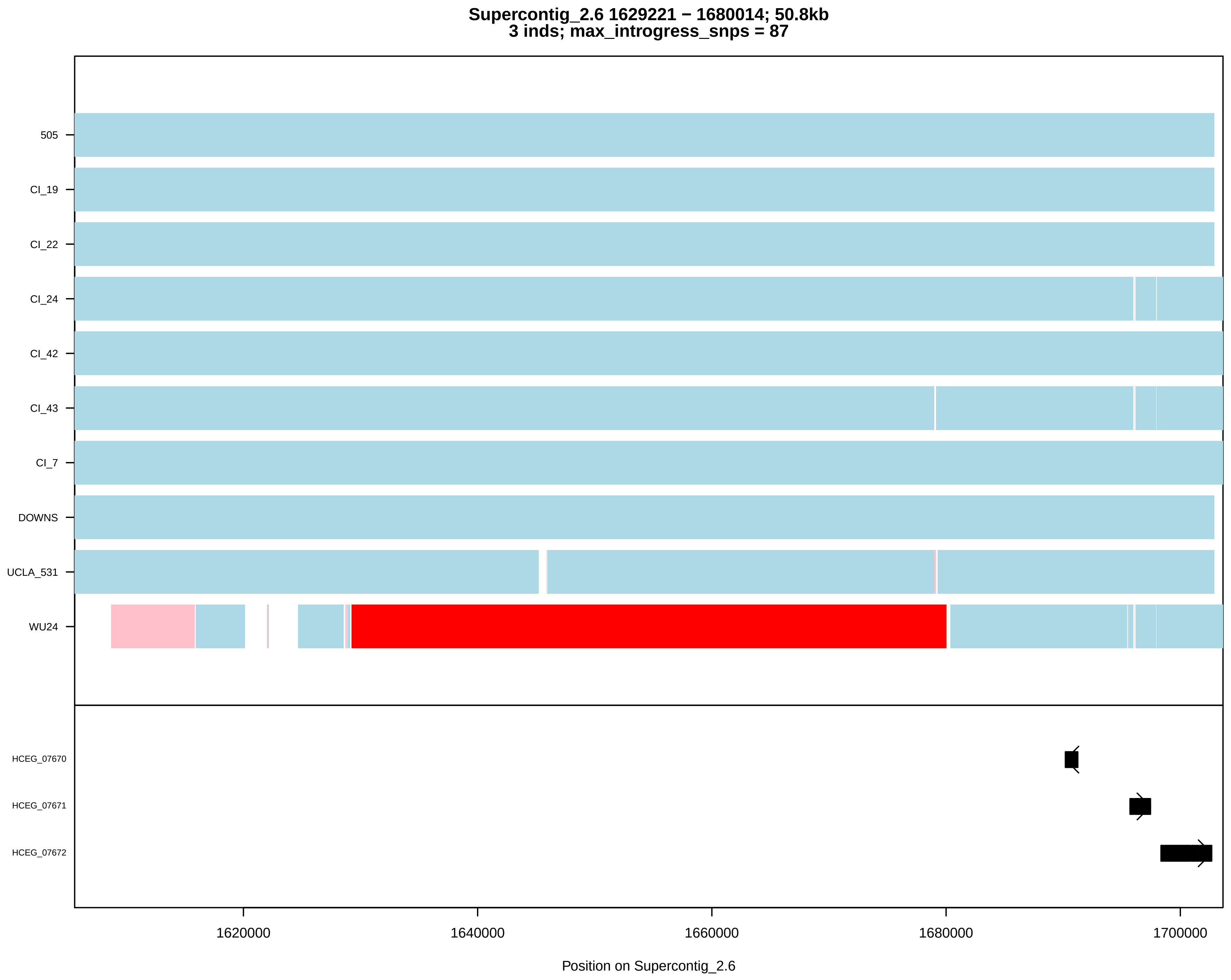

Supercontig_2.6 1629221 − 1680014; 50.8kb
3 inds; max_introgress_snps = 87
505
CI_19
CI_22
CI_24
CI_42
CI_43
CI_7
DOWNS
UCLA_531
WU24
HCEG_07670
HCEG_07671
HCEG_07672
1620000
1640000
1660000
1680000
1700000
Position on Supercontig_2.6

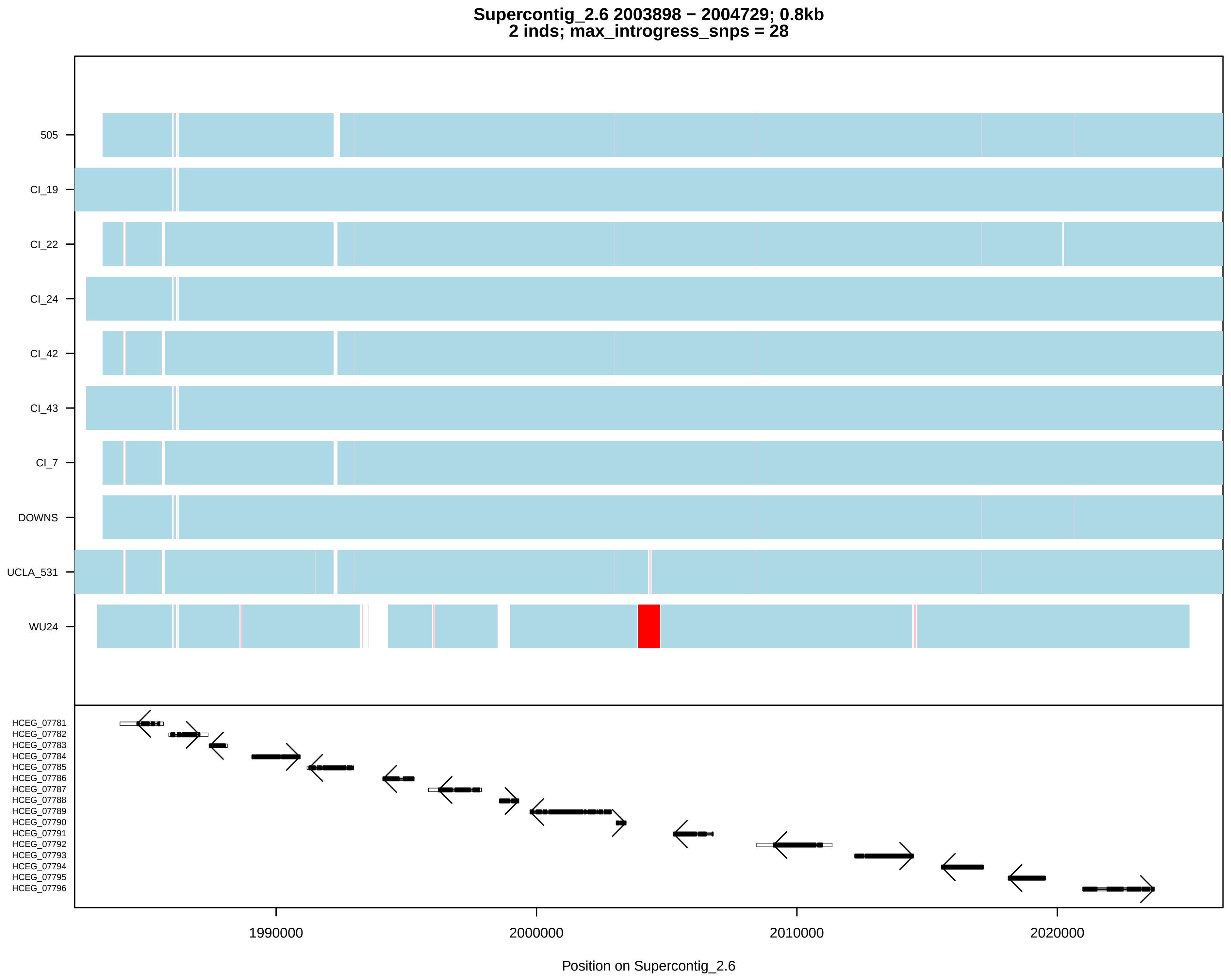
Supercontig_2.6 2003898 − 2004729; 0.8kb
2 inds; max_introgress_snps = 28
505
CI_19
CI_22
CI_24
CI_42
CI_43
CI_7
DOWNS
UCLA_531
WU24
HCEG_07781
HCEG_07782
HCEG_07783
HCEG_07784
HCEG_07785
HCEG_07786
HCEG_07787
HCEG_07788
HCEG_07789
HCEG_07790
HCEG_07791
HCEG_07792
HCEG_07793
HCEG_07794
HCEG_07795
HCEG_07796
1990000
2000000
2010000
2020000
Position on Supercontig_2.6

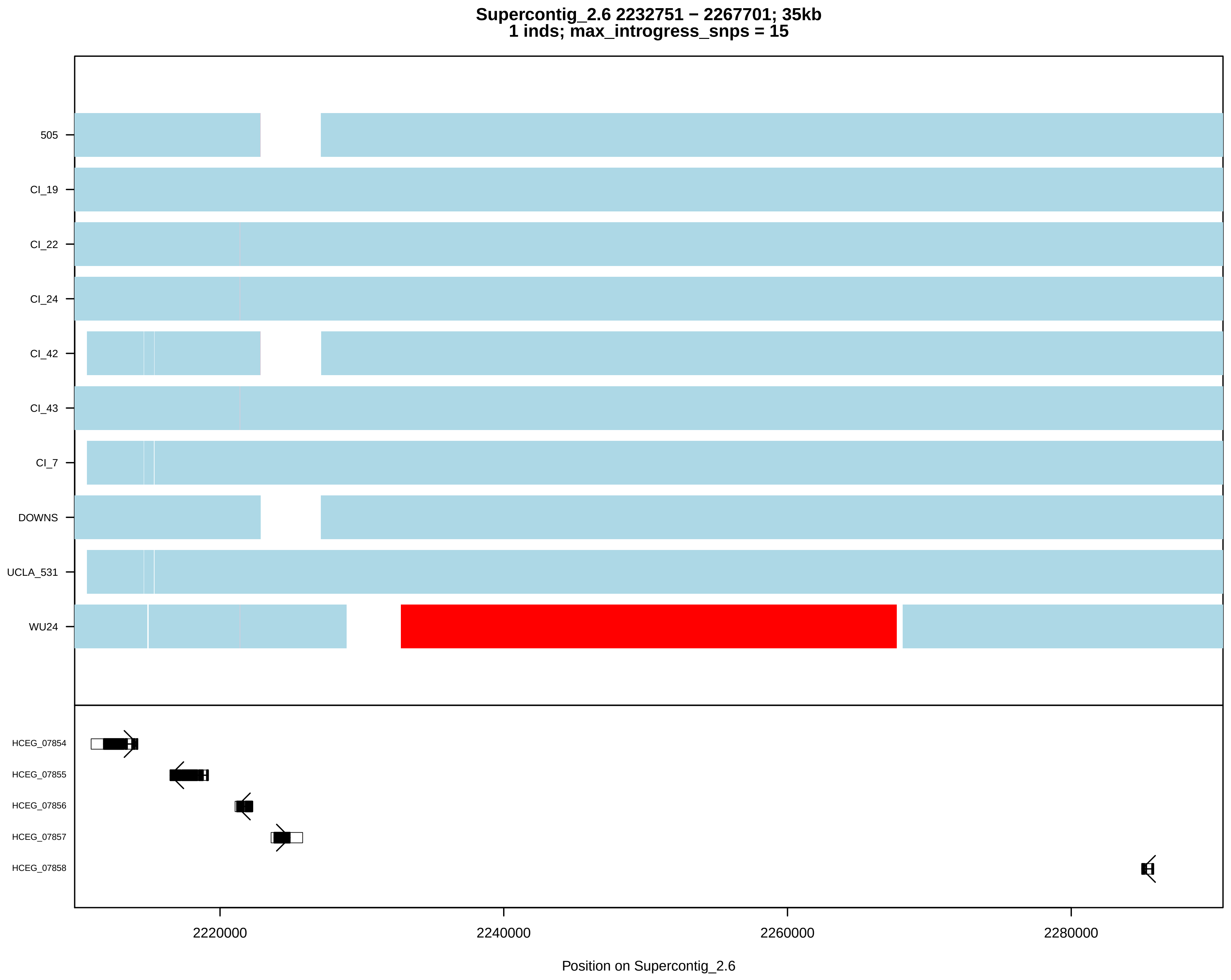

Supercontig_2.6 2232751 − 2267701; 35kb
1 inds; max_introgress_snps = 15
505
CI_19
CI_22
CI_24
CI_42
CI_43
CI_7
DOWNS
UCLA_531
WU24
HCEG_07854
HCEG_07855
HCEG_07856
HCEG_07857
HCEG_07858
2220000
2240000
2260000
2280000
Position on Supercontig_2.6

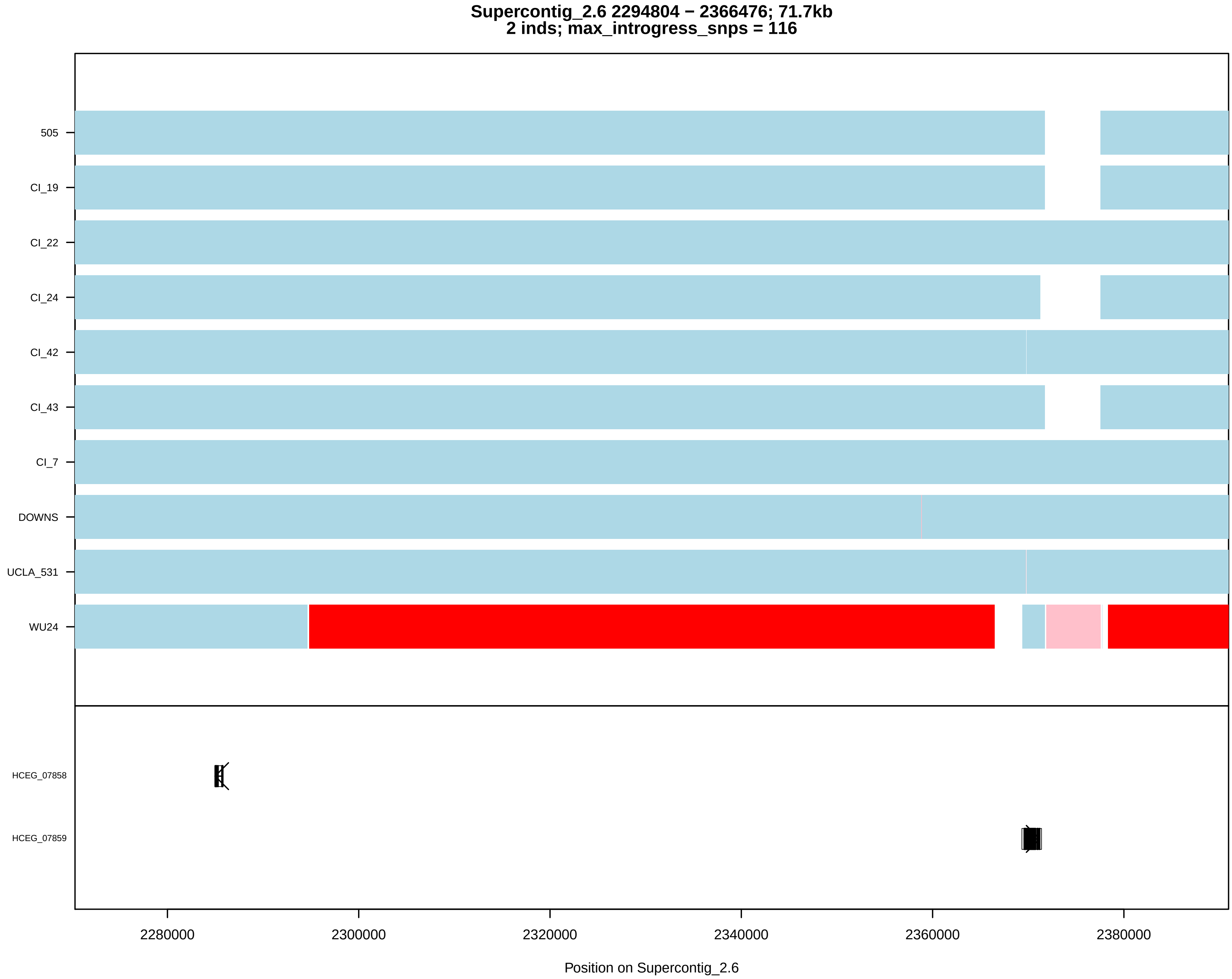

Supercontig_2.6 2294804 − 2366476; 71.7kb
2 inds; max_introgress_snps = 116
505
CI_19
CI_22
CI_24
CI_42
CI_43
CI_7
DOWNS
UCLA_531
WU24
HCEG_07858
HCEG_07859
2280000
2300000
2320000
2340000
2360000
2380000
Position on Supercontig_2.6

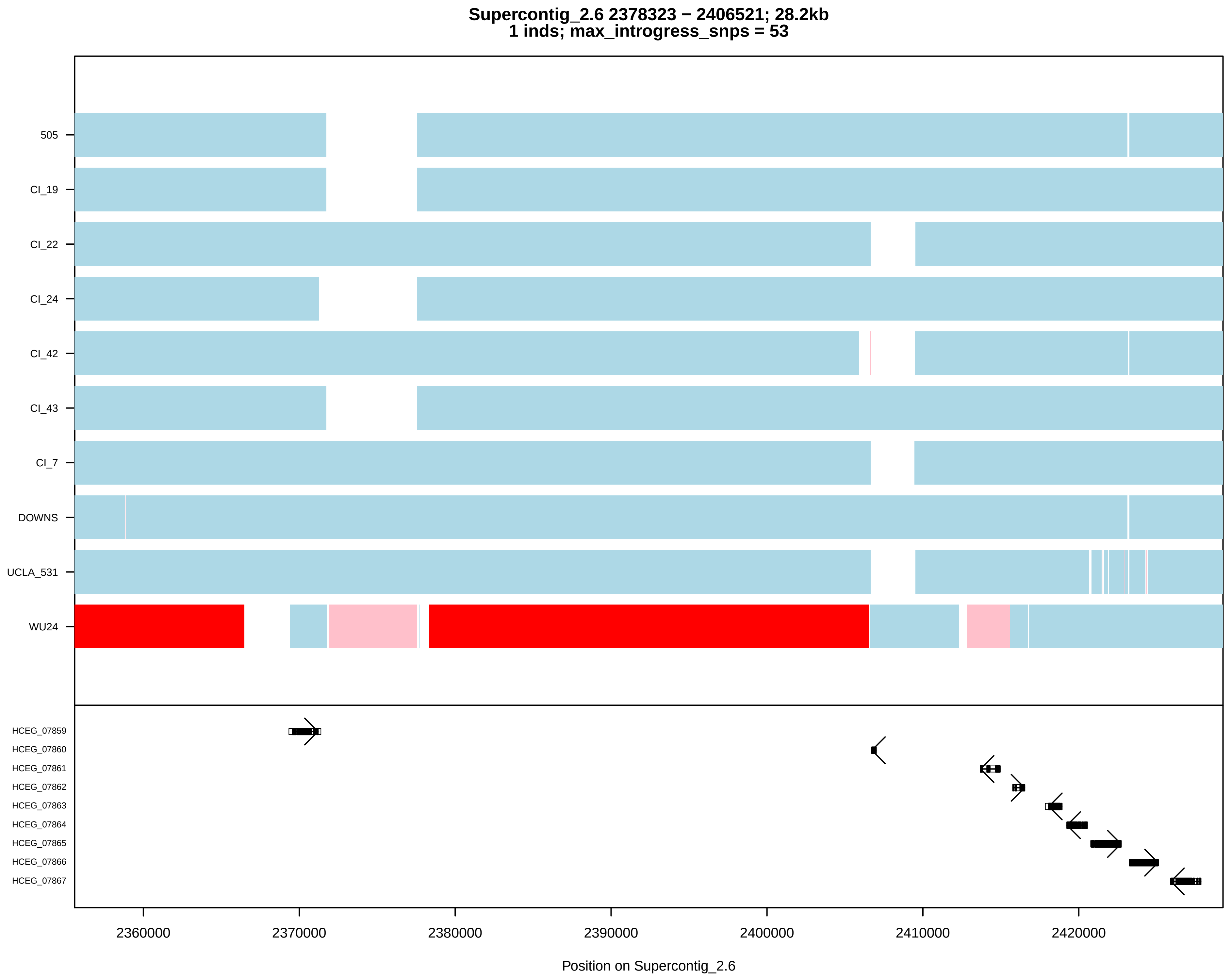

Supercontig_2.6 2378323 − 2406521; 28.2kb
1 inds; max_introgress_snps = 53
505
CI_19
CI_22
CI_24
CI_42
CI_43
CI_7
DOWNS
UCLA_531
WU24
HCEG_07859
HCEG_07860
HCEG_07861
HCEG_07862
HCEG_07863
HCEG_07864
HCEG_07865
HCEG_07866
HCEG_07867
2360000
2370000
2380000
2390000
2400000
2410000
2420000
Position on Supercontig_2.6

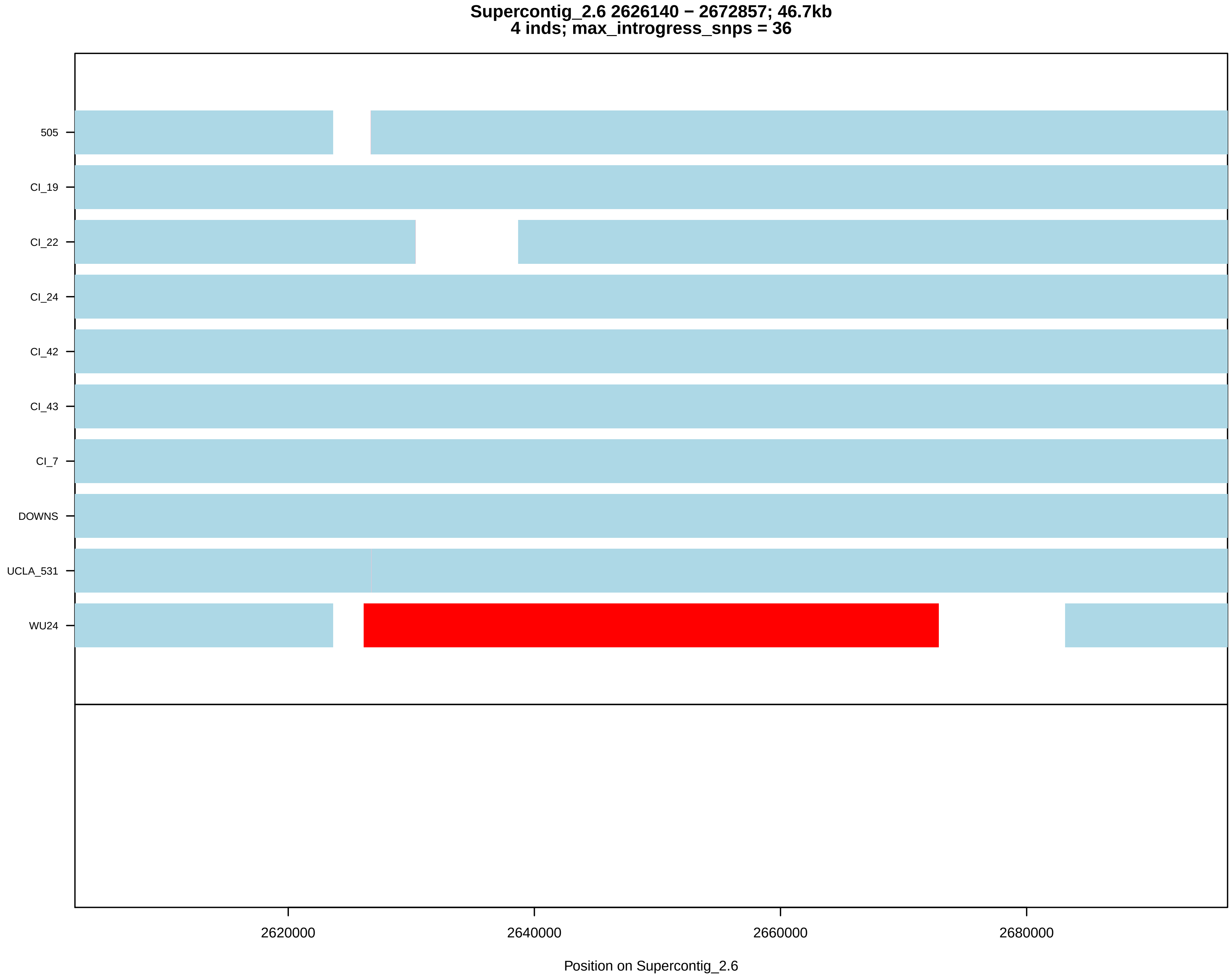
Supercontig_2.6 2626140 − 2672857; 46.7kb
4 inds; max_introgress_snps = 36
505
CI_19
CI_22
CI_24
CI_42
CI_43
CI_7
DOWNS
UCLA_531
WU24
2620000
2640000
2660000
2680000
Position on Supercontig_2.6

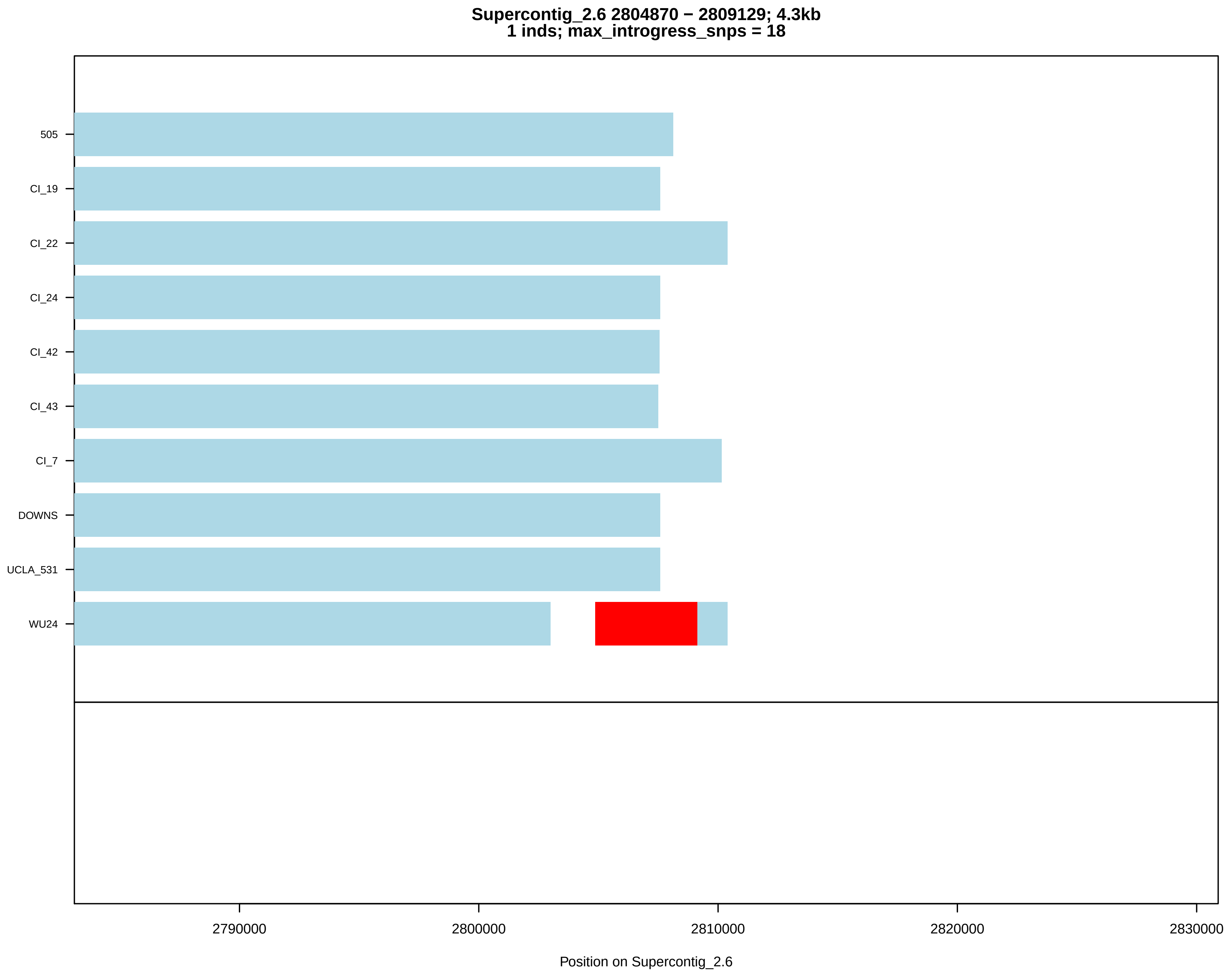

Supercontig_2.6 2804870 − 2809129; 4.3kb
1 inds; max_introgress_snps = 18
505
CI_19
CI_22
CI_24
CI_42
CI_43
CI_7
DOWNS
UCLA_531
WU24
2790000
2800000
2810000
2820000
2830000
Position on Supercontig_2.6

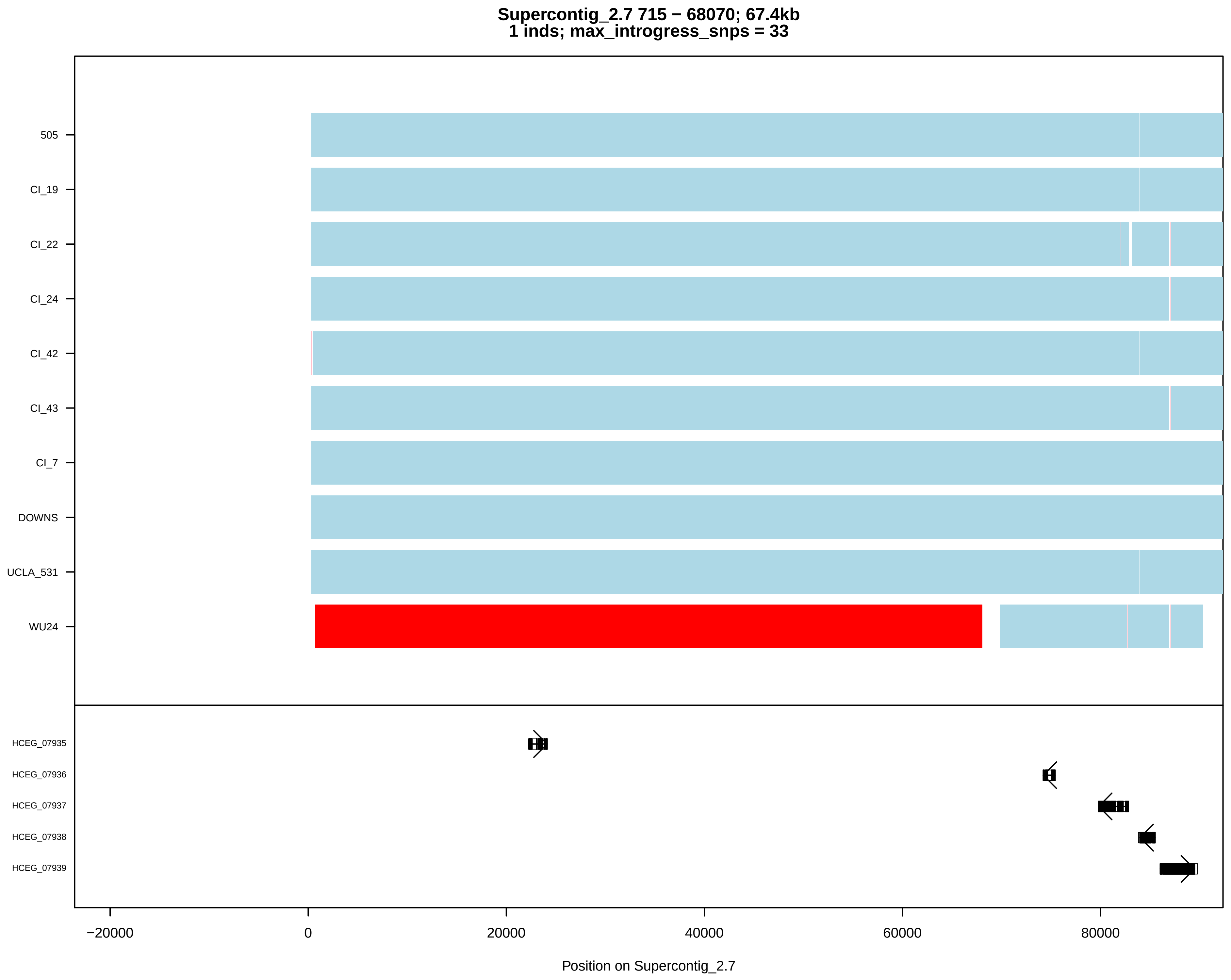

Supercontig_2.7 715 − 68070; 67.4kb
1 inds; max_introgress_snps = 33
505
CI_19
CI_22
CI_24
CI_42
CI_43
CI_7
DOWNS
UCLA_531
WU24
HCEG_07935
HCEG_07936
HCEG_07937
HCEG_07938
HCEG_07939
−20000
0
20000
40000
60000
80000
Position on Supercontig_2.7

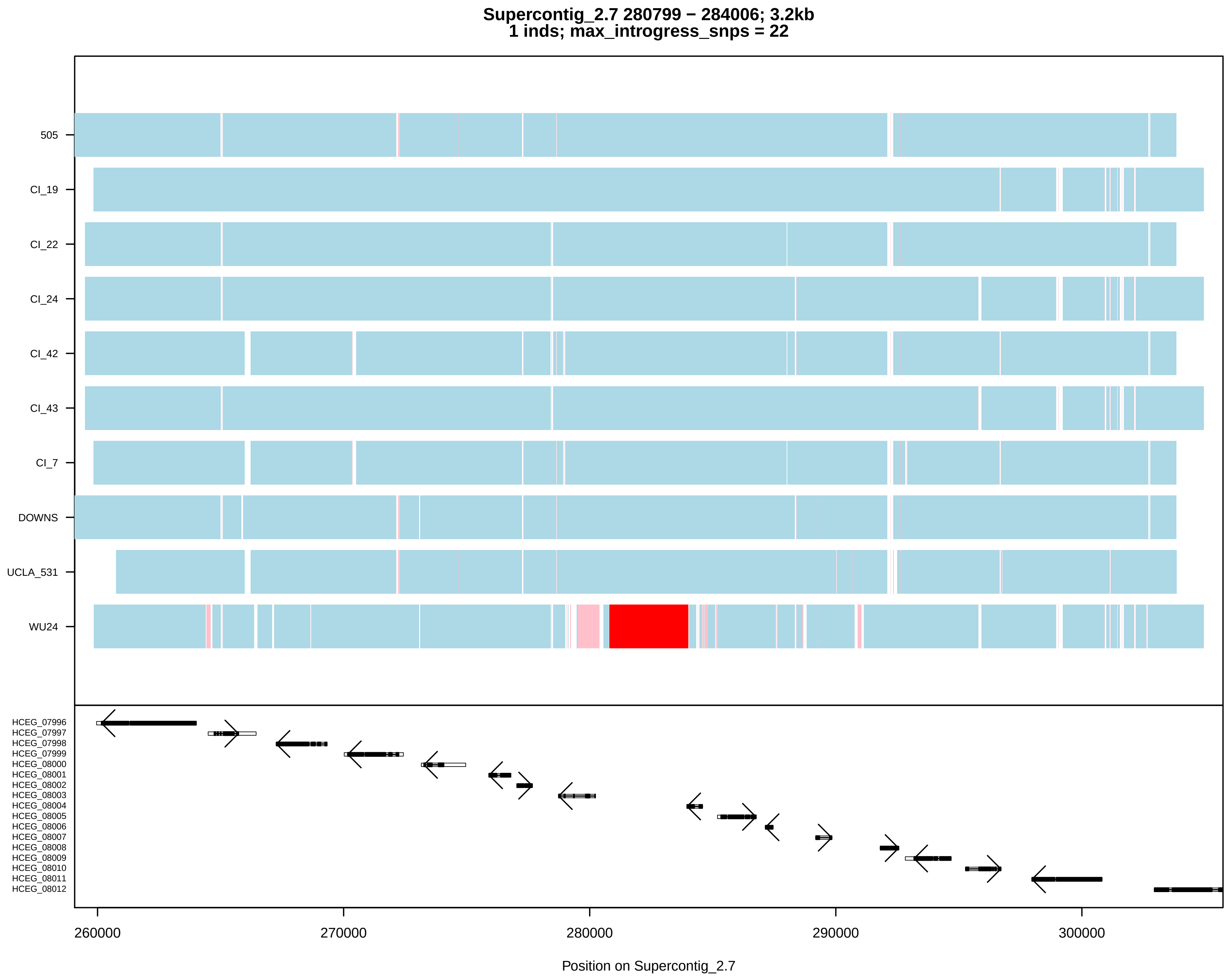
Supercontig_2.7 280799 − 284006; 3.2kb
1 inds; max_introgress_snps = 22
505
CI_19
CI_22
CI_24
CI_42
CI_43
CI_7
DOWNS
UCLA_531
WU24
HCEG_07996
HCEG_07997
HCEG_07998
HCEG_07999
HCEG_08000
HCEG_08001
HCEG_08002
HCEG_08003
HCEG_08004
HCEG_08005
HCEG_08006
HCEG_08007
HCEG_08008
HCEG_08009
HCEG_08010
HCEG_08011
HCEG_08012
260000
270000
280000
290000
300000
Position on Supercontig_2.7

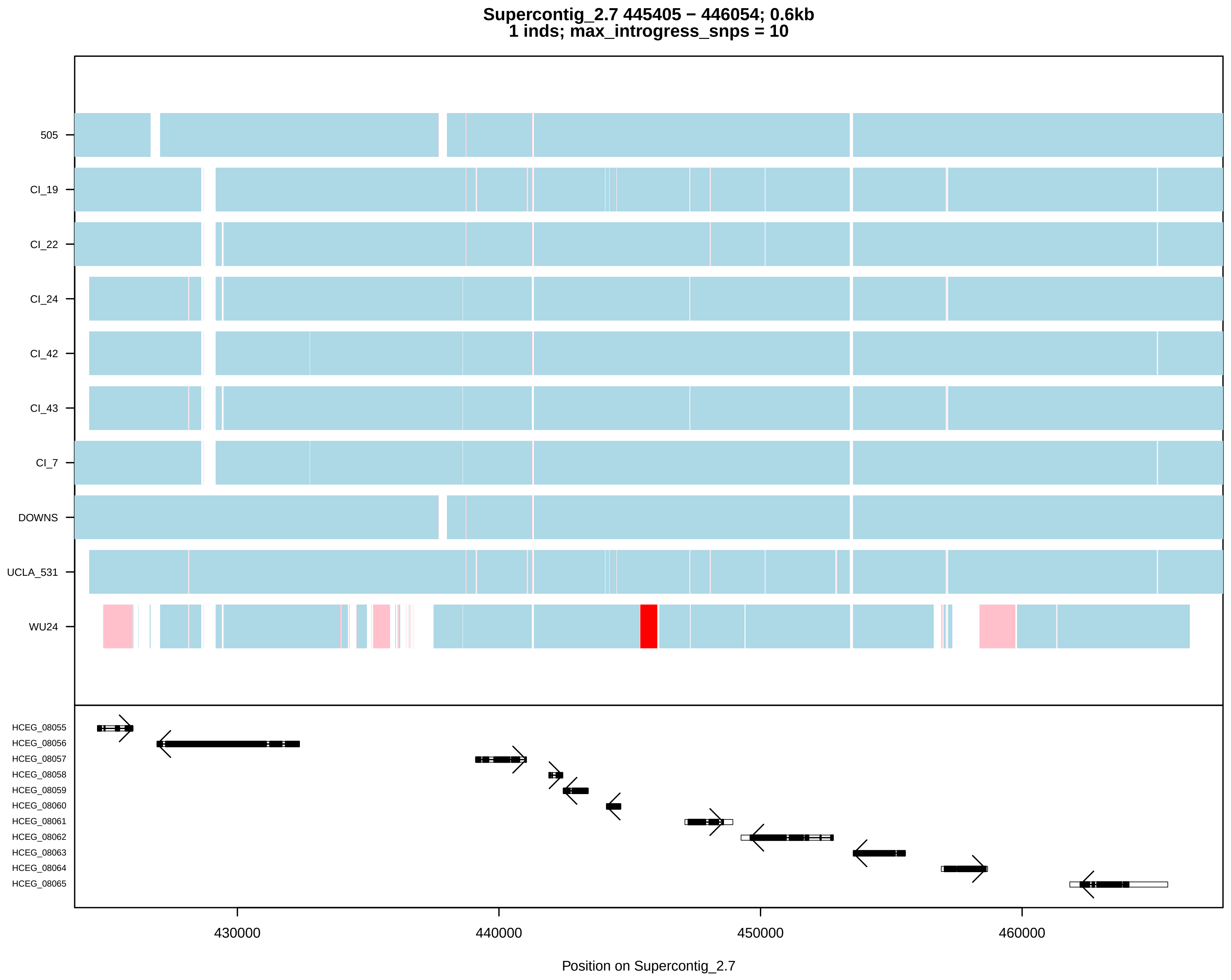

Supercontig_2.7 445405 − 446054; 0.6kb
1 inds; max_introgress_snps = 10
505
CI_19
CI_22
CI_24
CI_42
CI_43
CI_7
DOWNS
UCLA_531
WU24
HCEG_08055
HCEG_08056
HCEG_08057
HCEG_08058
HCEG_08059
HCEG_08060
HCEG_08061
HCEG_08062
HCEG_08063
HCEG_08064
HCEG_08065
430000
440000
450000
460000
Position on Supercontig_2.7

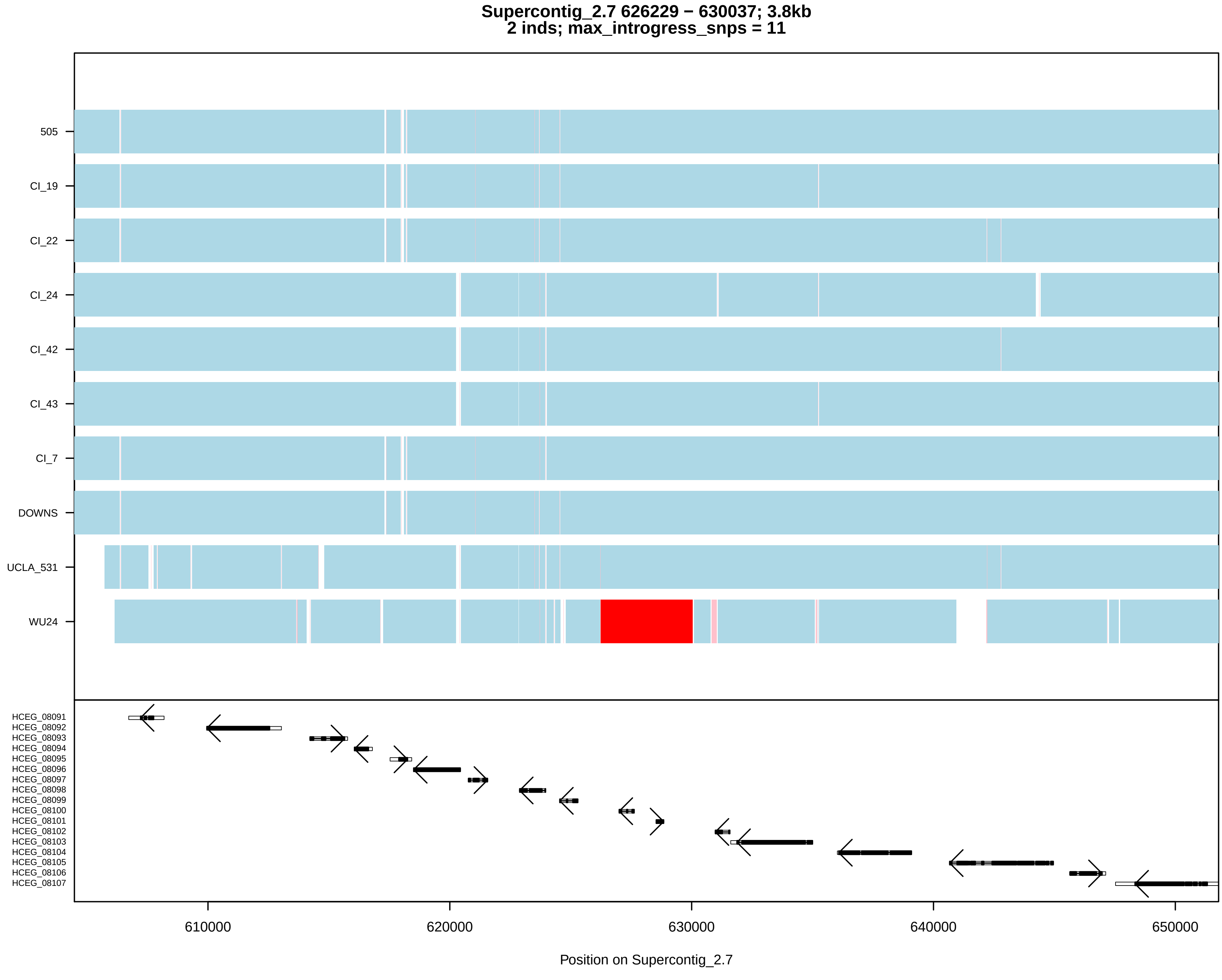
Supercontig_2.7 626229 − 630037; 3.8kb
2 inds; max_introgress_snps = 11
505
CI_19
CI_22
CI_24
CI_42
CI_43
CI_7
DOWNS
UCLA_531
WU24
HCEG_08091
HCEG_08092
HCEG_08093
HCEG_08094
HCEG_08095
HCEG_08096
HCEG_08097
HCEG_08098
HCEG_08099
HCEG_08100
HCEG_08101
HCEG_08102
HCEG_08103
HCEG_08104
HCEG_08105
HCEG_08106
HCEG_08107
610000
620000
630000
640000
650000
Position on Supercontig_2.7

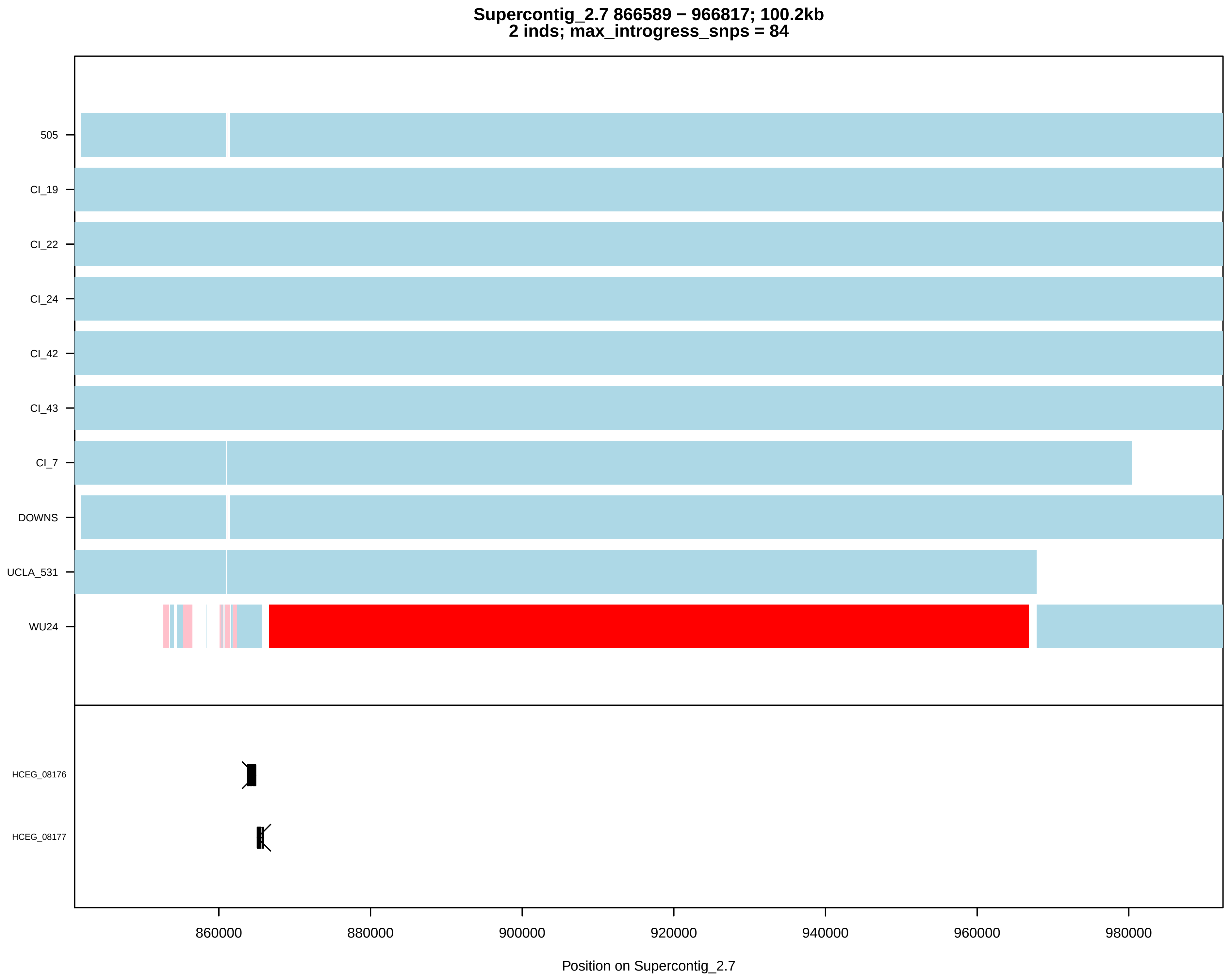
Supercontig_2.7 866589 − 966817; 100.2kb
2 inds; max_introgress_snps = 84
505
CI_19
CI_22
CI_24
CI_42
CI_43
CI_7
DOWNS
UCLA_531
WU24
HCEG_08176
HCEG_08177
860000
880000
900000
920000
940000
960000
980000
Position on Supercontig_2.7

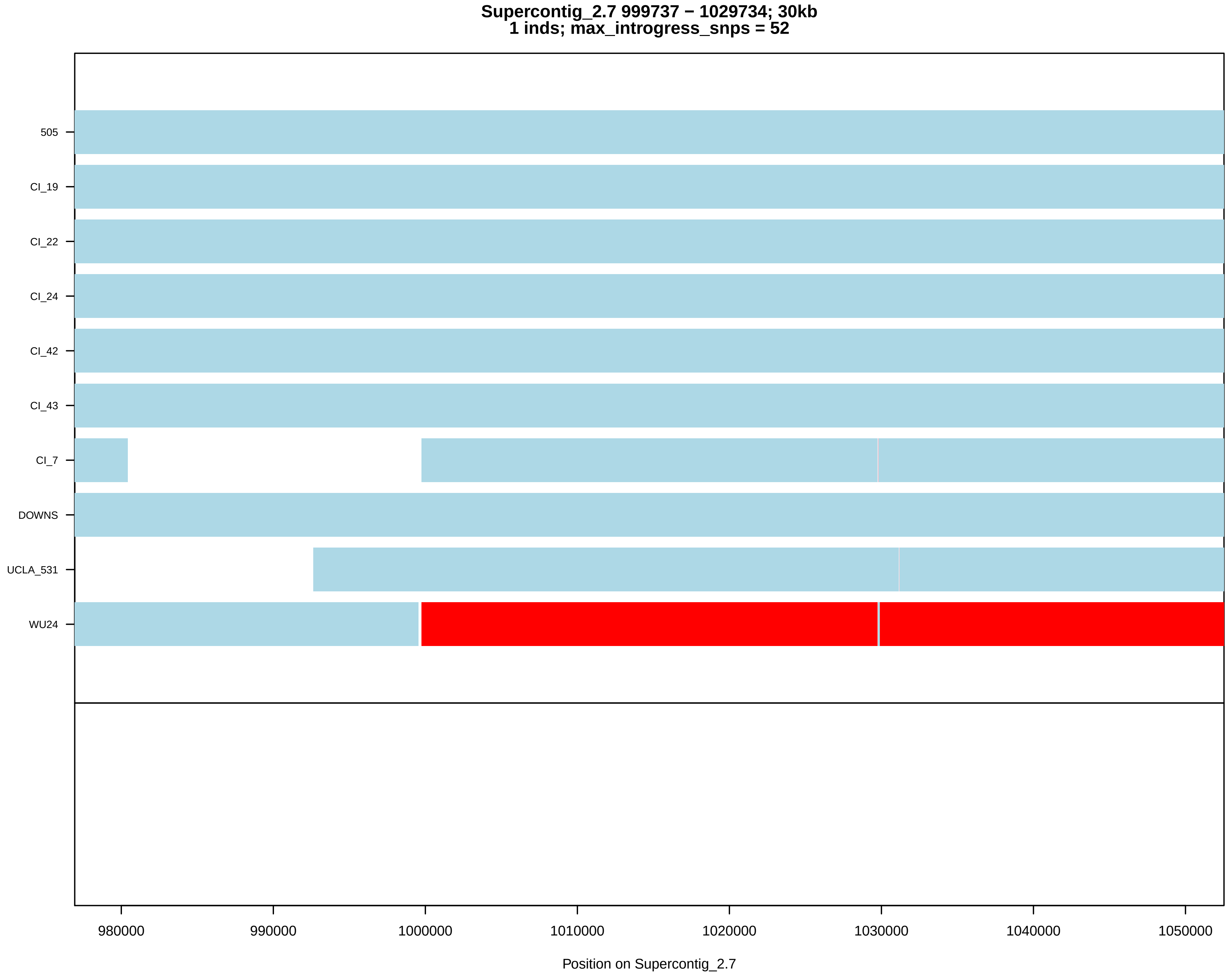
Supercontig_2.7 999737 − 1029734; 30kb
1 inds; max_introgress_snps = 52
505
CI_19
CI_22
CI_24
CI_42
CI_43
CI_7
DOWNS
UCLA_531
WU24
980000
990000
1000000
1010000
1020000
1030000
1040000
1050000
Position on Supercontig_2.7

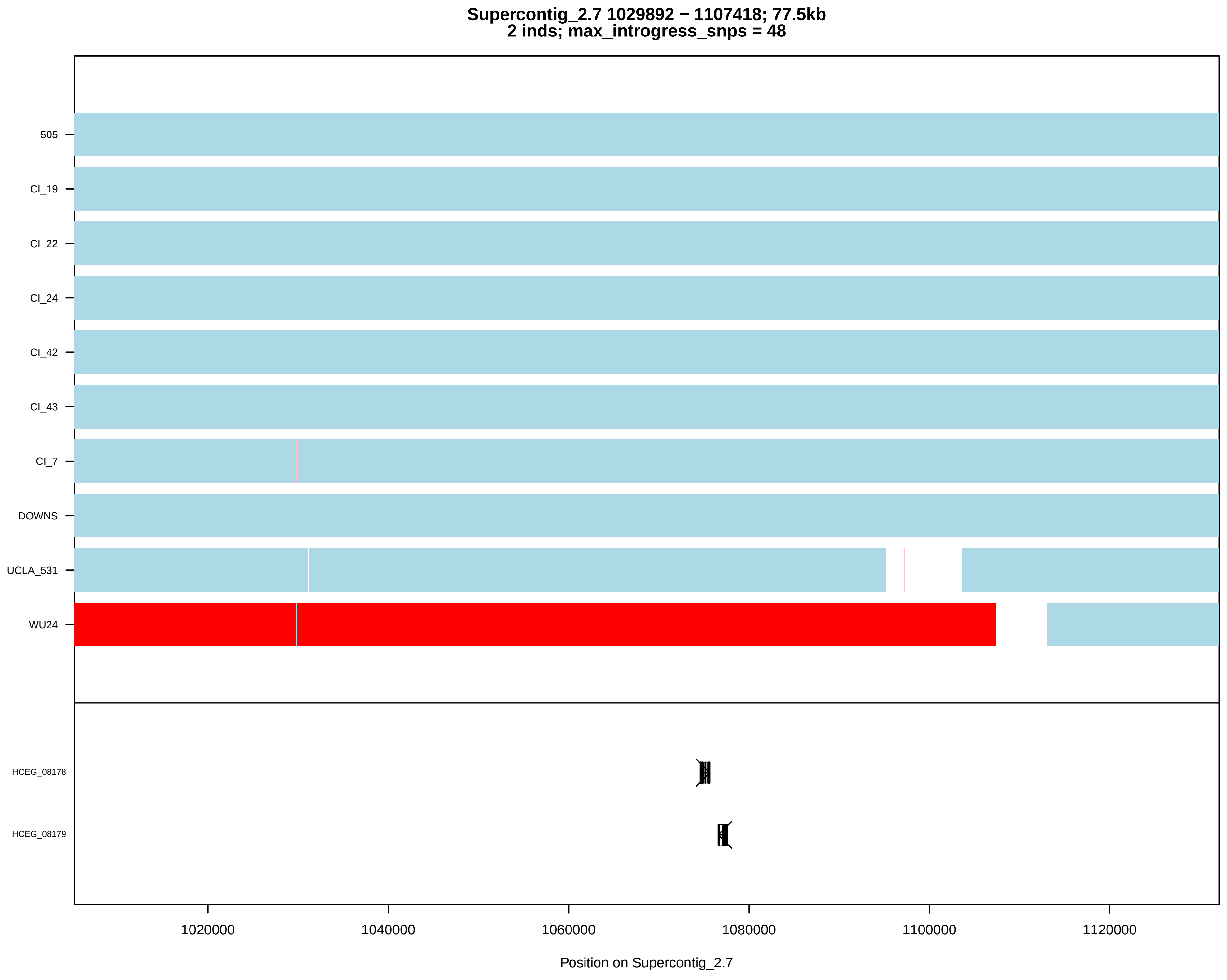

Supercontig_2.7 1029892 − 1107418; 77.5kb
2 inds; max_introgress_snps = 48
505
CI_19
CI_22
CI_24
CI_42
CI_43
CI_7
DOWNS
UCLA_531
WU24
HCEG_08178
HCEG_08179
1020000
1040000
1060000
1080000
1100000
1120000
Position on Supercontig_2.7

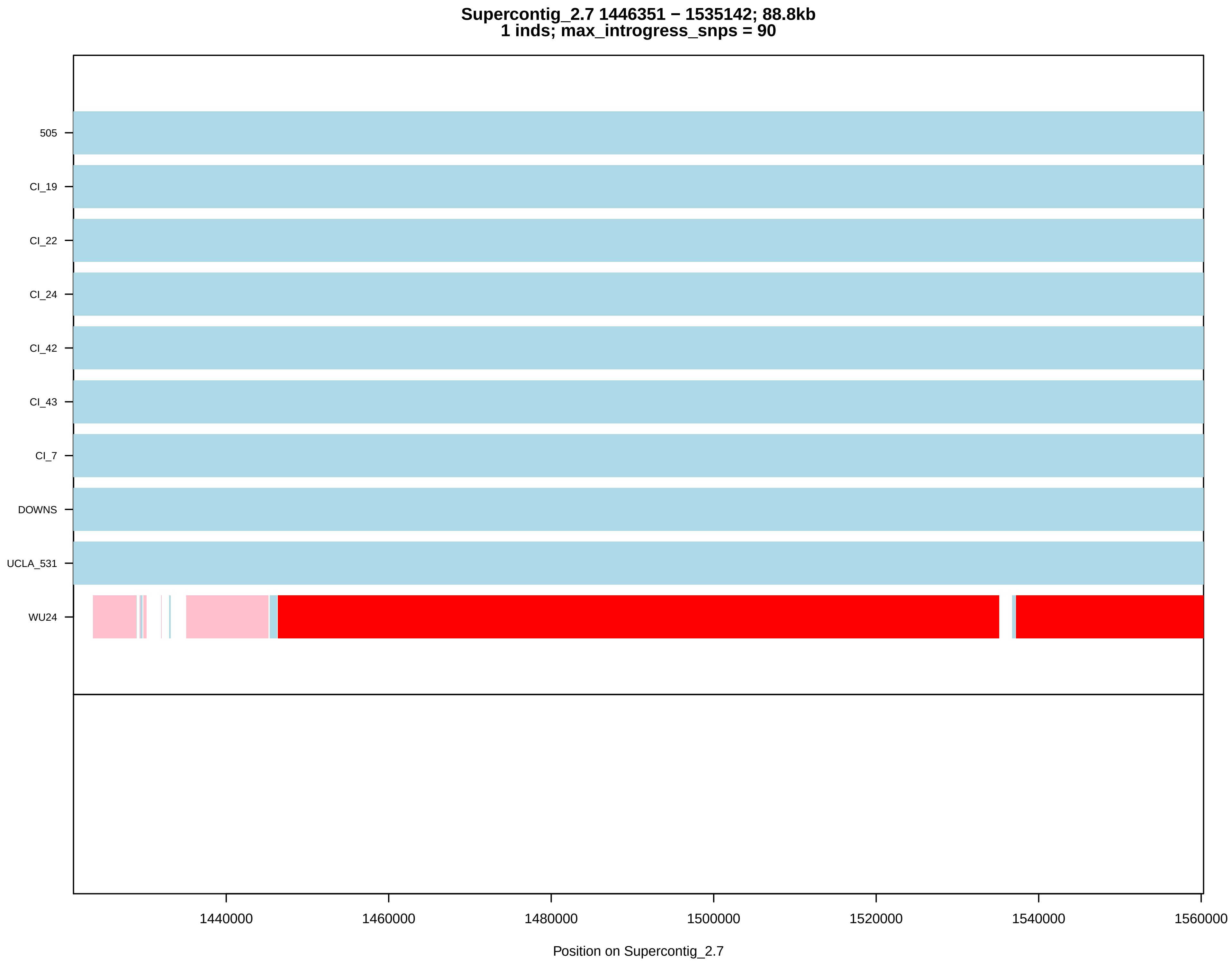

Supercontig_2.7 1446351 − 1535142; 88.8kb
1 inds; max_introgress_snps = 90
505
CI_19
CI_22
CI_24
CI_42
CI_43
CI_7
DOWNS
UCLA_531
WU24
1440000
1460000
1480000
1500000
1520000
1540000
1560000
Position on Supercontig_2.7

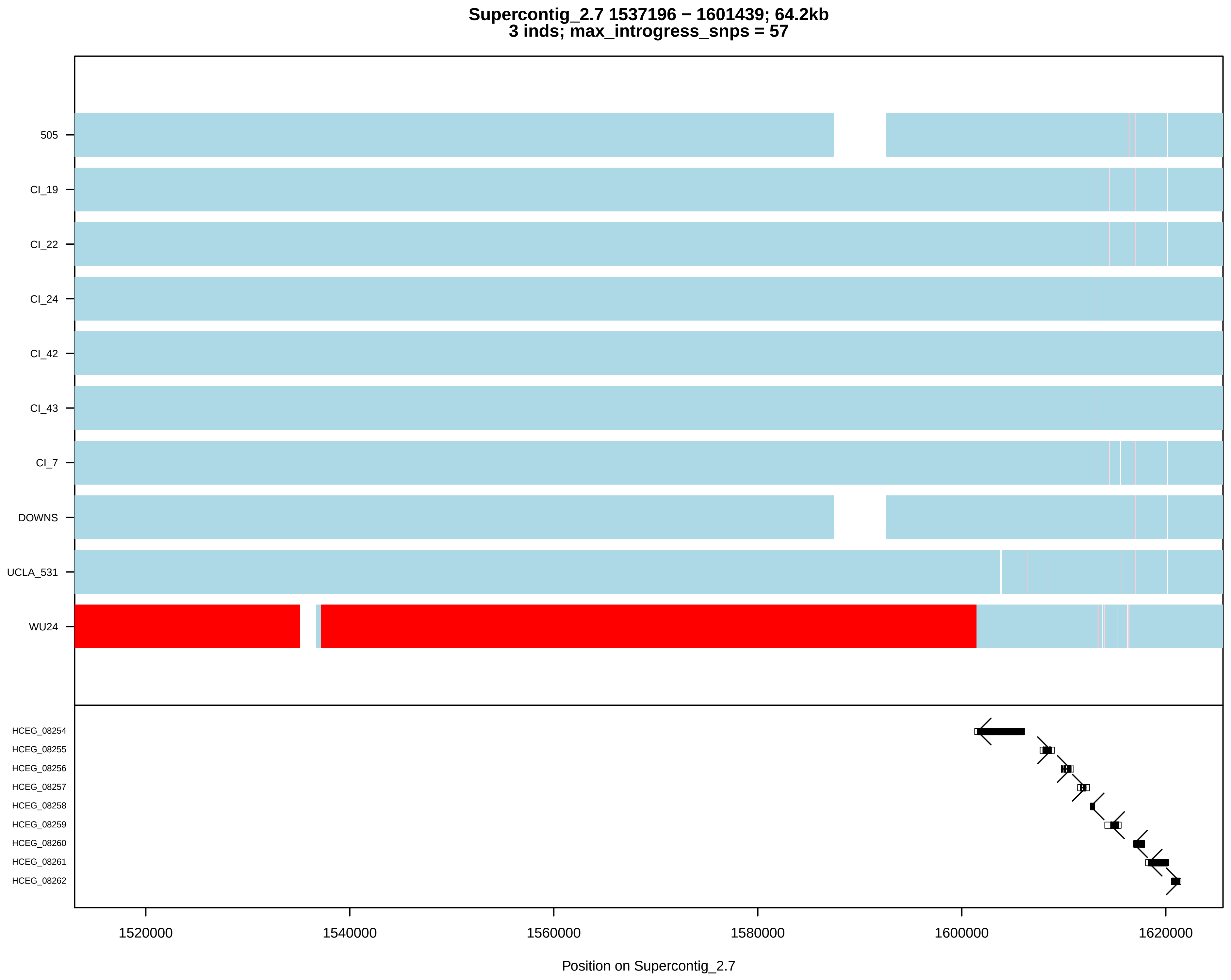
Supercontig_2.7 1537196 − 1601439; 64.2kb
3 inds; max_introgress_snps = 57
505
CI_19
CI_22
CI_24
CI_42
CI_43
CI_7
DOWNS
UCLA_531
WU24
HCEG_08254
HCEG_08255
HCEG_08256
HCEG_08257
HCEG_08258
HCEG_08259
HCEG_08260
HCEG_08261
HCEG_08262
1520000
1540000
1560000
1580000
1600000
1620000
Position on Supercontig_2.7

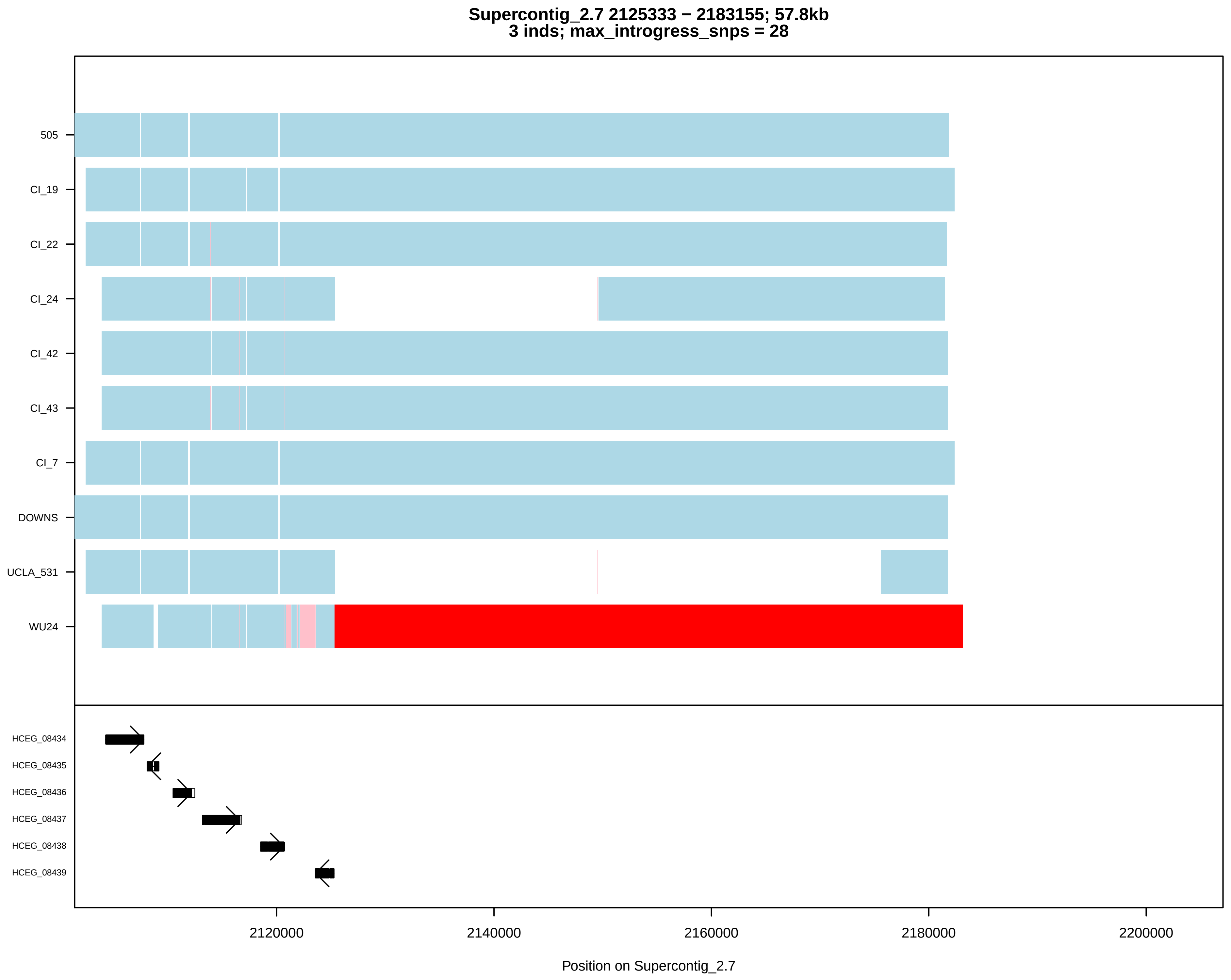
Supercontig_2.7 2125333 − 2183155; 57.8kb
3 inds; max_introgress_snps = 28
505
CI_19
CI_22
CI_24
CI_42
CI_43
CI_7
DOWNS
UCLA_531
WU24
HCEG_08434
HCEG_08435
HCEG_08436
HCEG_08437
HCEG_08438
HCEG_08439
2120000
2140000
2160000
2180000
2200000
Position on Supercontig_2.7

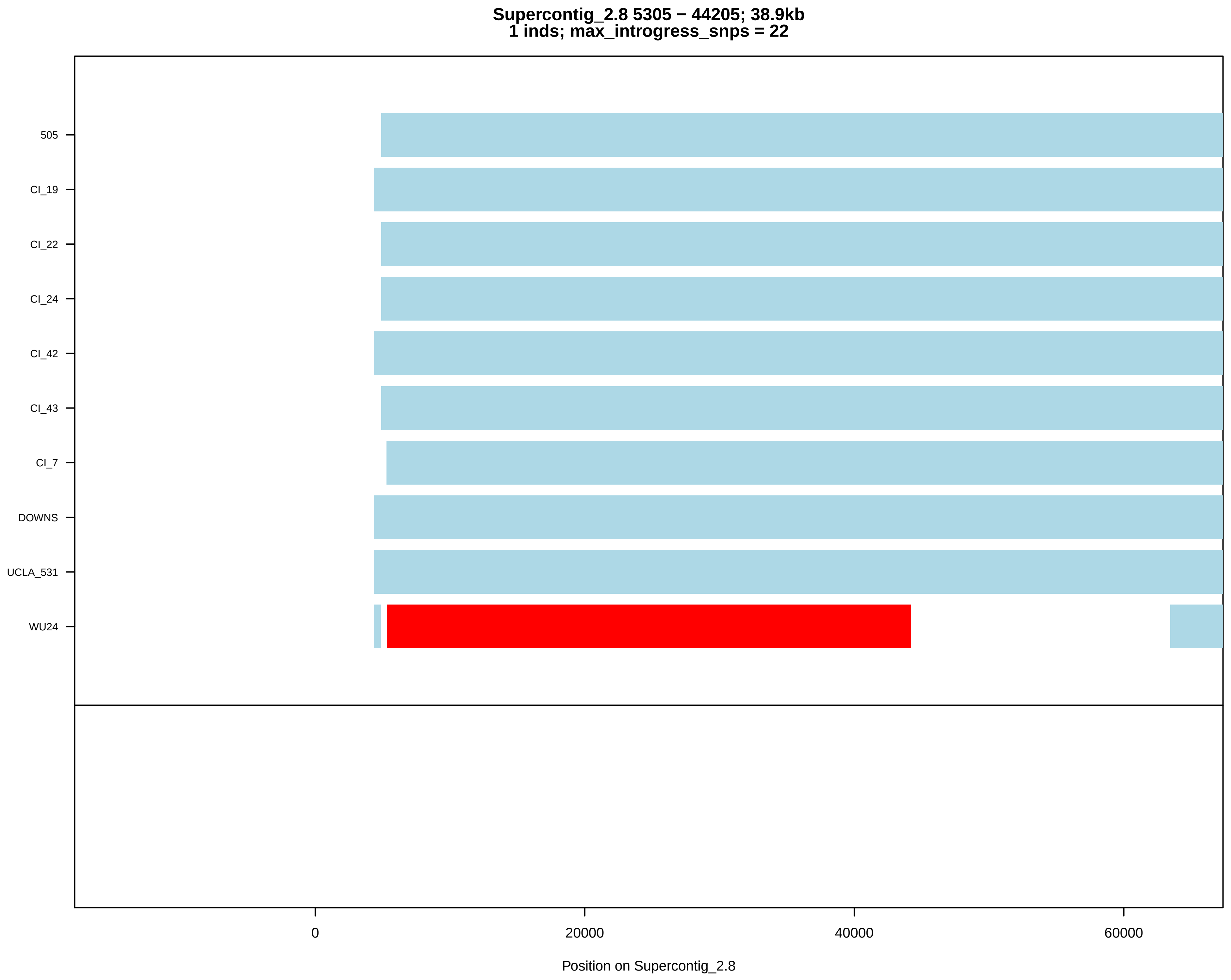

Supercontig_2.8 5305 − 44205; 38.9kb
1 inds; max_introgress_snps = 22
505
CI_19
CI_22
CI_24
CI_42
CI_43
CI_7
DOWNS
UCLA_531
WU24
0
20000
40000
60000
Position on Supercontig_2.8

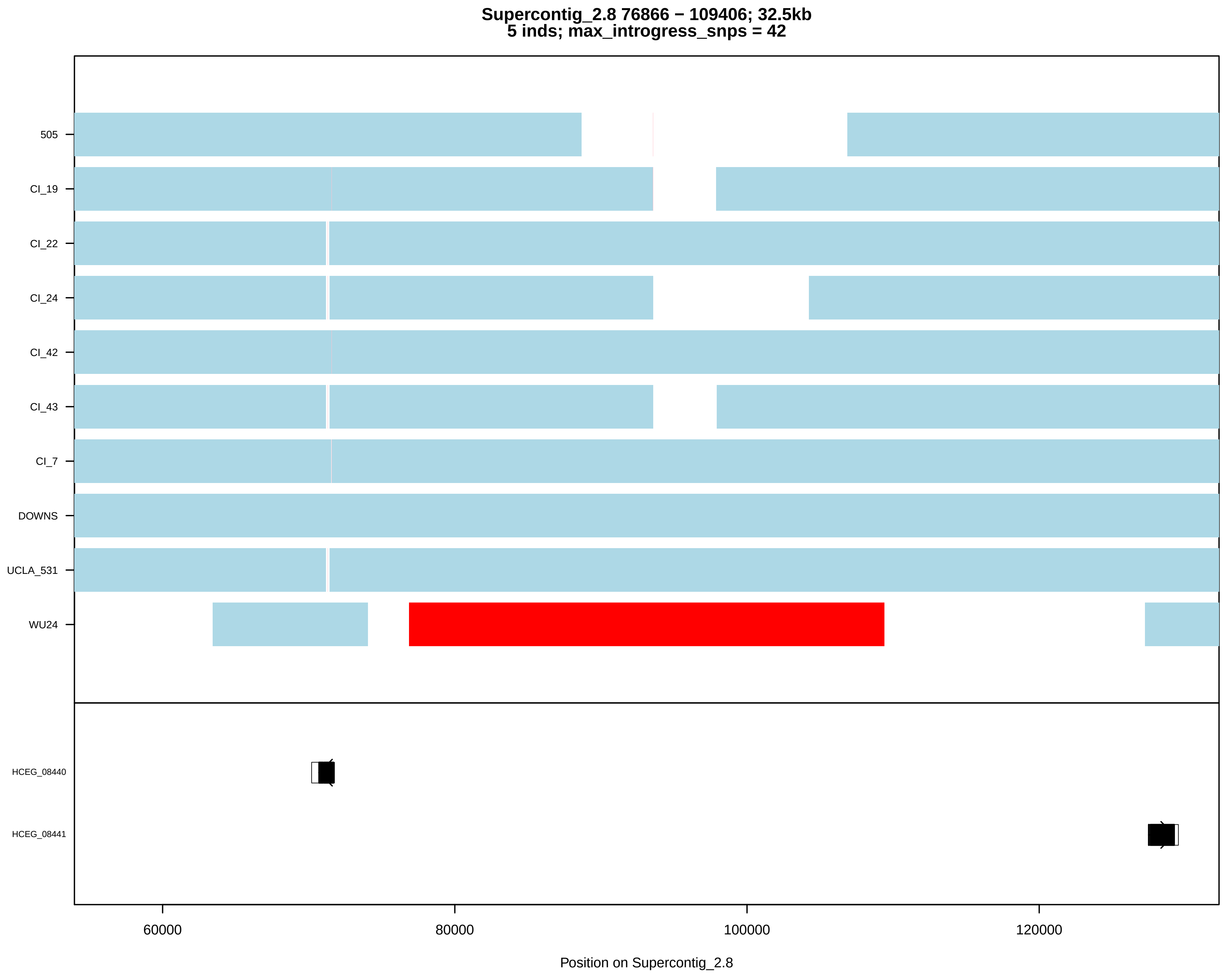
Supercontig_2.8 76866 − 109406; 32.5kb
5 inds; max_introgress_snps = 42
505
CI_19
CI_22
CI_24
CI_42
CI_43
CI_7
DOWNS
UCLA_531
WU24
HCEG_08440
HCEG_08441
60000
80000
100000
120000
Position on Supercontig_2.8

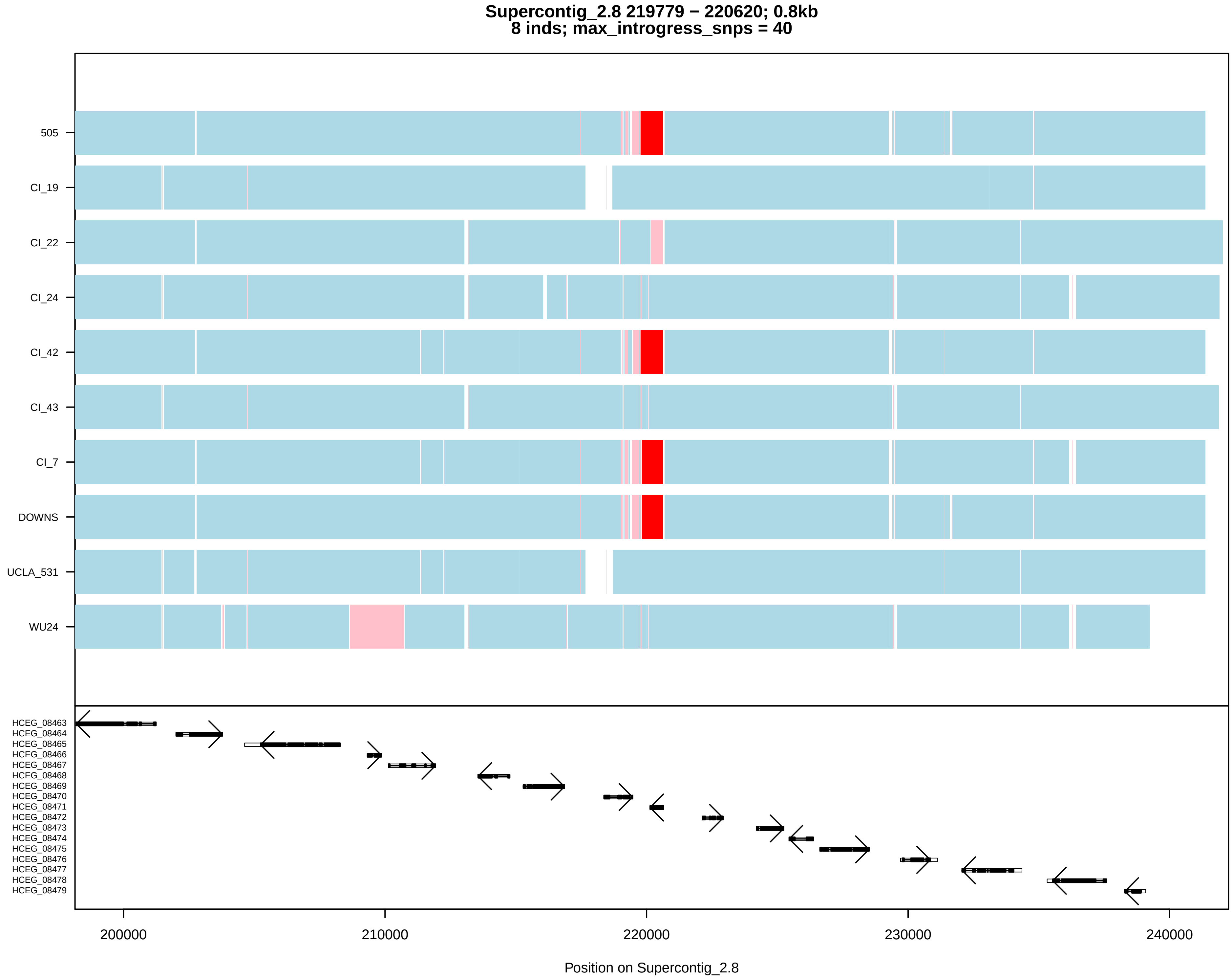

Supercontig_2.8 219779 − 220620; 0.8kb
8 inds; max_introgress_snps = 40
505
CI_19
CI_22
CI_24
CI_42
CI_43
CI_7
DOWNS
UCLA_531
WU24
HCEG_08463
HCEG_08464
HCEG_08465
HCEG_08466
HCEG_08467
HCEG_08468
HCEG_08469
HCEG_08470
HCEG_08471
HCEG_08472
HCEG_08473
HCEG_08474
HCEG_08475
HCEG_08476
HCEG_08477
HCEG_08478
HCEG_08479
200000
210000
220000
230000
240000
Position on Supercontig_2.8

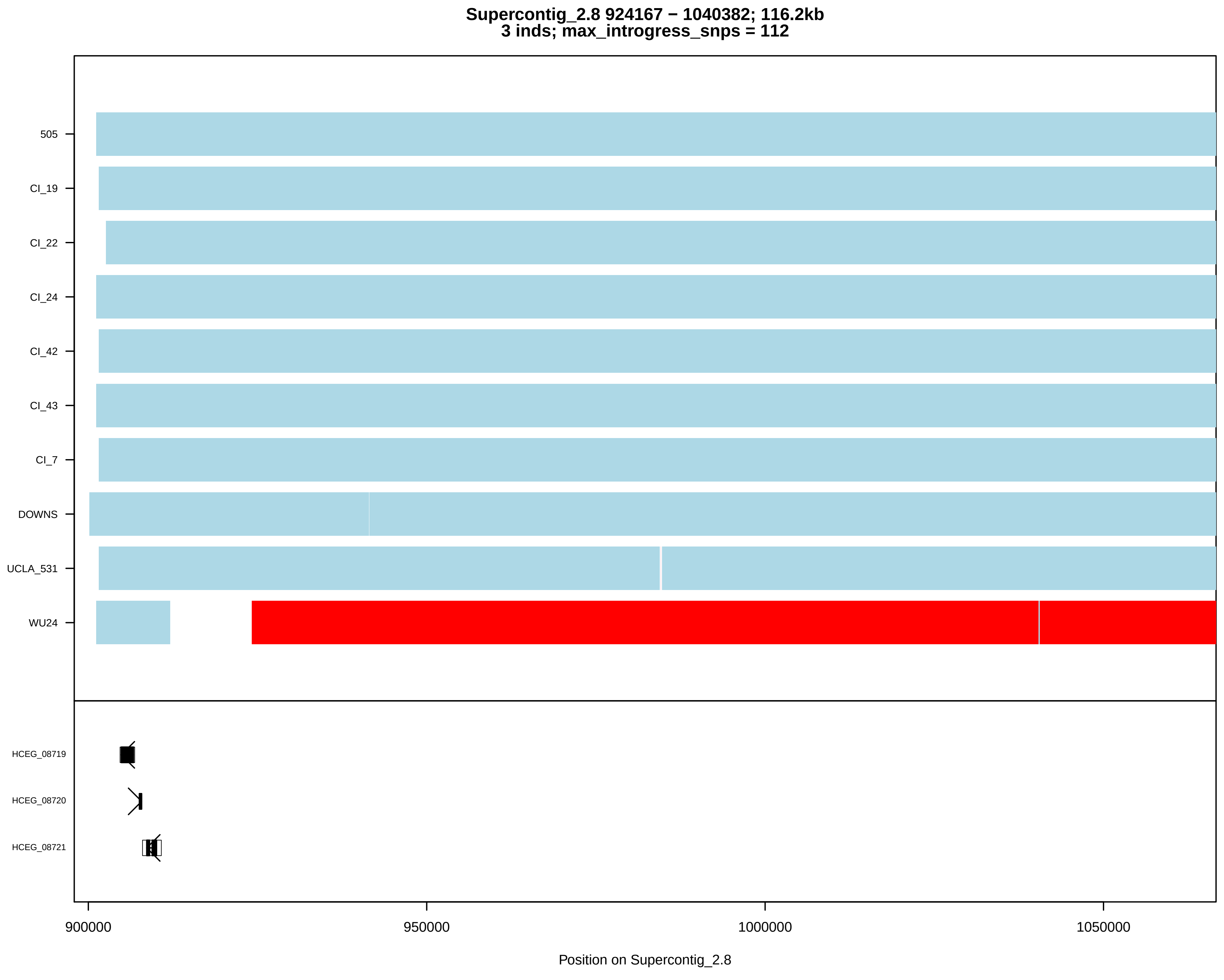

Supercontig_2.8 924167 − 1040382; 116.2kb
3 inds; max_introgress_snps = 112
505
CI_19
CI_22
CI_24
CI_42
CI_43
CI_7
DOWNS
UCLA_531
WU24
HCEG_08719
HCEG_08720
HCEG_08721
900000
950000
1000000
1050000
Position on Supercontig_2.8

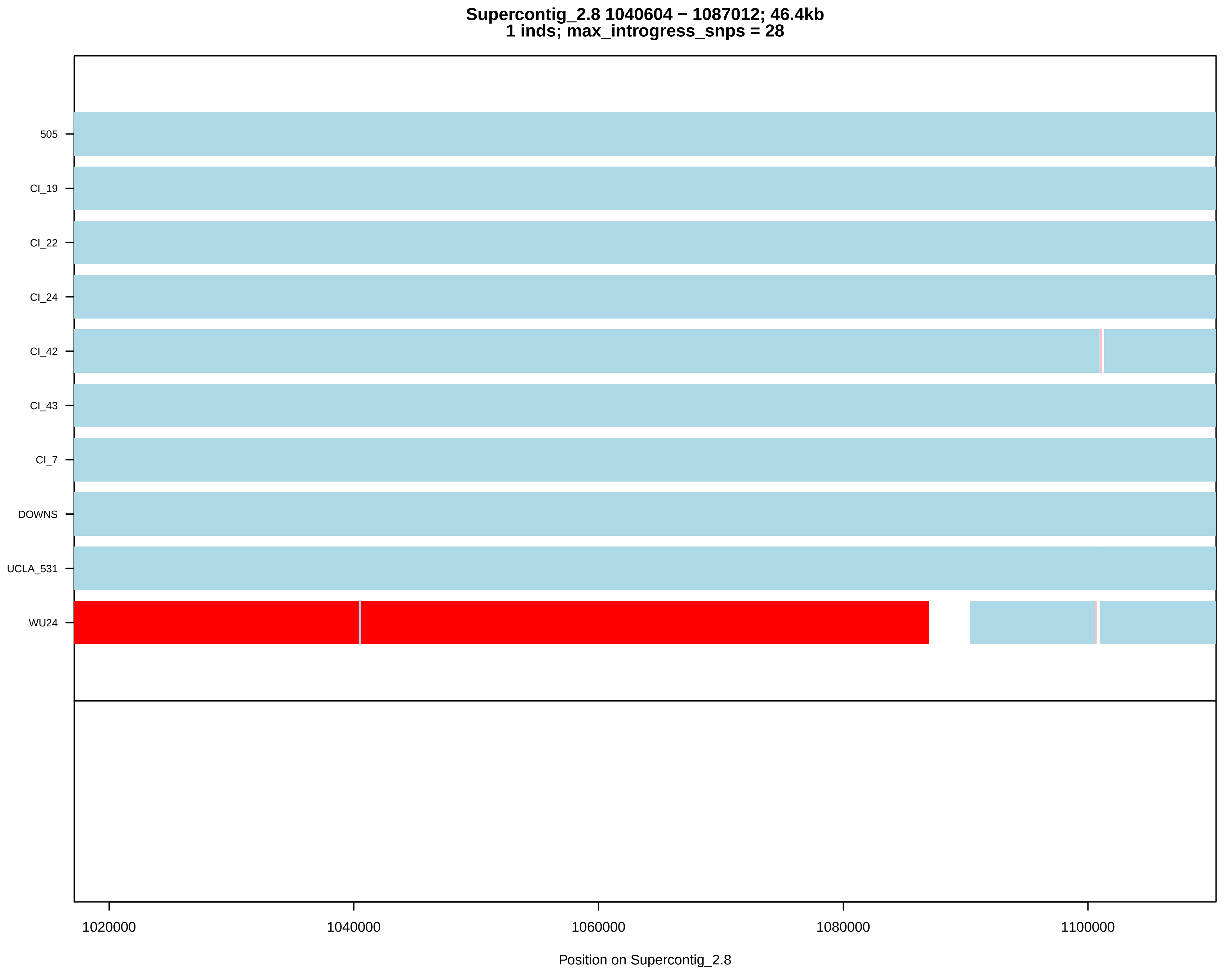
Supercontig_2.8 1040604 − 1087012; 46.4kb
1 inds; max_introgress_snps = 28
505
CI_19
CI_22
CI_24
CI_42
CI_43
CI_7
DOWNS
UCLA_531
WU24
1020000
1040000
1060000
1080000
1100000
Position on Supercontig_2.8

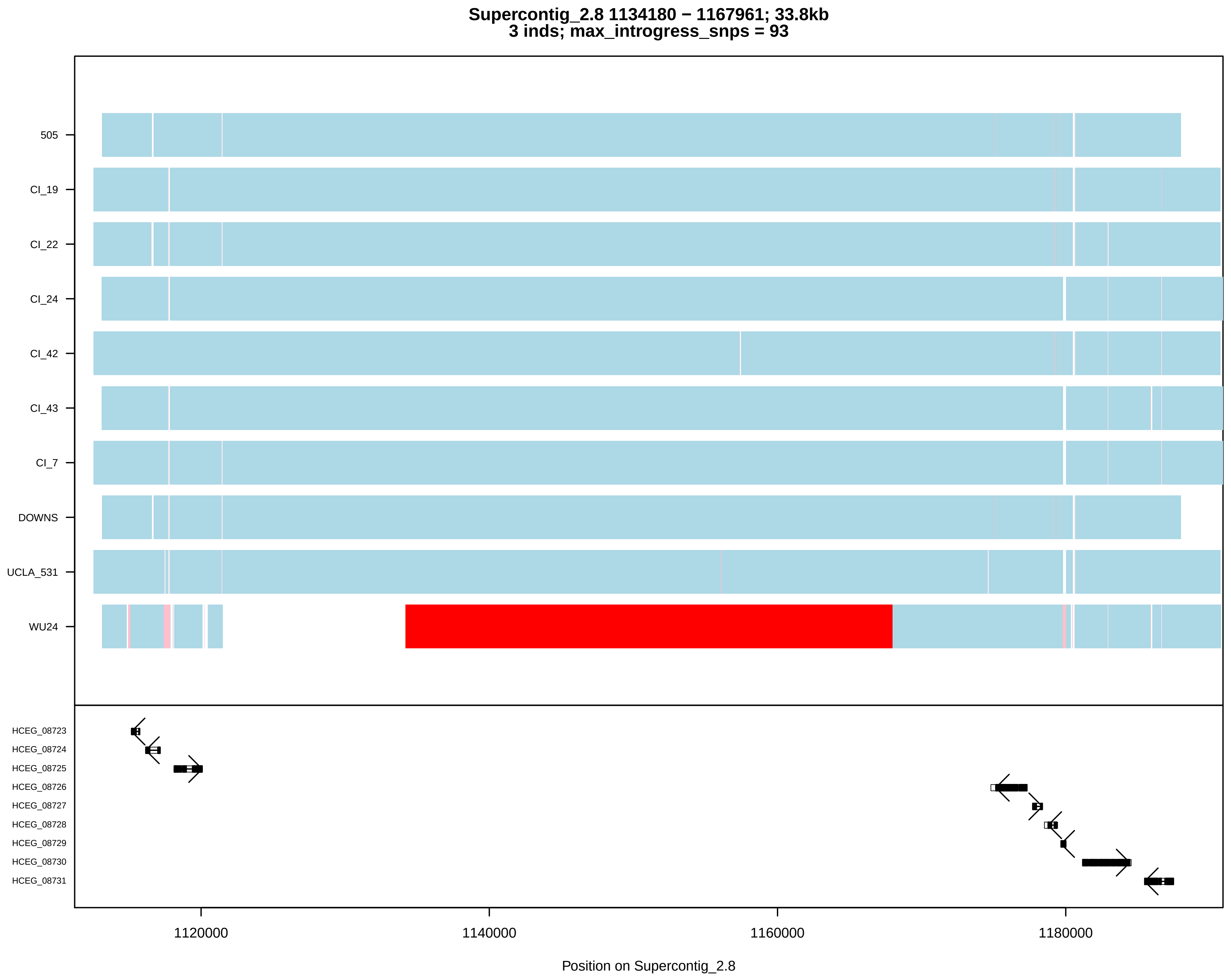
Supercontig_2.8 1134180 − 1167961; 33.8kb
3 inds; max_introgress_snps = 93
505
CI_19
CI_22
CI_24
CI_42
CI_43
CI_7
DOWNS
UCLA_531
WU24
HCEG_08723
HCEG_08724
HCEG_08725
HCEG_08726
HCEG_08727
HCEG_08728
HCEG_08729
HCEG_08730
HCEG_08731
1120000
1140000
1160000
1180000
Position on Supercontig_2.8

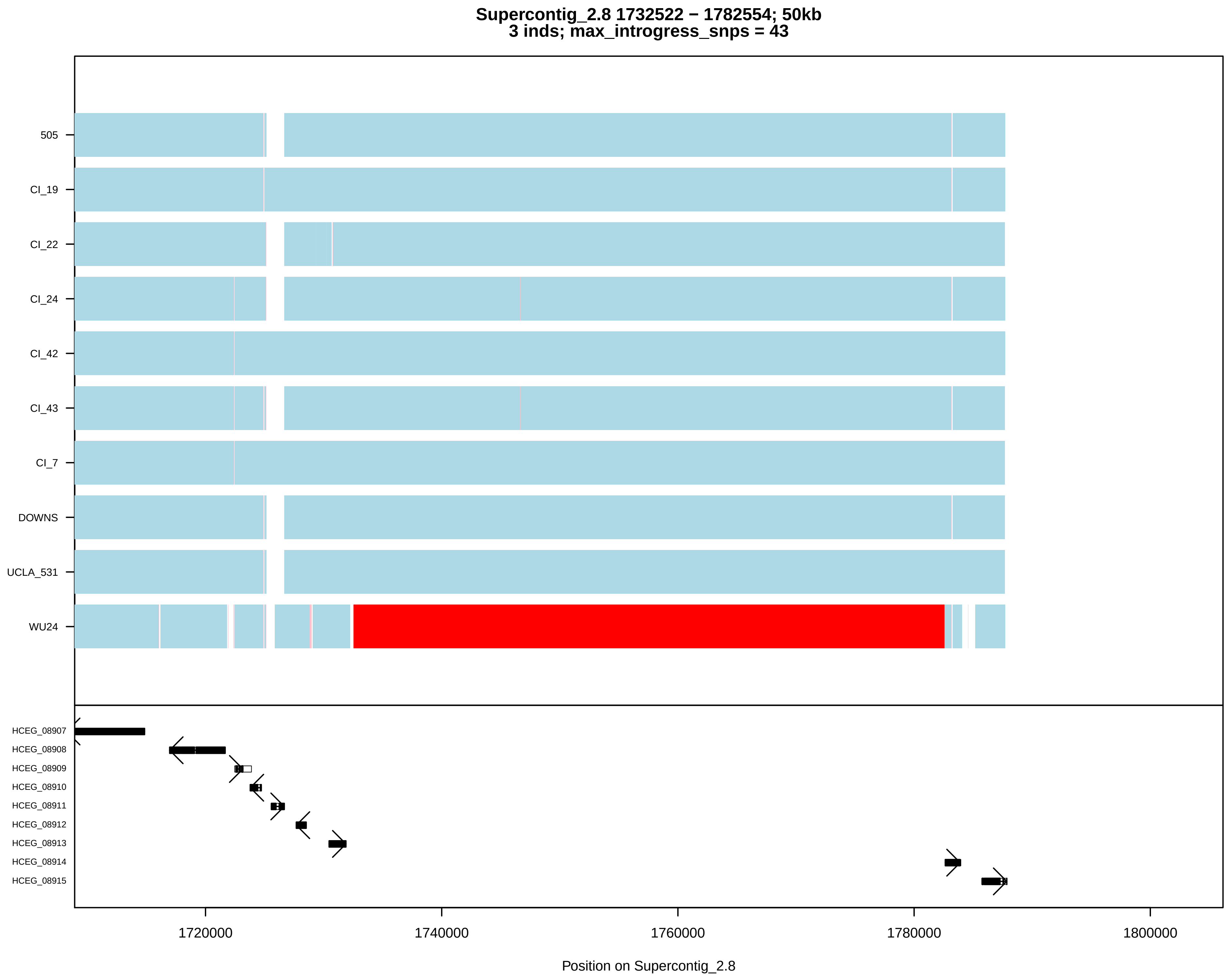

Supercontig_2.8 1732522 − 1782554; 50kb
3 inds; max_introgress_snps = 43
505
CI_19
CI_22
CI_24
CI_42
CI_43
CI_7
DOWNS
UCLA_531
WU24
HCEG_08907
HCEG_08908
HCEG_08909
HCEG_08910
HCEG_08911
HCEG_08912
HCEG_08913
HCEG_08914
HCEG_08915
1720000
1740000
1760000
1780000
1800000
Position on Supercontig_2.8

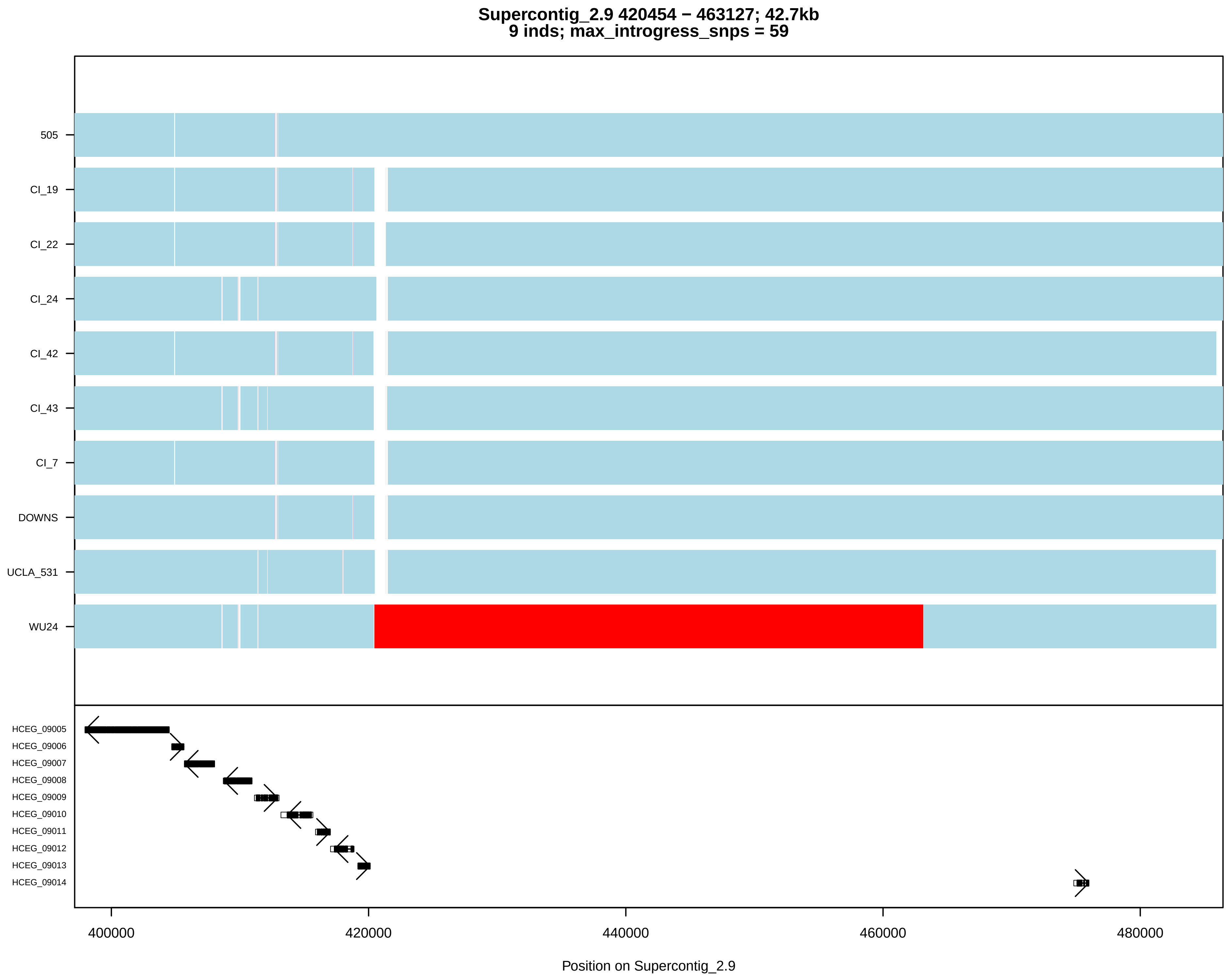
Supercontig_2.9 420454 − 463127; 42.7kb
9 inds; max_introgress_snps = 59
505
CI_19
CI_22
CI_24
CI_42
CI_43
CI_7
DOWNS
UCLA_531
WU24
HCEG_09005
HCEG_09006
HCEG_09007
HCEG_09008
HCEG_09009
HCEG_09010
HCEG_09011
HCEG_09012
HCEG_09013
HCEG_09014
400000
420000
440000
460000
480000
Position on Supercontig_2.9

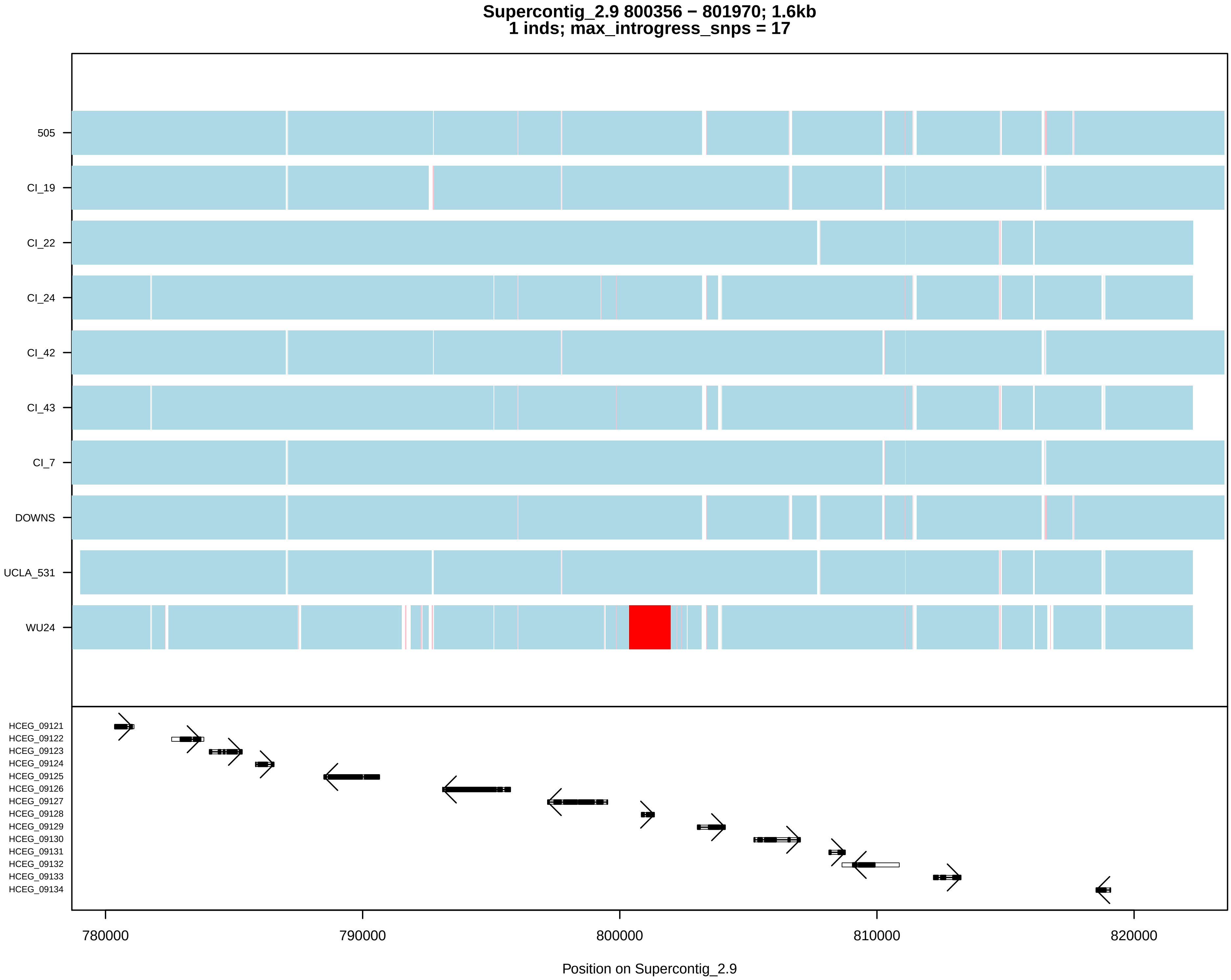

Supercontig_2.9 800356 − 801970; 1.6kb
1 inds; max_introgress_snps = 17
505
CI_19
CI_22
CI_24
CI_42
CI_43
CI_7
DOWNS
UCLA_531
WU24
HCEG_09121
HCEG_09122
HCEG_09123
HCEG_09124
HCEG_09125
HCEG_09126
HCEG_09127
HCEG_09128
HCEG_09129
HCEG_09130
HCEG_09131
HCEG_09132
HCEG_09133
HCEG_09134
780000
790000
800000
810000
820000
Position on Supercontig_2.9

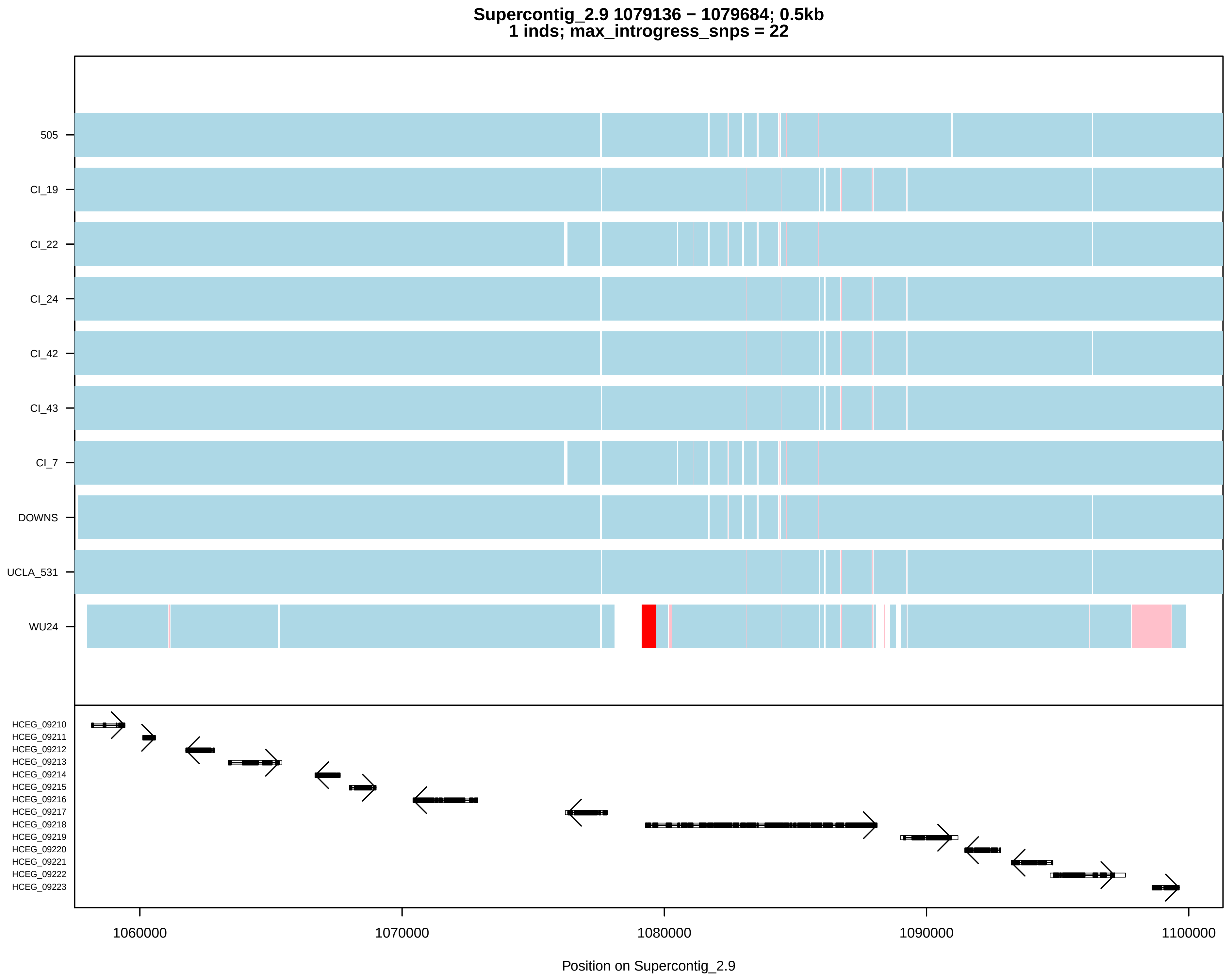
Supercontig_2.9 1079136 − 1079684; 0.5kb
1 inds; max_introgress_snps = 22
505
CI_19
CI_22
CI_24
CI_42
CI_43
CI_7
DOWNS
UCLA_531
WU24
HCEG_09210
HCEG_09211
HCEG_09212
HCEG_09213
HCEG_09214
HCEG_09215
HCEG_09216
HCEG_09217
HCEG_09218
HCEG_09219
HCEG_09220
HCEG_09221
HCEG_09222
HCEG_09223
1060000
1070000
1080000
1090000
1100000
Position on Supercontig_2.9

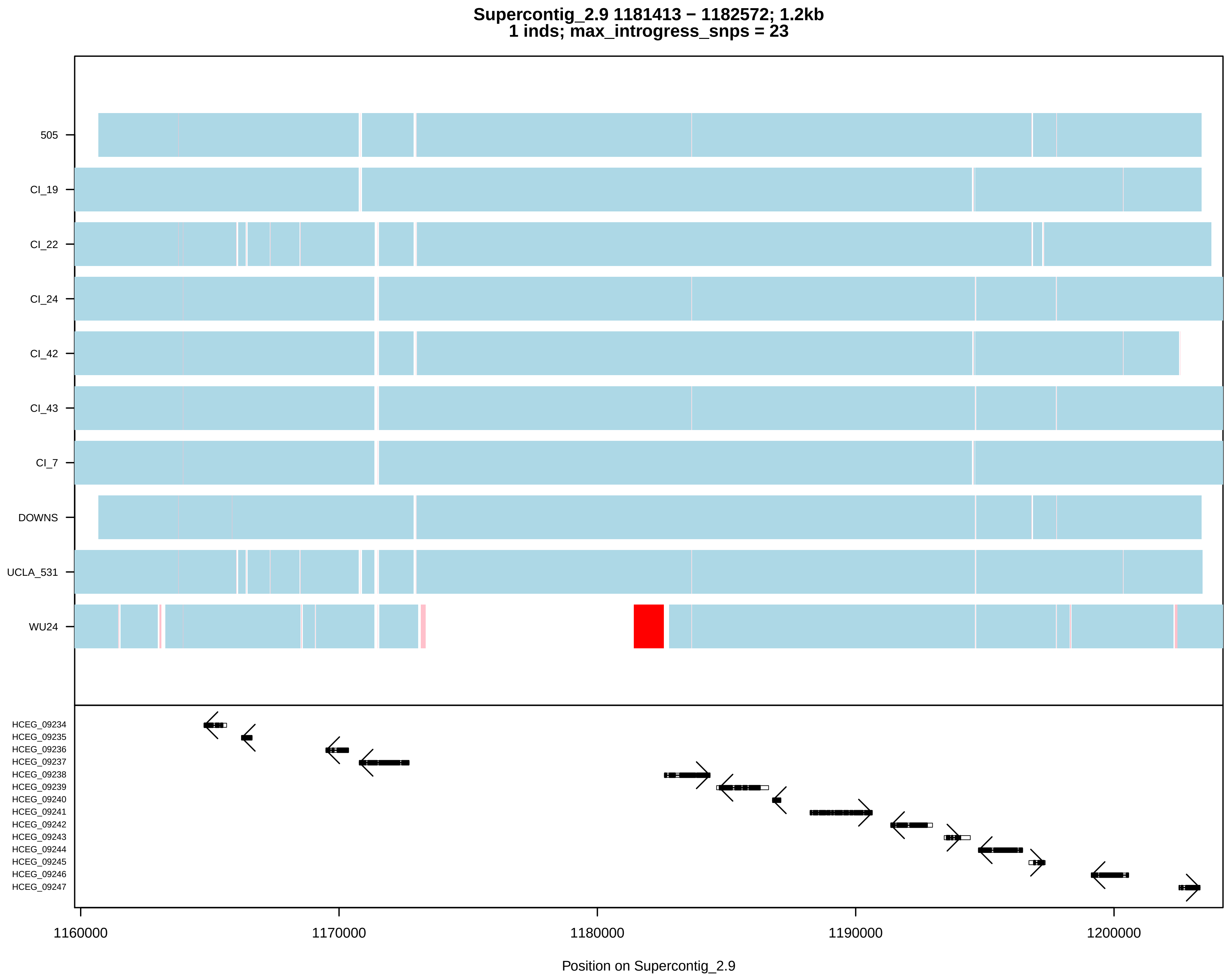
Supercontig_2.9 1181413 − 1182572; 1.2kb
1 inds; max_introgress_snps = 23
505
CI_19
CI_22
CI_24
CI_42
CI_43
CI_7
DOWNS
UCLA_531
WU24
HCEG_09234
HCEG_09235
HCEG_09236
HCEG_09237
HCEG_09238
HCEG_09239
HCEG_09240
HCEG_09241
HCEG_09242
HCEG_09243
HCEG_09244
HCEG_09245
HCEG_09246
HCEG_09247
1160000
1170000
1180000
1190000
1200000
Position on Supercontig_2.9

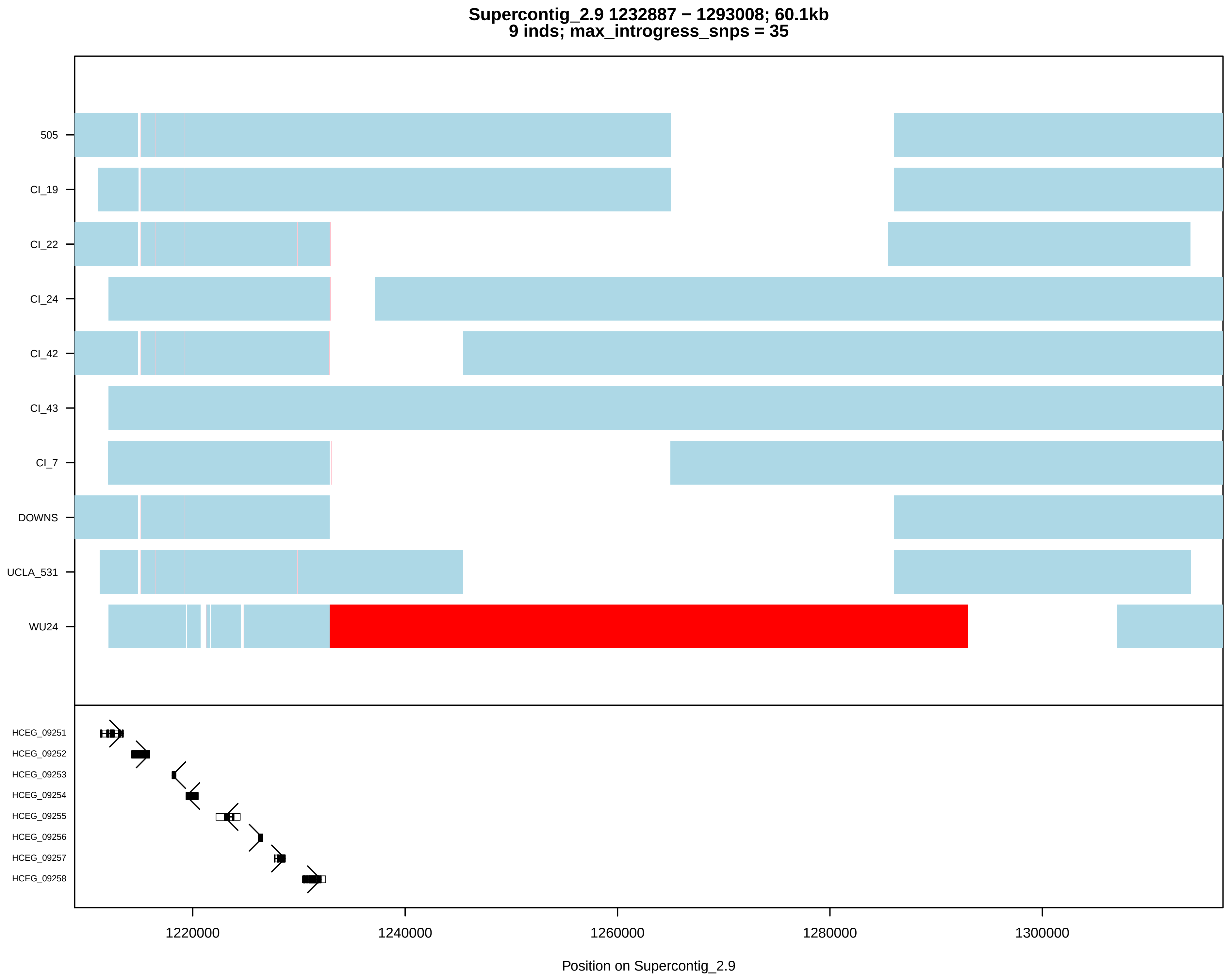
Supercontig_2.9 1232887 − 1293008; 60.1kb
9 inds; max_introgress_snps = 35
505
CI_19
CI_22
CI_24
CI_42
CI_43
CI_7
DOWNS
UCLA_531
WU24
HCEG_09251
HCEG_09252
HCEG_09253
HCEG_09254
HCEG_09255
HCEG_09256
HCEG_09257
HCEG_09258
1220000
1240000
1260000
1280000
1300000
Position on Supercontig_2.9

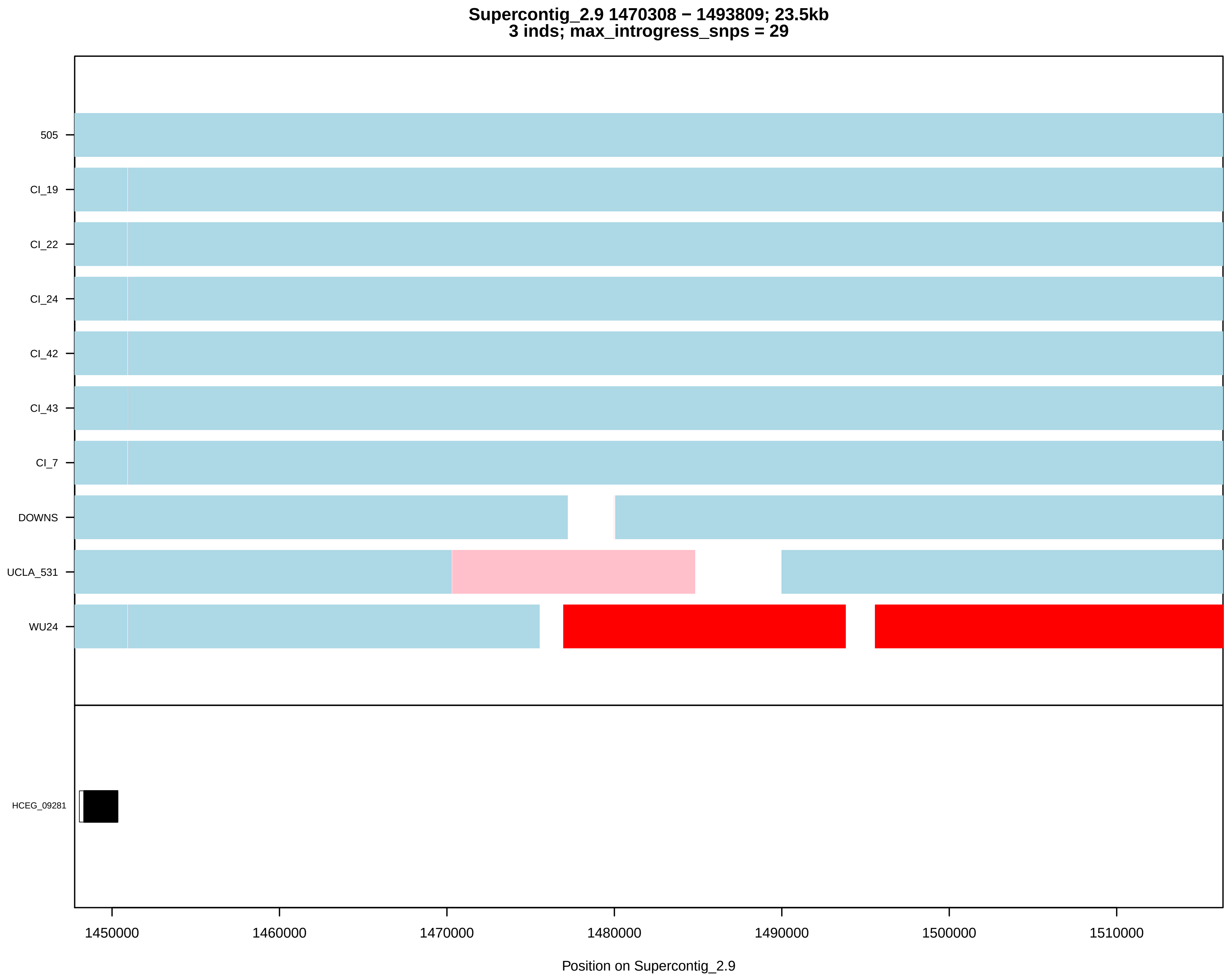
Supercontig_2.9 1470308 − 1493809; 23.5kb
3 inds; max_introgress_snps = 29
505
CI_19
CI_22
CI_24
CI_42
CI_43
CI_7
DOWNS
UCLA_531
WU24
HCEG_09281
1450000
1460000
1470000
1480000
1490000
1500000
1510000
Position on Supercontig_2.9

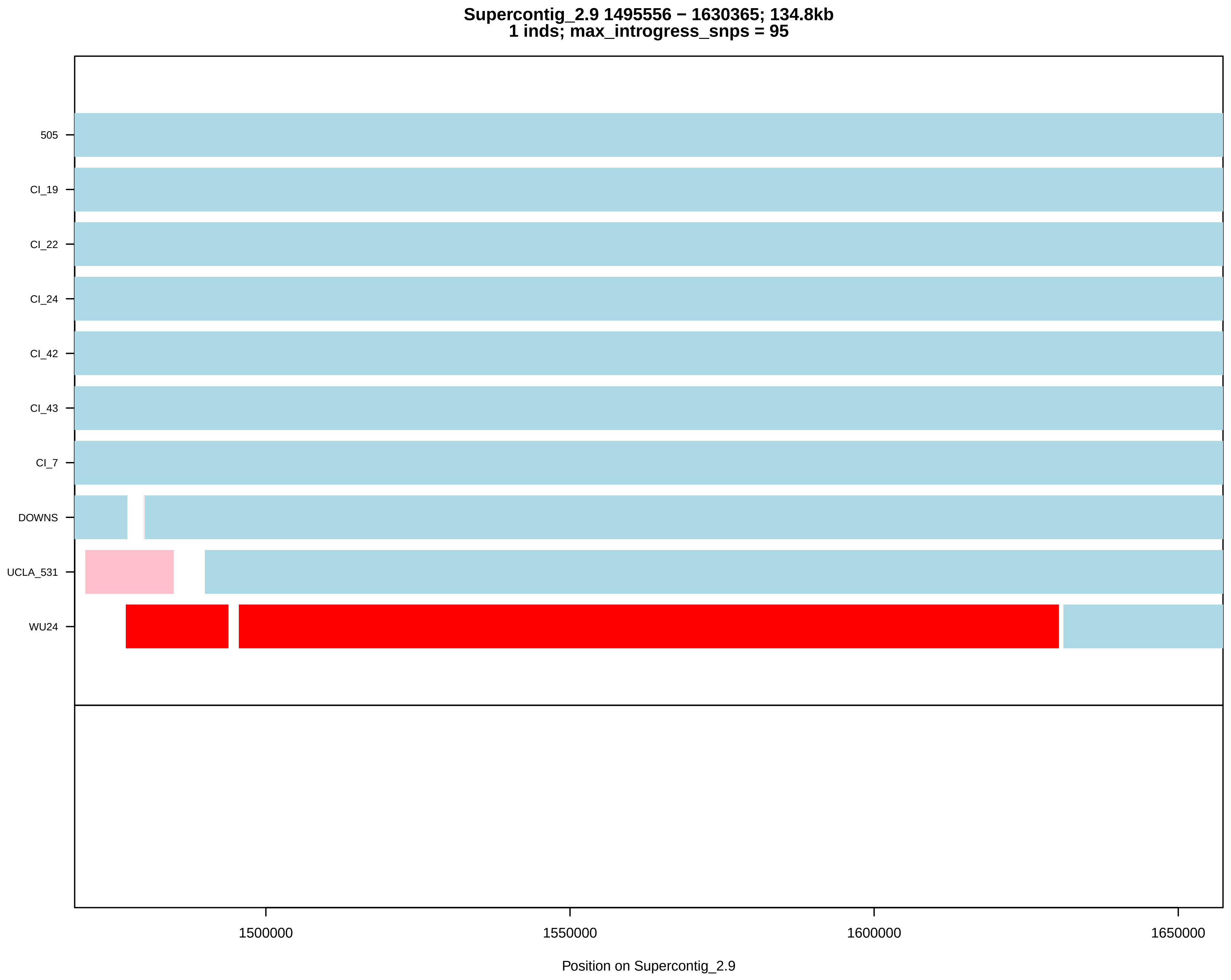

Supercontig_2.9 1495556 − 1630365; 134.8kb
1 inds; max_introgress_snps = 95
505
CI_19
CI_22
CI_24
CI_42
CI_43
CI_7
DOWNS
UCLA_531
WU24
1500000
1550000
1600000
1650000
Position on Supercontig_2.9
